# Supplementary material for: Biochemical neuroplasticity in the cerebellum after physical exercise: Systematic review and meta-analysis
Source: PLoS One. 2025 Aug 1;20(8):e0309259. doi: 10.1371/journal.pone.0309259 (PMC12316286; doi:10.1371/journal.pone.0309259)
Supplement: S2 File — (DOCX) [file pone.0309259.s002.docx]

***Supplementary Material 2***

**Title:** BIOCHEMICAL NEUROPLASTICITY IN THE CEREBELLUM AFTER PHYSICAL EXERCISE: SYSTEMATIC REVIEW AND META-ANALYSIS

1. **PICO Strategy**

**Question:** Is physical exercise able to reduce oxidative stress in the central nervous system?

**Population (P).** Experimental models with small rodents

**Intervention (I).** The interventions were performed on small rodents subjected to physical exercise with a protocol whose variables volume, intensity, and training frequency were well defined.

**Comparator (C).** We considered the comparison with small rodents not subjected to physical exercise.

**Outcome (O).** The main outcomes included studies that showed changes in the biochemistry of nervous tissue in the cerebellum of small rodents after physical exercise.

1. **Search Strategy**

| Units | MeSh | ENTRY TERM |
| --- | --- | --- |
| (P) – | Animal | Animal |
|  | Brain | Brain  Encephalon  Central Nervous Systems  Nervous System, Central  Nervous Systems, Central  Systems, Central Nervous  Cerebrospinal Axis  Axi, Cerebrospinal  Axis, Cerebrospinal  Cerebrospinal Axi |
|  | Exercise | Exercises  Physical Activity  Activities, Physical  Activity, Physical  Physical Activities  Exercise, Physical  Exercises, Physical  Physical Exercise  Physical Exercises  Acute Exercise  Acute Exercises  Exercise, Acute  Exercises, Acute  Exercise, Isometric  Exercises, Isometric  Isometric Exercises  Isometric Exercise  Exercise, Aerobic  Aerobic Exercise  Aerobic Exercises  Exercises, Aerobic  Exercise Training  Exercise Trainings  Training, Exercise  Trainings, Exercise |
|  | Physical Fitness | Fitness, Physical |
|  | Oxidative Stresses | Oxidative Stresses  Stress, Oxidative  Antioxidative Stress  Antioxidative Stresses  Stress, Antioxidative  Anti-oxidative Stress  Anti oxidative Stress  Anti-oxidative Stresses  Stress, Anti-oxidative  Oxidative Damage  Damage, Oxidative  Oxidative Damages  Oxidative Stress Injury  Injury, Oxidative Stress  Oxidative Stress Injuries  Stress Injury, Oxidative  Oxidative Injury  Injury, Oxidative  Oxidative Injuries  Oxidative Cleavage  Cleavage, Oxidative  Oxidative Cleavages  Oxidative DNA Damage  DNA Damage, Oxidative  Damage, Oxidative DNA  Oxidative DNA Damages  DNA Oxidative Damage  DNA Oxidative Damages  Damage, DNA Oxidative  Oxidative Damage, DNA  Oxidative and Nitrosative Stress  Oxidative Nitrative Stress  Nitrative Stress, Oxidative  Oxidative Nitrative Stresses  Stress, Oxidative Nitrative  Nitro-Oxidative Stress  Nitro Oxidative Stress  Nitro-Oxidative Stresses  Stress, Nitro-Oxidative  Stresses, Nitro-Oxidative |

**Search on Pub med - 864 19/04/2024**

("Exercise"[Title/Abstract] OR "Exercises"[Title/Abstract] OR "physical activity"[Title/Abstract] OR "activities physical"[Title/Abstract] OR "activity physical"[Title/Abstract] OR "physical activities"[Title/Abstract] OR "exercise physical"[Title/Abstract] OR "exercises physical"[Title/Abstract] OR "physical exercise"[Title/Abstract] OR "physical exercises"[Title/Abstract] OR "acute exercise"[Title/Abstract] OR "acute exercises"[Title/Abstract] OR "exercise acute"[Title/Abstract] OR "exercises acute"[Title/Abstract] OR "exercise isometric"[Title/Abstract] OR "exercises isometric"[Title/Abstract] OR "isometric exercises"[Title/Abstract] OR "isometric exercise"[Title/Abstract] OR "exercise aerobic"[Title/Abstract] OR "aerobic exercise"[Title/Abstract] OR "aerobic exercises"[Title/Abstract] OR "exercises aerobic"[Title/Abstract] OR "exercise training"[Title/Abstract] OR "exercise trainings"[Title/Abstract] OR "training exercise"[Title/Abstract] OR (("education"[MeSH Subheading] OR "education"[All Fields] OR "Training"[All Fields] OR "education"[MeSH Terms] OR "train"[All Fields] OR "train s"[All Fields] OR "trained"[All Fields] OR "training s"[All Fields] OR "Trainings"[All Fields] OR "trains"[All Fields]) AND "Exercise"[Title/Abstract]) OR "physical fitness"[Title/Abstract] OR "fitness physical"[Title/Abstract]) AND ("brain"[MeSH Terms] OR "brain"[All Fields] OR "brains"[All Fields] OR "brain s"[All Fields] OR "Encephalon"[Title/Abstract] OR "central nervous systems"[Title/Abstract] OR "nervous system central"[Title/Abstract] OR "nervous systems central"[Title/Abstract] OR "systems central nervous"[Title/Abstract] OR "cerebrospinal axis"[Title/Abstract] OR ("Axi"[All Fields] AND "Cerebrospinal"[Title/Abstract]) OR "axis cerebrospinal"[Title/Abstract] OR ("Cerebrospinal"[All Fields] AND "Axi"[Title/Abstract])) AND ("oxidative stresses"[Title/Abstract] OR "oxidative stresses"[Title/Abstract] OR "stress oxidative"[Title/Abstract] OR "antioxidative stress"[Title/Abstract] OR (("antioxidant s"[All Fields] OR "antioxidants"[Pharmacological Action] OR "antioxidants"[MeSH Terms] OR "antioxidants"[All Fields] OR "antioxidant"[All Fields] OR "antioxidating"[All Fields] OR "antioxidation"[All Fields] OR "Antioxidative"[All Fields] OR "antioxidatively"[All Fields] OR "antioxidatives"[All Fields] OR "antioxidizing"[All Fields]) AND "Stresses"[Title/Abstract]) OR "stress antioxidative"[Title/Abstract] OR "anti oxidative stress"[Title/Abstract] OR "anti oxidative stress"[Title/Abstract] OR "anti oxidative stresses"[Title/Abstract] OR "stress anti oxidative"[Title/Abstract] OR "oxidative damage"[Title/Abstract] OR "damage oxidative"[Title/Abstract] OR "oxidative damages"[Title/Abstract] OR "oxidative stress injury"[Title/Abstract] OR "injury oxidative stress"[Title/Abstract] OR "oxidative stress injuries"[Title/Abstract] OR "stress injury oxidative"[Title/Abstract] OR "oxidative injury"[Title/Abstract] OR "injury oxidative"[Title/Abstract] OR "oxidative injuries"[Title/Abstract] OR "oxidative cleavage"[Title/Abstract] OR "cleavage oxidative"[Title/Abstract] OR "oxidative cleavages"[Title/Abstract] OR "oxidative dna damage"[Title/Abstract] OR "dna damage oxidative"[Title/Abstract] OR "damage oxidative dna"[Title/Abstract] OR "oxidative dna damages"[Title/Abstract] OR "dna oxidative damage"[Title/Abstract] OR "dna oxidative damages"[Title/Abstract] OR "damage dna oxidative"[Title/Abstract] OR "oxidative damage dna"[Title/Abstract] OR ("Oxidative"[Title/Abstract] OR "nitrosative stress"[Title/Abstract]) OR "oxidative nitrative stress"[Title/Abstract] OR "nitrative stress oxidative"[Title/Abstract] OR "oxidative nitrative stresses"[Title/Abstract] OR "stress oxidative nitrative"[Title/Abstract] OR "nitro oxidative stress"[Title/Abstract] OR "nitro oxidative stress"[Title/Abstract] OR "nitro oxidative stresses"[Title/Abstract] OR "stress nitro oxidative"[Title/Abstract] OR (("Stress"[All Fields] OR "stressed"[All Fields] OR "Stresses"[All Fields] OR "stressful"[All Fields] OR "stressfulness"[All Fields] OR "stressing"[All Fields]) AND "Nitro-Oxidative"[Title/Abstract]))

**Search on Web of Science - 1147 19/04/2024**

TS=(oxidative stresses) OR TS=(oxidative stresses) OR TS=(stress oxidative) OR TS=(antioxidative stress) OR TS=(antioxidant) OR TS=(antioxidants) OR TS=(antioxidants) OR TS=(antioxidants) OR TS=(antioxidant) OR TS=(antioxidating) OR TS=(antioxidation) OR TS=(Antioxidative) OR TS=(antioxidatively) OR TS=(antioxidatives) OR TS=(antioxidizing) OR TS=(Stresses) OR TS=(stress antioxidative) OR TS=(anti oxidative stress) OR TS=(anti oxidative stress) OR TS=(anti oxidative stresses) OR TS=(stress anti oxidative) OR TS=(oxidative damage) OR TS=(damage oxidative) OR TS=(oxidative damages) OR TS=(oxidative stress injury) OR TS=(injury oxidative stress) OR TS=(oxidative stress injuries) OR TS=(stress injury oxidative) OR TS=(oxidative injury) OR TS=(injury oxidative) OR TS=(oxidative injuries) OR TS=(oxidative cleavage) OR TS=(cleavage oxidative) OR TS=(oxidative cleavages) OR TS=(oxidative dna damage) OR TS=(dna damage oxidative) OR TS=(damage oxidative dna) OR TS=(oxidative dna damages) OR TS=(dna oxidative damage) OR TS=(dna oxidative damages) OR TS=(damage dna oxidative) OR TS=(oxidative damage dna) OR TS=(Oxidative) OR TS=(nitrosative stress) OR TS=(oxidative nitrative stress) OR TS=(nitrative stress oxidative) OR TS=(oxidative nitrative stresses) OR TS=(stress oxidative nitrative) OR TS=(nitro oxidative stress) OR TS=(nitro oxidative stress) OR TS=(nitro oxidative stresses) OR TS=(stress nitro oxidative) OR TS=(Stress) OR TS=(stressed) OR TS=(Stresses) OR TS=(stressfulness) OR TS=(stressing) OR TS=(Nitro-Oxidative)

AND

TS=(Animals) OR TS=(Animalia ) OR TS=(Animal ) OR TS=(Metazoa)

AND

TS=(brain) OR TS=(brain) OR TS=(brains) OR TS=(brain) OR TS=(Encephalon) OR TS=(central nervous systems) OR TS=(nervous system central) OR TS=(nervous systems central) OR TS=(systems central nervous) OR TS=(cerebrospinal axis) OR TS=(Axi) OR TS=(Cerebrospinal) OR TS=(axis cerebrospinal) OR TS=(Cerebrospinal) OR TS=(Axi)

AND

TS=(Exercise) OR TS=(Exercises) OR TS=(physical activity) OR TS=(activities physical) OR TS=(activity physical) OR TS=(physical activities) OR TS=(exercise physical) OR TS=(exercises physical) OR TS=(physical exercise) OR TS=(physical exercises) OR TS=(acute exercise) OR TS=(acute exercises) OR TS=(exercise acute) OR TS=(exercises acute) OR TS=(exercise isometric) OR TS=(exercises isometric) OR TS=(isometric exercises) OR TS=(isometric exercise) OR TS=(exercise aerobic) OR TS=(aerobic exercise) OR TS=(aerobic exercises) OR TS=(exercises aerobic) OR TS=(exercise training) OR TS=(exercise trainings) OR TS=(training exercise) OR TS=(physical fitness) OR TS=(fitness physical)

**Search on ScienceDirect - 194 | 19/04/2024**

(Brain OR Encephalon OR “Central Nervous Systems”) AND (Exercise OR “Physical Activity” OR “Physical Fitness”) AND (“Oxidative Stress” OR “Oxidative Injury” OR “Oxidative Damage”)

**Search on Scopus - 902 | 19/04/2024**

( ( TITLE-ABS-KEY ( brain ) OR TITLE-ABS-KEY ( encephalon ) OR TITLE-ABS-KEY ( "central nervous systems" ) OR TITLE-ABS-KEY ( "nervous system, central" ) OR TITLE-ABS-KEY ( "nervous systems, central" ) OR TITLE-ABS-KEY ( "systems, central nervous" ) OR TITLE-ABS-KEY ( "cerebrospinal axis" ) OR TITLE-ABS-KEY ( "axi, cerebrospinal" ) OR TITLE-ABS-KEY ( "axis, cerebrospinal" ) OR TITLE-ABS-KEY ( "cerebrospinal axi" ) ) ) AND ( ( TITLE-ABS-KEY ( exercise ) OR TITLE-ABS-KEY ( exercises ) OR TITLE-ABS-KEY ( "physical activity" ) OR TITLE-ABS-KEY ( "activities, physical" ) OR TITLE-ABS-KEY ( "activity, physical" ) OR TITLE-ABS-KEY ( "physical activities" ) OR TITLE-ABS-KEY ( "exercise, physical" ) OR TITLE-ABS-KEY ( "exercises, physical" ) OR TITLE-ABS-KEY ( "physical exercise" ) OR TITLE-ABS-KEY ( "physical exercises" ) OR TITLE-ABS-KEY ( "acute exercise" ) OR TITLE-ABS-KEY ( "acute exercises" ) OR TITLE-ABS-KEY ( "exercise, acute" ) OR TITLE-ABS-KEY ( "exercises, acute" ) OR TITLE-ABS-KEY ( "exercise, isometric" ) OR TITLE-ABS-KEY ( "exercises, isometric" ) OR TITLE-ABS-KEY ( "isometric exercises" ) OR TITLE-ABS-KEY ( "isometric exercise" ) OR TITLE-ABS-KEY ( "exercise, aerobic" ) OR TITLE-ABS-KEY ( "aerobic exercise" ) OR TITLE-ABS-KEY ( "aerobic exercises" ) OR TITLE-ABS-KEY ( "exercises, aerobic" ) OR TITLE-ABS-KEY ( "exercise training" ) OR TITLE-ABS-KEY ( "exercise trainings" ) OR TITLE-ABS-KEY ( "training, exercise" ) OR TITLE-ABS-KEY ( "trainings, exercise" ) OR TITLE-ABS-KEY ( "fitness, physical" ) OR TITLE-ABS-KEY ( "physical fitness" ) ) ) AND ( ( TITLE-ABS-KEY ( "oxidative stress" ) OR TITLE-ABS-KEY ( "oxidative stresses" ) OR TITLE-ABS-KEY ( "stress, oxidative" ) OR TITLE-ABS-KEY ( "antioxidative stress" ) OR TITLE-ABS-KEY ( "antioxidative stresses" ) OR TITLE-ABS-KEY ( "stress, antioxidative" ) OR TITLE-ABS-KEY ( "anti-oxidative stress" ) OR TITLE-ABS-KEY ( "anti oxidative stress" ) OR TITLE-ABS-KEY ( "anti-oxidative stresses" ) OR TITLE-ABS-KEY ( "stress, anti-oxidative" ) OR TITLE-ABS-KEY ( "oxidative damage" ) OR TITLE-ABS-KEY ( "damage, oxidative" ) OR TITLE-ABS-KEY ( "oxidative damages" ) OR TITLE-ABS-KEY ( "oxidative stress injury" ) OR TITLE-ABS-KEY ( "injury, oxidative stress" ) OR TITLE-ABS-KEY ( "oxidative stress injuries" ) OR TITLE-ABS-KEY ( "stress injury, oxidative" ) OR TITLE-ABS-KEY ( "oxidative injury" ) OR TITLE-ABS-KEY ( "injury, oxidative" ) OR TITLE-ABS-KEY ( "oxidative injuries" ) OR TITLE-ABS-KEY ( "oxidative cleavage" ) OR TITLE-ABS-KEY ( "cleavage, oxidative" ) OR TITLE-ABS-KEY ( "oxidative cleavages" ) OR TITLE-ABS-KEY ( "oxidative dna damage" ) OR TITLE-ABS-KEY ( "dna damage, oxidative" ) OR TITLE-ABS-KEY ( "damage, oxidative dna" ) OR TITLE-ABS-KEY ( "oxidative dna damages" ) OR TITLE-ABS-KEY ( "dna oxidative damage" ) OR TITLE-ABS-KEY ( "dna oxidative damages" ) OR TITLE-ABS-KEY ( "damage, dna oxidative" ) OR TITLE-ABS-KEY ( "oxidative damage, dna" ) OR TITLE-ABS-KEY ( "oxidative nitrosative stress" ) OR TITLE-ABS-KEY ( "oxidative nitrative stress" ) OR TITLE-ABS-KEY ( "nitrative stress, oxidative" ) OR TITLE-ABS-KEY ( "oxidative nitrative stresses" ) OR TITLE-ABS-KEY ( "stress, oxidative nitrative" ) OR TITLE-ABS-KEY ( "nitro-oxidative stress" ) OR TITLE-ABS-KEY ( "nitro oxidative stress" ) OR TITLE-ABS-KEY ( "nitro-oxidative stresses" ) OR TITLE-ABS-KEY ( "stress, nitro-oxidative" ) OR TITLE-ABS-KEY ( "stresses, nitro-oxidative" ) ) ) AND ( TITLE-ABS-KEY ( animals ) OR TITLE-ABS-KEY ( animalia ) OR TITLE-ABS-KEY ( animal ) OR TITLE-ABS-KEY ( metazoa ) )

| **Title** | **Year** | **Authors** | **Notes** | **Motive** |
| --- | --- | --- | --- | --- |
| Perineuronal Nets: Plasticity, Protection, and Therapeutic Potential | 2019 | Reichelt, Amy C. and Hare, Dominic J. and Bussey, Timothy J. and Saksida, Lisa M. | Excluded | The article was excluded based on title and abstract screening as it did not meet the inclusion and exclusion criteria. |
| 88 - Effect of Acute Voluntary Exercise on Expression and Oxidative Modification of Low Density Lipoprotein Receptor-Related Protein 1 in Brain of Wild Type Mice as a Function of Age | 2017 | Boyd-Kimball, Debra and Gonczy, Katelyn and Lewis, Benjamin and Mason, Thomas | Included |  |
| Suppression of brain aging and neurodegenerative disorders by dietary restriction and environmental enrichment: molecular mechanisms | 2001 | Mattson, Mark P. and Duan, Wenzhen and Lee, Jaewon and Guo, Zhihong | Excluded | The article was excluded based on title and abstract screening as it did not meet the inclusion and exclusion criteria. |
| Voluntary exercise or amphetamine treatment, but not the combination, increases hippocampal brain-derived neurotrophic factor and synapsin I following cortical contusion injury in rats | 2008 | Griesbach, G.S. and Hovda, D.A. and Gomez-Pinilla, F. and Sutton, R.L. | Excluded | The article was excluded based on title and abstract screening as it did not meet the inclusion and exclusion criteria. |
| Effects of APOE4 on omega-3 brain metabolism across the lifespan | 2024 | Ebright, Brandon and Duro, Marlon V. and Chen, Kai and Louie, Stan and Yassine, Hussein N. | Excluded | The article was excluded based on title and abstract screening as it did not meet the inclusion and exclusion criteria. |
| Adaptation to oxidative challenge induced by chronic physical exercise prevents Na+,K+-ATPase activity inhibition after traumatic brain injury | 2009 | Lima, Frederico Diniz and Oliveira, Mauro Schneider and Furian, Ana Flávia and Souza, Mauren Assis and Rambo, Leonardo Magno and Ribeiro, Leandro Rodrigo and Silva, Luiz Fernando Almeida and Retamoso, Leandro Thies and Hoffmann, Maurício Scopel and Magni, Danieli Valnes and Pereira, Leticia and Fighera, Michele Rechia and Mello, Carlos Fernando and Royes, Luiz Fernando Freire | Excluded | The article was excluded based on title and abstract screening as it did not meet the inclusion and exclusion criteria. |
| Nutrition, sleep, physical exercise: Impact on mental health | 2016 | Rössler, W. | Excluded | The article was excluded based on title and abstract screening as it did not meet the inclusion and exclusion criteria. |
| In vivo magnetic resonance spectroscopy in chronic fatigue syndrome | 2004 | Chaudhuri, A and Behan, P.O | Excluded | The article was excluded based on title and abstract screening as it did not meet the inclusion and exclusion criteria. |
| Uric acid enhances longevity and endurance and protects the brain against ischemia | 2019 | Cutler, Roy G. and Camandola, Simonetta and Feldman, Neil H. and Yoon, Jeong Seon and Haran, James B. and Arguelles, Sandro and Mattson, Mark P. | Excluded | The article was excluded based on title and abstract screening as it did not meet the inclusion and exclusion criteria. |
| Regular voluntary exercise cures stress-induced impairment of cognitive function and cell proliferation accompanied by increases in cerebral IGF-1 and GST activity in mice | 2010 | Nakajima, Sanae and Ohsawa, Ikuroh and Ohta, Shigeo and Ohno, Makoto and Mikami, Toshio | Excluded | The article was excluded based on title and abstract screening as it did not meet the inclusion and exclusion criteria. |
| Grape powder prevents cognitive, behavioral, and biochemical impairments in a rat model of posttraumatic stress disorder | 2015 | Solanki, Naimesh and Alkadhi, Isam and Atrooz, Fatin and Patki, Gaurav and Salim, Samina | Excluded | The article was excluded based on title and abstract screening as it did not meet the inclusion and exclusion criteria. |
| Aerobic exercise regulates synaptic transmission and reactive oxygen species production in the paraventricular nucleus of spontaneously hypertensive rats | 2019 | Li, Cui and Li, Yan and Zhao, Ziqi and Lv, Yuanyuan and Gu, Boya and Zhao, Li | Excluded | The article was excluded based on title and abstract screening as it did not meet the inclusion and exclusion criteria. |
| Aerobic exercise attenuates LPS-induced cognitive dysfunction by reducing oxidative stress, glial activation, and neuroinflammation | 2024 | Choi, J.-W. and Jo, S.-W. and Kim, D.-E. and Paik, I.-Y. and Balakrishnan, R. | Included |  |
| Modulation of the neuronal network activity by P2X receptors and their involvement in neurological disorders | 2015 | Sáez-Orellana, F. and Godoy, P.A. and Silva-Grecchi, T. and Barra, K.M. and Fuentealba, J. | Excluded | The article was excluded based on title and abstract screening as it did not meet the inclusion and exclusion criteria. |
| Sugar in mind: Untangling a sweet and sour relationship beyond type 2 diabetes | 2019 | Cherbuin, Nicolas and Walsh, Erin I. | Excluded | The article was excluded based on title and abstract screening as it did not meet the inclusion and exclusion criteria. |
| DNP, mitochondrial uncoupling, and neuroprotection: A little dab'll do ya | 2017 | Geisler, John G. and Marosi, Krisztina and Halpern, Joshua and Mattson, Mark P. | Excluded | The article was excluded based on title and abstract screening as it did not meet the inclusion and exclusion criteria. |
| Effects of moderate exercise on cigarette smoke exposure-induced hippocampal oxidative stress values and neurological behaviors in mice | 2010 | Tuon, Talita and Valvassori, Samira S. and Lopes-Borges, Jéssica and Fries, Gabriel R. and Silva, Luciano A. and Kapczinski, Flavio and Quevedo, João and Pinho, Ricardo A. | Excluded | The article was excluded based on title and abstract screening as it did not meet the inclusion and exclusion criteria. |
| Catalase overexpression modulates metabolic parameters in a new ‘stress-less’ leptin-deficient mouse model | 2017 | Amos, Deborah L. and Robinson, Tanner and Massie, Melissa B. and Cook, Carla and Hoffsted, Alexis and Crain, Courtney and Santanam, Nalini | Excluded | The article was excluded based on title and abstract screening as it did not meet the inclusion and exclusion criteria. |
| The old guard: Age-related changes in microglia and their consequences | 2021 | Costa, Jéssica and Martins, Solange and Ferreira, Pedro A. and Cardoso, Ana M.S. and Guedes, Joana R. and Peça, João and Cardoso, Ana L. | Excluded | The article was excluded based on title and abstract screening as it did not meet the inclusion and exclusion criteria. |
| Pharmacological actions of melatonin in oxygen radical pathophysiology | 1997 | Reiter, Russel and Tang, Lei and Garcia, Joaquin J. and Muñoz-Hoyos, Antonio | Excluded | The article was excluded based on title and abstract screening as it did not meet the inclusion and exclusion criteria. |
| Long-term exercise treatment reduces oxidative stress in the hippocampus of aging rats | 2012 | Marosi, K. and Bori, Z. and Hart, N. and Sárga, L. and Koltai, E. and Radák, Z. and Nyakas, C. | Excluded | The article was excluded based on title and abstract screening as it did not meet the inclusion and exclusion criteria. |
| Effects of chrysin and early exercise on NF-κB signaling pathway during lung injury induced by focal cerebral ischemia-reperfusion in rats | 2018 | Huaping, P. | Excluded | The article was excluded based on title and abstract screening as it did not meet the inclusion and exclusion criteria. |
| Protective effect of exercise and sildenafil on acute stress and cognitive function | 2015 | Ozbeyli, Dilek and Gokalp, Ayse Gizem and Koral, Tolga and Ocal, Onur Yuksel and Dogan, Berkay and Akakin, Dilek and Yuksel, Meral and Kasimay, Ozgur | Excluded | The article was excluded based on title and abstract screening as it did not meet the inclusion and exclusion criteria. |
| Chapter 40 - Molecular Aspects of the Mediterranean Diet: Adiponectin, Brain Amyloid Precursor Protein, Apolipoprotein E and Caspase-3 mRNA | 2015 | El-Swefy, Sahar Elsayed and Atteia, Hebatallah Husseini and Preedy, Victor R. and Watson, Ronald Ross | Excluded | The article was excluded based on title and abstract screening as it did not meet the inclusion and exclusion criteria. |
| Aerobic and strength training induce changes in oxidative stress parameters and elicit modifications of various cellular components in skeletal muscle of aged rats | 2018 | Vilela, Thais Ceresér and Effting, Pauline Souza and dos Santos Pedroso, Giulia and Farias, Hemelin and Paganini, Lara and Rebelo Sorato, Helen and Nesi, Renata Tiescoski and de Andrade, Vanessa Moraes and de Pinho, Ricardo Aurino | Excluded | The article was excluded based on title and abstract screening as it did not meet the inclusion and exclusion criteria. |
| Carbonylated proteins in aging and exercise: immunoblot approaches | 1999 | Goto, S. and Nakamura, A. and Radak, Z. and Nakamoto, H. and Takahashi, R. and Yasuda, K. and Sakurai, Y. and Ishii, N. | Excluded | The article was excluded based on title and abstract screening as it did not meet the inclusion and exclusion criteria. |
| Voluntary exercise may engage proteasome function to benefit the brain after trauma | 2010 | Szabo, Zsofia and Ying, Zhe and Radak, Zsolt and Gomez-Pinilla, Fernando | Excluded | The article was excluded based on title and abstract screening as it did not meet the inclusion and exclusion criteria. |
| Treadmill exercise alleviated prenatal buprenorphine exposure-induced depression in rats | 2017 | Wu, Chih-Cheng and Hung, Chih-Jen and Lin, Shih-Yi and Wang, Ya-Yu and Chang, Cheng-Yi and Chen, Wen-Ying and Liao, Su-Lan and Raung, Shue-Ling and Yang, Ching-Ping and Chen, Chun-Jung | Excluded | The article was excluded based on title and abstract screening as it did not meet the inclusion and exclusion criteria. |
| Neuroprotective signaling and the aging brain: take away my food and let me run11Published on the World Wide Web on 24 August 2000. | 2000 | Mattson, Mark P. | Excluded | The article was excluded based on title and abstract screening as it did not meet the inclusion and exclusion criteria. |
| Negative effects of ultrafine particle exposure during forced exercise on the expression of Brain-Derived Neurotrophic Factor in the hippocampus of rats | 2012 | Bos, I. and De Boever, P. and Int Panis, L. and Sarre, S. and Meeusen, R. | Excluded | The article was excluded based on title and abstract screening as it did not meet the inclusion and exclusion criteria. |
| Role of SIRT3 in mitochondrial biology and its therapeutic implications in neurodegenerative disorders | 2023 | Mishra, Yogesh and Kaundal, Ravinder K | Excluded | The article was excluded based on title and abstract screening as it did not meet the inclusion and exclusion criteria. |
| Increased nuclear factor-κB and loss of p53 are key mechanisms in Myalgic Encephalomyelitis/chronic fatigue syndrome (ME/CFS) | 2012 | Morris, Gerwyn and Maes, Michael | Excluded | The article was excluded based on title and abstract screening as it did not meet the inclusion and exclusion criteria. |
| Aerobic exercises induce antioxidant pathways activation in rats | 2020 | Barghi, N and Bambaeichi, E and Rezaei-Tavirani, M and Khaledi, N | Included |  |
| Aging and low-intensity exercise change oxidative biomarkers in brain regions and radiographic measures of femur of wistar rats | 2020 | Silveira, E.M.S. and Santos, M.C.Q. and da Silva, T.C.B. and Silva, F.B.O. and Machado, C.V. and Elias, L. and Kolberg, A. and Kroth, A. and Partata, W.A. | Included |  |
| The effects of ozone exposure and sedentary lifestyle on neuronal microglia and mitochondrial bioenergetics of female Long-Evans rats | 2020 | Valdez, Matthew and Valdez, Joseph M. and Freeborn, Danielle and Johnstone, Andrew F.M. and Kodavanti, Prasada Rao S. | Excluded | The article was excluded based on title and abstract screening as it did not meet the inclusion and exclusion criteria. |
| 392 - Exercise and Probiotics Attenuate the Development of Alzheimer Disease in Mice | 2016 | Abraham, Dora and Radak, Zsolt and Feher, Janos | Excluded | The article was excluded based on title and abstract screening as it did not meet the inclusion and exclusion criteria. |
| Tianeptine exerts neuroprotective effects in the brain tissue of rats exposed to the chronic stress model | 2012 | Della, Franciela P. and Abelaira, Helena M. and Réus, Gislaine Z. and Antunes, Altamir R. and dos Santos, Maria Augusta B. and Zappelinni, Giovanni and Steckert, Amanda V. and Vuolo, Francieli and Galant, Letícia S. and Dal-Pizzol, Felipe and Kapczinski, Flávio and Quevedo, João | Excluded | The article was excluded based on title and abstract screening as it did not meet the inclusion and exclusion criteria. |
| Traumatic Brain Injury and Mitochondrial Dysfunction | 2015 | Hiebert, John B. and Shen, Qiuhua and Thimmesch, Amanda R. and Pierce, Janet D. | Excluded | The article was excluded based on title and abstract screening as it did not meet the inclusion and exclusion criteria. |
| COGNITIVE AND NEURAL MECHANISMS OF THE ACCELERATED AGING PHENOTYPE IN PTSD | 2019 | Friedman, Madeline and Salzman, Chloe and Neria, Yuval and Small, Scott and Brickman, Adam and Ciarleglio, Adam and Provenzano, Frank and Yehuda, Rachel and Flory, Janine and Szeszko, Philip and Rutherford, Bret | Excluded | The article was excluded based on title and abstract screening as it did not meet the inclusion and exclusion criteria. |
| Treadmill exercise restores high fat diet-induced disturbance of hippocampal neurogenesis through β2-adrenergic receptor-dependent induction of thioredoxin-1 and brain-derived neurotrophic factor | 2019 | Han, Tae-Kyung and Leem, Yea-Hyun and Kim, Hee-Sun | Excluded | The article was excluded based on title and abstract screening as it did not meet the inclusion and exclusion criteria. |
| The effects of aging in the hippocampus and cognitive decline | 2017 | Bettio, Luis E.B. and Rajendran, Luckshi and Gil-Mohapel, Joana | Excluded | The article was excluded based on title and abstract screening as it did not meet the inclusion and exclusion criteria. |
| Chapter 23 - Short chain fatty acids as epigenetic and metabolic regulators of neurocognitive health and disease | 2019 | Mihaylova, Maria M. and Stratton, Matthew S. and Ferguson, Bradley S. | Excluded | The article was excluded based on title and abstract screening as it did not meet the inclusion and exclusion criteria. |
| Taurine Supplementation for 48-Months Improved Glucose Tolerance and Changed ATP-Related Enzymes in Avians | 2023 | Neuwirth, L.S. and Gökhan, N. and Kaye, S. and Meehan, E.F. | Excluded | The article was excluded based on title and abstract screening as it did not meet the inclusion and exclusion criteria. |
| Electroacupuncture at ST25 corrected gut microbial dysbiosis and SNpc lipid peroxidation in Parkinson’s disease rats | 2024 | Hu, X.-M. and Song, L.-Z.-X. and Zhang, Z.-Z. and Ruan, X. and Li, H.-C. and Yu, Z. and Huang, L. | Excluded | The article was excluded based on title and abstract screening as it did not meet the inclusion and exclusion criteria. |
| Feeding the gut microbiome: impact on multiple sclerosis | 2023 | Bronzini, M. and Maglione, A. and Rosso, R. and Matta, M. and Masuzzo, F. and Rolla, S. and Clerico, M. | Excluded | The article was excluded based on title and abstract screening as it did not meet the inclusion and exclusion criteria. |
| Repeated Intravenous Administration of Human Neural Stem Cells Producing Choline Acetyltransferase Exerts Anti-Aging Effects in Male F344 Rats | 2023 | Kyung, J. and Kim, D. and Shin, K. and Park, D. and Hong, S.-C. and Kim, T.M. and Choi, E.-K. and Kim, Y.-B. | Excluded | The article was excluded based on title and abstract screening as it did not meet the inclusion and exclusion criteria. |
| A Narrative Review on REM Sleep Deprivation: A Promising Non-Pharmaceutical Alternative for Treating Endogenous Depression | 2023 | Crișan, C.A. and Milhem, Z. and Stretea, R. and Țața, I.-M. and Cherecheș, R.M. and Micluția, I.V. | Excluded | The article was excluded based on title and abstract screening as it did not meet the inclusion and exclusion criteria. |
| Myelin, aging, and physical exercise | 2023 | Graciani, A.L. and Gutierre, M.U. and Coppi, A.A. and Arida, R.M. and Gutierre, R.C. | Excluded | The article was excluded based on title and abstract screening as it did not meet the inclusion and exclusion criteria. |
| Nicorandil attenuates cognitive impairment after traumatic brain injury via inhibiting oxidative stress and inflammation: Involvement of BDNF and NGF | 2024 | Tu, Y. and Han, D. and Liu, Y. and Hong, D. and Chen, R. | Excluded | The article was excluded based on title and abstract screening as it did not meet the inclusion and exclusion criteria. |
| Mechanisms of the Beneficial Effects of Exercise on Brain-Derived Neurotrophic Factor Expression in Alzheimer’s Disease | 2023 | Jaberi, S. and Fahnestock, M. | Excluded | The article was excluded based on title and abstract screening as it did not meet the inclusion and exclusion criteria. |
| Combined caloric restriction and exercise provides greater metabolic and neurocognitive benefits than either as a monotherapy in obesity with or without estrogen deprivation | 2022 | Pratchayasakul, W. and Arunsak, B. and Suparan, K. and Sriwichaiin, S. and Chunchai, T. and Chattipakorn, N. and Chattipakorn, S.C. | Excluded | The article was excluded based on title and abstract screening as it did not meet the inclusion and exclusion criteria. |
| Depression and lifestyle: Focusing on nutrition, exercise, and their possible relevance to molecular mechanisms | 2023 | Kunugi, H. | Excluded | The article was excluded based on title and abstract screening as it did not meet the inclusion and exclusion criteria. |
| Kynurenine Pathway in Diabetes Mellitus—Novel Pharmacological Target? | 2023 | Kozieł, K. and Urbanska, E.M. | Excluded | The article was excluded based on title and abstract screening as it did not meet the inclusion and exclusion criteria. |
| Aerobic exercise training reduces deep-frying oil-induced apoptosis of hippocampal tissue by reducing oxidative stress in male rats | 2023 | Nikbin, S. and fardad, G. and Yazdi, S. and Bahman, M.H. and Ettefagh, P. and Khalegi, F. and Molaei, M. and Azizbeigi, K. and Guerra-Balic, M. and Montané, J. and Zargani, M. and Azarbayjani, M.A. | Excluded | The article was excluded based on title and abstract screening as it did not meet the inclusion and exclusion criteria. |
| Pulmonary hypertension alters blood flow distribution and impairs the hyperemic response in the rat diaphragm | 2023 | Schulze, K.M. and Horn, A.G. and Weber, R.E. and Behnke, B.J. and Poole, D.C. and Musch, T.I. | Excluded | The article was excluded based on title and abstract screening as it did not meet the inclusion and exclusion criteria. |
| Herbal Therapies for Weight Gain and Metabolic Abnormalities Induced by Atypical Antipsychotics: A Review Article | 2023 | Naghibi, H. and Salari, R. and Yousefi, M. and Khadem-Rezaiyan, M. and Ghanbarzadeh, M.R. and Bordbar, M.R.F. | Excluded | The article was excluded based on title and abstract screening as it did not meet the inclusion and exclusion criteria. |
| Inflammation, Autoimmunity and Neurodegenerative Diseases, Therapeutics and Beyond | 2024 | Garmendia, J.V. and De Sanctis, C.V. and Das, V. and Annadurai, N. and Hajduch, M. and De Sanctis, J.B. | Excluded | The article was excluded based on title and abstract screening as it did not meet the inclusion and exclusion criteria. |
| Alterations in antioxidant status, protein concentration, acetylcholinesterase, Na+, K+-ATPase, and Mg 2+-ATPase activities in rat brain after forced swimming | 2006 | Tsakiris, T. and Angelogianni, P. and Tesseromatis, C. and Tsakiris, S. and Tsopanakis, C. | Included |  |
| Undernutrition during development modulates endoplasmic reticulum stress genes in the hippocampus of juvenile rats: Involvement of oxidative stress | 2022 | Fernandes, M.S. and Pedroza, A.A. and de Andrade Silva, S.C. and de Lemos, M.D.T. and Bernardo, E.M. and Pereira, A.R. and dos Santos, T.M. and Lagranha, C. | Excluded | The article was excluded based on title and abstract screening as it did not meet the inclusion and exclusion criteria. |
| What Do Randomized Controlled Trials Inform Us About Potential Disease-Modifying Strategies for Parkinson’s Disease? | 2023 | Ong, W.-Y. and Leow, D.M.-K. and Herr, D.R. and Yeo, C.J.-J. | Excluded | The article was excluded based on title and abstract screening as it did not meet the inclusion and exclusion criteria. |
| Neuroprotective Effects of High-Intensity Interval Training through Neuroplastic Changes in a Restraint Stress-Induced Depression Model | 2023 | Hwang, D.-J. and Um, H.-S. and Choi, D.-H. and Cho, J.-Y. | Excluded | The article was excluded based on title and abstract screening as it did not meet the inclusion and exclusion criteria. |
| The dual and emerging role of physical exercise-induced TFEB activation in the protection against Alzheimer's disease | 2023 | Morais, G.P. and de Sousa Neto, I.V. and Marafon, B.B. and Ropelle, E.R. and Cintra, D.E. and Pauli, J.R. and Silva, A.S.R.D. | Excluded | The article was excluded based on title and abstract screening as it did not meet the inclusion and exclusion criteria. |
| Ketone bodies in cell physiology and cancer | 2024 | Giuliani, G. and Longo, V.D. | Excluded | The article was excluded based on title and abstract screening as it did not meet the inclusion and exclusion criteria. |
| Oxidative stress marker changes and cognitive decline prevention in Pb contaminated mice by Crocus sativus L. Supplementation and interval training | 2024 | Noruzi, S. and Meshkati, Z. and Nasiri, R. | Excluded | The article was excluded based on title and abstract screening as it did not meet the inclusion and exclusion criteria. |
| Exercise suppresses neuroinflammation for alleviating Alzheimer’s disease | 2023 | Wang, M. and Zhang, H. and Liang, J. and Huang, J. and Chen, N. | Excluded | The article was excluded based on title and abstract screening as it did not meet the inclusion and exclusion criteria. |
| Exosome-based crosstalk in glaucoma pathogenesis: a focus on oxidative stress and neuroinflammation | 2023 | Wang, L. and Wei, X. | Excluded | The article was excluded based on title and abstract screening as it did not meet the inclusion and exclusion criteria. |
| Cordyceps militaris acidic polysaccharides improve learning and memory impairment in mice with exercise fatigue through the PI3K/NRF2/HO-1 signalling pathway | 2023 | Bai, L. and Tan, C. and Ren, J. and Liu, J. and Zou, W. and Liu, G. and Sheng, Y. | Excluded | The article was excluded based on title and abstract screening as it did not meet the inclusion and exclusion criteria. |
| Early Intensive Neurorehabilitation in Traumatic Peripheral Nerve Injury—State of the Art | 2024 | Gouveia, D. and Cardoso, A. and Carvalho, C. and Oliveira, A.C. and Almeida, A. and Gamboa, Ó. and Lopes, B. and Coelho, A. and Alvites, R. and Varejão, A.S. and Maurício, A.C. and Ferreira, A. and Martins, Â. | Excluded | The article was excluded based on title and abstract screening as it did not meet the inclusion and exclusion criteria. |
| The Neuropathological Impacts of COVID-19: Challenges and Alternative Treatment Options for Alzheimer’s Like Brain Changes on Severely SARS-CoV-2 Infected Patients | 2023 | Rahman, M.R. and Akter, R. and Neelotpol, S. and Mayesha, I.I. and Afrose, A. | Excluded | The article was excluded based on title and abstract screening as it did not meet the inclusion and exclusion criteria. |
| Irisin: An unveiled bridge between physical exercise and a healthy brain | 2024 | Sadier, N.S. and El Hajjar, F. and Al Sabouri, A.A.K. and Abou-Abbas, L. and Siomava, N. and Almutary, A.G. and Tambuwala, M.M. | Excluded | The article was excluded based on title and abstract screening as it did not meet the inclusion and exclusion criteria. |
| Treadmill exercise can regulate the redox balance in the livers of APP/PS1 mice and reduce LPS accumulation in their brains through the gut-liver-kupffer cell axis | 2024 | Yuan, S. and Wang, Y. and Yang, J. and Tang, Y. and Wu, W. and Meng, X. and Jian, Y. and Lei, Y. and Liu, Y. and Tang, C. and Zhao, Z. and Zhao, F. and Liu, W. | Excluded | The article was excluded based on title and abstract screening as it did not meet the inclusion and exclusion criteria. |
| Impact of physical activity on brain oxidative metabolism and intrinsic capacities in young swiss mice fed a high fat diet | 2023 | Viguier, C. and Bullich, S. and Botella, M. and Fasseu, L. and Alfonso, A. and Rekik, K. and Gauzin, S. and Guiard, B.P. and Davezac, N. | Excluded | The article was excluded based on title and abstract screening as it did not meet the inclusion and exclusion criteria. |
| Exercise attenuates mitochondrial autophagy and neuronal degeneration in MPTP induced Parkinson’s disease by regulating inflammatory pathway | 2023 | Li, Z. and Lv, H. and Cui, X. and Di, W. and Cheng, X. and Liu, J. and Tripathi, A.S. | Excluded | The article was excluded based on title and abstract screening as it did not meet the inclusion and exclusion criteria. |
| Aerobic Treadmill Exercise Upregulates Epidermal Growth Factor Levels and Improves Learning and Memory in d-galactose-Induced Aging in a Mouse Model | 2023 | Guo, C. and Kong, X. and Fan, Y. and Zhang, R. | Excluded | The article was excluded based on title and abstract screening as it did not meet the inclusion and exclusion criteria. |
| Quantification and Proteomic Characterization of β-Hydroxybutyrylation Modification in the Hearts of AMPKα2 Knockout Mice | 2023 | Ding, W.-J. and Li, X.-H. and Tang, C.-M. and Yang, X.-C. and Sun, Y. and Song, Y.-P. and Ling, M.-Y. and Yan, R. and Gao, H.-Q. and Zhang, W.-H. and Yu, N. and Feng, J.-C. and Zhang, Z. and Xing, Y.-Q. | Excluded | The article was excluded based on title and abstract screening as it did not meet the inclusion and exclusion criteria. |
| Mitigation of aircraft noise-induced vascular dysfunction and oxidative stress by exercise, fasting, and pharmacological α1AMPK activation: molecular proof of a protective key role of endothelial α1AMPK against environmental noise exposure | 2023 | Kvandová, M. and Rajlic, S. and Stamm, P. and Schmal, I. and Mihaliková, D. and Kuntic, M. and Bayo Jimenez, M.T. and Hahad, O. and Kollárová, M. and Ubbens, H. and Strohm, L. and Frenis, K. and Duerr, G.D. and Foretz, M. and Viollet, B. and Ruan, Y. and Jiang, S. and Tang, Q. and Kleinert, H. and Rapp, S. and Gericke, A. and Schulz, E. and Oelze, M. and Keaney, J.F. and Daiber, A. and Kröller-Schön, S. and Jansen, T. and Münzel, T. | Excluded | The article was excluded based on title and abstract screening as it did not meet the inclusion and exclusion criteria. |
| Effect of resistance and endurance training with ursolic acid on oxidative stress and cognitive impairment in hippocampal tissue in HFD/STZ-induced aged diabetic rats | 2023 | Alizade, S. and Faramarzi, M. and Banitalebi, E. and Saghaei, E. | Excluded | The article was excluded based on title and abstract screening as it did not meet the inclusion and exclusion criteria. |
| Aerobic exercise combined with chlorogenic acid exerts neuroprotective effects and reverses cognitive decline in Alzheimer’s disease model mice (APP/PS1) via the SIRT1/ /PGC-1α/PPARγ signaling pathway | 2023 | Shi, D. and Hao, Z. and Qi, W. and Jiang, F. and Liu, K. and Shi, X. | Excluded | The article was excluded based on title and abstract screening as it did not meet the inclusion and exclusion criteria. |
| Effect of Various Pathological Conditions on Nitric Oxide Level and L-Citrulline Uptake in Motor Neuron-Like (NSC-34) Cell Lines | 2024 | Gautam, S. and Latif, S. and Kang, Y.-S. | Excluded | The article was excluded based on title and abstract screening as it did not meet the inclusion and exclusion criteria. |
| Physical exercise and traumatic brain injury: is it question of time? | 2024 | Mastrorilli, V. and Vecchioli, S.F. | Excluded | The article was excluded based on title and abstract screening as it did not meet the inclusion and exclusion criteria. |
| Effect of Induced Pulmonary Arterial Hypertension on Testicular Parameters of Wistar Rats Subjected to Resistance Exercise Training | 2023 | Guimarães-Ervilha, L.O. and Soares, L.L. and Bento, I.D.S.P. and Carvalho, R.P.R. and Lima, T.I. and Dias, F.C.R. and Bastos, D.S.S. and Cossoin, J.F.S. and Souza, A.C.F. and Reis, E.C.C. and Serrão, J.E. and Natali, A.J. and Machado-Neves, M. | Excluded | The article was excluded based on title and abstract screening as it did not meet the inclusion and exclusion criteria. |
| Therapeutic approaches to non-motor symptoms of parkinson's disease: A current update on preclinical evidence | 2023 | Weerasinghe-Mudiyanselage, P.D.E. and Kang, S. and Kim, J.-S. and Moon, C. | Excluded | The article was excluded based on title and abstract screening as it did not meet the inclusion and exclusion criteria. |
| Inflammation and mitochondrial dysfunction in affective disorders-novel understandings, novel treatments? | 2023 | Skokou, M. and Oikonomakis, V. and Andreopoulou, O. and Kypreos, K. and Gourzis, P. and Halaris, A. | Excluded | The article was excluded based on title and abstract screening as it did not meet the inclusion and exclusion criteria. |
| Aerobic and resistance exercises affect the BDNF/TrkB signaling pathway, and hippocampal neuron density of high-fat diet-induced obese elderly rats. | 2023 | Maryam, K. and Ali, H. | Excluded | The article was excluded based on title and abstract screening as it did not meet the inclusion and exclusion criteria. |
| Effects of early and late aerobic exercise on right heart failure induced by monocrotaline in rats with pulmonary hypertension | 2024 | Yuan, S. and Qingbo, W. and Yihua, P. and Chunmin, L. and Chuanyi, X. and Yan, Z. | Excluded | The article was excluded based on title and abstract screening as it did not meet the inclusion and exclusion criteria. |
| Pleiotropic and multi-systemic actions of physical exercise on PGC-1α signaling during the aging process | 2023 | Neto, I.V.D.S. and Pinto, A.P. and Muñoz, V.R. and de Cássia Marqueti, R. and Pauli, J.R. and Ropelle, E.R. and Silva, A.S.R.D. | Excluded | The article was excluded based on title and abstract screening as it did not meet the inclusion and exclusion criteria. |
| Cardioprotective and hypotensive mechanistic insights of hydroethanolic extract of Cucumis melo L. kernels in isoprenaline-induced cardiotoxicity based on metabolomics and in silico electrophysiological models | 2023 | Wahid, M. and Saqib, F. and Abbas, G. and Shah, S. and Alshammari, A. and Albekairi, T.H. and Ali, A. and Khurm, M. and Mubarak, M.S. | Excluded | The article was excluded based on title and abstract screening as it did not meet the inclusion and exclusion criteria. |
| Shortened Daily Photoperiod Alleviates Anxiety-like Behaviour by Antioxidant Effect and Changes Serum Fatty Acid Profile in Diabetic Rats | 2023 | Vasović, D.D. and Vesković, M. and Šutulović, N. and Hrnčić, D. and Takić, M. and Jerotić, Đ. and Matić, M. and Stanojlović, O. and Ivković, S. and Jovanović Macura, I. and Mladenović, D. | Excluded | The article was excluded based on title and abstract screening as it did not meet the inclusion and exclusion criteria. |
| Exogenous NADPH exerts a positive inotropic effect and enhances energy metabolism via SIRT3 in pathological cardiac hypertrophy and heart failure | 2023 | Qian, K. and Tang, J. and Ling, Y.-J. and Zhou, M. and Yan, X.-X. and Xie, Y. and Zhu, L.-J. and Nirmala, K. and Sun, K.-Y. and Qin, Z.-H. and Sheng, R. | Excluded | The article was excluded based on title and abstract screening as it did not meet the inclusion and exclusion criteria. |
| Occlusion preconditioned mice are resilient to hypobaric hypoxia-induced myocarditis and arrhythmias due to enhanced immunomodulation, metabolic homeostasis, and antioxidants defense | 2023 | Adzika, G.K. and Mprah, R. and Rizvi, R. and Adekunle, A.O. and Ndzie Noah, M.L. and Wowui, P.I. and Adzraku, S.Y. and Adu-Amankwaah, J. and Wang, F. and Lin, Y. and Fu, L. and Liu, X. and Xiang, J. and Sun, H. | Excluded | The article was excluded based on title and abstract screening as it did not meet the inclusion and exclusion criteria. |
| The efficacy and safety of ginger supplementation in patients with multiple sclerosis: A rationale and study protocol for a double-blind randomized controlled trial | 2023 | Foshati, S. and Poursadeghfard, M. and Heidari, Z. and Amani, R. | Excluded | The article was excluded based on title and abstract screening as it did not meet the inclusion and exclusion criteria. |
| High-intensity interval training improves long-term memory and increases hippocampal antioxidant activity and BDNF levels in ovariectomized Wistar rats | 2023 | Rocha-Gomes, A. and Alvarenga e Castro, T.P. and Almeida, P.R. and Balsamão Paes Leme, P.S. and da Silva, A.A. and Riul, T.R. and Bastos, C.P. and Leite, H.R. | Excluded | The article was excluded based on title and abstract screening as it did not meet the inclusion and exclusion criteria. |
| Unveiling the interplay of AMPK/SIRT1/PGC-1α axis in brain health: Promising targets against aging and NDDs | 2024 | Rakshe, Pratik Shankar and Dutta, Bhaskar Jyoti and Chib, Shivani and Maurya, Niyogita and Singh, Sanjiv | Excluded | The article was excluded based on title and abstract screening as it did not meet the inclusion and exclusion criteria. |
| Beneficial effects of moderate exercise on mice aging: Survival, behavior, oxidative stress, and mitochondrial electron transfer | 2004 | Navarro, A. and Gomez, C. and López-Cepero, J.M. and Boveris, A. | Included |  |
| Pregnancy as a valuable period for preventing hypoxia-ischemia brain damage | 2018 | Netto, C.A. and Sanches, E.F. and Odorcyk, F. and Duran-Carabali, L.E. and Sizonenko, S.V. | Excluded | The article was excluded based on title and abstract screening as it did not meet the inclusion and exclusion criteria. |
| SOD Derived from Bacillus amyloliquefaciens GF423 Has Protection Against Exercise-Induced Oxidative Stress in Healthy Subjects | 2020 | Nam, Yea-eun and Kim, Yunsoo and Lim, Yeni and Kim, Hye Jin and Kwon, Oran | Excluded | The article was excluded based on title and abstract screening as it did not meet the inclusion and exclusion criteria. |
| The novel exercise-induced hormone irisin protects against neuronal injury via activation of the Akt and ERK1/2 signaling pathways and contributes to the neuroprotection of physical exercise in cerebral ischemia | 2017 | Li, Dong-Jie and Li, Yong-Hua and Yuan, Hong-Bin and Qu, Le-Feng and Wang, Pei | Excluded | The article was excluded based on title and abstract screening as it did not meet the inclusion and exclusion criteria. |
| Chronically and acutely exercised rats: Biomarkers of oxidative stress and endogenous antioxidants | 2000 | Liu, J. and Yeo, H.C. and Övervik-Douki, E. and Hagen, T. and Doniger, S.J. and Chu, D.W. and Brooks, G.A. and Ames, B.N. | Included |  |
| The effects of Ellagic acid supplementation on neurotrophic, inflammation, and oxidative stress factors, and indoleamine 2, 3-dioxygenase gene expression in multiple sclerosis patients with mild to moderate depressive symptoms: A randomized, triple-blind, placebo-controlled trial | 2023 | Hajiluian, Ghazaleh and Karegar, Sahar Jafari and Shidfar, Farzad and Aryaeian, Naheed and Salehi, Masoud and Lotfi, Teyebeh and Farhangnia, Pooya and Heshmati, Javad and Delbandi, Ali-Akbar | Excluded | The article was excluded based on title and abstract screening as it did not meet the inclusion and exclusion criteria. |
| HIF1α is necessary for exercise-induced neuroprotection while HIF2α is needed for dopaminergic neuron survival in the substantia nigra pars compacta | 2015 | Smeyne, M. and Sladen, P. and Jiao, Y. and Dragatsis, I. and Smeyne, R.J. | Excluded | The article was excluded based on title and abstract screening as it did not meet the inclusion and exclusion criteria. |
| Inhaled molecular hydrogen attenuates intense acute exercise-induced hippocampal inflammation in sedentary rats | 2020 | Nogueira, Jonatas E. and de Deus, Junia L. and Amorim, Mateus R. and Batalhão, Marcelo E. and Leão, Ricardo M. and Carnio, Evelin C. and Branco, Luiz G.S. | Excluded | The article was excluded based on title and abstract screening as it did not meet the inclusion and exclusion criteria. |
| Effect of maternal exercise on biochemical parameters in rats submitted to neonatal hypoxia-ischemia | 2015 | Marcelino, Thiago Beltram and de Lemos Rodrigues, Patrícia Idalina and Miguel, Patrícia Maidana and Netto, Carlos Alexandre and Pereira Silva, Lenir Orlandi and Matté, Cristiane | Excluded | The article was excluded based on title and abstract screening as it did not meet the inclusion and exclusion criteria. |
| The beneficial effects of nettle supplementation and exercise on brain lesion and memory in rat | 2009 | Toldy, Anna and Atalay, Mustafa and Stadler, Krisztián and Sasvári, Mária and Jakus, Judit and Jung, Kyung J. and Chung, Hae Y. and Nyakas, Csaba and Radák, Zsolt | Excluded | The article was excluded based on title and abstract screening as it did not meet the inclusion and exclusion criteria. |
| Aberrant subcellular neuronal calcium regulation in aging and Alzheimer's disease | 2011 | Camandola, Simonetta and Mattson, Mark P. | Excluded | The article was excluded based on title and abstract screening as it did not meet the inclusion and exclusion criteria. |
| Corticosterone reduces brain mitochondrial function and expression of mitofusin, BDNF in depression-like rodents regardless of exercise preconditioning | 2012 | Liu, Weina and Zhou, Chenglin | Excluded | The article was excluded based on title and abstract screening as it did not meet the inclusion and exclusion criteria. |
| High intensity interval training protects from Post Traumatic Stress Disorder induced cognitive impairment | 2021 | Koyuncuoğlu, Türkan and Sevim, Hacer and Çetrez, Nurşen and Meral, Zeynep and Gönenç, Berfin and Kuntsal Dertsiz, Ekin and Akakın, Dilek and Yüksel, Meral and Kasımay Çakır, Özgür | Excluded | The article was excluded based on title and abstract screening as it did not meet the inclusion and exclusion criteria. |
| Hypoxia compounds exercise-induced free radical formation in humans; partitioning contributions from the cerebral and femoral circulation | 2018 | Bailey, Damian M. and Rasmussen, Peter and Evans, Kevin A. and Bohm, Aske M. and Zaar, Morten and Nielsen, Henning B. and Brassard, Patrice and Nordsborg, Nikolai B. and Homann, Pernille H. and Raven, Peter B. and McEneny, Jane and Young, Ian S. and McCord, Joe M. and Secher, Niels H. | Excluded | The article was excluded based on title and abstract screening as it did not meet the inclusion and exclusion criteria. |
| Chapter 1 - Mitochondrial dysfunction and affective disorders: Focus on diet, exercise, and aging | 2021 | Allen, Josh and Kalynchuk, Lisa E. and Caruncho, Hector J. and de Oliveira, Marcos Roberto | Excluded | The article was excluded based on title and abstract screening as it did not meet the inclusion and exclusion criteria. |
| Exercise-mediated downregulation of MALAT1 expression and implications in primary and secondary cancer prevention | 2020 | Paronetto, Maria Paola and Dimauro, Ivan and Grazioli, Elisa and Palombo, Ramona and Guidotti, Flavia and Fantini, Cristina and Sgrò, Paolo and De Francesco, Dario and Di Luigi, Luigi and Capranica, Laura and Caporossi, Daniela | Excluded | The article was excluded based on title and abstract screening as it did not meet the inclusion and exclusion criteria. |
| Comparing interval and continuous exercise training regimens on neurotrophic factors in rat brain | 2015 | Afzalpour, Mohammad Esmaiel and Chadorneshin, Hossein Taheri and Foadoddini, Mohsen and Eivari, Hossein Abtahi | Included |  |
| Interaction of exercise and ethanol on antioxidant enzymes in brain regions of the rat | 1996 | Somani, S.M. and Husain, K. and Diaz-Phillips, L. and Lanzotti, D.J. and Kareti, K.R. and Trammell, G.L. | Excluded | The article was excluded based on title and abstract screening as it did not meet the inclusion and exclusion criteria. |
| Aerobic interval training improves oxygen uptake efficiency by enhancing cerebral and muscular hemodynamics in patients with heart failure | 2013 | Fu, Tieh-cheng and Wang, Chao-Hung and Lin, Pay-Shin and Hsu, Chih-Chin and Cherng, Wen-Jin and Huang, Shu-Chun and Liu, Min-Hui and Chiang, Cheng-Lin and Wang, Jong-Shyan | Excluded | The article was excluded based on title and abstract screening as it did not meet the inclusion and exclusion criteria. |
| Chapter Four - Manganese Superoxide Dismutase and Oxidative Stress Modulation | 2015 | Bresciani, Guilherme and da Cruz, Ivana Beatrice Mânica and González-Gallego, Javier and Makowski, Gregory S. | Excluded | The article was excluded based on title and abstract screening as it did not meet the inclusion and exclusion criteria. |
| Role of Walnuts in Maintaining Brain Health with Age1, 2, 3 | 2014 | Poulose, Shibu M. and Miller, Marshall G. and Shukitt-Hale, Barbara | Excluded | The article was excluded based on title and abstract screening as it did not meet the inclusion and exclusion criteria. |
| Effects of green tea and physical exercise on memory impairments associated with aging | 2014 | Flôres, Maíra F. and Martins, Alexandre and Schimidt, Helen L. and Santos, Francielli W. and Izquierdo, Iván and Mello-Carpes, Pâmela B. and Carpes, Felipe P. | Excluded | The article was excluded based on title and abstract screening as it did not meet the inclusion and exclusion criteria. |
| Effects of age, dietary, and behavioral enrichment on brain mitochondria in a canine model of human aging | 2009 | Head, E. and Nukala, V.N. and Fenoglio, K.A. and Muggenburg, B.A. and Cotman, C.W. and Sullivan, P.G. | Excluded | The article was excluded based on title and abstract screening as it did not meet the inclusion and exclusion criteria. |
| Chapter Seven - Endocannabinoids and aging—Inflammation, neuroplasticity, mood and pain | 2021 | Park, Yongsoon and Watkins, Bruce A. and Litwack, Gerald | Excluded | The article was excluded based on title and abstract screening as it did not meet the inclusion and exclusion criteria. |
| Diabetes as a risk factor for Alzheimer’s disease in the Middle East and its shared pathological mediators | 2020 | Jayaraj, Richard L. and Azimullah, Sheikh and Beiram, Rami | Excluded | The article was excluded based on title and abstract screening as it did not meet the inclusion and exclusion criteria. |
| Circadian regulation of microglia function: Potential targets for treatment of Parkinson’s Disease | 2024 | Kou, Liang and Chi, Xiaosa and Sun, Yadi and Yin, Sijia and Wu, Jiawei and Zou, Wenkai and Wang, Yiming and Jin, Zongjie and Huang, Jinsha and Xiong, Nian and Xia, Yun and Wang, Tao | Excluded | The article was excluded based on title and abstract screening as it did not meet the inclusion and exclusion criteria. |
| Exercise effects on brain and behavior in healthy mice, Alzheimer’s disease and Parkinson’s disease model—A systematic review and meta-analysis | 2020 | da Costa Daniele, Thiago Medeiros and de Bruin, Pedro Felipe Carvalhedo and de Matos, Robson Salviano and de Bruin, Gabriela Sales and Maia Chaves, Cauby and de Bruin, Veralice Meireles Sales | Excluded | The article was excluded based on title and abstract screening as it did not meet the inclusion and exclusion criteria. |
| COVID-19: A pandemic that threatens physical and mental health by promoting physical inactivity | 2020 | Ali, Amira Mohammed and Kunugi, Hiroshi | Excluded | The article was excluded based on title and abstract screening as it did not meet the inclusion and exclusion criteria. |
| The effect of date seed (Phoenix dactylifera) supplementation on inflammation, oxidative stress biomarkers, and performance in active people: A blinded randomized controlled trial protocol | 2022 | Moslemi, Elham and Dehghan, Parvin and Khani, Mostafa | Excluded | The article was excluded based on title and abstract screening as it did not meet the inclusion and exclusion criteria. |
| Regular exercise improves cognitive function and decreases oxidative damage in rat brain | 1998 | Radak, Zsolt and Nyakas, Csaba and Kaneko, Takao and Goto, Sataro | Excluded | The article was excluded based on title and abstract screening as it did not meet the inclusion and exclusion criteria. |
| Melatonin plus physical exercise are highly neuroprotective in the 3xTg-AD mouse | 2012 | García-Mesa, Yoelvis and Giménez-Llort, Lydia and López, Luis C. and Venegas, Carmen and Cristòfol, Rosa and Escames, Germain and Acuña-Castroviejo, Darío and Sanfeliu, Coral | Excluded | The article was excluded based on title and abstract screening as it did not meet the inclusion and exclusion criteria. |
| The impact of early aerobic exercise on brain microvascular alterations induced by cerebral hypoperfusion | 2017 | Leardini-Tristão, Marina and Borges, Juliana Pereira and Freitas, Felipe and Rangel, Raquel and Daliry, Anissa and Tibiriçá, Eduardo and Estato, Vanessa | Excluded | The article was excluded based on title and abstract screening as it did not meet the inclusion and exclusion criteria. |
| The Institute for Ageing and Health, University of Newcastle, UK | 2002 | Edwardson, J.A. and Kirkwood, T.B.L. | Excluded | The article was excluded based on title and abstract screening as it did not meet the inclusion and exclusion criteria. |
| Chapter Two - Exercise and Parkinson's disease | 2019 | Xu, Xiaojiao and Fu, Zhenfa and Le, Weidong and Yau, Suk-Yu and So, Kwok-Fai | Excluded | The article was excluded based on title and abstract screening as it did not meet the inclusion and exclusion criteria. |
| Chapter Four - Melatonin and healthy aging | 2021 | Cardinali, Daniel P. and Litwack, Gerald | Excluded | The article was excluded based on title and abstract screening as it did not meet the inclusion and exclusion criteria. |
| Glucose-6-phosphate dehydrogenase deficiency and Alzheimer’s disease: Partners in crime? The hypothesis | 2015 | Ulusu, N. Nuray | Excluded | The article was excluded based on title and abstract screening as it did not meet the inclusion and exclusion criteria. |
| Mitochondrial enzyme activities as biochemical markers of aging | 2004 | Navarro, Ana | Excluded | The article was excluded based on title and abstract screening as it did not meet the inclusion and exclusion criteria. |
| Plant-Based Dietary Patterns, Plant Foods, and Age-Related Cognitive Decline | 2019 | Rajaram, Sujatha and Jones, Julie and Lee, Grace J | Excluded | The article was excluded based on title and abstract screening as it did not meet the inclusion and exclusion criteria. |
| Effects of Tai Chi on biomarkers and their implication to neurorehabilitation – a systemic review | 2022 | Liu, Howe and Salem, Yasser and Aggarwal, Sonali | Excluded | The article was excluded based on title and abstract screening as it did not meet the inclusion and exclusion criteria. |
| We need to move more: Neurobiological hypotheses of physical exercise as a treatment for Parkinson’s disease | 2015 | Monteiro-Junior, Renato S. and Cevada, Thais and Oliveira, Bruno R.R. and Lattari, Eduardo and Portugal, Eduardo M.M. and Carvalho, Alessandro and Deslandes, Andrea C. | Excluded | The article was excluded based on title and abstract screening as it did not meet the inclusion and exclusion criteria. |
| Changes in brain glutathione in patients with mild vascular cognitive impairment | 2023 | Chen, Jinghan Jenny and Herrmann, Nathan and Survilla, Kate and Black, Sandra and Ramirez, Joel and Andreazza, Ana and Gallagher, Damien and Graham, Simon and Lanctot, Krista | Excluded | The article was excluded based on title and abstract screening as it did not meet the inclusion and exclusion criteria. |
| Hippocampal UCP2 is essential for cognition and resistance to anxiety but not required for the benefits of exercise | 2014 | Wang, D. and Zhai, X. and Chen, P. and Yang, M. and Zhao, J. and Dong, J. and Liu, H. | Excluded | The article was excluded based on title and abstract screening as it did not meet the inclusion and exclusion criteria. |
| The effects of aerobic exercise training on oxidant–antioxidant balance, neurotrophic factor levels, and blood–brain barrier function in obese and non-obese men | 2017 | Roh, Hee-Tae and So, Wi-Young | Excluded | The article was excluded based on title and abstract screening as it did not meet the inclusion and exclusion criteria. |
| Purinergic signaling influences the neuroinflammatory outcomes of a testosterone-derived synthetic in female rats: Resistance training protective effects on brain health | 2024 | Pereira, A.D.S. and Bottari, N.B. and Nauderer, J.N. and Assmann, C.E. and Copetti, P.M. and Reichert, K.P. and Mostardeiro, V.B. and da Silveira, M.V. and Morsch, V.M.M. and Schetinger, M.R.C. | Excluded | The article was excluded based on title and abstract screening as it did not meet the inclusion and exclusion criteria. |
| Effects of aerobic exercise training in oxidative metabolism and mitochondrial biogenesis markers on prefrontal cortex in obese mice | 2022 | de Sousa Fernandes, M.S. and Aidar, F.J. and da Silva Pedroza, A.A. and de Andrade Silva, S.C. and Santos, G.C.J. and dos Santos Henrique, R. and Clemente, F.M. and Silva, A.F. and de Souza, R.F. and Ferreira, D.J. and Badicu, G. and Lagranha, C. and Nobari, H. | Excluded | The article was excluded based on title and abstract screening as it did not meet the inclusion and exclusion criteria. |
| Malformation of the Cortical Development Associated with Severe Clusters of Epileptic Seizures | 2023 | Cocchetto, A. and Gallucci, A. and Biggio, F. and Cantile, C. | Excluded | The article was excluded based on title and abstract screening as it did not meet the inclusion and exclusion criteria. |
| Anti-aging Effect of Rutin in Caenorhabditis elegans and D-Gal-Induced Aging Mouse Model | 2023 | Xinghua, L. and Yingying, H. and Shuai, W. and Guangping, L. | Excluded | The article was excluded based on title and abstract screening as it did not meet the inclusion and exclusion criteria. |
| Effects of treadmill exercise on metabolism and chronic neuroinflammation in type 1 diabetes mice of different sexes | 2024 | Yanli, X. and Siang, W. and Guodong, Z. | Excluded | The article was excluded based on title and abstract screening as it did not meet the inclusion and exclusion criteria. |
| Weekend warrior exercise model for protection from chronic mild stress-induced depression and ongoing cognitive impairment | 2023 | Öztürk, Ç.Ç. and Ataoğlu, S.N. and Arvas, A. and Tokol, H. and Yaprak, H. and Gürel, S. and Levent, H.N. and Akakın, D. and Şahin, A. and Çakır, B. and Kasımay, Ö. | Excluded | The article was excluded based on title and abstract screening as it did not meet the inclusion and exclusion criteria. |
| Comparison of lifelong and late life exercise on oxidative stress in the cerebellum. | 2009 | Cui L and Hofer T and Rani A and Leeuwenburgh C and Foster TC | Included |  |
| Exercise attenuates neuronal degeneration in Parkinson’s disease rat model by regulating the level of adenosine 2A receptor | 2023 | Li, R. and Jin, S. and He, J. and Peng, Y. and Wei, L. and Gu, L. | Excluded | The article was excluded based on title and abstract screening as it did not meet the inclusion and exclusion criteria. |
| Vitamin D (VD3) Intensifies the Effects of Exercise and Prevents Alterations of Behavior, Brain Oxidative Stress, and Neuroinflammation, in Hemiparkinsonian Rats | 2023 | da Costa, R.O. and Gadelha-Filho, C.V.J. and de Aquino, P.E.A. and Lima, L.A.R. and de Lucena, J.D. and Ribeiro, W.L.C. and Lima, F.A.V. and Neves, K.R.T. and de Barros Viana, G.S. | Excluded | The article was excluded based on title and abstract screening as it did not meet the inclusion and exclusion criteria. |
| Treadmill exercise promotes E3 ubiquitin ligase to remove amyloid β and P-tau and improve cognitive ability in APP/PS1 transgenic mice | 2022 | Xu, L. and Li, M. and Wei, A. and Yang, M. and Li, C. and Liu, R. and Zheng, Y. and Chen, Y. and Wang, Z. and Wang, K. and Wang, T. | Excluded | The article was excluded based on title and abstract screening as it did not meet the inclusion and exclusion criteria. |
| Very early environmental enrichment protects against apoptosis and improves functional recovery from hypoxic–ischemic brain injury | 2023 | Lee, H.Y. and Song, S.-Y. and Hwang, J. and Baek, A. and Baek, D. and Kim, S.H. and Park, J.H. and Choi, S. and Pyo, S. and Cho, S.-R. | Excluded | The article was excluded based on title and abstract screening as it did not meet the inclusion and exclusion criteria. |
| Exercise-Intervened Endothelial Progenitor Cell Exosomes Protect N2a Cells by Improving Mitochondrial Function | 2024 | Chen, S. and Sigdel, S. and Sawant, H. and Bihl, J. and Wang, J. | Excluded | The article was excluded based on title and abstract screening as it did not meet the inclusion and exclusion criteria. |
| Melatonin Alleviates Ovariectomy-Induced Cardiovascular Inflammation in Sedentary or Exercised Rats by Upregulating SIRT1 | 2022 | Arabacı Tamer, S. and Altınoluk, T. and Emran, M. and Korkmaz, S. and Yüksel, R.G. and Baykal, Z. and Dur, Z.S. and Levent, H.N. and Ural, M.A. and Yüksel, M. and Çevik, Ö. and Ercan, F. and Yıldırım, A. and Yeğen, B.Ç. | Excluded | The article was excluded based on title and abstract screening as it did not meet the inclusion and exclusion criteria. |
| Marine Plasmalogens: A Gift from the Sea with Benefits for Age-Associated Diseases | 2023 | Yamashita, S. and Miyazawa, T. and Higuchi, O. and Kinoshita, M. and Miyazawa, T. | Excluded | The article was excluded based on title and abstract screening as it did not meet the inclusion and exclusion criteria. |
| Systematic review and meta-analysis of the effects of exercise on cognitive impairment and neuroprotective mechanisms in diabetes mellitus animal models | 2024 | Liu, Z. and Huang, H. and Zhao, L. | Excluded | The article was excluded based on title and abstract screening as it did not meet the inclusion and exclusion criteria. |
| The deleterious effects of maternal protein deprivation on the brainstem are minimized with moderate physical activity by offspring during early life | 2024 | Bernardo, E.M. and da Silva Pedroza, A.A. and Ferreira, D.J.S. and de Andrade, S.C. and Rozendo, A. and de Sousa Fernandes, M.S. and Silva, T.L. and Fernandes, M.P. and Lagranha, C.J. | Excluded | The article was excluded based on title and abstract screening as it did not meet the inclusion and exclusion criteria. |
| Long-term lifestyle intervention is superior to transient modification for neuroprotection in D-galactose-induced aging rats | 2023 | Pantiya, P. and Thonusin, C. and Chunchai, T. and Pintana, H. and Ongnok, B. and Nawara, W. and Arunsak, B. and Kongkaew, A. and Chattipakorn, N. and Chattipakorn, S.C. | Excluded | The article was excluded based on title and abstract screening as it did not meet the inclusion and exclusion criteria. |
| Implication of Hippocampal Neurogenesis in Autism Spectrum Disorder: Pathogenesis and Therapeutic Implications | 2023 | Liu, C. and Liu, J. and Gong, H. and Liu, T. and Li, X. and Fan, X. | Excluded | The article was excluded based on title and abstract screening as it did not meet the inclusion and exclusion criteria. |
| Taurine deficiency associated with dilated cardiomyopathy and aging | 2024 | Ito, T. and Murakami, S. | Excluded | The article was excluded based on title and abstract screening as it did not meet the inclusion and exclusion criteria. |
| Higher untrained fitness exerts a neuroprotection in Independence to caloric restriction or exercise in high-fat diet-induced obesity | 2023 | Pantiya, P. and Thonusin, C. and Chunchai, T. and Ongnok, B. and Nawara, W. and Arunsak, B. and Chattipakorn, N. and Chattipakorn, S.C. | Excluded | The article was excluded based on title and abstract screening as it did not meet the inclusion and exclusion criteria. |
| Exercise intensity and sex alter neurometabolic, transcriptional, and functional recovery following traumatic brain injury | 2023 | White, B.A. and Ivey, J.T. and Velazquez-Cruz, R. and Oliverio, R. and Whitehead, B. and Pinti, M. and Hollander, J. and Ma, L. and Hu, G. and Weil, Z.M. and Karelina, K. | Excluded | The article was excluded based on title and abstract screening as it did not meet the inclusion and exclusion criteria. |
| Stroke in Parkinson’s disease: a review of epidemiological studies and potential pathophysiological mechanisms | 2023 | Elfil, M. and Bayoumi, A. and Sayed, A. and Aladawi, M. and Aboutaleb, P.E. and Grieb, L. and Tolba, H. and Tinaz, S. | Excluded | The article was excluded based on title and abstract screening as it did not meet the inclusion and exclusion criteria. |
| Starting maternal exercise, unlike reducing the intensity of exercise during pregnancy, prevents memory deficits in female offspring subject to maternal deprivation | 2023 | Carrazoni, G.S. and das Neves, B.-H.S. and dos Santos Soares, M. and Lima, K.R. and Mello-Carpes, P.B. | Excluded | The article was excluded based on title and abstract screening as it did not meet the inclusion and exclusion criteria. |
| Synthesis, characterization, in-silico, and pharmacological evaluation of new 2-amino-6‑trifluoromethoxy benzothiazole derivatives | 2023 | Malik, S. and Miana, G.A. and Ata, A. and Kanwal, M. and Maqsood, S. and Malik, I. and Kazmi, Z. | Excluded | The article was excluded based on title and abstract screening as it did not meet the inclusion and exclusion criteria. |
| Exercise May Increase Oxidative Stress in the Sciatic Nerve in Streptozotocin-Induced Diabetic Rats | 2024 | Nonaka, K. and Akiyama, J. and Une, S. | Excluded | The article was excluded based on title and abstract screening as it did not meet the inclusion and exclusion criteria. |
| The VEGFs/VEGFRs system in Alzheimer's and Parkinson's diseases: Pathophysiological roles and therapeutic implications | 2024 | Ceci, C. and Lacal, P.M. and Barbaccia, M.L. and Mercuri, N.B. and Graziani, G. and Ledonne, A. | Excluded | The article was excluded based on title and abstract screening as it did not meet the inclusion and exclusion criteria. |
| Effects of Exercise Training and L-Arginine Loaded Chitosan Nanoparticles on Hippocampus Histopathology, β-Secretase Enzyme Function, APP, Tau, Iba1and APOE-4 mRNA in Aging Rats | 2024 | Feizolahi, F. and Arabzadeh, E. and Sarshin, A. and Falahi, F. and Dehghannayeri, Z. and Ali Askari, A. and Wong, A. and Aghaei, F. and Zargani, M. | Excluded | The article was excluded based on title and abstract screening as it did not meet the inclusion and exclusion criteria. |
| Exercise Inhibits Doxorubicin-Induced Cardiotoxicity via Regulating B Cells | 2024 | Wang, J. and Liu, S. and Meng, X. and Zhao, X. and Wang, T. and Lei, Z. and Lehmann, H.I. and Li, G. and Alcaide, P. and Bei, Y. and Xiao, J. | Excluded | The article was excluded based on title and abstract screening as it did not meet the inclusion and exclusion criteria. |
| Klebsiella aerogenes ingestion elicits behavioral changes and innate immunity in the host, Caenorhabditis elegans | 2024 | Gowripriya, T. and Yashwanth, R. and James Prabhanand, B. and Suresh, R. and Balamurugan, K. | Excluded | The article was excluded based on title and abstract screening as it did not meet the inclusion and exclusion criteria. |
| Effects of physical exercise associated with a diet enriched with natural antioxidants on cerebral hypoperfusion and reperfusion injury in spontaneously hypertensive rats | 2023 | Lapi, D. and Federighi, G. and Lonardo, M.S. and Chiurazzi, M. and Muscariello, E. and Tenore, G. and Colantuoni, A. and Novellino, E. and Scuri, R. | Excluded | The article was excluded based on title and abstract screening as it did not meet the inclusion and exclusion criteria. |
| Resistance exercise alleviates the prefrontal lobe injury and dysfunction by activating SESN2/AMPK/PGC-1α signaling pathway and inhibiting oxidative stress and inflammation in mice with myocardial infarction | 2023 | Feng, L. and Li, B. and Cai, M. and Zhang, Z. and Zhao, Y. and Yong, S.S. and Tian, Z. | Excluded | The article was excluded based on title and abstract screening as it did not meet the inclusion and exclusion criteria. |
| The persistence of stress-induced physical inactivity in rats: an investigation of central monoamine neurotransmitters and skeletal muscle oxidative stress | 2023 | Buhr, T.J. and Reed, C.H. and Wee, O.M. and Lee, J.H. and Yuan, L.-L. and Fleshner, M. and Valentine, R.J. and Clark, P.J. | Excluded | The article was excluded based on title and abstract screening as it did not meet the inclusion and exclusion criteria. |
| Alleviation of Cognitive and Physical Fatigue with Enzymatic Porcine Placenta Hydrolysate Intake through Reducing Oxidative Stress and Inflammation in Intensely Exercised Rats | 2022 | Kim, M.J. and Zhang, T. and Kim, K.N. and Bae, G.W. and Yoon, S.M. and Yue, Y. and Wu, X. and Park, S. | Excluded | The article was excluded based on title and abstract screening as it did not meet the inclusion and exclusion criteria. |
| Diversity of endurance training effects on antioxidant defenses and oxidative damage in different brain regions of adolescent male rats | 2015 | Chalimoniuk, M. and Jagsz, S. and Sadowska-Krepa, E. and Chrapusta, S.J. and Klapcinska, B. and Langfort, J. | Included |  |
| YK11 induces oxidative stress and mitochondrial dysfunction in hippocampus: The interplay between a selective androgen receptor modulator (SARM) and exercise | 2023 | Dahleh, M.M.M. and Bortolotto, V.C. and Guerra, G.P. and Boeira, S.P. and Prigol, M. | Excluded | The article was excluded based on title and abstract screening as it did not meet the inclusion and exclusion criteria. |
| Comparison of the Effects of Constraint-Induced Movement Therapy and Unconstraint Exercise on Oxidative Stress and Limb Function—A Study on Human Patients and Rats with Cerebral Infarction | 2023 | Wang, D. and Li, L. and Pan, H. and Huang, L. and Sun, X. and He, C. and Wei, Q. | Excluded | The article was excluded based on title and abstract screening as it did not meet the inclusion and exclusion criteria. |
| Antioxidants Prevent the Effects of Physical Exercise on Visual Cortical Plasticity | 2023 | Sansevero, G. and Consorti, A. and Di Marco, I. and Terzibasi Tozzini, E. and Cellerino, A. and Sale, A. | Excluded | The article was excluded based on title and abstract screening as it did not meet the inclusion and exclusion criteria. |
| Suppression of neuroinflammation and α-synuclein oligomerization by rotarod walking exercise in subacute MPTP model of Parkinson's disease | 2023 | Leem, Y.-H. and Park, J.-S. and Park, J.-E. and Kim, D.-Y. and Kim, H.-S. | Excluded | The article was excluded based on title and abstract screening as it did not meet the inclusion and exclusion criteria. |
| The effects of aerobic exercise on neuroimmune responses in animals with traumatic peripheral nerve injury: a systematic review with meta-analyses | 2023 | Sleijser-Koehorst, M.L.S. and Koop, M.A. and Coppieters, M.W. and Lutke Schipholt, I.J. and Radisic, N. and Hooijmans, C.R. and Scholten-Peeters, G.G.M. | Excluded | The article was excluded based on title and abstract screening as it did not meet the inclusion and exclusion criteria. |
| Exploring the neuroprotective role of physical activity in cerebral small vessel disease | 2024 | Anderson, M.E. and Wind, E.J. and Robison, L.S. | Excluded | The article was excluded based on title and abstract screening as it did not meet the inclusion and exclusion criteria. |
| Chronic dietary exposure to arsenic at environmentally relevant concentrations impairs cognitive performance in adult zebrafish (Danio rerio) via oxidative stress and dopaminergic dysfunction | 2023 | Rachamalla, M. and Salahinejad, A. and Khan, M. and Datusalia, A.K. and Niyogi, S. | Excluded | The article was excluded based on title and abstract screening as it did not meet the inclusion and exclusion criteria. |
| Therapeutic advances for treating memory impairments in perinatal brain injuries with implications for cerebral palsy: a systematic review and meta-analysis of preclinical studies | 2023 | Calado, C.M.S.D.S. and Manhães-de-Castro, R. and Pereira, S.D.C. and da Silva Souza, V. and Visco, D.B. and de Silveira, B.S. and de Souza, S.L. and Toscano, A.E. | Excluded | The article was excluded based on title and abstract screening as it did not meet the inclusion and exclusion criteria. |
| Continuous and Intermittent Exercise Upregulates PGC-1α/ Irisin / BDNF Expression to Improve Obesity-induced Cognitive Impairment in Mice | 2023 | Zhang, P.-P. | Excluded | The article was excluded based on title and abstract screening as it did not meet the inclusion and exclusion criteria. |
| The Protective Effects of the Combination of Vitamin E and Swimming Exercise on Memory Impairment Induced by Exposure to Waterpipe Smoke | 2023 | Alzoubi, K.H. and Halboup, A.M. and Khabour, O.F. and Alomari, M.A. | Excluded | The article was excluded based on title and abstract screening as it did not meet the inclusion and exclusion criteria. |
| The protective effect of betelnut polyphenols on exercise-induced fatigue at high altitude and its network pharmacology in rats | 2023 | Sun, Y.-M. and Han, X.-Y. and Ma, J.-H. and Cheng, J.-F. and Wang, R. and Li, W.-B. | Excluded | The article was excluded based on title and abstract screening as it did not meet the inclusion and exclusion criteria. |
| Effects of Regular Exercise and Intermittent Fasting on Neurotransmitters, Inflammation, Oxidative Stress, and Brain-Derived Neurotrophic Factor in Cortex of Ovariectomized Rats | 2023 | Albrahim, T. and Alangry, R. and Alotaibi, R. and Almandil, L. and Alburikan, S. | Excluded | The article was excluded based on title and abstract screening as it did not meet the inclusion and exclusion criteria. |
| Features of a Balanced Healthy Diet with Cardiovascular and Other Benefits | 2023 | Manolis, A.A. and Manolis, T.A. and Melita, H. and Manolis, A.S. | Excluded | The article was excluded based on title and abstract screening as it did not meet the inclusion and exclusion criteria. |
| Effects of aerobic and resistance exercise on antioxidant stress index and brain-derived neurotrophic factor expression in the hippocampus of type 2 diabetic rats | 2023 | Xiaoping, S. and Chunying, L. and Mingjuan, L. and Shunchang, L. and Junzhi, S. and Quansheng, S. | Excluded | The article was excluded based on title and abstract screening as it did not meet the inclusion and exclusion criteria. |
| Neuroinflammation: Molecular Mechanisms and Therapeutic Perspectives | 2022 | Marino, M. and Mele, E. and Pastorino, G.M.G. and Meccariello, R. and Operto, F.F. and Santoro, A. and Viggiano, A. | Excluded | The article was excluded based on title and abstract screening as it did not meet the inclusion and exclusion criteria. |
| TAZ links exercise to mitochondrial biogenesis via mitochondrial transcription factor A | 2022 | Hwang, J.-H. and Kim, K.M. and Oh, H.T. and Yoo, G.D. and Jeong, M.G. and Lee, H. and Park, J. and Jeong, K. and Kim, Y.K. and Ko, Y.-G. and Hwang, E.S. and Hong, J.-H. | Excluded | The article was excluded based on title and abstract screening as it did not meet the inclusion and exclusion criteria. |
| Maternal treadmill exercise ameliorates impairment of neurological outcome, caspase-1 and NLRP3 gene expression alteration in neonatal hypoxia-ischemia rats | 2022 | Gorgij, E. and Fanaei, H. and Yaghmaei, P. and Shahraki, M.R. and Mirahmadi, H. | Excluded | The article was excluded based on title and abstract screening as it did not meet the inclusion and exclusion criteria. |
| Skeletal Muscle Uncoupling Proteins in Mice Models of Obesity | 2022 | Križančić Bombek, L. and Čater, M. | Excluded | The article was excluded based on title and abstract screening as it did not meet the inclusion and exclusion criteria. |
| Nutrition in Brain Aging: Its Relevance to Age-Associated Neurodegeneration | 2022 | Maruyama, W. and Nagai, M.S. and Naoi, M. | Excluded | The article was excluded based on title and abstract screening as it did not meet the inclusion and exclusion criteria. |
| Brain in metabolic syndrome model: The effect of exercises and caloric restriction | 2022 | Alev-Tuzuner, B. and Genc-Kahraman, N. and Ipekci, H. and Ustundag, U.V. and Tunali-Akbay, T. and Emekli-Alturfan, E. and Sener, G. and Yarat, A. | Excluded | The article was excluded based on title and abstract screening as it did not meet the inclusion and exclusion criteria. |
| Healthy dietary intake moderates the effects of age on brain iron concentration and working memory performance | 2021 | Zachariou, V. and Bauer, C.E. and Seago, E.R. and Panayiotou, G. and Hall, E.D. and Butterfield, D.A. and Gold, B.T. | Excluded | The article was excluded based on title and abstract screening as it did not meet the inclusion and exclusion criteria. |
| Protection against glutathione depletion-associated oxidative neuronal death by neurotransmitters norepinephrine and dopamine: Protein disulfide isomerase as a mechanistic target for neuroprotection | 2022 | Choi, H.J. and Chen, T.-X. and Hou, M.-J. and Song, J.H. and Li, P. and Liu, C.-F. and Wang, P. and Zhu, B.T. | Excluded | The article was excluded based on title and abstract screening as it did not meet the inclusion and exclusion criteria. |
| Prenatal stress and KCl-induced depolarization modulate cell death, hypothalamic-pituitary-adrenal axis genes, oxidative and inflammatory response in primary cortical neurons | 2021 | Luft, C. and Haute, G.V. and Wearick-Silva, L.E. and Antunes, K.H. and da Costa, M.S. and de Oliveira, J.R. and Donadio, M.V.F. | Excluded | The article was excluded based on title and abstract screening as it did not meet the inclusion and exclusion criteria. |
| Loss of Mitochondrial Ca2+Uniporter Limits Inotropic Reserve and Provides Trigger and Substrate for Arrhythmias in Barth Syndrome Cardiomyopathy | 2021 | Bertero, E. and Nickel, A. and Kohlhaas, M. and Hohl, M. and Sequeira, V. and Brune, C. and Schwemmlein, J. and Abeßer, M. and Schuh, K. and Kutschka, I. and Carlein, C. and Münker, K. and Atighetchi, S. and Müller, A. and Kazakov, A. and Kappl, R. and Von Der Malsburg, K. and Van Der Laan, M. and Schiuma, A.-F. and Böhm, M. and Laufs, U. and Hoth, M. and Rehling, P. and Kuhn, M. and Dudek, J. and Von Der Malsburg, A. and Prates Roma, L. and Maack, C. | Excluded | The article was excluded based on title and abstract screening as it did not meet the inclusion and exclusion criteria. |
| Pyrroloquinoline-quinone is more than an antioxidant: A vitamin-like accessory factor important in health and disease prevention | 2021 | Jonscher, K.R. and Chowanadisai, W. and Rucker, R.B. | Excluded | The article was excluded based on title and abstract screening as it did not meet the inclusion and exclusion criteria. |
| Cognitive–exercise dual-task intervention ameliorates cognitive decline in natural aging rats through reducing oxidative stress and enhancing synaptic plasticity | 2022 | Li, X.-L. and Tao, X. and Li, T.-C. and Zhu, Z.-M. and Huang, P.-L. and Gong, W.-J. | Excluded | The article was excluded based on title and abstract screening as it did not meet the inclusion and exclusion criteria. |
| Effects of 7-Week Aerobic Training and Piperine Supplementation on Oxidative Stress Markers of Brain Tissue in Male Wistar Rats Exposed to Paraquat | 2022 | Asadi Ferizi, S.M. and Hejazi, K. and Askari, V.R. and Haghighi, A.H. | Excluded | The article was excluded based on title and abstract screening as it did not meet the inclusion and exclusion criteria. |
| Glycolytic preconditioning in astrocytes mitigates trauma-induced neurodegeneration | 2021 | Fonseca, R.S. and Metang, P. and Egge, N. and Liu, Y. and Zuurbier, K.R. and Sivaprakasam, K. and Shirazi, S. and Chuah, A. and Arneaud, S.L.B. and Konopka, G. and Qian, D. and Douglas, P.M. | Excluded | The article was excluded based on title and abstract screening as it did not meet the inclusion and exclusion criteria. |
| Bioinformatics Analysis Identifies Potential Ferroptosis Key Genes in the Pathogenesis of Intracerebral Hemorrhage | 2021 | Liu, T. and Li, X. and Cui, Y. and Meng, P. and Zeng, G. and Wang, Y. and Wang, Q. | Excluded | The article was excluded based on title and abstract screening as it did not meet the inclusion and exclusion criteria. |
| Barren environment damages cognitive abilities in fish: Behavioral and transcriptome mechanisms | 2021 | Zhang, Z. and Fu, Y. and Shen, F. and Zhang, Z. and Guo, H. and Zhang, X. | Excluded | The article was excluded based on title and abstract screening as it did not meet the inclusion and exclusion criteria. |
| Exercise intensities modulate cognitive function in spontaneously hypertensive rats through oxidative mediated synaptic plasticity in hippocampus | 2021 | Lee, C.-C. and Wu, D.-Y. and Chen, S.-Y. and Lin, Y.-P. and Lee, T.-M. | Excluded | The article was excluded based on title and abstract screening as it did not meet the inclusion and exclusion criteria. |
| Irisin stimulates protective signaling pathways in rat hippocampal neurons | 2022 | Lourenco, M.V. and de Freitas, G.B. and Raony, I. and Ferreira, S.T. and De Felice, F.G. | Excluded | The article was excluded based on title and abstract screening as it did not meet the inclusion and exclusion criteria. |
| The Obese Brain: Mechanisms of Systemic and Local Inflammation, and Interventions to Reverse the Cognitive Deficit | 2022 | Salas-Venegas, V. and Flores-Torres, R.P. and Rodríguez-Cortés, Y.M. and Rodríguez-Retana, D. and Ramírez-Carreto, R.J. and Concepción-Carrillo, L.E. and Pérez-Flores, L.J. and Alarcón-Aguilar, A. and López-Díazguerrero, N.E. and Gómez-González, B. and Chavarría, A. and Konigsberg, M. | Excluded | The article was excluded based on title and abstract screening as it did not meet the inclusion and exclusion criteria. |
| Timely N-Acetyl-Cysteine and Environmental Enrichment Rescue Oxidative Stress-Induced Parvalbumin Interneuron Impairments via MMP9/RAGE Pathway: A Translational Approach for Early Intervention in Psychosis | 2021 | Dwir, D. and Cabungcal, J.-H. and Xin, L. and Giangreco, B. and Parietti, E. and Cleusix, M. and Jenni, R. and Klauser, P. and Conus, P. and Cuénod, M. and Steullet, P. and Do, K.Q. | Excluded | The article was excluded based on title and abstract screening as it did not meet the inclusion and exclusion criteria. |
| p27, The Cell Cycle and Alzheimer´s Disease | 2022 | García-Osta, A. and Dong, J. and Moreno-Aliaga, M.J. and Ramirez, M.J. | Excluded | The article was excluded based on title and abstract screening as it did not meet the inclusion and exclusion criteria. |
| Preconditioning exercise reduces hippocampal neuronal damage via increasing Klotho expression in ischemic rats | 2022 | Karizmeh, M.S. and Shabani, M. and Shabani, M. and Sardari, M. and Babaei, J.F. and Nabavizadeh, F. and Sadr, S.S. and Adeli, S. | Excluded | The article was excluded based on title and abstract screening as it did not meet the inclusion and exclusion criteria. |
| Adult Neurogenesis under Control of the Circadian System | 2022 | Ali, A.A.H. and von Gall, C. | Excluded | The article was excluded based on title and abstract screening as it did not meet the inclusion and exclusion criteria. |
| Dipeptide IF and Exercise Training Attenuate Hypertension in SHR Rats by Inhibiting Fibrosis and Hypertrophy and Activating AMPKα1, SIRT1, and PGC1α | 2022 | Ho, J.-H. and Baskaran, R. and Wang, M.-F. and Mohammedsaleh, Z.M. and Yang, H.-S. and Balasubramanian, B. and Lin, W.-T. | Excluded | The article was excluded based on title and abstract screening as it did not meet the inclusion and exclusion criteria. |
| Remnant cholesterol and atherosclerotic cardiovascular disease: Metabolism, mechanism, evidence, and treatment | 2022 | Wang, K. and Wang, R. and Yang, J. and Liu, X. and Shen, H. and Sun, Y. and Zhou, Y. and Fang, Z. and Ge, H. | Excluded | The article was excluded based on title and abstract screening as it did not meet the inclusion and exclusion criteria. |
| Voluntary Exercise Prevents Hypertensive Response Sensitization Induced by Angiotensin II | 2022 | Xue, B. and Cui, J.-L. and Guo, F. and Beltz, T.G. and Zhao, Z.-G. and Zhang, G.-S. and Johnson, A.K. | Excluded | The article was excluded based on title and abstract screening as it did not meet the inclusion and exclusion criteria. |
| Impact of the main cardiovascular risk factors on plasma extracellular vesicles and their influence on the heart’s vulnerability to ischemia-reperfusion injury | 2021 | Majka, M. and Kleibert, M. and Wojciechowska, M. | Excluded | The article was excluded based on title and abstract screening as it did not meet the inclusion and exclusion criteria. |
| Preconditioning Exercise in Rats Attenuates Early Brain Injury Resulting from Subarachnoid Hemorrhage by Reducing Oxidative Stress, Inflammation, and Neuronal Apoptosis | 2021 | Otsuka, S. and Setoyama, K. and Takada, S. and Nakanishi, K. and Terashi, T. and Norimatsu, K. and Tani, A. and Sakakima, H. and Maruyama, I. and Tancharoen, S. and Tanaka, E. and Kikuchi, K. | Excluded | The article was excluded based on title and abstract screening as it did not meet the inclusion and exclusion criteria. |
| 6-Hydroxydopamine-Induced Neurotoxicity in Rat Model of Parkinson’s Disease: Is Reversed via Anti-Oxidative Activities of Curcumin and Aerobic Exercise Therapy | 2022 | Sokouti, H. and Mohajeri, D. and Nourazar, M.A. | Excluded | The article was excluded based on title and abstract screening as it did not meet the inclusion and exclusion criteria. |
| Skeletal muscle atrophy: From mechanisms to treatments | 2021 | Yin, L. and Li, N. and Jia, W. and Wang, N. and Liang, M. and Yang, X. and Du, G. | Excluded | The article was excluded based on title and abstract screening as it did not meet the inclusion and exclusion criteria. |
| Downregulation of TREM2/NF-кB signaling may damage the blood-brain barrier and aggravate neuronal apoptosis in experimental rats with surgically injured brain | 2022 | Shi, M. and Gong, Y. and Wu, M. and Gu, H. and Yu, J. and Gao, F. and Ren, Z. and Qian, M. and Dang, B. and Chen, G. | Excluded | The article was excluded based on title and abstract screening as it did not meet the inclusion and exclusion criteria. |
| Glymphatic imaging and modulation of the optic nerve | 2022 | Kasi, A. and Liu, C. and Faiq, M. and Chan, K. | Excluded | The article was excluded based on title and abstract screening as it did not meet the inclusion and exclusion criteria. |
| The mitochondrial antioxidant sirtuin3 cooperates with lipid metabolism to safeguard neurogenesis in aging and depression | 2022 | Santos, S.S. and Moreira, J.B. and Costa, M. and Rodrigues, R.S. and Sebastião, A.M. and Xapelli, S. and Solá, S. | Excluded | The article was excluded based on title and abstract screening as it did not meet the inclusion and exclusion criteria. |
| A 10-day mild treadmill exercise performed before an epileptic seizure alleviates oxidative injury in the skeletal muscle and brain tissues of the rats | 2022 | Arabaci-Tamer, S. and Cilingir-Kaya, O.T. and Yuksel, M. and Yildirim, A. and Yegen, B.C. | Excluded | The article was excluded based on title and abstract screening as it did not meet the inclusion and exclusion criteria. |
| Natural antioxidants enhance the power of physical and mental activities versus risk factors inducing progression of Alzheimer's disease in rats | 2021 | Ali, A.A. and Abd El-Fattah, A.I. and Abu-Elfotuh, K. and Elariny, H.A. | Excluded | The article was excluded based on title and abstract screening as it did not meet the inclusion and exclusion criteria. |
| Magnesium Potentiates the Vortioxetine’s Effects on Physical Performances and Biological Changes in Exercise-Induced Stress in Rats | 2022 | Fotache, P.A. and Mititelu-Tartau, L. and Bogdan, M. and Buca, B.R. and Pavel, L.L. and Pelin, A.-M. and Meca, A.-D. and Tartau, C.-G. and Popa, G.E. | Excluded | The article was excluded based on title and abstract screening as it did not meet the inclusion and exclusion criteria. |
| Therapeutic Effects of High-Intensity Interval Training Exercise Alone and Its Combination with Ecdysterone Against Amyloid Beta-Induced Rat Model of Alzheimer’s Disease: A Behavioral, Biochemical, and Histological Study | 2022 | Gholipour, P. and Komaki, A. and Parsa, H. and Ramezani, M. | Excluded | The article was excluded based on title and abstract screening as it did not meet the inclusion and exclusion criteria. |
| The Endogenous Metabolite Glycerophosphocholine Promotes Longevity and Fitness in Caenorhabditis elegans | 2022 | Liu, J.-Y. and Zheng, R.-Q. and Wang, Y. and Liu, Y.-H. and Jiang, S. and Wang, X.-Z. and He, K. and Pan, X. and Zhou, T. and Li, T. and Xia, Q. and Zhang, W.-N. | Excluded | The article was excluded based on title and abstract screening as it did not meet the inclusion and exclusion criteria. |
| Xylooligosaccharides and aerobic training regulate metabolism and behavior in rats with streptozotocin-induced type 1 diabetes | 2022 | Choneva, M. and Shishmanova-Doseva, M. and Dimov, I. and Boyanov, K. and Dimitrov, I. and Vlaykova, T. and Georgieva, K. and Hrischev, P. and Bivolarska, A. | Excluded | The article was excluded based on title and abstract screening as it did not meet the inclusion and exclusion criteria. |
| Adropin increases with swimming exercise and exerts a protective effect on the brain of aged rats | 2022 | Parlak, H. and Ozkan, A. and Sinen, O. and Bulbul, M. and Aslan, M.A. and Agar, A. | Excluded | The article was excluded based on title and abstract screening as it did not meet the inclusion and exclusion criteria. |
| Dynamic Tests in Pituitary Endocrinology: Pitfalls in Interpretation during Aging | 2022 | Caputo, M. and Mele, C. and Ferrero, A. and Leone, I. and Daffara, T. and Marzullo, P. and Prodam, F. and Aimaretti, G. | Excluded | The article was excluded based on title and abstract screening as it did not meet the inclusion and exclusion criteria. |
| Differential effects of voluntary and forced exercise trainings on spatial learning ability and hippocampal biomarkers in aged female rats | 2022 | Belviranlı, M. and Okudan, N. | Excluded | The article was excluded based on title and abstract screening as it did not meet the inclusion and exclusion criteria. |
| Therapeutic Approach to Alzheimer’s Disease: Current Treatments and New Perspectives | 2022 | Pardo-Moreno, T. and González-Acedo, A. and Rivas-Domínguez, A. and García-Morales, V. and García-Cozar, F.J. and Ramos-Rodríguez, J.J. and Melguizo-Rodríguez, L. | Excluded | The article was excluded based on title and abstract screening as it did not meet the inclusion and exclusion criteria. |
| Hippocampal Oxidative Stress Induced by Radiofrequency Electromagnetic Radiation and the Neuroprotective Effects of Aerobic Exercise in Rats: A Randomized Control Trial | 2021 | Mojez, M.R. and Gaeini, A.A. and Choobineh, S. and Sheykhlouvand, M. | Excluded | The article was excluded based on title and abstract screening as it did not meet the inclusion and exclusion criteria. |
| Role of vitamins in the metabolic syndrome and cardiovascular disease | 2022 | Aguilera-Méndez, A. and Boone-Villa, D. and Nieto-Aguilar, R. and Villafaña-Rauda, S. and Molina, A.S. and Sobrevilla, J.V. | Excluded | The article was excluded based on title and abstract screening as it did not meet the inclusion and exclusion criteria. |
| Physical Training Protects Against Brain Toxicity in Mice Exposed to an Experimental Model of Glioblastoma | 2022 | Costa, A.K. and Marqueze, L.F.B. and Gattiboni, B.B. and Pedroso, G.S. and Vasconcellos, F.F. and Cunha, E.B.B. and Justa, H.C. and Baldissera, A.B. and Nagashima, S. and de Noronha, L. and Radak, Z. and Fernandes, L.C. and Pinho, R.A. | Excluded | The article was excluded based on title and abstract screening as it did not meet the inclusion and exclusion criteria. |
| A review of the preclinical and clinical studies on the role of the gut microbiome in aging and neurodegenerative diseases and its modulation | 2022 | Hashim, H.M. and Makpol, S. | Excluded | The article was excluded based on title and abstract screening as it did not meet the inclusion and exclusion criteria. |
| The Nuanced Metabolic Functions of Endogenous FGF21 Depend on the Nature of the Stimulus, Tissue Source, and Experimental Model | 2022 | Spann, R.A. and Morrison, C.D. and den Hartigh, L.J. | Excluded | The article was excluded based on title and abstract screening as it did not meet the inclusion and exclusion criteria. |
| Mechanisms of memory impairment in animal models of nontraumatic intracranial hemorrhage: A systematic review of the literature | 2022 | Peterson, C. and Umoye, A.O. and Puglisi, C.H. and Waldau, B. | Excluded | The article was excluded based on title and abstract screening as it did not meet the inclusion and exclusion criteria. |
| Memorable Food: Fighting Age-Related Neurodegeneration by Precision Nutrition | 2021 | Milošević, M. and Arsić, A. and Cvetković, Z. and Vučić, V. | Excluded | The article was excluded based on title and abstract screening as it did not meet the inclusion and exclusion criteria. |
| Exercise Training and Verbena officinalis L. Affect Pre-Clinical and Histological Parameters | 2022 | Rodrigues Oliveira, S.M. and Dias, E. and Girol, A.P. and Silva, H. and Pereira, M.D.L. | Excluded | The article was excluded based on title and abstract screening as it did not meet the inclusion and exclusion criteria. |
| Preclinical and clinical evidence of IGF-1 as a prognostic marker and acute intervention with ischemic stroke | 2021 | Hayes, C.A. and Valcarcel-Ares, M.N. and Ashpole, N.M. | Excluded | The article was excluded based on title and abstract screening as it did not meet the inclusion and exclusion criteria. |
| The beneficial effect of exercise against Alzheimer's disease may result from improved brain glucose metabolism | 2021 | Zhao, N. and Xu, B. | Excluded | The article was excluded based on title and abstract screening as it did not meet the inclusion and exclusion criteria. |
| Glucose 6-P Dehydrogenase—An Antioxidant Enzyme with Regulatory Functions in Skeletal Muscle during Exercise | 2022 | García-Domínguez, E. and Carretero, A. and Viña-Almunia, A. and Domenech-Fernandez, J. and Olaso-Gonzalez, G. and Viña, J. and Gomez-Cabrera, M.C. | Excluded | The article was excluded based on title and abstract screening as it did not meet the inclusion and exclusion criteria. |
| The oxidation-resistant CaMKII-MM281/282VV mutation does not prevent arrhythmias in CPVT1 | 2021 | Sadredini, M. and Manotheepan, R. and Lehnart, S.E. and Anderson, M.E. and Sjaastad, I. and Stokke, M.K. | Excluded | The article was excluded based on title and abstract screening as it did not meet the inclusion and exclusion criteria. |
| Exercise renovates H2S and Nrf2-related antioxidant pathways to suppress apoptosis in the natural ageing process of male rat cortex | 2021 | Lin, J.-Y. and Ho, T.-J. and Tsai, B.C.-K. and Chiang, C.-Y. and Kao, H.-C. and Kuo, W.-W. and Chen, R.-J. and Viswanadha, V.P. and Huang, C.-W. and Huang, C.-Y. | Excluded | The article was excluded based on title and abstract screening as it did not meet the inclusion and exclusion criteria. |
| DL-3-n-butylphthalide improved physical and learning and memory performance of rodents exposed to acute and chronic hypobaric hypoxia | 2021 | Xu, G. and Shi, Y.-K. and Sun, B.-D. and Liu, L. and Guo-Ji, E. and He, S. and Zhang, J.-Y. and Liu, B. and Hu, Q. and Chen, J. and Gao, Y.-Q. and Zhang, E.-L. | Excluded | The article was excluded based on title and abstract screening as it did not meet the inclusion and exclusion criteria. |
| Arctic Stress: Mechanisms and Experimental Models | 2022 | Gmoshinski, I.V. and Nikityuk, D.B. | Excluded | The article was excluded based on title and abstract screening as it did not meet the inclusion and exclusion criteria. |
| Hydrogen sulfide enhances adult neurogenesis in a mouse model of Parkinson's disease | 2021 | Wang, M. and Tang, J.-J. and Wang, L.-X. and Yu, J. and Zhang, L. and Qiao, C. | Excluded | The article was excluded based on title and abstract screening as it did not meet the inclusion and exclusion criteria. |
| Melatonin and zinc supplements with physical and mental activities subside neurodegeneration and hepatorenal injury induced by aluminum chloride in rats: Inclusion of GSK-3β-Wnt/β-catenin signaling pathway | 2022 | Abu-Elfotuh, K. and Hussein, F.H. and Abbas, A.N. and Al-Rekabi, M.D. and Barghash, S.S. and Zaghlool, S.S. and El-Emam, S.Z. | Excluded | The article was excluded based on title and abstract screening as it did not meet the inclusion and exclusion criteria. |
| Interactive Effects of Swimming High-Intensity Interval Training and Resveratrol Supplementation Improve Mitochondrial Protein Levels in the Hippocampus of Aged Rats | 2022 | Amirazodi, M. and Daryanoosh, F. and Mehrabi, A. and Gaeini, A. and Koushkie Jahromi, M. and Salesi, M. and Zarifkar, A.H. | Excluded | The article was excluded based on title and abstract screening as it did not meet the inclusion and exclusion criteria. |
| Exercise training reduces brainstem oxidative stress and restores normal breathing function in heart failure | 2021 | Díaz-Jara, E. and Díaz, H.S. and Rios-Gallardo, A. and Ortolani, D. and Andrade, D.C. and Toledo, C. and Pereyra, K.V. and Schwarz, K. and Ramirez, G. and Ortiz, F.C. and Andía, M.E. and Del Rio, R. | Excluded | The article was excluded based on title and abstract screening as it did not meet the inclusion and exclusion criteria. |
| Exercise and the brain in multiple sclerosis | 2022 | Lozinski, B.M. and Yong, V.W. | Excluded | The article was excluded based on title and abstract screening as it did not meet the inclusion and exclusion criteria. |
| Secondary hypoxic ischemia alters neurobehavioral outcomes, neuroinflammation, and oxidative stress in mice exposed to controlled cortical impact | 2021 | Oh, S.-K. and Park, H.-J. and Yu, G.-G. and Jeong, S.-H. and Lee, S.-W. and Kim, H. | Excluded | The article was excluded based on title and abstract screening as it did not meet the inclusion and exclusion criteria. |
| Treadmill Exercise Alleviates Brain Iron Dyshomeostasis Accelerating Neuronal Amyloid-β Production, Neuronal Cell Death, and Cognitive Impairment in Transgenic Mice Model of Alzheimer’s Disease | 2021 | Choi, D.-H. and Kwon, K.-C. and Hwang, D.-J. and Koo, J.-H. and Um, H.-S. and Song, H.-S. and Kim, J.-S. and Jang, Y. and Cho, J.-Y. | Excluded | The article was excluded based on title and abstract screening as it did not meet the inclusion and exclusion criteria. |
| Rat models of vocal deficits in parkinson’s disease | 2021 | Krasko, M.N. and Hoffmeister, J.D. and Schaen-Heacock, N.E. and Welsch, J.M. and Kelm-Nelson, C.A. and Ciucci, M.R. | Excluded | The article was excluded based on title and abstract screening as it did not meet the inclusion and exclusion criteria. |
| Swimming Improves Memory and Antioxidant Defense in an Animal Model of Duchenne Muscular Dystrophy | 2021 | Nocetti, P.M. and Alberti, A. and Freiberger, V. and Ventura, L. and Grigollo, L.R. and Andreau, C.S. and Júnior, R.J.N. and Martins, D.F. and Comim, C.M. | Excluded | The article was excluded based on title and abstract screening as it did not meet the inclusion and exclusion criteria. |
| Exercise-Induced Adipose Tissue Thermogenesis and Browning: How to Explain the Conflicting Findings? | 2022 | Zhu, Y. and Qi, Z. and Ding, S. | Excluded | The article was excluded based on title and abstract screening as it did not meet the inclusion and exclusion criteria. |
| The muscle-brain axis and neurodegenerative diseases: The key role of mitochondria in exercise-induced neuroprotection | 2021 | Burtscher, J. and Millet, G.P. and Place, N. and Kayser, B. and Zanou, N. | Excluded | The article was excluded based on title and abstract screening as it did not meet the inclusion and exclusion criteria. |
| Melatonin as an Antioxidant Agent in Stroke: An Updated Review | 2022 | Wang, J. and Gao, S. and Lenahan, C. and Gu, Y. and Wang, X. and Fang, Y. and Xu, W. and Wu, H. and Pan, Y. and Shao, A. and Zhang, J. | Excluded | The article was excluded based on title and abstract screening as it did not meet the inclusion and exclusion criteria. |
| Autophagy guided interventions to modify the cardiac phenotype of Danon disease | 2022 | Yadin, D. and Petrover, Z. and Shainberg, A. and Alcalai, R. and Waldman, M. and Seidman, J. and Seidman, C.E. and Abraham, N.G. and Hochhauser, E. and Arad, M. | Excluded | The article was excluded based on title and abstract screening as it did not meet the inclusion and exclusion criteria. |
| Lung-Brain Crosstalk in Sepsis: Protective Effect of Prophylactic Physical Exercise Against Inflammation and Oxidative Stress in Rats | 2022 | Giustina, A.D. and Rodrigues, J.F. and Bagio, E. and Bonfante, S. and Joaquim, L. and Zarbato, G. and Stork, S. and Machado, R.S. and de Souza Goldim, M.P. and Danielski, L.G. and Mathias, K. and Dacoregio, C. and Cardoso, T. and Predroso, G.S. and Venturini, L.M. and Zaccaron, R.P. and Silveira, P.C.L. and Pinho, R.A. and Petronilho, F. | Excluded | The article was excluded based on title and abstract screening as it did not meet the inclusion and exclusion criteria. |
| Storage Duration Affects the Quantification of Oxidative Stress Markers in the Gastrocnemius, Heart, and Brain of Mice Submitted to a Maximum Exercise | 2022 | Costa, K.B. and Leite, H.R. and Garcia, B.C.C. and Ottone, V.O. and Mendoncą, G.D.R. and Cordeiro, P.J. and Chaves, P.R. and Deus, F.A. and Tossige-Gomes, R. and Coimbra, C.C. and Rocha-Vieira, E. | Excluded | The article was excluded based on title and abstract screening as it did not meet the inclusion and exclusion criteria. |
| Neuroinflammation: The Role of Anthocyanins as Neuroprotectants | 2022 | Banji, O.J.F. and Banji, D. and Makeen, H.A. and Alqahtani, S.S. and Alshahrani, S. | Excluded | The article was excluded based on title and abstract screening as it did not meet the inclusion and exclusion criteria. |
| Long-term exercise pre-training attenuates Alzheimer’s disease–related pathology in a transgenic rat model of Alzheimer’s disease | 2022 | Yang, L. and Wu, C. and Li, Y. and Dong, Y. and Wu, C.Y.-C. and Lee, R.H.-C. and Brann, D.W. and Lin, H.W. and Zhang, Q. | Excluded | The article was excluded based on title and abstract screening as it did not meet the inclusion and exclusion criteria. |
| Curcumin Alleviates Restraint Stress-Induced Learning and Memory Deficit and Activity via Modulation of Biochemical, Morphology Changes, and Apoptosis in the Prefrontal Cortex and Hippocampus | 2022 | Pourheydar, B. and Abar, M. and Farjah, G. and Pourheydar, M. and Derafshpour, L. | Excluded | The article was excluded based on title and abstract screening as it did not meet the inclusion and exclusion criteria. |
| Vitamin D attenuated 6-OHDA-induced behavioural deficits, dopamine dysmetabolism, oxidative stress, and neuro-inflammation in mice | 2022 | Bayo-Olugbami, A. and Nafiu, A.B. and Amin, A. and Ogundele, O.M. and Lee, C.C. and Owoyele, B.V. | Excluded | The article was excluded based on title and abstract screening as it did not meet the inclusion and exclusion criteria. |
| Bee pollen increases hippocampal brain-derived neurotrophic factor and suppresses neuroinflammation in adult rats with chronic immobilization stress | 2022 | Saral, Ö. and Şahin, H. and Saral, S. and Alkanat, M. and Akyıldız, K. and Topçu, A. and Yılmaz, A. | Excluded | The article was excluded based on title and abstract screening as it did not meet the inclusion and exclusion criteria. |
| Effects of low-intensity training on the brain and muscle in the congenital muscular dystrophy 1D model | 2022 | Comim, C.M. and Soares, J.A. and Alberti, A. and Freiberger, V. and Ventura, L. and Dias, P. and Schactae, A.L. and Grigollo, L.R. and Steckert, A.V. and Martins, D.F. and Junior, R.J.N. and Vainzof, M. and Quevedo, J. | Excluded | The article was excluded based on title and abstract screening as it did not meet the inclusion and exclusion criteria. |
| Protective effects of forced exercise against topiramate-induced cognition impairment and enhancement of its antiepileptic activity: molecular and behavioral evidences | 2022 | Soleimani Meigoni, Z. and Jabari, F. and Motaghinejad, M. and Motevalian, M. | Excluded | The article was excluded based on title and abstract screening as it did not meet the inclusion and exclusion criteria. |
| Running from Stress: Neurobiological Mechanisms of Exercise-Induced Stress Resilience | 2022 | Nowacka-Chmielewska, M. and Grabowska, K. and Grabowski, M. and Meybohm, P. and Burek, M. and Małecki, A. | Excluded | The article was excluded based on title and abstract screening as it did not meet the inclusion and exclusion criteria. |
| Pre- and Post-Endurance Training Mitigates the Rat Pilocarpine-Induced Status Epilepticus and Epileptogenesis-Associated Deleterious Consequences | 2022 | Shishmanova-Doseva, M. and Georgieva, K. and Uzunova, Y. and Ioanidu, L. and Atanasova, M. and Nenchovska, Z. and Tchekalarova, J. | Excluded | The article was excluded based on title and abstract screening as it did not meet the inclusion and exclusion criteria. |
| Memantine modulates oxidative stress in the rat brain following experimental autoimmune encephalomyelitis | 2021 | Dąbrowska‐bouta, B. and Strużyńska, L. and Sidoryk‐węgrzynowicz, M. and Sulkowski, G. | Excluded | The article was excluded based on title and abstract screening as it did not meet the inclusion and exclusion criteria. |
| Neuroprotective Effects of Phytochemicals against Aluminum Chloride-Induced Alzheimer’s Disease through ApoE4/LRP1, Wnt3/β-Catenin/GSK3β, and TLR4/NLRP3 Pathways with Physical and Mental Activities in a Rat Model | 2022 | Hamdan, A.M.E. and Alharthi, F.H.J. and Alanazi, A.H. and El-Emam, S.Z. and Zaghlool, S.S. and Metwally, K. and Albalawi, S.A. and Abdu, Y.S. and Mansour, R.E.-S. and Salem, H.A. and Abd Elmageed, Z.Y. and Abu-Elfotuh, K. | Excluded | The article was excluded based on title and abstract screening as it did not meet the inclusion and exclusion criteria. |
| Achieving brain clearance and preventing neurodegenerative diseases—A glymphatic perspective | 2021 | Kylkilahti, T.M. and Berends, E. and Ramos, M. and Shanbhag, N.C. and Töger, J. and Markenroth Bloch, K. and Lundgaard, I. | Excluded | The article was excluded based on title and abstract screening as it did not meet the inclusion and exclusion criteria. |
| The impact of moderate-intensity swimming exercise on learning and memory in aged rats: The role of Sirtuin-1 | 2021 | Tunca, U. and Saygin, M. and Ozmen, O. and Aslankoc, R. and Yalcin, A. | Excluded | The article was excluded based on title and abstract screening as it did not meet the inclusion and exclusion criteria. |
| Effects of the combination of high-intensity interval training and Ecdysterone on learning and memory abilities, antioxidant enzyme activities, and neuronal population in an Amyloid-beta-induced rat model of Alzheimer's disease | 2022 | Gholipour, P. and Komaki, A. and Ramezani, M. and Parsa, H. | Excluded | The article was excluded based on title and abstract screening as it did not meet the inclusion and exclusion criteria. |
| Bioactive Peptides: Synthesis, Sources, Applications, and Proposed Mechanisms of Action | 2022 | Akbarian, M. and Khani, A. and Eghbalpour, S. and Uversky, V.N. | Excluded | The article was excluded based on title and abstract screening as it did not meet the inclusion and exclusion criteria. |
| Pharmacological effects of Eleutherococcus senticosus on the neurological disorders | 2022 | Li, X.-T. and Zhou, J.-C. and Zhou, Y. and Ren, Y.-S. and Huang, Y.-H. and Wang, S.-M. and Tan, L. and Yang, Z.-Y. and Ge, Y.-W. | Excluded | The article was excluded based on title and abstract screening as it did not meet the inclusion and exclusion criteria. |
| Exercise rapidly alters proteomes in mice following spinal cord demyelination | 2021 | Lozinski, B.M. and de Almeida, L.G.N. and Silva, C. and Dong, Y. and Brown, D. and Chopra, S. and Yong, V.W. and Dufour, A. | Excluded | The article was excluded based on title and abstract screening as it did not meet the inclusion and exclusion criteria. |
| Swimming exercise attenuates anxiety-like behavior by reducing brain oxidative stress in type 2 diabetic mice | 2021 | Matinfar, P. and Peeri, M. and Azarbayjani, M.A. | Excluded | The article was excluded based on title and abstract screening as it did not meet the inclusion and exclusion criteria. |
| Combination of stem cells and rehabilitation therapies for ischemic stroke | 2021 | Berlet, R. and Anthony, S. and Brooks, B. and Wang, Z.-J. and Sadanandan, N. and Shear, A. and Cozene, B. and Gonzales-Portillo, B. and Parsons, B. and Salazar, F.E. and Lezama Toledo, A.R. and Monroy, G.R. and Gonzales-Portillo, J.V. and Borlongan, C.V. | Excluded | The article was excluded based on title and abstract screening as it did not meet the inclusion and exclusion criteria. |
| Effects of Endurance Training on the Coenzyme Q Redox State in Rat Heart, Liver, and Brain at the Tissue and Mitochondrial Levels: Implications for Reactive Oxygen Species Formation and Respiratory Chain Remodeling | 2022 | Dominiak, K. and Galganski, L. and Budzinska, A. and Woyda-Ploszczyca, A. and Zoladz, J.A. and Jarmuszkiewicz, W. | Excluded | The article was excluded based on title and abstract screening as it did not meet the inclusion and exclusion criteria. |
| The glycolytic process in endothelial cells and its implications | 2022 | Leung, S.W.S. and Shi, Y. | Excluded | The article was excluded based on title and abstract screening as it did not meet the inclusion and exclusion criteria. |
| Effects of Polyphenols in Aging and Neurodegeneration Associated with Oxidative Stress | 2022 | Rivas, F. and Poblete-Aro, C. and Pando, M.E. and Allel, M.J. and Fernandez, V. and Soto, A. and Nova, P. and Garcia-Diaz, D. | Excluded | The article was excluded based on title and abstract screening as it did not meet the inclusion and exclusion criteria. |
| The Influence of Gut Microbiota on Neurogenesis: Evidence and Hopes | 2022 | Sarubbo, F. and Cavallucci, V. and Pani, G. | Excluded | The article was excluded based on title and abstract screening as it did not meet the inclusion and exclusion criteria. |
| Protective Effect of Ginsenosides from Stems and Leaves of Panax ginseng against Scopolamine-Induced Memory Damage via Multiple Molecular Mechanisms | 2022 | Wang, Y. and Zhang, J.-J. and Hou, J.-G. and Li, X. and Liu, W. and Zhang, J.-T. and Zheng, S.-W. and Su, F.-Y. and Li, W. | Excluded | The article was excluded based on title and abstract screening as it did not meet the inclusion and exclusion criteria. |
| The Impact of Physical Exercise on the Hippocampus in Physiological Condition and Ageing-Related Decline: Current Evidence from Animal and Human Studies | 2022 | Lauretta, G. and Ravalli, S. and Maugeri, G. and D’agata, V. and Di Rosa, M. and Musumeci, G. | Excluded | The article was excluded based on title and abstract screening as it did not meet the inclusion and exclusion criteria. |
| The Evolution of Ketosis: Potential Impact on Clinical Conditions | 2022 | Dilliraj, L.N. and Schiuma, G. and Lara, D. and Strazzabosco, G. and Clement, J. and Giovannini, P. and Trapella, C. and Narducci, M. and Rizzo, R. | Excluded | The article was excluded based on title and abstract screening as it did not meet the inclusion and exclusion criteria. |
| Comparative veterinary geroscience: mechanism of molecular, cellular, and tissue aging in humans, laboratory animal models, and companion dogs and cats | 2022 | McKenzie, B.A. | Excluded | The article was excluded based on title and abstract screening as it did not meet the inclusion and exclusion criteria. |
| Stimulants cocktail: Methylphenidate plus caffeine impairs memory and cognition and alters mitochondrial and oxidative status | 2021 | Freddo, N. and Soares, S.M. and Fortuna, M. and Pompermaier, A. and Varela, A.C.C. and Maffi, V.C. and Mozzato, M.T. and de Alcantara Barcellos, H.H. and Koakoski, G. and Barcellos, L.J.G. and Rossato-Grando, L.G. | Excluded | The article was excluded based on title and abstract screening as it did not meet the inclusion and exclusion criteria. |
| Modulatory effect of myokines on reactive oxygen species in ischemia/reperfusion | 2020 | Szabó, M.R. and Pipicz, M. and Csont, T. and Csonka, C. | Excluded | The article was excluded based on title and abstract screening as it did not meet the inclusion and exclusion criteria. |
| Does a Session of Endurance Training with Three Different Intensities Affect the Cerebral Dopamine Neurotrophic Factor, superoxide dismutase, and malondialdehyde Levels of Cerebral Cortex in Male Rats? | 2018 | Shirvani, H. and Aslani, J. and Mohammadi, Z.F. | Included |  |
| Hydrogen sulfide alleviates anxiety, motor, and cognitive dysfunctions in rats with maternal hyperhomocysteinemia via mitigation of oxidative stress | 2020 | Yakovleva, O. and Bogatova, K. and Mukhtarova, R. and Yakovlev, A. and Shakhmatova, V. and Gerasimova, E. and Ziyatdinova, G. and Hermann, A. and Sitdikova, G. | Excluded | The article was excluded based on title and abstract screening as it did not meet the inclusion and exclusion criteria. |
| Sex differences in long-term effects of exertional heat stroke on myocardial metabolism | 2020 | Bugyei-Twum, A. | Excluded | The article was excluded based on title and abstract screening as it did not meet the inclusion and exclusion criteria. |
| The Effect of Short-Term Physical Activity on the Oxidative Stress in Rats with Different Stress Resistance Profiles in Cerebral Hypoperfusion | 2020 | Chrishtop, V.V. and Tomilova, I.K. and Rumyantseva, T.A. and Mikhaylenko, E.V. and Avila-Rodriguez, M.F. and Mikhaleva, L.M. and Nikolenko, V.N. and Somasundaram, S.G. and Kirkland, C.E. and Bachurin, S.O. and Aliev, G. | Excluded | The article was excluded based on title and abstract screening as it did not meet the inclusion and exclusion criteria. |
| Exercise-Linked Irisin Prevents Mortality and Enhances Cognition in a Mice Model of Cerebral Ischemia by Regulating Klotho Expression | 2021 | Jin, Z. and Zhang, Z. and Ke, J. and Wang, Y. and Wu, H. | Excluded | The article was excluded based on title and abstract screening as it did not meet the inclusion and exclusion criteria. |
| Lepidium meyenii supplemented diet modulates neurobehavioral and biochemical parameters in mice fed high-fat high-sugar diet | 2021 | Olofinnade, A.T. and Alawode, A. and Onaolapo, A.Y. and Onaolapo, O.J. | Excluded | The article was excluded based on title and abstract screening as it did not meet the inclusion and exclusion criteria. |
| Preclinical techniques to investigate exercise training in vascular pathophysiology | 2021 | Sangha, G.S. and Goergen, C.J. and Prior, S.J. and Ranadive, S.M. and Clyne, A.M. | Excluded | The article was excluded based on title and abstract screening as it did not meet the inclusion and exclusion criteria. |
| Role of regular physical activity in neuroprotection against acute ischemia | 2020 | Raimondo, D.D. and Rizzo, G. and Musiari, G. and Tuttolomondo, A. and Pinto, A. | Excluded | The article was excluded based on title and abstract screening as it did not meet the inclusion and exclusion criteria. |
| Melatonin and healthy aging | 2021 | Cardinali, D.P. and Litwack G. and Toluca Lake, North Hollywood, CA | Excluded | The article was excluded based on title and abstract screening as it did not meet the inclusion and exclusion criteria. |
| X-Irradiation at 0.5 Gy after the forced swim test reduces forced swimming-induced immobility in mice | 2020 | Kataoka, T. and Shuto, H. and Yano, J. and Naoe, S. and Ishida, T. and Nakada, T. and Yamato, K. and Hanamoto, K. and Nomura, T. and Yamaoka, K. | Excluded | The article was excluded based on title and abstract screening as it did not meet the inclusion and exclusion criteria. |
| Protective effect of natrium diethyldithiocarbamate trihydrate (Nddct) on lead induced neurodegeneration in rats | 2021 | Kumar, S. and Maurya, H. and Sen, S. and Chakraborty, R. | Excluded | The article was excluded based on title and abstract screening as it did not meet the inclusion and exclusion criteria. |
| Preventive treatments to slow substantia nigra damage and Parkinson's disease progression: A critical perspective review | 2020 | Bjørklund, G. and Dadar, M. and Anderson, G. and Chirumbolo, S. and Maes, M. | Excluded | The article was excluded based on title and abstract screening as it did not meet the inclusion and exclusion criteria. |
| The cerebrovascular and neurological impact of chronic smoking on post-traumatic brain injury outcome and recovery: An in vivo study | 2020 | Sivandzade, F. and Alqahtani, F. and Sifat, A. and Cucullo, L. | Excluded | The article was excluded based on title and abstract screening as it did not meet the inclusion and exclusion criteria. |
| Possible neuroprotective mechanisms of physical exercise in neurodegeneration | 2020 | Mahalakshmi, B. and Maurya, N. and Lee, S.-D. and Kumar, V.B. | Excluded | The article was excluded based on title and abstract screening as it did not meet the inclusion and exclusion criteria. |
| Modulation of microRNAs as a potential molecular mechanism involved in the beneficial actions of physical exercise in Alzheimer disease | 2020 | Improta-Caria, A.C. and Nonaka, C.K.V. and Cavalcante, B.R.R. and De Sousa, R.A.L. and Júnior, R.A. and Souza, B.S.F. | Excluded | The article was excluded based on title and abstract screening as it did not meet the inclusion and exclusion criteria. |
| Skeletal muscle health and cognitive function: A narrative review | 2021 | Sui, S.X. and Williams, L.J. and Holloway-Kew, K.L. and Hyde, N.K. and Pasco, J.A. | Excluded | The article was excluded based on title and abstract screening as it did not meet the inclusion and exclusion criteria. |
| The Contribution of Plasma and Brain Vitamin C on Age and Gender-Related Cognitive Differences: A Mini-Review of the Literature | 2020 | Travica, N. and Ried, K. and Hudson, I. and Sali, A. and Scholey, A. and Pipingas, A. | Excluded | The article was excluded based on title and abstract screening as it did not meet the inclusion and exclusion criteria. |
| Pharmacological Strategies to Improve Dendritic Spines in Alzheimer's Disease | 2021 | Ettcheto, M. and Busquets, O. and Cano, A. and Sánchez-Lopez, E. and Manzine, P.R. and Espinosa-Jimenez, T. and Verdaguer, E. and Sureda, F.X. and Olloquequi, J. and Castro-Torres, R.D. and Auladell, C. and Folch, J. and Casadesús, G. and Camins, A. | Excluded | The article was excluded based on title and abstract screening as it did not meet the inclusion and exclusion criteria. |
| Exercise training combined with bifidobacterium longum OLP-01 supplementation improves exercise physiological adaption and performance | 2020 | Huang, W.-C. and Hsu, Y.-J. and Huang, C.-C. and Liu, H.-C. and Lee, M.-C. | Excluded | The article was excluded based on title and abstract screening as it did not meet the inclusion and exclusion criteria. |
| Regular aerobic exercise increased vegf levels in both soleus and gastrocnemius muscles correlated with hippocampal learning and vegf levels | 2021 | Karakilic, A. and Yuksel, O. and Kizildag, S. and Hosgorler, F. and Topcugil, B. and Ilgin, R. and Gumus, H. and Guvendi, G. and Koc, B. and Kandis, S. and Ates, M. and Uysal, N. | Excluded | The article was excluded based on title and abstract screening as it did not meet the inclusion and exclusion criteria. |
| The Changes of Brain Edema and Neurological Outcome, and the Probable Mechanisms in Diffuse Traumatic Brain Injury Induced in Rats with the History of Exercise | 2020 | Soltani, N. and Soltani, Z. and Khaksari, M. and Ebrahimi, G. and Hajmohammmadi, M. and Iranpour, M. | Excluded | The article was excluded based on title and abstract screening as it did not meet the inclusion and exclusion criteria. |
| Physical exercise-induced myokines in neurodegenerative diseases | 2021 | Lee, B. and Shin, M. and Park, Y. and Won, S.-Y. and Cho, K.S. | Excluded | The article was excluded based on title and abstract screening as it did not meet the inclusion and exclusion criteria. |
| Role of muscle-specific histone methyltransferase (Smyd1) in exercise-induced cardioprotection against pathological remodeling after myocardial infarction | 2020 | Liang, Q. and Cai, M. and Zhang, J. and Song, W. and Zhu, W. and Xi, L. and Tian, Z. | Excluded | The article was excluded based on title and abstract screening as it did not meet the inclusion and exclusion criteria. |
| Regular moderate exercise alleviates gastric oxidative damage in rats via the contribution of oxytocin receptors | 2020 | Arabacı Tamer, S. and Üçem, S. and Büke, B. and Güner, M. and Karaküçük, A.G. and Yiğit, N. and Şirvancı, S. and Çevik, Ö. and Ercan, F. and Yeğen, B.Ç. | Excluded | The article was excluded based on title and abstract screening as it did not meet the inclusion and exclusion criteria. |
| Ginsenoside Rd reverses cognitive deficits by modulating BDNF-dependent CREB pathway in chronic restraint stress mice | 2020 | Wang, H. and Jiang, N. and Lv, J. and Huang, H. and Liu, X. | Excluded | The article was excluded based on title and abstract screening as it did not meet the inclusion and exclusion criteria. |
| Protective effect of ginsenoside Rb1 against chronic restraint stress (CRS)-induced memory impairments in rats | 2021 | Jiang, N. and Wang, K. and Zhang, Y. and Huang, H. and Lv, J.-W. and Wang, Q. and Wang, H.-X. and Xia, T.-J. and Liu, X.-M. | Excluded | The article was excluded based on title and abstract screening as it did not meet the inclusion and exclusion criteria. |
| Towards a comprehensive etiopathogenetic and pathophysiological theory of multiple sclerosis | 2020 | Tobore, T.O. | Excluded | The article was excluded based on title and abstract screening as it did not meet the inclusion and exclusion criteria. |
| The role of natural antioxidants in the prevention of dementia—where do we stand and future perspectives | 2021 | Jurcau, A. | Excluded | The article was excluded based on title and abstract screening as it did not meet the inclusion and exclusion criteria. |
| Effects of caloric restriction diet on arterial hypertension and endothelial dysfunction | 2021 | Di Daniele, N. and Marrone, G. and Di Lauro, M. and Di Daniele, F. and Palazzetti, D. and Guerriero, C. and Noce, A. | Excluded | The article was excluded based on title and abstract screening as it did not meet the inclusion and exclusion criteria. |
| Exercise Training and Neurodegeneration in Mitochondrial Disorders: Insights From the Harlequin Mouse | 2020 | Fernández-de la Torre, M. and Fiuza-Luces, C. and Valenzuela, P.L. and Laine-Menéndez, S. and Arenas, J. and Martín, M.A. and Turnbull, D.M. and Lucia, A. and Morán, M. | Excluded | The article was excluded based on title and abstract screening as it did not meet the inclusion and exclusion criteria. |
| Ginkgobilobaextractprotectsearlybraininjuryaftersubarachnoid hemorrhage via inhibiting thioredoxin interacting protein/ NLRP3 signaling pathway | 2020 | Du, C. and Xi, C. and Wu, C. and Sha, J. and Zhang, J. and Li, C. | Excluded | The article was excluded based on title and abstract screening as it did not meet the inclusion and exclusion criteria. |
| Central and peripheral metabolic defects contribute to the pathogenesis of Alzheimer's disease: Targeting mitochondria for diagnosis and prevention | 2020 | Peng, Y. and Gao, P. and Shi, L. and Chen, L. and Liu, J. and Long, J. | Excluded | The article was excluded based on title and abstract screening as it did not meet the inclusion and exclusion criteria. |
| Ketone Body, 3-Hydroxybutyrate: Minor Metabolite - Major Medical Manifestations | 2020 | Møller, N. | Excluded | The article was excluded based on title and abstract screening as it did not meet the inclusion and exclusion criteria. |
| Lutein/zeaxanthin isomers regulate neurotrophic factors and synaptic plasticity in trained rats | 2021 | Orhan, C. and Erten, F. and Er, B. and Tuzcu, M. and Şahin, N. and Durmaz Kurşun, Ö.E. and Juturu, V. and Şahin, K. | Excluded | The article was excluded based on title and abstract screening as it did not meet the inclusion and exclusion criteria. |
| Oxidative stress in phenylketonuria—evidence from human studies and animal models, and possible implications for redox signaling | 2021 | Bortoluzzi, V.T. and Dutra Filho, C.S. and Wannmacher, C.M.D. | Excluded | The article was excluded based on title and abstract screening as it did not meet the inclusion and exclusion criteria. |
| The effects of voluntary complex and regular wheel running exercises on the levels of 8-oxoguanine DNA glycosylase, semaphorin 3B, H2O2, and apoptosis in the hippocampus of diabetic rats | 2021 | Fazelzadeh, M. and Afzalpour, M.E. and Fallah Mohammadi, Z. and Falah Mohammadi, H. | Excluded | The article was excluded based on title and abstract screening as it did not meet the inclusion and exclusion criteria. |
| A Walnut Diet in Combination with Enriched Environment Improves Cognitive Function and Affects Lipid Metabolites in Brain and Liver of Aged NMRI Mice | 2021 | Esselun, C. and Dilberger, B. and Silaidos, C.V. and Koch, E. and Schebb, N.H. and Eckert, G.P. | Excluded | The article was excluded based on title and abstract screening as it did not meet the inclusion and exclusion criteria. |
| Understanding normal brain aging | 2021 | Garaschuk, O. | Excluded | The article was excluded based on title and abstract screening as it did not meet the inclusion and exclusion criteria. |
| Ameliorative effects of endurance training and Matricaria chamomilla flowers hydroethanolic extract on cognitive deficit in type 2 diabetes rats | 2021 | Heidarianpour, A. and Mohammadi, F. and Keshvari, M. and Mirazi, N. | Excluded | The article was excluded based on title and abstract screening as it did not meet the inclusion and exclusion criteria. |
| Diabetes-Alzheimer's Disease Link: Targeting Mitochondrial Dysfunction and Redox Imbalance | 2021 | Carvalho, C. and Cardoso, S. | Excluded | The article was excluded based on title and abstract screening as it did not meet the inclusion and exclusion criteria. |
| Resveratrol improves heart function by moderating inflammatory processes in patients with systolic heart failure | 2020 | Gal, R. and Deres, L. and Horvath, O. and Eros, K. and Sandor, B. and Urban, P. and Soos, S. and Marton, Z. and Sumegi, B. and Toth, K. and Habon, T. and Halmosi, R. | Excluded | The article was excluded based on title and abstract screening as it did not meet the inclusion and exclusion criteria. |
| Effects of Exercise Training on Anxious-Depressive-like Behavior in Alzheimer Rat | 2020 | Wu, C. and Yang, L. and Li, Y. and Dong, Y.A.N. and Yang, B. and Tucker, L.D. and Zong, X. and Zhang, Q. | Excluded | The article was excluded based on title and abstract screening as it did not meet the inclusion and exclusion criteria. |
| Implications of Altered Ketone Metabolism and Therapeutic Ketosis in Heart Failure | 2020 | Selvaraj, S. and Kelly, D.P. and Margulies, K.B. | Excluded | The article was excluded based on title and abstract screening as it did not meet the inclusion and exclusion criteria. |
| Effect of a neuroprotective exercise protocol on oxidative state and BDNF levels in the rat hippocampus | 2008 | Cechetti, F. and Fochesatto, C. and Scopel, D. and Nardin, P. and Gonçalves, C.A. and Netto, C.A. and Siqueira, I.R. | Included |  |
| Effects of lifelong exercise on age-related body composition, oxidative stress, inflammatory cytokines, and skeletal muscle proteome in rats | 2020 | Gao, H.-E. and Wu, D.-S. and Sun, L. and Yang, L.-D. and Qiao, Y.-B. and Ma, S. and Wu, Z.-J. and Ruan, L. and Li, F.-H. | Excluded | The article was excluded based on title and abstract screening as it did not meet the inclusion and exclusion criteria. |
| A cross-sectional study of functional and metabolic changes during aging through the lifespan in male mice | 2021 | Petr, M.A. and Alfaras, I. and Krawcyzk, M. and Bair, W.-N. and Mitchell, S.J. and Morrell, C.H. and Studenski, S.A. and Price, N.L. and Fishbein, K.W. and Spencer, R.G. and Scheibye-Knudsen, M. and Lakatta, E.G. and Ferrucci, L. and Aon, M.A. and Bernier, M. and de Cabo, R. | Excluded | The article was excluded based on title and abstract screening as it did not meet the inclusion and exclusion criteria. |
| Age-related alterations at neuromuscular junction: Role of oxidative stress and epigenetic modifications | 2021 | Dobrowolny, G. and Barbiera, A. and Sica, G. and Scicchitano, B.M. | Excluded | The article was excluded based on title and abstract screening as it did not meet the inclusion and exclusion criteria. |
| Multicomponent Training Prevents Memory Deficit Related to Amyloid-β Protein-Induced Neurotoxicity | 2021 | Soares, C.B. and Daré, L.R. and Lima, K.R. and Lopes, L.F. and Santos, A.G.D. and Schimidt, H.L. and Carpes, F.P. and Lloret, A. and Viña, J. and Mello-Carpes, P.B. | Excluded | The article was excluded based on title and abstract screening as it did not meet the inclusion and exclusion criteria. |
| Physiological effects of deoxynivalenol from naturally contaminated corn on cerebral tryptophan metabolism, behavioral response, gastrointestinal immune status and health in pigs following a pair-feeding model | 2021 | Shen, Y.-B. and Weaver, A.C. and Kim, S.W. | Excluded | The article was excluded based on title and abstract screening as it did not meet the inclusion and exclusion criteria. |
| Treadmill Exercise during Pregnancy Decreased Vulnerability to Neonatal Hypoxia-Ischemia through Reducing Inflammation and Increasing Antiapoptotic Gene Expressions and Antioxidant Capacity in Rats | 2021 | Gorgij, E. and Fanaei, H. and Yaghmaei, P. and Shahraki, M.R. and Mirahmadi, H. | Excluded | The article was excluded based on title and abstract screening as it did not meet the inclusion and exclusion criteria. |
| Lactate and BDNF: Key mediators of exercise induced neuroplasticity? | 2020 | Müller, P. and Duderstadt, Y. and Lessmann, V. and Müller, N.G. | Excluded | The article was excluded based on title and abstract screening as it did not meet the inclusion and exclusion criteria. |
| A fundamental role for oxidants and intracellular calcium signals in Alzheimer’s pathogenesis—and how a comprehensive antioxidant strategy may aid prevention of this disorder | 2021 | McCarty, M.F. and Dinicolantonio, J.J. and Lerner, A. | Excluded | The article was excluded based on title and abstract screening as it did not meet the inclusion and exclusion criteria. |
| Effects of exercise-induced beta-hydroxybutyrate on muscle function and cognitive function | 2021 | Kwak, S.E. and Bae, J.H. and Lee, J.H. and Shin, H.E. and Zhang, D. and Cho, S.C. and Song, W. | Excluded | The article was excluded based on title and abstract screening as it did not meet the inclusion and exclusion criteria. |
| A Long-Term Enriched Environment Ameliorates the Accelerated Age-Related Memory Impairment Induced by Gestational Administration of Lipopolysaccharide: Role of Plastic Mitochondrial Quality Control | 2021 | Zhuang, Z.-Q. and Zhang, Z.-Z. and Zhang, Y.-M. and Ge, H.-H. and Sun, S.-Y. and Zhang, P. and Chen, G.-H. | Excluded | The article was excluded based on title and abstract screening as it did not meet the inclusion and exclusion criteria. |
| Protective effects of Colomast®, a new formulation of adelmidrol and sodium hyaluronate, in a mouse model of acute restraint stress | 2020 | D’amico, R. and Siracusa, R. and Fusco, R. and Cordaro, M. and Genovese, T. and Peritore, A.F. and Gugliandolo, E. and Crupi, R. and Impellizzeri, D. and Cuzzocrea, S. and Di Paola, R. | Excluded | The article was excluded based on title and abstract screening as it did not meet the inclusion and exclusion criteria. |
| Effect of exercise training on antioxidant system in brain regions of rat | 1995 | Rybak, L.P. and Somani, S.M. and Ravi, R. | Included |  |
| Oxidative/Nitroxidative Stress and Multiple Sclerosis | 2021 | Tobore, T.O. | Excluded | The article was excluded based on title and abstract screening as it did not meet the inclusion and exclusion criteria. |
| Effect of Aerobic Physical Exercise in an Animal Model of Duchenne Muscular Dystrophy | 2020 | Hoepers, A. and Alberti, A. and Freiberger, V. and Ventura, L. and Grigollo, L.R. and Andreu, C.S. and da Silva, B.B. and Martins, D.F. and Junior, R.J.N. and Streck, E.L. and Comim, C.M. | Excluded | The article was excluded based on title and abstract screening as it did not meet the inclusion and exclusion criteria. |
| Sex differences impact the pancreatic response to chronic immobilization stress in rats | 2021 | Abdel Hafez, S.M.N. and Allam, F.A.F.A. and Elbassuoni, E. | Excluded | The article was excluded based on title and abstract screening as it did not meet the inclusion and exclusion criteria. |
| Adiponectin role in neurodegenerative diseases: Focus on nutrition review | 2020 | Polito, R. and Di Meo, I. and Barbieri, M. and Daniele, A. and Paolisso, G. and Rizzo, M.R. | Excluded | The article was excluded based on title and abstract screening as it did not meet the inclusion and exclusion criteria. |
| Depletion of brain perivascular macrophages regulates acute restraint stress-induced neuroinflammation and oxidative/nitrosative stress in rat frontal cortex | 2020 | Sayd, A. and Vargas-Caraveo, A. and Perea-Romero, I. and Robledo-Montaña, J. and Caso, J.R. and Madrigal, J.L.M. and Leza, J.C. and Orio, L. and Garcia-Bueno, B. | Excluded | The article was excluded based on title and abstract screening as it did not meet the inclusion and exclusion criteria. |
| Concentration-dependent effects of dietary L-ascorbic acid fortification in the brains of healthy mice | 2021 | Olofinnade, A.T. and Onaolapo, A.Y. and Onaolapo, O.J. | Excluded | The article was excluded based on title and abstract screening as it did not meet the inclusion and exclusion criteria. |
| Exercise-induced peptide EIP-22 protect myocardial from ischaemia/reperfusion injury via activating JAK2/STAT3 signalling pathway | 2021 | Zhang, L. and Wang, X. and Zhang, H. and Feng, M. and Ding, J. and Zhang, B. and Cheng, Z. and Qian, L. | Excluded | The article was excluded based on title and abstract screening as it did not meet the inclusion and exclusion criteria. |
| Restraint-induced behavioral deficits are attenuated or impaired by pre- or post-injection of apomorphine: A context-based study | 2020 | Ikram, H. and Ahmed, S. and Haleem, D.J. | Excluded | The article was excluded based on title and abstract screening as it did not meet the inclusion and exclusion criteria. |
| Key signaling pathways in aging and potential interventions for healthy aging | 2021 | Yu, M. and Zhang, H. and Wang, B. and Zhang, Y. and Zheng, X. and Shao, B. and Zhuge, Q. and Jin, K. | Excluded | The article was excluded based on title and abstract screening as it did not meet the inclusion and exclusion criteria. |
| Endurance Exercise-Induced Autophagy/Mitophagy Coincides with a Reinforced Anabolic State and Increased Mitochondrial Turnover in the Cortex of Young Male Mouse Brain | 2021 | Kwon, I. and Jang, Y. and Lee, Y. | Excluded | The article was excluded based on title and abstract screening as it did not meet the inclusion and exclusion criteria. |
| Traditional Korean herbal formulae, Yuk-Mi-Ji-Hwang-Tang, ameliorates impairment of hippocampal memory ability by chronic restraint stress of mouse model | 2020 | Eom, T.-M. and Kwon, H.-H. and Shin, N. and Kim, D.-W. and Fang, Z. and Seol, I.-C. and Kim, Y.-S. and Kim, H.-G. and Yoo, H.-R. | Excluded | The article was excluded based on title and abstract screening as it did not meet the inclusion and exclusion criteria. |
| Beneficial Effects of Exercise on Depression and Anxiety During the Covid-19 Pandemic: A Narrative Review | 2020 | Hu, S. and Tucker, L. and Wu, C. and Yang, L. | Excluded | The article was excluded based on title and abstract screening as it did not meet the inclusion and exclusion criteria. |
| New approach to peripheral nerve injury: nutritional therapy | 2020 | Yildiran, H. and Macit, M.S. and Özata Uyar, G. | Excluded | The article was excluded based on title and abstract screening as it did not meet the inclusion and exclusion criteria. |
| Effect of Maternal Exercise on Diet-induced Redox Imbalance in Hippocampus of Adult Offspring | 2020 | August, P.M. and Hözer, R. and Rodrigues, K.S. and dos Santos, B.G. and Maurmann, R.M. and Scortegagna, M.C. and Matté, C. | Excluded | The article was excluded based on title and abstract screening as it did not meet the inclusion and exclusion criteria. |
| Role of alcohol drinking in Alzheimer’s disease, Parkinson’s disease, and amyotrophic lateral sclerosis | 2020 | Peng, B. and Yang, Q. and Joshi, R.B. and Liu, Y. and Akbar, M. and Song, B.-J. and Zhou, S. and Wang, X. | Excluded | The article was excluded based on title and abstract screening as it did not meet the inclusion and exclusion criteria. |
| Effects of maternal chewing on prenatal stress-induced cognitive impairments in the offspring via multiple molecular pathways | 2020 | Zhou, Q. and Suzuki, A. and Iinuma, M. and Wang, K.-Y. and Kubo, K.-Y. and Azuma, K. | Excluded | The article was excluded based on title and abstract screening as it did not meet the inclusion and exclusion criteria. |
| Preventive and Therapeutic Potential of Physical Exercise in Neurodegenerative Diseases | 2021 | Marques-Aleixo, I. and Beleza, J. and Sampaio, A. and Stevanović, J. and Coxito, P. and Gonçalves, I. and Ascensão, A. and Magalhães, J. | Excluded | The article was excluded based on title and abstract screening as it did not meet the inclusion and exclusion criteria. |
| Aerobic exercise attenuates neurodegeneration and promotes functional recovery – Why it matters for neurorehabilitation & neural repair | 2020 | Zhang, D. and Lu, Y. and Zhao, X. and Zhang, Q. and Li, L. | Excluded | The article was excluded based on title and abstract screening as it did not meet the inclusion and exclusion criteria. |
| Hyperbaric Oxygen Therapy and Vascular Complications in Diabetes Mellitus | 2020 | Resanović, I. and Zarić, B. and Radovanović, J. and Sudar-Milovanović, E. and Gluvić, Z. and Jevremović, D. and Isenović, E.R. | Excluded | The article was excluded based on title and abstract screening as it did not meet the inclusion and exclusion criteria. |
| Nuclear Factor [Erythroid-derived 2]-like 2 and Mitochondrial Transcription Factor A Contribute to Moderate-intensity Swimming Effectiveness against Memory Impairment in Young Mice Induced by Concomitant Exposure to a High-calorie Diet during the Early Life Period | 2021 | Jardim, N.S. and Müller, S.G. and Pase, F.M. and Nogueira, C.W. | Excluded | The article was excluded based on title and abstract screening as it did not meet the inclusion and exclusion criteria. |
| Valeriana fauriei Exerts Antidepressant-Like Effects through Anti-inflammatory and Antioxidant Activities by Inhibiting Brain-Derived Neurotrophic Factor Associated with Chronic Restraint Stress | 2020 | Choi, J.H. and Choi, J.H. and Lee, M.J. and Chang, Y. and Chang, Y. and Lee, S. and Kim, H.-J. and Lee, S.W. and Kim, Y.O. and Cho, I.-H. | Excluded | The article was excluded based on title and abstract screening as it did not meet the inclusion and exclusion criteria. |
| Physical exercise protects against mitochondria alterations in the 6-hidroxydopamine rat model of Parkinson's disease | 2020 | Ferreira, A.F.F. and Binda, K.H. and Singulani, M.P. and Pereira, C.P.M. and Ferrari, G.D. and Alberici, L.C. and Real, C.C. and Britto, L.R. | Excluded | The article was excluded based on title and abstract screening as it did not meet the inclusion and exclusion criteria. |
| The dichotomic role of macrophage migration inhibitory factor in neurodegeneration | 2020 | Basile, M.S. and Battaglia, G. and Bruno, V. and Mangano, K. and Fagone, P. and Petralia, M.C. and Nicoletti, F. and Cavalli, E. | Excluded | The article was excluded based on title and abstract screening as it did not meet the inclusion and exclusion criteria. |
| Immune-toxicity effects of scorpion venom on the hypothalamic pituitary adrenal axis during rest and activity phases in a rodent model | 2020 | Daachi, F. and Adi-Bessalem, S. and Megdad-Lamraoui, A. and Laraba-Djebari, F. | Excluded | The article was excluded based on title and abstract screening as it did not meet the inclusion and exclusion criteria. |
| Microglia and modifiable life factors: Potential contributions to cognitive resilience in aging | 2021 | Duggan, M.R. and Parikh, V. | Excluded | The article was excluded based on title and abstract screening as it did not meet the inclusion and exclusion criteria. |
| Neuroprotective effects of physical activity via the adaptation of astrocytes | 2021 | Maugeri, G. and D’agata, V. and Magrì, B. and Roggio, F. and Castorina, A. and Ravalli, S. and Di Rosa, M. and Musumeci, G. | Excluded | The article was excluded based on title and abstract screening as it did not meet the inclusion and exclusion criteria. |
| Comparing the effects of rosemary extract and treadmill exercise on the hippocampal function and antioxidant capacity in old rats | 2021 | Rasoolijazi, H. and Ofogh, S.N. and Ababzadeh, S. and Mehdizadeh, M. and Shabkhiz, F. | Excluded | The article was excluded based on title and abstract screening as it did not meet the inclusion and exclusion criteria. |
| Irisin Rescues Blood-Brain Barrier Permeability following Traumatic Brain Injury and Contributes to the Neuroprotection of Exercise in Traumatic Brain Injury | 2021 | Guo, P. and Jin, Z. and Wang, J. and Sang, A. and Wu, H. | Excluded | The article was excluded based on title and abstract screening as it did not meet the inclusion and exclusion criteria. |
| Long-Term Impact of Social Isolation and Molecular Underpinnings | 2020 | Arzate-Mejía, R.G. and Lottenbach, Z. and Schindler, V. and Jawaid, A. and Mansuy, I.M. | Excluded | The article was excluded based on title and abstract screening as it did not meet the inclusion and exclusion criteria. |
| The Reversal of Memory Deficits in an Alzheimer’s Disease Model Using Physical and Cognitive Exercise | 2020 | Dare, L.R. and Garcia, A. and Soares, C.B. and Lopes, L. and Neves, B.-H.S. and Dias, D.V. and Mello-Carpes, P.B. | Excluded | The article was excluded based on title and abstract screening as it did not meet the inclusion and exclusion criteria. |
| Idazoxan and efaroxan potentiate the endurance performances and the antioxidant activity of ephedrine in rats | 2021 | Rusu-Zota, G. and Burlui, A. and Rezus, E. and Paduraru, L. and Sorodoc, V. | Excluded | The article was excluded based on title and abstract screening as it did not meet the inclusion and exclusion criteria. |
| Targeting Keap1/Nrf2/ARE signaling pathway in multiple sclerosis | 2020 | Michaličková, D. and Hrnčíř, T. and Canová, N.K. and Slanař, O. | Excluded | The article was excluded based on title and abstract screening as it did not meet the inclusion and exclusion criteria. |
| Research progress and treatment strategies for anesthetic neurotoxicity | 2020 | Yang, F. and Zhao, H. and Zhang, K. and Wu, X. and Liu, H. | Excluded | The article was excluded based on title and abstract screening as it did not meet the inclusion and exclusion criteria. |
| Effects of high intensity interval training on neuro-cardiovascular dynamic changes and mitochondrial dysfunction induced by high-fat diet in rats | 2020 | Marques Neto, S.R. and Castiglione, R.C. and da Silva, T.C.B. and Paes, L.D.S. and Pontes, A. and Oliveira, D.F. and Ferraz, E.B. and Ade Caldas, C.C. and Nascimento, J.H.M. and Bouskela, E. | Excluded | The article was excluded based on title and abstract screening as it did not meet the inclusion and exclusion criteria. |
| Alterations in glutathione, nitric oxide and 3-nitrotyrosine levels following exercise and/or hyperbaric oxygen treatment in mice with diet-induced diabetes | 2020 | Gutiérrez-Camacho, L.R. and Kormanovski, A. and Del Castillo-Hernández, M.C. and Guevara-Balcázar, G. and Lara-Padilla, E. | Excluded | The article was excluded based on title and abstract screening as it did not meet the inclusion and exclusion criteria. |
| A Dual Role of ATM in Ischemic Preconditioning and Ischemic Injury | 2020 | Xie, G.-H. and Dai, H.-J. and Liu, F. and Zhang, Y.-P. and Zhu, L. and Nie, J.-J. and Wu, J.-H. | Excluded | The article was excluded based on title and abstract screening as it did not meet the inclusion and exclusion criteria. |
| Pharmacological connection of histamine-1 (H1) receptor mediated neuroprotective mechanism of ischemic preconditioning in rat | 2021 | Singh, P. and Sharma, B. | Excluded | The article was excluded based on title and abstract screening as it did not meet the inclusion and exclusion criteria. |
| Transcriptional changes involved in atrophying muscles during prolonged fasting in rats | 2020 | Ibrahim, M. and Wasselin, T. and Challet, E. and Van Dorsselaer, A. and Le Maho, Y. and Raclot, T. and Bertile, F. | Excluded | The article was excluded based on title and abstract screening as it did not meet the inclusion and exclusion criteria. |
| Mitochondrial Regulation of Microglial Immunometabolism in Alzheimer’s Disease | 2021 | Fairley, L.H. and Wong, J.H. and Barron, A.M. | Excluded | The article was excluded based on title and abstract screening as it did not meet the inclusion and exclusion criteria. |
| Hydrogen Sulfide Attenuates Renin Angiotensin and Aldosterone Pathological Signaling to Preserve Kidney Function and Improve Exercise Tolerance in Heart Failure | 2018 | Li, Z. and Organ, C.L. and Kang, J. and Polhemus, D.J. and Trivedi, R.K. and Sharp, T.E. and Jenkins, J.S. and Tao, Y.-X. and Xian, M. and Lefer, D.J. | Excluded | The article was excluded based on title and abstract screening as it did not meet the inclusion and exclusion criteria. |
| Effect of intensive exercise training and vitamin e supplementation on the content of rat brain neurotrophic factors | 2018 | Foadoddini, M. and Afzalpour, M.E. and TaheriChadorneshin, H. and Abtahi-Eivary, S.-H. | Included |  |
| Exercise-Induced Neuroprotection and Recovery of Motor Function in Animal Models of Parkinson's Disease | 2019 | Palasz, E. and Niewiadomski, W. and Gasiorowska, A. and Wysocka, A. and Stepniewska, A. and Niewiadomska, G. | Excluded | The article was excluded based on title and abstract screening as it did not meet the inclusion and exclusion criteria. |
| Blood-brain barrier permeability and physical exercise | 2019 | Małkiewicz, M.A. and Szarmach, A. and Sabisz, A. and Cubała, W.J. and Szurowska, E. and Winklewski, P.J. | Excluded | The article was excluded based on title and abstract screening as it did not meet the inclusion and exclusion criteria. |
| Exercise enhances the effectiveness of vitamin D therapy in rats with Alzheimer’s disease: emphasis on oxidative stress and inflammation | 2020 | Medhat, E. and Rashed, L. and Abdelgwad, M. and Aboulhoda, B.E. and Khalifa, M.M. and El-Din, S.S. | Excluded | The article was excluded based on title and abstract screening as it did not meet the inclusion and exclusion criteria. |
| Concentrations of the Selected Biomarkers of Endothelial Dysfunction in Response to Antiepileptic Drugs: A Literature Review | 2019 | Sarecka-Hujar, B. and Szołtysek-Bołdys, I. and Kopyta, I. and Dolińska, B. and Sobczak, A. | Excluded | The article was excluded based on title and abstract screening as it did not meet the inclusion and exclusion criteria. |
| Monosodium glutamate and treadmill exercise: Anxiety-like behavior and spreading depression features in young adult rats | 2019 | Vitor-de-Lima, S.M. and Medeiros, L.D.B. and Benevides, R.D.D.L. and dos Santos, C.N. and Lima da Silva, N.O. and Guedes, R.C.A. | Excluded | The article was excluded based on title and abstract screening as it did not meet the inclusion and exclusion criteria. |
| Proposed tandem effect of physical activity and sirtuin 1 and 3 activation in regulating glucose homeostasis | 2019 | Pacifici, F. and Di Cola, D. and Pastore, D. and Abete, P. and Guadagni, F. and Donadel, G. and Bellia, A. and Esposito, E. and Salimei, C. and Salimei, P.S. and Ricordi, C. and Lauro, D. and Della-Morte, D. | Excluded | The article was excluded based on title and abstract screening as it did not meet the inclusion and exclusion criteria. |
| Effects of aging and long-term physical activity on mitochondrial physiology and redox state of the cortex and cerebellum of female rats | 2022 | Mesquita, P.H.C. and Osburn, S.C. and Godwin, J.S. and Roberts, M.D. and Kavazis, A.N. | Included |  |
| Left ventricular hypertrophy: Roles of mitochondria CYP1B1 and melatonergic pathways in co-ordinatingwider pathophysiology | 2019 | Anderson, G. and Mazzoccoli, G. | Excluded | The article was excluded based on title and abstract screening as it did not meet the inclusion and exclusion criteria. |
| Avoiding Alzheimer’s disease: The important causative role of divalent copper ingestion | 2019 | Brewer, G.J. | Excluded | The article was excluded based on title and abstract screening as it did not meet the inclusion and exclusion criteria. |
| Mitochondrial dynamics, a key executioner in neurodegenerative diseases | 2019 | Panchal, K. and Tiwari, A.K. | Excluded | The article was excluded based on title and abstract screening as it did not meet the inclusion and exclusion criteria. |
| Exercise training upregulates Nrf2 protein in the rostral ventrolateral medulla of mice with heart failure | 2019 | Wafi, A.M. and Yu, L. and Gao, L. and Zucker, I.H. | Excluded | The article was excluded based on title and abstract screening as it did not meet the inclusion and exclusion criteria. |
| The effects of exercise on hippocampal inflammatory cytokine levels, brain oxidative stress markers and memory impairments induced by lipopolysaccharide in rats | 2019 | Jahangiri, Z. and Gholamnezhad, Z. and Hosseini, M. | Excluded | The article was excluded based on title and abstract screening as it did not meet the inclusion and exclusion criteria. |
| Molecular mechanisms of action of tocotrienols in cancer: Recent trends and advancements | 2019 | Aggarwal, V. and Kashyap, D. and Sak, K. and Tuli, H.S. and Jain, A. and Chaudhary, A. and Garg, V.K. and Sethi, G. and Yerer, M.B. | Excluded | The article was excluded based on title and abstract screening as it did not meet the inclusion and exclusion criteria. |
| Enhancement of hippocampal plasticity by physical exercise as a polypill for stress and depression: A review | 2019 | Li, A. and Yau, S.-Y. and Machado, S. and Wang, P. and Yuan, T.-F. and So, K.-F. | Excluded | The article was excluded based on title and abstract screening as it did not meet the inclusion and exclusion criteria. |
| Polyphenols as an effective therapeutic intervention against cognitive decline during normal and pathological brain aging | 2020 | Asha Devi, S. and Chamoli, A. | Excluded | The article was excluded based on title and abstract screening as it did not meet the inclusion and exclusion criteria. |
| Hydroxytyrosol, the major phenolic compound of olive oil, as an acute therapeutic strategy after ischemic stroke | 2019 | Calahorra, J. and Shenk, J. and Wielenga, V.H. and Verweij, V. and Geenen, B. and Dederen, P.J. and Peinado, M.Á. and Siles, E. and Wiesmann, M. and Kiliaan, A.J. | Excluded | The article was excluded based on title and abstract screening as it did not meet the inclusion and exclusion criteria. |
| The counteracting effects of exercise on high-fat diet-induced memory impairment: A systematic review | 2019 | Loprinzi, P.D. and Ponce, P. and Zou, L. and Li, H. | Excluded | The article was excluded based on title and abstract screening as it did not meet the inclusion and exclusion criteria. |
| α-Klotho Expression in Mouse Tissues Following Acute Exhaustive Exercise | 2019 | Rao, Z. and Zheng, L. and Huang, H. and Feng, Y. and Shi, R. | Excluded | The article was excluded based on title and abstract screening as it did not meet the inclusion and exclusion criteria. |
| Apitherapy for Parkinson's Disease: A Focus on the Effects of Propolis and Royal Jelly | 2020 | Ali, A.M. and Kunugi, H. | Excluded | The article was excluded based on title and abstract screening as it did not meet the inclusion and exclusion criteria. |
| Intermittent Fasting and Metabolic Switching: A Brief Overview | 2020 | Mishra, S. and Singh, B. | Excluded | The article was excluded based on title and abstract screening as it did not meet the inclusion and exclusion criteria. |
| Supplementation of Cannabis sativa L. Leaf powder accelerates functional recovery and ameliorates haemoglobin level following an induced injury to sciatic nerve in mouse model | 2019 | Aziz, N. and Rasul, A. and Malik, S.A. and Anwar, H. and Imran, A. and Razzaq, A. and Shaukat, A. and Kamran, S.K.S. and de Aguilar, J.-L.G. and Sun, T. and Hussain, G. | Excluded | The article was excluded based on title and abstract screening as it did not meet the inclusion and exclusion criteria. |
| Remote ischemic conditioning as a cytoprotective strategy in vasculopathies during hyperhomocysteinemia: An emerging research perspective | 2019 | Majumder, A. and Singh, M. and George, A.K. and Homme, R.P. and Laha, A. and Tyagi, S.C. | Excluded | The article was excluded based on title and abstract screening as it did not meet the inclusion and exclusion criteria. |
| The protective effects of green tea catechins in the management of neurodegenerative diseases: A review | 2019 | Farkhondeh, T. and Yazdi, H.S. and Samarghandian, S. | Excluded | The article was excluded based on title and abstract screening as it did not meet the inclusion and exclusion criteria. |
| Gender differences in nitric oxide and antioxidant response to physical stress in tissues of trained mice after hyperbaric oxygen preconditioning | 2019 | Kormanovski, A. and Castillo-Hernández, M.C. and Guevara-Balcázar, G. and Pérez, T. and Lara-Padilla, E. | Excluded | The article was excluded based on title and abstract screening as it did not meet the inclusion and exclusion criteria. |
| Modulatory effects of swimming exercise against malathion induced neurotoxicity in male and female rats | 2019 | Salama, O.A. and Attia, M.M. and Abdelrazek, M.A.S. | Excluded | The article was excluded based on title and abstract screening as it did not meet the inclusion and exclusion criteria. |
| TBC1D24-TLDc-related epilepsy exercise-induced dystonia: Rescue by antioxidants in a disease model | 2019 | Lüthy, K. and Mei, D. and Fischer, B. and De Fusco, M. and Swerts, J. and Paesmans, J. and Parrini, E. and Lubarr, N. and Meijer, I.A. and Mackenzie, K.M. and Lee, W.-T. and Cittaro, D. and Aridon, P. and Schoovaerts, N. and Versées, W. and Verstreken, P. and Casari, G. and Guerrini, R. | Excluded | The article was excluded based on title and abstract screening as it did not meet the inclusion and exclusion criteria. |
| New horizons of arterial stiffness developed using Cardio-Ankle Vascular Index (CAVI) | 2020 | Saiki, A. and Ohira, M. and Yamaguchi, T. and Nagayama, D. and Shimizu, N. and Shirai, K. and Tatsuno, I. | Excluded | The article was excluded based on title and abstract screening as it did not meet the inclusion and exclusion criteria. |
| Brain ageing and neurodegenerative disease: The role of cellular waste management | 2018 | Daniele, S. and Giacomelli, C. and Martini, C. | Excluded | The article was excluded based on title and abstract screening as it did not meet the inclusion and exclusion criteria. |
| Exercise training improves age-related changes in cerebral capillary vascularity through the upregulation of PI3K / Akt signaling | 2019 | Chanpakdee, C. and Viboolvorakul, S. and Patumraj, S. | Excluded | The article was excluded based on title and abstract screening as it did not meet the inclusion and exclusion criteria. |
| Mechanisms underlying metabolic syndrome-related sarcopenia and possible therapeutic measures | 2019 | Rubio-Ruiz, M.E. and Guarner-Lans, V. and Pérez-Torres, I. and Soto, M.E. | Excluded | The article was excluded based on title and abstract screening as it did not meet the inclusion and exclusion criteria. |
| Physical Exercise During Pregnancy Prevents Cognitive Impairment Induced by Amyloid-β in Adult Offspring Rats | 2019 | Klein, C.P. and Hoppe, J.B. and Saccomori, A.B. and dos Santos, B.G. and Sagini, J.P. and Crestani, M.S. and August, P.M. and Hözer, R.M. and Grings, M. and Parmeggiani, B. and Leipnitz, G. and Navas, P. and Salbego, C.G. and Matté, C. | Excluded | The article was excluded based on title and abstract screening as it did not meet the inclusion and exclusion criteria. |
| Diet-Induced Modification of the Sperm Epigenome Programs Metabolism and Behavior | 2020 | Bodden, C. and Hannan, A.J. and Reichelt, A.C. | Excluded | The article was excluded based on title and abstract screening as it did not meet the inclusion and exclusion criteria. |
| Reduced chronic restraint stress in mice overexpressing hyperactive proteasomes in the forebrain | 2020 | Kim, J.H. and Kim, A. and Yun, Y. and Park, S. and Lee, J.H. and Lee, Y.-S. and Lee, M.J. | Excluded | The article was excluded based on title and abstract screening as it did not meet the inclusion and exclusion criteria. |
| The Role of Redox Dysregulation in the Effects of Prenatal Stress on Embryonic Interneuron Migration | 2019 | Bittle, J. and Menezes, E.C. and McCormick, M.L. and Spitz, D.R. and Dailey, M. and Stevens, H.E. | Excluded | The article was excluded based on title and abstract screening as it did not meet the inclusion and exclusion criteria. |
| The effects of exercise fatigue on mglur1 expression and oxidative stress in striatum of rat | 2020 | Su, M. and Xu, D. and Chen, J. | Excluded | The article was excluded based on title and abstract screening as it did not meet the inclusion and exclusion criteria. |
| Resveratrol and its effects on the vascular system | 2019 | Breuss, J.M. and Atanasov, A.G. and Uhrin, P. | Excluded | The article was excluded based on title and abstract screening as it did not meet the inclusion and exclusion criteria. |
| Exercise and metformin counteract altered mitochondrial function in the insulinresistant brain | 2019 | Ruegsegger, G.N. and Vanderboom, P.M. and Dasari, S. and Klaus, K.A. and Kabiraj, P. and McCarthy, C.B. and Lucchinetti, C.F. and Nair, K.S. | Excluded | The article was excluded based on title and abstract screening as it did not meet the inclusion and exclusion criteria. |
| Potential widespread denitrosylation of brain proteins following prolonged restraint: proposed links between stress and central nervous system disease | 2019 | Foley, T.D. and Koval, K.S. and Gallagher, A.G. and Olsen, S.H. | Excluded | The article was excluded based on title and abstract screening as it did not meet the inclusion and exclusion criteria. |
| Physical exercise positively modulates DOX-induced hepatic oxidative stress, mitochondrial dysfunction and quality control signaling | 2019 | Santos-Alves, E. and Rizo-Roca, D. and Marques-Aleixo, I. and Coxito, P. and Martins, S. and Guimarães, J.T. and Oliveira, P.J. and Torrella, J.R. and Magalhães, J. and Ascensão, A. | Excluded | The article was excluded based on title and abstract screening as it did not meet the inclusion and exclusion criteria. |
| Doxorubicin and cisplatin induced cognitive impairment: The possible mechanisms and interventions | 2020 | Ongnok, B. and Chattipakorn, N. and Chattipakorn, S.C. | Excluded | The article was excluded based on title and abstract screening as it did not meet the inclusion and exclusion criteria. |
| The metabolic face of migraine — from pathophysiology to treatment | 2019 | Gross, E.C. and Lisicki, M. and Fischer, D. and Sándor, P.S. and Schoenen, J. | Excluded | The article was excluded based on title and abstract screening as it did not meet the inclusion and exclusion criteria. |
| Microbiota Alterations in Alzheimer’s Disease: Involvement of the Kynurenine Pathway and Inflammation | 2019 | Garcez, M.L. and Jacobs, K.R. and Guillemin, G.J. | Excluded | The article was excluded based on title and abstract screening as it did not meet the inclusion and exclusion criteria. |
| Protective effects of spirulina platensis, voluntary exercise and environmental interventions against adolescent stress-induced anxiety and depressive-like symptoms, oxidative stress and alterations of bdnf and 5ht-3 receptors of the prefrontal cortex in female rats | 2020 | Moradi-Kor, N. and Dadkhah, M. and Ghanbari, A. and Rashidipour, H. and Bandegi, A.R. and Barati, M. and Kokhaei, P. and Rashidy-Pour, A. | Excluded | The article was excluded based on title and abstract screening as it did not meet the inclusion and exclusion criteria. |
| Exercise and Parkinson's disease | 2019 | Xu, X. and Fu, Z. and Le, W. and Yau S.-Y. and So K.-F. | Excluded | The article was excluded based on title and abstract screening as it did not meet the inclusion and exclusion criteria. |
| Reduced neuroinflammation and improved functional recovery after traumatic brain injury by prophylactic diet supplementation in mice | 2019 | Yu, J. and Zhu, H. and Taheri, S. and Monday, W. and Perry, S. and Kindy, M.S. | Excluded | The article was excluded based on title and abstract screening as it did not meet the inclusion and exclusion criteria. |
| Exercise, diet and stress as modulators of gut microbiota: Implications for neurodegenerative diseases | 2020 | Gubert, C. and Kong, G. and Renoir, T. and Hannan, A.J. | Excluded | The article was excluded based on title and abstract screening as it did not meet the inclusion and exclusion criteria. |
| From obesity to hippocampal neurodegeneration: Pathogenesis and non-pharmacological interventions | 2020 | Lee, T.H.-Y. and Yau, S.-Y. | Excluded | The article was excluded based on title and abstract screening as it did not meet the inclusion and exclusion criteria. |
| Protective effects of taurine against inflammation, apoptosis, and oxidative stress in brain injury | 2018 | Niu, X. and Zheng, S. and Liu, H. and Li, S. | Excluded | The article was excluded based on title and abstract screening as it did not meet the inclusion and exclusion criteria. |
| Chemotherapy and cognition: comprehensive review on doxorubicin-induced chemobrain | 2019 | El-Agamy, S.E. and Abdel-Aziz, A.K. and Esmat, A. and Azab, S.S. | Excluded | The article was excluded based on title and abstract screening as it did not meet the inclusion and exclusion criteria. |
| The therapeutic effect of exercise on anxiety and bowel oxidative stress in the maternal separation animal model | 2020 | Khorjahani, A. and Peeri, M. and Azarbayjani, M.A. | Excluded | The article was excluded based on title and abstract screening as it did not meet the inclusion and exclusion criteria. |
| Cardiac remodelling in a swine model of chronic thromboembolic pulmonary hypertension: comparison of right vs. left ventricle | 2019 | Stam, K. and Cai, Z. and van der Velde, N. and van Duin, R. and Lam, E. and van der Velden, J. and Hirsch, A. and Duncker, D.J. and Merkus, D. | Excluded | The article was excluded based on title and abstract screening as it did not meet the inclusion and exclusion criteria. |
| The relationship between diet and frailty in aging | 2020 | Gimeno-Mallench, L. and Sanchez-Morate, E. and Parejo-Pedrajas, S. and Mas-Bargues, C. and Inglés, M. and Sanz-Ros, J. and Román-Domínguez, A. and Olaso, G. and Stromsnes, K. and Gambini, J. | Excluded | The article was excluded based on title and abstract screening as it did not meet the inclusion and exclusion criteria. |
| Postinfarction exercise training alleviates cardiac dysfunction and adverse remodeling via mitochondrial biogenesis and SIRT1/PGC-1α/PI3K/Akt signaling | 2019 | Jia, D. and Hou, L. and Lv, Y. and Xi, L. and Tian, Z. | Excluded | The article was excluded based on title and abstract screening as it did not meet the inclusion and exclusion criteria. |
| The novel perspectives of adipokines on brain health | 2019 | Lee, T.H.-Y. and Cheng, K.K.-Y. and Hoo, R.L.-C. and Siu, P.M.-F. and Yau, S.-Y. | Excluded | The article was excluded based on title and abstract screening as it did not meet the inclusion and exclusion criteria. |
| Silibinin Alleviates the Learning and Memory Defects in Overtrained Rats Accompanying Reduced Neuronal Apoptosis and Senescence | 2019 | Liu, B. and Liu, W. and Liu, P. and Liu, X. and Song, X. and Hayashi, T. and Onodera, S. and Ikejima, T. | Excluded | The article was excluded based on title and abstract screening as it did not meet the inclusion and exclusion criteria. |
| Voluntary, involuntary and forced exercises almost equally reverse behavioral impairment by regulating hippocampal neurotrophic factors and oxidative stress in experimental Alzheimer's disease model | 2019 | Belviranlı, M. and Okudan, N. | Excluded | The article was excluded based on title and abstract screening as it did not meet the inclusion and exclusion criteria. |
| Effect of Exercise Interventions on Kainate Induced Status Epilepticus and Associated Co-morbidities; A Systematic Review and Meta-Analysis | 2019 | Iqbal, M. and Ullah, S. and Zafar, S. and Nisar, T. and Liu, J.-X. and Liu, Y. | Excluded | The article was excluded based on title and abstract screening as it did not meet the inclusion and exclusion criteria. |
| Exercise Mitigates Alcohol Induced Endoplasmic Reticulum Stress Mediated Cognitive Impairment through ATF6-Herp Signaling | 2018 | George, A.K. and Behera, J. and Kelly, K.E. and Mondal, N.K. and Richardson, K.P. and Tyagi, N. | Excluded | The article was excluded based on title and abstract screening as it did not meet the inclusion and exclusion criteria. |
| Prevention and Treatment of Alzheimer's Disease: Biological Mechanisms of Exercise | 2019 | McGurran, H. and Glenn, J.M. and Madero, E.N. and Bott, N.T. | Excluded | The article was excluded based on title and abstract screening as it did not meet the inclusion and exclusion criteria. |
| Myeloperoxidase and septic conditions disrupt sphingolipid homeostasis in murine brain capillaries in vivo and immortalized human brain endothelial cells in vitro | 2020 | Goeritzer, M. and Bernhart, E. and Plastira, I. and Reicher, H. and Leopold, C. and Eichmann, T.O. and Rechberger, G. and Madreiter-Sokolowski, C.T. and Prasch, J. and Eller, P. and Graier, W.F. and Kratky, D. and Malle, E. and Sattler, W. | Excluded | The article was excluded based on title and abstract screening as it did not meet the inclusion and exclusion criteria. |
| Antiaging interventions: An insight into polyphenols and brain aging | 2018 | Devi, S.A. and Sekhar, S.R. | Excluded | The article was excluded based on title and abstract screening as it did not meet the inclusion and exclusion criteria. |
| Healthy aging: A bibliometric analysis of the literature | 2019 | Gu, Y.-H. and Bai, J.-B. and Chen, X.-L. and Wu, W.-W. and Liu, X.-X. and Tan, X.-D. | Excluded | The article was excluded based on title and abstract screening as it did not meet the inclusion and exclusion criteria. |
| BDNF is a mediator of glycolytic fiber-type specification in mouse skeletal muscle | 2019 | Delezie, J. and Weihrauch, M. and Maier, G. and Tejero, R. and Ham, D.J. and Gill, J.F. and Karrer-Cardel, B. and Rüegg, M.A. and Tabares, L. and Handschin, C. | Excluded | The article was excluded based on title and abstract screening as it did not meet the inclusion and exclusion criteria. |
| Effects of endurance physical training on hydroxyl radical generation in rat tissues | 1998 | Itoh, Hiroshi and Ohkuwa, Tetsuo and Yamamoto, Takako and Sato, Yuzo and Miyamura, Miharu and Naoi, Makoto | Included |  |
| Protective Effects of Yiqi Xingnao Oral Liquid on Cerebral Ischemia-Reperfusion Injury in Rats and Its Related Mechanisms | 2020 | Pang, T. and Zhao, J. and Zhang, F. and Piao, S. and Yang, H. and Jiao, J. and Tang, L. and Lu, W. and Chen, W. | Excluded | The article was excluded based on title and abstract screening as it did not meet the inclusion and exclusion criteria. |
| Impact of ketone salt containing supplement on cardiorespiratory and oxidative stress response in firefighters exercising in personal protective equipment | 2019 | McAllister, M.J. and Holland, A.M. and Chander, H. and Waldman, H.S. and Smith, J.W. and Basham, S.A. | Excluded | The article was excluded based on title and abstract screening as it did not meet the inclusion and exclusion criteria. |
| The Structure and the Regulation of Glycogen Phosphorylases in Brain | 2019 | Mathieu, C. and Dupret, J.-M. and Rodrigues-Lima, F. | Excluded | The article was excluded based on title and abstract screening as it did not meet the inclusion and exclusion criteria. |
| Brown rice-specific γ-oryzanol as a promising prophylactic avenue to protect against diabetes mellitus and obesity in humans | 2019 | Masuzaki, H. and Kozuka, C. and Okamoto, S. and Yonamine, M. and Tanaka, H. and Shimabukuro, M. | Excluded | The article was excluded based on title and abstract screening as it did not meet the inclusion and exclusion criteria. |
| Repeated Restraint Stress Decreases Na,K-ATPase Activity via Oxidative and Nitrosative Damage in the Frontal Cortex of Rats | 2018 | Novaes, L.S. and dos Santos, N.B. and Dragunas, G. and Perfetto, J.G. and Leza, J.C. and Scavone, C. and Munhoz, C.D. | Excluded | The article was excluded based on title and abstract screening as it did not meet the inclusion and exclusion criteria. |
| Neuroprotective effects of exercise in rodent models of memory deficit and Alzheimer's | 2019 | Jahangiri, Z. and Gholamnezhad, Z. and Hosseini, M. | Excluded | The article was excluded based on title and abstract screening as it did not meet the inclusion and exclusion criteria. |
| Salidroside protects the cardiac function of exhausted rats by inducing Nrf2 expression | 2020 | Xu, P. and Wang, Y. and Sun, W. and Sun, Y. and Lu, W. and Chang, Y. and Ping, Z. and Li, Y. and Cao, X. | Excluded | The article was excluded based on title and abstract screening as it did not meet the inclusion and exclusion criteria. |
| Oxidative stress in exercise training: the involvement of inflammation and peripheral signals | 2019 | Magherini, F. and Fiaschi, T. and Marzocchini, R. and Mannelli, M. and Gamberi, T. and Modesti, P.A. and Modesti, A. | Excluded | The article was excluded based on title and abstract screening as it did not meet the inclusion and exclusion criteria. |
| Effects of forced exercise on spatial memory and cytochrome c oxidase activity in aged rats | 2013 | Sampedro-Piquero, P and Zancada-Menendez, C and Begega, A and Mendez, M and Arias, JL | Included |  |
| The selenium-containing compound 3-((4-chlorophenyl)selanyl)-1-methyl-1H-indole reverses depressive-like behavior induced by acute restraint stress in mice: modulation of oxido-nitrosative stress and inflammatory pathway | 2019 | Casaril, A.M. and Domingues, M. and Bampi, S.R. and de Andrade Lourenço, D. and Padilha, N.B. and Lenardão, E.J. and Sonego, M. and Seixas, F.K. and Collares, T. and Nogueira, C.W. and Dantzer, R. and Savegnago, L. | Excluded | The article was excluded based on title and abstract screening as it did not meet the inclusion and exclusion criteria. |
| Regulation of sympathetic vasomotor activity by the hypothalamic paraventricular nucleus in normotensive and hypertensive states | 2018 | Dampney, R.A. and Michelini, L.C. and Li, D.-P. and Pan, H.-L. | Excluded | The article was excluded based on title and abstract screening as it did not meet the inclusion and exclusion criteria. |
| Comparing the Effects of Chlorogenic Acid and Ilex paraguariensis Extracts on Different Markers of Brain Alterations in Rats Subjected to Chronic Restraint Stress | 2019 | de Lima, M.E. and Ceolin Colpo, A.Z. and Maya-López, M. and Rangel-López, E. and Becerril-Chávez, H. and Galván-Arzate, S. and Villeda-Hernández, J. and Sánchez-Chapul, L. and Túnez, I. and Folmer, V. and Santamaría, A. | Excluded | The article was excluded based on title and abstract screening as it did not meet the inclusion and exclusion criteria. |
| Anti-ageing effect of Physalis alkekengi ethyl acetate layer on a d-galactose-induced mouse model through the reduction of cellular senescence and oxidative stress | 2020 | Sun, K. and Sun, Y. and Li, H. and Han, D. and Bai, Y. and Zhao, R. and Guo, Z. | Excluded | The article was excluded based on title and abstract screening as it did not meet the inclusion and exclusion criteria. |
| Astaxanthin: A Potential Mitochondrial-Targeted Antioxidant Treatment in Diseases and with Aging | 2019 | Sztretye, M. and Dienes, B. and Gönczi, M. and Czirják, T. and Csernoch, L. and Dux, L. and Szentesi, P. and Keller-Pintér, A. | Excluded | The article was excluded based on title and abstract screening as it did not meet the inclusion and exclusion criteria. |
| Emerging mechanistic underpinnings and therapeutic targets for chemotherapy-related cognitive impairment | 2019 | Gibson, E.M. and Monje, M. | Excluded | The article was excluded based on title and abstract screening as it did not meet the inclusion and exclusion criteria. |
| Synergistic effects of quercetin and regular exercise on the recovery of spatial memory and reduction of parameters of oxidative stress in an animal model of alzheimer’s disease | 2020 | Molaei, A. and Hatami, H. and Dehghan, G. and Sadeghian, R. and Khajehnasiri, N. | Excluded | The article was excluded based on title and abstract screening as it did not meet the inclusion and exclusion criteria. |
| Effect of exercise on type 2 diabetes-associated cognitive impairment in rats | 2019 | Mehta, B.K. and Singh, K.K. and Banerjee, S. | Excluded | The article was excluded based on title and abstract screening as it did not meet the inclusion and exclusion criteria. |
| Effects of capsinoids on daily physical activity, body composition and cold hypersensitivity in middle-aged and older adults: A randomized study | 2020 | Yokoyama, K. and Yamada, Y. and Akamatsu, Y. and Yoshinaka, Y. and Yamamoto, A. and Koizumi, T. and Ohyama, K. and Suzuki, K. and Hashimoto, M. and Sato, H. and Kimura, M. | Excluded | The article was excluded based on title and abstract screening as it did not meet the inclusion and exclusion criteria. |
| Nutrients in the Prevention of Alzheimer's Disease | 2019 | Cremonini, A.L. and Caffa, I. and Cea, M. and Nencioni, A. and Odetti, P. and Monacelli, F. | Excluded | The article was excluded based on title and abstract screening as it did not meet the inclusion and exclusion criteria. |
| Endurance training on rodent brain antioxidant capacity: A meta-analysis | 2019 | de Souza, R.F. and de Moraes, S.R.A. and Augusto, R.L. and de Freitas Zanona, A. and Matos, D. and Aidar, F.J. and da Silveira Andrade-da-Costa, B.L. | Included |  |
| Aerobic Physical Training Protects the Rat Brain Against Exercise-Heat Related Oxidative Damage through the Increased Expression of HSP70 | 2019 | Wang, D. and Ripley-Gonzalez, J.W. and Hu, Y. | Excluded | The article was excluded based on title and abstract screening as it did not meet the inclusion and exclusion criteria. |
| Application of Molecular Hydrogen as a Novel Antioxidant in Sports Science | 2020 | Kawamura, T. and Higashida, K. and Muraoka, I. | Excluded | The article was excluded based on title and abstract screening as it did not meet the inclusion and exclusion criteria. |
| Deletion of Bmal1 prevents diet-induced ectopic fat accumulation by controlling oxidative capacity in the skeletal muscle | 2018 | Wada, T. and Ichihashi, Y. and Suzuki, E. and Kosuge, Y. and Ishige, K. and Uchiyama, T. and Makishima, M. and Nakao, R. and Oishi, K. and Shimba, S. | Excluded | The article was excluded based on title and abstract screening as it did not meet the inclusion and exclusion criteria. |
| Is there a role for whole body vibration in protecting cardiovascular disease? | 2019 | Zornoff, L. and Minicucci, M.F. | Excluded | The article was excluded based on title and abstract screening as it did not meet the inclusion and exclusion criteria. |
| Aerobic physical exercise as a neuroprotector strategy for ethanol binge-drinking effects in the hippocampus and systemic redox status in rats | 2019 | Pamplona-Santos, D. and Lamarão-Vieira, K. and Nascimento, P.C. and Bittencourt, L.O. and Corrêa, M.G. and Dos Santos, S.M. and Cartágenes, S.C. and Fernandes, L.M.P. and Monteiro, M.C. and Maia, C.S.F. and Lima, R.R. | Excluded | The article was excluded based on title and abstract screening as it did not meet the inclusion and exclusion criteria. |
| Epigenetic effects of male obesity on sperm and offspring | 2018 | Zhou, Y. and Wu, H. and Huang, H. | Excluded | The article was excluded based on title and abstract screening as it did not meet the inclusion and exclusion criteria. |
| Evidences that maternal swimming exercise improves antioxidant defenses and induces mitochondrial biogenesis in the brain of young Wistar rats | 2013 | Marcelino, T.B. and Longoni, A. and Kudo, K.Y. and Stone, V. and Rech, A. and De Assis, A.M. and Scherer, E.B.S. and Da Cunha, M.J. and Wyse, A.T.S. and Pettenuzzo, L.F. and Leipnitz, G. and Matté, C. | Included |  |
| Effects of previous physical exercise to chronic stress on long-term aversive memory and oxidative stress in amygdala and hippocampus of rats | 2017 | dos Santos, T.M. and Kolling, J. and Siebert, C. and Biasibetti, H. and Bertó, C.G. and Grun, L.K. and Dalmaz, C. and Barbé-Tuana, F.M. and Wyse, A.T.S. | Excluded | The article was excluded based on title and abstract screening as it did not meet the inclusion and exclusion criteria. |
| Novel neuroprotective role of hydrogen sulfide in a rat model of stress brain injury | 2018 | Elbassuoni, E.A. and Nazmy, W.H. | Excluded | The article was excluded based on title and abstract screening as it did not meet the inclusion and exclusion criteria. |
| Erythropoietin prevents the effect of chronic restraint stress on the number of hippocampal CA3c dendritic terminals—relation to expression of genes involved in synaptic plasticity, angiogenesis, inflammation, and oxidative stress in male rats | 2018 | Aalling, N. and Hageman, I. and Miskowiak, K. and Orlowski, D. and Wegener, G. and Wortwein, G. | Excluded | The article was excluded based on title and abstract screening as it did not meet the inclusion and exclusion criteria. |
| C 60 fullerene prevents restraint stress-induced oxidative disorders in rat tissues: Possible involvement of the Nrf2/ARE-antioxidant pathway | 2018 | Gonchar, O.O. and Maznychenko, A.V. and Bulgakova, N.V. and Vereshchaka, I.V. and Tomiak, T. and Ritter, U. and Prylutskyy, Y.I. and Mankovska, I.M. and Kostyukov, A.I. | Excluded | The article was excluded based on title and abstract screening as it did not meet the inclusion and exclusion criteria. |
| Oxidative stress and inflammation: Their role in the pathogenesis of peripheral artery disease with or without type 2 diabetes mellitus | 2018 | Signorelli, S.S. and Katsiki, N. | Excluded | The article was excluded based on title and abstract screening as it did not meet the inclusion and exclusion criteria. |
| Long-lasting effects of fluoxetine and/or exercise augmentation on bio-behavioural markers of depression in pre-pubertal stress sensitive rats | 2017 | Schoeman, J.C. and Steyn, S.F. and Harvey, B.H. and Brink, C.B. | Excluded | The article was excluded based on title and abstract screening as it did not meet the inclusion and exclusion criteria. |
| Cardiac aging – benefits of exercise, Nrf2 activation and antioxidant signaling | 2017 | Narasimhan, M. and Rajasekaran, N.-S. | Excluded | The article was excluded based on title and abstract screening as it did not meet the inclusion and exclusion criteria. |
| eHSP70/iHSP70 and divergent functions on the challenge: effect of exercise and tissue specificity in response to stress | 2017 | Bittencourt, A. and Porto, R.R. | Excluded | The article was excluded based on title and abstract screening as it did not meet the inclusion and exclusion criteria. |
| NANOGOLD – BIOLOGICAL EFFECTS AND OCCUPATIONAL EXPOSURE LEVELS | 2017 | Świdwińska-Gajewska, A.M. and Czerczak, S. | Excluded | The article was excluded based on title and abstract screening as it did not meet the inclusion and exclusion criteria. |
| The ethanol extract of Aquilariae Lignum ameliorates hippocampal oxidative stress in a repeated restraint stress mouse model | 2017 | Lee, H.-Y. and Lee, J.-S. and Kim, H.-G. and Kim, W.-Y. and Lee, S.-B. and Choi, Y.-H. and Son, C.-G. | Excluded | The article was excluded based on title and abstract screening as it did not meet the inclusion and exclusion criteria. |
| Modulation of mitochondrial dynamics by treadmill training to improve gait and mitochondrial deficiency in a rat model of Parkinson's disease | 2017 | Chuang, C.-S. and Chang, J.-C. and Cheng, F.-C. and Liu, K.-H. and Su, H.-L. and Liu, C.-S. | Excluded | The article was excluded based on title and abstract screening as it did not meet the inclusion and exclusion criteria. |
| Physical Activity Modulates Common Neuroplasticity Substrates in Major Depressive and Bipolar Disorder | 2017 | Phillips, C. | Excluded | The article was excluded based on title and abstract screening as it did not meet the inclusion and exclusion criteria. |
| Static Magnetic Fields Modulate the Response of Different Oxidative Stress Markers in a Restraint Stress Model Animal | 2018 | Coballase-Urrutia, E. and Navarro, L. and Ortiz, J.L. and Verdugo-DÍaz, L. and Gallardo, J.M. and Hernández, M.E. and Estrada-Rojo, F. | Excluded | The article was excluded based on title and abstract screening as it did not meet the inclusion and exclusion criteria. |
| Role of oxidative stress and antioxidants in daily nutrition and human health | 2017 | Bjørklund, G. and Chirumbolo, S. | Excluded | The article was excluded based on title and abstract screening as it did not meet the inclusion and exclusion criteria. |
| Swimming attenuates D-galactose-induced brain aging via suppressing miR-34a-mediated autophagy impairment and abnormal mitochondrial dynamics | 2017 | Kou, X. and Li, J. and Liu, X. and Chang, J. and Zhao, Q. and Jia, S. and Fan, J. and Chen, N. | Excluded | The article was excluded based on title and abstract screening as it did not meet the inclusion and exclusion criteria. |
| Matrine attenuates D-galactose-induced aging-related behavior in mice via inhibition of cellular senescence and oxidative stress | 2018 | Sun, K. and Yang, P. and Zhao, R. and Bai, Y. and Guo, Z. | Excluded | The article was excluded based on title and abstract screening as it did not meet the inclusion and exclusion criteria. |
| Interactions between stress and physical activity on Alzheimer's disease pathology | 2018 | Yuede, C.M. and Timson, B.F. and Hettinger, J.C. and Yuede, K.M. and Edwards, H.M. and Lawson, J.E. and Zimmerman, S.D. and Cirrito, J.R. | Excluded | The article was excluded based on title and abstract screening as it did not meet the inclusion and exclusion criteria. |
| Charting the perfect storm: emerging biological interfaces between stress and stroke | 2017 | Kronenberg, G. and Schöner, J. and Nolte, C. and Heinz, A. and Endres, M. and Gertz, K. | Excluded | The article was excluded based on title and abstract screening as it did not meet the inclusion and exclusion criteria. |
| Neuroprotective effect of treadmill exercise against blunted brain insulin signaling, NADPH oxidase, and Tau hyperphosphorylation in rats fed a high-fat diet | 2018 | Jeong, J.-H. and Koo, J.-H. and Cho, J.-Y. and Kang, E.-B. | Excluded | The article was excluded based on title and abstract screening as it did not meet the inclusion and exclusion criteria. |
| Effect of diets with goat milk fat supplemented with exercise on anxiety and oxidative stress in the brains of adult rats | 2018 | Barbosa, M.Q. and Queiroga, R.D.C.R.E. and Bertozzo, C.C.D.M.S. and Araújo, D.F.D.S. and Oliveira, L.I.G. and Silva, J.Y.P. and Bomfim, M.A.D. and Guerra, G.C.B. and Costa, S. and Bessa, R. and Alves, S. and Barbosa Soares, J.K. | Excluded | The article was excluded based on title and abstract screening as it did not meet the inclusion and exclusion criteria. |
| The beneficial role of early exercise training following stroke and possible mechanisms | 2018 | Xing, Y. and Yang, S.-D. and Dong, F. and Wang, M.-M. and Feng, Y.-S. and Zhang, F. | Excluded | The article was excluded based on title and abstract screening as it did not meet the inclusion and exclusion criteria. |
| Aerobic exercise prevents rarefaction of pial collaterals and increased stroke severity that occur with aging | 2017 | Rzechorzek, W. and Zhang, H. and Buckley, B.K. and Hua, K. and Pomp, D. and Faber, J.E. | Excluded | The article was excluded based on title and abstract screening as it did not meet the inclusion and exclusion criteria. |
| Compounds from ilex paraguariensis extracts have antioxidant effects in the brains of rats subjected to chronic immobilization stress | 2017 | Colpo, A.C. and de Lima, M.E. and Maya-López, M. and Rosa, H. and Márquez-Curiel, C. and Galván-Arzate, S. and Santamaría, A. and Folmer, V. | Excluded | The article was excluded based on title and abstract screening as it did not meet the inclusion and exclusion criteria. |
| Previous physical exercise alters the hepatic profile of oxidative-inflammatory status and limits the secondary brain damage induced by severe traumatic brain injury in rats | 2017 | de Castro, M.R.T. and Ferreira, A.P.D.O. and Busanello, G.L. and da Silva, L.R.H. and da Silveira Junior, M.E.P. and Fiorin, F.D.S. and Arrifano, G. and Crespo-López, M.E. and Barcelos, R.P. and Cuevas, M.J. and Bresciani, G. and González-Gallego, J. and Fighera, M.R. and Royes, L.F.F. | Excluded | The article was excluded based on title and abstract screening as it did not meet the inclusion and exclusion criteria. |
| High-density lipoprotein (Hdl) cholesterol– more complicated than we think? | 2018 | Nessler, K. and Windak, A. and Grzybczak, R. and Nessler, M.B. and Siniarski, A. and Gajos, G. | Excluded | The article was excluded based on title and abstract screening as it did not meet the inclusion and exclusion criteria. |
| Treadmill running prevents age-related memory deficit and alters neurotrophic factors and oxidative damage in the hippocampus of Wistar rats | 2017 | Vanzella, C. and Neves, J.D. and Vizuete, A.F. and Aristimunha, D. and Kolling, J. and Longoni, A. and Gonçalves, C.A.S. and Wyse, A.T.S. and Netto, C.A. | Excluded | The article was excluded based on title and abstract screening as it did not meet the inclusion and exclusion criteria. |
| 2,3,5,4′-Tetrahydroxystilbene-2-O-beta-D-glucoside reverses stress-induced depression via inflammatory and oxidative stress pathways | 2018 | Jiang, C.-Y. and Qin, X.-Y. and Yuan, M.-M. and Lu, G.-J. and Cheng, Y. | Excluded | The article was excluded based on title and abstract screening as it did not meet the inclusion and exclusion criteria. |
| Royal jelly decreases corticosterone levels and improves the brain antioxidant system in restraint and cold stressed rats | 2017 | Teixeira, R.R. and de Souza, A.V. and Peixoto, L.G. and Machado, H.L. and Caixeta, D.C. and Vilela, D.D. and Baptista, N.B. and Franci, C.R. and Espindola, F.S. | Excluded | The article was excluded based on title and abstract screening as it did not meet the inclusion and exclusion criteria. |
| An endothelial link between the benefits of physical exercise in dementia | 2017 | Trigiani, L.J. and Hamel, E. | Excluded | The article was excluded based on title and abstract screening as it did not meet the inclusion and exclusion criteria. |
| Antioxidant action of grape seed polyphenols and aerobic exercise in improving neuronal number in the hippocampus is associated with decrease in lipid peroxidation and hydrogen peroxide in adult and middle-aged rats | 2018 | Abhijit, S. and Tripathi, S.J. and Bhagya, V. and Shankaranarayana Rao, B.S. and Subramanyam, M.V. and Asha Devi, S. | Excluded | The article was excluded based on title and abstract screening as it did not meet the inclusion and exclusion criteria. |
| Milestones in 200 years of Parkinson's disease research | 2017 | Patrik, B. | Excluded | The article was excluded based on title and abstract screening as it did not meet the inclusion and exclusion criteria. |
| Understanding the role of dysfunctional and healthy mitochondria in stroke pathology and its treatment | 2018 | Nguyen, H. and Zarriello, S. and Rajani, M. and Tuazon, J. and Napoli, E. and Borlongan, C.V. | Excluded | The article was excluded based on title and abstract screening as it did not meet the inclusion and exclusion criteria. |
| Effects of Physical Activity and Ginkgo Biloba on Cognitive Function and Oxidative Stress Modulation in Ischemic Rats | 2017 | Vaghef, L. and Bafandeh Gharamaleki, H. | Excluded | The article was excluded based on title and abstract screening as it did not meet the inclusion and exclusion criteria. |
| Neuroprotective effect of Clerodendrum serratum Linn. leaves extract against acute restraint stress-induced depressive-like behavioral symptoms in adult mice | 2017 | Vazhayil, B.K. and Rajagopal, S.S. and Thangavelu, T. and Swaminathan, G. and Rajagounder, E. | Excluded | The article was excluded based on title and abstract screening as it did not meet the inclusion and exclusion criteria. |
| Necrotizing enterocolitis is associated with acute brain responses in preterm pigs | 2018 | Sun, J. and Pan, X. and Christiansen, L.I. and Yuan, X.-L. and Skovgaard, K. and Chatterton, D.E.W. and Kaalund, S.S. and Gao, F. and Sangild, P.T. and Pankratova, S. | Excluded | The article was excluded based on title and abstract screening as it did not meet the inclusion and exclusion criteria. |
| Physical enrichment enhances memory function by regulating stress hormone and brain acetylcholinesterase activity in rats exposed to restraint stress | 2018 | Nawaz, A. and Batool, Z. and Shazad, S. and Rafiq, S. and Afzal, A. and Haider, S. | Excluded | The article was excluded based on title and abstract screening as it did not meet the inclusion and exclusion criteria. |
| Protective effect of low dose caffeine on psychological stress and cognitive function | 2017 | Çakır, Ö.K. and Ellek, N. and Salehin, N. and Hamamcı, R. and Keleş, H. and Kayalı, D.G. and Akakın, D. and Yüksel, M. and Özbeyli, D. | Excluded | The article was excluded based on title and abstract screening as it did not meet the inclusion and exclusion criteria. |
| Effects of heat and cold on health, with special reference to finnish sauna bathing | 2018 | Heinonen, I. and Laukkanen, J.A. | Excluded | The article was excluded based on title and abstract screening as it did not meet the inclusion and exclusion criteria. |
| Heme oxygenase-1 activity as a correlate to exercise-mediated amelioration of cognitive decline and neuropathological alterations in an aging rat model of dementia | 2018 | Kurucz, A. and Bombicz, M. and Kiss, R. and Priksz, D. and Varga, B. and Hortobágyi, T. and Trencsényi, G. and Szabó, R. and Pósa, A. and Gesztelyi, R. and Szilvássy, Z. and Juhász, B. | Excluded | The article was excluded based on title and abstract screening as it did not meet the inclusion and exclusion criteria. |
| Hepatoprotective effect of blocking N-methyl-D-aspartate receptors in male albino rats exposed to acute and repeated restraint stress | 2017 | Amin, S.N. and El-Aidi, A.A. and Zickri, M.B. and Rashed, L.A. and Hassan, S.S. | Excluded | The article was excluded based on title and abstract screening as it did not meet the inclusion and exclusion criteria. |
| An anti-inflammatory approach to the dietary management of multiple sclerosis: A condensed review | 2018 | Labuschagne, L. and Blaauw, R. | Excluded | The article was excluded based on title and abstract screening as it did not meet the inclusion and exclusion criteria. |
| Exercise as a protective mechanism against the negative effects of oxidative stress in first-episode psychosis: a biomarker-led study. | 2020 | Fisher E and Wood SJ and Elsworthy RJ and Upthegrove R and Aldred S | Included |  |
| The influence of vitamins E and C and exercise on brain aging | 2017 | Mock, J.T. and Chaudhari, K. and Sidhu, A. and Sumien, N. | Excluded | The article was excluded based on title and abstract screening as it did not meet the inclusion and exclusion criteria. |
| Irisin protects against neuronal injury induced by oxygen-glucose deprivation in part depends on the inhibition of ROS-NLRP3 inflammatory signaling pathway | 2017 | Peng, J. and Deng, X. and Huang, W. and Yu, J.-H. and Wang, J.-X. and Wang, J.-P. and Yang, S.-B. and Liu, X. and Wang, L. and Zhang, Y. and Zhou, X.-Y. and Yang, H. and He, Y.-Z. and Xu, F.-Y. | Excluded | The article was excluded based on title and abstract screening as it did not meet the inclusion and exclusion criteria. |
| Enhancement of memory function by antioxidant potential of Nigella sativa L. oil in restrained rats | 2017 | Sadaf, S. and Emad, S. and Siddiqui, N.A. and Ghafoor, S. and Yousuf, S. and Jabeen, B. and Haider, S. and Perveen, T. | Excluded | The article was excluded based on title and abstract screening as it did not meet the inclusion and exclusion criteria. |
| Protective effects of different exercise modalities in an Alzheimer's disease-like model | 2017 | Özbeyli, D. and Sarı, G. and Özkan, N. and Karademir, B. and Yüksel, M. and Çilingir Kaya, Ö.T. and Kasımay Çakır, Ö. | Excluded | The article was excluded based on title and abstract screening as it did not meet the inclusion and exclusion criteria. |
| Insulin resistance and mitochondrial dysfunction | 2017 | Gonzalez-Franquesa, A. and Patti, M.-E. | Excluded | The article was excluded based on title and abstract screening as it did not meet the inclusion and exclusion criteria. |
| Adult hippocampal neurogenesis: An important target associated with antidepressant effects of exercise | 2017 | Sun, L. and Sun, Q. and Qi, J. | Excluded | The article was excluded based on title and abstract screening as it did not meet the inclusion and exclusion criteria. |
| Exercise training alters kinetics of antioxidant enzymes in rat tissues | 1996 | Somani, S.M. and Husain, K. | Included |  |
| LC/QTOF profile and preliminary stability studies of an enriched flavonoid fraction of Cecropia pachystachya Trécul leaves with potential antidepressant-like activity | 2017 | Ortmann, C.F. and Abelaira, H.M. and Réus, G.Z. and Ignácio, Z.M. and Chaves, V.C. and dos Santos, T.C. and de Carvalho, P. and Carlessi, A.S. and Bruchchen, L. and Danielski, L.G. and Cardoso, S.G. and de Campos, A.M. and Petronilho, F. and Rebelo, J. and dos Santos Morais, M.O. and Vuolo, F. and Dal-Pizzol, F. and Streck, E.L. and Quevedo, J. and Reginatto, F.H. | Excluded | The article was excluded based on title and abstract screening as it did not meet the inclusion and exclusion criteria. |
| Long-term consumption of energy drinks induces biochemical and ultrastructural alterations in the heart muscle | 2018 | Munteanu, C. and Rosioru, C. and Tarba, C. and Lang, C. | Excluded | The article was excluded based on title and abstract screening as it did not meet the inclusion and exclusion criteria. |
| Nonhuman primate models of neurodegenerative disorders | 2017 | Emborg, M.E. | Excluded | The article was excluded based on title and abstract screening as it did not meet the inclusion and exclusion criteria. |
| Caffeine prevents high-intensity exercise-induced increase in enzymatic antioxidant and Na+-K+-ATPase activities and reduction of anxiolytic like-behaviour in rats | 2017 | Vieira, J.M. and Carvalho, F.B. and Gutierres, J.M. and Soares, M.S.P. and Oliveira, P.S. and Rubin, M.A. and Morsch, V.M. and Schetinger, M.R. and Spanevello, R.M. | Excluded | The article was excluded based on title and abstract screening as it did not meet the inclusion and exclusion criteria. |
| Exercise during adolescence attenuated depressive-like behaviors and hippocampal mitochondrial dysfunction following early life stress in adult male rats | 2018 | Masrour, F.F. and Peeri, M. and Hosseini, M.J. and Azarbayjani, M.A. | Excluded | The article was excluded based on title and abstract screening as it did not meet the inclusion and exclusion criteria. |
| Neuroprotective Effects and Mechanisms of Action of Multifunctional Agents Targeting Free Radicals, Monoamine Oxidase B and Cholinesterase in Parkinson’s Disease Model | 2017 | Liu, Z. and Cai, W. and Lang, M. and Yan, R. and Li, Z. and Zhang, G. and Yu, P. and Wang, Y. and Sun, Y. and Zhang, Z. | Excluded | The article was excluded based on title and abstract screening as it did not meet the inclusion and exclusion criteria. |
| Mechanisms underlying the impact of exercise training in pulmonary arterial hypertension | 2018 | Nogueira-Ferreira, R. and Moreira-Gonçalves, D. and Santos, M. and Trindade, F. and Ferreira, R. and Henriques-Coelho, T. | Excluded | The article was excluded based on title and abstract screening as it did not meet the inclusion and exclusion criteria. |
| Dietary fructose as a risk factor for non-alcoholic fatty liver disease (NAFLD) | 2017 | Alwahsh, S.M. and Gebhardt, R. | Excluded | The article was excluded based on title and abstract screening as it did not meet the inclusion and exclusion criteria. |
| Effect of physical exercise on brain and lipid metabolism in mouse models of multiple sclerosis | 2017 | Houdebine, L. and Gallelli, C.A. and Rastelli, M. and Sampathkumar, N.K. and Grenier, J. | Excluded | The article was excluded based on title and abstract screening as it did not meet the inclusion and exclusion criteria. |
| Health effects of carotenoids during pregnancy and lactation | 2017 | Zielińska, M.A. and Wesołowska, A. and Pawlus, B. and Hamułka, J. | Excluded | The article was excluded based on title and abstract screening as it did not meet the inclusion and exclusion criteria. |
| The Amyloid-β Oligomer Hypothesis: Beginning of the Third Decade | 2018 | Cline, E.N. and Bicca, M.A. and Viola, K.L. and Klein, W.L. | Excluded | The article was excluded based on title and abstract screening as it did not meet the inclusion and exclusion criteria. |
| Voluntary wheel running differentially affects disease outcomes in male and female mice with experimental autoimmune encephalomyelitis | 2017 | Mifflin, K.A. and Frieser, E. and Benson, C. and Baker, G. and Kerr, B.J. | Excluded | The article was excluded based on title and abstract screening as it did not meet the inclusion and exclusion criteria. |
| Dietary polyphenols and neurogenesis: Molecular interactions and implication for brain ageing and cognition | 2018 | Sarubbo, F. and Moranta, D. and Pani, G. | Excluded | The article was excluded based on title and abstract screening as it did not meet the inclusion and exclusion criteria. |
| Entrainment of the mouse circadian clock: Effects of stress, exercise, and nutrition | 2018 | Tahara, Y. and Shibata, S. | Excluded | The article was excluded based on title and abstract screening as it did not meet the inclusion and exclusion criteria. |
| Alcohol aggravates stress-induced cognitive deficits and hippocampal neurotoxicity: Protective effect of melatonin | 2017 | Rajput, P. and Jangra, A. and Kwatra, M. and Mishra, A. and Lahkar, M. | Excluded | The article was excluded based on title and abstract screening as it did not meet the inclusion and exclusion criteria. |
| High intensity interval training modulates hippocampal oxidative stress, BDNF and inflammatory mediators in rats | 2018 | Freitas, D.A. and Rocha-Vieira, E. and Soares, B.A. and Nonato, L.F. and Fonseca, S.R. and Martins, J.B. and Mendonça, V.A. and Lacerda, A.C. and Massensini, A.R. and Poortamns, J.R. and Meeusen, R. and Leite, H.R. | Excluded | The article was excluded based on title and abstract screening as it did not meet the inclusion and exclusion criteria. |
| Exercise training modulates the antioxidant enzymes in brain tissue: A study with reference to ageing | 2009 | Somasekar Reddy, N. and Shanmugam, K.R. and Mallikarjuna, K. and Sathyavelu Reddy, K. | Included |  |
| AMPK: Potential therapeutic target for ischemic stroke | 2018 | Jiang, S. and Li, T. and Ji, T. and Yi, W. and Yang, Z. and Wang, S. and Yang, Y. and Gu, C. | Excluded | The article was excluded based on title and abstract screening as it did not meet the inclusion and exclusion criteria. |
| Cellular microparticles and pathophysiology of traumatic brain injury | 2017 | Zhao, Z. and Zhou, Y. and Tian, Y. and Li, M. and Dong, J.-F. and Zhang, J. | Excluded | The article was excluded based on title and abstract screening as it did not meet the inclusion and exclusion criteria. |
| Ursolic acid: A versatile triterpenoid compound in regulating the aging | 2017 | Bakhtiari, N. and Moslemee-Jalalvand, E. and Kazemi, J. | Excluded | The article was excluded based on title and abstract screening as it did not meet the inclusion and exclusion criteria. |
| Neuroketotherapeutics: A modern review of a century-old therapy | 2018 | Koppel, S.J. and Swerdlow, R.H. | Excluded | The article was excluded based on title and abstract screening as it did not meet the inclusion and exclusion criteria. |
| Effects of prenatal exposure to WIFI signal (2.45 GHz) on postnatal development and behavior in rat: Influence of maternal restraint | 2017 | Othman, H. and Ammari, M. and Sakly, M. and Abdelmelek, H. | Excluded | The article was excluded based on title and abstract screening as it did not meet the inclusion and exclusion criteria. |
| New therapeutics to modulate mitochondrial function in neurodegenerative disorders | 2017 | Wilkins, H.M. and Morris, J.K. | Excluded | The article was excluded based on title and abstract screening as it did not meet the inclusion and exclusion criteria. |
| Effects of exercise on learning and memory, oxidative stress and nNOS expression in marginal division of striatum of ovariectomized rats | 2018 | Cui, J. and Guo, Y. and Yang, W. and Zhao, X. and Yu, F. and Tang, W. and Pang, B. and Su, X. | Excluded | The article was excluded based on title and abstract screening as it did not meet the inclusion and exclusion criteria. |
| Attenuation of acute restraint stress-induced depressive like behavior and hippocampal alterations with protocatechuic acid treatment in mice | 2017 | Thakare, V.N. and Dhakane, V.D. and Patel, B.M. | Excluded | The article was excluded based on title and abstract screening as it did not meet the inclusion and exclusion criteria. |
| Status of the brain antioxidant system at different growing periods after prenatal stress and N-Acetyl cysteine administration | 2017 | Bernhardt, L.K. and Madhyastha, S. and Bairy, L. and Kishore, A. | Excluded | The article was excluded based on title and abstract screening as it did not meet the inclusion and exclusion criteria. |
| Inflammation: a highly conserved, Janus-like phenomenon—a gastroenterologist’ perspective | 2018 | Ribaldone, D.G. and Pellicano, R. and Actis, G.C. | Excluded | The article was excluded based on title and abstract screening as it did not meet the inclusion and exclusion criteria. |
| Exercise as a Positive Modulator of Brain Function | 2018 | Alkadhi, K.A. | Excluded | The article was excluded based on title and abstract screening as it did not meet the inclusion and exclusion criteria. |
| Multiple Mechanisms Linking Type 2 Diabetes and Alzheimer's Disease: Testosterone as a Modifier | 2017 | Asih, P.R. and Tegg, M.L. and Sohrabi, H. and Carruthers, M. and Gandy, S.E. and Saad, F. and Verdile, G. and Ittner, L.M. and Martins, R.N. | Excluded | The article was excluded based on title and abstract screening as it did not meet the inclusion and exclusion criteria. |
| Physical exercise improves total antioxidant capacity and gene expression in rat hippocampal tissue | 2017 | Franzoni, F. and Federighi, G. and Fusi, J. and Agosta, V. and Cerri, E. and Banducci, R. and Petrocchi, A. and Bernardi, R. and Innocenti, A. and Pruneti, C. and Daniele, S. and Pellegrini, S. and Martini, C. and Scuri, R. and Galetta, F. | Excluded | The article was excluded based on title and abstract screening as it did not meet the inclusion and exclusion criteria. |
| Effects of repeated restraint stress and WiFi signal exposure on behavior and oxidative stress in rats | 2017 | Othman, H. and Ammari, M. and Sakly, M. and Abdelmelek, H. | Excluded | The article was excluded based on title and abstract screening as it did not meet the inclusion and exclusion criteria. |
| Myokine irisin-induced protection against oxidative stress in vitro. Involvement of heme oxygenase-1 and antioxidazing enzymes superoxide dismutase-2 and glutathione peroxidase | 2018 | Mazur-Bialy, A.I. and Kozlowska, K. and Pochec, E. and Bilski, J. and Brzozowski, T. | Excluded | The article was excluded based on title and abstract screening as it did not meet the inclusion and exclusion criteria. |
| Fatty acids, antioxidants and physical activity in brain aging | 2017 | Freitas, H.R. and Ferreira, G.C. and Trevenzoli, I.H. and Oliveira, K.J. and Reis, R.A.M. | Excluded | The article was excluded based on title and abstract screening as it did not meet the inclusion and exclusion criteria. |
| Repeated restraint stress increases seizure susceptibility by activation of hippocampal endoplasmic reticulum stress | 2017 | Zhu, X. and Dong, J. and Xia, Z. and Zhang, A. and Chao, J. and Yao, H. | Excluded | The article was excluded based on title and abstract screening as it did not meet the inclusion and exclusion criteria. |
| Oxidative Stress, Synaptic Dysfunction, and Alzheimer's Disease | 2017 | Tönnies, E. and Trushina, E. | Excluded | The article was excluded based on title and abstract screening as it did not meet the inclusion and exclusion criteria. |
| Physical Exercise Attenuates Experimental Autoimmune Encephalomyelitis by Inhibiting Peripheral Immune Response and Blood-Brain Barrier Disruption | 2017 | Souza, P.S. and Gonçalves, E.D. and Pedroso, G.S. and Farias, H.R. and Junqueira, S.C. and Marcon, R. and Tuon, T. and Cola, M. and Silveira, P.C.L. and Santos, A.R. and Calixto, J.B. and Souza, C.T. and de Pinho, R.A. and Dutra, R.C. | Excluded | The article was excluded based on title and abstract screening as it did not meet the inclusion and exclusion criteria. |
| Proteomic analysis of hippocampus in a mouse model of depression reveals neuroprotective function of ubiquitin C-terminal hydrolase l1 (UCH-L1) via stress-induced cysteine oxidative modifications | 2018 | Choi, J.-E. and Lee, J.-J. and Kang, W. and Jung Kim, H. and Cho, J.-H. and Han, P.-L. and Lee, K.-J. | Excluded | The article was excluded based on title and abstract screening as it did not meet the inclusion and exclusion criteria. |
| Lithium ameliorates sleep deprivation-induced mania-like behavior, hypothalamic-pituitary-adrenal (HPA) axis alterations, oxidative stress and elevations of cytokine concentrations in the brain and serum of mice | 2017 | Valvassori, S.S. and Resende, W.R. and Dal-Pont, G. and Sangaletti-Pereira, H. and Gava, F.F. and Peterle, B.R. and Carvalho, A.F. and Varela, R.B. and Dal-Pizzol, F. and Quevedo, J. | Excluded | The article was excluded based on title and abstract screening as it did not meet the inclusion and exclusion criteria. |
| Alzheimer's Disease: A Journey from Amyloid Peptides and Oxidative Stress, to Biomarker Technologies and Disease Prevention Strategies-Gains from AIBL and DIAN Cohort Studies | 2018 | Martins, R.N. and Villemagne, V. and Sohrabi, H.R. and Chatterjee, P. and Shah, T.M. and Verdile, G. and Fraser, P. and Taddei, K. and Gupta, V.B. and Rainey-Smith, S.R. and Hone, E. and Pedrini, S. and Lim, W.L. and Martins, I. and Frost, S. and Gupta, S. and O'Bryant, S. and Rembach, A. and Ames, D. and Ellis, K. and Fuller, S.J. and Brown, B. and Gardener, S.L. and Fernando, B. and Bharadwaj, P. and Burnham, S. and Laws, S.M. and Barron, A.M. and Goozee, K. and Wahjoepramono, E.J. and Asih, P.R. and Doecke, J.D. and Salvado, O. and Bush, A.I. and Rowe, C.C. and Gandy, S.E. and Masters, C.L. | Excluded | The article was excluded based on title and abstract screening as it did not meet the inclusion and exclusion criteria. |
| The neurobiological mechanisms of physical exercise in methamphetamine addiction | 2018 | Morais, A.P.D. and Pita, I.R. and Fontes-Ribeiro, C.A. and Pereira, F.C. | Excluded | The article was excluded based on title and abstract screening as it did not meet the inclusion and exclusion criteria. |
| Antifatigue Effects of Antrodia cinnamomea Cultured Mycelium via Modulation of Oxidative Stress Signaling in a Mouse Model | 2017 | Liu, Y. and Li, L. and An, S. and Zhang, Y. and Feng, S. and Zhao, L. and Teng, L. and Wang, D. | Excluded | The article was excluded based on title and abstract screening as it did not meet the inclusion and exclusion criteria. |
| Angiotensin II, oxidative stress, and sympathetic nervous system hyperactivity in heart failure | 2018 | Koba, S. | Excluded | The article was excluded based on title and abstract screening as it did not meet the inclusion and exclusion criteria. |
| Defective insulin signalling, mediated by inflammation, connects obesity to Alzheimer disease; relevant pharmacological therapies and preventive dietary interventions | 2017 | Rodriguez-Casado, A. and Toledano-Díaz, A. and Toledano, A. | Excluded | The article was excluded based on title and abstract screening as it did not meet the inclusion and exclusion criteria. |
| Effect of heat acclimation on mitochondrion of cerebral cortex and myocardium in rats stimulated by exercise in hygrothermal environment | 2017 | Liang, H. and Li, Y.-X. and Chen, X.-M. and Ren, H. and Chang, W.-J. and Shi, R.-F. and Ma, W.-L. | Excluded | The article was excluded based on title and abstract screening as it did not meet the inclusion and exclusion criteria. |
| Exercise training improves cardiac autonomic control, cardiac function, and arrhythmogenesis in rats with preserved-ejection fraction heart failure | 2017 | Andrade, D.C. and Arce-Alvarez, A. and Toledo, C. and Díaz, H.S. and Lucero, C. and Schultz, H.D. and Marcus, N.J. and Rio, R.D. | Excluded | The article was excluded based on title and abstract screening as it did not meet the inclusion and exclusion criteria. |
| Restraint stress exacerbates cardiac and adipose tissue pathology via β-adrenergic signaling in rats with metabolic syndrome | 2015 | Matsuura, N. and Nagasawa, K. and Minagawa, Y. and Ito, S. and Sano, Y. and Yamada, Y. and Hattori, T. and Watanabe, S. and Murohara, T. and Nagata, K. | Excluded | The article was excluded based on title and abstract screening as it did not meet the inclusion and exclusion criteria. |
| High-intensity Exercise Modifies the Effects of Stanozolol on Brain Oxidative Stress in Rats | 2015 | Camiletti-Moirón, D. and Aparicio, V.A. and Nebot, E. and Medina, G. and Martínez, R. and Kapravelou, G. and Andrade, A. and Porres, J.M. and López-Jurado, M. and Aranda, P. | Excluded | The article was excluded based on title and abstract screening as it did not meet the inclusion and exclusion criteria. |
| Neurohormetic phytochemicals: An evolutionary-bioenergetic perspective | 2015 | Murugaiyah, V. and Mattson, M.P. | Excluded | The article was excluded based on title and abstract screening as it did not meet the inclusion and exclusion criteria. |
| Gender differences in nitric oxide and antioxidant response to physical stress in tissues of trained mice | 2019 | Kormanovski, A. and Castillo-Hernández, M.C. and Guevara-Balcázar, G. and Pérez, T. and Lara-Padilla, E. | Included |  |
| Magnetic resonance imaging biomarkers of exercise-induced improvement of oxidative stress and inflammation in the brain of old high-fat-fed ApoE−/− mice | 2016 | Chirico, E.N. and Di Cataldo, V. and Chauveau, F. and Geloën, A. and Patsouris, D. and Thézé, B. and Martin, C. and Vidal, H. and Rieusset, J. and Pialoux, V. and Canet-Soulas, E. | Excluded | The article was excluded based on title and abstract screening as it did not meet the inclusion and exclusion criteria. |
| Pharmacological and biochemical studies on the protective effects of melatonin during stress-induced behavioral and immunological changes in relation to oxidative stress in rats | 2015 | Pal, R. and Gulati, K. and Banerjee, B.D. and Ray, A. | Excluded | The article was excluded based on title and abstract screening as it did not meet the inclusion and exclusion criteria. |
| Protective effects of physical exercise on MDMA-induced cognitive and mitochondrial impairment | 2016 | Taghizadeh, G. and Pourahmad, J. and Mehdizadeh, H. and Foroumadi, A. and Torkaman-Boutorabi, A. and Hassani, S. and Naserzadeh, P. and Shariatmadari, R. and Gholami, M. and Rouini, M.R. and Sharifzadeh, M. | Excluded | The article was excluded based on title and abstract screening as it did not meet the inclusion and exclusion criteria. |
| Glutathione status and reactive oxygen generation in tissues of young and old exercised rats | 1997 | Ohkuwa, T. and Sato, Y. and Naoi, M. | Included |  |
| The mitochondrial uncoupler DNP triggers brain cell mTOR signaling network reprogramming and CREB pathway up-regulation | 2015 | Liu, D. and Zhang, Y. and Gharavi, R. and Park, H.R. and Lee, J. and Siddiqui, S. and Telljohann, R. and Nassar, M.R. and Cutler, R.G. and Becker, K.G. and Mattson, M.P. | Excluded | The article was excluded based on title and abstract screening as it did not meet the inclusion and exclusion criteria. |
| Are there deleterious cardiac effects of acute and chronic endurance exercise? | 2016 | Eijsvogels, T.M.H. and Fernandez, A.B. and Thompson, P.D. | Excluded | The article was excluded based on title and abstract screening as it did not meet the inclusion and exclusion criteria. |
| Amyloid β oligomers in Alzheimer’s disease pathogenesis, treatment, and diagnosis | 2015 | Viola, K.L. and Klein, W.L. | Excluded | The article was excluded based on title and abstract screening as it did not meet the inclusion and exclusion criteria. |
| Comparative Analysis of Antioxidant Properties of Comenic Acid and Potassium Comenate in Modeled Immobilization Stress | 2015 | Shurygina, L.V. and Zlishcheva, E.I. and Khablyuk, V.V. and Kravtsova, A.N. and Abramova, N.O. and Zlishcheva, L.I. and Kravtsov, A.A. | Excluded | The article was excluded based on title and abstract screening as it did not meet the inclusion and exclusion criteria. |
| Regular Exercise Combined With Curcumin Supplementation: Protective Effects against Lead-Induced Cerebellar Oxidative Damage in an Animal Model | 2016 | Habibian, M. and Moosavi, S.J. and Farzanegi, P. | Excluded | The article was excluded based on title and abstract screening as it did not meet the inclusion and exclusion criteria. |
| Health span-extending activity of human amniotic membrane-and adipose tissue-derived stem cells in F344 rats | 2015 | Kim, D. and Kyung, J. and Park, D. and Choi, E.-K. and Kim, K.S. and Shin, K. and Lee, H. and Shin, I.S. and Kang, S.K. and Ra, J.C. and Kim, Y.-B. | Excluded | The article was excluded based on title and abstract screening as it did not meet the inclusion and exclusion criteria. |
| Synergistic effects of diet and exercise on hippocampal function in chronically stressed mice | 2015 | Hutton, C.P. and Déry, N. and Rosa, E. and Lemon, J.A. and Rollo, C.D. and Boreham, D.R. and Fahnestock, M. and deCatanzaro, D. and Wojtowicz, J.M. and Becker, S. | Excluded | The article was excluded based on title and abstract screening as it did not meet the inclusion and exclusion criteria. |
| Potential antidepressant-like activity of silymarin in the acute restraint stress in mice: Modulation of corticosterone and oxidative stress response in cerebral cortex and hippocampus | 2016 | Thakare, V.N. and Dhakane, V.D. and Patel, B.M. | Excluded | The article was excluded based on title and abstract screening as it did not meet the inclusion and exclusion criteria. |
| High-Intensity Exercise Training Protects the Brain Against Autoimmune Neuroinflammation: Regulation of Microglial Redox and Pro-inflammatory Functions | 2021 | Zaychik, Y. and Fainstein, N. and Touloumi, O. and Goldberg, Y. and Hamdi, L. and Segal, S. and Nabat, H. and Zoidou, S. and Grigoriadis, N. and Katz, A. and Ben-Hur, T. and Einstein, O. | Included |  |
| Pivotal role of α2 Na+ pumps and their high affinity ouabain binding site in cardiovascular health and disease | 2016 | Blaustein, M.P. and Chen, L. and Hamlyn, J.M. and Leenen, F.H.H. and Lingrel, J.B. and Wier, W.G. and Zhang, J. | Excluded | The article was excluded based on title and abstract screening as it did not meet the inclusion and exclusion criteria. |
| Pretreatment with curcumin attenuates anxiety while strengthens memory performance after one short stress experience in male rats | 2015 | Haider, S. and Naqvi, F. and Batool, Z. and Tabassum, S. and Sadir, S. and Liaquat, L. and Naqvi, F. and Zuberi, N.A. and Shakeel, H. and Perveen, T. | Excluded | The article was excluded based on title and abstract screening as it did not meet the inclusion and exclusion criteria. |
| Maternal hydroxytyrosol administration improves neurogenesis and cognitive function in prenatally stressed offspring | 2015 | Zheng, A. and Li, H. and Cao, K. and Xu, J. and Zou, X. and Li, Y. and Chen, C. and Liu, J. and Feng, Z. | Excluded | The article was excluded based on title and abstract screening as it did not meet the inclusion and exclusion criteria. |
| Antistress Effects of Rosa rugosa Thunb. on Total Sleep Deprivation-Induced Anxiety-Like Behavior and Cognitive Dysfunction in Rat: Possible Mechanism of Action of 5-HT 6 Receptor Antagonist | 2016 | Na, J.-R. and Oh, D.-R. and Han, S. and Kim, Y.-J. and Choi, E. and Bae, D. and Oh, D.H. and Lee, Y.-H. and Kim, S. and Jun, W. | Excluded | The article was excluded based on title and abstract screening as it did not meet the inclusion and exclusion criteria. |
| Combined Low-Intensity Exercise and Ascorbic Acid Attenuates Kainic Acid-Induced Seizure and Oxidative Stress in Mice | 2016 | Kim, H.-J. and Song, W. and Jin, E.H. and Kim, J. and Chun, Y. and An, E.N. and Park, S. | Excluded | The article was excluded based on title and abstract screening as it did not meet the inclusion and exclusion criteria. |
| Diastolic dysfunction: Potential new diagnostics and therapies | 2015 | Jeong, E.-M. and Dudley, S.C. | Excluded | The article was excluded based on title and abstract screening as it did not meet the inclusion and exclusion criteria. |
| Treadmill exercise activates Nrf2 antioxidant system to protect the nigrostriatal dopaminergic neurons from MPP+ toxicity | 2015 | Tsou, Y.-H. and Shih, C.-T. and Ching, C.-H. and Huang, J.-Y. and Jen, C.J. and Yu, L. and Kuo, Y.-M. and Wu, F.-S. and Chuang, J.-I. | Excluded | The article was excluded based on title and abstract screening as it did not meet the inclusion and exclusion criteria. |
| Lipid peroxidation: Inhibition, effects and mechanisms | 2016 | Catalá, A. | Excluded | The article was excluded based on title and abstract screening as it did not meet the inclusion and exclusion criteria. |
| Differential expression of hypothalamic, metabolic and inflammatory genes in response to short-term calorie restriction in juvenile obese- and lean-prone JCR rats | 2015 | Diane, A. and Pierce, W.D. and Mangat, R. and Borthwick, F. and Nelson, R. and Russell, J.C. and Heth, C.D. and Jacobs, R.L. and Vine, D.F. and Proctor, S.D. | Excluded | The article was excluded based on title and abstract screening as it did not meet the inclusion and exclusion criteria. |
| Age-dependent effects of esculetin on mood-related behavior and cognition from stressed mice are associated with restoring brain antioxidant status | 2016 | Martín-Aragón, S. and Villar, Á. and Benedí, J. | Excluded | The article was excluded based on title and abstract screening as it did not meet the inclusion and exclusion criteria. |
| Testosterone depletion increases the susceptibility of brain tissue to oxidative damage in a restraint stress mouse model | 2016 | Son, S.-W. and Lee, J.-S. and Kim, H.-G. and Kim, D.-W. and Ahn, Y.-C. and Son, C.-G. | Excluded | The article was excluded based on title and abstract screening as it did not meet the inclusion and exclusion criteria. |
| Organ and tissue-dependent effect of resveratrol and exercise on antioxidant defenses of old mice | 2015 | Tung, B.T. and Rodriguez-Bies, E. and Thanh, H.N. and Le-Thi-Thu, H. and Navas, P. and Sanchez, V.M. and López-Lluch, G. | Excluded | The article was excluded based on title and abstract screening as it did not meet the inclusion and exclusion criteria. |
| Physical exercise prevents short and long-term deficits on aversive and recognition memory and attenuates brain oxidative damage induced by maternal deprivation | 2015 | Neves, B.-H. and Menezes, J. and Souza, M.A. and Mello-Carpes, P.B. | Excluded | The article was excluded based on title and abstract screening as it did not meet the inclusion and exclusion criteria. |
| Effect of glycation inhibitors on aging and age-related diseases | 2016 | Sadowska-Bartosz, I. and Bartosz, G. | Excluded | The article was excluded based on title and abstract screening as it did not meet the inclusion and exclusion criteria. |
| Preimplantation maternal stress impairs embryo development by inducing oviductal apoptosis with activation of the Fas system | 2016 | Zheng, L.-L. and Tan, X.-W. and Cui, X.-Z. and Yuan, H.-J. and Li, H. and Jiao, G.-Z. and Ji, C.-L. and Tan, J.-H. | Excluded | The article was excluded based on title and abstract screening as it did not meet the inclusion and exclusion criteria. |
| An “enigmatic” l-carnosine (β-alanyl-l-histidine)? cell proliferative activity as a fundamental property of a natural dipeptide inherent to traditional antioxidant, anti-aging biological activities: Balancing and a hormonally correct agent, novel patented oral therapy dosage formulation for mobil... | 2015 | Babizhayev, M.A. and Yegorov, Y.E. | Excluded | The article was excluded based on title and abstract screening as it did not meet the inclusion and exclusion criteria. |
| Slowing down glioblastoma progression in mice by running or the anti-malarial drug dihydroartemisinin? Induction of oxidative stress in murine glioblastoma therapy | 2016 | Lemke, D. and Pledl, H.-W. and Zorn, M. and Jugold, M. and Green, E. and Blaes, J. and Löw, S. and Hertenstein, A. and Ott, M. and Sahm, F. and Steffen, A.-C. and Weiler, M. and Winkler, F. and Platten, M. and Dong, Z. and Wick, W. | Excluded | The article was excluded based on title and abstract screening as it did not meet the inclusion and exclusion criteria. |
| High-protein diet induces oxidative stress in rat brain: Protective action of high-intensity exercise against lipid peroxidation | 2015 | Camiletti-Móiron, D. and Arianna Aparicio, V. and Nebot, E. and Medina, G. and Martínez, R. and Kapravelou, G. and Andrade, A. and Porres, J.M. and López-Jurado, M. and Aranda, P. | Excluded | The article was excluded based on title and abstract screening as it did not meet the inclusion and exclusion criteria. |
| Acute exercise increases hippocampal TNF-α, Caspase-3 and Caspase-7 expression in healthy young and older mice | 2015 | Packer, N. and Hoffman-Goetz, L. | Excluded | The article was excluded based on title and abstract screening as it did not meet the inclusion and exclusion criteria. |
| Modulation of angiotensin II signaling following exercise training in heart failure | 2015 | Zucker, I.H. and Schultz, H.D. and Patel, K.P. and Wang, H. | Excluded | The article was excluded based on title and abstract screening as it did not meet the inclusion and exclusion criteria. |
| Why is obesity such a problem in the 21st century? The intersection of palatable food, cues and reward pathways, stress, and cognition | 2015 | Morris, M.J. and Beilharz, J.E. and Maniam, J. and Reichelt, A.C. and Westbrook, R.F. | Excluded | The article was excluded based on title and abstract screening as it did not meet the inclusion and exclusion criteria. |
| Molecular hydrogen in sports medicine: New therapeutic perspectives | 2015 | Ostojic, S.M. | Excluded | The article was excluded based on title and abstract screening as it did not meet the inclusion and exclusion criteria. |
| Neurobiological effects of exercise on major depressive disorder: A systematic review | 2016 | Schuch, F.B. and Deslandes, A.C. and Stubbs, B. and Gosmann, N.P. and Silva, C.T.B.D. and Fleck, M.P.D.A. | Excluded | The article was excluded based on title and abstract screening as it did not meet the inclusion and exclusion criteria. |
| Effects of Acorus calamus rhizome extract on the neuromodulatory system in restraint stress male rats | 2015 | Reddy, S. and Rao, G. and Shetty, B. and Hn, G. | Excluded | The article was excluded based on title and abstract screening as it did not meet the inclusion and exclusion criteria. |
| Effect of vitamin E on cerebral cortical oxidative stress and brain-derived neurotrophic factor gene expression induced by hypoxia and exercise in rats | 2015 | Sakr, H.F. and Abbas, A.M. and El Samanoudy, A.Z. | Excluded | The article was excluded based on title and abstract screening as it did not meet the inclusion and exclusion criteria. |
| Linkage of stress with neuromuscular disorders | 2016 | Ashraf, G.M. and Ali, A. and Tabrez, S. and Zaidi, S.K. and Shakil, S. and Alam, M.Z. and Rehan, M. and Aliev, G. | Excluded | The article was excluded based on title and abstract screening as it did not meet the inclusion and exclusion criteria. |
| The shared neuroanatomy and neurobiology of comorbid chronic pain and PTSD: Therapeutic implications | 2015 | Scioli-Salter, E.R. and Forman, D.E. and Otis, J.D. and Gregor, K. and Valovski, I. and Rasmusson, A.M. | Excluded | The article was excluded based on title and abstract screening as it did not meet the inclusion and exclusion criteria. |
| Combination therapies for neurobehavioral and cognitive recovery after experimental traumatic brain injury: Is more better? | 2016 | Kline, A.E. and Leary, J.B. and Radabaugh, H.L. and Cheng, J.P. and Bondi, C.O. | Excluded | The article was excluded based on title and abstract screening as it did not meet the inclusion and exclusion criteria. |
| Hormone-brain-aging relationships, broadly reactive with imidazole-containing dipeptides: Targeting of telomere attrition as an aging biomarker and dynamic telomerase activity flirting | 2015 | Babizhayev, M.A. and Vishnyakova, K.S. and Yegorov, Y.E. | Excluded | The article was excluded based on title and abstract screening as it did not meet the inclusion and exclusion criteria. |
| White matter injury in ischemic stroke | 2016 | Wang, Y. and Liu, G. and Hong, D. and Chen, F. and Ji, X. and Cao, G. | Excluded | The article was excluded based on title and abstract screening as it did not meet the inclusion and exclusion criteria. |
| Neural mechanisms of exercise: Effects on gut miccrobiota and depression | 2015 | Yuan, T.-F. and Rocha, N.B.F. and Paes, F. and Arias-Carrión, O. and Machado, S. and De Sá Filho, A.S. | Excluded | The article was excluded based on title and abstract screening as it did not meet the inclusion and exclusion criteria. |
| Does PGC1α/FNDC5/BDNF Elicit the Beneficial Effects of Exercise on Neurodegenerative Disorders? | 2016 | Jodeiri Farshbaf, M. and Ghaedi, K. and Megraw, T.L. and Curtiss, J. and Shirani Faradonbeh, M. and Vaziri, P. and Nasr-Esfahani, M.H. | Excluded | The article was excluded based on title and abstract screening as it did not meet the inclusion and exclusion criteria. |
| Carbon monoxide may be an important molecule In migraine and other headaches | 2014 | Arngrim, N. and Schytz, H.W. and Hauge, M.K. and Ashina, M. and Olesen, J. | Excluded | The article was excluded based on title and abstract screening as it did not meet the inclusion and exclusion criteria. |
| Featured Article: Accelerated decline of physical strength in peroxiredoxin-3 knockout mice | 2016 | Zhang, Y.-G. and Wang, L. and Kaifu, T. and Li, J. and Li, X. and Li, L. | Excluded | The article was excluded based on title and abstract screening as it did not meet the inclusion and exclusion criteria. |
| Late running is not too late against Alzheimer's pathology | 2016 | Herring, A. and Münster, Y. and Metzdorf, J. and Bolczek, B. and Krüssel, S. and Krieter, D. and Yavuz, I. and Karim, F. and Roggendorf, C. and Stang, A. and Wang, Y. and Hermann, D.M. and Teuber-Hanselmann, S. and Keyvani, K. | Excluded | The article was excluded based on title and abstract screening as it did not meet the inclusion and exclusion criteria. |
| Saffron (its active constituent, crocin) supplementation attenuates lipid peroxidation and protects against tissue injury | 2016 | Altinoz, E. and Ozmen, T. and Oner, Z. and Elbe, H. and Erdemli, M.E. and Bag, H.G. | Excluded | The article was excluded based on title and abstract screening as it did not meet the inclusion and exclusion criteria. |
| New insights into the trophic and cytoprotective effects of creatine in in vitro and in vivo models of cell maturation | 2016 | Sestili, P. and Ambrogini, P. and Barbieri, E. and Sartini, S. and Fimognari, C. and Calcabrini, C. and Diaz, A.R. and Guescini, M. and Polidori, E. and Luchetti, F. and Canonico, B. and Lattanzi, D. and Cuppini, R. and Papa, S. and Stocchi, V. | Excluded | The article was excluded based on title and abstract screening as it did not meet the inclusion and exclusion criteria. |
| Neuroprotective mechanism of losartan and its interaction with nimesulide against chronic fatigue stress | 2015 | Kumar, A. and Singh, B. and Mishra, J. and Sah, S.P. and Pottabathini, R. | Excluded | The article was excluded based on title and abstract screening as it did not meet the inclusion and exclusion criteria. |
| Type 2 deiodinase disruption in astrocytes results in anxiety-depressive-like behavior in male mice | 2016 | Bocco, B.M.L.C. and Werneck-De-castro, J.P. and Oliveira, K.C. and Fernandes, G.W. and Fonseca, T.L. and Nascimento, B.P.P. and Mcaninch, E.A. and Ricci, E. and Kvárta-Papp, Z. and Fekete, C. and Bernardi, M.M. and Gereben, B. and Bianco, A.C. and Ribeiro, M.O. | Excluded | The article was excluded based on title and abstract screening as it did not meet the inclusion and exclusion criteria. |
| Down syndrome and Alzheimer's disease: Common pathways, common goals | 2015 | Hartley, D. and Blumenthal, T. and Carrillo, M. and DiPaolo, G. and Esralew, L. and Gardiner, K. and Granholm, A.-C. and Iqbal, K. and Krams, M. and Lemere, C. and Lott, I. and Mobley, W. and Ness, S. and Nixon, R. and Potter, H. and Reeves, R. and Sabbagh, M. and Silverman, W. and Tycko, B. and Whitten, M. and Wisniewski, T. | Excluded | The article was excluded based on title and abstract screening as it did not meet the inclusion and exclusion criteria. |
| Environmental factors as modulators of neurodegeneration: Insights from gene-environment interactions in Huntington's disease | 2015 | Mo, C. and Hannan, A.J. and Renoir, T. | Excluded | The article was excluded based on title and abstract screening as it did not meet the inclusion and exclusion criteria. |
| Dysfunctional endothelial progenitor cells in cardiovascular diseases: Role of NADPH oxidase | 2015 | Peng, J. and Liu, B. and Ma, Q.-L. and Luo, X.-J. | Excluded | The article was excluded based on title and abstract screening as it did not meet the inclusion and exclusion criteria. |
| Neural mechanisms of exercise: Anti-depression, neurogenesis, and serotonin signaling | 2015 | Yuan, T.-F. and Paes, F. and Arias-Carrión, O. and Rocha, N.B.F. and De Sá Filho, A.S. and Machado, S. | Excluded | The article was excluded based on title and abstract screening as it did not meet the inclusion and exclusion criteria. |
| Exercise as Gene Therapy: BDNF and DNA Damage Repair | 2016 | Schmidt, R.H. and Nickerson, J.M. and Boatright, J.H. | Excluded | The article was excluded based on title and abstract screening as it did not meet the inclusion and exclusion criteria. |
| The incremental induction of neuroprotective properties by multiple therapeutic strategies for primary and secondary neural injury | 2015 | Lee, S. and Park, S. and Won, J. and Lee, S.-R. and Chang, K.-T. and Hong, Y. | Excluded | The article was excluded based on title and abstract screening as it did not meet the inclusion and exclusion criteria. |
| (-)Epigallocatechin-3-gallate decreases the stress-induced impairment of learning and memory in rats | 2015 | Soung, H.-S. and Wang, M.-H. and Tseng, H.-C. and Fang, H.-W. and Chang, K.-C. | Excluded | The article was excluded based on title and abstract screening as it did not meet the inclusion and exclusion criteria. |
| Effects of pre-natal alcohol exposure on hippocampal synaptic plasticity: Sex, age and methodological considerations | 2016 | Fontaine, C.J. and Patten, A.R. and Sickmann, H.M. and Helfer, J.L. and Christie, B.R. | Excluded | The article was excluded based on title and abstract screening as it did not meet the inclusion and exclusion criteria. |
| Sodium Phenylbutyrate and Edaravone Abrogate Chronic Restraint Stress-Induced Behavioral Deficits: Implication of Oxido-Nitrosative, Endoplasmic Reticulum Stress Cascade, and Neuroinflammation | 2017 | Jangra, A. and Sriram, C.S. and Dwivedi, S. and Gurjar, S.S. and Hussain, M.I. and Borah, P. and Lahkar, M. | Excluded | The article was excluded based on title and abstract screening as it did not meet the inclusion and exclusion criteria. |
| 3-Hydroxybutyrate regulates energy metabolism and induces BDNF expression in cerebral cortical neurons | 2016 | Marosi, K. and Kim, S.W. and Moehl, K. and Scheibye-Knudsen, M. and Cheng, A. and Cutler, R. and Camandola, S. and Mattson, M.P. | Excluded | The article was excluded based on title and abstract screening as it did not meet the inclusion and exclusion criteria. |
| Age-related changes in the brain antioxidant status: modulation by dietary supplementation of Decalepis hamiltonii and physical exercise | 2016 | Ravikiran, T. and Sowbhagya, R. and Anupama, S.K. and Anand, S. and Bhagyalakshmi, D. | Excluded | The article was excluded based on title and abstract screening as it did not meet the inclusion and exclusion criteria. |
| The Impact of Previous Physical Training on Redox Signaling after Traumatic Brain Injury in Rats: A Behavioral and Neurochemical Approach | 2016 | Fiorin, F.D.S. and Ferreira, A.P.D.O. and Ribeiro, L.R. and Silva, L.F.A. and De Castro, M.R.T. and Da Silva, L.R.H. and Da Silveira, M.E.P. and Zemolin, A.P.P. and Dobrachinski, F. and De Oliveira, S.M. and Franco, J.L. and Soares, F.A. and Furian, A.F. and Oliveira, M.S. and Fighera, M.R. and Royes, L.F.F. | Excluded | The article was excluded based on title and abstract screening as it did not meet the inclusion and exclusion criteria. |
| Cardioprotective effects of early and late aerobic exercise training in experimental pulmonary arterial hypertension | 2015 | Moreira-Gonçalves, D. and Ferreira, R. and Fonseca, H. and Padrão, A.I. and Moreno, N. and Silva, A.F. and Vasques-Nóvoa, F. and Gonçalves, N. and Vieira, S. and Santos, M. and Amado, F. and Duarte, J.A. and Leite-Moreira, A.F. and Henriques-Coelho, T. | Excluded | The article was excluded based on title and abstract screening as it did not meet the inclusion and exclusion criteria. |
| The role of skeletal muscle in amyotrophic lateral sclerosis | 2016 | Loeffler, J.-P. and Picchiarelli, G. and Dupuis, L. and Gonzalez De Aguilar, J.-L. | Excluded | The article was excluded based on title and abstract screening as it did not meet the inclusion and exclusion criteria. |
| Oxidative stress and cancer; the role of hesperidin, a citrus natural bioflavonoid, as a cancer chemoprotective agent | 2016 | Ahmadi, A. and Shadboorestan, A. | Excluded | The article was excluded based on title and abstract screening as it did not meet the inclusion and exclusion criteria. |
| Evaluation of some biochemical parameters and brain oxidative stress in experimental rats exposed chronically to silver nitrate and the protective role of vitamin E and selenium | 2016 | Gueroui, M. and Kechrid, Z. | Excluded | The article was excluded based on title and abstract screening as it did not meet the inclusion and exclusion criteria. |
| The neuroprotective effects of preconditioning exercise on brain damage and neurotrophic factors after focal brain ischemia in rats | 2016 | Otsuka, S. and Sakakima, H. and Sumizono, M. and Takada, S. and Terashi, T. and Yoshida, Y. | Excluded | The article was excluded based on title and abstract screening as it did not meet the inclusion and exclusion criteria. |
| The effect of diabetes mellitus on apoptosis in hippocampus: Cellular and molecular aspects | 2016 | Sadeghi, A. and Hami, J. and Razavi, S. and Esfandiary, E. and Hejazi, Z. | Excluded | The article was excluded based on title and abstract screening as it did not meet the inclusion and exclusion criteria. |
| Exercise does not protect against peripheral and central effects of a high cholesterol diet given ad libitum in old ApoE-/- mice | 2016 | Di Cataldo, V. and Géloën, A. and Langlois, J.-B. and Chauveau, F. and Thézé, B. and Hubert, V. and Wiart, M. and Chirico, E.N. and Rieusset, J. and Vidal, H. and Pialoux, V. and Canet-Soulas, E. | Excluded | The article was excluded based on title and abstract screening as it did not meet the inclusion and exclusion criteria. |
| Hippocampal increase of 5-hmC in the glucocorticoid receptor gene following acute stress | 2015 | Li, S. and Papale, L.A. and Kintner, D.B. and Sabat, G. and Barrett-Wilt, G.A. and Cengiz, P. and Alisch, R.S. | Excluded | The article was excluded based on title and abstract screening as it did not meet the inclusion and exclusion criteria. |
| Ibuprofen intake increases exercise time to exhaustion: A possible role for preventing exercise-induced fatigue | 2016 | Lima, F.D. and Stamm, D.N. and Della Pace, I.D. and Ribeiro, L.R. and Rambo, L.M. and Bresciani, G. and Ferreira, J. and Rossato, M.F. and Silva, M.A. and Pereira, M.E. and Ineu, R.P. and Santos, A.R. and Bobinski, F. and Fighera, M.R. and Royes, L.F.F. | Excluded | The article was excluded based on title and abstract screening as it did not meet the inclusion and exclusion criteria. |
| Protective effect of eugenol against restraint stress-induced gastrointestinal dysfunction: Potential use in irritable bowel syndrome | 2015 | Garabadu, D. and Shah, A. and Singh, S. and Krishnamurthy, S. | Excluded | The article was excluded based on title and abstract screening as it did not meet the inclusion and exclusion criteria. |
| Exercise-mimetic AICAR transiently benefits brain function | 2015 | Guerrieri, D. and van Praag, H. | Excluded | The article was excluded based on title and abstract screening as it did not meet the inclusion and exclusion criteria. |
| The Effect of Western Diet on Cognition in Humans | 2015 | Francis, H.M. and Stevenson, R.J. | Excluded | The article was excluded based on title and abstract screening as it did not meet the inclusion and exclusion criteria. |
| Dynamic regulation of cerebral DNA repair genes by psychological stress | 2015 | Forsberg, K. and Aalling, N. and Wörtwein, G. and Loft, S. and Møller, P. and Hau, J. and Hageman, I. and Jørgensen, M.B. and Jørgensen, A. | Excluded | The article was excluded based on title and abstract screening as it did not meet the inclusion and exclusion criteria. |
| Obesity and neuroinflammation: A pathway to cognitive impairment | 2014 | Miller, A.A. and Spencer, S.J. | Excluded | The article was excluded based on title and abstract screening as it did not meet the inclusion and exclusion criteria. |
| Calorie restriction improves cognitive decline via up-regulation of brain-derived neurotrophic factor: Tropomyosin-related kinase B in hippocampus of obesity-induced hypertensive rats | 2015 | Kishi, T. and Hirooka, Y. and Nagayama, T. and Isegawa, K. and Katsuki, M. and Takesue, K. and Sunagawa, K. | Excluded | The article was excluded based on title and abstract screening as it did not meet the inclusion and exclusion criteria. |
| Voluntary exercise prevents oxidative stress in the brain of phenylketonuria mice | 2016 | Mazzola, P.N. and Bruinenberg, V. and Anjema, K. and van Vliet, D. and Dutra-Filho, C.S. and van Spronsen, F.J. and van der Zee, E.A. | Excluded | The article was excluded based on title and abstract screening as it did not meet the inclusion and exclusion criteria. |
| High-intensity interval training improves cerebellar antioxidant capacity without affecting cognitive functions in rats | 2019 | Freitas, DA and Rocha-Vieira, E and De Sousa, RAL and Soares, BA and Rocha-Gomes, A and Garcia, BCC and Cassilhas, RC and Mendonça, VA and Camargos, ACR and De Gregorio, JAM and Lacerda, ACR and Leite, HR | Included |  |
| Hormetic effects of regular exercise in aging: Correlation with oxidative stress | 2007 | Goto, S. and Naito, H. and Kaneko, T. and Chung, H. and Radák, Z. | Included |  |
| Biomarkers for assessing population and individual health and disease related to stress and adaptation | 2015 | McEwen, B.S. | Excluded | The article was excluded based on title and abstract screening as it did not meet the inclusion and exclusion criteria. |
| Role of myokines in the maintenance of whole-body metabolic homeostasis | 2016 | Kostrominova, T.Y. | Excluded | The article was excluded based on title and abstract screening as it did not meet the inclusion and exclusion criteria. |
| Exercise Training Improves the Altered Renin-Angiotensin System in the Rostral Ventrolateral Medulla of Hypertensive Rats | 2016 | Ren, C.-Z. and Yang, Y.-H. and Sun, J.-C. and Wu, Z.-T. and Zhang, R.-W. and Shen, D. and Wang, Y.-K. | Excluded | The article was excluded based on title and abstract screening as it did not meet the inclusion and exclusion criteria. |
| The traditional drug Gongjin-Dan ameliorates chronic fatigue in a forced-stress mouse exercise model | 2015 | Hong, S.-S. and Lee, J.-Y. and Lee, J.-S. and Lee, H.-W. and Kim, H.-G. and Lee, S.-K. and Park, B.-K. and Son, C.-G. | Excluded | The article was excluded based on title and abstract screening as it did not meet the inclusion and exclusion criteria. |
| Translating mechanisms of neuroprotection, regeneration, and repair to treatment of spinal cord injury | 2015 | Siddiqui, A.M. and Khazaei, M. and Fehlings, M.G. | Excluded | The article was excluded based on title and abstract screening as it did not meet the inclusion and exclusion criteria. |
| Physical Activity, Air Pollution and the Brain | 2014 | Bos, I. and De Boever, P. and Int Panis, L. and Meeusen, R. | Excluded | The article was excluded based on title and abstract screening as it did not meet the inclusion and exclusion criteria. |
| Physical exercise prevents motor disorders and striatal oxidative imbalance after cerebral ischemia-reperfusion | 2015 | Sosa, P.M. and Altermann, C. and Vieira, A.S. and Mello-Carpes, P.B. and Schimidt, H.L. and Carpes, F.P. and Cibin, F.W.S. | Excluded | The article was excluded based on title and abstract screening as it did not meet the inclusion and exclusion criteria. |
| How does physical activity and different models of exercise training affect oxidative parameters and memory? | 2019 | Feter, N. and Spanevello, R.M. and Soares, M.S.P. and Spohr, L. and Pedra, N.S. and Bona, N.P. and Freitas, M.P. and Gonzales, N.G. and Ito, L.G.M.S. and Stefanello, F.M. and Rombaldi, A.J. | Included |  |
| Exercise Regulation of Cognitive Function and Neuroplasticity in the Healthy and Diseased Brain | 2015 | Hamilton, G.F. and Rhodes, J.S. and Bouchard C. | Excluded | The article was excluded based on title and abstract screening as it did not meet the inclusion and exclusion criteria. |
| Influence of chronic exercise on reserpine-induced oxidative stress in rats: Behavioral and antioxidant evaluations | 2008 | Teixeira, A.M. and Trevizol, F. and Colpo, G. and Garcia, S.C. and Charão, M. and Pereira, R.P. and Fachinetto, R. and Rocha, J.B.T. and Bürger, M.E. | Included |  |
| In the rush for green gold: Can green tea delay age-progressive brain neurodegeneration? | 2012 | Mandel, S.A. and Youdim, M.B.H. | Excluded | The article was excluded based on title and abstract screening as it did not meet the inclusion and exclusion criteria. |
| Pharmacological properties of physical exercise in the elderly | 2014 | Viña, J. and Borras, C. and Sanchis-Gomar, F. and Martinez-Bello, V.E. and Olaso-Gonzalez, G. and Gambini, J. and Ingles, M. and Gomez-Cabrera, M.C. | Excluded | The article was excluded based on title and abstract screening as it did not meet the inclusion and exclusion criteria. |
| The effects of cocoa supplementation, caloric restriction, and regular exercise, on oxidative stress markers of brain and memory in the rat model | 2013 | Radák, Z. and Silye, G. and Bartha, C. and Jakus, J. and Stefanovits-Bányai, T. and Atalay, M. and Marton, O. and Koltai, E. | Excluded | The article was excluded based on title and abstract screening as it did not meet the inclusion and exclusion criteria. |
| Activity-dependent, stress-responsive BDNF signaling and the quest for optimal brain health and resilience throughout the lifespan | 2013 | Rothman, S.M. and Mattson, M.P. | Excluded | The article was excluded based on title and abstract screening as it did not meet the inclusion and exclusion criteria. |
| Alzheimer's Disease Related Markers, Cellular Toxicity and Behavioral Deficits Induced Six Weeks after Oligomeric Amyloid-β Peptide Injection in Rats | 2013 | Zussy, C. and Brureau, A. and Keller, E. and Marchal, S. and Blayo, C. and Delair, B. and Ixart, G. and Maurice, T. and Givalois, L. | Excluded | The article was excluded based on title and abstract screening as it did not meet the inclusion and exclusion criteria. |
| Implication of the nutritional and nonnutritional factors in the context of preservation of cognitive performance in patients with dementia/depression and Alzheimer disease | 2013 | Aliev, G. and Ashraf, G.M. and Kaminsky, Y.G. and Sheikh, I.A. and Sudakov, S.K. and Yakhno, N.N. and Benberin, V.V. and Bachurin, S.O. | Excluded | The article was excluded based on title and abstract screening as it did not meet the inclusion and exclusion criteria. |
| The theory of bipolar disorder as an illness of accelerated aging: Implications for clinical care and research | 2014 | Rizzo, L.B. and Costa, L.G. and Mansur, R.B. and Swardfager, W. and Belangero, S.I. and Grassi-Oliveira, R. and McIntyre, R.S. and Bauer, M.E. and Brietzke, E. | Excluded | The article was excluded based on title and abstract screening as it did not meet the inclusion and exclusion criteria. |
| Modifications of skeletal muscle ryanodine receptor type 1 and exercise intolerance in heart failure | 2013 | Rullman, E. and Andersson, D.C. and Melin, M. and Reiken, S. and Mancini, D.M. and Marks, A.R. and Lund, L.H. and Gustafsson, T. | Excluded | The article was excluded based on title and abstract screening as it did not meet the inclusion and exclusion criteria. |
| Repeated short-term daily exercise ameliorates oxidative cerebral damage and the resultant motor dysfunction after transient ischemia in rats | 2013 | Hamakawa, M. and Ishida, A. and Tamakoshi, K. and Shimada, H. and Nakashima, H. and Noguchi, T. and Toyokuni, S. and Ishida, K. | Excluded | The article was excluded based on title and abstract screening as it did not meet the inclusion and exclusion criteria. |
| Antioxidant food supplements and obesity-related inflammation | 2013 | Mangge, H. and Summers, K. and Almer, G. and Prassl, R. and Weghuber, D. and Schnedl, W. and Fuchs, D. | Excluded | The article was excluded based on title and abstract screening as it did not meet the inclusion and exclusion criteria. |
| Resveratrol for prenatal-stress-induced oxidative damage in growing brain and its consequences on survival of neurons | 2014 | Madhyastha, S. and Sahu, S.S. and Rao, G. | Excluded | The article was excluded based on title and abstract screening as it did not meet the inclusion and exclusion criteria. |
| Coenzyme Q10 supplementation and regular physical exercise affect the level of glutathione and superoxide dismutase in the brain | 2013 | Revan, S. and Okudan, N. and Balci, S.S. and Belviranli, M. and Pepe, H. and Gökbel, H. | Excluded | The article was excluded based on title and abstract screening as it did not meet the inclusion and exclusion criteria. |
| Central command dysfunction in rats with heart failure is mediated by brain oxidative stress and normalized by exercise training | 2014 | Koba, S. and Hisatome, I. and Watanabe, T. | Excluded | The article was excluded based on title and abstract screening as it did not meet the inclusion and exclusion criteria. |
| The value of selected in vitro and in silico methods to predict acute oral toxicity in a regulatory context: Results from the European Project ACuteTox | 2013 | Prieto, P. and Kinsner-Ovaskainen, A. and Stanzel, S. and Albella, B. and Artursson, P. and Campillo, N. and Cecchelli, R. and Cerrato, L. and Díaz, L. and Di Consiglio, E. and Guerra, A. and Gombau, L. and Herrera, G. and Honegger, P. and Landry, C. and O'Connor, J.E. and Páez, J.A. and Quintas, G. and Svensson, R. and Turco, L. and Zurich, M.G. and Zurbano, M.J. and Kopp-Schneider, A. | Excluded | The article was excluded based on title and abstract screening as it did not meet the inclusion and exclusion criteria. |
| Late-Onset Running Biphasically Improves Redox Balance, Energy- and Methylglyoxal-Related Status, as well as SIRT1 Expression in Mouse Hippocampus | 2012 | Falone, S. and D'Alessandro, A. and Mirabilio, A. and Cacchio, M. and Di Ilio, C. and Di Loreto, S. and Amicarelli, F. | Excluded | The article was excluded based on title and abstract screening as it did not meet the inclusion and exclusion criteria. |
| Obesity-induced cerebral hypoperfusion derived from endothelial dysfunction: One of the risk factors for alzheimer’s disease | 2014 | Toda, N. and Ayajiki, K. and Okamura, T. | Excluded | The article was excluded based on title and abstract screening as it did not meet the inclusion and exclusion criteria. |
| Clinical perspective on oxidative stress in sporadic amyotrophic lateral sclerosis | 2013 | D'Amico, E. and Factor-Litvak, P. and Santella, R.M. and Mitsumoto, H. | Excluded | The article was excluded based on title and abstract screening as it did not meet the inclusion and exclusion criteria. |
| Seven sirtuins for seven deadly diseases ofaging | 2013 | Morris, B.J. | Excluded | The article was excluded based on title and abstract screening as it did not meet the inclusion and exclusion criteria. |
| BDNF and exercise enhance neuronal DNA repair by stimulating CREB-mediated production of apurinic/apyrimidinic endonuclease 1 | 2014 | Yang, J.-L. and Lin, Y.-T. and Chuang, P.-C. and Bohr, V.A. and Mattson, M.P. | Excluded | The article was excluded based on title and abstract screening as it did not meet the inclusion and exclusion criteria. |
| Fueling and imaging brain activation | 2012 | Dienel, G.A. | Excluded | The article was excluded based on title and abstract screening as it did not meet the inclusion and exclusion criteria. |
| Continuous exercise training and curcumin attenuate changes in brain-derived neurotrophic factor and oxidative stress induced by lead acetate in the hippocampus of male rats | 2013 | Hosseinzadeh, S. and Roshan, V.D. and Mahjoub, S. | Excluded | The article was excluded based on title and abstract screening as it did not meet the inclusion and exclusion criteria. |
| Exercise modulates redox-sensitive small GTPase activity in the brain microvasculature in a model of brain metastasis formation | 2014 | Wolff, G. and Balke, J.E. and Andras, I.E. and Park, M. and Toborek, M. | Excluded | The article was excluded based on title and abstract screening as it did not meet the inclusion and exclusion criteria. |
| Diet and cognition: Interplay between cell metabolism and neuronal plasticity | 2013 | Gomez-Pinilla, F. and Tyagi, E. | Excluded | The article was excluded based on title and abstract screening as it did not meet the inclusion and exclusion criteria. |
| Physical training exerts neuroprotective effects in the regulation of neurochemical factors in an animal model of Parkinson's disease | 2012 | Tuon, T. and Valvassori, S.S. and Lopes-Borges, J. and Luciano, T. and Trom, C.B. and Silva, L.A. and Quevedo, J. and Souza, C.T. and Lira, F.S. and Pinho, R.A. | Excluded | The article was excluded based on title and abstract screening as it did not meet the inclusion and exclusion criteria. |
| The role of mitochondrial dysfunctions due to oxidative and nitrosative stress in the chronic pain or chronic fatigue syndromes and fibromyalgia patients: Peripheral and central mechanisms as therapeutic targets? | 2013 | Meeus, M. and Nijs, J. and Hermans, L. and Goubert, D. and Calders, P. | Excluded | The article was excluded based on title and abstract screening as it did not meet the inclusion and exclusion criteria. |
| Memory deficits and oxidative stress in cerebral ischemia-reperfusion: Neuroprotective role of physical exercise and green tea supplementation | 2014 | Schimidt, H.L. and Vieira, A. and Altermann, C. and Martins, A. and Sosa, P. and Santos, F.W. and Mello-Carpes, P.B. and Izquierdo, I. and Carpes, F.P. | Excluded | The article was excluded based on title and abstract screening as it did not meet the inclusion and exclusion criteria. |
| Intense long-term training impairs brain health compared with moderate exercise: Experimental evidence and mechanisms | 2022 | Sangüesa, G. and Batlle, M. and Muñoz-Moreno, E. and Soria, G. and Alcarraz, A. and Rubies, C. and Sitjà-Roqueta, L. and Solana, E. and Martínez-Heras, E. and Meza-Ramos, A. and Amaro, S. and Llufriu, S. and Mont, L. and Guasch, E. | Included |  |
| Chronic Intermittent Fasting Improves Cognitive Functions and Brain Structures in Mice | 2013 | Li, L. and Wang, Z. and Zuo, Z. | Excluded | The article was excluded based on title and abstract screening as it did not meet the inclusion and exclusion criteria. |
| Sustained running in rats administered corticosterone prevents the development of depressive behaviors and enhances hippocampal neurogenesis and synaptic plasticity without increasing neurotrophic factor levels | 2014 | Yau, S.-Y. and Li, A. and Zhang, E.-D. and Christie, B.R. and Xu, A. and Lee, T.M.C. and So, K.-F. | Excluded | The article was excluded based on title and abstract screening as it did not meet the inclusion and exclusion criteria. |
| A study on the antioxidant effect of Coriolus versicolor polysaccharide in rat brain tissues. | 2013 | Chen, J. and Jin, X. and Zhang, L. and Yang, L. | Excluded | The article was excluded based on title and abstract screening as it did not meet the inclusion and exclusion criteria. |
| Protective effects of ginsenoside Rg1 on chronic restraint stress induced learning and memory impairments in male mice | 2014 | Wang, Y. and Kan, H. and Yin, Y. and Wu, W. and Hu, W. and Wang, M. and Li, W. and Li, W. | Excluded | The article was excluded based on title and abstract screening as it did not meet the inclusion and exclusion criteria. |
| Myalgic encephalomyelitis/chronic fatigue syndrome and encephalomyelitis disseminata/multiple sclerosis show remarkable levels of similarity in phenomenology and neuroimmune characteristics | 2013 | Morris, G. and Maes, M. | Excluded | The article was excluded based on title and abstract screening as it did not meet the inclusion and exclusion criteria. |
| Exercising the worry away: How inflammation, oxidative and nitrogen stress mediates the beneficial effect of physical activity on anxiety disorder symptoms and behaviours | 2013 | Moylan, S. and Eyre, H.A. and Maes, M. and Baune, B.T. and Jacka, F.N. and Berk, M. | Excluded | The article was excluded based on title and abstract screening as it did not meet the inclusion and exclusion criteria. |
| Exercise ameliorates cognition impairment due to restraint stress-induced oxidative insult and reduced BDNF level | 2013 | Kwon, D.-H. and Kim, B.-S. and Chang, H. and Kim, Y.-I. and Jo, S.A. and Leem, Y.-H. | Excluded | The article was excluded based on title and abstract screening as it did not meet the inclusion and exclusion criteria. |
| Pathological parainflammation and endoplasmic reticulum stress in depression: Potential translational targets through the CNS insulin, klotho and PPAR-γ systems | 2013 | Gold, P.W. and Licinio, J. and Pavlatou, M.G. | Excluded | The article was excluded based on title and abstract screening as it did not meet the inclusion and exclusion criteria. |
| Mitochondria impact brain function and cognition | 2014 | Picard, M. and McEwen, B.S. | Excluded | The article was excluded based on title and abstract screening as it did not meet the inclusion and exclusion criteria. |
| Chronic ethanol exposure during adolescence in rats induces motor impairments and cerebral cortex damage associated with oxidative stress | 2014 | Teixeira, F.B. and Santana, L.N.D.S. and Bezerra, F.R. and De Carvalho, S. and Fontes Jr., E.A. and Prediger, R.D. and Crespo-López, M.E. and Maia, C.S.F. and Lima, R.R. | Excluded | The article was excluded based on title and abstract screening as it did not meet the inclusion and exclusion criteria. |
| Aerobic endurance capacity affects spatial memory and SIRT1 is a potent modulator of 8-oxoguanine repair | 2013 | Sarga, L. and Hart, N. and Koch, L.G. and Britton, S.L. and Hajas, G. and Boldogh, I. and Ba, X. and Radak, Z. | Excluded | The article was excluded based on title and abstract screening as it did not meet the inclusion and exclusion criteria. |
| Increased oxidative stress is associated with the development of organophosphate-induced delayed neuropathy | 2012 | Masoud, A. and Sandhir, R. | Excluded | The article was excluded based on title and abstract screening as it did not meet the inclusion and exclusion criteria. |
| Long-term consequences of developmental alcohol exposure on brain structure and function: Therapeutic benefits of physical activity | 2013 | Klintsova, A.Y. and Hamilton, G.F. and Boschen, K.E. | Excluded | The article was excluded based on title and abstract screening as it did not meet the inclusion and exclusion criteria. |
| Physical activity and the endocannabinoid system: An overview | 2014 | Tantimonaco, M. and Ceci, R. and Sabatini, S. and Catani, M.V. and Rossi, A. and Gasperi, V. and Maccarrone, M. | Excluded | The article was excluded based on title and abstract screening as it did not meet the inclusion and exclusion criteria. |
| Vigorous Exercise Training Improves Reactivity of Cerebral Arterioles and Reduces Brain Injury Following Transient Focal Ischemia | 2014 | Arrick, D.M. and Yang, S. and Li, C. and Cananzi, S. and Mayhan, W.G. | Excluded | The article was excluded based on title and abstract screening as it did not meet the inclusion and exclusion criteria. |
| Endothelial nitric oxide: Protector of a healthy mind | 2014 | Katusic, Z.S. and Austin, S.A. | Excluded | The article was excluded based on title and abstract screening as it did not meet the inclusion and exclusion criteria. |
| Exercise facilitates the action of dietary DHA on functional recovery after brain trauma | 2013 | Wu, A. and Ying, Z. and Gomez-Pinilla, F. | Excluded | The article was excluded based on title and abstract screening as it did not meet the inclusion and exclusion criteria. |
| Sleep and the single neuron: The role of global slow oscillations in individual cell rest | 2013 | Vyazovskiy, V.V. and Harris, K.D. | Excluded | The article was excluded based on title and abstract screening as it did not meet the inclusion and exclusion criteria. |
| Antidepressant-like behavioral, anatomical, and biochemical effects of petroleum ether extract from maca (Lepidium meyenii) in mice exposed to chronic unpredictable mild stress | 2014 | Ai, Z. and Cheng, A.-F. and Yu, Y.-T. and Yu, L.-J. and Jin, W. | Excluded | The article was excluded based on title and abstract screening as it did not meet the inclusion and exclusion criteria. |
| Biochemical, biomedical and metabolic aspects of imidazole-containing dipeptides with the inherent complexity to neurodegenerative diseases and various states of mental well-being: A challenging correction and neurotherapeutic pharmaceutical biotechnology for treating cognitive deficits, depressi... | 2014 | Babizhayev, M.A. | Excluded | The article was excluded based on title and abstract screening as it did not meet the inclusion and exclusion criteria. |
| Chronic exercise training versus acute endurance exercise in reducing neurotoxicity in rats exposed to lead acetate | 2013 | Shahandeh, M. and Roshan, V.D. and Hosseinzadeh, S. and Mahjoub, S. and Sarkisian, V. | Excluded | The article was excluded based on title and abstract screening as it did not meet the inclusion and exclusion criteria. |
| Nutrition and the biology of human ageing: Cognitive decline/food intake & caloric restriction | 2013 | Le Coutre, J. and Mattson, M.P. and Dillin, A. and Friedman, J. and Bistrian, B. | Excluded | The article was excluded based on title and abstract screening as it did not meet the inclusion and exclusion criteria. |
| Exercise modifies amphetamine relapse: Behavioral and oxidative markers in rats | 2014 | Segat, H.J. and Kronbauer, M. and Roversi, K. and Schuster, A.J. and Vey, L.T. and Roversi, K. and Pase, C.S. and Antoniazzi, C.T.D. and Burger, M.E. | Excluded | The article was excluded based on title and abstract screening as it did not meet the inclusion and exclusion criteria. |
| Oophorectomy hinders antioxidant adaptation promoted by swimming in wistar rats | 2013 | Macedo, U.B.O. and Martins, R.R. and Freire Neto, F.P. and Oliveira, Y.M.C. and Medeiros, A.C. and Brandão-Neto, J. and de Rezende, A.A. and Almeida, M.G. | Excluded | The article was excluded based on title and abstract screening as it did not meet the inclusion and exclusion criteria. |
| Antioxidant treatment strategies for hyperphenylalaninemia | 2013 | Mazzola, P.N. and Karikas, G.A. and Schulpis, K.H. and Dutra-Filho, C.S. | Excluded | The article was excluded based on title and abstract screening as it did not meet the inclusion and exclusion criteria. |
| Chronic treadmill running protects hippocampal neurons from hypobaric hypoxia-induced apoptosis in rats | 2013 | Lin, C. and Wu, C.-J. and Wei, I.-H. and Tsai, M.-H. and Chang, N.-W. and Yang, T.-T. and Kuo, Y.-M. | Excluded | The article was excluded based on title and abstract screening as it did not meet the inclusion and exclusion criteria. |
| Healing hearts and minds: Can antidepressants repair the heart? | 2012 | Murphy, A.M. | Excluded | The article was excluded based on title and abstract screening as it did not meet the inclusion and exclusion criteria. |
| Basic models modeling resistance training: An update for basic scientists interested in study skeletal muscle hypertrophy | 2014 | Cholewa, J. and Guimarães-Ferreira, L. and da Silva Teixeira, T. and Naimo, M.A. and Zhi, X. and de Sá, R.B.D.P. and Lodetti, A. and Cardozo, M.Q. and Zanchi, N.E. | Excluded | The article was excluded based on title and abstract screening as it did not meet the inclusion and exclusion criteria. |
| The dose-dependent antioxidant effects of physical exercise in the hippocampus of mice | 2014 | Speck, A.E. and Tromm, C.B. and Pozzi, B.G. and Paganini, C.S. and Tuon, T. and Silveira, P.C.L. and Aguiar, A.S. and Pinho, R.A. | Excluded | The article was excluded based on title and abstract screening as it did not meet the inclusion and exclusion criteria. |
| Cell biology of normal brain aging: Synaptic plasticity-cell death | 2013 | Dorszewska, J. | Excluded | The article was excluded based on title and abstract screening as it did not meet the inclusion and exclusion criteria. |
| Swim training attenuates oxidative damage and promotes neuroprotection in cerebral cortical slices submitted to oxygen glucose deprivation | 2012 | Leite, H.R. and Mourão, F.A.G. and Drumond, L.E. and Ferreira-Vieira, T.H. and Bernardes, D. and Silva, J.F. and Lemos, V.S. and Moraes, M.F.D. and Pereira, G.S. and Carvalho-Tavares, J. and Massensini, A.R. | Excluded | The article was excluded based on title and abstract screening as it did not meet the inclusion and exclusion criteria. |
| S100B as a marker for brain damage and blood-brain barrier disruption following exercise | 2014 | Koh, S.X.T. and Lee, J.K.W. | Excluded | The article was excluded based on title and abstract screening as it did not meet the inclusion and exclusion criteria. |
| Exercise-Induced Neuroprotection of Hippocampus in APP/PS1 Transgenic Mice via Upregulation of Mitochondrial 8-Oxoguanine DNA Glycosylase | 2014 | Bo, H. and Kang, W. and Jiang, N. and Wang, X. and Zhang, Y. and Ji, L.L. | Excluded | The article was excluded based on title and abstract screening as it did not meet the inclusion and exclusion criteria. |
| Physical exercise as a possible strategy for brain protection: Evidence from mitochondrial-mediated mechanisms | 2012 | Marques-Aleixo, I. and Oliveira, P.J. and Moreira, P.I. and Magalhães, J. and Ascensão, A. | Excluded | The article was excluded based on title and abstract screening as it did not meet the inclusion and exclusion criteria. |
| Implications of oxidative damage to proteins and DNA in aging and its intervention by caloric restriction and exercise | 2013 | Goto, S. and Radak, Z. | Excluded | The article was excluded based on title and abstract screening as it did not meet the inclusion and exclusion criteria. |
| Aerobic exercise combined with antioxidative treatment does not counteract moderate- or mid-stage alzheimer-like pathophysiology of APP/PS1 mice | 2013 | Xu, Z.-Q. and Zhang, L.-Q. and Wang, Q. and Marshall, C. and Xiao, N. and Gao, J.-Y. and Wu, T. and Ding, J. and Hu, G. and Xiao, M. | Excluded | The article was excluded based on title and abstract screening as it did not meet the inclusion and exclusion criteria. |
| Creatine, L-Carnitine, and ω 3 Polyunsaturated Fatty Acid Supplementation from Healthy to Diseased Skeletal Muscle | 2014 | D'Antona, G. and Nabavi, S.M. and Micheletti, P. and Di Lorenzo, A. and Aquilani, R. and Nisoli, E. and Rondanelli, M. and Daglia, M. | Excluded | The article was excluded based on title and abstract screening as it did not meet the inclusion and exclusion criteria. |
| Does exercise reduce brain oxidative stress? A systematic review | 2013 | Camiletti-Moirón, D. and Aparicio, V.A. and Aranda, P. and Radak, Z. | Excluded | The article was excluded based on title and abstract screening as it did not meet the inclusion and exclusion criteria. |
| Detraining Differentially Preserved Beneficial Effects of Exercise on Hypertension: Effects on Blood Pressure, Cardiac Function, Brain Inflammatory Cytokines and Oxidative Stress | 2012 | Agarwal, D. and Dange, R.B. and Vila, J. and Otamendi, A.J. and Francis, J. | Excluded | The article was excluded based on title and abstract screening as it did not meet the inclusion and exclusion criteria. |
| Theacrine, a purine alkaloid obtained from Camellia assamica var. kucha, attenuates restraint stress-provoked liver damage in mice | 2013 | Li, W.-X. and Li, Y.-F. and Zhai, Y.-J. and Chen, W.-M. and Kurihara, H. and He, R.-R. | Excluded | The article was excluded based on title and abstract screening as it did not meet the inclusion and exclusion criteria. |
| Impact of exercise training on preeclampsia: Potential preventive mechanisms | 2012 | Genest, D.S. and Falcao, S. and Gutkowska, J. and Lavoie, J.L. | Excluded | The article was excluded based on title and abstract screening as it did not meet the inclusion and exclusion criteria. |
| Voluntary exercise protects against methamphetamine-induced oxidative stress in brain microvasculature and disruption of the blood-brain barrier | 2013 | Toborek, M. and Seelbach, M.J. and Rashid, C.S. and András, I.E. and Chen, L. and Park, M. and Esser, K.A. | Excluded | The article was excluded based on title and abstract screening as it did not meet the inclusion and exclusion criteria. |
| Exercise protects against chronic restraint stress-induced oxidative stress in the cortex and hippocampus | 2013 | Gerecke, K.M. and Kolobova, A. and Allen, S. and Fawer, J.L. | Excluded | The article was excluded based on title and abstract screening as it did not meet the inclusion and exclusion criteria. |
| Neuroprotective effect of physical exercise in a mouse model of alzheimer's disease induced by β-Amyloid1-40 peptide | 2013 | Souza, L.C. and Filho, C.B. and Goes, A.T.R. and Fabbro, L.D. and De Gomes, M.G. and Savegnago, L. and Oliveira, M.S. and Jesse, C.R. | Excluded | The article was excluded based on title and abstract screening as it did not meet the inclusion and exclusion criteria. |
| Mussel oligopeptides ameliorate cognition deficit and attenuate brain senescence in d-galactose-induced aging mice | 2013 | Zhou, Y. and Dong, Y. and Xu, Q. and He, Y. and Tian, S. and Zhu, S. and Zhu, Y. and Dong, X. | Excluded | The article was excluded based on title and abstract screening as it did not meet the inclusion and exclusion criteria. |
| Quercetin enhances exercise-mediated neuroprotective effects in brain ischemic rats | 2014 | Chang, H.-C. and Yang, Y.-R. and Wang, P.S. and Wang, R.-Y. | Excluded | The article was excluded based on title and abstract screening as it did not meet the inclusion and exclusion criteria. |
| Biomarkers for ischemic preconditioning: Finding the responders | 2014 | Koch, S. and Della-Morte, D. and Dave, K.R. and Sacco, R.L. and Perez-Pinzon, M.A. | Excluded | The article was excluded based on title and abstract screening as it did not meet the inclusion and exclusion criteria. |
| Exercise training reverses the deleterious effect of sucrose intake on insulin resistance and visceral fat mass deposition on mice | 2014 | Soares, J.C.M. and Folmer, V. and Puntel, G. and Da Rocha, J.B.T. | Excluded | The article was excluded based on title and abstract screening as it did not meet the inclusion and exclusion criteria. |
| Prenatal treatment of Down syndrome: A reality? | 2014 | Guedj, F. and Bianchi, D.W. and Delabar, J.-M. | Excluded | The article was excluded based on title and abstract screening as it did not meet the inclusion and exclusion criteria. |
| Vascular risk factors and neurodegeneration in ageing related dementias: Alzheimer's disease and vascular dementia | 2013 | Akinyemi, R.O. and Mukaetova-Ladinska, E.B. and Attems, J. and Ihara, M. and Kalaria, R.N. | Excluded | The article was excluded based on title and abstract screening as it did not meet the inclusion and exclusion criteria. |
| Combined impact of exercise and temperature in learning and memory performance of fluoride toxicated rats | 2012 | Basha, P.M. and Sujitha, N.S. | Excluded | The article was excluded based on title and abstract screening as it did not meet the inclusion and exclusion criteria. |
| New vistas for the treatment of obesity: Turning the tide against the leading cause of morbidity and cardiovascular mortality in the developed world | 2014 | Lepor, N.E. and Fouchia, D.D. and McCullough, P.A. | Excluded | The article was excluded based on title and abstract screening as it did not meet the inclusion and exclusion criteria. |
| Aerobic exercise effects on neuroprotection and brain repair following stroke: A systematic review and perspective | 2014 | Austin, M.W. and Ploughman, M. and Glynn, L. and Corbett, D. | Excluded | The article was excluded based on title and abstract screening as it did not meet the inclusion and exclusion criteria. |
| Role of mitochondrial homeostasis and dynamics in Alzheimer's disease | 2013 | Selfridge, J.E. and Lezi, E. and Lu, J. and Swerdlow, R.H. | Excluded | The article was excluded based on title and abstract screening as it did not meet the inclusion and exclusion criteria. |
| The redox-associated adaptive response of brain to physical exercise | 2014 | Radak, Z. and Ihasz, F. and Koltai, E. and Goto, S. and Taylor, A.W. and Boldogh, I. | Excluded | The article was excluded based on title and abstract screening as it did not meet the inclusion and exclusion criteria. |
| Preconditioning for Traumatic Brain Injury | 2013 | Yokobori, S. and Mazzeo, A.T and Hosein, K. and Gajavelli, S. and Dietrich, W.D. and Bullock, M.R. | Excluded | The article was excluded based on title and abstract screening as it did not meet the inclusion and exclusion criteria. |
| Assessing competence of broccoli consumption on inflammatory and antioxidant pathways in restraint-induced models: Estimation in rat hippocampus and prefrontal cortex | 2013 | Khalaj, L. and Chavoshi Nejad, S. and Mohammadi, M. and Sarraf Zadeh, S. and Hossein Pour, M. and Ashabi, G. and Khodagholi, F. and Ahmadiani, A. | Excluded | The article was excluded based on title and abstract screening as it did not meet the inclusion and exclusion criteria. |
| Interaction of exercise training and chronic ethanol ingestion on antioxidant system of rat brain regions | 1997 | Somani, S.M. and Husain, K. | Included |  |
| Novel mechanistic insights into treadmill exercise based rescue of social defeat-induced anxiety-like behavior and memory impairment in rats | 2014 | Patki, G. and Solanki, N. and Atrooz, F. and Ansari, A. and Allam, F. and Jannise, B. and Maturi, J. and Salim, S. | Excluded | The article was excluded based on title and abstract screening as it did not meet the inclusion and exclusion criteria. |
| Effects of drugs on exercise performance | 2013 | Baulin, S.I. and Rogacheva, S.M. and Afanaseva, S.V. | Excluded | The article was excluded based on title and abstract screening as it did not meet the inclusion and exclusion criteria. |
| Pre-ischemic exercise alleviates oxidative damage following ischemic stroke in rats | 2014 | Feng, R. and Zhang, M. and Wang, X. and Li, W.-B. and Ren, S.-Q. and Zhang, F. | Excluded | The article was excluded based on title and abstract screening as it did not meet the inclusion and exclusion criteria. |
| Mechanisms involved in oxidative stress regulation | 2013 | Kouretas, D. and Tsatsakis, A.M. and Domingo, J.L. and Wallace Hayes, A. | Excluded | The article was excluded based on title and abstract screening as it did not meet the inclusion and exclusion criteria. |
| A review of lifestyle factors that contribute to important pathways associated with major depression: Diet, sleep and exercise | 2013 | Lopresti, A.L. and Hood, S.D. and Drummond, P.D. | Excluded | The article was excluded based on title and abstract screening as it did not meet the inclusion and exclusion criteria. |
| Antioxidant effect of organic purple grape juice on exhaustive exercise | 2013 | Dalla Corte, C.L. and de Carvalho, N.R. and Amaral, G.P. and Puntel, G.O. and Silva, L.F.A. and Retamoso, L.T. and Royes, L.F.F. and Bresciani, G.B. and da Cruz, I.B.M. and Rocha, J.B.T. and Barrio Lera, J.P. and Soares, F.A.A. | Excluded | The article was excluded based on title and abstract screening as it did not meet the inclusion and exclusion criteria. |
| Pharmacological approach to cardiovascular risk in metabolic syndrome | 2013 | Bellis, A. and Trimarco, B. | Excluded | The article was excluded based on title and abstract screening as it did not meet the inclusion and exclusion criteria. |
| Time-dependent effects of training on cardiovascular control in spontaneously hypertensive rats: Role for Brain oxidative stress and inflammation and baroreflex sensitivity | 2014 | Masson, G.S. and Costa, T.S.R. and Yshii, L. and Fernandes, D.C. and Soares, P.P.S. and Laurindo, F.R. and Scavone, C. and Michelini, L.C. | Excluded | The article was excluded based on title and abstract screening as it did not meet the inclusion and exclusion criteria. |
| Long term running biphasically improves methylglyoxal-related metabolism, redox homeostasis and neurotrophic support within adult mouse brain cortex | 2012 | Falone, S. and D'Alessandro, A. and Mirabilio, A. and Petruccelli, G. and Cacchio, M. and Di Ilio, C. and Di Loreto, S. and Amicarelli, F. | Excluded | The article was excluded based on title and abstract screening as it did not meet the inclusion and exclusion criteria. |
| Exercise normalizes altered expression of proteins in the ventral hippocampus of rats subjected to maternal separation | 2012 | Daniels, W.M.U. and Marais, L. and Stein, D.J. and Russell, V.A. | Excluded | The article was excluded based on title and abstract screening as it did not meet the inclusion and exclusion criteria. |
| The influence of stress on the content of free radical oxidation products in subcellular brain fractions in rats at pubertal age | 2009 | Davydov, V.V. and Volkova, Y.V. | Excluded | The article was excluded based on title and abstract screening as it did not meet the inclusion and exclusion criteria. |
| Different approaches, one target: Understanding cellular mechanisms of Parkinson's and Alzheimer's diseases | 2012 | Torrão, A.S. and Café-Mendes, C.C. and Real, C.C. and Hernandes, M.S. and Ferreira, A.F.B. and Santos, T.O. and Chaves-Kirsten, G.P. and Mazucanti, C.H.Y. and Ferro, E.S. and Scavone, C. and Britto, L.R.G. | Excluded | The article was excluded based on title and abstract screening as it did not meet the inclusion and exclusion criteria. |
| Physical exercise protects against Alzheimer's disease in 3xTg-AD mice | 2011 | García-Mesa, Y. and López-Ramos, J.C. and Giménez-Llort, L. and Revilla, S. and Guerra, R. and Gruart, A. and Laferla, F.M. and Cristòfol, R. and Delgado-García, J.M. and Sanfeliu, C. | Excluded | The article was excluded based on title and abstract screening as it did not meet the inclusion and exclusion criteria. |
| Melatonin plus exercise-based neurorehabilitative therapy for spinal cord injury | 2010 | Hong, Y. and Palaksha, K.J. and Park, K. and Park, S. and Kim, H.-D. and Reiter, R.J. and Chang, K.-T. | Excluded | The article was excluded based on title and abstract screening as it did not meet the inclusion and exclusion criteria. |
| Combined influence of intermittent exercise and temperature stress on the modulation of fluoride toxicity | 2012 | Basha, P.M. and Sujitha, N.S. | Excluded | The article was excluded based on title and abstract screening as it did not meet the inclusion and exclusion criteria. |
| Life-long spontaneous exercise does not prolong lifespan but improves health span in mice. | 2013 | Garcia-Valles R and Gomez-Cabrera MC and Rodriguez-Mañas L and Garcia-Garcia FJ and Diaz A and Noguera I and Olaso-Gonzalez G and Viña J | Included |  |
| Chronic exercise modulates RAS components and improves balance between pro-and anti-inflammatory cytokines in the brain of SHR | 2011 | Agarwal, D. and Welsch, M.A. and Keller, J.N. and Francis, J. | Excluded | The article was excluded based on title and abstract screening as it did not meet the inclusion and exclusion criteria. |
| Exercise and Bipolar Disorder: A Review of Neurobiological Mediators | 2009 | Alsuwaidan, M.T. and Kucyi, A. and Law, C.W.Y. and McIntyre, R.S. | Excluded | The article was excluded based on title and abstract screening as it did not meet the inclusion and exclusion criteria. |
| Effect of acute and chronic exercise on oxidant-antioxidant equilibrium in rat hippocampus, prefrontal cortex and striatum | 2009 | Aksu, I. and Topcu, A. and Camsari, U.M. and Acikgoz, O. | Excluded | The article was excluded based on title and abstract screening as it did not meet the inclusion and exclusion criteria. |
| Triggering endogenous neuroprotective processes through exercise in models of dopamine deficiency | 2009 | Zigmond, M.J. and Cameron, J.L. and Leak, R.K. and Mirnics, K. and Russell, V.A. and Smeyne, R.J. and Smith, A.D. | Excluded | The article was excluded based on title and abstract screening as it did not meet the inclusion and exclusion criteria. |
| Adult neurogenesis transiently generates oxidative stress | 2012 | Walton, N.M. and Shin, R. and Tajinda, K. and Heusner, C.L. and Kogan, J.H. and Miyake, S. and Chen, Q. and Tamura, K. and Matsumoto, M. | Excluded | The article was excluded based on title and abstract screening as it did not meet the inclusion and exclusion criteria. |
| Anxiety- and depression-like behaviors are accompanied by an increase in oxidative stress in a rat model of fetal alcohol spectrum disorders: Protective effects of voluntary physical exercise | 2012 | Brocardo, P.S. and Boehme, F. and Patten, A. and Cox, A. and Gil-Mohapel, J. and Christie, B.R. | Excluded | The article was excluded based on title and abstract screening as it did not meet the inclusion and exclusion criteria. |
| Biogerontology research in Israel | 2011 | Globerson, A. and Reznick, A.Z. | Excluded | The article was excluded based on title and abstract screening as it did not meet the inclusion and exclusion criteria. |
| Nutrition and physical activity in NAFLD: An overview of the epidemiological evidence | 2011 | Zelber-Sagi, S. and Ratziu, V. and Oren, R. | Excluded | The article was excluded based on title and abstract screening as it did not meet the inclusion and exclusion criteria. |
| Aging brain: Prevention of oxidative stress by vitamin E and exercise | 2009 | Asha Devi, S. | Excluded | The article was excluded based on title and abstract screening as it did not meet the inclusion and exclusion criteria. |
| Dosimetry and toxicology of inhaled ultrafine particles | 2009 | Schmid, O. and Möller, W. and Semmler-Behnke, M. and A. Ferron, G. and Karg, E. and Lipka, J. and Schulz, H. and Kreyling, W.G. and Stoeger, T. | Excluded | The article was excluded based on title and abstract screening as it did not meet the inclusion and exclusion criteria. |
| Chronic renal failure alters endothelial function in cerebral circulation in mice | 2011 | Bugnicourt, J.-M. and da Silveira, C. and Bengrine, A. and Godefroy, O. and Baumbach, G. and Sevestre, H. and Bode-Boeger, S.M. and Kielstein, J.T. and Massy, Z.A. and Chillon, J.-M. | Excluded | The article was excluded based on title and abstract screening as it did not meet the inclusion and exclusion criteria. |
| Exercise prevents sleep deprivation-associated anxiety-like behavior in rats: Potential role of oxidative stress mechanisms | 2011 | Vollert, C. and Zagaar, M. and Hovatta, I. and Taneja, M. and Vu, A. and Dao, A. and Levine, A. and Alkadhi, K. and Salim, S. | Excluded | The article was excluded based on title and abstract screening as it did not meet the inclusion and exclusion criteria. |
| Baroreflex modulation during sleep and in obstructive sleep apnea syndrome | 2012 | Cortelli, P. and Lombardi, C. and Montagna, P. and Parati, G. | Excluded | The article was excluded based on title and abstract screening as it did not meet the inclusion and exclusion criteria. |
| Does vigorous exercise have a neuroprotective effect in Parkinson disease? | 2011 | Ahlskog, J.E. | Excluded | The article was excluded based on title and abstract screening as it did not meet the inclusion and exclusion criteria. |
| The effect of exercise and oxidant-antioxidant intervention on the levels of neurotrophins and free radicals in spinal cord of rats | 2009 | Siamilis, S. and Jakus, J. and Nyakas, C. and Costa, A. and Mihalik, B. and Falus, A. and Radak, Z. | Excluded | The article was excluded based on title and abstract screening as it did not meet the inclusion and exclusion criteria. |
| Ascorbic acid protects against restraint stress-induced memory deficits in wistar rats | 2009 | Kumar, R.S. and Narayanan, S.N. and Nayak, S. | Excluded | The article was excluded based on title and abstract screening as it did not meet the inclusion and exclusion criteria. |
| Stress, exercise, and Alzheimer's disease: A neurovascular pathway | 2011 | Nation, D.A. and Hong, S. and Jak, A.J. and Delano-Wood, L. and Mills, P.J. and Bondi, M.W. and Dimsdale, J.E. | Excluded | The article was excluded based on title and abstract screening as it did not meet the inclusion and exclusion criteria. |
| Immune senescence and brain aging: can rejuvenation of immunity reverse memory loss? | 2009 | Ron-Harel, N. and Schwartz, M. | Excluded | The article was excluded based on title and abstract screening as it did not meet the inclusion and exclusion criteria. |
| Central mechanisms of abnormal sympathoexcitation in chronic heart failure | 2012 | Kishi, T. and Hirooka, Y. | Excluded | The article was excluded based on title and abstract screening as it did not meet the inclusion and exclusion criteria. |
| In vivo antioxidant status: A putative target of antidepressant action | 2009 | Zafir, A. and Ara, A. and Banu, N. | Excluded | The article was excluded based on title and abstract screening as it did not meet the inclusion and exclusion criteria. |
| Physical activity attenuates intermittent hypoxia-induced spatial learning deficits and oxidative stress | 2010 | Gozal, D. and Nair, D. and Goldbart, A.D. | Excluded | The article was excluded based on title and abstract screening as it did not meet the inclusion and exclusion criteria. |
| Can aerobic training restore the level of BDNF in the hippocampus of rats exposed to lead acetate? | 2011 | Shahandeh, M. and Dabidi Roshan, V. and Mahjoub, S. and Sarkisian, V. | Excluded | The article was excluded based on title and abstract screening as it did not meet the inclusion and exclusion criteria. |
| Aging: An important factor for the pathogenesis of neurodegenerative diseases | 2009 | Farooqui, T. and Farooqui, A.A. | Excluded | The article was excluded based on title and abstract screening as it did not meet the inclusion and exclusion criteria. |
| Oxidative stress: Biomarkers and novel therapeutic pathways | 2010 | Maiese, K. and Chong, Z.Z. and Hou, J. and Shang, Y.C. | Excluded | The article was excluded based on title and abstract screening as it did not meet the inclusion and exclusion criteria. |
| Neurobiological basis of chemotherapy-induced cognitive impairment: A review of rodent research | 2011 | Seigers, R. and Fardell, J.E. | Excluded | The article was excluded based on title and abstract screening as it did not meet the inclusion and exclusion criteria. |
| Analysis of uncoupling protein 2-deficient mice upon anaesthesia and sedation revealed a role for UCP2 in locomotion | 2012 | Alves-Guerra, M.-C. and Aheng, C. and Pecqueur, C. and Masscheleyn, S. and Tharaux, P.L. and Druilhe, A. and Ricquier, D. and Challet, E. and Miroux, B. | Excluded | The article was excluded based on title and abstract screening as it did not meet the inclusion and exclusion criteria. |
| Exercise and physical therapy in early management of Parkinson disease | 2011 | Alonso-Frech, F. and Sanahuja, J.J. and Rodriguez, A.M. | Excluded | The article was excluded based on title and abstract screening as it did not meet the inclusion and exclusion criteria. |
| Physical exercise exacerbates memory deficits induced by intracerebroventricular stz but improves insulin regulation of H 2O2 production in mice synaptosomes | 2012 | Muller, A.P. and Zimmer, E.R. and Haas, C.B. and Oses, J.P. and Martimbianco De Assis, A. and Galina, A. and Souza, D.O. and Portela, L.V. | Excluded | The article was excluded based on title and abstract screening as it did not meet the inclusion and exclusion criteria. |
| Forced treadmill exercise prevents oxidative stress and memory deficits following chronic cerebral hypoperfusion in the rat | 2012 | Cechetti, F. and Worm, P.V. and Elsner, V.R. and Bertoldi, K. and Sanches, E. and Ben, J. and Siqueira, I.R. and Netto, C.A. | Excluded | The article was excluded based on title and abstract screening as it did not meet the inclusion and exclusion criteria. |
| Exercise training normalizes impaired NOS-dependent responses of cerebral arterioles in type 1 diabetic rats | 2011 | Mayhan, W.G. and Arrick, D.M. and Patel, K.P. and Sun, H. | Excluded | The article was excluded based on title and abstract screening as it did not meet the inclusion and exclusion criteria. |
| Exercise training and experimental diabetes modulate heat shock protein response in brain | 2010 | Lappalainen, Z. and Lappalainen, J. and Oksala, N.K.J. and Laaksonen, D.E. and Khanna, S. and Sen, C.K. and Atalay, M. | Excluded | The article was excluded based on title and abstract screening as it did not meet the inclusion and exclusion criteria. |
| Recruiting adaptive cellular stress responses for successful brain ageing | 2012 | Stranahan, A.M. and Mattson, M.P. | Excluded | The article was excluded based on title and abstract screening as it did not meet the inclusion and exclusion criteria. |
| Cardiac aging: From molecular mechanisms to significance in human health and disease | 2012 | Dai, D.-F. and Chen, T. and Johnson, S.C. and Szeto, H. and Rabinovitch, P.S. | Excluded | The article was excluded based on title and abstract screening as it did not meet the inclusion and exclusion criteria. |
| Treadmill training restores spatial cognitive deficits and neurochemical alterations in the hippocampus of rats submitted to an intracerebroventricular administration of streptozotocin | 2010 | Rodrigues, L. and Dutra, M.F. and Ilha, J. and Biasibetti, R. and Quincozes-Santos, A. and Leite, M.C. and Marcuzzo, S. and Achaval, M. and Gonçalves, C.-A. | Excluded | The article was excluded based on title and abstract screening as it did not meet the inclusion and exclusion criteria. |
| Cardiomyopathy of duchenne muscular dystrophy: Current understanding and future directions | 2011 | Spurney, C.F. | Excluded | The article was excluded based on title and abstract screening as it did not meet the inclusion and exclusion criteria. |
| Exercise training causes sympathoinhibition through antioxidant effect in the rostral ventrolateral medulla of hypertensive rats | 2012 | Kishi, T. and Hirooka, Y. and Katsuki, M. and Ogawa, K. and Shinohara, K. and Isegawa, K. and Sunagawa, K. | Excluded | The article was excluded based on title and abstract screening as it did not meet the inclusion and exclusion criteria. |
| Swimming training prevents pentylenetetrazol-induced inhibition of Na +, K+-ATPase activity, seizures, and oxidative stress | 2009 | Souza, M.A. and Oliveira, M.S. and Furian, A.F. and Rambo, L.M. and Ribeiro, L.R. and Lima, F.D. and Corte, L.C.D. and Silva, L.F.A. and Retamoso, L.T. and Corte, C.L.D. and Puntel, G.O. and De Avila, D.S. and Soares, F.A.A. and Fighera, M.R. and De Mello, C.F. and Royes, L.F.F. | Excluded | The article was excluded based on title and abstract screening as it did not meet the inclusion and exclusion criteria. |
| Activities of α-asarone in various animal seizure models and in biochemical assays might be essentially accounted for by antioxidant properties | 2010 | Pages, N. and Maurois, P. and Delplanque, B. and Bac, P. and Stables, J.P. and Tamariz, J. and Chamorro, G. and Vamecq, J. | Excluded | The article was excluded based on title and abstract screening as it did not meet the inclusion and exclusion criteria. |
| Reduction of cerebral oxidative stress following environmental enrichment in mice with alzheimer-like pathology | 2010 | Herring, A. and Blome, M. and Ambrée, O. and Sachser, N. and Paulus, W. and Keyvani, K. | Excluded | The article was excluded based on title and abstract screening as it did not meet the inclusion and exclusion criteria. |
| Immune cell inflammatory cytokine responses differ between central and systemic compartments in response to acute exercise in mice | 2012 | Pervaiz, N. and Hoffman-Goetz, L. | Excluded | The article was excluded based on title and abstract screening as it did not meet the inclusion and exclusion criteria. |
| Low-density lipoprotein receptor-related protein 1: A physiological Aβ homeostatic mechanism with multiple therapeutic opportunities | 2012 | Sagare, A.P. and Deane, R. and Zlokovic, B.V. | Excluded | The article was excluded based on title and abstract screening as it did not meet the inclusion and exclusion criteria. |
| Antistress activity of ethanolic extract of asparagus racemosus willd roots in mice | 2012 | Joshi, T. and Sah, S.P. and Singh, A. | Excluded | The article was excluded based on title and abstract screening as it did not meet the inclusion and exclusion criteria. |
| Regulation of neuron mitochondrial biogenesis and relevance to brain health | 2010 | Onyango, I.G. and Lu, J. and Rodova, M. and Lezi, E. and Crafter, A.B. and Swerdlow, R.H. | Excluded | The article was excluded based on title and abstract screening as it did not meet the inclusion and exclusion criteria. |
| Low glial angiotensinogen improves body habitus, diastolic function, and exercise tolerance in aging male rats | 2012 | Groban, L. and Wang, H. and MacHado, F.S.M. and Trask, A.J. and Kritchevsky, S.B. and Ferrario, C.M. and Diz, D.I. | Excluded | The article was excluded based on title and abstract screening as it did not meet the inclusion and exclusion criteria. |
| High-intensity physical exercise disrupts implicit memory in mice: Involvement of the striatal glutathione antioxidant system and intracellular signaling | 2010 | Aguiar, A.S. and Boemer, G. and Rial, D. and Cordova, F.M. and Mancini, G. and Walz, R. and de Bem, A.F. and Latini, A. and Leal, R.B. and Pinho, R.A. and Prediger, R.D.S. | Excluded | The article was excluded based on title and abstract screening as it did not meet the inclusion and exclusion criteria. |
| Neurophysiological and epigenetic effects of physical exercise on the aging process | 2011 | Kaliman, P. and Párrizas, M. and Lalanza, J.F. and Camins, A. and Escorihuela, R.M. and Pallàs, M. | Excluded | The article was excluded based on title and abstract screening as it did not meet the inclusion and exclusion criteria. |
| Protective effect of melatonin on the oxidative stress caused by diabetes and forced swimming exercise on rat brain tissue | 2012 | Biçer, M. | Excluded | The article was excluded based on title and abstract screening as it did not meet the inclusion and exclusion criteria. |
| Brain in movement: The role of physical exercise in parkinson's disease | 2010 | Aguiar, A.S. and Prediger, R.D.S. | Excluded | The article was excluded based on title and abstract screening as it did not meet the inclusion and exclusion criteria. |
| Nutrition and the Brain | 2010 | Morley, J.E. | Excluded | The article was excluded based on title and abstract screening as it did not meet the inclusion and exclusion criteria. |
| Physical exercise reverses glutamate uptake and oxidative stress effects of chronic homocysteine administration in the rat | 2012 | Da Cunha, M.J. and Da Cunha, A.A. and Ferreira, A.G.K. and Machado, F.R. and Schmitz, F. and Lima, D.D. and Delwing, D. and Mussulini, B.H.M. and Wofchuk, S. and Netto, C.A. and Wyse, A.T.S. | Excluded | The article was excluded based on title and abstract screening as it did not meet the inclusion and exclusion criteria. |
| Molecular distinction between physiological and pathological cardiac hypertrophy: Experimental findings and therapeutic strategies | 2010 | Bernardo, B.C. and Weeks, K.L. and Pretorius, L. and McMullen, J.R. | Excluded | The article was excluded based on title and abstract screening as it did not meet the inclusion and exclusion criteria. |
| Rho-kinase inhibition: A novel therapeutic target for the treatment of cardiovascular diseases | 2010 | Dong, M. and Yan, B.P. and Liao, J.K. and Lam, Y.-Y. and Yip, G.W.K. and Yu, C.-M. | Excluded | The article was excluded based on title and abstract screening as it did not meet the inclusion and exclusion criteria. |
| The combination of exercise training and α-lipoic acid treatment has therapeutic effects on the pathogenic phenotypes of Alzheimer's disease in NSE/APPsw-transgenic mice | 2010 | Cho, J.Y. and Um, H.S. and Kang, E.B. and Cho, I.H. and Kim, C.H. and Cho, J.S. and Hwang, D.Y. | Excluded | The article was excluded based on title and abstract screening as it did not meet the inclusion and exclusion criteria. |
| Impaired adaptive cellular responses to oxidative stress and the pathogenesis of Alzheimer's disease | 2011 | Texel, S.J. and Mattson, M.P. | Excluded | The article was excluded based on title and abstract screening as it did not meet the inclusion and exclusion criteria. |
| Exercise during pregnancy mitigates Alzheimer-like pathology in mouse offspring | 2012 | Herring, A. and Donath, A. and Yarmolenko, M. and Uslar, E. and Conzen, C. and Kanakis, D. and Bosma, C. and Worm, K. and Paulus, W. and Keyvani, K. | Excluded | The article was excluded based on title and abstract screening as it did not meet the inclusion and exclusion criteria. |
| Biogerontology in Italy | 2011 | Odetti, P. and Bergamini, E. | Excluded | The article was excluded based on title and abstract screening as it did not meet the inclusion and exclusion criteria. |
| Is physical activity beneficial for recovery in temporal lobe epilepsy? Evidences from animal studies | 2009 | Arida, R.M. and Scorza, F.A. and Scorza, C.A. and Cavalheiro, E.A. | Excluded | The article was excluded based on title and abstract screening as it did not meet the inclusion and exclusion criteria. |
| Peripheral immune system and neuroimmune communication impairment in a mouse model of Alzheimer's disease | 2012 | Giménez-Llort, L. and Maté, I. and Manassra, R. and Vida, C. and De la Fuente, M. | Excluded | The article was excluded based on title and abstract screening as it did not meet the inclusion and exclusion criteria. |
| Regulation of central angiotensin type 1 receptors and sympathetic outflow in heart failure | 2009 | Zucker, I.H. and Schultz, H.D. and Patel, K.P. and Wang, W. and Gao, L. | Excluded | The article was excluded based on title and abstract screening as it did not meet the inclusion and exclusion criteria. |
| Gender-specific neuroimmunoendocrine response to treadmill exercise in 3xTg-AD mice | 2010 | Giménez-Llort, L. and García, Y. and Buccieri, K. and Revilla, S. and Suol, C. and Cristofol, R. and Sanfeliu, C. | Excluded | The article was excluded based on title and abstract screening as it did not meet the inclusion and exclusion criteria. |
| Mild Exercise Differently Affects Proteostasis and Oxidative Stress on Motor Areas During Neurodegeneration: A Comparative Study of Three Treadmill Running Protocols. | 2019 | Melo KP and Silva CM and Almeida MF and Chaves RS and Marcourakis T and Cardoso SM and Demasi M and Netto LES and Ferrari MFR | Included |  |
| Moderate treadmill exercise prevents oxidative stress-induced anxiety-like behavior in rats | 2010 | Salim, S. and Sarraj, N. and Taneja, M. and Saha, K. and Tejada-Simon, M.V. and Chugh, G. | Included |  |
| Effects of physical exercise on cognitive alterations and oxidative stress in an APP/PSN1 transgenic model of Alzheimer's disease | 2012 | Pareja-Galeano, H. and Brioche, T. and Sanchís-Gomar, F. and Escriva, C. and Dromant, M. and Go;mez-Cabrera, M.C. and Viña, J. | Excluded | The article was excluded based on title and abstract screening as it did not meet the inclusion and exclusion criteria. |
| Acute exercise and thioredoxin-1 in rat brain, and alpha-lipoic acid and thioredoxin-interacting protein response, in diabetes | 2010 | Lappalainen, Z. and Lappalainen, J. and Laaksonen, D.E. and Oksala, N.K.J. and Khanna, S. and Sen, C.K. and Atalay, M. | Excluded | The article was excluded based on title and abstract screening as it did not meet the inclusion and exclusion criteria. |
| Physical activity as an exogenous risk factor in motor neuron disease (MND): A review of the evidence | 2009 | Harwood, C.A. and McDermott, C.J. and Shaw, P.J. | Excluded | The article was excluded based on title and abstract screening as it did not meet the inclusion and exclusion criteria. |
| The role of PGC-1α on mitochondrial function and apoptotic susceptibility in muscle | 2009 | Adhihetty, P.J. and Uguccioni, G. and Leick, L. and Hidalgo, J. and Pilegaard, H. and Hood, D.A. | Excluded | The article was excluded based on title and abstract screening as it did not meet the inclusion and exclusion criteria. |
| Caloric restriction | 2011 | Speakman, J.R. and Mitchell, S.E. | Excluded | The article was excluded based on title and abstract screening as it did not meet the inclusion and exclusion criteria. |
| Regulation of neuronal oxidative and nitrosative stress by endogenous protective pathways and disease processes | 2011 | Hardingham, G.E. and Lipton, S.A. | Excluded | The article was excluded based on title and abstract screening as it did not meet the inclusion and exclusion criteria. |
| Hippocampal gene expression patterns underlying the enhancement of memory by running in aged mice | 2010 | Stranahan, A.M. and Lee, K. and Becker, K.G. and Zhang, Y. and Maudsley, S. and Martin, B. and Cutler, R.G. and Mattson, M.P. | Excluded | The article was excluded based on title and abstract screening as it did not meet the inclusion and exclusion criteria. |
| Bedside-to-bench conference: Research agenda for idiopathic fatigue and aging | 2010 | Alexander, N.B. and Taffet, G.E. and Horne, F.M. and Eldadah, B.A. and Ferrucci, L. and Nayfield, S. and Studenski, S. | Excluded | The article was excluded based on title and abstract screening as it did not meet the inclusion and exclusion criteria. |
| Dietary and behavioral interventions protect against age related activation of caspase cascades in the canine brain | 2011 | Snigdha, S. and Berchtold, N. and Astarita, G. and Saing, T. and Piomelli, D. and Cotman, C.W. | Excluded | The article was excluded based on title and abstract screening as it did not meet the inclusion and exclusion criteria. |
| The pattern of brain-derived neurotrophic factor gene expression in the hippocampus of diabetic rats | 2010 | Salehi, I. and Farajnia, S. and Mohammadi, M. and Ghannad, M.S. | Excluded | The article was excluded based on title and abstract screening as it did not meet the inclusion and exclusion criteria. |
| 182nd ENMC International Workshop: RYR1-related myopathies, 15-17th April 2011, Naarden, The Netherlands | 2012 | Jungbluth, H. and Dowling, J.J. and Ferreiro, A. and Muntoni, F. | Excluded | The article was excluded based on title and abstract screening as it did not meet the inclusion and exclusion criteria. |
| From clinic to bench: Physical therapy, exercise and Parkinson's disease | 2012 | Aguiar, A.S. and Prediger, R.D.S. | Excluded | The article was excluded based on title and abstract screening as it did not meet the inclusion and exclusion criteria. |
| Modification by vitamin E and exercise of oxidative stress in regions of aging rat brain: Studies on superoxide dismutase isoenzymes and protein oxidation status | 2006 | Jolitha, A.B. and Subramanyam, M.V.V. and Asha Devi, S. | Included |  |
| Additive anticonvulsant effects of creatine supplementation and physical exercise against pentylenetetrazol-induced seizures | 2009 | Rambo, L.M. and Ribeiro, L.R. and Oliveira, M.S. and Furian, A.F. and Lima, F.D. and Souza, M.A. and Silva, L.F.A. and Retamoso, L.T. and Corte, C.L.D. and Puntel, G.O. and de Avila, D.S. and Soares, F.A.A. and Fighera, M.R. and Mello, C.F. and Royes, L.F.F. | Excluded | The article was excluded based on title and abstract screening as it did not meet the inclusion and exclusion criteria. |
| Healthy brain and well-being: Sedentary lifestyle can impact cognitive ability adversely | 2011 | Asha Devi, S. and Manjula, K.R. and Shankaranarayana Rao, B.S. | Excluded | The article was excluded based on title and abstract screening as it did not meet the inclusion and exclusion criteria. |
| Possible role of no modulators in protective effect of trazodone and citalopram (antidepressants) in acute immobilization stress in mice | 2010 | Kumar, A. and Garg, R. and Gaur, V. and Kumar, P. | Excluded | The article was excluded based on title and abstract screening as it did not meet the inclusion and exclusion criteria. |
| The effect of regular training with vitamin E supplementation on the thioredoxine system in rats | 2010 | Metin, G. and Kucur, M. and Işman, F. and Altan, M. and Mengi, M. and Çakar, L. and Gümüştaş, K. and Zengin, E. | Excluded | The article was excluded based on title and abstract screening as it did not meet the inclusion and exclusion criteria. |
| Physical therapy, exercise and Parkinson's disease | 2012 | Aguiar, A.S. and Prediger, R.D.S. | Excluded | The article was excluded based on title and abstract screening as it did not meet the inclusion and exclusion criteria. |
| Beneficial effects of an innovative exercise model on motor and oxidative disorders induced by haloperidol in rats | 2011 | Teixeira, A. and Müller, L.G. and Reckziegel, P. and Boufleur, N. and Pase, C.S. and Villarinho, J.G. and Fachinetto, R. and Ferreira, J. and Rocha, J.B.T. and Bürger, M.E. | Excluded | The article was excluded based on title and abstract screening as it did not meet the inclusion and exclusion criteria. |
| Effect of St. John's Wort (Hypericum perforatum) treatment on restraint stress-induced behavioral and biochemical alteration in mice | 2010 | Kumar, A. and Garg, R. and Prakash, A.K. | Excluded | The article was excluded based on title and abstract screening as it did not meet the inclusion and exclusion criteria. |
| Carbohydrate-energy restriction may protect the rat brain against oxidative damage and improve physical performance | 2003 | De Oliveira, S.L. and Diniz, D.B. and Amaya-Farfan, J. | Excluded | The article was excluded based on title and abstract screening as it did not meet the inclusion and exclusion criteria. |
| Oxidative stress in rats exercised at different intensities | 2009 | de Araújo, M.B. and Voltarelli, F.A. and Contarteze, R.V.L. and de Barros Manchado-Gobatto, F. and de Mello, M.A.R. | Included |  |
| The influence of severe long-term exercise on the mouse hippocampus | 2002 | Sumitani, K. and Miyamoto, O. and Yamagami, S. and Okada, Y. and Itano, T. and Murakami, T. and Negi, T. | Excluded | The article was excluded based on title and abstract screening as it did not meet the inclusion and exclusion criteria. |
| Mechanistic insights into diabetes mellitus and oxidative stress | 2007 | Maiese, K. and Chong, Z.Z. and Shang, Y.C. | Excluded | The article was excluded based on title and abstract screening as it did not meet the inclusion and exclusion criteria. |
| Oxidative stress induced by intense and exhaustive exercise impairs murine cognitive function | 2007 | Rosa, E.F. and Takahashi, S. and Aboulafia, J. and Nouailhetas, V.L.A. and Oliveira, M.G.M. | Included |  |
| Effects of vitamin C and rutin on metabolism of free radicals in tissue of exhaustive swimming mice | 2005 | Gu, H.-Y. and Zhang, Y.-Y. and Zhai, J. and Sun, L.-Y. and Wang, T. and Bai, S.-Y. | Excluded | The article was excluded based on title and abstract screening as it did not meet the inclusion and exclusion criteria. |
| Effect of physical exercise on markers of acute cardiotoxicity induced by d-amphetamine in an animal model | 2006 | Ferreira, M.T. and Ferreira, R. and Carvalho, F. and Duarte, J.A. | Excluded | The article was excluded based on title and abstract screening as it did not meet the inclusion and exclusion criteria. |
| Caloric restriction and brain function | 2008 | Gillette-Guyonnet, S. and Vellas, B. | Excluded | The article was excluded based on title and abstract screening as it did not meet the inclusion and exclusion criteria. |
| Mitochondrial biogenesis and healthy aging | 2008 | López-Lluch, G. and Irusta, P.M. and Navas, P. and de Cabo, R. | Excluded | The article was excluded based on title and abstract screening as it did not meet the inclusion and exclusion criteria. |
| License to run: Exercise impacts functional plasticity in the intact and injured central nervous system by using neurotrophins | 2005 | Vaynman, S. and Gomez-Pinilla, F. | Excluded | The article was excluded based on title and abstract screening as it did not meet the inclusion and exclusion criteria. |
| Exercise and brain health - Implications for multiple sclerosis: Part 1 - Neuronal growth factors | 2008 | White, L.J. and Castellano, V. | Excluded | The article was excluded based on title and abstract screening as it did not meet the inclusion and exclusion criteria. |
| Interaction between alcohol and exercise: Physiological and haematological implications | 2005 | El-Sayed, M.S. and Ali, N. and Ali, Z.E.-S. | Excluded | The article was excluded based on title and abstract screening as it did not meet the inclusion and exclusion criteria. |
| Distribution of coenzyme Q homologues in brain | 2002 | Albano, C.B. and Muralikrishnan, D. and Ebadi, M. | Excluded | The article was excluded based on title and abstract screening as it did not meet the inclusion and exclusion criteria. |
| Docosahexaenoic acid abundance in the brain: A biodevice to combat oxidative stress | 2002 | Yavin, E. and Brand, A. and Green, P. | Excluded | The article was excluded based on title and abstract screening as it did not meet the inclusion and exclusion criteria. |
| Aging and biomedicine 2005: Where should we go from here? | 2005 | Barton, M. | Excluded | The article was excluded based on title and abstract screening as it did not meet the inclusion and exclusion criteria. |
| Oxidative Stress Is a Central Target for Physical Exercise Neuroprotection Against Pathological Brain Aging | 2015 | Garciá-Mesa, Y. and Colie, S. and Corpas, R. and Cristòfol, R. and Comellas, F. and Nebreda, A.R. and Gim'Nez-Llort, L. and Sanfeliu, C. | Included |  |
| Administration of melatonin and related indoles prevents exercise-induced cellular oxidative changes in rats | 1997 | Hara, M. and Iigo, M. and Ohtani-Kaneko, R. and Nakamura, N. and Suzuki, T. and Reiter, R.J. and Hirata, K. | Excluded | The article was excluded based on title and abstract screening as it did not meet the inclusion and exclusion criteria. |
| Is metabolic syndrome X a disorder of the brain? | 2008 | Das, U.N. | Excluded | The article was excluded based on title and abstract screening as it did not meet the inclusion and exclusion criteria. |
| Oxygen consumption and usage during physical exercise: The balance between oxidative stress and ROS-dependent adaptive signaling | 2013 | Radak, Z. and Zhao, Z. and Koltai, E. and Ohno, H. and Atalay, M. | Included |  |
| Protective action of a hexane crude extract of Pterodon emarginatus fruits against oxidative and nitrosative stress induced by acute exercise in rats | 2005 | Paula, F.B.A. and Gouvêa, C.M.C.P. and Alfredo, P.P. and Salgado, I. | Excluded | The article was excluded based on title and abstract screening as it did not meet the inclusion and exclusion criteria. |
| The effects of vitamin C supplementation on oxidative stress and antioxidant content in the brains of chronically exercised rats | 2005 | Coşkun, Ş. and Gönül, B. and Güzel, N.A. and Balabanli, B. | Excluded | The article was excluded based on title and abstract screening as it did not meet the inclusion and exclusion criteria. |
| The influence of diet and physical activity on brain repair and neurosurgical outcome | 2008 | Gomez-Pinilla, F. and Kostenkova, K. | Excluded | The article was excluded based on title and abstract screening as it did not meet the inclusion and exclusion criteria. |
| Physical Exercise Attenuates Oxidative Stress and Morphofunctional Cerebellar Damages Induced by the Ethanol Binge Drinking Paradigm from Adolescence to Adulthood in Rats | 2019 | Lamarão-Vieira, K. and Pamplona-Santos, D. and Nascimento, P.C. and Corrêa, M.G. and Bittencourt, L.O. and Dos Santos, S.M. and Cartágenes, S.C. and Fernandes, L.M.P. and Monteiro, M.C. and Maia, C.S.F. and Lima, R.R. | Included |  |
| Physical exercise improves brain cortex and cerebellum mitochondrial bioenergetics and alters apoptotic, dynamic and auto(mito)phagy markers | 2015 | Marques-Aleixo, I. and Santos-Alves, E. and Balça, M.M. and Rizo-Roca, D. and Moreira, P.I. and Oliveira, P.J. and Magalhães, J. and Ascensão, A. | Included |  |
| Age peculiarities of changes of superoxide dismutase, glutathione peroxidase and catalase activity in the brain of rats during immobilized stress | 2004 | Suvorova, I.N. and Davydov, V.V. | Excluded | The article was excluded based on title and abstract screening as it did not meet the inclusion and exclusion criteria. |
| Physical exercise mitigates doxorubicin-induced brain cortex and cerebellum mitochondrial alterations and cellular quality control signaling | 2016 | Marques-Aleixo, I. and Santos-Alves, E. and Balça, M.M. and Moreira, P.I. and Oliveira, P.J. and Magalhães, J. and Ascensão, A. | Included |  |
| Visfatin: Structure, function and relation to diabetes mellitus and other dysfunctions | 2008 | Adeghate, E. | Excluded | The article was excluded based on title and abstract screening as it did not meet the inclusion and exclusion criteria. |
| Biological markers of age-related memory deficits: Treatment of senescent physiology | 2006 | Foster, T.C. | Excluded | The article was excluded based on title and abstract screening as it did not meet the inclusion and exclusion criteria. |
| Arterial pulse wave velocity, inflammatory markers, pathological GH and IGF states, cardiovascular and cerebrovascular disease | 2008 | Graham, M.R. and Evans, P. and Davies, B. and Baker, J.S. | Excluded | The article was excluded based on title and abstract screening as it did not meet the inclusion and exclusion criteria. |
| Brain mitochondrial dysfunction in aging: Conditions that improve survival, neurological performance and mitochondrial function | 2007 | Navarro, A. and Boveris, A. | Excluded | The article was excluded based on title and abstract screening as it did not meet the inclusion and exclusion criteria. |
| Intense exercise potentiates oxidative stress in striatum of reserpine-treated animals | 2009 | Teixeira, A.M. and Reckziegel, P. and Müller, L. and Pereira, R.P. and Roos, D.H. and Rocha, J.B.T. and Bürger, M.E. | Excluded | The article was excluded based on title and abstract screening as it did not meet the inclusion and exclusion criteria. |
| Oxidative stress and role of antioxidant and ω-3 essential fatty acid supplementation in schizophrenia | 2001 | Mahadik, S.P. and Evans, D. and Lal, H. | Excluded | The article was excluded based on title and abstract screening as it did not meet the inclusion and exclusion criteria. |
| Prior exercise protects against oxidative stress and motor deficit in a rat model of Parkinson's disease. | 2020 | Oliveira LOD and da Silva PIC and Filho RPR and Progênio RCS and de Oliveira VDPS and Silva RC and de Andrade MC and da Silva Freitas JJ and Kietzer KS | Included |  |
| Resistance exercise training attenuates alcohol-induced cardiac oxidative stress | 2006 | Chicco, A.J. and Mccarty, H. and Reed, A.H. and Story, R.R. and Westerlind, K.C. and Turner, R.T. and Hayward, R. | Excluded | The article was excluded based on title and abstract screening as it did not meet the inclusion and exclusion criteria. |
| Impaired exercise capacity, but unaltered mitochondrial respiration in skeletal or cardiac muscle of mice lacking cellular prion protein | 2005 | Nico, P.B.C. and Lobão-Soares, B. and Landemberger, M.C. and Marques Jr., W. and Tasca, C.I. and De Mello, C.F. and Walz, R. and Carlotti Jr., C.G. and Brentani, R.R. and Sakamoto, A.C. and Bianchin, M.M. | Excluded | The article was excluded based on title and abstract screening as it did not meet the inclusion and exclusion criteria. |
| The effect of exercise and nettle supplementation on oxidative stress markers in the rat brain | 2005 | Toldy, A. and Stadler, K. and Sasvári, M. and Jakus, J. and Jung, K.J. and Chung, H.Y. and Berkes, I. and Nyakas, C. and Radák, Z. | Excluded | The article was excluded based on title and abstract screening as it did not meet the inclusion and exclusion criteria. |
| ADP-Regulation of mitochondrial free radical production is different with complex I- or complex II-linked substrates: Implications for the exercise paradox and brain hypermetabolism | 1997 | Herrero, A. and Barja, G. | Excluded | The article was excluded based on title and abstract screening as it did not meet the inclusion and exclusion criteria. |
| The effect of n-acetylcysteine and deferoxamine on exercise-induced oxidative damage in striatum and hippocampus of mice | 2008 | Aguiar Jr., A.S. and Tuon, T. and Soares, F.S. and Da Rocha, L.G.C. and Silveira, P.C. and Pinho, R.A. | Excluded | The article was excluded based on title and abstract screening as it did not meet the inclusion and exclusion criteria. |
| Docosahexaenoic acid dietary supplementation enhances the effects of exercise on synaptic plasticity and cognition | 2008 | Wu, A. and Ying, Z. and Gomez-Pinilla, F. | Excluded | The article was excluded based on title and abstract screening as it did not meet the inclusion and exclusion criteria. |
| Systemic adaptation to oxidative challenge induced by regular exercise | 2008 | Radak, Z. and Chung, H.Y. and Goto, S. | Excluded | The article was excluded based on title and abstract screening as it did not meet the inclusion and exclusion criteria. |
| Intense exercise induces mitochondrial dysfunction in mice brain | 2008 | Aguiar Jr., A.S. and Tuon, T. and Pinho, C.A. and Silva, L.A. and Andreazza, A.C. and Kapczinski, F. and Quevedo, J. and Streck, E.L. and Pinho, R.A. | Excluded | The article was excluded based on title and abstract screening as it did not meet the inclusion and exclusion criteria. |
| One molecule, many derivatives: A never-ending interaction of melatonin with reactive oxygen and nitrogen species? | 2007 | Tan, D.-X. and Manchester, L.C. and Terron, M.P. and Flores, L.J. and Reiter, R.J. | Excluded | The article was excluded based on title and abstract screening as it did not meet the inclusion and exclusion criteria. |
| Neuronal calcium mishandling and the pathogenesis of Alzheimer's disease | 2008 | Bezprozvanny, I. and Mattson, M.P. | Excluded | The article was excluded based on title and abstract screening as it did not meet the inclusion and exclusion criteria. |
| Exercise training acts as a therapeutic strategy for reduction of the pathogenic phonetypes for Alzheimer's disease in an NSE/APPSw-transgenic model | 2008 | Um, H.S. and Kang, E.B. and Leem, Y.H. and Cho, I.H. and Yang, C.H. and Chae, K.R. and Hwang, D.Y. and Cho, J.Y. | Excluded | The article was excluded based on title and abstract screening as it did not meet the inclusion and exclusion criteria. |
| Protective effect of qinggong changchun dan on peroxidation injury induced by exercise in mice | 2005 | Cao, K. and Tong, J.-M. | Excluded | The article was excluded based on title and abstract screening as it did not meet the inclusion and exclusion criteria. |
| Protective effects of early exercise on neuroinflammation, and neurotoxicity associated by traumatic brain injury: a behavioral and neurochemical approach. | 2022 | Rafie F and Khaksari M and Amiresmaili S and Soltani Z and Pourranjbar M and Shirazpour S and Jafari E | Included |  |
| The harmful effect of exercise on reducing taurine concentration in the tissues of rats treated with CC14 administration | 2004 | Miyazaki, T. and Matsuzaki, Y. and Ikegami, T. and Miyakawa, S. and Doy, M. and Tanaka, N. and Bouscarel, B. | Excluded | The article was excluded based on title and abstract screening as it did not meet the inclusion and exclusion criteria. |
| Age peculiarities in changes of free radical oxidation processes in the brain of rats with hypothyroidism | 2007 | Ali, K.A. and Davydov, V.V. | Excluded | The article was excluded based on title and abstract screening as it did not meet the inclusion and exclusion criteria. |
| Novel mechanisms of sympathetic regulation in chronic heart failure | 2006 | Zucker, I.H. | Excluded | The article was excluded based on title and abstract screening as it did not meet the inclusion and exclusion criteria. |
| Prophylactic Activation of Neuroprotective Stress Response Pathways by Dietary and Behavioral Manipulations | 2004 | Mattson, M.P. and Duan, W. and Wan, R. and Guo, Z. | Excluded | The article was excluded based on title and abstract screening as it did not meet the inclusion and exclusion criteria. |
| Quercetin supplementation does not enhance cerebellar mitochondrial biogenesis and oxidative status in exercised rats | 2015 | Casuso, R.A. and Martínez-Amat, A. and Hita-Contreras, F. and Camiletti-Moirón, D. and Aranda, P. and Martínez-López, E. | Included |  |
| Ageing and neuronal vulnerability | 2006 | Mattson, M.P. and Magnus, T. | Excluded | The article was excluded based on title and abstract screening as it did not meet the inclusion and exclusion criteria. |
| Regional responses in antioxidant system to exercise training and dietary Vitamin E in aging rat brain | 2004 | Devi, S.Asha and Kiran, T.Ravi | Included |  |
| Coupling energy metabolism with a mechanism to support brain-derived neurotrophic factor-mediated synaptic plasticity | 2006 | Vaynman, S. and Ying, Z. and Wu, A. and Gomez-Pinilla, F. | Excluded | The article was excluded based on title and abstract screening as it did not meet the inclusion and exclusion criteria. |
| Effect of L-carnitine administration on the modulated rat brain protein concentration, acetylcholinesterase, Na+K+-ATPase and Mg2+-ATPase activities induced by forced swimming | 2008 | Tsakiris, T. and Angelogianni, P. and Tesseromatis, C. and Tsakiris, S. and Schulpis, K.H. | Excluded | The article was excluded based on title and abstract screening as it did not meet the inclusion and exclusion criteria. |
| Brain IRS2 signaling coordinates life span and nutrient homeostasis | 2007 | Taguchi, A. and Wartschow, L.M. and White, M.F. | Excluded | The article was excluded based on title and abstract screening as it did not meet the inclusion and exclusion criteria. |
| Regular exercise attenuates oxidative stress in aging rat tissues: A possible mechanism toward anti-aging medicine | 2007 | Goto, S. and Radák, Z. | Included |  |
| Editorial | 2007 | Di Matteo, V. and Esposito, E. and Di Giovanni, G. | Excluded | The article was excluded based on title and abstract screening as it did not meet the inclusion and exclusion criteria. |
| Hyperbaric oxygen as an adjuvant for athletes | 2005 | Ishii, Y. and Deie, M. and Adachi, N. and Yasunaga, Y. and Sharman, P. and Miyanaga, Y. and Ochi, M. | Excluded | The article was excluded based on title and abstract screening as it did not meet the inclusion and exclusion criteria. |
| Arterial desaturation during exercise in man: Implication for O 2 uptake and work capacity | 2003 | Nielsen, H.B. | Excluded | The article was excluded based on title and abstract screening as it did not meet the inclusion and exclusion criteria. |
| Induction of oxidative stress by restraint stress and corticosterone treatments in rats | 2009 | Zafir, A. and Banu, N. | Excluded | The article was excluded based on title and abstract screening as it did not meet the inclusion and exclusion criteria. |
| Exercise training prevents arterial baroreflex dysfunction in rats treated with central angiotensin II | 2007 | Pan, Y.-X. and Gao, L. and Wang, W.-Z. and Zheng, H. and Liu, D. and Patel, K.P. and Zucker, I.H. and Wang, W. | Excluded | The article was excluded based on title and abstract screening as it did not meet the inclusion and exclusion criteria. |
| Ranolazine: New Paradigm for Management of Myocardial Ischemia, Myocardial Dysfunction, and Arrhythmias | 2008 | Stone, P.H. | Excluded | The article was excluded based on title and abstract screening as it did not meet the inclusion and exclusion criteria. |
| Hormesis and exercise: How the cell copes with oxidative stress | 2008 | Ji, L.L. and Radak, Z. and Goto, S. | Excluded | The article was excluded based on title and abstract screening as it did not meet the inclusion and exclusion criteria. |
| The effect of exercise on hippocampal integrity: Review of recent research | 2005 | Kiraly, M.A. and Kiraly, S.J. | Excluded | The article was excluded based on title and abstract screening as it did not meet the inclusion and exclusion criteria. |
| Oxidative stress and ischemic injuries in heat stroke | 2007 | Chang, C.-K. and Chang, C.-P. and Liu, S.-Y. and Lin, M.-T. and Sharma H.S. and Laboratory of Cerebrovascular Res., Dept. of Surgical Sci., Division of Anaesthesiol. and Intens,... | Excluded | The article was excluded based on title and abstract screening as it did not meet the inclusion and exclusion criteria. |
| Anthracycline cardiotoxicity: From bench to bedside | 2008 | Gianni, L. and Herman, E.H. and Lipshultz, S.E. and Minotti, G. and Sarvazyan, N. and Sawyer, D.B. | Excluded | The article was excluded based on title and abstract screening as it did not meet the inclusion and exclusion criteria. |
| Adaptation to exercise-induced oxidative stress: From muscle to brain | 2001 | Radak, Z. and Taylor, A.W. and Ohno, H. and Goto, S. | Excluded | The article was excluded based on title and abstract screening as it did not meet the inclusion and exclusion criteria. |
| Regular exercise improves cognitive function and decreases oxidative damage in rat brain | 2001 | Radák, Zsolt and Kaneko, Takao and Tahara, Shoichi and Nakamoto, Hideko and Pucsok, Jozsef and Sasvári, Mária and Nyakas, Csaba and Goto, Sataro | Included |  |
| Regular exercise prevents oxidative stress in the brain of hyperphenylalaninemic rats | 2011 | Mazzola, P.N. and Terra, M. and Rosa, A.P. and Mescka, C.P. and Moraes, T.B. and Piccoli, B. and Jacques, C.E. and Dalazen, G. and Cortes, M.X. and Coelho, J. and Dutra-Filho, C.S. | Included |  |
| Levodopa therapy from the neuroprotection viewpoint: From a clinical outlook | 2005 | Kondo, T. | Excluded | The article was excluded based on title and abstract screening as it did not meet the inclusion and exclusion criteria. |
| The mitochondrial energy transduction system and the aging process | 2007 | Navarro, A. and Boveris, A. | Excluded | The article was excluded based on title and abstract screening as it did not meet the inclusion and exclusion criteria. |
| Prevention of Alzheimer's disease: Putative nutritive factors | 2007 | Asada, T. | Excluded | The article was excluded based on title and abstract screening as it did not meet the inclusion and exclusion criteria. |
| Diabetes impairs exercise training-associated thioredoxin response and glutathione status in rat brain | 2009 | Lappalainen, Z. and Lappalainen, J. and Oksala, N.K.J. and Laaksonen, D.E. and Khanna, S. and Sen, C.K. and Atalay, M. | Excluded | The article was excluded based on title and abstract screening as it did not meet the inclusion and exclusion criteria. |
| Brain mitochondrial dysfunction in aging | 2008 | Boveris, A. and Navarro, A. | Excluded | The article was excluded based on title and abstract screening as it did not meet the inclusion and exclusion criteria. |
| Long term dietary restriction ameliorates swimming exercise-induced oxidative stress in brain and lung of middle-aged rat | 2009 | Aydin, C. and Sonat, F. and Sahin, S.K. and Cangul, I.T. and Ozkaya, G. | Excluded | The article was excluded based on title and abstract screening as it did not meet the inclusion and exclusion criteria. |
| Acute exhaustive exercise does not alter lipid peroxidation levels and antioxidant enzyme activities in rat hippocampus, prefrontal cortex and striatum | 2006 | Acikgoz, O. and Aksu, I. and Topcu, A. and Kayatekin, B.M. | Excluded | The article was excluded based on title and abstract screening as it did not meet the inclusion and exclusion criteria. |
| Effect of endurance exercise on the tissue 8-hydroxy-deoxyguanosine content in dogs | 1997 | Okamura, K. and Doi, T. and Sakurai, M. and Hamada, K. and Yoshioka, Y. and Sumida, S. and Sugawa-Katayama, Y. | Excluded | The article was excluded based on title and abstract screening as it did not meet the inclusion and exclusion criteria. |
| Single bout of exercise eliminates the immobilization-caused oxidative stress in rat brain | 1999 | Radak, Zsolt and Kaneko, Takao and Nyakas, Csaba and Goto, Sataro | Included |  |
| Part-B: Preface | 2001 | Kovacic, P. | Excluded | The article was excluded based on title and abstract screening as it did not meet the inclusion and exclusion criteria. |
| Meeting Report. Central and peripheral mechanisms of aging and frailty: A report on the 8th longevity consortium symposium, Santa Fe, New Mexico, May 16-18, 2007 | 2007 | Kahn, A.J. and Austad, S. and Pahor, M. and Harris, T. and Goodpaster, B. and Leewenburgh, C. and Wolkow, C. and Raz, N. and Klann, E. and Kondratov, R. and Stone, K. and Antebi, A. and Gaglia, M. and Tower, J. and Yuan, R. | Excluded | The article was excluded based on title and abstract screening as it did not meet the inclusion and exclusion criteria. |
| Pathophysiological factors underlying heatstroke | 2006 | Yan, Y.-E. and Zhao, Y.-Q. and Wang, H. and Fan, M. | Excluded | The article was excluded based on title and abstract screening as it did not meet the inclusion and exclusion criteria. |
| Effects of exercise on brain function: Role of free radicals | 2007 | Radak, Z. and Kumagai, S. and Taylor, A.W. and Naito, H. and Goto, S. | Excluded | The article was excluded based on title and abstract screening as it did not meet the inclusion and exclusion criteria. |
| Single bout of exercise eliminates the immobilization-induced oxidative stress in rat brain | 2001 | Radak, Z. and Sasvari, M. and Nyakas, C. and Kaneko, T. and Tahara, S. and Ohno, H. and Goto, S. | Included |  |
| Myocardial antioxidant status and oxidative stress after combined action of exercise training and ethanol in two different age groups of male albino rats | 2007 | Pushpalatha, K. and Nishanth, K. and Sathyavelu Reddy, K. | Excluded | The article was excluded based on title and abstract screening as it did not meet the inclusion and exclusion criteria. |
| L-cysteine's effect on modulated rat brain enzymes with forced swimming | 2009 | Tsakiris, T. and Angelogianni, P. and Tesseromatis, C. and Tsakiris, S. and Schulpis, K.H. | Excluded | The article was excluded based on title and abstract screening as it did not meet the inclusion and exclusion criteria. |
| Dexrazoxane: A review of its use for cardioprotection during anthracycline chemotherapy | 2005 | Cvetković, R.S. and Scott, L.J. | Excluded | The article was excluded based on title and abstract screening as it did not meet the inclusion and exclusion criteria. |
| Animal models and possible human application of immunological restoration in the elderly | 2002 | Hirokawa, K. and Utsuyama, M. | Excluded | The article was excluded based on title and abstract screening as it did not meet the inclusion and exclusion criteria. |
| Effect of exercise training on ethanol-induced oxidative damage in aged rats | 2009 | Mallikarjuna, K. and Nishanth, K. and Hou, C.-W. and Kuo, C.-H. and Sathyavelu Reddy, K. | Excluded | The article was excluded based on title and abstract screening as it did not meet the inclusion and exclusion criteria. |
| Protective response of methanolic extract of Ocimum sanctum, Withania somnifera and Zingiber officinalis on swimming-induced oxidative damage on cardiac, skeletal and brain tissues in male rat: A duration dependent study | 2006 | Misra, D.S. and Maiti, R. and Mallick, C. and Ghosh, D. | Excluded | The article was excluded based on title and abstract screening as it did not meet the inclusion and exclusion criteria. |
| Age-related responses of the rat cerebral cortex: Influence of vitamin E and exercise on the cholinergic system | 2009 | Jolitha, A.B. and Subramanyam, M.V.V. and Asha Devi, S. | Excluded | The article was excluded based on title and abstract screening as it did not meet the inclusion and exclusion criteria. |
| Tissue changes in glutathione metabolism and lipid peroxidation induced by swimming are partially prevented by melatonin | 1996 | Hara, M. and Abe, M. and Suzuki, T. and Reiter, R.J. | Excluded | The article was excluded based on title and abstract screening as it did not meet the inclusion and exclusion criteria. |
| Effects of Resistance Training on Oxidative Stress Markers and Muscle Damage in Spinal Cord Injured Rats | 2022 | Barros, ND and Aidar, FJ and Marcal, AC and Santos, JL and de Souza, RF and Menezes, JL and Gomes, MZ and de Matos, DG and Neves, EB and Carneiro, ALG and de Almeida-Neto, PF and Cabral, BGDT and Neto, RVB and Knechtle, B and Clemente, FM and Camargo, EA | Excluded | The article was excluded based on title and abstract screening as it did not meet the inclusion and exclusion criteria. |
| Exercise training decreases DNA damage and increases DNA repair and resistance against oxidative stress of proteins in aged rat skeletal muscle | 2002 | Radák, Z and Naito, H and Kaneko, T and Tahara, S and Nakamoto, H and Takahashi, R and Cardozo-Pelaez, F and Goto, S | Excluded | The article was excluded based on title and abstract screening as it did not meet the inclusion and exclusion criteria. |
| Neuroprotective effect of nerolidol in traumatic brain injury associated behavioural comorbidities in rats | 2021 | Kaur, A and Jaiswal, G and Brar, J and Kumar, P | Excluded | The article was excluded based on title and abstract screening as it did not meet the inclusion and exclusion criteria. |
| Effects of electrotactic exercise and antioxidant EUK-134 on oxidative stress relief in <i>Caenorhabditis elegans</i> | 2021 | Pham, TTH and Huang, WY and Chen, CS and Chiu, WT and Chuang, HS | Excluded | The article was excluded based on title and abstract screening as it did not meet the inclusion and exclusion criteria. |
| Stress-induced multiple organ damage in rats is ameliorated by the antioxidant and anxiolytic effects of regular exercise | 2010 | Çakir, B. and Kasimay, O. and Kolgazi, M. and Ersoy, Y. and Ercan, F. and Yeǧen, B.C. | Included |  |
| Exercise therapy to prevent and treat Alzheimer's disease | 2023 | Pahlavani, HA | Excluded | The article was excluded based on title and abstract screening as it did not meet the inclusion and exclusion criteria. |
| Kolaviron, a <i>Garcinia kola</i> Biflavonoid Complex, Protects Against Ischemia/Reperfusion Injury: Pertinent Mechanistic Insights from Biochemical and Physical Evaluations in Rat Brain | 2015 | Akinmoladun, AC and Akinrinola, BL and Olaleye, MT and Farombi, EO | Excluded | The article was excluded based on title and abstract screening as it did not meet the inclusion and exclusion criteria. |
| A systematic review of exercise modalities that reduce pro-inflammatory cytokines in humans and animals' models with mild cognitive impairment or dementia | 2023 | Ayari, S and Abellard, A and Carayol, M and Guedj, E and Gavarry, O | Excluded | The article was excluded based on title and abstract screening as it did not meet the inclusion and exclusion criteria. |
| Genetic damage in multiple organs of acutely exercised rats | 2010 | Pozzi, R and Rosa, JC and Eguchi, R and do Nascimento, CMO and Oyama, LM and Aguiar, O and Chaves, MD and Ribeiro, DA | Excluded | The article was excluded based on title and abstract screening as it did not meet the inclusion and exclusion criteria. |
| Maternal Deprivation Induces Memory Deficits That Are Reduced by One Aerobic Exercise Shot Performed after the Learning Session | 2019 | Sosa, PM and Neves, BHS and Carrazoni, GS and Gomes, GM and Del Rosso, G and Ramborger, BP and Rohers, R and Mello-Carpes, PB | Excluded | The article was excluded based on title and abstract screening as it did not meet the inclusion and exclusion criteria. |
| Animal models of resistance exercise and their application to neuroscience research | 2016 | Strickland, JC and Smith, MA | Excluded | The article was excluded based on title and abstract screening as it did not meet the inclusion and exclusion criteria. |
| Physical Exercise Alleviates Health Defects, Symptoms, and Biomarkers in Schizophrenia Spectrum Disorder | 2015 | Archer, T and Kostrzewa, RM | Excluded | The article was excluded based on title and abstract screening as it did not meet the inclusion and exclusion criteria. |
| Protective effect of metformin on D-galactose-induced aging model in mice | 2018 | Fatemi, I and Khaluoi, A and Kaeidi, A and Shamsizadeh, A and Heydari, S and Allahtavakoli, M | Excluded | The article was excluded based on title and abstract screening as it did not meet the inclusion and exclusion criteria. |
| Aerobic exercise improves ejaculatory behaviors and complements dapoxetine treatment by upregulating the BDNF-5-HT duo: a pilot study in rats | 2023 | Huang, YY and Peng, DW and Liu, QS and Jiang, H and Zhang, XS | Excluded | The article was excluded based on title and abstract screening as it did not meet the inclusion and exclusion criteria. |
| Neuroprotection Afforded by an Enriched Mediterranean-like Diet Is Modified by Exercise in a Rat Male Model of Cerebral Ischemia | 2024 | Romaus-Sanjurjo, D and Castañón-Apilánez, M and López-Arias, E and Custodia, A and Martin-Martín, C and Ouro, A and López-Cancio, E and Sobrino, T | Excluded | The article was excluded based on title and abstract screening as it did not meet the inclusion and exclusion criteria. |
| The effect of two types of diet on apoptosis indexes, lipid profile and histopathological outcome in acute kidney injury during exercise | 2022 | Sabet, N and Soltani, Z and Khaksari, M and Iranpour, M and Afshar, RM and Mehdiabadi, FM and Raji-Amirhasani, A | Excluded | The article was excluded based on title and abstract screening as it did not meet the inclusion and exclusion criteria. |
| Changes in markers of brain serotonin activity in response to chronic exercise in senior men | 2014 | Melancon, MO and Lorrain, D and Dionne, IJ | Excluded | The article was excluded based on title and abstract screening as it did not meet the inclusion and exclusion criteria. |
| Heat treatment and protective potentials of luteolin-7-O-glucoside against cisplatin genotoxic and cytotoxic effects | 2020 | Maatouk, M and Abed, B and Bouhlel, I and Krifa, M and Khlifi, R and Ioannou, I and Ghedira, K and Ghedira, LC | Excluded | The article was excluded based on title and abstract screening as it did not meet the inclusion and exclusion criteria. |
| Nutritional interventions to improve neurophysiological impairments following traumatic brain injury: A systematic review | 2021 | McGeown, JP and Hume, PA and Theadom, A and Quarrie, KL and Borotkanics, R | Excluded | The article was excluded based on title and abstract screening as it did not meet the inclusion and exclusion criteria. |
| Anti-oxidative effects produced by environmental enrichment in the hippocampus and cerebral cortex of male and female rats | 2015 | Mármol, F and Rodríguez, CA and Sánchez, J and Chamizo, VD | Excluded | The article was excluded based on title and abstract screening as it did not meet the inclusion and exclusion criteria. |
| The effects of prolonged stress exposure on the brain of rats and insights to understand the impact of work-related stress on caregivers | 2023 | Pujo, JM and Fitriani, DY and Ben Saad, H and Ghariani, M and Dghim, A and Mellouli, M and Burin, A and Mutricy, R and Houcke, S and Roujansky, A and Mansyur, M and Nkontcho, F and de Toffol, B and Ben Amara, I and Kallel, H | Excluded | The article was excluded based on title and abstract screening as it did not meet the inclusion and exclusion criteria. |
| Swimming exercise enhances the hippocampal antioxidant status of female Wistar rats | 2015 | Stone, V and Kudo, KY and Marcelino, TB and August, PM and Matte, C | Excluded | The article was excluded based on title and abstract screening as it did not meet the inclusion and exclusion criteria. |
| Rapid Cellular Genesis and Apoptosis: Effects of Exercise in the Adult Rat | 2011 | Kerr, AL and Swain, RA | Excluded | The article was excluded based on title and abstract screening as it did not meet the inclusion and exclusion criteria. |
| Physical Training Moderates Blood-Brain-Barrier Disruption and Improves Cognitive Dysfunction Related to Transient Brain Ischemia in Rats | 2019 | Shamsaei, N and Abdi, H and Moradi, F | Excluded | The article was excluded based on title and abstract screening as it did not meet the inclusion and exclusion criteria. |
| Effects of Resistance Training and <i>Bowdichia virgilioides</i> Hydroethanolic Extract on Oxidative Stress Markers in Rats Submitted to Peripheral Nerve Injury | 2020 | Costa, LS and Aidar, FJ and de Matos, DG and de Oliveira, JU and dos Santos, JL and Almeida-Neto, PFD and de Souza, RF and Pereira, DD and Garrido, ND and Nunes-Silva, A and Marçal, AC and Estevam, CD and Cabral, BGDT and Reis, VM and Teixeira, MM | Excluded | The article was excluded based on title and abstract screening as it did not meet the inclusion and exclusion criteria. |
| Anti-Oxidative Effects of Rooibos Tea (Aspalathus linearis) on Immobilization-Induced Oxidative Stress in Rat Brain | 2014 | Hong, IS and Lee, HY and Kim, HP | Excluded | The article was excluded based on title and abstract screening as it did not meet the inclusion and exclusion criteria. |
| Intermittent Hypoxic Conditioning Alleviates Post-Traumatic Stress Disorder-Induced Damage and Dysfunction of Rat Visceral Organs and Brain | 2020 | Manukhina, EB and Tseilikman, VE and Karpenko, MN and Pestereva, NS and Tseilikman, OB and Komelkova, MV and Kondashevskaya, MV and Goryacheva, AV and Lapshin, MS and Platkovskii, PO and Sarapultsev, AP and Alliluev, AV and Downey, HF | Excluded | The article was excluded based on title and abstract screening as it did not meet the inclusion and exclusion criteria. |
| Influence of Aerobic Training and Combinations of Interventions on Cognition and Neuroplasticity after Stroke | 2016 | Constans, A and Pin-Barre, C and Temprado, JJ and Decherchi, P and Laurin, J | Excluded | The article was excluded based on title and abstract screening as it did not meet the inclusion and exclusion criteria. |
| A Systematic Review and Meta-Analysis on the Effects of Exercise on the Endocannabinoid System | 2022 | Desai, S and Borg, B and Cuttler, C and Crombie, KM and Rabinak, CA and Hill, MN and Marusak, HA | Excluded | The article was excluded based on title and abstract screening as it did not meet the inclusion and exclusion criteria. |
| INFLUENCE OF PHYSICAL EXERCISE ON COGNITION: AN UPDATE ON PHYSIOLOGICAL MECHANISMS | 2014 | Merege, CAA and Alves, CRR and Sepúlveda, CA and Costa, AD and Lancha, AH and Gualano, B | Excluded | The article was excluded based on title and abstract screening as it did not meet the inclusion and exclusion criteria. |
| Shaping the adult brain with exercise during development: Emerging evidence and knowledge gaps | 2019 | Perez, EC and Bravo, DR and Rodgers, SP and Khan, AR and Leasure, JL | Excluded | The article was excluded based on title and abstract screening as it did not meet the inclusion and exclusion criteria. |
| The Influence of Dietary Factors in Central Nervous System Plasticity and Injury Recovery | 2011 | Gomez-Pinilla, F and Gomez, AG | Excluded | The article was excluded based on title and abstract screening as it did not meet the inclusion and exclusion criteria. |
| Antioxidant Enzymes in Brain Cortex of Rats Exposed to Acute, Chronic and Combined Stress | 2016 | Pejic, S and Stojiljkovic, V and Todorovic, A and Gavrilovic, L and Pavlovic, I and Popovic, N and Pajovic, SB | Excluded | The article was excluded based on title and abstract screening as it did not meet the inclusion and exclusion criteria. |
| Toluene mediated oxidative stress and granulo-monocytopoiesis | 2003 | Bozic, T and Stevanovic, J and Kovacevic, M and Jovic, S and Lukic, S and Petakov, M and Borozan, S and Mijacevic, Z and Knezevic, M and Bulajic, S | Excluded | The article was excluded based on title and abstract screening as it did not meet the inclusion and exclusion criteria. |
| Exercise is brain food: The effects of physical activity on cognitive function | 2008 | Ploughman, M | Excluded | The article was excluded based on title and abstract screening as it did not meet the inclusion and exclusion criteria. |
| Estimation of salivary protein thiols and total antioxidant power of saliva in brain tumor patients | 2010 | Suma, HR and Prabhu, K and Shenoy, RP and Annaswamy, R and Rao, S and Rao, A | Excluded | The article was excluded based on title and abstract screening as it did not meet the inclusion and exclusion criteria. |
| Beneficial Effects of Exercise Pretreatment in a Sporadic Alzheimer's Rat Model | 2018 | Wu, CY and Yang, LD and Tucker, D and Dong, Y and Zhu, L and Duan, R and Liu, TCY and Zhang, QG | Excluded | The article was excluded based on title and abstract screening as it did not meet the inclusion and exclusion criteria. |
| The Effects of Acute Physical Exercise on Memory, Peripheral BDNF, and Cortisol in Young Adults | 2016 | Hötting, K and Schickert, N and Kaiser, J and Röder, B and Schmidt-Kassow, M | Excluded | The article was excluded based on title and abstract screening as it did not meet the inclusion and exclusion criteria. |
| Possible involvement of GABAergic modulation in the protective effect of gabapentin against immobilization stress-induced behavior alterations and oxidative damage in mice | 2007 | Kumar, A and Goyal, R | Excluded | The article was excluded based on title and abstract screening as it did not meet the inclusion and exclusion criteria. |
| An overview of the molecular and physiological antidepressant mechanisms of physical exercise in animal models of depression | 2022 | de Oliveira, LRS and Machado, FSM and Rocha-Dias, I and Magalhaes, CODE and De Sousa, RAL and Cassilhas, RC | Excluded | The article was excluded based on title and abstract screening as it did not meet the inclusion and exclusion criteria. |
| The Contribution of Physical Exercise to Brain Resilience | 2021 | Arida, RM and Teixeira-Machado, L | Excluded | The article was excluded based on title and abstract screening as it did not meet the inclusion and exclusion criteria. |
| Exercise increases BDNF levels in the striatum and decreases depressive-like behavior in chronically stressed rats | 2009 | Marais, L and Stein, DJ and Daniels, WMU | Excluded | The article was excluded based on title and abstract screening as it did not meet the inclusion and exclusion criteria. |
| Physical exercise preventsmemory impairment in an animal model of hypertension through modulation of CD39 and CD73 activities and A<sub>2A</sub> receptor expression | 2019 | Cardoso, AM and Manfredi, LH and Zanini, D and Bagatini, MD and Gutierres, JM and Carvalho, F and Tremblay, A and Belló-Klein, A and Rubin, MA and Morsch, VM and Sévigny, J and Schetinger, MRC | Excluded | The article was excluded based on title and abstract screening as it did not meet the inclusion and exclusion criteria. |
| Cortical and hippocampal expression of inflammatory and intracellular signaling proteins in aged rats submitted to aerobic and resistance physical training | 2018 | Henrique, JS and França, EF and Cardoso, FD and Serra, FT and de Almeida, AA and Fernandes, J and Arida, RM and da Silva, SG | Excluded | The article was excluded based on title and abstract screening as it did not meet the inclusion and exclusion criteria. |
| AEROBIC EXERCISE PREVENTS AGE-DEPENDENT COGNITIVE DECLINE AND REDUCES ANXIETY-RELATED BEHAVIORS IN MIDDLE-AGED AND OLD RATS | 2012 | Pietrelli, A and Lopez-Costa, J and Goñi, R and Brusco, A and Basso, N | Excluded | The article was excluded based on title and abstract screening as it did not meet the inclusion and exclusion criteria. |
| Early exercise promotes positive hippocampal plasticity and improves spatial memory in the adult life of rats | 2012 | da Silva, SG and Unsain, N and Mascó, DH and Toscano-Silva, M and de Amorim, HA and Araújo, BHS and Simoes, PSR and Naffah-Mazzacoratti, MD and Mortara, RA and Scorza, FA and Cavalheiro, EA and Arida, RM | Excluded | The article was excluded based on title and abstract screening as it did not meet the inclusion and exclusion criteria. |
| Neonatal hypoxia-ischemia caused mild motor dysfunction, recovered by acrobatic training, without affecting morphological structures involved in motor control in rats | 2019 | Confortim, HD and Deniz, BF and de Almeida, W and Miguel, PM and Bronauth, L and Vieira, MC and de Oliveira, BC and Pereira, LO | Excluded | The article was excluded based on title and abstract screening as it did not meet the inclusion and exclusion criteria. |
| The use of telomere length as a predictive biomarker for injury prognosis in juvenile rats following a concussion/mild traumatic brain injury | 2016 | Hehar, H and Mychasiuk, R | Excluded | The article was excluded based on title and abstract screening as it did not meet the inclusion and exclusion criteria. |
| Physical exercise mitigates behavioral impairments in a rat model of sporadic Alzheimer's disease | 2020 | Bernardo, TC and Beleza, J and Rizo-Roca, D and Santos-Alves, E and Leal, C and Martins, MJ and Ascensao, A and Magalhaes, J | Excluded | The article was excluded based on title and abstract screening as it did not meet the inclusion and exclusion criteria. |
| Environmental enrichment and exercise are better than social enrichment to reduce memory deficits in amyloid beta neurotoxicity | 2018 | Lima, MGP and Schimidt, HL and Garcia, A and Daré, LR and Carpes, FP and Izquierdo, I and Mello-Carpes, PB | Excluded | The article was excluded based on title and abstract screening as it did not meet the inclusion and exclusion criteria. |
| Neonatal treatment with resveratrol decreases postural and strength impairments and improves mitochondrial function in the somatosensory cortex rats submitted to cerebral palsy | 2023 | Souza, VD and Manhaes-de-Castro, R and Pereira, SD and Calado, CMSD and de Silveira, BS and Araújo, ERD and Silva, SCD and dos Santos, OH Jr and Lagranha, CJ and da Silva, LKTM and Toscano, AE | Excluded | The article was excluded based on title and abstract screening as it did not meet the inclusion and exclusion criteria. |
| Administration of diphenyl diselenide (PhSe)<sub>2</sub> following repetitive mild traumatic brain injury exacerbates anxiety-like symptomology in a rat model | 2020 | Yamakawa, GR and Eyolfson, E and Weerawardhena, H and Mychasiuk, R | Excluded | The article was excluded based on title and abstract screening as it did not meet the inclusion and exclusion criteria. |
| The Effects of Creatine Supplementation and Physical Exercise on Traumatic Brain Injury | 2016 | Royes, LFF and Cassol, G | Excluded | The article was excluded based on title and abstract screening as it did not meet the inclusion and exclusion criteria. |
| Physical Exercise Decreases Endoplasmic Reticulum Stress in Central and Peripheral Tissues of Rodents: A Systematic Review | 2023 | Fernandes, MSD and Badicu, G and Santos, GCJ and Filgueira, TO and Henrique, RD and de Souza, RF and Aidar, FJ and Souto, FO and Brum, PC and Lagranha, CJ | Excluded | The article was excluded based on title and abstract screening as it did not meet the inclusion and exclusion criteria. |
| The effects of different aerobic exercise intensities on serum serotonin concentrations and their association with Stroop task performance: a randomized controlled trial | 2016 | Zimmer, P and Stritt, C and Bloch, W and Schmidt, FP and Hübner, ST and Binnebössel, S and Schenk, A and Oberste, M | Excluded | The article was excluded based on title and abstract screening as it did not meet the inclusion and exclusion criteria. |
| Cognitive decline prevention in offspring of Pb<SUP>+2</SUP> exposed mice by maternal aerobic training and Cur/CaCO<sub>3</sub>@Cur supplementations: In vitro and in vivo studies | 2021 | Amooei, M and Meshkati, Z and Nasiri, R and Dakhili, AB | Excluded | The article was excluded based on title and abstract screening as it did not meet the inclusion and exclusion criteria. |
| Exercise and Implicit Memory: A Brief Systematic Review | 2018 | Loprinzi, PD and Edwards, MK | Excluded | The article was excluded based on title and abstract screening as it did not meet the inclusion and exclusion criteria. |
| Swimming training attenuates oxidative damage and increases enzymatic but not non-enzymatic antioxidant defenses in the rat brain | 2016 | Nonato, L.F. and Rocha-Vieira, E. and Tossige-Gomes, R. and Soares, A.A. and Soares, B.A. and Freitas, D.A. and Oliveira, M.X. and Mendonça, V.A. and Lacerda, A.C. and Massensini, A.R. and Leite, H.R. | Included |  |
| Acute Effects of Moderate and Strenuous Running on Trace Element Distribution in the Brain, Liver, and Spleen of Trained Rats | 2013 | Ergen, K and Ince, H and Düzova, H and Karakoç, Y and Emre, MH | Excluded | The article was excluded based on title and abstract screening as it did not meet the inclusion and exclusion criteria. |
| Selenium supplementation prevents lipid peroxidation caused by arduous exercise in rat brain tissue | 2011 | Akil, M and Bicer, M and Menevse, E and Baltaci, AK and Mogulkoc, R | Excluded | The article was excluded based on title and abstract screening as it did not meet the inclusion and exclusion criteria. |
| Prior short-term exercise prevents behavioral and biochemical abnormalities induced by single prolonged stress in a rat model of posttraumatic stress disorder | 2022 | Mirjalili, R and Shokouh, E and Dehkordi, NS and Afsari, R and Shafia, S and Rashidy-Pour, A | Excluded | The article was excluded based on title and abstract screening as it did not meet the inclusion and exclusion criteria. |
| Interactive and delayed effects of pyridostigmine and physical stress on biochemical and histological changes in peripheral tissues of mice | 2000 | Somani, SM and Husain, K and Asha, T and Helfert, R | Excluded | The article was excluded based on title and abstract screening as it did not meet the inclusion and exclusion criteria. |
| Possible GABAergic mechanism in the protective effect of allopregnenolone against immobilization stress | 2009 | Kumar, A and Goyal, R and Prakash, A | Excluded | The article was excluded based on title and abstract screening as it did not meet the inclusion and exclusion criteria. |
| Physical exercise neuroprotects ovariectomized 3xTg-AD mice through BDNF mechanisms | 2014 | García-Mesa, Y and Pareja-Galeano, H and Bonet-Costa, V and Revilla, S and Gómez-Cabrera, MC and Gambini, J and Giménez-Llort, L and Cristòfol, R and Viña, J and Sanfeliu, C | Excluded | The article was excluded based on title and abstract screening as it did not meet the inclusion and exclusion criteria. |
| Behavioral dysfunction, brain oxidative stress, and impaired mitochondrial electron transfer in aging mice | 2002 | Navarro, A and Del Pino, MJS and Gómez, C and Peralta, JL and Boveris, A | Excluded | The article was excluded based on title and abstract screening as it did not meet the inclusion and exclusion criteria. |
| Potential roles for vitamins E and C in combination in modulating exhaustive swimming and high altitude-associated lung injury in rats | 2012 | Al-Hashem, FH | Excluded | The article was excluded based on title and abstract screening as it did not meet the inclusion and exclusion criteria. |
| Effects of high-intensity endurance exercise training in the G93A mouse model of amyotrophic lateral sclerosis | 2004 | Mahoney, DJ and Rodriguez, C and Devries, M and Yasuda, N and Tarnopolsky, MA | Excluded | The article was excluded based on title and abstract screening as it did not meet the inclusion and exclusion criteria. |
| Nerve Growth Factor, Stress and Diseases | 2021 | Ceci, FM and Ferraguti, G and Petrella, C and Greco, A and Tirassa, P and Iannitelli, A and Ralli, M and Vitali, M and Ceccanti, M and Chaldakov, GN and Versacci, P and Fiore, M | Excluded | The article was excluded based on title and abstract screening as it did not meet the inclusion and exclusion criteria. |
| Effect of Exercise on Diabetes-Induced Oxidative Stress in the Rat Hippocampus | 2012 | Alipour, M and Salehi, I and Soufi, FG | Excluded | The article was excluded based on title and abstract screening as it did not meet the inclusion and exclusion criteria. |
| Swimming exercise ameliorates depressive-like behavior by anti-inflammation activity, rebalancing gut Escherichia coli and Lactobacilli | 2022 | Ding, ZF and Du, LF | Excluded | The article was excluded based on title and abstract screening as it did not meet the inclusion and exclusion criteria. |
| Exercise as an antidepressant: exploring its therapeutic potential | 2023 | Hwang, DJ and Koo, JH and Kim, TK and Jang, YC and Hyun, AH and Yook, JS and Yoon, CS and Cho, JY | Excluded | The article was excluded based on title and abstract screening as it did not meet the inclusion and exclusion criteria. |
| Acetyl-L-carnitine as a putative candidate for the treatment of stress-related psychiatric disorders: Novel evidence from a zebrafish model | 2019 | Marcon, M and Mocelin, R and de Oliveira, DL and Araujo, ASD and Herrmann, AP and Piato, A | Excluded | The article was excluded based on title and abstract screening as it did not meet the inclusion and exclusion criteria. |
| Brain and Spinal Cord Interaction: A Dietary Curcumin Derivative Counteracts Locomotor and Cognitive Deficits After Brain Trauma | 2011 | Wu, AG and Ying, Z and Schubert, D and Gomez-Pinilla, F | Excluded | The article was excluded based on title and abstract screening as it did not meet the inclusion and exclusion criteria. |
| Increase in Blood-Brain Barrier Permeability, Oxidative Stress, and Activated Microglia in a Rat Model of Blast-Induced Traumatic Brain Injury | 2010 | Readnower, RD and Chavko, M and Adeeb, S and Conroy, MD and Pauly, JR and McCarron, RM and Sullivan, PG | Excluded | The article was excluded based on title and abstract screening as it did not meet the inclusion and exclusion criteria. |
| Long-term effects of environmental stimulation following hypoxia-ischemia on the oxidative state and BDNF Revels in rat hippocampus and frontal cortex | 2009 | Pereira, LO and Nabinger, PM and Strapasson, ACP and Nardin, P and Gonçalves, CAS and Siqueira, IR and Netto, CA | Excluded | The article was excluded based on title and abstract screening as it did not meet the inclusion and exclusion criteria. |
| Possible role of citalopram and desipramine against sleep deprivation-induced anxiety like-behavior alterations and oxidative damage in mice | 2008 | Garg, R and Kumar, A | Excluded | The article was excluded based on title and abstract screening as it did not meet the inclusion and exclusion criteria. |
| Evolutionary Basis of Human Running and Its Impact on Neural Function | 2016 | Schulkin, J | Excluded | The article was excluded based on title and abstract screening as it did not meet the inclusion and exclusion criteria. |
| Aerobic exercise increases sprouting angiogenesis in the male rat motor cortex | 2020 | Stevenson, ME and Miller, CC and Owen, HA and Swain, RA | Excluded | The article was excluded based on title and abstract screening as it did not meet the inclusion and exclusion criteria. |
| Antifatigue effect of sea buckthorn seed oil on swimming fatigue in mice | 2023 | An, ZH and Wang, YF and Li, XB and Jin, H and Gong, YL | Excluded | The article was excluded based on title and abstract screening as it did not meet the inclusion and exclusion criteria. |
| Enhanced physical endurance and improved memory performance following taurine administration in rats | 2017 | Sajid, I and Ahmad, S and Emad, S and Batool, Z and Khaliq, S and Anis, L and Tabassum, S and Madiha, S and Liaquat, L and Sadir, S and Perveen, T and Haider, S | Excluded | The article was excluded based on title and abstract screening as it did not meet the inclusion and exclusion criteria. |
| Coherences Between the Metabolic Syndrome, Depression, Stress and Physical Activity | 2009 | Neumann, NU and Frasch, K | Excluded | The article was excluded based on title and abstract screening as it did not meet the inclusion and exclusion criteria. |
| Combination of quercetin and exercise training attenuates depression in rats with 1,2-dimethylhydrazine-induced colorectal cancer: Possible involvement of inflammation and BDNF signalling | 2020 | Sadighparvar, S and Darband, SG and Yousefi, B and Kaviani, M and Ghaderi-Pakdel, F and Mihanfar, A and Babaei, G and Mobaraki, K and Majidinia, M | Excluded | The article was excluded based on title and abstract screening as it did not meet the inclusion and exclusion criteria. |
| Voluntary exercise protects against stress-induced decreased in brain-derived neurotrophic factor protein expression | 2004 | Adlard, PA and Cotman, CW | Excluded | The article was excluded based on title and abstract screening as it did not meet the inclusion and exclusion criteria. |
| Central gene expression changes associated with enhanced neuroendocrine and autonomic response habituation to repeated noise stress after voluntary wheel running in rats | 2013 | Sasse, SK and Nyhuis, TJ and Masini, CV and Day, HEW and Campeau, S | Excluded | The article was excluded based on title and abstract screening as it did not meet the inclusion and exclusion criteria. |
| Tanshinol borneol ester on nanostructured lipid carriers has longer brain and systemic effector retention and better antioxidant activity in vivo | 2018 | Yuan, XY and Fei, FH and Sun, HM and Xiao, CN and Zhao, XF and Zhang, YJ and Zheng, XH | Excluded | The article was excluded based on title and abstract screening as it did not meet the inclusion and exclusion criteria. |
| Effect of physical exercise on changes in activities of creatine kinase, cytochrome c oxidase and ATP levels caused by ovariectomy | 2014 | Siebert, C and Kolling, J and Scherer, EBS and Schmitz, F and da Cunha, MJ and Mackedanz, V and de Andrade, RB and Wannmacher, CMD and Wyse, ATS | Excluded | The article was excluded based on title and abstract screening as it did not meet the inclusion and exclusion criteria. |
| Oxidative stress in response to heat stress in wild caught Namaqua rock mice, <i>Micaelamys namaquensis</i> | 2021 | Jacobs, PJ and Oosthuizen, MK and Mitchell, C and Blount, JD and Bennett, NC | Excluded | The article was excluded based on title and abstract screening as it did not meet the inclusion and exclusion criteria. |
| The Role of PGC-1α/UCP2 Signaling in the Beneficial Effects of Physical Exercise on the Brain | 2019 | Bristot, VJD and Alves, ACD and Cardoso, LR and Scheffer, DD and Aguiar, AS | Excluded | The article was excluded based on title and abstract screening as it did not meet the inclusion and exclusion criteria. |
| Physical exercise ameliorates psychiatric disorders and cognitive dysfunctions by hippocampal mitochondrial function and neuroplasticity in post-traumatic stress disorder | 2019 | Seo, JH and Park, HS and Park, SS and Kim, CJ and Kim, DH and Kim, TW | Excluded | The article was excluded based on title and abstract screening as it did not meet the inclusion and exclusion criteria. |
| Cardiovascular and sympathetic effects of nitric oxide inhibition at rest and during static exercise in humans | 1997 | Owlya, R and Vollenweider, L and Trueb, L and Sartori, C and Lepori, M and Nicod, P and Scherrer, U | Excluded | The article was excluded based on title and abstract screening as it did not meet the inclusion and exclusion criteria. |
| Reduced miR-659-3p Levels Correlate with Progranulin Increase in Hypoxic Conditions: Implications for Frontotemporal Dementia | 2016 | Piscopo, P and Grasso, M and Fontana, F and Crestini, A and Puopolo, M and Del Vescovo, V and Venerosi, A and Calamandrei, G and Vencken, SF and Greene, CM and Confaloni, A and Denti, MA | Excluded | The article was excluded based on title and abstract screening as it did not meet the inclusion and exclusion criteria. |
| Creatine-facilitated protection of stress caused by disrupted circadian rhythm | 2018 | Burjanadze, G and Shengelia, M and Dachanidze, N and Mikadze, M and Menabde, K and Koshoridze, N | Excluded | The article was excluded based on title and abstract screening as it did not meet the inclusion and exclusion criteria. |
| Brain monoamines, exercise, and behavioral stress: Animal models | 1997 | Dishman, RK | Excluded | The article was excluded based on title and abstract screening as it did not meet the inclusion and exclusion criteria. |
| Effects of swimming exercise on neuropathic pain in a rat model: role of glutamate | 2024 | Ghanbari, A and Ghasemi, S and Khaleghian, A | Excluded | The article was excluded based on title and abstract screening as it did not meet the inclusion and exclusion criteria. |
| Irisin Contributes to Neuroprotection by Promoting Mitochondrial Biogenesis After Experimental Subarachnoid Hemorrhage | 2021 | Tu, TQ and Yin, SG and Pang, JW and Zhang, XH and Zhang, LF and Zhang, YX and Xie, YK and Guo, KC and Chen, LG and Peng, JH and Jiang, Y | Excluded | The article was excluded based on title and abstract screening as it did not meet the inclusion and exclusion criteria. |
| Chronic voluntary wheel running facilitates corticosterone response habituation to repeated audiogenic stress exposure in male rats | 2008 | Sasse, SK and Greenwood, BN and Masini, CV and Nyhuis, TJ and Fleshner, M and Day, HEW and Campeau, S | Excluded | The article was excluded based on title and abstract screening as it did not meet the inclusion and exclusion criteria. |
| Does brain-derived neurotrophic factor mediate the effects of exercise on memory? | 2019 | Loprinzi, PD | Excluded | The article was excluded based on title and abstract screening as it did not meet the inclusion and exclusion criteria. |
| Voluntary Wheel Running Did Not Alter Gene Expression in 5xfad Mice, but in Wild-Type Animals Exclusively after One-Day of Physical Activity | 2021 | Wierczeiko, A and Gammel, L and Radyushkin, K and Nguyen, VTT and Todorov, H and Gerber, S and Endres, K | Excluded | The article was excluded based on title and abstract screening as it did not meet the inclusion and exclusion criteria. |
| Short-term exposure to radiofrequency radiation and metabolic enzymes' activities during pregnancy and prenatal development | 2022 | Tomruk, A and Ozgur-Buyukatalay, E and Ozturk, GG and Ulusu, NN | Excluded | The article was excluded based on title and abstract screening as it did not meet the inclusion and exclusion criteria. |
| Role of BDNF val66met polymorphism in modulating exercised-induced emotional memories | 2017 | Keyan, D and Bryant, RA | Excluded | The article was excluded based on title and abstract screening as it did not meet the inclusion and exclusion criteria. |
| Exercise improves visual deficits tested by visual evoked potentials in streptozotocin-induced diabetic rats | 2007 | Özkaya, YG and Agar, A and Hacioglu, G and Yargicoglu, P | Excluded | The article was excluded based on title and abstract screening as it did not meet the inclusion and exclusion criteria. |
| Impact of monomeric and aggregated wild-type and A30P/A53T double-mutant α-synuclein on antioxidant mechanism and glutamate metabolic profile of cultured astrocytes | 2022 | Raj, A and Kaushal, A and Datta, I | Excluded | The article was excluded based on title and abstract screening as it did not meet the inclusion and exclusion criteria. |
| Cigarette Smoke Inhibits Brain Mitochondrial Adaptations of Exercised Mice | 2011 | Speck, AE and Fraga, D and Soares, P and Scheffer, DL and Silva, LA and Aguiar, AS and Estreck, EL and Pinho, RA | Excluded | The article was excluded based on title and abstract screening as it did not meet the inclusion and exclusion criteria. |
| Brain angiotensin AT<sub>1</sub> receptors as specific regulators of cardiovascular reactivity to acute psychoemotional stress | 2011 | Mayorov, DN | Excluded | The article was excluded based on title and abstract screening as it did not meet the inclusion and exclusion criteria. |
| Impact of two different types of exercise training on AMPH addiction: Role of hippocampal neurotrophins | 2022 | Segat, HJ and Martini, F and Roversi, K and Rosa, SG and Muller, SG and Rossato, DR and Nogueira, CW and Burger, ME | Excluded | The article was excluded based on title and abstract screening as it did not meet the inclusion and exclusion criteria. |
| The Cerebral Protective Effect of Novel Erinacines from <i>Hericium erinaceus</i> Mycelium on In Vivo Mild Traumatic Brain Injury Animal Model and Primary Mixed Glial Cells via Nrf2-Dependent Pathways | 2024 | Lee, KF and Hsieh, YY and Tung, SY and Teng, CC and Cheng, KC and Hsieh, MC and Huang, CY and Lee, KC and Lee, LY and Chen, WP and Chen, CC and Kuo, HC | Excluded | The article was excluded based on title and abstract screening as it did not meet the inclusion and exclusion criteria. |
| The effect of regular exercise on antioxidant enzyme activities and lipid peroxidation levels in both hippocampi after occluding one carotid in rat | 2014 | Moghaddasi, M and Javanmard, SH and Reisi, P and Tajadini, M and Taati, M | Excluded | The article was excluded based on title and abstract screening as it did not meet the inclusion and exclusion criteria. |
| Physical activity and environmental enrichment: Behavioural effects of exposure to different housing conditions in mice | 2019 | Rabadán, R and Ramos-Campos, M and Redolat, R and Mesa-Gresa, P | Excluded | The article was excluded based on title and abstract screening as it did not meet the inclusion and exclusion criteria. |
| Swimming exercise effects on the expression of HSP70 and iNOS in hippocampus and prefrontal cortex in combined stress | 2010 | Liu, X and Yang, LJ and Fan, SJ and Jiang, H and Pan, F | Excluded | The article was excluded based on title and abstract screening as it did not meet the inclusion and exclusion criteria. |
| Effect of endurance training on seizure susceptibility, behavioral changes and neuronal damage after kainate-induced status epilepticus in spontaneously hypertensive rats | 2015 | Tchelzalarova, J and Shishmanova, M and Atanasova, D and Stefanova, M and Alova, L and Lazarov, N and Georgieva, K | Excluded | The article was excluded based on title and abstract screening as it did not meet the inclusion and exclusion criteria. |
| Optimal physical performance in athletes: Key roles of dopamine in a specific neurotransmitter hormonal mechanism | 1995 | Gilbert, C | Excluded | The article was excluded based on title and abstract screening as it did not meet the inclusion and exclusion criteria. |
| How the enriched get richer? Experience-dependent modulation of microRNAs and the therapeutic effects of environmental enrichment | 2020 | Kuznetsova, M and Wilson, C and Hannan, AJ and Renoir, T | Excluded | The article was excluded based on title and abstract screening as it did not meet the inclusion and exclusion criteria. |
| Sportomics: Building a new concept in metabolic studies and exercise science | 2014 | Bassini, A and Cameron, LC | Excluded | The article was excluded based on title and abstract screening as it did not meet the inclusion and exclusion criteria. |
| Antidepressant Effect and Modulation of the Redox System Mediated by Tannic Acid on Lipopolysaccharide-Induced Depressive and Inflammatory Changes in Mice | 2020 | Luduvico, KP and Spohr, L and Soares, MSP and Teixeira, FC and de Farias, AS and Bona, NP and Pedra, NS and Felix, ADC and Spanevello, RM and Stefanello, FM | Excluded | The article was excluded based on title and abstract screening as it did not meet the inclusion and exclusion criteria. |
| Treadmill exercise ameliorates memory deficits and hippocampal inflammation in ovalbumin-sensitized juvenile rats | 2020 | Mokhtari-Zaer, A and Hosseini, M and Roshan, NM and Boskabady, MH | Excluded | The article was excluded based on title and abstract screening as it did not meet the inclusion and exclusion criteria. |
| The complex role of physical exercise and reactive oxygen species on brain | 2013 | Radak, Zsolt and Marton, Orsolya and Nagy, Eniko and Koltai, Erika and Goto, Sataro | Included |  |
| Forced Treadmill Exercise Prevents Spatial Memory Deficits in Aged Rats Probably Through the Activation of Na<SUP>+</SUP>, K<SUP>+</SUP>-ATPase in the Hippocampus | 2017 | Vanzella, C and Sanches, EF and Odorcyk, FK and Nicola, F and Kolling, J and Longoni, A and dos Santos, TM and Wyse, ATD and Netto, CA | Excluded | The article was excluded based on title and abstract screening as it did not meet the inclusion and exclusion criteria. |
| GIT2 Acts as a Systems-Level Coordinator of Neurometabolic Activity and Pathophysiological Aging | 2016 | Martin, B and Chadwick, W and Janssens, J and Premont, RT and Schmalzigaug, R and Becker, KG and Lehrmann, E and Wood, WH and Zhang, YQ and Siddiqui, S and Park, SS and Cong, WN and Daimon, CM and Maudsley, S | Excluded | The article was excluded based on title and abstract screening as it did not meet the inclusion and exclusion criteria. |
| Effects of varied-intensity endurance exercise training on oxidative and antioxidant factors in the liver of rats with valproic acid-induced autism | 2023 | Mirzavandi, F and Sabet, N and Aminzadeh, A and Heidari, M and Pouya, F and Moslemizadeh, A and Parizi, AS and Bashiri, H | Excluded | The article was excluded based on title and abstract screening as it did not meet the inclusion and exclusion criteria. |
| Training the brain: could it improve multiple sclerosis treatment? | 2020 | Silva, BA and Miglietta, EA and Ferrari, CC | Excluded | The article was excluded based on title and abstract screening as it did not meet the inclusion and exclusion criteria. |
| Resistance exercise was safe for the pregnancy and offspring?s development and partially protected rats against early life stress-induced effects | 2023 | Santos, ASD and Segabinazi, E and de Almeida, W and Faustino, AM and Bronauth, LP and dos Santos, TM and Ferreira, FS and Wyse, ATS and Marcuzzo, S and Pereira, LO | Excluded | The article was excluded based on title and abstract screening as it did not meet the inclusion and exclusion criteria. |
| Potential clinical applications of Ashwagandha (<i>Withania somnifera</i>) in medicine and neuropsychiatry | 2022 | D'Cruz, M and Andrade, C | Excluded | The article was excluded based on title and abstract screening as it did not meet the inclusion and exclusion criteria. |
| The life-shortening effect of reduced physical activity is abolished by a fat rich diet | 1998 | Mlekusch, W and Tillian, M and Lamprecht, M and Oettl, K and Krainz, H and Reibnegger, G | Excluded | The article was excluded based on title and abstract screening as it did not meet the inclusion and exclusion criteria. |
| Physical activity in a swimming pool attenuates memory impairment by reducing glutamate and inflammatory cytokines and increasing BDNF in the brain of mice with type 2 diabetes | 2023 | Shekarchian, M and Peeri, M and Azarbayjani, MA | Excluded | The article was excluded based on title and abstract screening as it did not meet the inclusion and exclusion criteria. |
| EXERCISE AFFECTS MEMORY ACQUISITION, ANXIETY-LIKE SYMPTOMS AND ACTIVITY OF MEMBRANE-BOUND ENZYME IN BRAIN OF RATS FED WITH DIFFERENT DIETARY FATS: IMPAIRMENTS OF <i>TRANS</i> FAT | 2011 | Teixeira, AM and Pase, CS and Boufleur, N and Roversi, K and Barcelos, RCS and Benvegnú, DM and Segat, HJ and Dias, VT and Reckziegel, P and Trevizol, F and Dolci, GS and Carvalho, NR and Soares, FAA and Rocha, JBT and Emanuelli, T and Bürger, ME | Excluded | The article was excluded based on title and abstract screening as it did not meet the inclusion and exclusion criteria. |
| Exercise and Mental Health: Many Reasons to Move | 2009 | Deslandes, A and Moraes, H and Ferreira, C and Veiga, H and Silveira, H and Mouta, R and Pompeu, FAMS and Coutinho, ES and Laks, J | Excluded | The article was excluded based on title and abstract screening as it did not meet the inclusion and exclusion criteria. |
| Evolution of Neuroplasticity in Response to Physical Activity in Old Age: The Case for Dancing | 2017 | Müller, P and Rehfeld, K and Schmicker, M and Hökelmann, A and Dordevic, M and Lessmann, V and Brigadski, T and Kaufmann, J and Müller, NG | Excluded | The article was excluded based on title and abstract screening as it did not meet the inclusion and exclusion criteria. |
| Advanced glycation end products, oxidative stress and metalloproteinases are altered in the cerebral microvasculature during aging | 2007 | Safciuc, F and Constantin, A and Manea, A and Nicolae, M and Popov, D and Raicu, M and Alexandru, D and Constantinescu, E | Excluded | The article was excluded based on title and abstract screening as it did not meet the inclusion and exclusion criteria. |
| Low-speed treadmill running exercise improves memory function after transient middle cerebral artery occlusion in rats | 2013 | Shimada, H and Hamakawa, M and Ishida, A and Tamakoshi, K and Nakashima, H and Ishida, K | Excluded | The article was excluded based on title and abstract screening as it did not meet the inclusion and exclusion criteria. |
| Effects of Simultaneous Exposure to a Western Diet and Wheel-Running Training on Brain Energy Metabolism in Female Rats | 2021 | Nowacka-Chmielewska, MM and Liskiewicz, D and Grabowska, K and Liskiewicz, A and Marczak, L and Wojakowska, A and Pondel, N and Grabowski, M and Barski, JJ and Malecki, A | Excluded | The article was excluded based on title and abstract screening as it did not meet the inclusion and exclusion criteria. |
| MOLECULAR ASPECTS INVOLVED IN SWIMMING EXERCISE TRAINING REDUCING ANHEDONIA IN A RAT MODEL OF DEPRESSION | 2011 | Sigwalt, AR and Budde, H and Helmich, I and Glaser, V and Ghisoni, K and Lanza, S and Cadore, EL and Lhullier, FLR and de Bem, AF and Hohl, A and de Matos, FJ and de Oliveira, PA and Prediger, RD and Guglielmo, LGA and Latini, A | Excluded | The article was excluded based on title and abstract screening as it did not meet the inclusion and exclusion criteria. |
| Moderate-Intensity Physical Exercise Protects Against Experimental 6-Hydroxydopamine-Induced Hemiparkinsonism Through Nrf2-Antioxidant Response Element Pathway | 2016 | Aguiar, AS and Duzzioni, M and Remor, AP and Tristao, FSM and Matheus, FC and Raisman-Vozari, R and Latini, A and Prediger, RD | Excluded | The article was excluded based on title and abstract screening as it did not meet the inclusion and exclusion criteria. |
| PHYSICAL EXERCISE AS A NOVEL ANTIDEPRESSANT AGENT - POSSIBLE ROLE OF SEROTONIN RECEPTOR SUBTYPES | 1994 | DEY, S | Excluded | The article was excluded based on title and abstract screening as it did not meet the inclusion and exclusion criteria. |
| Social isolation stress-induced oxidative damage in mouse brain and its modulation by majonoside-R2, a Vietnamese ginseng saponin | 2005 | Huong, NTT and Murakami, Y and Tohda, M and Watanabe, H and Matsumoto, K | Excluded | The article was excluded based on title and abstract screening as it did not meet the inclusion and exclusion criteria. |
| Effects of long-term voluntary exercise on learning and memory processes: dependency of the task and level of exercise | 2009 | García-Capdevila, S and Portell-Cortés, I and Torras-Garcia, M and Coll-Andreu, M and Costa-Miserachs, D | Excluded | The article was excluded based on title and abstract screening as it did not meet the inclusion and exclusion criteria. |
| Early Life Stress Affects <i>Bdnf</i> Regulation: A Role for Exercise Interventions | 2022 | Campbell, TS and Donoghue, KM and Ghosh, U and Nelson, CM and Roth, TL | Excluded | The article was excluded based on title and abstract screening as it did not meet the inclusion and exclusion criteria. |
| Hypothalamic <i>Crh</i>/<i>Avp</i>, Plasmatic Glucose and Lactate Remain Unchanged During Habituation to Forced Exercise | 2020 | Toval, A and Vicente-Conesa, F and Martínez-Ortega, P and Kutsenko, Y and Morales-Delgado, N and Garrigos, D and Alonso, A and Do Couto, BR and Popovic, M and Ferran, JL | Excluded | The article was excluded based on title and abstract screening as it did not meet the inclusion and exclusion criteria. |
| Oxidative Stress-Induced Adverse Effects of Three Statins Following Single or Repetitive Treatments in Mice | 2024 | Al-Shalchi, RF and Mohammad, FK | Excluded | The article was excluded based on title and abstract screening as it did not meet the inclusion and exclusion criteria. |
| BDNF induction with mild exercise in the rat hippocampus | 2007 | Soya, H and Nakamura, T and Deocaris, CC and Kimpara, A and Iimura, M and Fujikawa, T and Chang, H and McEwen, BS and Nishijima, T | Excluded | The article was excluded based on title and abstract screening as it did not meet the inclusion and exclusion criteria. |
| Compulsive exercise acutely upregulates rat hippocampal brain-derived neurotrophic factor | 2006 | Huang, AM and Jen, CJ and Chen, HF and Yu, L and Kuo, YM and Chen, HI | Excluded | The article was excluded based on title and abstract screening as it did not meet the inclusion and exclusion criteria. |
| Cerebrospinal fluid and plasma metabolomics of acute endurance exercise | 2022 | Li, KF and Schön, M and Naviaux, JC and Monk, JM and Alchus-Laiferová, N and Wang, L and Straka, I and Matejicka, P and Valkovic, P and Ukropec, J and Tarnopolsky, MA and Naviaux, RK and Ukropcová, B | Excluded | The article was excluded based on title and abstract screening as it did not meet the inclusion and exclusion criteria. |
| Physical activity, but not environmental complexity, facilitates HPA axis response habituation to repeated audiogenic stress despite neurotrophin mRNA regulation in both conditions | 2010 | Nyhuis, TJ and Masini, CV and Sasse, SK and Day, HEW and Campeau, S | Excluded | The article was excluded based on title and abstract screening as it did not meet the inclusion and exclusion criteria. |
| Synergistic effect of melatonin on exercise-induced neuronal reconstruction and functional recovery in a spinal cord injury animal model | 2010 | Park, K and Lee, Y and Park, S and Lee, S and Hong, Y and Lee, SK and Hong, Y | Excluded | The article was excluded based on title and abstract screening as it did not meet the inclusion and exclusion criteria. |
| Voluntary exercise does not ameliorate context memory and hyperarousal in a mouse model for post-traumatic stress disorder (PTSD) | 2013 | Cacciaglia, R and Krause-Utz, A and Vogt, MA and Schmahl, C and Flor, H and Gass, P | Excluded | The article was excluded based on title and abstract screening as it did not meet the inclusion and exclusion criteria. |
| Role of neurosteroid allopregnanolone on age-related differences in exercise-induced hypoalgesia in rats | 2019 | Aoyama, B and Kawano, T and Iwata, H and Nishigaki, A and Yamanaka, D and Tateiwa, H and Shigematsu-Locatelli, M and Eguchi, S and Locatelli, FM and Yokoyama, M | Excluded | The article was excluded based on title and abstract screening as it did not meet the inclusion and exclusion criteria. |
| The effect of exercise on the peripheral nerve in streptozotocin (STZ)-induced diabetic rats | 2015 | Jin, HY and Lee, KA and Park, TS | Excluded | The article was excluded based on title and abstract screening as it did not meet the inclusion and exclusion criteria. |
| The effect of exercise on brain antioxidant status of diabetic rats | 2002 | Özkaya, YG and Agar, A and Yargiçoglu, P and Hacioglu, G and Bilmen-Sarikçioglu, S and Özen, I and Alicigüzel, Y | Excluded | The article was excluded based on title and abstract screening as it did not meet the inclusion and exclusion criteria. |
| Stress induces rapid changes in central catecholaminergic activity in <i>Anolis carolinensis</i>:: Restraint and forced physical activity | 2005 | Waters, RP and Emerson, AJ and Watt, MJ and Forster, GL and Swallow, JG and Summers, CH | Excluded | The article was excluded based on title and abstract screening as it did not meet the inclusion and exclusion criteria. |
| Neuroprotective effects of resistance physical exercise on the APP/PS1 mouse model of Alzheimer's disease | 2023 | Campos, HC and Ribeiro, DE and Hashiguchi, D and Glaser, T and Milanis, MD and Gimenes, C and Suchecki, D and Arida, RM and Ulrich, H and Longo, BM | Excluded | The article was excluded based on title and abstract screening as it did not meet the inclusion and exclusion criteria. |
| VILAZODONE ENHANCES THE ENDURANCE CAPACITY AND IMPROVES THE BIOLOGICAL CHANGES IN TREADMILL TEST IN RATS | 2023 | Fotache, PA and Mititelu-Tartau, L and Serban, IL and Hancianu, M and Mircea, CG and Pelin, AM and Pavel, LL | Excluded | The article was excluded based on title and abstract screening as it did not meet the inclusion and exclusion criteria. |
| Differential effects of exercise on brain opioid receptor binding and activation in rats | 2015 | Arida, RM and da Silva, SG and de Almeida, AA and Cavlheiro, EA and Zavala-Tecuapetla, C and Brand, S and Rocha, L | Excluded | The article was excluded based on title and abstract screening as it did not meet the inclusion and exclusion criteria. |
| Modification of Neuromuscular Junction Protein Expression by Exercise and Doxorubicin | 2020 | Huertas, AM and Morton, AB and Hinkey, JM and Ichinoseki-Sekine, N and Smuder, AJ | Excluded | The article was excluded based on title and abstract screening as it did not meet the inclusion and exclusion criteria. |
| THE IMPACT OF THE FREQUENCY OF MODERATE EXERCISE ON MEMORY AND BRAIN-DERIVED NEUROTROPHIC FACTOR SIGNALING IN YOUNG ADULT AND MIDDLE-AGED RATS | 2012 | Costa, MS and Ardais, AP and Fioreze, GT and Mioranzza, S and Botton, PHS and Souza, D and Rocha, JBT and Porciúncula, LO | Excluded | The article was excluded based on title and abstract screening as it did not meet the inclusion and exclusion criteria. |
| Protective abilities of pyridoxine in experimental oxidative stress settings in vivo and in vitro | 2017 | Danielyan, KE and Simonyan, AA | Excluded | The article was excluded based on title and abstract screening as it did not meet the inclusion and exclusion criteria. |
| Acute stress affects the global DNA methylation profile in rat brain: Modulation by physical exercise | 2015 | Rodrigues, GM and Toffoli, LV and Manfredo, MH and Francis-Oliveira, J and Silva, AS and Raquel, HA and Martins-Pinge, MC and Moreira, EG and Fernandes, KB and Pelosi, GG and Gomes, MV | Excluded | The article was excluded based on title and abstract screening as it did not meet the inclusion and exclusion criteria. |
| Single session, high-intensity aerobic exercise fails to affect plasticity-related protein expression in the rat sensorimotor cortex | 2019 | Thacker, JS and Yeung, D and Chambers, PJ and Tupling, AR and Staines, WR and Mielke, JG | Excluded | The article was excluded based on title and abstract screening as it did not meet the inclusion and exclusion criteria. |
| Altered behavioural response to acute stress in mice lacking cellular prion protein | 2005 | Nico, PBC and De-Paris, F and Vinadé, ER and Amaral, OB and Rockenbach, I and Soares, BL and Guarnieri, R and Wichert-Ana, L and Calvo, F and Walz, R and Izquierdo, I and Sakamoto, AC and Brentani, R and Martins, VR and Bianchin, MM | Excluded | The article was excluded based on title and abstract screening as it did not meet the inclusion and exclusion criteria. |
| Neuroinflammation and aberrant hippocampal plasticity in a mouse model of emotional stress evoked by exposure to ultrasound of alternating frequencies | 2019 | Pavlov, D and Bettendorff, L and Gorlova, A and Olkhovik, A and Kalueff, AV and Ponomarev, ED and Inozemtsev, A and Chekhonin, V and Lesch, KP and Anthony, DC and Strekalova, T | Excluded | The article was excluded based on title and abstract screening as it did not meet the inclusion and exclusion criteria. |
| Physical exercise versus fluoxetine: Antagonistic effects on cortical spreading depression in Wistar rats | 2015 | Monteiro, HMC and Barreto-Silva, NL and dos Santos, GE and Santos, AD and Sousa, MSB and Amâncio-dos-Santos, A | Excluded | The article was excluded based on title and abstract screening as it did not meet the inclusion and exclusion criteria. |
| Exercise Maintains Dendritic Complexity in an Animal Model of Posttraumatic Stress Disorder | 2016 | Hoffman, JR and Cohen, H and Ostfeld, I and Kaplan, Z and Zohar, J and Cohen, H | Excluded | The article was excluded based on title and abstract screening as it did not meet the inclusion and exclusion criteria. |
| Sexual trauma and the female brain | 2016 | Shors, TJ and Millon, EM | Excluded | The article was excluded based on title and abstract screening as it did not meet the inclusion and exclusion criteria. |
| Two weeks of moderate intensity locomotor training increased corticosterone concentrations but did not alter the number of adropin-immunoreactive cells in the hippocampus of diabetic type 2 and control rats | 2021 | Ziarniak, K and Dudek, M and Matuszewska, J and Bijoch, L and Skrzypski, M and Celichowski, J and Sliwowska, JH | Excluded | The article was excluded based on title and abstract screening as it did not meet the inclusion and exclusion criteria. |
| Physical activity-antidepressant treatment combination: impact on brain-derived neurotrophic factor and behavior in an animal model | 2001 | Russo-Neustadt, A and Ha, T and Ramirez, R and Kesslak, JP | Excluded | The article was excluded based on title and abstract screening as it did not meet the inclusion and exclusion criteria. |
| Cognitive decline in heart failure: Biomolecular mechanisms and benefits of exercise | 2022 | Maroofi, A and Moro, T and Agrimi, J and Safari, F | Excluded | The article was excluded based on title and abstract screening as it did not meet the inclusion and exclusion criteria. |
| Stress and the gut-brain axis: Cognitive performance, mood state, and biomarkers of blood-brain barrier and intestinal permeability following severe physical and psychological stress | 2022 | Varanoske, AN and McClung, HL and Sepowitz, JJ and Halagarda, CJ and Farina, EK and Berryman, CE and Lieberman, HR and McClung, JP and Pasiakos, SM and Karl, JP | Excluded | The article was excluded based on title and abstract screening as it did not meet the inclusion and exclusion criteria. |
| Effects of Exercise Training during Advanced Maternal Age on the Cognitive Function of Offspring | 2022 | Kim, TW and Park, SS and Park, HS | Excluded | The article was excluded based on title and abstract screening as it did not meet the inclusion and exclusion criteria. |
| The effect of swimming exercise on lipid peroxidation in the rat brain, liver and heart. | 2003 | Turgut, G. and Demir, S. and Genç, O. and Karabulut, I. and Akalin, N. | Included |  |
| High Protein Diet Induces Oxidative Stress in Rat Cerebral Cortex and Hypothalamus | 2019 | Zebrowska, E and Maciejczyk, M and Zendzian-Piotrowska, M and Zalewska, A and Chabowski, A | Excluded | The article was excluded based on title and abstract screening as it did not meet the inclusion and exclusion criteria. |
| Effects of exogenous taurine supplementation on the growth, antioxidant capacity, intestine immunity, and resistance against <i>Streptococcus agalactiae</i> in juvenile golden pompano (<i>Trachinotus ovatus</i>) fed with a low-fishmeal diet | 2022 | Liu, JX and Guo, HY and Zhu, KC and Liu, BS and Zhang, N and Zhang, DC | Excluded | The article was excluded based on title and abstract screening as it did not meet the inclusion and exclusion criteria. |
| Strength exercise suppresses STZ-induced spatial memory impairment and modulates BDNF/ERK-CAMKII/CREB signalling pathway in the hippocampus of mice | 2020 | Martini, F and Leite, MR and Rosa, SG and Klann, IP and Nogueira, CW | Excluded | The article was excluded based on title and abstract screening as it did not meet the inclusion and exclusion criteria. |
| Anti-apoptotic Effects of Red Ginseng on Oxidative Stress Induced by Hydrogen Peroxide in SK-N-SH Cells | 2010 | Kim, EH and Lee, MJ and Kim, IH and Pyo, S and Choi, KT and Rhee, DK | Excluded | The article was excluded based on title and abstract screening as it did not meet the inclusion and exclusion criteria. |
| Attenuating heatstroke-induced acute lung inflammation, edema, and injury in rats by exercise preconditioning | 2013 | Chang, CH and Kao, CH and Chio, CC and Lin, CH and Lin, MT and Chang, CP | Excluded | The article was excluded based on title and abstract screening as it did not meet the inclusion and exclusion criteria. |
| <i>Mentha rotundifolia</i> (L.) Huds. and <i>Salvia officinalis</i> L. hydrosols mitigate aging related comorbidities in rats | 2024 | Boualam, K and Ibork, H and Lahboub, Z and Sobeh, M and Taghzouti, K | Excluded | The article was excluded based on title and abstract screening as it did not meet the inclusion and exclusion criteria. |
| Swimming exercise prevents behavioural disturbances induced by an intracerebroventricular injection of amyloid-beta(1-42) peptide through modulation of cytokine/NF-kappaB pathway and indoleamine-2,3-dioxygenase in mouse brain | 2017 | Souza, LC and Jesse, CR and Del Fabbro, L and de Gomes, MG and Goes, ATR and Borges, C and Luchese, C and Pereira, AAM and Boeira, SP | Excluded | The article was excluded based on title and abstract screening as it did not meet the inclusion and exclusion criteria. |
| Heat and dehydration induced oxidative damage and antioxidant defenses following incubator heat stress and a simulated heat wave in wild caught four-striped field mice <i>Rhabdomys dilectus</i> | 2020 | Jacobs, PJ and Oosthuizen, MK and Mitchell, C and Blount, JD and Bennett, NC | Excluded | The article was excluded based on title and abstract screening as it did not meet the inclusion and exclusion criteria. |
| Protection Against Oxidative Stress Caused by Intermittent Cold Exposure by Combined Supplementation with Vitamin E and C in the Aging Rat Hypothalamus | 2013 | Manjula, KR and Subramanyam, MVV and Devi, SA | Excluded | The article was excluded based on title and abstract screening as it did not meet the inclusion and exclusion criteria. |
| Exercise and Dementia | 2020 | Gholamnezhad, Z and Boskabady, MH and Jahangiri, Z | Excluded | The article was excluded based on title and abstract screening as it did not meet the inclusion and exclusion criteria. |
| Voluntary exercise delays progressive deterioration of markers of metabolism and behavior in a mouse model of Parkinson's disease | 2019 | Lai, JH and Chen, KY and Wu, JCC and Olson, L and Brené, S and Huang, CZ and Chen, YH and Kang, SJ and Ma, KH and Hoffer, BJ and Hsieh, TH and Chiang, YH | Excluded | The article was excluded based on title and abstract screening as it did not meet the inclusion and exclusion criteria. |
| The effect of voluntary wheel running on the antioxidant status is dependent on sociability conditions | 2020 | Cunha, M.P. and Machado, D.G. and Mancini, G. and Glaser, V. and de Paula Martins, R. and de Bem, A.F. and Latini, A. and Dafre, A.L. and Rodrigues, A.L.S. | Included |  |
| Ferulic acid as a therapeutic agent in depression: Evidence from preclinical studies | 2023 | Dong, XY and Zhao, DX | Excluded | The article was excluded based on title and abstract screening as it did not meet the inclusion and exclusion criteria. |
| Exercise offers anxiolytic potential: A role for stress and brain noradrenergic-galaninergic mechanisms | 2012 | Sciolino, NR and Holmes, PV | Excluded | The article was excluded based on title and abstract screening as it did not meet the inclusion and exclusion criteria. |
| Regulatory role of cytokines on etiology of depression in animal models: their biological mechanisms and clinical implication with physical exercise | 2022 | Park, HJ and Rhie, SJ and Shim, I | Excluded | The article was excluded based on title and abstract screening as it did not meet the inclusion and exclusion criteria. |
| Simulated weightlessness to induce chronic hypoactivity of brain norepinephrine for exercise and stress studies | 1997 | Sothmann, M and Kastello, GK | Excluded | The article was excluded based on title and abstract screening as it did not meet the inclusion and exclusion criteria. |
| Garlic as an anti-fatigue agent | 2007 | Morihara, N and Nishihama, T and Ushijima, M and Ide, N and Takeda, H and Hayama, M | Excluded | The article was excluded based on title and abstract screening as it did not meet the inclusion and exclusion criteria. |
| The effects of exercise treatment on learning and memory ability, and cognitive performance in diet-induced prediabetes animals | 2020 | Luvuno, M and Khathi, A and Mabandla, MV | Excluded | The article was excluded based on title and abstract screening as it did not meet the inclusion and exclusion criteria. |
| Exercise, learned helplessness, and the stress-resistant brain | 2008 | Greenwood, BN and Fleshner, M | Excluded | The article was excluded based on title and abstract screening as it did not meet the inclusion and exclusion criteria. |
| The effects of post-extinction exercise on cocaine-primed and stress-induced reinstatement of cocaine seeking in rats | 2015 | Ogbonmwan, YE and Schroeder, JP and Holmes, PV and Weinshenker, D | Excluded | The article was excluded based on title and abstract screening as it did not meet the inclusion and exclusion criteria. |
| Metabolic syndrome: Aggression control mechanisms gone out of control | 2010 | Belsare, PV and Watve, MG and Ghaskadbi, SS and Bhat, DS and Yajnik, CS and Jog, M | Excluded | The article was excluded based on title and abstract screening as it did not meet the inclusion and exclusion criteria. |
| Neuroprotector Effect of Daily 8-Minutes of High-Intensity Interval Training in Rat Aβ<sub>1-42</sub> Alzheimer Disease Model | 2020 | Vasconcelos, FSL and da Rocha-e-Silva, RC and Martins, JER and Godinho, WDN and da Costa, VV and Ribeiro, JKC and da Silva, CA and Ceccatto, VM and Soares, PM and Evangelista, JSAM | Excluded | The article was excluded based on title and abstract screening as it did not meet the inclusion and exclusion criteria. |
| Regular voluntary exercise reduces anxiety-related behaviour and impulsiveness in mice | 2004 | Binder, E and Droste, SK and Ohl, F and Reul, JMHM | Excluded | The article was excluded based on title and abstract screening as it did not meet the inclusion and exclusion criteria. |
| Implications of Physical Exercise on Episodic Memory and Anxiety: The Role of the Serotonergic System | 2023 | Illesca-Matus, R and Ardiles, NM and Munoz, F and Moya, PR | Excluded | The article was excluded based on title and abstract screening as it did not meet the inclusion and exclusion criteria. |
| Studies on the anti-fatigue activities of <i>Irpex lacteus</i> polysaccharide-enriched extract in mouse model | 2019 | Wang, J and Li, CL and Hu, WJ and Li, LZ and Cai, GS and Liu, Y and Wang, D | Excluded | The article was excluded based on title and abstract screening as it did not meet the inclusion and exclusion criteria. |
| Determination of dose- and time-dependent hepatotoxicity and apoptosis of Lanthanum oxide nanoparticles in female Swiss albino mice | 2024 | Alyami, NM and Alobadi, H and Maodaa, S and Alothman, NS and Almukhlafi, H and Yaseen, KN and Alnakhli, ZA and Alshiban, NM and Elnagar, DM and Rady, A and Alharthi, WA and Almetari, B and Almeer, R and Alarifi, S and Ali, D | Excluded | The article was excluded based on title and abstract screening as it did not meet the inclusion and exclusion criteria. |
| Protective effects of niacin following high fat rich diet: an in-vivo and in-silico study | 2023 | Samad, N and Manzoor, N and Batool, A and Noor, A and Khaliq, S and Aurangzeb, S and Bhatti, SA and Imran, I | Excluded | The article was excluded based on title and abstract screening as it did not meet the inclusion and exclusion criteria. |
| Long-Term Moderate Exercise Rescues Age-Related Decline in Hippocampal Neuronal Complexity and Memory | 2018 | Tsai, SF and Ku, NW and Wang, TF and Yang, YH and Shih, YH and Wu, SY and Lee, CW and Yu, M and Yang, TT and Kuo, YM | Excluded | The article was excluded based on title and abstract screening as it did not meet the inclusion and exclusion criteria. |
| Fish oil supplementation and physical exercise program: Distinct effects on different memory tasks | 2013 | Rachetti, ALF and Arida, RM and Patti, CL and Zanin, KA and Fernades-Santos, L and Frussa, R and da Silva, SG and Scorza, FA and Cysneiros, RM | Excluded | The article was excluded based on title and abstract screening as it did not meet the inclusion and exclusion criteria. |
| Possible GABAergic Modulation in the Protective Effect of Allopregnanolone on Sleep Deprivation-Induced Anxiety-Like Behavior and Oxidative Damage in Mice | 2008 | Singh, A and Kumar, A | Excluded | The article was excluded based on title and abstract screening as it did not meet the inclusion and exclusion criteria. |
| The intergenerational effects of parental physical activity on offspring brain and neurocognition in humans: A scoping review | 2022 | Valkenborghs, SR and Dent, PC and Stillman, CM | Excluded | The article was excluded based on title and abstract screening as it did not meet the inclusion and exclusion criteria. |
| Swimming exercise reduces oxidative stress and liver damage indices of male rats exposed to electromagnetic radiation | 2023 | Amiri, H and Shabkhiz, F and Pournemati, P and Quchan, AHSK and Fard, RZ | Excluded | The article was excluded based on title and abstract screening as it did not meet the inclusion and exclusion criteria. |
| Dietary Vitamin C and E Modulates Antioxidant Levels in Blood, Brain, Liver, Muscle, and Testes in Diabetic Aged Rats | 2011 | Naziroglu, M and Butterworth, PJ and Sonmez, TT | Excluded | The article was excluded based on title and abstract screening as it did not meet the inclusion and exclusion criteria. |
| Possible involvement of GABAergic mechanism in protective effect of melatonin against sleep deprivation-induced behaviour modification and oxidative damage in mice | 2009 | Kumar, A and Singh, A | Excluded | The article was excluded based on title and abstract screening as it did not meet the inclusion and exclusion criteria. |
| Chronic treadmill running does not enhance mitochondrial oxidative capacity in the cortex or striatum | 2015 | Herbst, EAF and Roussakis, C and Matravadia, S and Holloway, GP | Excluded | The article was excluded based on title and abstract screening as it did not meet the inclusion and exclusion criteria. |
| Influence of physical activity on addiction parameters of rats exposed to amphetamine which were previously supplemented with hydrogenated vegetable fat | 2017 | Segat, HJ and Barcelos, RCS and Metz, VG and Rosa, HZ and Roversi, K and Antoniazzi, CTD and Vey, LT and Kronbauer, M and Veit, JC and Piccolo, JC and Emanuelli, T and Burger, ME | Excluded | The article was excluded based on title and abstract screening as it did not meet the inclusion and exclusion criteria. |
| <i>Drosophila melanogaster</i> as a model to study age and sex differences in brain injury and neurodegeneration after mild head trauma | 2023 | Ye, CT and Behnke, JA and Hardin, KR and Zheng, JQ | Excluded | The article was excluded based on title and abstract screening as it did not meet the inclusion and exclusion criteria. |
| Intermittent fasting and cognitive performance-Targeting BDNF as potential strategy to optimise brain health | 2022 | Seidler, K and Barrow, M | Excluded | The article was excluded based on title and abstract screening as it did not meet the inclusion and exclusion criteria. |
| Effects of Two Training Programs on Transcriptional Levels of Neurotrophins and Glial Cells Population in Hippocampus of Experimental Multiple Sclerosis | 2018 | Naghibzadeh, M and Ranjbar, R and Tabandeh, MR and Habibi, A | Excluded | The article was excluded based on title and abstract screening as it did not meet the inclusion and exclusion criteria. |
| Interaction of Brain-derived Neurotrophic Factor, Exercise, and Fear Extinction: Implications for Post-traumatic Stress Disorder | 2024 | Antolasic, EJ and Jaehne, EJ and van den Buuse, M | Excluded | The article was excluded based on title and abstract screening as it did not meet the inclusion and exclusion criteria. |
| Melatonin as a chronobiotic/cytoprotector: its role in healthy aging | 2019 | Cardinali, DP | Excluded | The article was excluded based on title and abstract screening as it did not meet the inclusion and exclusion criteria. |
| Forced treadmill running modifies gut microbiota with alleviations of cognitive impairment and Alzheimer?s disease pathology in 3xTg-AD mice | 2023 | Jin, YY and Kim, T and Kang, H | Excluded | The article was excluded based on title and abstract screening as it did not meet the inclusion and exclusion criteria. |
| The impact of regional astrocyte interferon-γ signaling during chronic autoimmunity: a novel role for the immunoproteasome | 2020 | Smith, BC and Sinyuk, M and Jenkins, JE and Psenicka, MW and Williams, JL | Excluded | The article was excluded based on title and abstract screening as it did not meet the inclusion and exclusion criteria. |
| Voluntary physical exercise protects against behavioral and endocrine reactivity to social and environmental stressors in the prairie vole | 2018 | Watanasriyakul, WT and Wardwell, J and McNeal, N and Schultz, R and Woodbury, M and Dagner, A and Cox, M and Grippo, AJ | Excluded | The article was excluded based on title and abstract screening as it did not meet the inclusion and exclusion criteria. |
| May Exercise Prevent Addiction? | 2011 | Fontes-Ribeiro, CA and Marques, E and Pereira, FC and Silva, AP and Macedo, TRA | Excluded | The article was excluded based on title and abstract screening as it did not meet the inclusion and exclusion criteria. |
| Non-invasive assessment of blood-brain barrier (BBB) permeability using a gamma camera to detect <SUP>99</SUP>technetium-gluceptate extravasation in rat brain | 2001 | Esposito, P and Jacobson, S and Connolly, R and Gheorghe, D and Theoharides, TC | Excluded | The article was excluded based on title and abstract screening as it did not meet the inclusion and exclusion criteria. |
| DIHYDROERGOCRISTINE - REVIEW OF THE PHARMACOLOGY AND TOXICOLOGY | 1992 | COPPI, G | Excluded | The article was excluded based on title and abstract screening as it did not meet the inclusion and exclusion criteria. |
| Endurance training effects on neurotransmitter release in rat striatum: An in vivo microdialysis study | 1997 | Meeusen, R and Smolders, I and Saare, S and DeMeirleir, K and Keizer, H and Serneels, M and Ebinger, G and Michotte, Y | Excluded | The article was excluded based on title and abstract screening as it did not meet the inclusion and exclusion criteria. |
| Nonpharmacological treatment options for Alzheimer's disease: from animal testing to clinical studies | 2020 | Türkez, H and Arslan, ME and Di Stefano, A and Cacciatore, I and Mardinoglu, A | Excluded | The article was excluded based on title and abstract screening as it did not meet the inclusion and exclusion criteria. |
| Exercise Enhances the Behavioral Responses to Acute Stress in an Animal Model of PTSD | 2015 | Hoffman, JR and Ostfeld, I and Kaplan, Z and Zohar, J and Cohen, H | Excluded | The article was excluded based on title and abstract screening as it did not meet the inclusion and exclusion criteria. |
| Exercise-induced plasticity of AMPA-type glutamate receptor subunits in the rat brain | 2010 | Real, CC and Ferreira, AFB and Hernandes, MS and Britto, LRG and Pires, RS | Excluded | The article was excluded based on title and abstract screening as it did not meet the inclusion and exclusion criteria. |
| Stress During the Pre-pubertal Period Leads to Long-Term Diet-Dependent Changes in Anxiety-Like Behavior and in Oxidative Stress Parameters in Male Adult Rats | 2013 | Arcego, DM and Krolow, R and Lampert, C and Noschang, C and Pettenuzzo, LF and Marcolin, ML and Toniazzo, AP and Dalmaz, C | Excluded | The article was excluded based on title and abstract screening as it did not meet the inclusion and exclusion criteria. |
| Resistance exercise improves hippocampus-dependent memory | 2012 | Cassilhas, RC and Lee, KS and Venåncio, DP and Oliveira, MGM and Tufik, S and de Mello, MT | Excluded | The article was excluded based on title and abstract screening as it did not meet the inclusion and exclusion criteria. |
| EFFECT OF PRETREATMENT WITH VITAMIN-E OR DIAZEPAM ON BRAIN METABOLISM OF STRESSED RATS | 1993 | SHAHEEN, AA and HAMDY, MA and KHEIRELDIN, AA and LINDSTROM, P and ELFATTAH, AAA | Excluded | The article was excluded based on title and abstract screening as it did not meet the inclusion and exclusion criteria. |
| Therapeutic strategies for targeting excessive central sympathetic activation in human hypertension | 2010 | Fisher, JP and Fadel, PJ | Excluded | The article was excluded based on title and abstract screening as it did not meet the inclusion and exclusion criteria. |
| Voluntary wheel running: a useful rodent model for investigating mechanisms of stress robustness and exercise motivation | 2019 | Greenwood, BN and Fleshner, M | Excluded | The article was excluded based on title and abstract screening as it did not meet the inclusion and exclusion criteria. |
| Influence of dietary selenium supplementation and exercise on thiol-containing enzymes in mice | 2003 | Soares, JCM and Folmer, V and Rocha, JBT | Excluded | The article was excluded based on title and abstract screening as it did not meet the inclusion and exclusion criteria. |
| Impaired thermoregulation in spontaneously hypertensive rats during physical exercise is related to reduced hypothalamic neuronal activation | 2020 | Drummond, LR and Campos, HO and Lima, PMD and Fonseca, CG and Kunstetter, AC and Rodrigues, QT and Szawka, RE and Natali, AJ and Prímola-Gomes, TN and Wanner, SP and Coimbra, CC | Excluded | The article was excluded based on title and abstract screening as it did not meet the inclusion and exclusion criteria. |
| The Potential Role of Previous Physical Exercise Program to Reduce Seizure Susceptibility: A Systematic Review and Meta-Analysis of Animal Studies | 2021 | Arida, RM and Passos, AA and Graciani, AL and Brogin, JAF and Ribeiro, MDL and Faber, J and Gutierre, RC and Teixeira-Machado, L | Excluded | The article was excluded based on title and abstract screening as it did not meet the inclusion and exclusion criteria. |
| Neuroprotective Effects of Treadmill Exercise in Hippocampus of Ovariectomized and Diabetic Rats | 2022 | Zare, Z and Zarbakhsh, S and Tehrani, M and Mohammadi, M | Excluded | The article was excluded based on title and abstract screening as it did not meet the inclusion and exclusion criteria. |
| Brain mitochondrial dysfunction in aging, neurodegeneration, and Parkinson's disease | 2010 | Navarro, A and Boveris, A | Excluded | The article was excluded based on title and abstract screening as it did not meet the inclusion and exclusion criteria. |
| Alpha-lipoic acid does not alter stress protein response to acute exercise in diabetic brain | 2010 | Lappalainen, J and Lappalainen, Z and Oksala, NKJ and Laaksonen, DE and Khanna, S and Sen, CK and Atalay, M | Excluded | The article was excluded based on title and abstract screening as it did not meet the inclusion and exclusion criteria. |
| Running Reduces Uncontrollable Stress-Evoked Serotonin and Potentiates Stress-Evoked Dopamine Concentrations in the Rat Dorsal Striatum | 2015 | Clark, PJ and Amat, J and McConnell, SO and Ghasem, PR and Greenwood, BN and Maier, SF and Fleshner, M | Excluded | The article was excluded based on title and abstract screening as it did not meet the inclusion and exclusion criteria. |
| VEGF is necessary for exercise-induced adult hippocampal neurogenesis | 2003 | Fabel, K and Fabel, K and Tam, B and Kaufer, D and Baiker, A and Simmons, N and Kuo, CJ and Palmer, TD | Excluded | The article was excluded based on title and abstract screening as it did not meet the inclusion and exclusion criteria. |
| Exercise, antidepressant treatment, and BDNF mRNA expression in the aging brain | 2004 | Garza, AA and Ha, TG and Garcia, C and Chen, MJ and Russo-Neustadt, AA | Excluded | The article was excluded based on title and abstract screening as it did not meet the inclusion and exclusion criteria. |
| Swimming exercise attenuates psychological dependence and voluntary methamphetamine consumption in methamphetamine withdrawn rats | 2016 | Damghani, F and Bigdeli, I and Miladi-Gorji, H and Fadaei, A | Excluded | The article was excluded based on title and abstract screening as it did not meet the inclusion and exclusion criteria. |
| Does calorie restriction improve cognition? | 2020 | Dias, IR and Santos, CD and Magalhaes, CODE and de Oliveira, LRS and Peixoto, MFD and De Sousa, RAL and Cassilhas, RC | Excluded | The article was excluded based on title and abstract screening as it did not meet the inclusion and exclusion criteria. |
| Brain-derived neurotrophic factor as a regulator of systemic and brain energy metabolism and cardiovascular health | 2012 | Rothman, SM and Griffioen, KJ and Wan, RQ and Mattson, MP | Excluded | The article was excluded based on title and abstract screening as it did not meet the inclusion and exclusion criteria. |
| Effect of stress on the rehabilitation performance of rats with repetitive mild fluid percussion-induced traumatic brain injuries | 2024 | Wang, YL and Chen, CC and Chang, CP | Excluded | The article was excluded based on title and abstract screening as it did not meet the inclusion and exclusion criteria. |
| Selective Nitric Oxide Synthase Inhibitor 7-Nitroindazole Protects against Cocaine-Induced Oxidative Stress in Rat Brain | 2015 | Vitcheva, V and Simeonova, R and Kondeva-Burdina, M and Mitcheva, M | Excluded | The article was excluded based on title and abstract screening as it did not meet the inclusion and exclusion criteria. |
| Melatonin ameliorates cognitive memory by regulation of cAMP-response element-binding protein expression and the anti-inflammatory response in a rat model of post-traumatic stress disorder | 2018 | Lee, B and Shim, I and Lee, H and Hahm, DH | Excluded | The article was excluded based on title and abstract screening as it did not meet the inclusion and exclusion criteria. |
| Electromagnetic Fields Modify Redox Balance in the Rat Gastrointestinal Tract | 2021 | Sieron, K and Knapik, K and Onik, G and Romuk, E and Birkner, E and Kwiatek, S and Sieron, A | Excluded | The article was excluded based on title and abstract screening as it did not meet the inclusion and exclusion criteria. |
| Asiatic acid ameliorates life and health span in fruit fly | 2023 | Liu, XC and Huan, P and Ming, L | Excluded | The article was excluded based on title and abstract screening as it did not meet the inclusion and exclusion criteria. |
| Reducing Allostatic Load in Depression and Anxiety Disorders: Physical Activity and Yoga Practice as Add-On Therapies | 2020 | D'Alessio, L and Korman, GP and Sarudiansky, M and Guelman, LR and Scévola, L and Pastore, A and Obregón, A and Roldán, EJA | Excluded | The article was excluded based on title and abstract screening as it did not meet the inclusion and exclusion criteria. |
| A review of interventions against fetal alcohol spectrum disorder targeting oxidative stress | 2018 | Zhang, YP and Wang, HX and Li, Y and Peng, Y | Excluded | The article was excluded based on title and abstract screening as it did not meet the inclusion and exclusion criteria. |
| From molecular biology to anti-aging cognitive-behavioral practices - The pioneering research of Walter Pierpaoli on the pineal and bone marrow foreshadows the contemporary revolution in stem cell and regenerative biology | 2005 | Bushell, WC | Excluded | The article was excluded based on title and abstract screening as it did not meet the inclusion and exclusion criteria. |
| Amelioration of ischaemia reperfusion-induced cerebral injury in mice by liposomes containing <i>Allium cepa</i> fraction administered intranasally | 2018 | Singh, V and Krishan, P and Shri, R | Excluded | The article was excluded based on title and abstract screening as it did not meet the inclusion and exclusion criteria. |
| Physical exercise prevents mice from L-Kynurenine-induced depression-like behavior | 2020 | Su, CH and Chuang, HC and Hong, CJ | Excluded | The article was excluded based on title and abstract screening as it did not meet the inclusion and exclusion criteria. |
| Augmenting trauma-focused psychotherapy for post-traumatic stress disorder with brief aerobic exercise in Australia: a randomised clinical trial | 2023 | Bryant, RA and Dawson, KS and Azevedo, S and Yadav, S and Cahill, C and Kenny, L and Maccallum, F and Tran, J and Rawson, N and Tockar, J and Garber, B and Keyan, D | Excluded | The article was excluded based on title and abstract screening as it did not meet the inclusion and exclusion criteria. |
| The effect of various morphine weaning regimens on the sequelae of opioid tolerance involving physical dependency, anxiety and hippocampus cell neurodegeneration in rats | 2015 | Motaghinejad, M and Karimian, SM and Motaghinejad, O and Shabab, B and Asadighaleni, M and Fatima, S | Excluded | The article was excluded based on title and abstract screening as it did not meet the inclusion and exclusion criteria. |
| EEG Brain Activity in Dynamic Health Qigong Training: Same Effects for Mental Practice and Physical Training? | 2017 | Henz, D and Schöllhorn, WI | Excluded | The article was excluded based on title and abstract screening as it did not meet the inclusion and exclusion criteria. |
| Anti-fatigue activity of an arabinan-rich pectin from acerola (<i>Malpighia emarginata</i>) | 2018 | Klosterhoff, RR and Kanazawa, LKS and Furlanetto, ALDM and Peixoto, JVC and Corso, CR and Adami, ER and Iacomini, M and Fogaça, RTH and Acco, A and Cadena, SMSC and Andreatini, R and Cordeiro, LMC | Excluded | The article was excluded based on title and abstract screening as it did not meet the inclusion and exclusion criteria. |
| The combined effects of exercise and foods in preventing neurological and cognitive disorders | 2011 | Gomez-Pinilla, F | Excluded | The article was excluded based on title and abstract screening as it did not meet the inclusion and exclusion criteria. |
| Treatment Options for Motor and Non-Motor Symptoms of Parkinson's Disease | 2021 | Church, FC | Excluded | The article was excluded based on title and abstract screening as it did not meet the inclusion and exclusion criteria. |
| The hormesis principle of neuroplasticity and neuroprotection | 2024 | Mattson, MP and Leak, RK | Excluded | The article was excluded based on title and abstract screening as it did not meet the inclusion and exclusion criteria. |
| The Nature of Destructive and Compensator-Restorative Processes in the Rat Heart Myocardium after a Traumatic Brain Injury Performing Various Physical Exercises | 2021 | Esfehani, M and Raginov, IS and Vakhitov, IK and Izosimova, AV and Vakhitov, BI | Excluded | The article was excluded based on title and abstract screening as it did not meet the inclusion and exclusion criteria. |
| Anxiolytic-like effects of treadmill exercise on an animal model of post-traumatic stress disorder and its mechanism | 2020 | Zhang, J and Xue, R and Li, YF and Zhang, YZ and Wei, HW | Excluded | The article was excluded based on title and abstract screening as it did not meet the inclusion and exclusion criteria. |
| Combined effects of <i>Rhodiola rosea</i> and caffeine supplementation on aerobic endurance and muscle explosiveness: a synergistic approach | 2024 | Yun, HZ and Lu, B and Su, WB and Wang, JJ and Zheng, J and Wang, J and Wang, ZL and Li, Y and Sun, YW and Liu, C | Excluded | The article was excluded based on title and abstract screening as it did not meet the inclusion and exclusion criteria. |
| Interventions that Improve Body and Brain Bioenergetics for Parkinson's Disease Risk Reduction and Therapy | 2014 | Mattson, MP | Excluded | The article was excluded based on title and abstract screening as it did not meet the inclusion and exclusion criteria. |
| Differential behavioral and neurochemical effects of exercise, reboxetine and citalopram with the forced swim test | 2009 | Arunrut, T and Alejandre, H and Chen, M and Cha, J and Russo-Neustadt, A | Excluded | The article was excluded based on title and abstract screening as it did not meet the inclusion and exclusion criteria. |
| Effect of exercise-induced neurogenesis on cognitive function deficit in a rat model of vascular dementia | 2016 | Choi, DH and Lee, KH and Lee, J | Excluded | The article was excluded based on title and abstract screening as it did not meet the inclusion and exclusion criteria. |
| Effect of low-intensity treadmill exercise on behavioural measures and hippocampal parvalbumin immunoreactivity in the rat | 2013 | Nguyen, JCD and Killcross, AS and Jenkins, TA | Excluded | The article was excluded based on title and abstract screening as it did not meet the inclusion and exclusion criteria. |
| Effect of pulsed infrared laser radiation on DNA synthesis in tissues of intact rats and during strenuous physical exercise | 1995 | Zubkova, SM and Mikhailik, LV | Excluded | The article was excluded based on title and abstract screening as it did not meet the inclusion and exclusion criteria. |
| Baseline and stress-induced plasma corticosterone concentrations of mice selectively bred for high voluntary wheel running | 2007 | Malisch, JL and Saltzman, W and Gomes, FR and Rezende, EL and Jeske, DR and Garland, T | Excluded | The article was excluded based on title and abstract screening as it did not meet the inclusion and exclusion criteria. |
| Three weeks voluntary running wheel exercise increases endoplasmic reticulum stress in the brain of mice | 2010 | Kim, Y and Park, M and Boghossian, S and York, DA | Excluded | The article was excluded based on title and abstract screening as it did not meet the inclusion and exclusion criteria. |
| Responses of serum corticosterone and corticosteroid-binding globulin to acute and prolonged stress in the rat | 1999 | Tinnikov, AA | Excluded | The article was excluded based on title and abstract screening as it did not meet the inclusion and exclusion criteria. |
| Berberine Ameliorate Haloperidol and 3-Nitropropionic Acid-Induced Neurotoxicity in Rats | 2022 | Kadir, A and Singh, J and Rahi, V and Kumar, P | Excluded | The article was excluded based on title and abstract screening as it did not meet the inclusion and exclusion criteria. |
| Cross-fostering selectively bred High Runner mice affects adult body mass but not voluntary exercise | 2021 | Cadney, MD and Schwartz, NE and McNamara, MP and Schmill, MP and Castro, AA and Hillis, DA and Garland, T | Excluded | The article was excluded based on title and abstract screening as it did not meet the inclusion and exclusion criteria. |
| The Biological Role of Carnosine and Its Possible Applications in Medicine | 2013 | Budzen, S and Rymaszewska, J | Excluded | The article was excluded based on title and abstract screening as it did not meet the inclusion and exclusion criteria. |
| The Application of Nanotechnology for the Diagnosis and Treatment of Brain Diseases and Disorders | 2021 | Ngowi, EE and Wang, YZ and Qian, L and Helmy, YASH and Anyomi, B and Li, T and Zheng, M and Jiang, ES and Duan, SF and Wei, JS and Wu, DD and Ji, XY | Excluded | The article was excluded based on title and abstract screening as it did not meet the inclusion and exclusion criteria. |
| An Understated Comorbidity: The Impact of Homelessness on Traumatic Brain Injury | 2023 | Monsour, M and Lee, JY and Borlongan, CV | Excluded | The article was excluded based on title and abstract screening as it did not meet the inclusion and exclusion criteria. |
| Regular Low-Intensity Exercise Prevents Cognitive Decline and a Depressive-Like State Induced by Physical Inactivity in Mice: A New Physical Inactivity Experiment Model | 2022 | Kim, J and Park, J and Mikami, T | Excluded | The article was excluded based on title and abstract screening as it did not meet the inclusion and exclusion criteria. |
| The protective role of exercise against age-related neurodegeneration | 2022 | Sujkowski, A and Hong, LK and Wessells, RJ and Todi, SV | Excluded | The article was excluded based on title and abstract screening as it did not meet the inclusion and exclusion criteria. |
| The indirect NMDAR antagonist acamprosate induces postischemic neurologic recovery associated with sustained neuroprotection and neuroregeneration | 2015 | Doeppner, TR and Pehlke, JR and Kaltwasser, B and Schlechter, J and Kilic, E and Bähr, M and Hermann, DM | Excluded | The article was excluded based on title and abstract screening as it did not meet the inclusion and exclusion criteria. |
| Freewheel running prevents learned helplessness/behavioral depression: Role of dorsal raphe serotonergic neurons | 2003 | Greenwood, BN and Foley, TE and Day, HEW and Campisi, J and Hammack, SH and Campeau, S and Maier, SF and Fleshner, M | Excluded | The article was excluded based on title and abstract screening as it did not meet the inclusion and exclusion criteria. |
| Small-Molecule Anticonvulsant Agents with Potent In Vitro Neuroprotection and Favorable Drug-Like Properties | 2014 | Smith, GR and Brenneman, DE and Zhang, Y and Du, YM and Reitz, AB | Excluded | The article was excluded based on title and abstract screening as it did not meet the inclusion and exclusion criteria. |
| Physical Exercise and Neuroinflammation in Major Depressive Disorder | 2019 | Ignácio, ZM and da Silva, RS and Plissari, ME and Quevedo, J and Réus, GZ | Excluded | The article was excluded based on title and abstract screening as it did not meet the inclusion and exclusion criteria. |
| Can coconut oil and treadmill exercise during the critical period of brain development ameliorate stress-related effects on anxiety-like behavior and episodic-like memory in young rats? | 2018 | da Silva, DD and Tavares, MG and do Nascimento, CKB and Lira, EC and dos Santos, AA and Maia, LMSD and Hornsby, MBD | Excluded | The article was excluded based on title and abstract screening as it did not meet the inclusion and exclusion criteria. |
| Impact of Exercise and Vitamin B<sub>1</sub> Intake on Hippocampal Brain-Derived Neurotrophic Factor and Spatial Memory Performance in a Rat Model of Stress | 2015 | Dief, AE and Samy, DM and Dowedar, FI | Excluded | The article was excluded based on title and abstract screening as it did not meet the inclusion and exclusion criteria. |
| Circadian rhythms and depression: effects of exercise in an animal model | 1999 | Solberg, LC and Horton, TH and Turek, FW | Excluded | The article was excluded based on title and abstract screening as it did not meet the inclusion and exclusion criteria. |
| Caloric Restriction in Humans: Impact on Physiological, Psychological, and Behavioral Outcomes | 2011 | Redman, LM and Ravussin, E | Excluded | The article was excluded based on title and abstract screening as it did not meet the inclusion and exclusion criteria. |
| Exercise training improves cardiac function and attenuates arrhythmia in CPVT mice | 2012 | Kurtzwald-Josefson, E and Hochhauser, E and Katz, G and Porat, E and Seidman, JG and Seidman, CE and Chepurko, Y and Shainberg, A and Eldar, M and Arad, M | Excluded | The article was excluded based on title and abstract screening as it did not meet the inclusion and exclusion criteria. |
| Nutritional and exercise-based interventions in the treatment of amyotrophic lateral sclerosis | 2009 | Patel, BP and Hamadeh, MJ | Excluded | The article was excluded based on title and abstract screening as it did not meet the inclusion and exclusion criteria. |
| Voluntary Wheel Running Reduces Vesicle Development in an Endometriosis Animal Model Through Modulation of Immune Parameters | 2022 | Appleyard, CB and Cruz, ML and Velazquez-Cruz, J and Rivera-Mendez, RM and Jimenez-Garcia, JG and Rivera, LA and Mendez-Casillas, MD and Flores, I and Al-Nakkash, L and Chompre, G | Excluded | The article was excluded based on title and abstract screening as it did not meet the inclusion and exclusion criteria. |
| Brain hyperthermia as physiological and pathological phenomena | 2005 | Kiyatkin, EA | Excluded | The article was excluded based on title and abstract screening as it did not meet the inclusion and exclusion criteria. |
| Adolescent swimming exercise following maternal valproic acid treatment improves cognition and reduces stress-related symptoms in offspring mice: Role of sex and brain cytokines | 2023 | Barzegari, A and Mahdirejei, HA and Hanani, M and Esmaeili, MH and Salari, AA | Excluded | The article was excluded based on title and abstract screening as it did not meet the inclusion and exclusion criteria. |
| Physical exercise promotes astrocyte coverage of microvessels in a model of chronic cerebral hypoperfusion | 2020 | Leardini-Tristao, M and Andrade, G and Garcia, C and Reis, PA and Lourenço, M and Moreira, ETS and Lima, FRS and Castro-Faria-Neto, HC and Tibirica, E and Estato, V | Excluded | The article was excluded based on title and abstract screening as it did not meet the inclusion and exclusion criteria. |
| Differential Effects of Voluntary and Forced Exercise on Stress Responses after Traumatic Brain Injury | 2012 | Griesbach, GS and Tio, DL and Vincelli, J and McArthur, DL and Taylor, AN | Excluded | The article was excluded based on title and abstract screening as it did not meet the inclusion and exclusion criteria. |
| Impact of Stress on Epilepsy: Focus on Neuroinflammation-A Mini Review | 2021 | Espinosa-Garcia, C and Zeleke, H and Rojas, A | Excluded | The article was excluded based on title and abstract screening as it did not meet the inclusion and exclusion criteria. |
| Circulating Spexin Is Associated with Body Mass Index and Fat Mass but Not with Physical Activity and Psychological Parameters in Women across a Broad Body Weight Spectrum | 2022 | Suhs, M and Stengel, A and Rudolph, A and Schaper, S and Wölk, E and Kobelt, P and Rose, M and Hofmann, T | Excluded | The article was excluded based on title and abstract screening as it did not meet the inclusion and exclusion criteria. |
| Dandelion (<i>Taraxacum mongolicum</i> Hand.-Mazz.) suppresses the decrease in physical strength and exercise capacity caused by insufficient liver function | 2022 | Zhang, YL and Li, B and Wu, GL | Excluded | The article was excluded based on title and abstract screening as it did not meet the inclusion and exclusion criteria. |
| Exercise alters molecular rhythms in the central nervous system that negatively correlate with depression-like behavior | 2024 | Yu, L and Xue, HN and Li, ZT | Excluded | The article was excluded based on title and abstract screening as it did not meet the inclusion and exclusion criteria. |
| Intranasally administered IGF-1 inhibits spreading depression <i>in vivo</i> | 2017 | Grinberg, YY and Zitzow, LA and Kraig, RP | Excluded | The article was excluded based on title and abstract screening as it did not meet the inclusion and exclusion criteria. |
| Long-term voluntary wheel running does not alter vascular amyloid burden but reduces neuroinflammation in the Tg-SwDI mouse model of cerebral amyloid angiopathy | 2019 | Robison, LS and Popescu, DL and Anderson, ME and Francis, N and Hatfield, J and Sullivan, JK and Beigelman, SI and Xu, F and Anderson, BJ and Van Nostrand, WE and Robinson, JK | Excluded | The article was excluded based on title and abstract screening as it did not meet the inclusion and exclusion criteria. |
| Gradually Increased Training Intensity Benefits Rehabilitation Outcome after Stroke by BDNF Upregulation and Stress Suppression | 2014 | Sun, J and Ke, Z and Yip, SP and Hu, XL and Zheng, XX and Tong, KY | Excluded | The article was excluded based on title and abstract screening as it did not meet the inclusion and exclusion criteria. |
| Physical exercise affects the epigenetic programming of rat brain and modulates the adaptive response evoked by repeated restraint stress | 2016 | Kashimoto, RK and Toffoli, LV and Manfredo, MHF and Volpini, VL and Martins-Pinge, MC and Pelosi, GG and Gomes, MV | Excluded | The article was excluded based on title and abstract screening as it did not meet the inclusion and exclusion criteria. |
| Disturbed Oligodendroglial Maturation Causes Cognitive Dysfunction in Schizophrenia: A New Hypothesis | 2023 | Falkai, P and Rossner, MJ and Raabe, FJ and Wagner, E and Keeser, D and Maurus, I and Roell, L and Chang, EMY and Seitz-Holland, J and Schulze, TG and Schmitt, A | Excluded | The article was excluded based on title and abstract screening as it did not meet the inclusion and exclusion criteria. |
| Brain Aging and Electrophysiological Signaling: Revisiting the Spreading Depression Model | 2019 | Guedes, RCA and Abadie-Guedes, R | Excluded | The article was excluded based on title and abstract screening as it did not meet the inclusion and exclusion criteria. |
| Brain region-dependent gene networks associated with selective breeding for increased voluntary wheel-running behavior | 2018 | Zhang, P and Rhodes, JS and Garland, T and Perez, SD and Southey, BR and Rodriguez-Zas, SL | Excluded | The article was excluded based on title and abstract screening as it did not meet the inclusion and exclusion criteria. |
| Hippocampal mossy fiber sprouting induced by forced and voluntary physical exercise | 2010 | Toscano-Silva, M and da Silva, SG and Scorza, FA and Bonvent, JJ and Cavalheiro, EA and Arida, RM | Excluded | The article was excluded based on title and abstract screening as it did not meet the inclusion and exclusion criteria. |
| ON STRESS | 1991 | WIEGANT, VM | Excluded | The article was excluded based on title and abstract screening as it did not meet the inclusion and exclusion criteria. |
| Light Treatment for Cognitive Improvement Through Multiple Mechanisms | 2023 | Chen, JY and Zhang, LZ and Wang, ZC | Excluded | The article was excluded based on title and abstract screening as it did not meet the inclusion and exclusion criteria. |
| Treadmill exercise induces age and protocol-dependent epigenetic changes in prefrontal cortex of Wistar rats | 2016 | Cechinel, LR and Basso, CG and Bertoldi, K and Schallenberger, B and de Meireles, LCF and Siqueira, IR | Excluded | The article was excluded based on title and abstract screening as it did not meet the inclusion and exclusion criteria. |
| Physical Activity Reduces Epilepsy Incidence: a Retrospective Cohort Study in Swedish Cross-Country Skiers and an Experimental Study in Seizure-Prone Synapsin II Knockout Mice | 2019 | Ahl, M and Avdic, U and Strandberg, MC and Chugh, D and Andersson, E and Hållmarker, U and James, S and Deierborg, T and Ekdahl, CT | Excluded | The article was excluded based on title and abstract screening as it did not meet the inclusion and exclusion criteria. |
| Bioenergetics and synaptic plasticity as potential targets for individualizing treatment for depression | 2018 | Price, JB and Bronars, C and Erhardt, S and Cullen, KR and Schwieler, L and Berk, M and Walder, K and McGee, SL and Frye, MA and Tye, SJ | Excluded | The article was excluded based on title and abstract screening as it did not meet the inclusion and exclusion criteria. |
| Creatine and the Liver: Metabolism and Possible Interactions | 2016 | Barcelos, RP and Stefanello, ST and Mauriz, JL and González-Gallego, J and Soares, FAA | Excluded | The article was excluded based on title and abstract screening as it did not meet the inclusion and exclusion criteria. |
| The capacity for acute exercise to modulate emotional memories: A review of findings and mechanisms | 2019 | Keyan, D and Bryant, RA | Excluded | The article was excluded based on title and abstract screening as it did not meet the inclusion and exclusion criteria. |
| Voluntary wheel running protects against the increase in ethanol consumption induced by social stress in mice | 2020 | Reguilón, MD and Ferrer-Pérez, C and Ballestín, R and Miñarro, J and Rodríguez-Arias, M | Excluded | The article was excluded based on title and abstract screening as it did not meet the inclusion and exclusion criteria. |
| Beneficial Effects of Physical Activity and Crocin Against Adolescent Stress Induced Anxiety or Depressive-Like Symptoms and Dendritic Morphology Remodeling in Prefrontal Cortex in Adult Male Rats | 2019 | Ghalandari-Shamami, M and Nourizade, S and Yousefi, B and Vafaei, AA and Pakdel, R and Rashidy-Pour, A | Excluded | The article was excluded based on title and abstract screening as it did not meet the inclusion and exclusion criteria. |
| Neural Mechanism of Exercise: Neurovascular Responses to Exercise | 2015 | Yuan, TF and Rocha, NBF and Paes, F and Arias-Carrión, O and Machado, S and de Sá, AS | Excluded | The article was excluded based on title and abstract screening as it did not meet the inclusion and exclusion criteria. |
| Early effects of a high-caloric diet and physical exercise on brain volumetry and behavior: a combined MRI and histology study in mice | 2017 | Sack, M and Lenz, JN and Jakovcevski, M and Biedermann, SV and Falfan-Melgoza, C and Deussing, J and Bielohuby, M and Bidlingmaier, M and Pfister, F and Stalla, GK and Sartorius, A and Gass, P and Weber-Fahr, W and Fuss, J and Auer, MK | Excluded | The article was excluded based on title and abstract screening as it did not meet the inclusion and exclusion criteria. |
| Brain norepinephrine changes with simulated weightlessness and relation to exercise training | 1999 | Kastello, GM and Sothmann, MS | Excluded | The article was excluded based on title and abstract screening as it did not meet the inclusion and exclusion criteria. |
| Pentoxifylline prevents post-traumatic stress disorder induced memory impairment | 2018 | Alzoubi, KH and Khabour, OF and Ahmed, M | Excluded | The article was excluded based on title and abstract screening as it did not meet the inclusion and exclusion criteria. |
| Modification of Hippocampal Markers of Synaptic Plasticity by Memantine in Animal Models of Acute and Repeated Restraint Stress: Implications for Memory and Behavior | 2015 | Amin, SN and El-Aidi, AA and Ali, MM and Attia, YM and Rashed, LA | Excluded | The article was excluded based on title and abstract screening as it did not meet the inclusion and exclusion criteria. |
| Parvalbumin interneuron alterations in stress-related mood disorders: A systematic review | 2021 | Perlman, G and Tanti, A and Mechawar, N | Excluded | The article was excluded based on title and abstract screening as it did not meet the inclusion and exclusion criteria. |
| Behavioral and Biochemical Interaction Between Nicotine and Chronic Unpredictable Mild Stress in Mice | 2017 | Biala, G and Pekala, K and Boguszewska-Czubara, A and Michalak, A and Kruk-Slomka, M and Budzynska, B | Excluded | The article was excluded based on title and abstract screening as it did not meet the inclusion and exclusion criteria. |
| Behavioural study of the D-galactose induced aging model in C57BL/6J mice | 2005 | Wei, HF and Li, L and Song, QJ and Ai, HX and Chu, J and Li, W | Excluded | The article was excluded based on title and abstract screening as it did not meet the inclusion and exclusion criteria. |
| Physical Exercise Counteracts Stress-induced Upregulation of Melanin-concentrating Hormone in the Brain and Stress-induced Persisting Anxiety-like Behaviors | 2016 | Kim, TK and Han, PL | Excluded | The article was excluded based on title and abstract screening as it did not meet the inclusion and exclusion criteria. |
| Caffeine-supplemented diet modulates oxidative stress markers and improves locomotor behavior in the lobster cockroach <i>Nauphoeta cinerea</i> | 2018 | da Silva, CS and de Lima, RDG and Elekofehinti, OO and Ogunbolude, Y and Duarte, AE and Rocha, JBT and de Menezes, IRA and Barros, LM and Tsopmo, A and Lukong, KE and Kamdem, JP | Excluded | The article was excluded based on title and abstract screening as it did not meet the inclusion and exclusion criteria. |
| Combined treatment with GSNO and CAPE accelerates functional recovery via additive antioxidant activities in a mouse model of TBI | 2018 | Khan, M and Shunmugavel, A and Dhammu, TS and Khan, H and Singh, I and Singh, AK | Excluded | The article was excluded based on title and abstract screening as it did not meet the inclusion and exclusion criteria. |
| Dietary amelioration of locomotor, neurotransmitter and mitochondrial aging | 2010 | Aksenov, V and Long, JG and Lokuge, S and Foster, JA and Liu, JK and Rollo, CD | Excluded | The article was excluded based on title and abstract screening as it did not meet the inclusion and exclusion criteria. |
| Salivary glands as the source of plasma brain-derived neurotrophic factor in stressed rats engaged in biting behavior | 2010 | Saruta, J and Kondo, Y and Sato, C and Shiiki, N and Tsukinoki, K and Sato, S | Excluded | The article was excluded based on title and abstract screening as it did not meet the inclusion and exclusion criteria. |
| Altered Oxidative Stress Profile in the Cortex of Mice Fed an Enriched Branched-Chain Amino Acids Diet: Possible Link With Amyotrophic Lateral Sclerosis? | 2011 | Piscopo, P and Crestini, A and Adduci, A and Ferrante, A and Massari, M and Popoli, P and Vanacore, N and Confaloni, A | Excluded | The article was excluded based on title and abstract screening as it did not meet the inclusion and exclusion criteria. |
| Biological basis of neuroprotection and neurotherapeutic effects of Whole Body Periodic Acceleration (pGz) | 2014 | Adams, JA and Uryash, A and Bassuk, J and Sackner, MA and Kurlansky, P | Excluded | The article was excluded based on title and abstract screening as it did not meet the inclusion and exclusion criteria. |
| Effects of chronic restraint stress on the global DNA methylation profile of rat lung cells: Modulation by physical exercise | 2017 | Toffoli, LV and Volpini, VL and Nascimento, LM and Silva, WR and Verissimo, LF and Estrada, VB and Pelosi, GG and Gomes, MV | Excluded | The article was excluded based on title and abstract screening as it did not meet the inclusion and exclusion criteria. |
| The Link between Oxidative Stress, Redox Status, Bioenergetics and Mitochondria in the Pathophysiology of ALS | 2021 | Obrador, E and Salvador-Palmer, R and López-Blanch, R and Jihad-Jebbar, A and Vallés, SL and Estrela, JM | Excluded | The article was excluded based on title and abstract screening as it did not meet the inclusion and exclusion criteria. |
| Intranasal insulin intake and exercise improve memory function in amyloid-β induced Alzheimer's-like disease in rats: Involvement of hippocampal BDNF-TrkB receptor | 2024 | Larijani, SF and Hassanzadeh, G and Zahmatkesh, M and Radfar, F and Farahmandfar, M | Excluded | The article was excluded based on title and abstract screening as it did not meet the inclusion and exclusion criteria. |
| Interactive Effects of Endurance Training and Royal Jelly Consumption on Motor Balance and Pain Threshold in Animal Model of the Alzheimer Disease | 2020 | Hosseini, SA and Salehi, OR and Farzanegi, P and Farkhaie, F and Darvishpour, AR and Roozegar, S | Excluded | The article was excluded based on title and abstract screening as it did not meet the inclusion and exclusion criteria. |
| EXERCISE TRAINING ALTERS CARDIOVASCULAR AND HORMONAL RESPONSES TO INTRACRANIAL SELF-STIMULATION | 1993 | BURGESS, ML and DAVIS, JM and BORG, TK and WILSON, SP and BURGESS, WA and BUGGY, J | Excluded | The article was excluded based on title and abstract screening as it did not meet the inclusion and exclusion criteria. |
| Mild stress facilitates learning and exercise improves retention in aged mice | 2011 | Adlard, PA and Engesser-Cesar, C and Cotman, CW | Excluded | The article was excluded based on title and abstract screening as it did not meet the inclusion and exclusion criteria. |
| Anticonvulsant effects of<i> Cymbopogon</i><i> giganteus</i> extracts with possible effects on fully kindled seizures and anxiety in experimental rodent model of mesio-temporal epilepsy induced by pilocarpine | 2022 | Pale, S and Neteydji, S and Taiwe, GS and Emegam, NK and Bum, EN | Excluded | The article was excluded based on title and abstract screening as it did not meet the inclusion and exclusion criteria. |
| Nature of stress: Differential effects on brain acetylcholinesterase activity and memory in rats | 2005 | Das, A and Rai, D and Dikshit, M and Palit, G and Nath, C | Excluded | The article was excluded based on title and abstract screening as it did not meet the inclusion and exclusion criteria. |
| Distinct, Time-Dependent Effects of Voluntary Exercise on Circadian and Ultradian Rhythms and Stress Responses of Free Corticosterone in the Rat Hippocampus | 2009 | Droste, SK and Collins, A and Lightman, SL and Linthorst, ACE and Reul, JMHM | Excluded | The article was excluded based on title and abstract screening as it did not meet the inclusion and exclusion criteria. |
| Central autonomic integration of psychological stressors: Focus on cardiovascular modulation | 2005 | McDougall, SJ and Widdop, RE and Lawrence, AJ | Excluded | The article was excluded based on title and abstract screening as it did not meet the inclusion and exclusion criteria. |
| Organization of brain somatomotor-sympathetic circuits | 2008 | Kerman, IA | Excluded | The article was excluded based on title and abstract screening as it did not meet the inclusion and exclusion criteria. |
| Involuntary swimming exercise in pregnant rats disturbs ERK1/2 signaling in embryonic neurons through increased cortisol in the amniotic fluid | 2018 | Jang, Y and Lee, B and Kim, EK and Shim, WS and Yang, YD and Kim, SM | Excluded | The article was excluded based on title and abstract screening as it did not meet the inclusion and exclusion criteria. |
| EFFECT OF THE DIFFERENT DOSES OF ACRYLAMIDE ON ACETYLOCHOLINOESTERASE ACTIVITY, THIOL GROUPS, MALONDIALDEHYDE CONCENTRATIONS IN HYPOTHALAMUS AND SELECTED MUSCLES OF MICE | 2017 | Kopanska, M and Czech, J and Zagata, P and Dobrek, L and Thor, P and Formicki, G | Excluded | The article was excluded based on title and abstract screening as it did not meet the inclusion and exclusion criteria. |
| Dietary factors, hormesis and health | 2008 | Mattson, MP | Excluded | The article was excluded based on title and abstract screening as it did not meet the inclusion and exclusion criteria. |
| Affective dysfunction in a mouse model of Rett syndrome: Therapeutic effects of environmental stimulation and physical activity | 2016 | Kondo, MA and Gray, LJ and Pelka, GJ and Leang, SK and Christodoulou, J and Tam, PPL and Hannan, AJ | Excluded | The article was excluded based on title and abstract screening as it did not meet the inclusion and exclusion criteria. |
| Stress and Alterations in Bones: An interdisciplinary Perspective | 2017 | Wippert, PM and Rector, M and Kuhn, G and Wuertz-Kozak, K | Excluded | The article was excluded based on title and abstract screening as it did not meet the inclusion and exclusion criteria. |
| Activity-based anorexia has differential effects on apical dendritic branching in dorsal and ventral hippocampal CA1 | 2014 | Chowdhury, TG and Barbarich-Marsteller, NC and Chan, TE and Aoki, C | Excluded | The article was excluded based on title and abstract screening as it did not meet the inclusion and exclusion criteria. |
| Effects of spontaneous and forced running on activation of hypothalamic corticotropin-releasing hormone neurons in rats | 2007 | Yanagita, S and Amemiya, S and Suzuki, S and Kita, I | Excluded | The article was excluded based on title and abstract screening as it did not meet the inclusion and exclusion criteria. |
| Neuroprotective effect of solanesol against 3-nitropropionic acid-induced Huntington's disease-like behavioral, biochemical, and cellular alterations: Restoration of coenzyme-Q10-mediated mitochondrial dysfunction | 2018 | Mehan, S and Monga, V and Rani, M and Dudi, R and Ghimire, K | Excluded | The article was excluded based on title and abstract screening as it did not meet the inclusion and exclusion criteria. |
| From the Gut to the Brain: The Role of Enteric Glial Cells and Their Involvement in the Pathogenesis of Parkinson's Disease | 2024 | Montalbán-Rodríguez, A and Abalo, R and López-Gómez, L | Excluded | The article was excluded based on title and abstract screening as it did not meet the inclusion and exclusion criteria. |
| Effect of fermented porcine placenta on physical fatigue in mice | 2016 | Kim, HY and Han, NR and Kim, NR and Lee, M and Kim, J and Kim, CJ and Jeong, HJ and Kim, HM | Excluded | The article was excluded based on title and abstract screening as it did not meet the inclusion and exclusion criteria. |
| Recovery of Stress Response Coincides with Responsiveness to Voluntary Exercise after Traumatic Brain Injury | 2014 | Griesbach, GS and Tio, DL and Nair, S and Hovda, DA | Excluded | The article was excluded based on title and abstract screening as it did not meet the inclusion and exclusion criteria. |
| Depression, anxiety-like behavior and memory impairment are associated with increased oxidative stress and inflammation in a rat model of social stress | 2013 | Patki, G and Solanki, N and Atrooz, F and Allam, F and Salim, S | Excluded | The article was excluded based on title and abstract screening as it did not meet the inclusion and exclusion criteria. |
| Environmental enrichment-behavior-oxidative stress interactions in the aged rat -: Issues for a therapeutic approach in human aging | 2004 | Fernández, CI and Collazo, J and Bauza, Y and Castellanos, MR and López, O | Excluded | The article was excluded based on title and abstract screening as it did not meet the inclusion and exclusion criteria. |
| EFFECTS OF INTRACRANIAL SELF-STIMULATION ON SELECTED PHYSIOLOGICAL VARIABLES IN RATS | 1993 | BURGESS, ML and DAVIS, JM and WILSON, SP and BORG, TK and BURGESS, WA and BUGGY, J | Excluded | The article was excluded based on title and abstract screening as it did not meet the inclusion and exclusion criteria. |
| Protective neuroendocrine effects of environmental enrichment and voluntary exercise against social isolation: evidence for mediation by limbic structures | 2019 | Watanasriyakul, WT and Normann, MC and Akinbo, OI and Colburn, W and Dagner, A and Grippo, AJ | Excluded | The article was excluded based on title and abstract screening as it did not meet the inclusion and exclusion criteria. |
| Differential protein expression in the nucleus accumbens of high and low active mice | 2015 | Ferguson, DP and Dangott, LJ and Vellers, HL and Schmitt, EE and Lightfoot, JT | Excluded | The article was excluded based on title and abstract screening as it did not meet the inclusion and exclusion criteria. |
| Increased Nerve Growth Factor Serum Levels in Top Athletes | 2013 | Bonini, M and Fioretti, D and Sargentini, V and Del Giacco, S and Rinaldi, M and Tranquilli, C and Bonini, S | Excluded | The article was excluded based on title and abstract screening as it did not meet the inclusion and exclusion criteria. |
| Benefit effect of REM-sleep deprivation on memory impairment induced by intensive exercise in male wistar rats: with respect to hippocampal BDNF and TrkB | 2019 | Mahboubi, S and Nasehi, M and Imani, A and Sadat-Shirazi, MS and Zarrindast, MR and Vousooghi, N and Noroozian, M | Excluded | The article was excluded based on title and abstract screening as it did not meet the inclusion and exclusion criteria. |
| NADPH oxidase and redox status in amygdala, hippocampus and cortex of male Wistar rats in an animal model of post-traumatic stress disorder | 2018 | Petrovic, R and Puskas, L and Jevtic Dozudic, G and Stojkovic, T and Velimirovic, M and Nikolic, T and Zivkovic, M and Djorovic, DJ and Nenadovic, M and Petronijevic, N | Excluded | The article was excluded based on title and abstract screening as it did not meet the inclusion and exclusion criteria. |
| The Calm Mouse: An Animal Model of Stress Reduction | 2012 | Gurfein, BT and Stamm, AW and Bacchetti, P and Dallman, MF and Nadkarni, NA and Milush, JM and Touma, C and Palme, R and Di Borgo, CP and Fromentin, G and Lown-Hecht, R and Konsman, JP and Acree, M and Premenko-Lanier, M and Darcel, N and Hecht, FM and Nixon, DF | Excluded | The article was excluded based on title and abstract screening as it did not meet the inclusion and exclusion criteria. |
| A Novel Animal Model Simulating the Beginning of Combat Exposure | 2017 | Kim, D and Kim, CY and Koo, H and Heo, Y and Cheon, K | Excluded | The article was excluded based on title and abstract screening as it did not meet the inclusion and exclusion criteria. |
| Environmental enrichment, exercise and corticosterone affect endothelial cell proliferation in adult rat hippocampus and prefrontal cortex | 2008 | Ekstrand, J and Hellsten, J and Tingström, A | Excluded | The article was excluded based on title and abstract screening as it did not meet the inclusion and exclusion criteria. |
| A model of negative emotional contagion between male-female rat dyads: Effects of voluntary exercise on stress-induced behavior and BDNF-TrkB signaling | 2021 | Meade, GM and Charron, LS and Kilburn, LW and Pei, Z and Wang, HY and Robinson, S | Excluded | The article was excluded based on title and abstract screening as it did not meet the inclusion and exclusion criteria. |
| Pre-pubertal, low-intensity exercise does not require concomitant venlafaxine to induce robust, late-life antidepressant effects in Flinders sensitive line rats | 2020 | Steyn, SF and Harvey, BH and Brink, CB | Excluded | The article was excluded based on title and abstract screening as it did not meet the inclusion and exclusion criteria. |
| INFLUENCE OF PERINATAL <i>TRANS</i> FAT ON BEHAVIORAL RESPONSES AND BRAIN OXIDATIVE STATUS OF ADOLESCENT RATS ACUTELY EXPOSED TO STRESS | 2013 | Pase, CS and Roversi, K and Trevizol, F and Roversi, K and Kuhn, FT and Schuster, AJ and Vey, LT and Dias, VT and Barcelos, RCS and Piccolo, J and Emanuelli, T and Bürger, ME | Excluded | The article was excluded based on title and abstract screening as it did not meet the inclusion and exclusion criteria. |
| A new SOD mimic, Mn(III) <i>ortho N</i>-butoxyethylpyridylporphyrin, combines superb potency and lipophilicity with low toxicity | 2012 | Rajic, Z and Tovmasyan, A and Spasojevic, I and Sheng, HX and Lu, MM and Li, AM and Gralla, EB and Warner, DS and Benov, L and Batinic-Haberle, I | Excluded | The article was excluded based on title and abstract screening as it did not meet the inclusion and exclusion criteria. |
| Physical exercise promotes memory capability by enhancing hippocampal mitochondrial functions and inhibiting apoptosis in obesity-induced insulin resistance by high fat diet | 2018 | Park, HS and Cho, HS and Kim, TW | Excluded | The article was excluded based on title and abstract screening as it did not meet the inclusion and exclusion criteria. |
| LONG-TERM EXPOSURE TO HIGH FAT DIET IS BAD FOR YOUR BRAIN: EXACERBATION OF FOCAL ISCHEMIC BRAIN INJURY | 2011 | Langdon, KD and Clarke, J and Corbett, D | Excluded | The article was excluded based on title and abstract screening as it did not meet the inclusion and exclusion criteria. |
| Sodium valproate enhances doxorubicin-induced cognitive dysfunction in Wistar rats | 2017 | Verma, T and Mallik, SB and Ramalingayya, GV and Nayak, PG and Kishore, A and Pai, KSR and Nandakumar, K | Excluded | The article was excluded based on title and abstract screening as it did not meet the inclusion and exclusion criteria. |
| Antioxidant treatment reduces blast-induced cochlear damage and hearing loss | 2012 | Ewert, DL and Lu, JZ and Li, W and Du, XP and Floyd, R and Kopke, R | Excluded | The article was excluded based on title and abstract screening as it did not meet the inclusion and exclusion criteria. |
| Changed gene expression in brains of mice exposed to traffic in a highway tunnel | 2012 | Bos, I and De Boever, P and Emmerechts, J and Buekers, J and Vanoirbeek, J and Meeusen, R and Van Poppel, M and Nemery, B and Nawrot, T and Panis, LI | Excluded | The article was excluded based on title and abstract screening as it did not meet the inclusion and exclusion criteria. |
| Exercise effects stress-induced analgesia and spatial learning in rats | 2006 | Blustein, JE and McLaughlin, M and Hoffman, JR | Excluded | The article was excluded based on title and abstract screening as it did not meet the inclusion and exclusion criteria. |
| BEHAVIORAL-CORRELATES OF SYMPATHOADRENAL REACTIVITY - THE TOUGHNESS MODEL | 1991 | DIENSTBIER, RA | Excluded | The article was excluded based on title and abstract screening as it did not meet the inclusion and exclusion criteria. |
| Western diet is associated with a smaller hippocampus: a longitudinal investigation | 2015 | Jacka, FN and Cherbuin, N and Anstey, KJ and Sachdev, P and Butterworth, P | Excluded | The article was excluded based on title and abstract screening as it did not meet the inclusion and exclusion criteria. |
| Discovery of small-molecule compounds and natural products against Parkinson's disease: Pathological mechanism and structural modification | 2022 | Zhong, ZC and He, XR and Ge, JM and Zhu, JL and Yao, CS and Cai, H and Ye, XY and Xie, T and Bai, RR | Excluded | The article was excluded based on title and abstract screening as it did not meet the inclusion and exclusion criteria. |
| Cycling and Spatial Navigation in an Enriched, Immersive 3D Virtual Park Environment: A Feasibility Study in Younger and Older Adults | 2019 | Sakhare, AR and Yang, V and Stradford, J and Tsang, I and Ravichandran, R and Pa, J | Excluded | The article was excluded based on title and abstract screening as it did not meet the inclusion and exclusion criteria. |
| Involvement in Sports, Hippocampal Volume, and Depressive Symptoms in Children | 2019 | Gorham, LS and Jernigan, T and Hudziak, J and Barch, DM | Excluded | The article was excluded based on title and abstract screening as it did not meet the inclusion and exclusion criteria. |
| Construction of a Brain-specific SLC23A2 Gene Knockout Mice Model | 2023 | Cao, B and Xia, Y and Cai, ZX and Wang, ZY and Tang, C and Song, YH | Excluded | The article was excluded based on title and abstract screening as it did not meet the inclusion and exclusion criteria. |
| Sex-specific issues of central and peripheral arginine-vasopressin concentrations in neurocritical care patients | 2022 | Podtschaske, AH and Martin, J and Ulm, B and Jungwirth, B and Kagerbauer, SM | Excluded | The article was excluded based on title and abstract screening as it did not meet the inclusion and exclusion criteria. |
| The Neuroscience of Resilience | 2018 | Hunter, RG and Gray, JD and McEwen, BS | Excluded | The article was excluded based on title and abstract screening as it did not meet the inclusion and exclusion criteria. |
| Enriched Environment Prevents Hypobaric Hypoxia Induced Memory Impairment and Neurodegeneration: Role of BDNF/PI3K/GSK3β Pathway Coupled with CREB Activation | 2013 | Jain, V and Baitharu, I and Prasad, D and Ilavazhagan, G | Excluded | The article was excluded based on title and abstract screening as it did not meet the inclusion and exclusion criteria. |
| Effect of treadmill exercise on catalepsy and the expression of the BDNF gene in 1-methyl-4-phenyl-1,2,3,6-tetrahydropyridine-induced Parkinson in male <i>NMRI</i> mice | 2020 | Nazif, NN and Khosravi, M and Ahmadi, R and Bananej, M and Majd, A | Excluded | The article was excluded based on title and abstract screening as it did not meet the inclusion and exclusion criteria. |
| Exercise-induced re-programming of age-related metabolic changes in microglia is accompanied by a reduction in senescent cells | 2020 | Mela, V and Mota, BC and Milner, M and McGinley, A and Mills, KHG and Kelly, AM and Lynch, MA | Excluded | The article was excluded based on title and abstract screening as it did not meet the inclusion and exclusion criteria. |
| Voluntary wheel running modulates glutamate receptor subunit gene expression and stress hormone release in Lewis rats | 2003 | Makatsori, A and Duncko, R and Schwendt, M and Moncek, F and Johansson, BB and Jezova, D | Excluded | The article was excluded based on title and abstract screening as it did not meet the inclusion and exclusion criteria. |
| Running during adolescence rescues a maternal separation-induced memory impairment in female mice: Potential role of differential exon-specific BDNF expression | 2017 | Wearick-Silva, LE and Marshall, P and Viola, TW and Centeno-Silva, A and de Azeredo, LA and Orso, R and Li, X and Donadio, MV and Bredy, TW and Grassi-Oliveira, R | Excluded | The article was excluded based on title and abstract screening as it did not meet the inclusion and exclusion criteria. |
| Cellular responses to moderate and heavy exercise | 1998 | Nehlsen-Cannarella, SL | Excluded | The article was excluded based on title and abstract screening as it did not meet the inclusion and exclusion criteria. |
| INCREASED GENE EXPRESSION OF SELECTED VESICULAR AND GLIAL GLUTAMATE TRANSPORTERS IN THE FRONTAL CORTEX IN RATS EXPOSED TO VOLUNTARY WHEEL RUNNING | 2017 | Graban, J and Hlavacova, N and Jezova, D | Excluded | The article was excluded based on title and abstract screening as it did not meet the inclusion and exclusion criteria. |
| Physical activity alters the brain Hsp72 and IL-1β responses to peripheral <i>E-coli</i> challenge | 2005 | Nickerson, M and Elphick, GF and Campisi, J and Greenwood, BN and Fleshner, M | Excluded | The article was excluded based on title and abstract screening as it did not meet the inclusion and exclusion criteria. |
| The Effect of REM Sleep Deprivation on mTOR Signaling-Induced by Severe Physical Exercise | 2019 | Mahboubi, S and Nasehi, M and Imani, A and Sadat-Shirazi, MS and Zarrindast, MR and Vousooghi, N and Noroozian, M | Excluded | The article was excluded based on title and abstract screening as it did not meet the inclusion and exclusion criteria. |
| Constitutive PGC-1α Overexpression in Skeletal Muscle Does Not Improve Morphological Outcome in Mouse Models of Brain Irradiation or Cortical Stroke | 2018 | Karlsson, L and González-Alvarado, MN and Larrosa-Flor, M and Osman, A and Börjesson, M and Blomgren, K and Kuhn, HG | Excluded | The article was excluded based on title and abstract screening as it did not meet the inclusion and exclusion criteria. |
| The defects of the hippocampal ripples and theta rhythm in depression, and the effects of physical exercise on their amelioration | 2024 | Koketsu, S and Matsubara, K and Ueki, Y and Shinohara, Y and Inoue, K and Murakami, S and Ueki, T | Excluded | The article was excluded based on title and abstract screening as it did not meet the inclusion and exclusion criteria. |
| Epigallocatechin gallate ameliorates behavioral and biochemical deficits in rat model of load-induced chronic fatigue syndrome | 2011 | Sachdeva, AK and Kuhad, A and Chopra, K | Excluded | The article was excluded based on title and abstract screening as it did not meet the inclusion and exclusion criteria. |
| Beneficial effects of <i>Spirulina platensis</i>, voluntary exercise and environmental enrichment against adolescent stress induced deficits in cognitive functions, hippocampal BDNF and morphological remolding in adult female rats | 2019 | Moradi-Kor, N and Ghanbari, A and Rashidipour, H and Yousefi, B and Bandegi, AR and Rashidy-Pour, A | Excluded | The article was excluded based on title and abstract screening as it did not meet the inclusion and exclusion criteria. |
| EFFECTS OF EXERCISE ON IMMUNE FUNCTIONS OF UNDERNOURISHED MICE | 1992 | FILTEAU, SM and MENZIES, RA and KAIDO, TJ and OGRADY, MP and GELDERD, JB and HALL, NRS | Excluded | The article was excluded based on title and abstract screening as it did not meet the inclusion and exclusion criteria. |
| Effect of exercise on microglial activation and transcriptome of hippocampus in fluorosis mice | 2021 | Wang, JX and Yue, BJ and Zhang, XH and Guo, X and Sun, ZL and Niu, RY | Excluded | The article was excluded based on title and abstract screening as it did not meet the inclusion and exclusion criteria. |
| Effect of stress on prepulse inhibition: a systematic review | 2021 | Pujante-Gil, S and Manzanedo, C and Arenas, MC | Excluded | The article was excluded based on title and abstract screening as it did not meet the inclusion and exclusion criteria. |
| Environmental enrichment, new neurons and the neurobiology of individuality | 2019 | Kempermann, G | Excluded | The article was excluded based on title and abstract screening as it did not meet the inclusion and exclusion criteria. |
| Long term treadmill exercise performed to chronic social isolated rats regulate anxiety behavior without improving learning | 2018 | Cevik, OS and Sahin, L and Tamer, L | Excluded | The article was excluded based on title and abstract screening as it did not meet the inclusion and exclusion criteria. |
| High-Intense Interval Training Prevents Cognitive Impairment and Increases the Expression of Muscle Genes FNDC5 and PPARGC1A in a Rat Model of Alzheimer's Disease | 2022 | Godinho, WDN and Vasconcelos, FSL and Pinto, DV and Alves, JO and Nascimento, TD and de Aguiar, ID and Almeida, GNS and Ceccatto, VM and Soares, PM | Excluded | The article was excluded based on title and abstract screening as it did not meet the inclusion and exclusion criteria. |
| Artificial light at night suppresses the day-night cardiovascular variability: evidence from humans and rats | 2024 | Molcan, L and Babarikova, K and Cvikova, D and Kincelova, N and Kubincova, L and Sutovska, HM | Excluded | The article was excluded based on title and abstract screening as it did not meet the inclusion and exclusion criteria. |
| Neuroplasticity-related correlates of environmental enrichment combined with physical activity differ between the sexes | 2019 | Kokras, N and Sotiropoulos, I and Besinis, D and Tzouveka, EL and Almeida, OFX and Sousa, N and Dalla, C | Excluded | The article was excluded based on title and abstract screening as it did not meet the inclusion and exclusion criteria. |
| Repeated novel cage exposure-induced improvement of early Alzheimer's-like cognitive and amyloid changes in TASTPM mice is unrelated to changes in brain endocannabinoids levels | 2009 | Pardon, MC and Sarmad, S and Rattray, I and Bates, TE and Scullion, GA and Marsden, CA and Barrett, DA and Lowe, J and Kendall, DA | Excluded | The article was excluded based on title and abstract screening as it did not meet the inclusion and exclusion criteria. |
| Venlafaxine inhibits naloxone-precipitated morphine withdrawal symptoms: Role of inflammatory cytokines and nitric oxide | 2020 | Mansouri, MT and Naghizadeh, B and Ghorbanzadeh, B and Amirgholami, N and Houshmand, G and Alboghobeish, S | Excluded | The article was excluded based on title and abstract screening as it did not meet the inclusion and exclusion criteria. |
| Disrupted energy metabolism and neuronal circuit dysfunction in cognitive impairment and Alzheimer's disease | 2011 | Kapogiannis, D and Mattson, MP | Excluded | The article was excluded based on title and abstract screening as it did not meet the inclusion and exclusion criteria. |
| Identification and management of cognitive decline in companion animals and the comparisons with Alzheimer disease: A review | 2013 | Cory, J | Excluded | The article was excluded based on title and abstract screening as it did not meet the inclusion and exclusion criteria. |
| The environmental enrichment model revisited: A translatable paradigm to study the stress of our modern lifestyle | 2022 | Rojas-Carvajal, M and Sequeira-Cordero, A and Brenes, JC | Excluded | The article was excluded based on title and abstract screening as it did not meet the inclusion and exclusion criteria. |
| Melatonin as a Potential Approach to Anxiety Treatment | 2022 | Repova, K and Baka, T and Krajcirovicova, K and Stanko, P and Aziriova, S and Reiter, RJ and Simko, F | Excluded | The article was excluded based on title and abstract screening as it did not meet the inclusion and exclusion criteria. |
| Novel approaches to alcohol rehabilitation: Modification of stress-responsive brain regions through environmental enrichment | 2019 | Pang, TY and Hannan, AJ and Lawrence, AJ | Excluded | The article was excluded based on title and abstract screening as it did not meet the inclusion and exclusion criteria. |
| Physical exercise counteracts the increase in velocity of propagation of cortical spreading depression imposed by early over-nutrition in rats | 2020 | Monteiro, HMC and de Mendonça, DC and Sousa, MSB and Amancio-dos-Santos, A | Excluded | The article was excluded based on title and abstract screening as it did not meet the inclusion and exclusion criteria. |
| Treadmill exercise sex-dependently alters susceptibility to depression-like behaviour, cytokines and BDNF in the hippocampus and prefrontal cortex of rats with sporadic Alzheimer-like disease | 2021 | Naghibi, S and Joneydi, MS and Barzegari, A and Davoodabadi, A and Ebrahimi, A and Eghdami, E and Fahimpour, N and Ghorbani, M and Mohammadikia, E and Rostami, M and Salari, AA | Excluded | The article was excluded based on title and abstract screening as it did not meet the inclusion and exclusion criteria. |
| Environmental and Genetic Activation of a Brain-Adipocyte BDNF/Leptin Axis Causes Cancer Remission and Inhibition | 2010 | Cao, L and Liu, XL and Lin, EJD and Wang, CS and Choi, EY and Riban, V and Lin, B and During, MJ | Excluded | The article was excluded based on title and abstract screening as it did not meet the inclusion and exclusion criteria. |
| Mastication as a Stress-Coping Behavior | 2015 | Kubo, KY and Iinuma, M and Chen, HY | Excluded | The article was excluded based on title and abstract screening as it did not meet the inclusion and exclusion criteria. |
| Aging and Stress: Past Hypotheses, Present Approaches and Perspectives | 2011 | Garrido, P | Excluded | The article was excluded based on title and abstract screening as it did not meet the inclusion and exclusion criteria. |
| Resveratrol prevents akinesia and restores neuronal tyrosine hydroxylase immunoreactivity in the substantia <i>nigra pars</i> compacta of diabetic rats | 2014 | Bagatini, PB and Xavier, LL and Neues, LT and Saur, L and Barbosa, S and Baptista, PPA and Augustin, OA and de Senna, PN and Mestriner, RG and Souto, AA and Achaval, M | Excluded | The article was excluded based on title and abstract screening as it did not meet the inclusion and exclusion criteria. |
| Brain Temperature in Spontaneously Hypertensive Rats during Physical Exercise in Temperate and Warm Environments | 2016 | Drummond, LR and Kunstetter, AC and Vaz, FF and Campos, HO and de Andrade, AGP and Coimbra, CC and Natali, AJ and Wanner, SP and Prímola-Gomes, TN | Excluded | The article was excluded based on title and abstract screening as it did not meet the inclusion and exclusion criteria. |
| Evaluation of betulinic acid effects on pain, memory, anxiety, catalepsy, and oxidative stress in animal model of Parkinson's disease | 2023 | Abrishamdar, M and Farbood, Y and Sarkaki, A and Rashno, M and Badavi, M | Excluded | The article was excluded based on title and abstract screening as it did not meet the inclusion and exclusion criteria. |
| Anorexia nervosa as a motivated behavior: Relevance of anxiety, stress, fear and learning | 2015 | Guarda, AS and Schreyer, CC and Boersma, GJ and Tamashiro, KL and Moran, TH | Excluded | The article was excluded based on title and abstract screening as it did not meet the inclusion and exclusion criteria. |
| Sex-dependent and independent effects of long-term voluntary wheel running on Bdnf mRNA and protein expression | 2016 | Venezia, AC and Guth, LM and Sapp, RM and Spangenburg, EE and Roth, SM | Excluded | The article was excluded based on title and abstract screening as it did not meet the inclusion and exclusion criteria. |
| Omega-3 polyunsaturated fatty acid encapsulation system: Physical and oxidative stability, and medical applications | 2022 | Du, QW and Zhou, LH and Li, MH and Lyu, F and Liu, JH and Ding, YT | Excluded | The article was excluded based on title and abstract screening as it did not meet the inclusion and exclusion criteria. |
| Hormonal and psychological influences on performance anxiety in adolescent female volleyball players: a multi-approach study | 2024 | Rossi, C and Amato, A and Alesi, M and Alioto, A and Schiera, G and Drid, P and Messina, G and Pagliaro, A and Di Liegro, I and Proia, P | Excluded | The article was excluded based on title and abstract screening as it did not meet the inclusion and exclusion criteria. |
| Garlic (<i>Allium sativum</i>) improves anxiety- and depressive-related behaviors and brain oxidative stress in diabetic rats | 2020 | Rahmani, G and Farajdokht, F and Mohaddes, G and Babri, S and Ebrahimi, V and Ebrahimi, H | Excluded | The article was excluded based on title and abstract screening as it did not meet the inclusion and exclusion criteria. |
| Exercise protects myelinated fibers of white matter in a rat model of depression | 2018 | Xiao, Q and Wang, FF and Luo, YM and Chen, LM and Chao, FL and Tan, CX and Gao, Y and Huang, CX and Zhang, L and Liang, X and Tang, J and Qi, YQ and Jiang, L and Zhang, Y and Zhou, CN and Tang, Y | Excluded | The article was excluded based on title and abstract screening as it did not meet the inclusion and exclusion criteria. |
| The effect of endurance training and testosterone supplementation on the expression of blood spinal cord barrier proteins in rats | 2019 | Nierwinska, K and Nowacka-Chmielewska, M and Bernacki, J and Jagsz, S and Chalimoniuk, M and Langfort, J and Malecki, A | Excluded | The article was excluded based on title and abstract screening as it did not meet the inclusion and exclusion criteria. |
| The comparative effects of environmental enrichment with exercise and serotonin transporter blockade on serotonergic neurons in the dorsal raphe nucleus | 2012 | Macgillivray, L and Reynolds, KB and Rosebush, PI and Mazurek, MF | Excluded | The article was excluded based on title and abstract screening as it did not meet the inclusion and exclusion criteria. |
| The links between membrane composition, metabolic rate and lifespan | 2008 | Hulbert, AJ | Excluded | The article was excluded based on title and abstract screening as it did not meet the inclusion and exclusion criteria. |
| Effect of a dietary combined vitamin C and E supplementation on the liver, kidneys and brain arginase activity in non-pregnant and pregnant rats with streptozotocin-induced diabetes | 2006 | Erisir, M and Naziroglu, M and Tasdemir, B and Simsek, M | Excluded | The article was excluded based on title and abstract screening as it did not meet the inclusion and exclusion criteria. |
| Peripheral insulin-like growth factor-I produces antidepressant-like behavior and contributes to the effect of exercise | 2009 | Duman, CH and Schlesinger, L and Terwilliger, R and Russell, DS and Newton, SS and Duman, RS | Excluded | The article was excluded based on title and abstract screening as it did not meet the inclusion and exclusion criteria. |
| Effects of moderate treadmill exercise and fluoxetine on behavioural and cognitive deficits, hypothalamic-pituitary-adrenal axis dysfunction and alternations in hippocampal BDNF and mRNA expression of apoptosis related proteins in a rat model of post-traumatic stress disorder | 2017 | Shafia, S and Vafaei, AA and Samaei, SA and Bandegi, AR and Rafiei, A and Valadan, R and Hosseini-Khah, Z and Mohammadkhani, R and Rashidy-Pour, A | Excluded | The article was excluded based on title and abstract screening as it did not meet the inclusion and exclusion criteria. |
| Physical exercise prevents behavioral alterations in a reserpine-treated zebrafish: A putative depression model | 2022 | Martins, GMD and Petersen, BD and Ruebensam, G and da Silva, JMK and Gaspary, KV and Wiprich, MT and Altenhofen, S and Bonan, CD | Excluded | The article was excluded based on title and abstract screening as it did not meet the inclusion and exclusion criteria. |
| Brain glycogen supercompensation following exhaustive exercise | 2012 | Matsui, T and Ishikawa, T and Ito, H and Okamoto, M and Inoue, K and Lee, M and Fujikawa, T and Ichitani, Y and Kawanaka, K and Soya, H | Excluded | The article was excluded based on title and abstract screening as it did not meet the inclusion and exclusion criteria. |
| Moderate exercise prevents neurodegeneration in D-galactose-induced aging mice | 2016 | Li, L and Xu, M and Shen, B and Li, M and Gao, Q and Wei, SG | Excluded | The article was excluded based on title and abstract screening as it did not meet the inclusion and exclusion criteria. |
| Adolescent voluntary exercise attenuated hippocampal innate immunity responses and depressive-like behaviors following maternal separation stress in male rats | 2016 | Sadeghi, M and Peeri, M and Hosseini, MJ | Excluded | The article was excluded based on title and abstract screening as it did not meet the inclusion and exclusion criteria. |
| Rehabilitative training tasks improve spatial learning impairment in the water maze following hypoxic-ischemic insult in neonatal rats | 2006 | Ikeda, T and Mishima, K and Aoo, N and Harada, K and Liu, AX and Egashira, N and Iwasaki, K and Fujiwara, M and Ikenoue, T | Excluded | The article was excluded based on title and abstract screening as it did not meet the inclusion and exclusion criteria. |
| Effects of β-alanine administration on selected parameters of oxidative stress and phosphoryltransfer network in cerebral cortex and cerebellum of rats | 2013 | Gemelli, T and de Andrade, RB and Rojas, DB and Bonorino, NF and Mazzola, PN and Tortorelli, LS and Funchal, C and Dutra, CS and Wannmacher, CMD | Excluded | The article was excluded based on title and abstract screening as it did not meet the inclusion and exclusion criteria. |
| Environmental Enrichment Protects the Retina from Early Diabetic Damage in Adult Rats | 2014 | Dorfman, D and Aranda, ML and Fleitas, MFG and Chianelli, MS and Fernandez, DC and Sande, PH and Rosenstein, RE | Excluded | The article was excluded based on title and abstract screening as it did not meet the inclusion and exclusion criteria. |
| The effects of physical exercise on nonmotor symptoms and on neuroimmune RAGE network in experimental parkinsonism | 2017 | Viana, SD and Pita, IR and Lemos, C and Rial, D and Couceiro, P and Rodrigues-Santos, P and Caramelo, F and Carvalho, F and Ali, SF and Prediger, RD and Ribeiro, CAF and Pereira, FC | Excluded | The article was excluded based on title and abstract screening as it did not meet the inclusion and exclusion criteria. |
| Metallothionein is a Potential Therapeutic Strategy for Amyotrophic Lateral Sclerosis | 2017 | Ono, S | Excluded | The article was excluded based on title and abstract screening as it did not meet the inclusion and exclusion criteria. |
| The investigation of levels of the peptide adrenomedullin (ADM) and the enzyme tyrosine hydroxylase (TH) in rat tissue after exposure to physiological stress | 2006 | Yurekli, M and Yarpuzlu, AA | Excluded | The article was excluded based on title and abstract screening as it did not meet the inclusion and exclusion criteria. |
| The effects of resistance training and spirulina on the performance of the antioxidant system with emphasis on <i>mir125b</i>, <i>mir146a </i>and cognitive function in stanazolol-induced neurotoxicity in rats | 2022 | Agahi, MRH and Mosallanejad, Z and Salehi, OR | Excluded | The article was excluded based on title and abstract screening as it did not meet the inclusion and exclusion criteria. |
| Brain microdialysis in exercise research | 2001 | Meeusen, R and Piacentini, MF and De Meirleir, K | Excluded | The article was excluded based on title and abstract screening as it did not meet the inclusion and exclusion criteria. |
| Effect of mitochondrial cofactors and antioxidants supplementation on cognition in the aged canine | 2016 | Snigdha, S and de Rivera, C and Milgram, NW and Cotman, CW | Excluded | The article was excluded based on title and abstract screening as it did not meet the inclusion and exclusion criteria. |
| The role of enriched environment in neural development and repair | 2022 | Han, Y and Yuan, M and Guo, YS and Shen, XY and Gao, ZK and Bi, X | Excluded | The article was excluded based on title and abstract screening as it did not meet the inclusion and exclusion criteria. |
| Moderate Treadmill Exercise Protects Synaptic Plasticity of the Dentate Gyrus and Related Signaling Cascade in a Rat Model of Alzheimer's Disease | 2015 | Dao, AT and Zagaar, MA and Alkadhi, KA | Excluded | The article was excluded based on title and abstract screening as it did not meet the inclusion and exclusion criteria. |
| What's wrong with my mouse cage? Methodological considerations for modeling lifestyle factors and gene-environment interactions in mice | 2016 | Mo, C and Renoir, T and Hannan, AJ | Excluded | The article was excluded based on title and abstract screening as it did not meet the inclusion and exclusion criteria. |
| Pharmacological activation of the amygdala, but not single prolonged footshock-induced acute stress, interferes with cue-induced motivation toward food rewards in rats | 2023 | Lai, CW and Chang, CH | Excluded | The article was excluded based on title and abstract screening as it did not meet the inclusion and exclusion criteria. |
| Radiation-induced neuropathological changes in the oligodendrocyte lineage with relevant clinical manifestations and therapeutic strategies | 2022 | Lee, RX and Tang, FR | Excluded | The article was excluded based on title and abstract screening as it did not meet the inclusion and exclusion criteria. |
| Sleep disturbances and severe stress as glial activators: key targets for treating central sensitization in chronic pain patients? | 2017 | Nijs, J and Loggia, ML and Polli, A and Moens, M and Huysmans, E and Goudman, L and Meeus, M and Vanderweeën, L and Ickmans, K and Clauw, D | Excluded | The article was excluded based on title and abstract screening as it did not meet the inclusion and exclusion criteria. |
| The role of nutrition in Alzheimer's disease: epidemiological evidence | 2009 | Morris, MC | Excluded | The article was excluded based on title and abstract screening as it did not meet the inclusion and exclusion criteria. |
| Effects of long-term voluntary exercise on the mouse hypothalamic-pituitary-adrenocortical axis | 2003 | Droste, SK and Gesing, A and Ulbricht, S and Müller, MB and Linthorst, ACE and Reul, JMHM | Excluded | The article was excluded based on title and abstract screening as it did not meet the inclusion and exclusion criteria. |
| The 1-Week and 8-Month Effects of a Ketogenic Diet or Ketone Salt Supplementation on Multi-Organ Markers of Oxidative Stress and Mitochondrial Function in Rats | 2017 | Kephart, WC and Mumford, PW and Mao, XS and Romero, MA and Hyatt, HW and Zhang, YF and Mobley, CB and Quindry, JC and Young, KC and Beck, DT and Martin, JS and McCullough, DJ and D'Agostino, DP and Lowery, RP and Wilson, JM and Kavazis, AN and Roberts, MD | Excluded | The article was excluded based on title and abstract screening as it did not meet the inclusion and exclusion criteria. |
| Social Isolation Stress Induces Anxious-Depressive-Like Behavior and Alterations of Neuroplasticity-Related Genes in Adult Male Mice | 2016 | Ieraci, A and Mallei, A and Popoli, M | Excluded | The article was excluded based on title and abstract screening as it did not meet the inclusion and exclusion criteria. |
| Antidepressant and Proneurogenic Influence of Environmental Enrichment in Mice: Protective Effects <i>vs</i> Recovery | 2011 | Llorens-Martín, M and Tejeda, GS and Trejo, JL | Excluded | The article was excluded based on title and abstract screening as it did not meet the inclusion and exclusion criteria. |
| Cellular and molecular mechanisms of immunomodulation in the brain through environmental enrichment | 2014 | Singhal, G and Jaehne, EJ and Corrigan, F and Baune, BT | Excluded | The article was excluded based on title and abstract screening as it did not meet the inclusion and exclusion criteria. |
| A review of post-concussion syndrome and psychological factors associated with concussion | 2015 | Broshek, DK and De Marco, AP and Freeman, JR | Excluded | The article was excluded based on title and abstract screening as it did not meet the inclusion and exclusion criteria. |
| Swimming exercise improves cognitive and behavioral disorders in male NMRI mice with sporadic Alzheimer-like disease | 2020 | Bashiri, H and Enayati, M and Bashiri, A and Salari, AA | Excluded | The article was excluded based on title and abstract screening as it did not meet the inclusion and exclusion criteria. |
| Venous gas embolism as a predictive tool for improving CNS decompression safety | 2012 | Mollerlokken, A and Gaustad, SE and Havnes, MB and Gutvik, CR and Hjelde, A and Wisloff, U and Brubakk, AO | Excluded | The article was excluded based on title and abstract screening as it did not meet the inclusion and exclusion criteria. |
| Maternal exposure to silicon dioxide nanoparticles reduces hippocampal neurogenesis and synaptogenesis and induces neurodegeneration in rat offspring hippocampus | 2022 | Mahdipour, R and Ebrahimi, V and Hosseini, M and Soukhtanloo, M and Rastegar-Moghaddam, SH and Malvandi, AM and Mohammadipour, A | Excluded | The article was excluded based on title and abstract screening as it did not meet the inclusion and exclusion criteria. |
| Methylene blue improves sensorimotor phenotype and decreases anxiety in parallel with activating brain mitochondria biogenesis in mid-age mice | 2016 | Gureev, AP and Syromyatnikov, MY and Gorbacheva, TM and Starkov, AA and Popov, VN | Excluded | The article was excluded based on title and abstract screening as it did not meet the inclusion and exclusion criteria. |
| A high-fat, refined-carbohydrate diet induces endothelial dysfunction and oxidant/antioxidant imbalance and depresses NOS protein expression | 2005 | Roberts, CK and Barnard, RJ and Sindhu, RK and Jurczak, M and Ehdaie, A and Vaziri, ND | Excluded | The article was excluded based on title and abstract screening as it did not meet the inclusion and exclusion criteria. |
| Rehabilitation Improves Behavioral Recovery and Lessens Cell Death Without Affecting Iron, Ferritin, Transferrin, or Inflammation After Intracerebral Hemorrhage in Rats | 2014 | Caliaperumal, J and Colbourne, F | Excluded | The article was excluded based on title and abstract screening as it did not meet the inclusion and exclusion criteria. |
| Early Life Sleep Deprivation: Role of Oxido-Inflammatory Processes | 2019 | Atrooz, F and Liu, HS and Kochi, C and Salim, S | Excluded | The article was excluded based on title and abstract screening as it did not meet the inclusion and exclusion criteria. |
| Resistance Exercise Training Attenuates the Loss of Endogenous GLP-1 Receptor in the Hypothalamus of Type 2 Diabetic Rats | 2019 | Park, SH and Yoon, JH and Seo, DY and Kim, TN and Ko, JR and Han, J | Excluded | The article was excluded based on title and abstract screening as it did not meet the inclusion and exclusion criteria. |
| Oxidative damage in the senescence-accelerated mouse | 1998 | Mori, A and Utsumi, K and Liu, JK and Hosokawa, M | Excluded | The article was excluded based on title and abstract screening as it did not meet the inclusion and exclusion criteria. |
| Individual variation in the (patho)physiology of energy balance | 2011 | Boersma, GJ and Benthem, L and van Dijk, G and Scheurink, AJW | Excluded | The article was excluded based on title and abstract screening as it did not meet the inclusion and exclusion criteria. |
| A single episode of stress during adolescence impairs short-term memory and increases risk behaviour in an animal model of attention-deficit/ hyperactivity disorder | 2021 | Raony, I and Geraldo, A and Pandolfo, P | Excluded | The article was excluded based on title and abstract screening as it did not meet the inclusion and exclusion criteria. |
| Achieving and maintaining cognitive vitality with aging | 2002 | Fillit, HM and Butler, RN and O'Connell, AW and Albert, MS and Birren, JE and Cotman, CW and Greenough, WT and Gold, PE and Kramer, AF and Kuller, LH and Perls, TT and Sahagan, BG and Tully, T | Excluded | The article was excluded based on title and abstract screening as it did not meet the inclusion and exclusion criteria. |
| Environmental Enrichment Stimulates Immune Cell Secretion of Exosomes that Promote CNS Myelination and May Regulate Inflammation | 2016 | Pusic, KM and Pusic, AD and Kraig, RP | Excluded | The article was excluded based on title and abstract screening as it did not meet the inclusion and exclusion criteria. |
| Behavioural changes after different stress paradigms: prepulse inhibition increased after physical, but not emotional stress | 2003 | Pijlman, FTA and Herremans, AHJ and van de Kieft, J and Kruse, CG and van Ree, JM | Excluded | The article was excluded based on title and abstract screening as it did not meet the inclusion and exclusion criteria. |
| Using the Activity-based Anorexia Rodent Model to Study the Neurobiological Basis of Anorexia Nervosa | 2015 | Chowdhury, TG and Chen, YW and Aoki, C | Excluded | The article was excluded based on title and abstract screening as it did not meet the inclusion and exclusion criteria. |
| Effect of exercise, exercise withdrawal, and continued regular exercise on excitability and long-term potentiation in the dentate gyrus of hippocampus | 2016 | Radahmadi, M and Hosseini, N and Alaei, H | Excluded | The article was excluded based on title and abstract screening as it did not meet the inclusion and exclusion criteria. |
| Adaptogenic and central nervous system effects of single doses of 3% rosavin and 1% salidroside <i>Rhodiola rosea</i> L. extract in mice | 2007 | Perfumi, M and Mattioli, L | Excluded | The article was excluded based on title and abstract screening as it did not meet the inclusion and exclusion criteria. |
| Life on Magnet: Long-Term Exposure of Moderate Static Magnetic Fields on the Lifespan and Healthspan of Mice | 2023 | Fan, YX and Yu, X and Yu, B and Ji, XM and Tian, XF and Song, C and Zhang, X | Excluded | The article was excluded based on title and abstract screening as it did not meet the inclusion and exclusion criteria. |
| Chemotherapy and Cognitive Impairment: An Animal Model Approach | 2017 | Winocur, G | Excluded | The article was excluded based on title and abstract screening as it did not meet the inclusion and exclusion criteria. |
| Effect of rilmenidine on the cardiovascular responses to stress in the conscious rabbit | 1998 | Burke, SL and Malpas, SC and Head, GA | Excluded | The article was excluded based on title and abstract screening as it did not meet the inclusion and exclusion criteria. |
| Exercise training reverses the age-related decline in tyrosine hydroxylase expression in rat hypothalamus | 1997 | Tumer, N and LaRochelle, JS and Yurekli, M | Excluded | The article was excluded based on title and abstract screening as it did not meet the inclusion and exclusion criteria. |
| Conditioned media from AICAR-treated skeletal muscle cells increases neuronal differentiation of adult neural progenitor cells | 2019 | Moon, HY and Javadi, S and Stremlau, M and Yoon, KJ and Becker, B and Kang, SU and Zhao, XY and van Praag, H | Excluded | The article was excluded based on title and abstract screening as it did not meet the inclusion and exclusion criteria. |
| Daily Intermittent Normobaric Hypoxia Over 2 Weeks Reduces BDNF Plasma Levels in Young Adults - A Randomized Controlled Feasibility Study | 2018 | Becke, A and Müller, P and Dordevic, M and Lessmann, V and Brigadski, T and Müller, NG | Excluded | The article was excluded based on title and abstract screening as it did not meet the inclusion and exclusion criteria. |
| Spinal cord stimulation and the induction of c-fos and heat shock protein 72 in the central nervous system of rats | 1998 | DeJongste, MJL and Hautvast, RWM and Ruiters, MHJ and Ter Horst, GJ | Excluded | The article was excluded based on title and abstract screening as it did not meet the inclusion and exclusion criteria. |
| Simultaneous Changes in Sleep, qEEG, Physiology, Behaviour and Neurochemistry in Rats Exposed to Repeated Social Defeat Stress | 2016 | Ahnaou, A and Drinkenburg, WHIM | Excluded | The article was excluded based on title and abstract screening as it did not meet the inclusion and exclusion criteria. |
| Cerium oxide nanozyme modulate the 'exercise' redox biology of skeletal muscle | 2017 | Arya, A and Sethy, NK and Gangwar, A and Bhargava, N and Dubey, A and Roy, M and Srivastava, G and Singh, SK and Das, M and Bhargava, K | Excluded | The article was excluded based on title and abstract screening as it did not meet the inclusion and exclusion criteria. |
| Neurodevelopmental Consequences of Gestational and Lactational Exposure to Pyrethroids in Rats | 2016 | Syed, F and John, PJ and Soni, I | Excluded | The article was excluded based on title and abstract screening as it did not meet the inclusion and exclusion criteria. |
| Selective brain cooling: a multiple regulatory mechanism | 2004 | Caputa, M | Excluded | The article was excluded based on title and abstract screening as it did not meet the inclusion and exclusion criteria. |
| Does Postmenopausal Estrogen Use Confer Neuroprotection? | 2009 | Loucks, TL and Berga, SL | Excluded | The article was excluded based on title and abstract screening as it did not meet the inclusion and exclusion criteria. |
| Aging increases basal but not stress-induced levels of corticosterone in the brain of the awake rat | 2012 | Garrido, P and de Blas, M and Del Arco, A and Segovia, G and Mora, F | Excluded | The article was excluded based on title and abstract screening as it did not meet the inclusion and exclusion criteria. |
| Dyskinesia in Parkinson's disease: mechanisms and current non-pharmacological interventions | 2014 | Heumann, R and Moratalla, R and Herrero, MT and Chakrabarty, K and Drucker-Colín, R and Garcia-Montes, JR and Simola, N and Morelli, M | Excluded | The article was excluded based on title and abstract screening as it did not meet the inclusion and exclusion criteria. |
| Evaluation of the ergogenic properties of ginseng - An update | 2000 | Bahrke, MS and Morgan, WP | Excluded | The article was excluded based on title and abstract screening as it did not meet the inclusion and exclusion criteria. |
| Concurrent Effects of Exercise and Curcumin on Spatial Learning and Memory in Sensitized Male Mice Following Morphine Administration | 2019 | Elhampour, L and Azarbayjani, MA and Nasehi, M and Peeri, M | Excluded | The article was excluded based on title and abstract screening as it did not meet the inclusion and exclusion criteria. |
| Regular exercise prevents the development of hyperglucocorticoidemia via adaptations in the brain and adrenal glands in male Zucker diabetic fatty rats | 2010 | Campbell, JE and Király, MA and Atkinson, DJ and D'souza, AM and Vranic, M and Riddell, MC | Excluded | The article was excluded based on title and abstract screening as it did not meet the inclusion and exclusion criteria. |
| "Brain training" improves cognitive performance and survival in a transgenic mouse model of Huntington's disease | 2011 | Wood, NI and Glynn, D and Morton, AJ | Excluded | The article was excluded based on title and abstract screening as it did not meet the inclusion and exclusion criteria. |
| Morphological and cellular changes within embryonic striatal grafts associated with enriched environment and involuntary exercise | 2006 | Döbrössy, MD and Dunnett, SB | Excluded | The article was excluded based on title and abstract screening as it did not meet the inclusion and exclusion criteria. |
| INTRACRANIAL SELF-STIMULATION MOTIVATES TREADMILL RUNNING IN RATS | 1991 | BURGESS, ML and DAVIS, JM and BORG, TK and BUGGY, J | Excluded | The article was excluded based on title and abstract screening as it did not meet the inclusion and exclusion criteria. |
| Is Adult Hippocampal Neurogenesis Really Relevant for the Treatment of Psychiatric Disorders? | 2021 | Carli, M and Aringhieri, S and Kolachalam, S and Longoni, B and Grenno, G and Rossi, M and Gemignani, A and Fornai, F and Maggio, R and Scarselli, M | Excluded | The article was excluded based on title and abstract screening as it did not meet the inclusion and exclusion criteria. |
| A review of the multi-level adaptations for maximizing aerobic dive duration in marine mammals: from biochemistry to behavior | 2014 | Davis, RW | Excluded | The article was excluded based on title and abstract screening as it did not meet the inclusion and exclusion criteria. |
| Effect of Continuous and Interval Exercise on The Expression of Heat Shock Proteins in Animal Model | 2009 | Shabkhiz, F and Goodarzi, M and Rajabi, R and Shahrokhi, S | Excluded | The article was excluded based on title and abstract screening as it did not meet the inclusion and exclusion criteria. |
| Effects of physical and psychological stressors on behavior, macrophage activity, and Ehrlich tumor growth | 2003 | Palermo-Neto, J and Massoco, CD and de Souza, WR | Excluded | The article was excluded based on title and abstract screening as it did not meet the inclusion and exclusion criteria. |
| Exposure to chronic stressor upsurges the excitability of serotoninergic neurons and diminishes concentrations of circulating corticosteroids in rats two weeks thereafter | 2022 | Dremencov, E and Grinchii, D and Hrivikova, K and Lapshin, M and Komelkova, M and Graban, J and Puhova, A and Tseilikman, O and Tseilikman, V and Jezova, D | Excluded | The article was excluded based on title and abstract screening as it did not meet the inclusion and exclusion criteria. |
| Aging and Susceptibility to Toluene in Rats: A Pharmacokinetic, Biomarker, and Physiological Approach | 2010 | Gordon, CJ and Gottipolu, RR and Kenyon, EM and Thomas, R and Schladweiler, MC and Mack, CM and Shannahan, JH and Wallenborn, JG and Nyska, A and MacPhail, RC and Richards, JE and Devito, M and Kodavanti, UP | Excluded | The article was excluded based on title and abstract screening as it did not meet the inclusion and exclusion criteria. |
| Clinical and Preclinical Systematic Review of<i>Panax ginseng</i>C. A. Mey and Its Compounds for Fatigue | 2020 | Jin, TY and Rong, PQ and Liang, HY and Zhang, PP and Zheng, GQ and Lin, Y | Excluded | The article was excluded based on title and abstract screening as it did not meet the inclusion and exclusion criteria. |
| Effects of exercise and dietary epigallocatechin gallate and β-alanine on skeletal muscle in aged mice | 2016 | Pence, BD and Gibbons, TE and Bhattacharya, TK and Mach, H and Ossyra, JM and Petr, G and Martin, SA and Wang, L and Rubakhin, SS and Sweedler, JV and McCusker, RH and Kelley, KW and Rhodes, JS and Johnson, RW and Woods, JA | Excluded | The article was excluded based on title and abstract screening as it did not meet the inclusion and exclusion criteria. |
| Central infusion of interleukin-1 receptor antagonist blocks the reduction in social behavior produced by prior stressor exposure | 2009 | Arakawa, H and Blandino, P and Deak, T | Excluded | The article was excluded based on title and abstract screening as it did not meet the inclusion and exclusion criteria. |
| NEUROBIOLOGY OF FIBROMYALGIA AND CHRONIC WIDESPREAD PAIN | 2016 | Sluka, KA and Clauw, DJ | Excluded | The article was excluded based on title and abstract screening as it did not meet the inclusion and exclusion criteria. |
| Activity wheel running reduces escape latency and alters brain monoamine levels after footshock | 1997 | Dishman, RK and Renner, KJ and Youngstedt, SD and Reigle, TG and Bunnell, BN and Burke, KA and Yoo, HS and Mougey, EH and Meyerhof, JL | Excluded | The article was excluded based on title and abstract screening as it did not meet the inclusion and exclusion criteria. |
| It takes two to tango: Widening our understanding of the onset of schizophrenia from a neuro-angiogenic perspective | 2022 | Casas, BS and Arancibia-Altamirano, D and Acevedo-La Rosa, F and Garrido-Jara, D and Maksaev, V and Pérez-Monje, D and Palma, V | Excluded | The article was excluded based on title and abstract screening as it did not meet the inclusion and exclusion criteria. |
| The SHR Y chromosome increases cardiovascular, endocrine, and behavioral responses to stress compared to the WKY Y chromosome | 2012 | Dickey, C and Toot, J and Terwilliger, M and Payne, R and Turner, M and Ely, D | Excluded | The article was excluded based on title and abstract screening as it did not meet the inclusion and exclusion criteria. |
| Probiotic <i>Lactobacillus casei</i> strain Shirota relieves stress-associated symptoms by modulating the gut-brain interaction in human and animal models | 2016 | Takada, M and Nishida, K and Kataoka-Kato, A and Gondo, Y and Ishikawa, H and Suda, K and Kawai, M and Hoshi, R and Watanabe, O and Igarashi, T and Kuwano, Y and Miyazaki, K and Rokutan, K | Excluded | The article was excluded based on title and abstract screening as it did not meet the inclusion and exclusion criteria. |
| Stress biomarkers in a rat model of decompression sickness | 2007 | Montcalm-Smith, E and Caviness, J and Chen, Y and McCarron, RM | Excluded | The article was excluded based on title and abstract screening as it did not meet the inclusion and exclusion criteria. |
| The role of the autonomic nervous system in polycystic ovary syndrome | 2024 | Yu, Y and Chen, T and Zheng, Z and Jia, F and Liao, Y and Ren, YH and Liu, XM and Liu, Y | Excluded | The article was excluded based on title and abstract screening as it did not meet the inclusion and exclusion criteria. |
| Negligible influence of moderate to severe hyperthermia on blood-brain barrier permeability and neuronal parenchymal integrity in healthy men | 2021 | Shepley, BR and Ainslie, PN and Hoiland, RL and Donnelly, J and Sekhon, MS and Zetterberg, H and Blennow, K and Bain, AR | Excluded | The article was excluded based on title and abstract screening as it did not meet the inclusion and exclusion criteria. |
| Gene-environment interactions and construct validity in preclinical models of psychiatric disorders | 2011 | Burrows, EL and McOmish, CE and Hannan, AJ | Excluded | The article was excluded based on title and abstract screening as it did not meet the inclusion and exclusion criteria. |
| Dominance status alters restraint-induced neural activity in brain regions controlling stress vulnerability | 2017 | Cooper, MA and Seddighi, S and Barnes, AK and Grizzell, JA and Dulka, BN and Clinard, CT | Excluded | The article was excluded based on title and abstract screening as it did not meet the inclusion and exclusion criteria. |
| The impact of acute and chronic catecholamines on respiratory responses to hypoxic stress in the rat | 2013 | Hauton, D and Holmes, A and Ziff, O and Kumar, P | Excluded | The article was excluded based on title and abstract screening as it did not meet the inclusion and exclusion criteria. |
| Effect of <i>Salvia officinalis</i> and <i>S</i>. <i>sclarea</i> on rats with a high-fat hypercaloric diet | 2021 | Lieshchova, MA and Bohomaz, AA and Brygadyrenko, VV | Excluded | The article was excluded based on title and abstract screening as it did not meet the inclusion and exclusion criteria. |
| Abdominal surgery increases activity in several phoenixin immunoreactive nuclei | 2023 | Friedrich, T and Goebel-Stengel, M and Schalla, MA and Kobelt, P and Rose, M and Stengel, A | Excluded | The article was excluded based on title and abstract screening as it did not meet the inclusion and exclusion criteria. |
| Vertebrate brains at the pilot light | 2004 | Lutz, PL and Nilsson, GE | Excluded | The article was excluded based on title and abstract screening as it did not meet the inclusion and exclusion criteria. |
| Sagan Dalya Tea, a New "Old" Probable Adaptogenic Drug: Metabolic Characterization and Bioactivity Potentials of <i>Rhododendron adamsii</i> Leaves | 2021 | Olennikov, DN and Nikolaev, VM and Chirikova, NK | Excluded | The article was excluded based on title and abstract screening as it did not meet the inclusion and exclusion criteria. |
| Anti-Stress Effects of <i>d</i>-Limonene and Its Metabolite Perillyl Alcohol | 2014 | d'Alessio, PA and Bisson, JF and Béné, MC | Excluded | The article was excluded based on title and abstract screening as it did not meet the inclusion and exclusion criteria. |
| A neuromechanical model for <i>Drosophila</i> larval crawling based on physical measurements | 2022 | Sun, XY and Liu, YT and Liu, C and Mayumi, K and Ito, K and Nose, A and Kohsaka, H | Excluded | The article was excluded based on title and abstract screening as it did not meet the inclusion and exclusion criteria. |
| Perinatal Depression and the Role of Synaptic Plasticity in Its Pathogenesis and Treatment | 2023 | Shenoy, S and Ibrahim, S | Excluded | The article was excluded based on title and abstract screening as it did not meet the inclusion and exclusion criteria. |
| Induction of distinct neuroinflammatory markers and gut dysbiosis by differential pyridostigmine bromide dosing in a chronic mouse model of GWI showing persistent exercise fatigue and cognitive impairment | 2022 | Kozlova, EV and Carabelli, B and Bishay, AE and Liu, R and Denys, ME and Macbeth, JC and Piamthai, V and Crawford, MS and McCole, DF and Zur Nieden, NI and Hsiao, A and Curras-Collazo, MC | Excluded | The article was excluded based on title and abstract screening as it did not meet the inclusion and exclusion criteria. |
| Chronic psychosocial stress reduces the density of dopamine transporters | 2000 | Isovich, E and Mijnster, MJ and Flügge, G and Fuchs, E | Excluded | The article was excluded based on title and abstract screening as it did not meet the inclusion and exclusion criteria. |
| Effects of <i>Ipomoea carnea</i> aqueous fraction intake by dams during pregnancy on the physical and neurobehavioral development of rat offspring | 2003 | Schwarz, A and Górnak, SL and Bernardi, MM and Dagli, MLZ and Spinosa, HS | Excluded | The article was excluded based on title and abstract screening as it did not meet the inclusion and exclusion criteria. |
| Attenuation of Acute and Chronic Restraint Stress-induced Perturbations in Experimental Animals by <i>Nelumbo nucifera</i> Gaertn | 2008 | Kulkarni, MP and Juvekar, AR | Excluded | The article was excluded based on title and abstract screening as it did not meet the inclusion and exclusion criteria. |
| Beta <i>N</i>-acetylglucosaminyltransferase V (Mgat5) deficiency reduces the depression-like phenotype in mice | 2008 | Soleimani, L and Roder, JC and Dennis, JW and Lipina, T | Excluded | The article was excluded based on title and abstract screening as it did not meet the inclusion and exclusion criteria. |
| Brain-Derived Neurotrophic Factor, Depression, and Physical Activity: Making the Neuroplastic Connection | 2017 | Phillips, C | Excluded | The article was excluded based on title and abstract screening as it did not meet the inclusion and exclusion criteria. |
| Analysis of the anxiolytic-like effect of TRH and the response of amygdalar TRHergic neurons in anxiety | 2008 | Gutiérrez-Mariscal, M and de Gortari, P and López-Rubalcava, C and Martínez, A and Joseph-Bravo, P | Excluded | The article was excluded based on title and abstract screening as it did not meet the inclusion and exclusion criteria. |
| Response of mice to continuous 5-day passive hyperthermia resembles human heat acclimation | 2011 | Sareh, H and Tulapurkar, ME and Shah, NG and Singh, IS and Hasday, JD | Excluded | The article was excluded based on title and abstract screening as it did not meet the inclusion and exclusion criteria. |
| Remarkable cell recovery from cerebral ischemia in rats using an adaptive escalator-based rehabilitation mechanism | 2019 | Chen, CC and Wang, YL and Chang, CP | Excluded | The article was excluded based on title and abstract screening as it did not meet the inclusion and exclusion criteria. |
| The E3 Ubiquitin Ligase CRL5 Regulates Dentate Gyrus Morphogenesis, Adult Neurogenesis, and Animal Behavior | 2022 | Reyes, RV and Hino, K and Canales, CP and Dickson, EJ and La Torre, A and Simo, S | Excluded | The article was excluded based on title and abstract screening as it did not meet the inclusion and exclusion criteria. |
| INCREASED BLOOD-BRAIN-BARRIER PERMEABILITY FOLLOWING ACUTE SHORT-TERM SWIMMING EXERCISE IN CONSCIOUS NORMOTENSIVE YOUNG-RATS | 1991 | SHARMA, HS and CERVOSNAVARRO, J and DEY, PK | Excluded | The article was excluded based on title and abstract screening as it did not meet the inclusion and exclusion criteria. |
| Cryopreservation of rat cortical synaptosomes and analysis of glucose and glutamate transporter activities, and mitochondrial function | 1998 | Begley, JG and Butterfield, DA and Keller, JN and Koppal, T and Drake, J and Mattson, MP | Excluded | The article was excluded based on title and abstract screening as it did not meet the inclusion and exclusion criteria. |
| The glucocorticoid system is required for the voluntary exercise-induced enhancement of learning and memory in rats | 2011 | Hajisoltani, R and Rashidy-Pour, A and Vafaei, AA and Ghaderdoost, B and Bandegi, AR and Motamedi, F | Excluded | The article was excluded based on title and abstract screening as it did not meet the inclusion and exclusion criteria. |
| Modifications of arterial baroreflexes: Obligatory roles in cardiovascular regulation in stress and poststress recovery | 1996 | Nosaka, S | Excluded | The article was excluded based on title and abstract screening as it did not meet the inclusion and exclusion criteria. |
| Density-induced social stress alters oxytocin and vasopressin activities in the brain of a small rodent species | 2021 | Huang, SL and Li, GL and Pan, YL and Song, MJ and Zhao, JD and Wan, XR and Krebs, CJ and Wang, ZX and Han, WN and Zhang, ZB | Excluded | The article was excluded based on title and abstract screening as it did not meet the inclusion and exclusion criteria. |
| Bioaccumulation and ecotoxicological responses of juvenile white seabream (<i>Diplodus sargus</i>) exposed to triclosan, warming and acidification | 2019 | Maulvault, AL and Camacho, C and Barbosa, V and Alves, R and Anacleto, P and Cunha, SC and Fernandes, JO and Pousao-Ferreira, P and Paula, JR and Rosa, R and Diniz, M and Marques, A | Excluded | The article was excluded based on title and abstract screening as it did not meet the inclusion and exclusion criteria. |
| Effect of aging and late onset dietary restriction on antioxidant enzymes and proteasome activities, and protein carbonylation of rat and skeletal muscle and tendon | 2002 | Radák, Z and Takahashi, R and Kumiyama, A and Nakamoto, H and Ohno, H and Ookawara, T and Goto, S | Excluded | The article was excluded based on title and abstract screening as it did not meet the inclusion and exclusion criteria. |
| Early life stress, the development of aggression and neuroendocrine and neurobiological correlates: What can we learn from animal models? | 2009 | Veenema, AH | Excluded | The article was excluded based on title and abstract screening as it did not meet the inclusion and exclusion criteria. |
| Animal model for progressive resistance exercise: a detailed description of model and its implications for basic research in exercise | 2013 | Cassilhas, RC and Reis, IT and Venâncio, D and Fernandes, J and Tufik, S and de Mello, MT | Excluded | The article was excluded based on title and abstract screening as it did not meet the inclusion and exclusion criteria. |
| Stress adversely affects efficacy of physostigmine-scopolamine pretreatment against soman in guinea pigs | 2005 | Philippens, IHCHM and Joosen, MJA and Vanwersch, RAP | Excluded | The article was excluded based on title and abstract screening as it did not meet the inclusion and exclusion criteria. |
| Possible use of repeated cold stress for reducing fatigue in chronic fatigue syndrome: a hypothesis | 2007 | Shevchuk, NA | Excluded | The article was excluded based on title and abstract screening as it did not meet the inclusion and exclusion criteria. |
| Exercise alters the profile of phospholipid molecular species in rat skeletal muscle | 2004 | Mitchell, TW and Turner, N and Hulbert, AJ and Else, PL and Hawley, JA and Lee, JS and Bruce, CR and Blanksby, SJ | Excluded | The article was excluded based on title and abstract screening as it did not meet the inclusion and exclusion criteria. |
| Neurogenesis-independent antidepressant-like effects of enriched environment is dependent on adiponectin | 2015 | Nicolas, S and Veyssière, J and Gandin, C and Zsürger, N and Pietri, M and Heurteaux, C and Glaichenhaus, N and Petit-Paitel, A and Chabry, J | Excluded | The article was excluded based on title and abstract screening as it did not meet the inclusion and exclusion criteria. |
| Physical predictors, behavioural/emotional attributes and neurochemical determinants of dominant behaviour | 2014 | Chichinadze, K and Chichinadze, N and Gachechiladze, L and Lazarashvili, A and Nikolaishvili, M | Excluded | The article was excluded based on title and abstract screening as it did not meet the inclusion and exclusion criteria. |
| Analysis of NAPA Gene Expression in Brain Structures of Wistar Rats during the Formation of Long-Term Spatial Memory and Physical Activity under Stress Situation | 2023 | Gruden, MA and Ratmirov, AM and Storozheva, ZI and Sewell, RDE | Excluded | The article was excluded based on title and abstract screening as it did not meet the inclusion and exclusion criteria. |
| Calmatives for the excitable horse: A review of L-tryptophan | 2005 | Grimmett, A and Sillence, MN | Excluded | The article was excluded based on title and abstract screening as it did not meet the inclusion and exclusion criteria. |
| Adolescent stress and neural plasticity in hamsters: a vasopressin-serotonin model of inappropriate aggressive behaviour | 2000 | Ferris, CF | Excluded | The article was excluded based on title and abstract screening as it did not meet the inclusion and exclusion criteria. |
| Impaired mitochondrial complex I function as a candidate driver in the biological stress response and a concomitant stress-induced brain metabolic reprogramming in male mice | 2020 | Emmerzaal, TL and Preston, G and Geenen, B and Verweij, V and Wiesmann, M and Vasileiou, E and Grüter, F and de Groot, C and Schoorl, J and de Veer, R and Roelofs, M and Arts, M and Hendriksen, Y and Klimars, E and Donti, TR and Graham, BH and Morava, E and Rodenburg, RJ and Kozicz, T | Excluded | The article was excluded based on title and abstract screening as it did not meet the inclusion and exclusion criteria. |
| Selective brain cooling in mammals and birds | 2001 | Jessen, C | Excluded | The article was excluded based on title and abstract screening as it did not meet the inclusion and exclusion criteria. |
| Swimming reduces the severity of physical and psychological dependence and voluntary morphine consumption in morphine dependent rats | 2015 | Fadaei, A and Gorji, HM and Hosseini, SM | Excluded | The article was excluded based on title and abstract screening as it did not meet the inclusion and exclusion criteria. |
| Effects of prenatal exposure to manganese on postnatal development and behavior in mice: Influence of maternal restraint | 2002 | Torrente, M and Colomina, MT and Domingo, JL | Excluded | The article was excluded based on title and abstract screening as it did not meet the inclusion and exclusion criteria. |
| <i>Aralia elata</i> var. <i>mandshurica</i> (Rupr. & Maxim.) J.Wen: An overview of pharmacological studies | 2016 | Shikov, AN and Pozharitskaya, ON and Makarov, VG | Excluded | The article was excluded based on title and abstract screening as it did not meet the inclusion and exclusion criteria. |
| Crayfish <i>Procambarus clarkii</i> shows circadian variations in different parameters of the GSH cycle | 2001 | Durán-Lizarraga, ME and Prieto-Sagredo, J and Gonsebatt, ME and Fanjul-Moles, ML | Excluded | The article was excluded based on title and abstract screening as it did not meet the inclusion and exclusion criteria. |
| The effects of high intensity exercise on learning and memory impairments followed by combination of sleep deprivation and demyelination induced by etidium bromide | 2019 | Rajizadeh, MA and Sheibani, V and Bejeshk, MA and Borzadaran, FM and Saghari, H and Esmaeilpour, K | Excluded | The article was excluded based on title and abstract screening as it did not meet the inclusion and exclusion criteria. |
| The Phenotypic Patterns of Essential Hypertension Are the Key to Identifying "High Blood Pressure" Genes | 2010 | Korner, PI | Excluded | The article was excluded based on title and abstract screening as it did not meet the inclusion and exclusion criteria. |
| Mild exercise increases dihydrotestosterone in hippocampus providing evidence for androgenic mediation of neurogenesis | 2012 | Okamoto, M and Hojo, Y and Inoue, K and Matsui, T and Kawato, S and McEwen, BS and Soya, H | Excluded | The article was excluded based on title and abstract screening as it did not meet the inclusion and exclusion criteria. |
| New Insights into Depressive Disorder with Respect to Low-Grade Inflammation and Fish Oil Intake | 2021 | Tung, TH and Nguyen, NTK and Huang, SY | Excluded | The article was excluded based on title and abstract screening as it did not meet the inclusion and exclusion criteria. |
| Priming for welfare: gut microbiota is associated with equitation conditions and behavior in horse athletes | 2020 | Mach, N and Ruet, A and Clark, A and Bars-Cortina, D and Ramayo-Caldas, Y and Crisci, E and Pennarun, S and Dhorne-Pollet, S and Foury, A and Moisan, MP and Lansade, L | Excluded | The article was excluded based on title and abstract screening as it did not meet the inclusion and exclusion criteria. |
| STRESS AND INTERLEUKIN-1-BETA-INDUCED ACTIVATION OF C-FOS, NGFI-B AND CRF GENE-EXPRESSION THE HYPOTHALAMIC PVN - COMPARISON BETWEEN SPRAGUE-DAWLEY, FISHER-344 AND LEWIS RATS | 1994 | RIVEST, S and RIVIER, C | Excluded | The article was excluded based on title and abstract screening as it did not meet the inclusion and exclusion criteria. |
| Potential effects of anesthetic agents on heat shock proteins in the laboratory rat | 2003 | Kilgore, JL and Ross, CR and Saunders, DK | Excluded | The article was excluded based on title and abstract screening as it did not meet the inclusion and exclusion criteria. |
| Distinct metabolic adjustments arise from acclimation to constant hypoxia and intermittent hypoxia in estuarine killifish (<i>Fundulus heteroclitus</i>) | 2018 | Borowiec, BG and McClelland, GB and Rees, BB and Scott, GR | Excluded | The article was excluded based on title and abstract screening as it did not meet the inclusion and exclusion criteria. |
| Multiple Sclerosis and Neuroplasticity: Pathogenesis and Therapeutic Projections Events | 2009 | Andrade-Talavera, Y and Bergado, JA and Pentón-Rol, G | Excluded | The article was excluded based on title and abstract screening as it did not meet the inclusion and exclusion criteria. |
| Lifestyle Interventions Improving Cannabinoid Tone During COVID-19 Lockdowns May Enhance Compliance With Preventive Regulations and Decrease Psychophysical Health Complications | 2021 | Brugnatelli, V and Facco, E and Zanette, G | Excluded | The article was excluded based on title and abstract screening as it did not meet the inclusion and exclusion criteria. |
| Toxicity and effects of copper oxide nanoparticles on cognitive performances in rats | 2020 | Ouni, S and Askri, D and Jeljeli, M and Abdelmalek, H and Sakly, M and Amara, S | Excluded | The article was excluded based on title and abstract screening as it did not meet the inclusion and exclusion criteria. |
| IRON-DEFICIENCY - NONHEMATOLOGICAL MANIFESTATIONS | 1991 | PRASAD, AN and PRASAD, C | Excluded | The article was excluded based on title and abstract screening as it did not meet the inclusion and exclusion criteria. |
| Effects of early rearing enrichments on modulation of brain monoamines and hypothalamic-pituitary-interrenal axis (HPI axis) of fish mahseer (<i>Tor putitora</i>) | 2020 | Ullah, I and Zuberi, A and Rehman, H and Ali, Z and Thornqvist, PO and Winberg, S | Excluded | The article was excluded based on title and abstract screening as it did not meet the inclusion and exclusion criteria. |
| The Link Between Stress and Endometriosis: from Animal Models to the Clinical Scenario | 2020 | Appleyard, CB and Flores, I and Torres-Reverón, A | Excluded | The article was excluded based on title and abstract screening as it did not meet the inclusion and exclusion criteria. |
| Swimming exercise reverses CUMS-induced changes in depression-like behaviors and hippocampal plasticity-related proteins | 2018 | Liu, WN and Xue, XL and Xia, J and Liu, JT and Qi, ZT | Excluded | The article was excluded based on title and abstract screening as it did not meet the inclusion and exclusion criteria. |
| Early use of oleanolic acid provides protection against 6-hydroxydopamine induced dopamine neurodegeneration | 2015 | Mabandla, MV and Nyoka, M and Daniels, WMU | Excluded | The article was excluded based on title and abstract screening as it did not meet the inclusion and exclusion criteria. |
| Repression of Tau Hyperphosphorylation by Chronic Endurance Exercise in Aged Transgenic Mouse Model of Tauopathies | 2009 | Leem, YH and Lim, HJ and Shim, SB and Cho, JY and Kim, BS and Han, PL | Excluded | The article was excluded based on title and abstract screening as it did not meet the inclusion and exclusion criteria. |
| Assessment of Myocardial Blood Flow (MBF) in Humans Using Arterial Spin Labeling (ASL): Feasibility and Noise Analysis | 2009 | Zun, ZH and Wong, EC and Nayak, KS | Excluded | The article was excluded based on title and abstract screening as it did not meet the inclusion and exclusion criteria. |
| Modulation of the hippocampal protein machinery in voluntary and treadmill exercising rats | 2008 | Chen, WQ and Diao, WF and Viidik, A and Skalicky, M and Höger, H and Lubec, G | Excluded | The article was excluded based on title and abstract screening as it did not meet the inclusion and exclusion criteria. |
| The polychlorinated biphenyls (PCBS) environmental pollutants and their effects on the Nervous System and health | 2009 | Miller-Pérez, C and Sánchez-Islas, E and Mucio-Ramírez, S and Mendoza-Sotelo, J and León-Olea, M | Excluded | The article was excluded based on title and abstract screening as it did not meet the inclusion and exclusion criteria. |
| Physical and social enrichment influences the adaptability-related behaviors of black rockfish <i>Sebastes schlegelii</i>: An effect mediated by social behaviors, HPI axis and neurogenesis | 2023 | Zhang, ZH and Chen, QK and Guan, XT and Gong, MY and Zhang, JT and Cheng, F and Zhang, XM | Excluded | The article was excluded based on title and abstract screening as it did not meet the inclusion and exclusion criteria. |
| Differential effects of chronic escapable versus inescapable stress on male syrian hamster (<i>Mesocricetus auratus</i>) reproductive behavior | 2003 | Holmer, HK and Rodman, JE and Helmreich, DL and Parfitt, DB | Excluded | The article was excluded based on title and abstract screening as it did not meet the inclusion and exclusion criteria. |
| Repeated mild traumatic brain injury causes sex-specific increases in cell proliferation and inflammation in juvenile rats | 2023 | Neale, KJ and Reid, HMO and Sousa, B and Mcdonagh, E and Morrison, J and Shultz, S and Eyolfson, E and Christie, BR | Excluded | The article was excluded based on title and abstract screening as it did not meet the inclusion and exclusion criteria. |
| Ginsenoside Rg3 modulates spatial memory and fear memory extinction by the HPA axis and BDNF-TrkB pathway in a rat post-traumatic stress disorder | 2022 | Sur, B and Lee, B | Excluded | The article was excluded based on title and abstract screening as it did not meet the inclusion and exclusion criteria. |
| Heatstroke, susceptibility to heat and stress | 2002 | Bourdon, L and Canini, F and Buguet, A | Excluded | The article was excluded based on title and abstract screening as it did not meet the inclusion and exclusion criteria. |
| Voluntary Exercise During Adolescence Mitigated Negative the Effects of Maternal Separation Stress on the Depressive-Like Behaviors of Adult Male Rats: Role of NMDA Receptors | 2018 | Masrour, FF and Peeri, M and Azarbayjani, MA and Hosseini, MJ | Excluded | The article was excluded based on title and abstract screening as it did not meet the inclusion and exclusion criteria. |
| Anhedonia and motivational deficits in rats:: Impact of chronic social stress | 2005 | Rygula, R and Abumaria, N and Flügge, G and Fuchs, E and Rüther, E and Havemann-Reinecke, U | Excluded | The article was excluded based on title and abstract screening as it did not meet the inclusion and exclusion criteria. |
| Physical and Environmental Stressors Induces Region Specific Neuronal Damage in Hippocampal Subfields of Mice | 2012 | Meena, P and Sharma, D and Jain, A and Sukhwal, P and Suhalka, P and Bhatnagar, M | Excluded | The article was excluded based on title and abstract screening as it did not meet the inclusion and exclusion criteria. |
| The neurobiology of stress and development | 2007 | Gunnar, M and Quevedo, K | Excluded | The article was excluded based on title and abstract screening as it did not meet the inclusion and exclusion criteria. |
| Childhood physical abuse predicts stressor-evoked activity within central visceral control regions | 2015 | Banihashemi, L and Sheu, LK and Midei, AJ and Gianaros, PJ | Excluded | The article was excluded based on title and abstract screening as it did not meet the inclusion and exclusion criteria. |
| The Emerging Role of Atrial Natriuretic Peptide in Psychiatry | 2021 | Marazziti, D and Barberi, FM and Mucci, F and Maglio, A and Dell'Oste, V and Dell'Osso, L | Excluded | The article was excluded based on title and abstract screening as it did not meet the inclusion and exclusion criteria. |
| Altered behavior and neural activity in conspecific cagemates co-housed with mouse models of brain disorders | 2016 | Yang, H and Jung, S and Seo, J and Khalid, A and Yoo, JS and Park, J and Kim, S and Moon, J and Lee, ST and Jung, KH and Chu, K and Lee, SK and Jeon, D | Excluded | The article was excluded based on title and abstract screening as it did not meet the inclusion and exclusion criteria. |
| Metabolism of monoamines in invertebrates: The relative importance of monoamine oxidase in different phyla | 2004 | Sloley, BD | Excluded | The article was excluded based on title and abstract screening as it did not meet the inclusion and exclusion criteria. |
| The Role of Microbiome in Brain Development and Neurodegenerative Diseases | 2022 | Nandwana, V and Nandwana, NK and Das, Y and Saito, M and Panda, T and Das, S and Almaguel, F and Hosmane, NS and Das, BC | Excluded | The article was excluded based on title and abstract screening as it did not meet the inclusion and exclusion criteria. |
| Brain-derived neurotrophic factor and its receptor tropomyosin-related kinase B in the mechanism of action of antidepressant therapies | 2008 | Kozisek, ME and Middlemas, D and Bylund, DB | Excluded | The article was excluded based on title and abstract screening as it did not meet the inclusion and exclusion criteria. |
| Object location and object recognition memory impairments, motivation deficits and depression in a model of Gulf War illness | 2014 | Hattiangady, B and Mishra, V and Kodali, M and Shuai, B and Rao, X and Shetty, AK | Excluded | The article was excluded based on title and abstract screening as it did not meet the inclusion and exclusion criteria. |
| Metabolic scaling: a many-splendoured thing | 2004 | Suarez, RK and Darveau, CA and Childress, JJ | Excluded | The article was excluded based on title and abstract screening as it did not meet the inclusion and exclusion criteria. |
| Housing conditions and sacrifice protocol affect neural activity and vocal behavior in a songbird species, the zebra finch (<i>Taeniopygia guttata</i>) | 2015 | Elie, JE and Soula, HA and Trouvé, C and Mathevon, N and Vignal, C | Excluded | The article was excluded based on title and abstract screening as it did not meet the inclusion and exclusion criteria. |
| c-Fos mapping of brain regions activated by multi-modal and electric foot shock stress | 2018 | Lin, XX and Itoga, CA and Taha, S and Li, MH and Chen, R and Sami, K and Berton, F and Francesconi, W and Xu, XM | Excluded | The article was excluded based on title and abstract screening as it did not meet the inclusion and exclusion criteria. |
| Running wheel exercise reduces α-synuclein aggregation and improves motor and cognitive function in a transgenic mouse model of Parkinson's disease | 2017 | Zhou, WB and Barkow, JC and Freed, CR | Excluded | The article was excluded based on title and abstract screening as it did not meet the inclusion and exclusion criteria. |
| Why may allopregnanolone help alleviate loneliness? | 2015 | Cacioppo, S and Cacioppo, JT | Excluded | The article was excluded based on title and abstract screening as it did not meet the inclusion and exclusion criteria. |
| Cultivating a healthy neuro-immune network: A health psychology approach | 2019 | Bower, JE and Kuhlman, KR and Haydon, MD and Boyle, CC and Radin, A | Excluded | The article was excluded based on title and abstract screening as it did not meet the inclusion and exclusion criteria. |
| Brain-Derived Neurotrophic Factor, Kynurenine Pathway, and Lipid-Profiling Alterations as Potential Animal Welfare Indicators in Dairy Cattle | 2023 | Favole, A and Testori, C and Bergagna, S and Gennero, MS and Ingravalle, F and Costa, B and Barresi, S and Curti, P and Barberis, F and Ganio, S and Orusa, R and Costassa, EV and Berrone, E and Vernè, M and Scaglia, M and Palmitessa, C and Gallo, M and Tessarolo, C and Pederiva, S and Ferrari, A and Lorenzi, V and Fusi, F and Brunelli, L and Pastorelli, R and Cagnotti, G and Casalone, C and Caramelli, M and Corona, C | Excluded | The article was excluded based on title and abstract screening as it did not meet the inclusion and exclusion criteria. |
| Repetitive Concussive Traumatic Brain Injury Interacts with Post-Injury Foot Shock Stress to Worsen Social and Depression-Like Behavior in Mice | 2013 | Klemenhagen, KC and O'Brien, SP and Brody, DL | Excluded | The article was excluded based on title and abstract screening as it did not meet the inclusion and exclusion criteria. |
| Effects of Maternal Separation and Subsequent Stress on Behaviors and Brain Monoamines in Rats | 2023 | Mavrenkova, PV and Khlebnikova, NN and Alchinova, IB and Demorzhi, MS and Shoibonov, BB and Karganov, MY | Excluded | The article was excluded based on title and abstract screening as it did not meet the inclusion and exclusion criteria. |
| Bactericidal and Cytotoxic Properties of Silver Nanoparticles | 2019 | Liao, CZ and Li, YC and Tjong, SC | Excluded | The article was excluded based on title and abstract screening as it did not meet the inclusion and exclusion criteria. |
| NEUROENDOCRINE AND NEUROCHEMICAL CONSEQUENCES OF LONG-TERM SLEEP-DEPRIVATION IN RATS - SIMILARITIES TO SOME FEATURES OF DEPRESSION | 1991 | PATCHEV, V and FELSZEGHY, K and KORANYI, L | Excluded | The article was excluded based on title and abstract screening as it did not meet the inclusion and exclusion criteria. |
| Cannabidiol has a unique effect on global brain activity: a pharmacological, functional MRI study in awake mice | 2021 | Sadaka, AH and Ozuna, AG and Ortiz, RJ and Kulkarni, P and Johnson, CT and Bradshaw, HB and Cushing, BS and Li, AL and Hohmann, AG and Ferris, CF | Excluded | The article was excluded based on title and abstract screening as it did not meet the inclusion and exclusion criteria. |
| Effect of a nutritive-tonic drink on stress-induced serum glucocorticoid in the mouse | 2004 | Sakata, Y | Excluded | The article was excluded based on title and abstract screening as it did not meet the inclusion and exclusion criteria. |
| THE ROLE OF BRAIN-SEROTONIN - A NEUROPHYSIOLOGIC PERSPECTIVE | 1990 | JACOBS, BL and WILKINSON, LO and FORNAL, CA | Excluded | The article was excluded based on title and abstract screening as it did not meet the inclusion and exclusion criteria. |
| Reduced voluntary running performance is associated with impaired coordination as a result of muscle satellite cell depletion in adult mice | 2015 | Jackson, JR and Kirby, TJ and Fry, CS and Cooper, RL and McCarthy, JJ and Peterson, CA and Dupont-Versteegden, EE | Excluded | The article was excluded based on title and abstract screening as it did not meet the inclusion and exclusion criteria. |
| Regular Moderate or Intense Exercise Prevents Depression-Like Behavior without Change of Hippocampal Tryptophan Content in Chronically Tryptophan-Deficient and Stressed Mice | 2013 | Lee, H and Ohno, M and Ohta, S and Mikami, T | Excluded | The article was excluded based on title and abstract screening as it did not meet the inclusion and exclusion criteria. |
| Pharmacology of <i>Schisandra chinensis</i> Bail.:: An overview of Russian research and uses in medicine | 2008 | Panossian, A and Wikman, G | Excluded | The article was excluded based on title and abstract screening as it did not meet the inclusion and exclusion criteria. |
| Neuroimmune Interactions in Stress | 2010 | Costa-Pinto, FA and Palermo-Neto, J | Excluded | The article was excluded based on title and abstract screening as it did not meet the inclusion and exclusion criteria. |
| Training Rats to Voluntarily Dive Underwater: Investigations of the Mammalian Diving Response | 2014 | McCulloch, PF | Excluded | The article was excluded based on title and abstract screening as it did not meet the inclusion and exclusion criteria. |
| Behavioral and endocrine evaluation of the stressfulness of single-pen housing compared to group-housing and social isolation conditions | 2015 | Guesdon, V and Meurisse, M and Chesneau, D and Picard, S and Lévy, F and Chaillou, E | Excluded | The article was excluded based on title and abstract screening as it did not meet the inclusion and exclusion criteria. |
| The increase in TNF-α levels is implicated in NF-κB activation and inducible nitric oxide synthase expression in brain cortex after immobilization stress | 2002 | Madrigal, JLM and Hurtado, O and Moro, MA and Lizasoain, I and Lorenzo, P and Castrillo, A and Boscá, L and Leza, JC | Excluded | The article was excluded based on title and abstract screening as it did not meet the inclusion and exclusion criteria. |
| ADOLESCENT EXPOSURE TO COCAINE INCREASES ANXIETY-LIKE BEHAVIOR AND INDUCES MORPHOLOGIC AND NEUROCHEMICAL CHANGES IN THE HIPPOCAMPUS OF ADULT RATS | 2016 | Zhu, W and Mao, Z and Zhu, C and Li, M and Cao, C and Guan, Y and Yuan, J and Xie, G and Guan, X | Excluded | The article was excluded based on title and abstract screening as it did not meet the inclusion and exclusion criteria. |
| Standardized Environmental Enrichment Supports Enhanced Brain Plasticity in Healthy Rats and Prevents Cognitive Impairment in Epileptic Rats | 2013 | Fares, RP and Belmeguenai, A and Sanchez, PE and Kouchi, HY and Bodennec, J and Morales, A and Georges, B and Bonnet, C and Bouvard, S and Sloviter, RS and Bezin, L | Excluded | The article was excluded based on title and abstract screening as it did not meet the inclusion and exclusion criteria. |
| Adult male rats exposed to an alcohol diet exhibit a blunted adrenocorticotropic hormone response to immune or physical stress: Possible role of nitric oxide | 1995 | Rivier, C | Excluded | The article was excluded based on title and abstract screening as it did not meet the inclusion and exclusion criteria. |
| Spironolactone decreases the somatic signs of opiate withdrawal by blocking the mineralocorticoid receptors (MR) | 2014 | Navarro-Zaragoza, J and Laorden, ML and Milanés, MV | Excluded | The article was excluded based on title and abstract screening as it did not meet the inclusion and exclusion criteria. |
| Hypothalamic-Pituitary-Adrenocortical Dysfunction in Elderly, Male Marathon Runners: Feedback Sensitivity, Stress Response, and Effects on Verbal Memory | 2017 | Deuschle, M and Gotthardt, U and Schweiger, U and Dettling, M and Holsboer, F and Heuser, I | Excluded | The article was excluded based on title and abstract screening as it did not meet the inclusion and exclusion criteria. |
| Hypertension and the metabolic syndrome:: Closely related central origin? | 2000 | Björntorp, P and Holm, G and Rosmond, R and Folkow, B | Excluded | The article was excluded based on title and abstract screening as it did not meet the inclusion and exclusion criteria. |
| A chronic low-dose magnesium L-lactate administration has a beneficial effect on the myocardium and the skeletal muscles | 2022 | Pinto, MM and Dubouchaud, H and Jouve, C and Rigaudière, JP and Patrac, V and Bouvier, D and Hininger-Favier, I and Walrand, S and Demaison, L | Excluded | The article was excluded based on title and abstract screening as it did not meet the inclusion and exclusion criteria. |
| Maternal exercise during pregnancy affects mitochondrial enzymatic activity and biogenesis in offspring brain | 2013 | Park, JW and Kim, MH and Eo, SJ and Lee, EH and Kang, JS and Chang, HK and Leem, YH | Excluded | The article was excluded based on title and abstract screening as it did not meet the inclusion and exclusion criteria. |
| The Effect of β-Alanine Supplementation on Performance, Cognitive Function and Resiliency in Soldiers | 2023 | Ostfeld, I and Hoffman, JR | Excluded | The article was excluded based on title and abstract screening as it did not meet the inclusion and exclusion criteria. |
| Social status and modern-type depression: A review | 2019 | Komori, T and Makinodan, M and Kishimoto, T | Excluded | The article was excluded based on title and abstract screening as it did not meet the inclusion and exclusion criteria. |
| Long-term effects of the periadolescent environment on exploratory activity and aggressive behaviour in mice: social versus physical enrichment | 2004 | Pietropaolo, S and Branchi, I and Cirulli, F and Chiarotti, F and Aloe, L and Alleva, E | Excluded | The article was excluded based on title and abstract screening as it did not meet the inclusion and exclusion criteria. |
| Long-term food restriction prevents aging-associated sphingolipid turnover dysregulation in the brain | 2014 | Babenko, NA and Shakhova, EG | Excluded | The article was excluded based on title and abstract screening as it did not meet the inclusion and exclusion criteria. |
| Type 2 diabetes - An introduction to the development and use of animal models | 2006 | Kaplan, JR and Wagner, JD | Excluded | The article was excluded based on title and abstract screening as it did not meet the inclusion and exclusion criteria. |
| Selective targeting of chronic social stress-induced activated neurons identifies neurogenesis-related genes to be associated with resilience in female mice | 2022 | Guilherme, MD and Tsoutsouli, T and Chongtham, MC and Winter, J and Gerber, S and Müller, MB and Endres, K | Excluded | The article was excluded based on title and abstract screening as it did not meet the inclusion and exclusion criteria. |
| Voluntary alcohol intake after noise exposure in adolescent rats: Hippocampal-related behavioral alterations | 2018 | Miceli, M and Molina, SJ and Forcada, A and Acosta, GB and Guelman, LR | Excluded | The article was excluded based on title and abstract screening as it did not meet the inclusion and exclusion criteria. |
| Brain oxytocin inhibits the (re)activity of the hypothalamo-pituitary-adrenal axis in male rats:: involvement of hypothalamic and limbic brain regions | 2000 | Neumann, ID and Krömer, SA and Toschi, N and Ebner, K | Excluded | The article was excluded based on title and abstract screening as it did not meet the inclusion and exclusion criteria. |
| Antidepressant-like Effects of p-Coumaric Acid on LPS-induced Depressive and Inflammatory Changes in Rats | 2018 | Lee, S and Kim, HB and Hwang, ES and Kim, ES and Kim, SS and Jeon, TD and Song, MC and Lee, JS and Chung, MC and Maeng, S and Park, JH | Excluded | The article was excluded based on title and abstract screening as it did not meet the inclusion and exclusion criteria. |
| The mouse that roared: neural mechanisms of social hierarchy | 2014 | Wang, F and Kessels, HW and Hu, HL | Excluded | The article was excluded based on title and abstract screening as it did not meet the inclusion and exclusion criteria. |
| Pathophysiological interpretation of fetal heart rate tracings in clinical practice | 2023 | Jia, YJ and Ghi, T and Pereira, S and Perez-Bonfils, AG and Chandraharan, E | Excluded | The article was excluded based on title and abstract screening as it did not meet the inclusion and exclusion criteria. |
| Constitutive Neurogenesis in the Brain of Different Vertebrate Groups | 2020 | Stukaneva, ME and Pushchina, EV | Excluded | The article was excluded based on title and abstract screening as it did not meet the inclusion and exclusion criteria. |
| Dual influences of early life stress induced by limited bedding on walking adaptability and <i>Bdnf/TrkB</i> and <i>Drd1</i>/<i>Drd2</i> gene expression in different mouse brain regions | 2019 | Wearick-Silva, LE and Orso, R and Martins, LA and Creutzberg, KC and Centeno-Silva, A and Xavier, LL and Grassi-Oliveira, R and Mestriner, RG | Excluded | The article was excluded based on title and abstract screening as it did not meet the inclusion and exclusion criteria. |
| Therapeutic benefit of environmental enrichment on optic neuritis | 2019 | Aranda, ML and Fleitas, MFG and Dieguez, HH and Milne, GA and Devouassoux, JD and Sarmiento, MIK and Chianelli, M and Sande, PH and Dorfman, D and Rosenstein, RE | Excluded | The article was excluded based on title and abstract screening as it did not meet the inclusion and exclusion criteria. |
| Mouse social stress induces increased fear conditioning, helplessness and fatigue to physical challenge together with markers of altered immune and dopamine function | 2014 | Azzinnari, D and Sigrist, H and Staehli, S and Palme, R and Hildebrandt, T and Leparc, G and Hengerer, B and Seifritz, E and Pryce, CR | Excluded | The article was excluded based on title and abstract screening as it did not meet the inclusion and exclusion criteria. |
| Heat stress, even extreme, does not induce penetration of pyridostigmine into the brain of guinea pigs | 1998 | Lallement, G and Foquin, A and Baubichon, D and Burckhart, MF and Carpentier, P and Canini, F | Excluded | The article was excluded based on title and abstract screening as it did not meet the inclusion and exclusion criteria. |
| Brains in the city: Neurobiological effects of urbanization | 2015 | Lambert, KG and Nelson, RJ and Jovanovic, T and Cerdá, M | Excluded | The article was excluded based on title and abstract screening as it did not meet the inclusion and exclusion criteria. |
| Effect of caffeine supplementation on the extracellular heat shock protein 72 response to exercise | 2006 | Whitham, M and Walker, GJ and Bishop, NC | Excluded | The article was excluded based on title and abstract screening as it did not meet the inclusion and exclusion criteria. |
| Exercise reverses the effects of early life stress on orexin cell reactivity in male but not female rats | 2014 | James, MH and Campbell, EJ and Walker, FR and Smith, DW and Richardson, HN and Hodgson, DM and Dayas, CV | Excluded | The article was excluded based on title and abstract screening as it did not meet the inclusion and exclusion criteria. |
| Announced rewards counteract the impairment of anticipatory behaviour in socially stressed rats | 2005 | van der Harst, JE and Baars, AM and Spruijt, BM | Excluded | The article was excluded based on title and abstract screening as it did not meet the inclusion and exclusion criteria. |
| Animal Models for Anorexia Nervosa-A Systematic Review | 2021 | Scharner, S and Stengel, A | Excluded | The article was excluded based on title and abstract screening as it did not meet the inclusion and exclusion criteria. |
| Effects of chemical and thermal stress on acetylcholinesterase activity in the brain of the bank vole, <i>Myodes glareolus</i> | 2014 | Swiergosz-Kowalewska, R and Molenda, P and Halota, A | Excluded | The article was excluded based on title and abstract screening as it did not meet the inclusion and exclusion criteria. |
| Neurotrophic factor-α1, a novel tropin is critical for the prevention of stress-induced hippocampal CA3 cell death and cognitive dysfunction in mice: comparison to BDNF | 2021 | Xiao, L and Sharma, VK and Toulabi, L and Yang, XY and Lee, C and Abebe, D and Peltekian, A and Arnaoutova, I and Lou, H and Loh, YP | Excluded | The article was excluded based on title and abstract screening as it did not meet the inclusion and exclusion criteria. |
| Neuropeptide Y impairs the acquisition of conditioned defeat in Syrian hamsters | 2019 | Lacey, T and Sweeting, J and Kingston, R and Smith, M and Markham, CM | Excluded | The article was excluded based on title and abstract screening as it did not meet the inclusion and exclusion criteria. |
| ANIMAL MODELS OF FETAL ALCOHOL SPECTRUM DISORDERS: IMPACT OF THE SOCIAL ENVIRONMENT | 2009 | Kelly, SJ and Goodlett, CR and Hannigan, JH | Excluded | The article was excluded based on title and abstract screening as it did not meet the inclusion and exclusion criteria. |
| Housing quality and behavior affect brain health and anxiety in healthy Japanese adults | 2021 | Pineda, JCD and Kokubun, K and Ikaga, T and Yamakawa, Y | Excluded | The article was excluded based on title and abstract screening as it did not meet the inclusion and exclusion criteria. |
| Promoting Successful Cognitive Aging: A Comprehensive Review | 2010 | Daffner, KR | Excluded | The article was excluded based on title and abstract screening as it did not meet the inclusion and exclusion criteria. |
| Environmental and pharmacological modulations of cellular plasticity: Role in the pathophysiology and treatment of depression | 2013 | Ota, KT and Duman, RS | Excluded | The article was excluded based on title and abstract screening as it did not meet the inclusion and exclusion criteria. |
| Effect of voluntary wheel running on neuroactive steroid levels in murine experimental autoimmune encephalomyelitis | 2018 | Mifflin, K and Baker, GB and Kerr, BJ | Excluded | The article was excluded based on title and abstract screening as it did not meet the inclusion and exclusion criteria. |
| Cognitive endophenotypes, gene-environment interactions and experience-dependent plasticity in animal models of schizophrenia | 2016 | Burrows, EL and Hannan, AJ | Excluded | The article was excluded based on title and abstract screening as it did not meet the inclusion and exclusion criteria. |
| Inhibitory effects of forced swim stress and corticosterone on the acquisition but not expression of morphine-induced conditioned place preference: Involvement of glucocorticoid receptor in the basolateral amygdala | 2013 | Attarzadeh-Yazdi, G and Karimi, S and Azizi, P and Yazdi-Ravandi, S and Hesam, S and Haghparast, A | Excluded | The article was excluded based on title and abstract screening as it did not meet the inclusion and exclusion criteria. |
| Brain-derived neurotrophic factor. From nerve growth factor to modulator of brain plasticity in cognitive processes and psychiatric diseases | 2006 | Laske, C and Eschweiler, G | Excluded | The article was excluded based on title and abstract screening as it did not meet the inclusion and exclusion criteria. |
| Social environment enrichment alleviates anxiety-like behavior in mice: Involvement of the dopamine system | 2024 | Wang, Q and Wang, YQ and Tian, YY and Li, YY and Han, J and Tai, FD and Jia, R | Excluded | The article was excluded based on title and abstract screening as it did not meet the inclusion and exclusion criteria. |
| Stress induces altered CRE/CREB pathway activity and BDNF expression in the hippocampus of glucocorticoid receptor-impaired mice | 2011 | Alboni, S and Tascedda, F and Corsini, D and Benatti, C and Caggia, F and Capone, G and Barden, N and Blom, JMC and Brunello, N | Excluded | The article was excluded based on title and abstract screening as it did not meet the inclusion and exclusion criteria. |
| Diversity of daily activities is associated with greater hippocampal volume | 2022 | Urban-Wojcik, EJ and Lee, S and Grupe, DW and Quinlan, L and Gresham, L and Hammond, A and Charles, ST and Lachman, ME and Almeida, DM and Davidson, RJ and Schaefer, SM | Excluded | The article was excluded based on title and abstract screening as it did not meet the inclusion and exclusion criteria. |
| Hormonal and Neurological Aspects of Dog Walking for Dog Owners and Pet Dogs | 2021 | Akiyama, J and Ohta, M | Excluded | The article was excluded based on title and abstract screening as it did not meet the inclusion and exclusion criteria. |
| Application of Mass Spectrometry to Characterize Localization and Efficacy of Nanoceria In Vivo | 2014 | Heckman, KL and Erlichman, J and Reed, K and Skeels, M | Excluded | The article was excluded based on title and abstract screening as it did not meet the inclusion and exclusion criteria. |
| Voluntary exercise does not affect stress-induced tachycardia, but improves resistance to cardiac arrhythmias in rats | 2011 | Beig, MI and Callister, R and Saint, DA and Bondarenko, E and Walker, FR and Day, TA and Nalivaiko, E | Excluded | The article was excluded based on title and abstract screening as it did not meet the inclusion and exclusion criteria. |
| Identification of metabolomic changes before and after exercise regimen in stress induced rats | 2017 | Sowndarya, R and Doss, VA | Excluded | The article was excluded based on title and abstract screening as it did not meet the inclusion and exclusion criteria. |
| The gut microbiome as a driver of individual variation in cognition and functional behaviour | 2018 | Davidson, GL and Cooke, AC and Johnson, CN and Quinn, JL | Excluded | The article was excluded based on title and abstract screening as it did not meet the inclusion and exclusion criteria. |
| Fluoxetine reverses the behavioral despair induced by neurogenic stress in mice: role of <i>N</i>-methyl-D-aspartate and opioid receptors | 2016 | Haj-Mirzaian, A and Kordjazy, N and Ostadhadi, S and Amiri, S and Haj-Mirzaian, A and Dehpour, A | Excluded | The article was excluded based on title and abstract screening as it did not meet the inclusion and exclusion criteria. |
| The Effect of Photoluminescence of Bioceramic Irradiation on Middle Cerebral Arterial Occlusion in Rats | 2016 | Zhang, L and Chan, P and Liu, ZM and Hwang, LL and Lin, KC and Chan, WP and Leung, TK and Choy, CS | Excluded | The article was excluded based on title and abstract screening as it did not meet the inclusion and exclusion criteria. |
| Precious but convenient means of prevention and treatment: physiological molecular mechanisms of interaction between exercise and motor factors and Alzheimer's disease | 2023 | Hao, ZK and Liu, KR and Zhou, L and Chen, P | Excluded | The article was excluded based on title and abstract screening as it did not meet the inclusion and exclusion criteria. |
| Lipopolysaccharide exacerbates chronic restraint stress-induced neurobehavioral deficits: Mechanisms by redox imbalance, ASK1-related apoptosis, autophagic dysregulation | 2021 | Kwatra, M and Ahmed, S and Gangipangi, VK and Panda, SR and Gupta, N and Shantanu, PA and Gawali, B and Naidu, VGM | Excluded | The article was excluded based on title and abstract screening as it did not meet the inclusion and exclusion criteria. |
| PHYSICAL EFFORT AFFECTS HEATSTROKE THERMOREGULATORY RESPONSE AND MORTALITY IN RATS | 2015 | Geng, Y and Peng, N and Liu, YN and Li, XG and Li, BL and Peng, LQ and Ma, Q and Su, L | Excluded | The article was excluded based on title and abstract screening as it did not meet the inclusion and exclusion criteria. |
| The anxiolytic etifoxine protects against convulsant and anxiogenic aspects of the alcohol withdrawal syndrome in mice | 2009 | Verleye, M and Heulard, I and Gillardin, JM | Excluded | The article was excluded based on title and abstract screening as it did not meet the inclusion and exclusion criteria. |
| Nutrition, brain function and cognitive performance | 2003 | Lieberman, HR | Excluded | The article was excluded based on title and abstract screening as it did not meet the inclusion and exclusion criteria. |
| Behavioural defences in animals against pathogens and parasites: parallels with the pillars of medicine in humans | 2011 | Hart, BL | Excluded | The article was excluded based on title and abstract screening as it did not meet the inclusion and exclusion criteria. |
| Intracerebroventricularly Injected Streptozotocin Exerts Subtle Effects on the Cognitive Performance of Long-Evans Rats | 2021 | Gáspár, A and Hutka, B and Ernyey, AJ and Tajti, BT and Varga, BT and Zádori, ZS and Gyertyán, I | Excluded | The article was excluded based on title and abstract screening as it did not meet the inclusion and exclusion criteria. |
| Sex and Estrous Cycle Effects on Anxiety- and Depression-Related Phenotypes in a Two-Hit Developmental Stress Model | 2019 | Jaric, I and Rocks, D and Cham, H and Herchek, A and Kundakovic, M | Excluded | The article was excluded based on title and abstract screening as it did not meet the inclusion and exclusion criteria. |
| The blood lactate/pyruvate equilibrium affair | 2022 | Brooks, GA and Osmond, AD and Leija, RG and Curl, CC and Arevalo, JA and Duong, JJ and Horning, MA | Excluded | The article was excluded based on title and abstract screening as it did not meet the inclusion and exclusion criteria. |
| Genome-wide analysis of aging and learning-related genes in the hippocampal dentate gyrus | 2008 | Burger, C and Lopez, MC and Baker, HV and Mandel, RJ and Muzyczka, N | Excluded | The article was excluded based on title and abstract screening as it did not meet the inclusion and exclusion criteria. |
| Effect of long-term caffeine administration on depressive-like behavior in rats exposed to chronic unpredictable stress | 2012 | Pechlivanova, DM and Tchekalarova, JD and Alova, LH and Petkov, VV and Nikolov, RP and Yakimova, KS | Excluded | The article was excluded based on title and abstract screening as it did not meet the inclusion and exclusion criteria. |
| High housing density increases stress hormone- or disease-associated fecal microbiota in male Brandt's voles (<i>Lasiopodomys brandtii</i>) | 2020 | Liu, J and Huang, SL and Li, GL and Zhao, JD and Lu, W and Zhang, ZB | Excluded | The article was excluded based on title and abstract screening as it did not meet the inclusion and exclusion criteria. |
| Intranasal oxytocin administration improves depression-like behaviors in adult rats that experienced neonatal maternal deprivation | 2016 | Ji, HY and Su, WL and Zhou, RC and Feng, J and Lin, Y and Zhang, YM and Wang, XM and Chen, XY and Li, J | Excluded | The article was excluded based on title and abstract screening as it did not meet the inclusion and exclusion criteria. |
| Daily exposure to a touchscreen-paradigm and associated food restriction evokes an increase in adrenocortical and neural activity in mice | 2016 | Mallien, AS and Palme, R and Richetto, J and Muzzillo, C and Richter, SH and Vogt, MA and Inta, D and Riva, MA and Vollmayr, B and Gass, P | Excluded | The article was excluded based on title and abstract screening as it did not meet the inclusion and exclusion criteria. |
| Better stress coping associated with lower tau in amyloid-positive cognitively unimpaired older adults | 2020 | Arenaza-Urquijo, EM and Przybelski, SA and Machulda, MM and Knopman, DS and Lowe, VJ and Mielke, MM and Reddy, AL and Geda, YE and Jack, CR and Petersen, RC and Vemuri, P | Excluded | The article was excluded based on title and abstract screening as it did not meet the inclusion and exclusion criteria. |
| Angiotensin II type 1 receptor blockade by telmisartan prevents stress-induced impairment of memory via HPA axis deactivation and up-regulation of brain-derived neurotrophic factor gene expression | 2016 | Wincewicz, D and Juchniewicz, A and Waszkiewicz, N and Braszko, J | Excluded | The article was excluded based on title and abstract screening as it did not meet the inclusion and exclusion criteria. |
| Treadmill Exercise Prevents Increase of Neuroinflammation Markers Involved in the Dopaminergic Damage of the 6-OHDA Parkinson's Disease Model | 2017 | Real, CC and Garcia, PC and Britto, LRG | Excluded | The article was excluded based on title and abstract screening as it did not meet the inclusion and exclusion criteria. |
| Physical Activity and Spatial Memory Are Minimally Affected by Moderate Growth Restriction in Preterm Piglets | 2020 | Cao, MQ and Brunse, A and Thymann, T and Sangild, PT | Excluded | The article was excluded based on title and abstract screening as it did not meet the inclusion and exclusion criteria. |
| Cognitive dysfunction syndrome: updated behavioral and clinical evaluations as a tool to evaluate the well-being of aging dogs | 2014 | Pineda, S and Olivares, A and Mos, B and Ibañez, M | Excluded | The article was excluded based on title and abstract screening as it did not meet the inclusion and exclusion criteria. |
| Rostral elements of sympatho-motor circuitry: A virally mediated transsynaptic tracing study | 2006 | Kerman, IA and Akil, H and Watson, SJ | Excluded | The article was excluded based on title and abstract screening as it did not meet the inclusion and exclusion criteria. |
| SELECTIVE BRAIN COOLING IN RESTING AND EXERCISING NORWEGIAN REINDEER (RANGIFER-TARANDUS-TARANDUS) | 1993 | KUHNEN, G and MERCER, JB | Excluded | The article was excluded based on title and abstract screening as it did not meet the inclusion and exclusion criteria. |
| VENTILATORY AND METABOLIC RESPONSES TO COLD AND CO2 IN INTACT AND CAROTID BODY-DENERVATED AWAKE RATS | 1993 | GAUTIER, H and BONORA, M and TRINH, HC | Excluded | The article was excluded based on title and abstract screening as it did not meet the inclusion and exclusion criteria. |
| The biology of aging | 2009 | Le Gall, JY and Ardaillou, R | Excluded | The article was excluded based on title and abstract screening as it did not meet the inclusion and exclusion criteria. |
| Antiaging treatments have been legally prescribed for approximately thirty years | 2004 | Ukraintseva, SV and Arbeev, KG and Michalsky, AI and Yashin, AI | Excluded | The article was excluded based on title and abstract screening as it did not meet the inclusion and exclusion criteria. |
| Valence of physical stimuli, not housing conditions, affects behaviour and frontal cortical brain activity in sheep | 2014 | Vögeli, S and Lutz, J and Wolf, M and Wechsler, B and Gygax, L | Excluded | The article was excluded based on title and abstract screening as it did not meet the inclusion and exclusion criteria. |
| Neuroprotective effects of insulin-like growth factor-I (IGF-I) following a penetrating brain injury in rats | 2003 | Kazanis, I and Bozas, E and Philippidis, H and Stylianopoulou, F | Excluded | The article was excluded based on title and abstract screening as it did not meet the inclusion and exclusion criteria. |
| Chronic immobilization stress differentially affects the expression of BDNF mRNA and protein in the mouse hippocampus | 2004 | Adlard, PA and Perreau, VM and Cotman, CW | Excluded | The article was excluded based on title and abstract screening as it did not meet the inclusion and exclusion criteria. |
| The effects of leptin hormone on locomotor activity in Syrian hamsters (<i>Mesocricetus auratus</i>) | 2011 | Gündüz, B and Karakas, A | Excluded | The article was excluded based on title and abstract screening as it did not meet the inclusion and exclusion criteria. |
| CELLULAR-DISTRIBUTION OF IMMUNOREACTIVE SOMATOSTATIN IN THE BASAL FOREBRAIN OF HORSES AND PONIES | 1993 | MELROSE, PA and KNIGGE, KM | Excluded | The article was excluded based on title and abstract screening as it did not meet the inclusion and exclusion criteria. |
| Gnawing behavior of a mouse in a narrow cylinder: A simple system for the study of muscle activity, fatigue, and stress | 2002 | Ayada, K and Tadano, T and Endo, Y | Excluded | The article was excluded based on title and abstract screening as it did not meet the inclusion and exclusion criteria. |
| Inflammation and post-traumatic stress disorder | 2019 | Hori, H and Kim, Y | Excluded | The article was excluded based on title and abstract screening as it did not meet the inclusion and exclusion criteria. |
| Prolonged isolation stress accelerates the onset of Alzheimer's disease-related pathology in 5xFAD mice despite running wheels and environmental enrichment | 2020 | Peterman, JL and White, JD and Calcagno, A and Hagen, C and Quiring, M and Paulhus, K and Gurney, T and Eimerbrink, MJ and Curtis, M and Boehm, GW and Chumley, MJ | Excluded | The article was excluded based on title and abstract screening as it did not meet the inclusion and exclusion criteria. |
| The obesogenic side of Genistein | 2023 | Xiang, J and Mlambo, R and Dube, P and Machona, O and Shaw, I and Seid, Y and He, YJ and Luo, M and Hong, TT and He, BS and Zhou, WH and Tan, SW | Excluded | The article was excluded based on title and abstract screening as it did not meet the inclusion and exclusion criteria. |
| Ambient temperature influences the neural benefits of exercise | 2016 | Maynard, ME and Chung, C and Comer, A and Nelson, K and Tran, J and Werries, N and Barton, EA and Spinetta, M and Leasure, JL | Excluded | The article was excluded based on title and abstract screening as it did not meet the inclusion and exclusion criteria. |
| Temperature and Heart Rate Responses to Exercise following Mild Traumatic Brain Injury | 2013 | Griesbach, GS and Tio, DL and Nair, S and Hovda, DA | Excluded | The article was excluded based on title and abstract screening as it did not meet the inclusion and exclusion criteria. |
| ENRICHED ENVIRONMENT INFLUENCES HORMONAL STATUS AND HIPPOCAMPAL BRAIN DERIVED NEUROTROPHIC FACTOR IN A SEX DEPENDENT MANNER | 2009 | Bakos, J and Hlavacova, N and Rajman, M and Ondicova, K and Koros, C and Kitraki, E and Steinbusch, HWM and Jezova, D | Excluded | The article was excluded based on title and abstract screening as it did not meet the inclusion and exclusion criteria. |
| Effects of Environmental Enrichment on Doublecortin and BDNF Expression along the Dorso-Ventral Axis of the Dentate Gyrus | 2017 | Gualtieri, F and Brégère, C and Laws, GC and Armstrong, EA and Wylie, NJ and Moxham, TT and Guzman, R and Boswell, T and Smulders, TV | Excluded | The article was excluded based on title and abstract screening as it did not meet the inclusion and exclusion criteria. |
| Repeated maternal separation: Alcohol consumption, anxious behavior and corticosterone were reversed by a non-pharmacological treatment | 2019 | Odeon, MM and Acosta, GB | Excluded | The article was excluded based on title and abstract screening as it did not meet the inclusion and exclusion criteria. |
| Rectal temperature measurement results in artifactual evidence of selective brain cooling | 2001 | Maloney, SK and Fuller, A and Mitchell, G and Mitchell, D | Excluded | The article was excluded based on title and abstract screening as it did not meet the inclusion and exclusion criteria. |
| Daphnetin alleviates experimental autoimmune encephalomyelitis by suppressing Th1 and Th17 cells and upregulating Th2 and regulatory T cells | 2022 | Soltanmohammadi, A and Tavaf, MJ and Zargarani, S and Yazdanpanah, E and Sadighi-Moghaddam, B and Yousefi, B and Sameni, HR and Haghmorad, D | Excluded | The article was excluded based on title and abstract screening as it did not meet the inclusion and exclusion criteria. |
| Dealing With Stress in Cats: What Is New About the Olfactory Strategy? | 2022 | Zhang, LN and Bian, ZW and Liu, QS and Deng, BC | Excluded | The article was excluded based on title and abstract screening as it did not meet the inclusion and exclusion criteria. |
| Altered gene expression of the innate immune, neuroendocrine, and nuclear factor-kappa B (NF-κB) systems is associated with posttraumatic stress disorder in military personnel | 2016 | Guardado, P and Olivera, A and Rusch, HL and Roy, M and Martin, C and Lejbman, N and Lee, H and Gill, JM | Excluded | The article was excluded based on title and abstract screening as it did not meet the inclusion and exclusion criteria. |
| Interactions of plasma osmolality with arterial and central venous pressures in control of sympathetic activity and heart rate in humans | 2005 | Charkoudian, N and Eisenach, JH and Joyner, MJ and Roberts, SK and Wick, DE | Excluded | The article was excluded based on title and abstract screening as it did not meet the inclusion and exclusion criteria. |
| Activity-based anorexia activates CRF immunoreactive neurons in female rats | 2018 | Scharner, S and Friedrich, T and Goebel-Stengel, M and Kobelt, P and Rose, M and Stengel, A | Excluded | The article was excluded based on title and abstract screening as it did not meet the inclusion and exclusion criteria. |
| NEUROPROTECTIVE EFFECTS OF CEREBROLYSIN, A COMBINATION OF DIFFERENT ACTIVE FRAGMENTS OF NEUROTROPHIC FACTORS AND PEPTIDES ON THE WHOLE BODY HYPERTHERMIA-INDUCED NEUROTOXICITY: MODULATORY ROLES OF CO-MORBIDITY FACTORS AND NANOPARTICLE INTOXICATION | 2012 | Sharma, HS and Sharma, A and Mössler, H and Muresanu, DF | Excluded | The article was excluded based on title and abstract screening as it did not meet the inclusion and exclusion criteria. |
| A Growing Dilemma: Antenatal Corticosteroids and Long-Term Consequences | 2022 | Asztalos, EV and Murphy, KE and Matthews, SG | Excluded | The article was excluded based on title and abstract screening as it did not meet the inclusion and exclusion criteria. |
| Performance of the intracerebroventricularly injected streptozotocin Alzheimer's disease model in a translationally relevant, aged and experienced rat population | 2022 | Gáspár, A and Hutka, B and Ernyey, AJ and Tajti, BT and Varga, BT and Zádori, ZS and Gyertyán, I | Excluded | The article was excluded based on title and abstract screening as it did not meet the inclusion and exclusion criteria. |
| Effects of environmental enrichment on anxiety responses, spatial memory and cytochrome c oxidase activity in adult rats | 2013 | Sampedro-Piquero, P and Zancada-Menendez, C and Begega, A and Rubio, S and Arias, JL | Excluded | The article was excluded based on title and abstract screening as it did not meet the inclusion and exclusion criteria. |
| Moderate treadmill exercise ameliorates amyloid-β-induced learning and memory impairment, possibly via increasing AMPK activity and up-regulation of the PGC-1α/FNDC5/BDNF pathway | 2018 | Azimi, M and Gharakhanlou, R and Naghdi, N and Khodadadi, D and Heysieattalab, S | Excluded | The article was excluded based on title and abstract screening as it did not meet the inclusion and exclusion criteria. |
| Deficit in BDNF does not increase vulnerability to stress but dampens antidepressant-like effects in the unpredictable chronic mild stress | 2009 | Ibarguen-Vargas, Y and Surget, A and Vourc'h, P and Leman, S and Andres, CR and Gardier, AM and Belzung, C | Excluded | The article was excluded based on title and abstract screening as it did not meet the inclusion and exclusion criteria. |
| Effects of stress on catecholamine stores in central and peripheral tissues of long-term socially isolated rats | 2006 | Dronjak, S and Gavrilovic, L | Excluded | The article was excluded based on title and abstract screening as it did not meet the inclusion and exclusion criteria. |
| Effects of prenatal stress and exercise on dentate granule cells maturation and spatial memory in adolescent mice | 2010 | Bustamante, C and Bilbao, P and Contreras, W and Martínez, M and Mendoza, A and Reyes, A and Pascual, R | Excluded | The article was excluded based on title and abstract screening as it did not meet the inclusion and exclusion criteria. |
| Differential expression of c-<i>fos</i> mRNA within neurocircuits of male hamsters exposed to acute or chronic defeat | 1999 | Kollack-Walker, S and Don, C and Watson, SJ and Akil, H | Excluded | The article was excluded based on title and abstract screening as it did not meet the inclusion and exclusion criteria. |
| Exercise pretraining protects against cerebral ischaemia induced by heat stroke in rats | 2007 | Chen, YW and Chen, SH and Chou, W and Lo, YM and Hung, CH and Lin, MT | Excluded | The article was excluded based on title and abstract screening as it did not meet the inclusion and exclusion criteria. |
| Effects of childhood poverty and chronic stress on emotion regulatory brain function in adulthood | 2013 | Kim, P and Evans, GW and Angstadt, M and Ho, SS and Sripada, CS and Swain, JE and Liberzon, I and Phan, KL | Excluded | The article was excluded based on title and abstract screening as it did not meet the inclusion and exclusion criteria. |
| Structural and functional effects of social isolation on the hippocampus of rats with traumatic brain injury | 2015 | Khodaie, B and Lotfinia, AA and Ahmadi, M and Lotfinia, M and Jafarian, M and Karimzadeh, F and Coulon, P and Gorji, A | Excluded | The article was excluded based on title and abstract screening as it did not meet the inclusion and exclusion criteria. |
| Voluntary exercise and tail shock have differential effects on amphetamine-induced dopaminergic toxicity in adult BALB/c mice | 2006 | Carlson, KM and Wagner, GC | Excluded | The article was excluded based on title and abstract screening as it did not meet the inclusion and exclusion criteria. |
| Brain and abdominal temperatures at fatigue in rats exercising in the heat | 1998 | Fuller, A and Carter, RN and Mitchell, D | Excluded | The article was excluded based on title and abstract screening as it did not meet the inclusion and exclusion criteria. |
| Moderate developmental undernutrition: Impact on growth and cognitive function in youth and old age | 2010 | Gilbert, ME and MacPhail, R and Baldwin, J and Moser, VC and Chernoff, N | Excluded | The article was excluded based on title and abstract screening as it did not meet the inclusion and exclusion criteria. |
| Efficiency of newly formulated functional instant soup mixtures as dietary supplements for elderly | 2020 | Mohamed, RS and Abozed, SS and El-Damhougy, S and Salama, MF and Hussein, MM | Excluded | The article was excluded based on title and abstract screening as it did not meet the inclusion and exclusion criteria. |
| Novel stress increases hypothalamic-pituitary-adrenal activity in mice with a raised bite | 2016 | Miyake, H and Mori, D and Katayama, T and Fujiwara, S and Sato, Y and Azuma, K and Kubo, K | Excluded | The article was excluded based on title and abstract screening as it did not meet the inclusion and exclusion criteria. |
| Deciphering the Neural Control of Sympathetic Nerve Activity: Status Report and Directions for Future Research | 2017 | Barman, SM and Yates, BJ | Excluded | The article was excluded based on title and abstract screening as it did not meet the inclusion and exclusion criteria. |
| Hyperthermia increases interleukin-6 in mouse skeletal muscle | 2012 | Welc, SS and Phillips, NA and Oca-Cossio, J and Wallet, SM and Chen, DL and Clanton, TL | Excluded | The article was excluded based on title and abstract screening as it did not meet the inclusion and exclusion criteria. |
| Chronic pain and adult hippocampal neurogenesis: translational implications from preclinical studies | 2017 | Grilli, M | Excluded | The article was excluded based on title and abstract screening as it did not meet the inclusion and exclusion criteria. |
| Exercise modulates neuronal activation in the micturition circuit of chronically stressed rats: A multidisciplinary approach to the study of urologic chronic pelvic pain syndrome (MAPP) research network study | 2020 | Holschneider, DP and Wang, Z and Guo, YM and Sanford, MT and Yeh, J and Mao, JJ and Zhang, R and Rodriguez, LV | Excluded | The article was excluded based on title and abstract screening as it did not meet the inclusion and exclusion criteria. |
| Effect of oleuropein on cognitive deficits and changes in hippocampal brain-derived neurotrophic factor and cytokine expression in a rat model of post-traumatic stress disorder | 2018 | Lee, B and Shim, I and Lee, H and Hahm, DH | Excluded | The article was excluded based on title and abstract screening as it did not meet the inclusion and exclusion criteria. |
| Developmental consequences of antenatal dexamethasone treatment in nonhuman primates | 2005 | Coe, CL and Lubach, GR | Excluded | The article was excluded based on title and abstract screening as it did not meet the inclusion and exclusion criteria. |
| Hypothalamic mechanisms coordinating cardiorespiratory function during exercise and defensive behaviour | 2008 | Dampney, RAL and Horiuchi, J and McDowall, LM | Excluded | The article was excluded based on title and abstract screening as it did not meet the inclusion and exclusion criteria. |
| Sex differences in resilience: Experiential factors and their mechanisms | 2020 | Fallon, IP and Tanner, MK and Greenwood, BN and Baratta, M | Excluded | The article was excluded based on title and abstract screening as it did not meet the inclusion and exclusion criteria. |
| Forced, not voluntary, exercise effectively induces neuroprotection in stroke | 2008 | Hayes, K and Sprague, S and Guo, M and Davis, W and Friedman, A and Kumar, A and Jimenez, DF and Ding, YC | Excluded | The article was excluded based on title and abstract screening as it did not meet the inclusion and exclusion criteria. |
| Heterozygous mice deficient in <i>Atp1a3</i> exhibit motor deficits by chronic restraint stress | 2014 | Sugimoto, H and Ikeda, K and Kawakami, K | Excluded | The article was excluded based on title and abstract screening as it did not meet the inclusion and exclusion criteria. |
| Exercise partly reverses the effect of maternal separation on hippocampal proteins in 6-hydroxydopamine-lesioned rat brain | 2013 | Dimatelis, JJ and Hendricks, S and Hsieh, J and Vlok, NM and Bugarith, K and Daniels, WMU and Russell, VA | Excluded | The article was excluded based on title and abstract screening as it did not meet the inclusion and exclusion criteria. |
| The Molecular Effects of Environmental Enrichment on Alzheimer's Disease | 2022 | Liew, AKY and Teo, CH and Soga, T | Excluded | The article was excluded based on title and abstract screening as it did not meet the inclusion and exclusion criteria. |
| Prenatal Lipopolysaccharide Exposure Affects Maternal Behavior and Male Offspring Sexual Behavior in Adulthood | 2010 | Bernardi, MM and Kirsten, TB and Matsuoka, SM and Teodorov, E and Habr, SF and Penteado, SHWN and Palermo-Neto, J | Excluded | The article was excluded based on title and abstract screening as it did not meet the inclusion and exclusion criteria. |
| Unravelling the health effects of fasting: a long road from obesity treatment to healthy life span increase and improved cognition | 2020 | de Toledo, FW and Grundler, F and Sirtori, CR and Ruscica, M | Excluded | The article was excluded based on title and abstract screening as it did not meet the inclusion and exclusion criteria. |
| Epigenetic and gene expression changes in the adolescent brain: What have we learned from animal models? | 2016 | Mychasiuk, R and Metz, GAS | Excluded | The article was excluded based on title and abstract screening as it did not meet the inclusion and exclusion criteria. |
| Sleep in the United States Military | 2020 | Good, CH and Brager, AJ and Capaldi, VF and Mysliwiec, V | Excluded | The article was excluded based on title and abstract screening as it did not meet the inclusion and exclusion criteria. |
| Binge eating in pre-clinical models | 2015 | Rospond, B and Szpigiel, J and Sadakierska-Chudy, A and Filip, M | Excluded | The article was excluded based on title and abstract screening as it did not meet the inclusion and exclusion criteria. |
| Hypomagnetic Field Alters Circadian Rhythm and Increases Algesia in Adult Male Mice | 2015 | Mo, WC and Fu, JP and Ding, HM and Liu, Y and Hua, Q and He, RQ | Excluded | The article was excluded based on title and abstract screening as it did not meet the inclusion and exclusion criteria. |
| THE EFFECTS OF EXERCISE ON THE ORAL CONSUMPTION OF MORPHINE AND METHADONE IN RATS | 1994 | MCLACHLAN, CD and HAY, M and COLEMAN, GJ | Excluded | The article was excluded based on title and abstract screening as it did not meet the inclusion and exclusion criteria. |
| Pig Skin Gelatin Hydrolysates Attenuate Acetylcholine Esterase Activity and Scopolamine-induced Impairment of Memory and Learning Ability of Mice | 2020 | Kim, D and Kim, YHB and Ham, JS and Lee, SK and Jang, A | Excluded | The article was excluded based on title and abstract screening as it did not meet the inclusion and exclusion criteria. |
| Role for gastrointestinal endotoxins in enhancement of heat tolerance by physical fitness | 1998 | Sakurada, S and Hales, JRS | Excluded | The article was excluded based on title and abstract screening as it did not meet the inclusion and exclusion criteria. |
| Cardiac and systemic rejuvenation after cardiosphere-derived cell therapy in senescent rats | 2017 | Grigorian-Shamagian, L and Liu, WX and Fereydooni, S and Middleton, RC and Valle, J and Cho, JH and Marbán, E | Excluded | The article was excluded based on title and abstract screening as it did not meet the inclusion and exclusion criteria. |
| Absence of selective brain cooling in free-ranging zebras in their natural habitat | 2000 | Fuller, A and Maloney, SK and Kamerman, PR and Mitchell, G and Mitchell, D | Excluded | The article was excluded based on title and abstract screening as it did not meet the inclusion and exclusion criteria. |
| Sex-dependent and -independent regulation of thyrotropin-releasing hormone expression in the hypothalamic dorsomedial nucleus by negative energy balance, exercise, and chronic stress br | 2022 | Vargas, Y and de Oca, MPM and Sánchez-Jaramillo, E and Jaimes-Hoy, L and Sánchez-Islas, E and Uribe, RM and Joseph-Bravo, P and Charli, JL | Excluded | The article was excluded based on title and abstract screening as it did not meet the inclusion and exclusion criteria. |
| Effect of <i>Rhodiola rosea</i> Rhizome and <i>Punica granatum</i> Fruit Peel on the Metabolic Processes and Physiological Activity of Rats Fed with Excessively Fat Diet | 2023 | Lieshchova, M and Brygadyrenko, V | Excluded | The article was excluded based on title and abstract screening as it did not meet the inclusion and exclusion criteria. |
| Changes in organ perfusion and weight ratios in post-simulated microgravity recovery | 2002 | Deever, DB and Young, RS and Wang, SJ and Bradshaw, BA and Miles, J and Pettis, CR and Witten, ML | Excluded | The article was excluded based on title and abstract screening as it did not meet the inclusion and exclusion criteria. |
| Beneficial effects of exercise and its molecular mechanisms on depression in rats | 2006 | Zheng, H and Liu, YY and Li, W and Yang, B and Chen, DB and Wang, XJ and Jiang, Z and Wang, HX and Wang, ZG and Cornelisson, G and Halberg, F | Excluded | The article was excluded based on title and abstract screening as it did not meet the inclusion and exclusion criteria. |
| THE EFFECTS OF PRENATAL EXPOSURE TO HYPOXIA ON THE BEHAVIOR OF RATS DURING THEIR LIFE-SPAN | 1994 | JANICKE, B and COPER, H | Excluded | The article was excluded based on title and abstract screening as it did not meet the inclusion and exclusion criteria. |
| Age-Related Cognitive Impairment as a Sign of Geriatric Neurocardiovascular Interactions: May Polyphenols Play a Protective Role? | 2015 | Jagla, F and Pechanova, O | Excluded | The article was excluded based on title and abstract screening as it did not meet the inclusion and exclusion criteria. |
| Environmental enrichment rescues the effects of early life inflammation on markers of synaptic transmission and plasticity | 2016 | Kentner, AC and Khoury, A and Queiroz, EL and MacRae, M | Excluded | The article was excluded based on title and abstract screening as it did not meet the inclusion and exclusion criteria. |
| Long-Term Social Isolation Reduces Expression of the BDNF Precursor and Prolyl Endopeptidase in the Rat Brain | 2021 | Shirenova, SD and Khlebnikova, NN and Krupina, NA | Excluded | The article was excluded based on title and abstract screening as it did not meet the inclusion and exclusion criteria. |
| Differential effects of social stress on central serotonergic activity and emotional reactivity in Lewis and spontaneously hypertensive rats | 1998 | Berton, O and Aguerre, S and Sarrieau, A and Mormede, P and Chaouloff, F | Excluded | The article was excluded based on title and abstract screening as it did not meet the inclusion and exclusion criteria. |
| MATERNAL DEPRIVATION OF RAT PUPS REDUCES BODY WEIGHT AND ALTERS BEHAVIOR IN ADULTHOOD IN A GENDER-SPECIFIC MANNER | 2015 | Dordevic, J and Mitic, M and Lukic, I and Adzic, M | Excluded | The article was excluded based on title and abstract screening as it did not meet the inclusion and exclusion criteria. |
| Magnetic brain stimulation using iron oxide nanoparticle-mediated selective treatment of the left prelimbic cortex as a novel strategy to rapidly improve depressive-like symptoms in mice | 2020 | Lu, QB and Sun, JF and Yang, QY and Cai, WW and Xia, MQ and Wu, FF and Gu, N and Zhang, ZJ | Excluded | The article was excluded based on title and abstract screening as it did not meet the inclusion and exclusion criteria. |
| Neurobiological Effects of Combined Exposure to Antiorthostatic Suspension, Quasi-Chronic Gamma Irradiation, and Heavy Ion Irradiation in Rats | 2023 | Perevezentsev, AA and Lebedeva-Georgievskaya, KB and Kuznetsova, OS and Kudrin, VS and Pikalov, VA and Shtemberg, AS | Excluded | The article was excluded based on title and abstract screening as it did not meet the inclusion and exclusion criteria. |
| Effects of chronic fluoxetine treatment on anxiety- and depressive-like behaviors in adolescent rodents - systematic review and meta-analysis | 2022 | Kryst, J and Majcher-Maslanka, I and Chocyk, A | Excluded | The article was excluded based on title and abstract screening as it did not meet the inclusion and exclusion criteria. |
| Cross-Generational Impact of Epigenetic Male Influence on Physical Activity in Rat | 2022 | Sudakov, SK and Bogdanova, NG and Nazarova, GA | Excluded | The article was excluded based on title and abstract screening as it did not meet the inclusion and exclusion criteria. |
| Complex Environmental Rearing Enhances Social Salience and Affects Hippocampal Corticotropin Releasing Hormone Receptor Expression in a Sex-Specific Manner | 2018 | Kentner, AC and Lima, E and Migliore, MM and Shin, J and Scalia, S | Excluded | The article was excluded based on title and abstract screening as it did not meet the inclusion and exclusion criteria. |
| Monoamine reuptake inhibition and mood-enhancing potential of a specified oregano extract | 2011 | Mechan, AO and Fowler, A and Seifert, N and Rieger, H and Wöhrle, T and Etheve, S and Wyss, A and Schüler, G and Colletto, B and Kilpert, C and Aston, J and Elliott, JM and Goralczyk, R and Mohajeri, MH | Excluded | The article was excluded based on title and abstract screening as it did not meet the inclusion and exclusion criteria. |
| NEUROSCIENCE FOREFRONT REVIEW NEUROGENESIS ALONG THE SEPTO-TEMPORAL AXIS OF THE HIPPOCAMPUS: ARE DEPRESSION AND OF ANTIDEPRESSANTS REGION-SPECIFIC? | 2013 | Tanti, A and Belzung, C | Excluded | The article was excluded based on title and abstract screening as it did not meet the inclusion and exclusion criteria. |
| An overview of SSR149415, a selective nonpeptide vasopressin V<sub>1b</sub> receptor antagonist for the treatment of stress-related disorders | 2005 | Serradeil-Le Gal, C and Wagnon, J and Tonnerre, B and Roux, R and Garcia, G and Griebel, G and Aulombard, A | Excluded | The article was excluded based on title and abstract screening as it did not meet the inclusion and exclusion criteria. |
| Adolescent nonpharmacological interventions for early-life stress and their mechanisms | 2023 | Li, Y and Shi, DD and Wang, Z | Excluded | The article was excluded based on title and abstract screening as it did not meet the inclusion and exclusion criteria. |
| Potential role of creatine as an anticonvulsant agent: evidence from preclinical studies | 2023 | Alraddadi, EA and Khojah, AM and Alamri, FF and Kecheck, HK and Altaf, WF and Khouqeer, Y | Excluded | The article was excluded based on title and abstract screening as it did not meet the inclusion and exclusion criteria. |
| Exposure to novelty and forced swimming evoke stressor-dependent changes in extracellular GABA in the rat hippocampus | 2007 | de Groote, L and Linthorst, ACE | Excluded | The article was excluded based on title and abstract screening as it did not meet the inclusion and exclusion criteria. |
| HOUSING ENVIRONMENT MODULATES PHYSIOLOGICAL AND BEHAVIORAL RESPONSES TO ANXIOGENIC STIMULI IN TRAIT ANXIETY MALE RATS | 2014 | Ravenelle, R and Santolucito, HB and Byrnes, EM and Byrnes, JJ and Donaldson, ST | Excluded | The article was excluded based on title and abstract screening as it did not meet the inclusion and exclusion criteria. |
| Predicting susceptibility and resilience in an animal model of post-traumatic stress disorder (PTSD) | 2020 | Colucci, P and Marchetta, E and Mancini, GF and Alva, P and Chiarotti, F and Hasan, MT and Campolongo, P | Excluded | The article was excluded based on title and abstract screening as it did not meet the inclusion and exclusion criteria. |
| Molecular mechanisms of experience-dependent structural and functional plasticity in the brain | 2017 | Kondo, M | Excluded | The article was excluded based on title and abstract screening as it did not meet the inclusion and exclusion criteria. |
| Effect of exercise on synaptophysin and calcium/calmodulin-dependent protein kinase levels in prefrontal cortex and hippocampus of a rat model of developmental stress | 2009 | Hescham, S and Grace, L and Kellaway, LA and Bugarith, K and Russell, VA | Excluded | The article was excluded based on title and abstract screening as it did not meet the inclusion and exclusion criteria. |
| Cognitive enrichment to increase fish welfare in aquaculture: A review | 2023 | Kleiber, A and Stomp, M and Rouby, M and Ferreira, VHB and Bégout, ML and Benhaïm, D and Labbé, L and Tocqueville, A and Levadoux, M and Calandreau, L and Guesdon, V and Colson, V | Excluded | The article was excluded based on title and abstract screening as it did not meet the inclusion and exclusion criteria. |
| The Gut-Brain Axis and the Microbiome in Anxiety Disorders, Post-Traumatic Stress Disorder and Obsessive-Compulsive Disorder | 2024 | Mackay, M and Yang, BH and Dursun, SM and Baker, GB | Excluded | The article was excluded based on title and abstract screening as it did not meet the inclusion and exclusion criteria. |
| Neuropeptide W increases mean arterial pressure as a result of behavioral arousal | 2013 | Pate, AT and Yosten, GLC and Samson, WK | Excluded | The article was excluded based on title and abstract screening as it did not meet the inclusion and exclusion criteria. |
| Environmental-Enrichment-Related Variations in Behavioral, Biochemical, and Physiologic Responses of Sprague-Dawley and Long Evans Rats | 2010 | Konkle, ATM and Kentner, AC and Baker, SL and Stewart, A and Bielajew, C | Excluded | The article was excluded based on title and abstract screening as it did not meet the inclusion and exclusion criteria. |
| High fat diet induces brain insulin resistance and cognitive impairment in mice | 2017 | Kothari, V and Luo, YW and Tornabene, T and O'Neill, AM and Greene, MW and Geetha, T and Babu, JR | Excluded | The article was excluded based on title and abstract screening as it did not meet the inclusion and exclusion criteria. |
| Shaping Adult Phenotypes Through Early Life Environments | 2009 | Weaver, ICG | Excluded | The article was excluded based on title and abstract screening as it did not meet the inclusion and exclusion criteria. |
| BOLD fMRI in awake prairie voles: A platform for translational social and affective neuroscience | 2016 | Yee, JR and Kenkel, WM and Kulkarni, P and Moore, K and Perkeybile, AM and Toddes, S and Amacker, JA and Carter, CS and Ferris, CF | Excluded | The article was excluded based on title and abstract screening as it did not meet the inclusion and exclusion criteria. |
| ON THE FUNCTIONS OF MONOAMINE-OXIDASE, THE EMOTIONS, AND ADAPTATION TO STRESS | 1993 | RICHARDSON, JS | Excluded | The article was excluded based on title and abstract screening as it did not meet the inclusion and exclusion criteria. |
| Promoting Successful Cognitive Aging: A Ten-Year Update | 2021 | Krivanek, TJ and Gale, SA and McFeeley, BM and Nicastri, CM and Daffner, KR | Excluded | The article was excluded based on title and abstract screening as it did not meet the inclusion and exclusion criteria. |
| The gut microbiota and nervous system: Age-defined and age-defying | 2021 | Madison, AA and Kiecolt-Glaser, JK | Excluded | The article was excluded based on title and abstract screening as it did not meet the inclusion and exclusion criteria. |
| Two cholinesterase inhibitors trigger dissimilar effects on behavior and body weight in C57BL/6 mice: The case of chlorpyrifos and rivastigmine | 2017 | Basaure, P and Peris-Sampedro, F and Cabré, M and Reverte, I and Colomina, MT | Excluded | The article was excluded based on title and abstract screening as it did not meet the inclusion and exclusion criteria. |
| A virtual rat for simulating environmental and exertional heat stress | 2014 | Rakesh, V and Stallings, JD and Reifman, J | Excluded | The article was excluded based on title and abstract screening as it did not meet the inclusion and exclusion criteria. |
| Can feed additives be used to promote positive behaviour in laying hens? A review | 2022 | Makinde, TO and Adewole, DI | Excluded | The article was excluded based on title and abstract screening as it did not meet the inclusion and exclusion criteria. |
| Bruton's tyrosine kinase drives neuroinflammation and anxiogenic behavior in mouse models of stress | 2021 | Ghosh, S and Mohammed, Z and Singh, I | Excluded | The article was excluded based on title and abstract screening as it did not meet the inclusion and exclusion criteria. |
| Anoxia induces thermotolerance in the locust flight system | 2002 | Wu, BS and Lee, JK and Thompson, KM and Walker, VK and Moyes, CD and Robertson, RM | Excluded | The article was excluded based on title and abstract screening as it did not meet the inclusion and exclusion criteria. |
| Treadmill Exercise Reverses Depression Model-Induced Alteration of Dendritic Spines in the Brain Areas of Mood Circuit | 2019 | Zhuang, PC and Tan, ZN and Jia, ZY and Wang, BJ and Grady, JJ and Ma, XM | Excluded | The article was excluded based on title and abstract screening as it did not meet the inclusion and exclusion criteria. |
| Voluntary exercise reduces plasma cortisol levels and improves transitory memory impairment in young and aged <i>Octodon degus</i> | 2019 | Estrada, C and Cuenca, L and Cano-Fernandez, L and Gil-Martinez, AL and Sanchez-Rodrigo, C and González-Cuello, AM and Fernandez-Villalba, E and Herrero, MT | Excluded | The article was excluded based on title and abstract screening as it did not meet the inclusion and exclusion criteria. |
| Social stress adapts signaling pathways involved in stimulation of the hypothalamic-pituitary-adrenal axis | 1999 | Bugajski, J | Excluded | The article was excluded based on title and abstract screening as it did not meet the inclusion and exclusion criteria. |
| Anxiolytic- and antidepressant-like effects of the non-peptide vasopressin V<sub>1b</sub> receptor antagonist, SSR149415, suggest an innovative approach for the treatment of stress-related disorders | 2002 | Griebel, G and Simiand, J and Gal, CSL and Wagnon, J and Pascal, M and Scatton, B and Maffrand, JP and Soubrié, P | Excluded | The article was excluded based on title and abstract screening as it did not meet the inclusion and exclusion criteria. |
| Blood pressure reactivity to emotional stress is reduced in AT<sub>1A</sub>-receptor knockout mice on normal, but not high salt intake | 2009 | Chen, DA and La Greca, L and Head, GA and Walther, T and Mayorov, DN | Excluded | The article was excluded based on title and abstract screening as it did not meet the inclusion and exclusion criteria. |
| TRPV1 blockers as potential new treatments for psychiatric disorders | 2022 | Iglesias, LP and Aguiar, DC and Moreira, FA | Excluded | The article was excluded based on title and abstract screening as it did not meet the inclusion and exclusion criteria. |
| Hippocampal Neurogenesis and Dendritic Plasticity Support Running-Improved Spatial Learning and Depression-Like Behaviour in Stressed Rats | 2011 | Yau, SY and Lau, BWM and Tong, JB and Wong, R and Ching, YP and Qiu, G and Tang, SW and Lee, TMC and So, KF | Excluded | The article was excluded based on title and abstract screening as it did not meet the inclusion and exclusion criteria. |
| Neuroprotective role of a monoterpene (thymol) on diazepam induced withdrawal symptoms in rats | 2021 | Saleem, S and Naqvi, F and Batool, A and Naqvi, SH and Naqvi, F and Batool, Z and Tabassum, S and Haider, S | Excluded | The article was excluded based on title and abstract screening as it did not meet the inclusion and exclusion criteria. |
| Early deprivation leads to altered behavioural, autonomic and endocrine responses to environmental challenge in adult Fischer rats | 2006 | Rüedi-Bettschen, D and Zhang, WN and Russig, H and Ferger, B and Weston, A and Pedersen, EM and Feldon, J and Pryce, CR | Excluded | The article was excluded based on title and abstract screening as it did not meet the inclusion and exclusion criteria. |
| Establishment of In Vivo Brain Imaging Method in Conscious Mice | 2010 | Mizuma, H and Shukuri, M and Hayashi, T and Watanabe, Y and Onoe, H | Excluded | The article was excluded based on title and abstract screening as it did not meet the inclusion and exclusion criteria. |
| Voluntary exercise alters GABA<sub>A</sub> receptor subunit and glutamic acid decarboxylase-67 gene expression in the rat forebrain | 2010 | Hill, LE and Droste, SK and Nutt, DJ and Linthorst, ACE and Reul, JMHM | Excluded | The article was excluded based on title and abstract screening as it did not meet the inclusion and exclusion criteria. |
| Hyperresponsiveness of hypothalamic-pituitary-adrenal axis to combined dexamethasone/corticotropin-releasing hormone challenge in female borderline personality disorder subjects with a history of sustained childhood abuse | 2002 | Rinne, T and de Kloet, ER and Wouters, L and Goekoop, JG and DeRijk, RH and van den Brink, W | Excluded | The article was excluded based on title and abstract screening as it did not meet the inclusion and exclusion criteria. |
| The BDNF Val66Met Polymorphism Does Not Increase Susceptibility to Activity-Based Anorexia in Rats | 2022 | Pietrucci, CL and Milton, LK and Greaves, E and Stefanidis, A and van den Buuse, M and Oldfield, BJ and Foldi, CJ | Excluded | The article was excluded based on title and abstract screening as it did not meet the inclusion and exclusion criteria. |
| Neonatal stress disrupts the glymphatic system development and increases the susceptibility to Parkinson&apos;s disease in later life | 2024 | Song, J and Li, ZH and Xue, XY and Meng, JC and Zhu, WX and Hu, SF and Xu, GY and Wang, LH | Excluded | The article was excluded based on title and abstract screening as it did not meet the inclusion and exclusion criteria. |
| Long-term effects of early-life environmental manipulations in rodents and primates:: Potential animal models in depression research | 2005 | Pryce, CR and Rüedi-Bettschen, D and Dettling, AC and Weston, A and Russig, H and Ferger, B and Feldon, J | Excluded | The article was excluded based on title and abstract screening as it did not meet the inclusion and exclusion criteria. |
| The Ultradian and Circadian Rhythms of Free Corticosterone in the Brain are Not Affected by Gender: An <i>In Vivo</i> Microdialysis Study in Wistar Rats | 2009 | Droste, SK and de Groote, L and Lightman, SL and Reul, JMHM and Linthorst, ACE | Excluded | The article was excluded based on title and abstract screening as it did not meet the inclusion and exclusion criteria. |
| Increased depression-related behavior during the postpartum period in inbred BALB/c and C57BL/6 strains | 2019 | Shoji, H and Miyakawa, T | Excluded | The article was excluded based on title and abstract screening as it did not meet the inclusion and exclusion criteria. |
| Gender differences in the effect of prenatal alcohol exposure on the hypothalamic-pituitary-adrenal axis response to immune signals | 1996 | Lee, S and Rivier, C | Excluded | The article was excluded based on title and abstract screening as it did not meet the inclusion and exclusion criteria. |
| Attitudes to and beliefs about animal assisted therapy for children with disabilities | 2017 | Yap, E and Scheinberg, A and Williams, K | Excluded | The article was excluded based on title and abstract screening as it did not meet the inclusion and exclusion criteria. |
| Positive effects of running exercise on astrocytes in the medial prefrontal cortex in an animal model of depression | 2022 | Huang, DJ and Xiao, Q and Tang, J and Liang, X and Wang, J and Hu, ML and Jiang, YH and Liu, L and Qin, L and Zhou, M and Li, Y and Zhu, PL and Deng, YH and Li, J and Zhou, CN and Luo, YM and Tang, Y | Excluded | The article was excluded based on title and abstract screening as it did not meet the inclusion and exclusion criteria. |
| Mechanisms of fatigue during exercise-heat stress | 2002 | Strachan, A and Maughan, R | Excluded | The article was excluded based on title and abstract screening as it did not meet the inclusion and exclusion criteria. |
| Changes in ornithine decarboxylase activity and putrescine concentrations after spinal cord compression injury in the rat | 1999 | Mautes, AEM and Paschen, W and Röhn, G and Nacimiento, AC | Excluded | The article was excluded based on title and abstract screening as it did not meet the inclusion and exclusion criteria. |
| The complex interplay of genetics, epigenetics, and environment in the predisposition to alcohol dependence | 2011 | Díaz-Anzaldúa, A and Díaz-Martínez, A and Díaz-Martínez, LR | Excluded | The article was excluded based on title and abstract screening as it did not meet the inclusion and exclusion criteria. |
| Understanding Addiction Using Animal Models | 2019 | Kuhn, BN and Kalivas, PW and Bobadilla, AC | Excluded | The article was excluded based on title and abstract screening as it did not meet the inclusion and exclusion criteria. |
| LIMITATIONS ON ARTERIOVENOUS COOLING OF THE BLOOD-SUPPLY TO THE HUMAN BRAIN | 1994 | NUNNELEY, SA and NELSON, DA | Excluded | The article was excluded based on title and abstract screening as it did not meet the inclusion and exclusion criteria. |
| Chronic REM Sleep Restriction in Juvenile Male Rats Induces Anxiety-Like Behavior and Alters Monoamine Systems in the Amygdala and Hippocampus | 2018 | Rocha-Lopes, JD and Machado, RB and Suchecki, D | Excluded | The article was excluded based on title and abstract screening as it did not meet the inclusion and exclusion criteria. |
| PROTECTIVE EFFECTS OF VORTIOXETINE IN PREDATOR SCENT STRESS MODEL OF POST-TRAUMATIC STRESS DISORDER IN RATS: ROLE ON NEUROPLASTICITY AND APOPTOSIS | 2019 | Ozbeyli, D and Aykac, A and Alaca, N and Hazar-Yavuz, AN and Ozkan, N and Sener, G | Excluded | The article was excluded based on title and abstract screening as it did not meet the inclusion and exclusion criteria. |
| Brain hyperthermia induced by MDMA ('ecstasy'): modulation by environmental conditions | 2004 | Brown, PL and Kiyatkin, EA | Excluded | The article was excluded based on title and abstract screening as it did not meet the inclusion and exclusion criteria. |
| Regulation of circulating chromogranin B levels in heart failure | 2018 | Heidrich, FM and Melz, C and Buechau, MS and Pfluecke, C and Quick, S and Speiser, U and Poitz, DM and Augstein, A and Ruf, T and Wässnig, NK and Youssef, A and Strasser, RH and Wiedemann, S | Excluded | The article was excluded based on title and abstract screening as it did not meet the inclusion and exclusion criteria. |
| β-endorphin regulates alcohol consumption induced by exercise restriction in female mice | 2016 | McGonigle, CE and Nentwig, TB and Wilson, DE and Rhinehart, EM and Grisel, JE | Excluded | The article was excluded based on title and abstract screening as it did not meet the inclusion and exclusion criteria. |
| The effect of combined hyperbaric oxygen and iloprost treatment on the prevention of spinal cord ischaemia-reperfusion injury: an experimental study | 2013 | Ilhan, G and Aksun, M and Ozpak, B and Gunes, T and Bozok, S and Durakoglugil, ME and Gucer, H and Gurbuz, A | Excluded | The article was excluded based on title and abstract screening as it did not meet the inclusion and exclusion criteria. |
| Sleep and Core Body Temperature Alterations Induced by Space Radiation in Rats | 2023 | Sanford, LD and Adkins, AM and Boden, AF and Gotthold, JD and Harris, RD and Shuboni-Mulligan, D and Wellman, LL and Britten, RA | Excluded | The article was excluded based on title and abstract screening as it did not meet the inclusion and exclusion criteria. |
| Regulation of brain temperature in winter-acclimatized reindeer under heat stress | 2011 | Blix, AS and Walloe, L and Folkow, LP | Excluded | The article was excluded based on title and abstract screening as it did not meet the inclusion and exclusion criteria. |
| Changes in ear-pinna temperature as a useful measure of stress in sheep (Ovis aries) | 2005 | Lowe, TE and Cook, CJ and Ingram, JR and Harris, PJ | Excluded | The article was excluded based on title and abstract screening as it did not meet the inclusion and exclusion criteria. |
| Physical activity and depression: Towards understanding the antidepressant mechanisms of physical activity. | 2019 | Kandola A and Ashdown-Franks G and Hendrikse J and Sabiston CM and Stubbs B | Excluded | The article was excluded based on title and abstract screening as it did not meet the inclusion and exclusion criteria. |
| Emerging roles of oxidative stress in brain aging and Alzheimer's disease. | 2021 | Ionescu-Tucker A and Cotman CW | Excluded | The article was excluded based on title and abstract screening as it did not meet the inclusion and exclusion criteria. |
| Brain metabolism in health, aging, and neurodegeneration. | 2017 | Camandola S and Mattson MP | Excluded | The article was excluded based on title and abstract screening as it did not meet the inclusion and exclusion criteria. |
| Cardiopulmonary Rehabilitation in Long-COVID-19 Patients with Persistent Breathlessness and Fatigue: The COVID-Rehab Study. | 2022 | Besnier F and Bérubé B and Malo J and Gagnon C and Grégoire CA and Juneau M and Simard F and L'Allier P and Nigam A and Iglésies-Grau J and Vincent T and Talamonti D and Dupuy EG and Mohammadi H and Gayda M and Bherer L | Excluded | The article was excluded based on title and abstract screening as it did not meet the inclusion and exclusion criteria. |
| Nutritional Factors Affecting Adult Neurogenesis and Cognitive Function. | 2017 | Poulose SM and Miller MG and Scott T and Shukitt-Hale B | Excluded | The article was excluded based on title and abstract screening as it did not meet the inclusion and exclusion criteria. |
| BDNF Impact on Biological Markers of Depression-Role of Physical Exercise and Training. | 2021 | Murawska-Ciałowicz E and Wiatr M and Ciałowicz M and Gomes de Assis G and Borowicz W and Rocha-Rodrigues S and Paprocka-Borowicz M and Marques A | Excluded | The article was excluded based on title and abstract screening as it did not meet the inclusion and exclusion criteria. |
| Physiological implications of altitude training for endurance performance at sea level: a review. | 1997 | Bailey DM and Davies B | Excluded | The article was excluded based on title and abstract screening as it did not meet the inclusion and exclusion criteria. |
| Oxygen imaging of hypoxic pockets in the mouse cerebral cortex. | 2024 | Beinlich FRM and Asiminas A and Untiet V and Bojarowska Z and Plá V and Sigurdsson B and Timmel V and Gehrig L and Graber MH and Hirase H and Nedergaard M | Excluded | The article was excluded based on title and abstract screening as it did not meet the inclusion and exclusion criteria. |
| Exercise and retinal health. | 2019 | Li HY and Rong SS and Hong X and Guo R and Yang FZ and Liang YY and Li A and So KF | Excluded | The article was excluded based on title and abstract screening as it did not meet the inclusion and exclusion criteria. |
| Irisin: An anti-inflammatory exerkine in aging and redox-mediated comorbidities. | 2023 | Trettel CDS and Pelozin BRA and Barros MP and Bachi ALL and Braga PGS and Momesso CM and Furtado GE and Valente PA and Oliveira EM and Hogervorst E and Fernandes T | Excluded | The article was excluded based on title and abstract screening as it did not meet the inclusion and exclusion criteria. |
| Exercise-linked consequences on epilepsy. | 2021 | Cavalcante BRR and Improta-Caria AC and Melo VH and De Sousa RAL | Excluded | The article was excluded based on title and abstract screening as it did not meet the inclusion and exclusion criteria. |
| The role of exercise in brain DNA damage. | 2020 | Vilela TC and de Andrade VM and Radak Z and de Pinho RA | Excluded | The article was excluded based on title and abstract screening as it did not meet the inclusion and exclusion criteria. |
| Metabolic Syndrome and Neuroprotection. | 2018 | Etchegoyen M and Nobile MH and Baez F and Posesorski B and González J and Lago N and Milei J and Otero-Losada M | Excluded | The article was excluded based on title and abstract screening as it did not meet the inclusion and exclusion criteria. |
| Neurobiology of exercise. | 2006 | Dishman RK and Berthoud HR and Booth FW and Cotman CW and Edgerton VR and Fleshner MR and Gandevia SC and Gomez-Pinilla F and Greenwood BN and Hillman CH and Kramer AF and Levin BE and Moran TH and Russo-Neustadt AA and Salamone JD and Van Hoomissen JD and Wade CE and York DA and Zigmond MJ | Excluded | The article was excluded based on title and abstract screening as it did not meet the inclusion and exclusion criteria. |
| PTSD, Immune System, and Inflammation. | 2023 | Pivac N and Vuic B and Sagud M and Nedic Erjavec G and Nikolac Perkovic M and Konjevod M and Tudor L and Svob Strac D and Uzun S and Kozumplik O and Uzun S and Mimica N | Excluded | The article was excluded based on title and abstract screening as it did not meet the inclusion and exclusion criteria. |
| The mitochondrial multi-omic response to exercise training across tissues. | 2023 | Amar D and Gay NR and Jimenez-Morales D and Beltran PMJ and Ramaker ME and Raja AN and Zhao B and Sun Y and Marwaha S and Gaul D and Hershman SG and Xia A and Lanza I and Fernandez FM and Montgomery SB and Hevener AL and Ashley EA and Walsh MJ and Sparks LM and Burant CF and Rector RS and Thyfault J and Wheeler MT and Goodpaster BH and Coen PM and Schenk S and Bodine SC and Lindholm ME | Excluded | The article was excluded based on title and abstract screening as it did not meet the inclusion and exclusion criteria. |
| The Therapeutic Role of Exercise and Probiotics in Stressful Brain Conditions. | 2022 | Martínez-Guardado I and Arboleya S and Grijota FJ and Kaliszewska A and Gueimonde M and Arias N | Excluded | The article was excluded based on title and abstract screening as it did not meet the inclusion and exclusion criteria. |
| The mammalian circadian clock and its entrainment by stress and exercise. | 2017 | Tahara Y and Aoyama S and Shibata S | Excluded | The article was excluded based on title and abstract screening as it did not meet the inclusion and exclusion criteria. |
| Physical exercise, reactive oxygen species and neuroprotection. | 2016 | Radak Z and Suzuki K and Higuchi M and Balogh L and Boldogh I and Koltai E | Excluded | The article was excluded based on title and abstract screening as it did not meet the inclusion and exclusion criteria. |
| The Role of Exercise in Maintaining Mitochondrial Proteostasis in Parkinson's Disease. | 2023 | Li J and Xu Y and Liu T and Xu Y and Zhao X and Wei J | Excluded | The article was excluded based on title and abstract screening as it did not meet the inclusion and exclusion criteria. |
| Exercise and mitochondrial remodeling to prevent age-related neurodegeneration. | 2023 | O'Reilly CL and Miller BF and Lewis TL Jr | Excluded | The article was excluded based on title and abstract screening as it did not meet the inclusion and exclusion criteria. |
| Physical Activity vs. Redox Balance in the Brain: Brain Health, Aging and Diseases. | 2021 | Sutkowy P and Woźniak A and Mila-Kierzenkowska C and Szewczyk-Golec K and Wesołowski R and Pawłowska M and Nuszkiewicz J | Excluded | The article was excluded based on title and abstract screening as it did not meet the inclusion and exclusion criteria. |
| Exercise, Nrf2 and Antioxidant Signaling in Cardiac Aging. | 2016 | Narasimhan M and Rajasekaran NS | Excluded | The article was excluded based on title and abstract screening as it did not meet the inclusion and exclusion criteria. |
| Insight Into the Mechanism of Exercise Preconditioning in Ischemic Stroke. | 2022 | Zhu Y and Sun Y and Hu J and Pan Z | Excluded | The article was excluded based on title and abstract screening as it did not meet the inclusion and exclusion criteria. |
| Comprehensive Review on Neuro-degenerative Type 3 DM. | 2022 | Chandarana CV and Roy S | Excluded | The article was excluded based on title and abstract screening as it did not meet the inclusion and exclusion criteria. |
| Dietary habits, lifestyle factors and neurodegenerative diseases. | 2020 | Popa-Wagner A and Dumitrascu DI and Capitanescu B and Petcu EB and Surugiu R and Fang WH and Dumbrava DA | Excluded | The article was excluded based on title and abstract screening as it did not meet the inclusion and exclusion criteria. |
| Oxidative stress and Down syndrome. Do antioxidants play a role in therapy? | 2014 | Muchová J and Žitňanová I and Ďuračková Z | Excluded | The article was excluded based on title and abstract screening as it did not meet the inclusion and exclusion criteria. |
| Physical activity for cognitive health promotion: An overview of the underlying neurobiological mechanisms. | 2023 | Chen C and Nakagawa S | Excluded | The article was excluded based on title and abstract screening as it did not meet the inclusion and exclusion criteria. |
| Molecular and cellular pathways contributing to brain aging. | 2021 | Zia A and Pourbagher-Shahri AM and Farkhondeh T and Samarghandian S | Excluded | The article was excluded based on title and abstract screening as it did not meet the inclusion and exclusion criteria. |
| Exploring the effect of exercise training on testicular function. | 2019 | Matos B and Howl J and Ferreira R and Fardilha M | Excluded | The article was excluded based on title and abstract screening as it did not meet the inclusion and exclusion criteria. |
| Electrophysiological and Imaging Biomarkers to Evaluate Exercise Training in Patients with Neuromuscular Disease: A Systematic Review. | 2023 | Pomp L and Jeneson JAL and van der Pol WL and Bartels B | Excluded | The article was excluded based on title and abstract screening as it did not meet the inclusion and exclusion criteria. |
| The Systemic Effects of Exercise on the Systemic Effects of Alzheimer's Disease. | 2022 | Aczel D and Gyorgy B and Bakonyi P and BukhAri R and Pinho R and Boldogh I and Yaodong G and Radak Z | Excluded | The article was excluded based on title and abstract screening as it did not meet the inclusion and exclusion criteria. |
| Central mechanisms for exercise training-induced reduction in sympatho-excitation in chronic heart failure. | 2015 | Haack KK and Zucker IH | Excluded | The article was excluded based on title and abstract screening as it did not meet the inclusion and exclusion criteria. |
| Promoting brain health through exercise and diet in older adults: a physiological perspective. | 2016 | Jackson PA and Pialoux V and Corbett D and Drogos L and Erickson KI and Eskes GA and Poulin MJ | Excluded | The article was excluded based on title and abstract screening as it did not meet the inclusion and exclusion criteria. |
| Exercise mimetics: a novel strategy to combat neuroinflammation and Alzheimer's disease. | 2024 | Zhao R | Excluded | The article was excluded based on title and abstract screening as it did not meet the inclusion and exclusion criteria. |
| Association Between Accelerometer-Derived Physical Activity Measurements and Brain Structure: A Population-Based Cohort Study. | 2022 | Fox FAU and Diers K and Lee H and Mayr A and Reuter M and Breteler MMB and Aziz NA | Excluded | The article was excluded based on title and abstract screening as it did not meet the inclusion and exclusion criteria. |
| Immune-regulating effects of exercise on cigarette smoke-induced inflammation. | 2018 | Madani A and Alack K and Richter MJ and Krüger K | Excluded | The article was excluded based on title and abstract screening as it did not meet the inclusion and exclusion criteria. |
| Exercise alters the circadian rhythm of REV-ERB-α and downregulates autophagy-related genes in peripheral and central tissues. | 2022 | da Rocha AL and Pinto AP and Bedo BLS and Morais GP and Oliveira LC and Carolino ROG and Pauli JR and Simabuco FM and de Moura LP and Ropelle ER and Cintra DE and Rivas DA and da Silva ASR | Excluded | The article was excluded based on title and abstract screening as it did not meet the inclusion and exclusion criteria. |
| Mood and metabolism: Anhedonia as a clinical target in Type 2 diabetes. | 2016 | Carter J and Swardfager W | Excluded | The article was excluded based on title and abstract screening as it did not meet the inclusion and exclusion criteria. |
| The Link between Exercise and Homocysteine in the Alzheimer's Disease: A Bioinformatic Network Model. | 2021 | Leão LL and Felício LFF and Engedal K and Tangen GG and Kristiansen KM and Santos SHS and de Paula AMB and Monteiro-Junior RS | Excluded | The article was excluded based on title and abstract screening as it did not meet the inclusion and exclusion criteria. |
| Potential Effects of Nrf2 in Exercise Intervention of Neurotoxicity Caused by Methamphetamine Oxidative Stress. | 2022 | Chen L and Ru Q and Xiong Q and Yang J and Xu G and Wu Y | Excluded | The article was excluded based on title and abstract screening as it did not meet the inclusion and exclusion criteria. |
| Resistance Training Modulates Reticulum Endoplasmic Stress, Independent of Oxidative and Inflammatory Responses, in Elderly People. | 2022 | Estébanez B and Visavadiya NP and Vargas JE and Rivera-Viloria M and Khamoui AV and de Paz JA and Huang CJ | Excluded | The article was excluded based on title and abstract screening as it did not meet the inclusion and exclusion criteria. |
| Realising the Potential of Urine and Saliva as Diagnostic Tools in Sport and Exercise Medicine. | 2017 | Lindsay A and Costello JT | Excluded | The article was excluded based on title and abstract screening as it did not meet the inclusion and exclusion criteria. |
| Effects of Resistance Exercise on Cerebral Redox Regulation and Cognition: An Interplay Between Muscle and Brain. | 2019 | Pinho RA and Aguiar AS Jr and Radák Z | Excluded | The article was excluded based on title and abstract screening as it did not meet the inclusion and exclusion criteria. |
| Aging process, cognitive decline and Alzheimer`s disease: can strength training modulate these responses? | 2015 | Portugal EM and Vasconcelos PG and Souza R and Lattari E and Monteiro-Junior RS and Machado S and Deslandes AC | Excluded | The article was excluded based on title and abstract screening as it did not meet the inclusion and exclusion criteria. |
| Exercise ameliorates hippocampal damage induced by Wi-Fi radiation; a biochemical, histological, and immunohistochemical study. | 2023 | Mohamed AO and Hafez SMNA and Ibrahim RA and Rifaai RA | Excluded | The article was excluded based on title and abstract screening as it did not meet the inclusion and exclusion criteria. |
| Neurochemical and Cognitive Beneficial Effects of Moderate Physical Activity and Catechin in Aged Rats. | 2021 | Ramis MR and Sarubbo F and Moranta D and Tejada S and Lladó J and Miralles A and Esteban S | Excluded | The article was excluded based on title and abstract screening as it did not meet the inclusion and exclusion criteria. |
| Lactate transport and signaling in the brain: potential therapeutic targets and roles in body-brain interaction. | 2015 | Bergersen LH | Excluded | The article was excluded based on title and abstract screening as it did not meet the inclusion and exclusion criteria. |
| Endocrinology and Pediatric Exercise Science-2016. | 2017 | Eliakim A | Excluded | The article was excluded based on title and abstract screening as it did not meet the inclusion and exclusion criteria. |
| Effects of Exercise Training on Neurotrophic Factors and Blood-Brain Barrier Permeability in Young-Old and Old-Old Women. | 2022 | Cho SY and Roh HT | Excluded | The article was excluded based on title and abstract screening as it did not meet the inclusion and exclusion criteria. |
| Role of physical activity in ameliorating neuropsychiatric symptoms in Alzheimer disease: A narrative review. | 2019 | Veronese N and Solmi M and Basso C and Smith L and Soysal P | Excluded | The article was excluded based on title and abstract screening as it did not meet the inclusion and exclusion criteria. |
| Aerobic exercise and nano-curcumin supplementation improve inflammation in elderly females with metabolic syndrome. | 2020 | Osali A | Excluded | The article was excluded based on title and abstract screening as it did not meet the inclusion and exclusion criteria. |
| Brain-derived neurotrophic factor mediates cognitive improvements following acute exercise. | 2017 | Borror A | Excluded | The article was excluded based on title and abstract screening as it did not meet the inclusion and exclusion criteria. |
| Pathophysiology linking depression and type 2 diabetes: Psychotherapy, physical exercise, and fecal microbiome transplantation as damage control. | 2021 | Subba R and Sandhir R and Singh SP and Mallick BN and Mondal AC | Excluded | The article was excluded based on title and abstract screening as it did not meet the inclusion and exclusion criteria. |
| Obesity promotes oxidative stress and exacerbates blood-brain barrier disruption after high-intensity exercise. | 2017 | Roh HT and Cho SY and So WY | Excluded | The article was excluded based on title and abstract screening as it did not meet the inclusion and exclusion criteria. |
| Brain-derived neurotrophic factor among patients with alcoholism. | 2021 | Martín-González C and Romero-Acevedo L and Fernández-Rodríguez CM and Medina-Vega L and García-Rodríguez A and Ortega-Toledo P and González-Navarrete L and Vera-Delgado VE and González-Reimers E | Excluded | The article was excluded based on title and abstract screening as it did not meet the inclusion and exclusion criteria. |
| Exercise for the diabetic brain: how physical training may help prevent dementia and Alzheimer's disease in T2DM patients. | 2016 | Bertram S and Brixius K and Brinkmann C | Excluded | The article was excluded based on title and abstract screening as it did not meet the inclusion and exclusion criteria. |
| Exploring the Role of Lipid-Binding Proteins and Oxidative Stress in Neurodegenerative Disorders: A Focus on the Neuroprotective Effects of Nutraceutical Supplementation and Physical Exercise. | 2022 | Scarfò G and Piccarducci R and Daniele S and Franzoni F and Martini C | Excluded | The article was excluded based on title and abstract screening as it did not meet the inclusion and exclusion criteria. |
| Exercise and Syzygium aromaticum reverse memory deficits, apoptosis and mitochondrial dysfunction of the hippocampus in Alzheimer's disease. | 2022 | Panahzadeh F and Mirnasuri R and Rahmati M | Excluded | The article was excluded based on title and abstract screening as it did not meet the inclusion and exclusion criteria. |
| Spirometry and oxidative stress after rebreather diving in warm water. | 2018 | Bosco G and Rizzato A and Quartesan S and Camporesi E and Mrakic-Sposta S and Moretti S and Balestra C and Rubini A | Excluded | The article was excluded based on title and abstract screening as it did not meet the inclusion and exclusion criteria. |
| Male and Female Mitochondria Respond Differently after Exercising in Acute Hypoxia. | 2023 | Lai Y and Loy F and Isola M and Noli R and Rinaldi A and Lobina C and Vargiu R and Cesare Marincola F and Isola R | Excluded | The article was excluded based on title and abstract screening as it did not meet the inclusion and exclusion criteria. |
| Mildronate protects heart mtDNA from oxidative stress toxicity induced by exhaustive physical exercise. | 2021 | Gureev AP and Sadovnikova IS and Shaforostova EA and Starkov AA and Popov VN | Excluded | The article was excluded based on title and abstract screening as it did not meet the inclusion and exclusion criteria. |
| Neuroprotective effects of strength training in a neuroinflammatory animal model. | 2022 | de Gregório E and Mendes GC and Somensi LB and Freire CG and Lopes LF and Lima KR and Carrazoni GS and Neves BS and Picua SS and da Silva LM and Mello-Carpes PB and Bonini JS and da Silva WC | Excluded | The article was excluded based on title and abstract screening as it did not meet the inclusion and exclusion criteria. |
| Impact of aerobic exercise on cognitive impairment and oxidative stress markers in methamphetamine-dependent patients. | 2018 | Zhang K and Zhang Q and Jiang H and Du J and Zhou C and Yu S and Hashimoto K and Zhao M | Excluded | The article was excluded based on title and abstract screening as it did not meet the inclusion and exclusion criteria. |
| Voluntary Physical Exercise Induces Expression and Epigenetic Remodeling of VegfA in the Rat Hippocampus. | 2018 | Sølvsten CAE and de Paoli F and Christensen JH and Nielsen AL | Excluded | The article was excluded based on title and abstract screening as it did not meet the inclusion and exclusion criteria. |
| Treadmill exercise training alleviates diabetes-induced depressive-like behavior and cognitive impairment by improving hippocampal CA1 neurons injury in db/db mice. | 2022 | Zhou Z and Wang M and Huang C and Li Y and Gao L and Zhu Y and Ying C and Zhou X | Excluded | The article was excluded based on title and abstract screening as it did not meet the inclusion and exclusion criteria. |
| An Enriched Environment Ameliorates Oxidative Stress and Olfactory Dysfunction in Parkinson's Disease with α-Synucleinopathy. | 2018 | Wi S and Lee JW and Kim M and Park CH and Cho SR | Excluded | The article was excluded based on title and abstract screening as it did not meet the inclusion and exclusion criteria. |
| Strength training and running elicit different neuroprotective outcomes in a β-amyloid peptide-mediated Alzheimer's disease model. | 2019 | Schimidt HL and Garcia A and Izquierdo I and Mello-Carpes PB and Carpes FP | Excluded | The article was excluded based on title and abstract screening as it did not meet the inclusion and exclusion criteria. |
| Physical and cognitive training are able to prevent recognition memory deficits related to amyloid beta neurotoxicity. | 2019 | Rossi Dare L and Garcia A and Alves N and Ventura Dias D and de Souza MA and Mello-Carpes PB | Excluded | The article was excluded based on title and abstract screening as it did not meet the inclusion and exclusion criteria. |
| All roads lead to Rome - a review of the potential mechanisms by which exerkines exhibit neuroprotective effects in Alzheimer's disease. | 2022 | Liang YY and Zhang LD and Luo X and Wu LL and Chen ZW and Wei GH and Zhang KQ and Du ZA and Li RZ and So KF and Li A | Excluded | The article was excluded based on title and abstract screening as it did not meet the inclusion and exclusion criteria. |
| Regulation of Redox Profile and Genomic Instability by Physical Exercise Contributes to Neuroprotection in Mice with Experimental Glioblastoma. | 2023 | Marqueze LFB and Costa AK and Pedroso GS and Vasconcellos FF and Pilger BI and Kindermann S and Andrade VM and Alves ACB and Nery T and Silva AA Jr and Carvalhal SRS and Zazula MF and Naliwaiko K and Fernandes LC and Radak Z and Pinho RA | Excluded | The article was excluded based on title and abstract screening as it did not meet the inclusion and exclusion criteria. |
| Increasing energy expenditure through exercise and low ambient temperature offers oxidative protection to the hypothalamus after high-fat feeding to mice. | 2022 | Jaroslawska J and Gospodarska E and Korytko A | Excluded | The article was excluded based on title and abstract screening as it did not meet the inclusion and exclusion criteria. |
| Exercise decreases susceptibility to homocysteine seizures: the role of oxidative stress. | 2014 | Hrncic D and Rasic-Markovic A and Lekovic J and Krstic D and Colovic M and Macut D and Susic V and Djuric D and Stanojlovic O | Excluded | The article was excluded based on title and abstract screening as it did not meet the inclusion and exclusion criteria. |
| Diazepam and exercise training combination synergistically reduces lipopolysaccharide-induced anxiety-like behavior and oxidative stress in the prefrontal cortex of mice. | 2023 | Amouzad Mahdirejei H and Peeri M and Azarbayjani MA and Masrour FF | Excluded | The article was excluded based on title and abstract screening as it did not meet the inclusion and exclusion criteria. |
| Induction of brain Nrf2-HO-1 pathway and antinociception after different physical training paradigms in mice. | 2018 | Tutakhail A and Nazary QA and Lebsir D and Kerdine-Romer S and Coudore F | Excluded | The article was excluded based on title and abstract screening as it did not meet the inclusion and exclusion criteria. |
| Physical Exercise Improves Aging-Related Changes in Angiotensin, IGF-1, SIRT1, SIRT3, and VEGF in the Substantia Nigra. | 2018 | Muñoz A and Corrêa CL and Lopez-Lopez A and Costa-Besada MA and Diaz-Ruiz C and Labandeira-Garcia JL | Excluded | The article was excluded based on title and abstract screening as it did not meet the inclusion and exclusion criteria. |
| The mammalian exercise pressor reflex in health and disease. | 2006 | Smith SA and Mitchell JH and Garry MG | Excluded | The article was excluded based on title and abstract screening as it did not meet the inclusion and exclusion criteria. |
| Research trends and hotspots of exercise therapy in Panvascular disease: A bibliometric analysis. | 2023 | Xu X and Xu XD and Liang Y and Xu T and Shao FR and Zhu L and Ren K | Excluded | The article was excluded based on title and abstract screening as it did not meet the inclusion and exclusion criteria. |
| Exercise and Urtica dioica extract ameliorate hippocampal insulin signaling, oxidative stress, neuroinflammation, and cognitive function in STZ-induced diabetic rats. | 2021 | Rahmati M and Keshvari M and Mirnasouri R and Chehelcheraghi F | Excluded | The article was excluded based on title and abstract screening as it did not meet the inclusion and exclusion criteria. |
| A pilot study to assess the effect of acute exercise on brain glutathione. | 2018 | Rai S and Chowdhury A and Reniers RLEP and Wood SJ and Lucas SJE and Aldred S | Excluded | The article was excluded based on title and abstract screening as it did not meet the inclusion and exclusion criteria. |
| Effects of resistance exercise on antioxidant enzyme activities and apoptosis-related protein expression of hippocampus in OLETF rats. | 2018 | Lee MK and Jung CS and Yoon JH and Lee N | Excluded | The article was excluded based on title and abstract screening as it did not meet the inclusion and exclusion criteria. |
| [Neurovascular coupling responses and cognitive function: The impact of aging and the interventional effect of exercise]. | 2023 | He YM and Wu CL and Dong YM and Wu HD and Wang Q and Jiang N | Excluded | The article was excluded based on title and abstract screening as it did not meet the inclusion and exclusion criteria. |
| The Interaction Effect of Sleep Deprivation and Treadmill Exercise in Various Durations on Spatial Memory with Respect to the Oxidative Status of Rats. | 2023 | Kholghi G and Alipour V and Rezaie M and Zarrindast MR and Vaseghi S | Excluded | The article was excluded based on title and abstract screening as it did not meet the inclusion and exclusion criteria. |
| The effects of moderate exercise and overtraining on learning and memory, hippocampal inflammatory cytokine levels, and brain oxidative stress markers in rats | 2019 | Jahangiri, Z. and Gholamnezhad, Z. and Hosseini, M. and Beheshti, F. and Kasraie, N. | Included |  |
| Voluntary exercise normalizes the proteomic landscape in muscle and brain and improves the phenotype of progeroid mice. | 2019 | Ross JM and Coppotelli G and Branca RM and Kim KM and Lehtiö J and Sinclair DA and Olson L | Excluded | The article was excluded based on title and abstract screening as it did not meet the inclusion and exclusion criteria. |
| Exercise Training Attenuates Proinflammatory Cytokines, Oxidative Stress and Modulates Neurotransmitters in the Rostral Ventrolateral Medulla of Salt-Induced Hypertensive Rats. | 2018 | Li HB and Huo CJ and Su Q and Li X and Bai J and Zhu GQ and Kang YM | Excluded | The article was excluded based on title and abstract screening as it did not meet the inclusion and exclusion criteria. |
| Beneficial effects of intermittent fasting and caloric restriction on the cardiovascular and cerebrovascular systems. | 2005 | Mattson MP and Wan R | Excluded | The article was excluded based on title and abstract screening as it did not meet the inclusion and exclusion criteria. |
| Sympathetic influence on cerebral blood flow and metabolism during exercise in humans. | 2011 | Seifert T and Secher NH | Excluded | The article was excluded based on title and abstract screening as it did not meet the inclusion and exclusion criteria. |
| Moderate exercise training and chronic caloric restriction modulate redox status in rat hippocampus. | 2011 | Santin K and da Rocha RF and Cechetti F and Quincozes-Santos A and de Souza DF and Nardin P and Rodrigues L and Leite MC and Moreira JC and Salbego CG and Gonçalves CA | Excluded | The article was excluded based on title and abstract screening as it did not meet the inclusion and exclusion criteria. |
| Exercise plays a preventive role against Alzheimer's disease. | 2010 | Radak Z and Hart N and Sarga L and Koltai E and Atalay M and Ohno H and Boldogh I | Excluded | The article was excluded based on title and abstract screening as it did not meet the inclusion and exclusion criteria. |
| ACE2 gene combined with exercise training attenuates central AngII/AT1 axis function and oxidative stress in a prehypertensive rat model. | 2022 | Chen XY and Lin C and Liu GY and Pei C and Xu GQ and Gao L and Wang SZ and Pan YX | Excluded | The article was excluded based on title and abstract screening as it did not meet the inclusion and exclusion criteria. |
| Voluntary exercise promotes beneficial anti-aging mechanisms in SAMP8 female brain. | 2015 | Bayod S and Guzmán-Brambila C and Sanchez-Roige S and Lalanza JF and Kaliman P and Ortuño-Sahagun D and Escorihuela RM and Pallàs M | Excluded | The article was excluded based on title and abstract screening as it did not meet the inclusion and exclusion criteria. |
| Effects of Regular Taekwondo Intervention on Oxidative Stress Biomarkers and Myokines in Overweight and Obese Adolescents. | 2020 | Roh HT and Cho SY and So WY | Excluded | The article was excluded based on title and abstract screening as it did not meet the inclusion and exclusion criteria. |
| Exercise-induced oxidative-nitrosative stress is associated with impaired dynamic cerebral autoregulation and blood-brain barrier leakage. | 2011 | Bailey DM and Evans KA and McEneny J and Young IS and Hullin DA and James PE and Ogoh S and Ainslie PN and Lucchesi C and Rockenbauer A and Culcasi M and Pietri S | Excluded | The article was excluded based on title and abstract screening as it did not meet the inclusion and exclusion criteria. |
| Mitochondrial dysfunction in myotonic dystrophy type 1. | 2018 | Gramegna LL and Giannoccaro MP and Manners DN and Testa C and Zanigni S and Evangelisti S and Bianchini C and Oppi F and Poda R and Avoni P and Lodi R and Liguori R and Tonon C | Excluded | The article was excluded based on title and abstract screening as it did not meet the inclusion and exclusion criteria. |
| Effects of hemp seed alone and combined with aerobic exercise on metabolic parameters, oxidative stress, and neurotrophic factors in young sedentary men. | 2022 | Mohammadrezaei A and Kavakeb A and Abbasalizad-Farhangi M and Mesgari-Abbasi M | Excluded | The article was excluded based on title and abstract screening as it did not meet the inclusion and exclusion criteria. |
| Long-term exercise training improves memory in middle-aged men and modulates peripheral levels of BDNF and Cathepsin B. | 2019 | De la Rosa A and Solana E and Corpas R and Bartrés-Faz D and Pallàs M and Vina J and Sanfeliu C and Gomez-Cabrera MC | Excluded | The article was excluded based on title and abstract screening as it did not meet the inclusion and exclusion criteria. |
| Cpt1a silencing in AgRP neurons improves cognitive and physical capacity and promotes healthy aging in male mice. | 2024 | Ibeas K and Griñán-Ferré C and Del Mar Romero M and Sebastián D and Bastías-Pérez M and Gómez R and Soler-Vázquez MC and Zagmutt S and Pallás M and Castell M and Belsham DD and Mera P and Herrero L and Serra D | Excluded | The article was excluded based on title and abstract screening as it did not meet the inclusion and exclusion criteria. |
| Multicomponent exercise program effects on fitness and cognitive function of elderlies with mild cognitive impairment: Involvement of oxidative stress and BDNF. | 2022 | Rondão CAM and Mota MP and Oliveira MM and Peixoto F and Esteves D | Excluded | The article was excluded based on title and abstract screening as it did not meet the inclusion and exclusion criteria. |
| Hotspot and Frontier Analysis of Exercise Training Therapy for Heart Failure Complicated With Depression Based on Web of Science Database and Big Data Analysis. | 2021 | Wang Y and Jia Y and Li M and Jiao S and Zhao H | Excluded | The article was excluded based on title and abstract screening as it did not meet the inclusion and exclusion criteria. |
| Aerobic training is safe and improves exercise capacity in patients with mitochondrial myopathy. | 2006 | Jeppesen TD and Schwartz M and Olsen DB and Wibrand F and Krag T and Dunø M and Hauerslev S and Vissing J | Excluded | The article was excluded based on title and abstract screening as it did not meet the inclusion and exclusion criteria. |
| Exercise training attenuates hypertension and cardiac hypertrophy by modulating neurotransmitters and cytokines in hypothalamic paraventricular nucleus. | 2014 | Jia LL and Kang YM and Wang FX and Li HB and Zhang Y and Yu XJ and Qi J and Suo YP and Tian ZJ and Zhu Z and Zhu GQ and Qin DN | Excluded | The article was excluded based on title and abstract screening as it did not meet the inclusion and exclusion criteria. |
| The effects of moderate-, strenuous- and over-training on oxidative stress markers, DNA repair, and memory, in rat brain | 2005 | Ogonovszky, H. and Berkes, I. and Kumagai, S. and Kaneko, T. and Tahara, S. and Goto, S. and Radák, Z. | Included |  |
| Effects of aerobic exercise training on peripheral brain-derived neurotrophic factor and eotaxin-1 levels in obese young men. | 2016 | Cho SY and Roh HT | Excluded | The article was excluded based on title and abstract screening as it did not meet the inclusion and exclusion criteria. |
| Aerobic training associated with an active lifestyle exerts a protective effect against oxidative damage in hypothalamus and liver: The involvement of energy metabolism. | 2021 | Scariot PPM and Manchado-Gobatto FB and Van Ginkel PR and Prolla TA and Gobatto CA | Excluded | The article was excluded based on title and abstract screening as it did not meet the inclusion and exclusion criteria. |
| Endurance Exercise Mediates Neuroprotection Against MPTP-mediated Parkinson's Disease via Enhanced Neurogenesis, Antioxidant Capacity, and Autophagy. | 2018 | Jang Y and Kwon I and Song W and Cosio-Lima LM and Lee Y | Excluded | The article was excluded based on title and abstract screening as it did not meet the inclusion and exclusion criteria. |
| Effects of mild running on substantia nigra during early neurodegeneration. | 2018 | Almeida MF and Silva CM and Chaves RS and Lima NCR and Almeida RS and Melo KP and Demasi M and Fernandes T and Oliveira EM and Netto LES and Cardoso SM and Ferrari MFR | Excluded | The article was excluded based on title and abstract screening as it did not meet the inclusion and exclusion criteria. |
| Physical Exercise for Treatment of Mood Disorders: A Critical Review. | 2016 | Hearing CM and Chang WC and Szuhany KL and Deckersbach T and Nierenberg AA and Sylvia LG | Excluded | The article was excluded based on title and abstract screening as it did not meet the inclusion and exclusion criteria. |
| Brain and muscle adaptation to high-fat diets and exercise: Metabolic transporters, enzymes and substrates in the rat cortex and muscle. | 2020 | Béland-Millar A and Takimoto M and Hamada T and Messier C | Excluded | The article was excluded based on title and abstract screening as it did not meet the inclusion and exclusion criteria. |
| Treadmill exercise rescues mitochondrial function and motor behavior in the CAG(140) knock-in mouse model of Huntington's disease. | 2020 | Caldwell CC and Petzinger GM and Jakowec MW and Cadenas E | Excluded | The article was excluded based on title and abstract screening as it did not meet the inclusion and exclusion criteria. |
| The effects of training and detraining on memory, neurotrophins and oxidative stress markers in rat brain | 2006 | Radak, Z. and Toldy, A. and Szabo, Z. and Siamilis, S. and Nyakas, C. and Silye, G. and Jakus, J. and Goto, S. | Included |  |
| Cathepsin-B dependent autophagy ameliorates steatoheaptitis in chronic exercise rats. | 2020 | Guo R and Yu Q and Liong EC and Fung ML and Tipoe GL | Excluded | The article was excluded based on title and abstract screening as it did not meet the inclusion and exclusion criteria. |
| Land/Water Aerobic Activities: Two Sides of the Same Coin. A Comparative Analysis on the Effects in Cognition of Alzheimer's Disease. | 2024 | Gelfo F and Petrosini L and Mandolesi L and Landolfo E and Caruso G and Balsamo F and Bonarota S and Bozzali M and Caltagirone C and Serra L | Excluded | The article was excluded based on title and abstract screening as it did not meet the inclusion and exclusion criteria. |
| Effect of low-intensity motor balance and coordination exercise on cognitive functions, hippocampal Aβ deposition, neuronal loss, neuroinflammation, and oxidative stress in a mouse model of Alzheimer's disease. | 2021 | Nakanishi K and Sakakima H and Norimatsu K and Otsuka S and Takada S and Tani A and Kikuchi K | Excluded | The article was excluded based on title and abstract screening as it did not meet the inclusion and exclusion criteria. |
| Functionality and Neurocognition in Patients With Bipolar Disorder After a Physical-Exercise Program (FINEXT-BD Study): Protocol of a Randomized Interventionist Program. | 2020 | García S and Gorostegi-Anduaga I and García-Corres E and Maldonado-Martín S and MacDowell KS and Bermúdez-Ampudia C and Apodaca MJ and Pérez-Landaluce I and Tobalina-Larrea I and Leza JC and González-Pinto A | Excluded | The article was excluded based on title and abstract screening as it did not meet the inclusion and exclusion criteria. |
| Impact of aerobic exercise, sex, and metabolic syndrome on markers of oxidative stress: results from the Brain in Motion study. | 2020 | Rytz CL and Pialoux V and Mura M and Martin A and Hogan DB and Hill MD and Poulin MJ | Excluded | The article was excluded based on title and abstract screening as it did not meet the inclusion and exclusion criteria. |
| Combined and individual strategy of exercise generated preconditioning and low dose copper nanoparticles serve as superlative approach to ameliorate ISO-induced myocardial infarction in rats. | 2018 | Sharma AK and Kumar A and Taneja G and Nagaich U and Deep A and Datusalia AK and Rajput SK | Excluded | The article was excluded based on title and abstract screening as it did not meet the inclusion and exclusion criteria. |
| Reduction of oxidative stress and inflammatory signaling in the commissural nucleus of the solitary tract (commNTS) and rostral ventrolateral medulla (RVLM) in treadmill trained rats. | 2021 | Farah HMAT and Farah DMAT and Nunes J and Ogihara CA and Cafarchio EM and Nadiak GKC and da Conceição RR and Giannocco G and De Angelis K and Irigoyen MC and Farah VMA and Sato MA | Excluded | The article was excluded based on title and abstract screening as it did not meet the inclusion and exclusion criteria. |
| Effect of green tea extract supplementation on exercise-induced delayed onset muscle soreness and muscular damage. | 2018 | da Silva W and Machado ÁS and Souza MA and Mello-Carpes PB and Carpes FP | Excluded | The article was excluded based on title and abstract screening as it did not meet the inclusion and exclusion criteria. |
| Does a single bout of exercise impacts BDNF, oxidative stress and epigenetic markers in spinal cord injury patients? | 2019 | Goldhardt MG and Andreia A and Dorneles GP and da Silva IR and Pochmann D and Peres A and Rostirola Elsner V | Excluded | The article was excluded based on title and abstract screening as it did not meet the inclusion and exclusion criteria. |
| Combined effect of aerobic interval training and selenium nanoparticles on expression of IL-15 and IL-10/TNF-α ratio in skeletal muscle of 4T1 breast cancer mice with cachexia. | 2017 | Molanouri Shamsi M and Chekachak S and Soudi S and Quinn LS and Ranjbar K and Chenari J and Yazdi MH and Mahdavi M | Excluded | The article was excluded based on title and abstract screening as it did not meet the inclusion and exclusion criteria. |
| Taurine supplementation reduces myeloperoxidase and matrix-metalloproteinase-9 levels and improves the effects of exercise in cognition and physical fitness in older women. | 2021 | Chupel MU and Minuzzi LG and Furtado GE and Santos ML and Ferreira JP and Filaire E and Teixeira AM | Excluded | The article was excluded based on title and abstract screening as it did not meet the inclusion and exclusion criteria. |
| Exercise training restores oxidative stress and nitric oxide synthases in the rostral ventrolateral medulla of renovascular hypertensive rats. | 2015 | Sousa LE and Magalhães WG and Bezerra FS and Santos RA and Campagnole-Santos MJ and Isoldi MC and Alzamora AC | Excluded | The article was excluded based on title and abstract screening as it did not meet the inclusion and exclusion criteria. |
| Voluntary exercise counteracts Aβ25-35-induced memory impairment in mice. | 2013 | Wang Q and Xu Z and Tang J and Sun J and Gao J and Wu T and Xiao M | Excluded | The article was excluded based on title and abstract screening as it did not meet the inclusion and exclusion criteria. |
| Mitochondrial SIRT3 Mediates Adaptive Responses of Neurons to Exercise and Metabolic and Excitatory Challenges. | 2016 | Cheng A and Yang Y and Zhou Y and Maharana C and Lu D and Peng W and Liu Y and Wan R and Marosi K and Misiak M and Bohr VA and Mattson MP | Excluded | The article was excluded based on title and abstract screening as it did not meet the inclusion and exclusion criteria. |
| The effects of exercise on depressive- and anxiety-like behaviors as well as lung and hippocampus oxidative stress in ovalbumin-sensitized juvenile rats. | 2018 | Mokhtari-Zaer A and Hosseini M and Boskabady MH | Excluded | The article was excluded based on title and abstract screening as it did not meet the inclusion and exclusion criteria. |
| Pretreatment with crocin along with treadmill exercise ameliorates motor and memory deficits in hemiparkinsonian rats by anti-inflammatory and antioxidant mechanisms. | 2019 | Shahidani S and Rajaei Z and Alaei H | Excluded | The article was excluded based on title and abstract screening as it did not meet the inclusion and exclusion criteria. |
| Regular Aerobic Exercise-Alleviated Dysregulation of CAMKIIα Carbonylation to Mitigate Parkinsonism via Homeostasis of Apoptosis With Autophagy. | 2020 | Liu W and Fu R and Wang Z and Liu S and Tang C and Li L and Yin D | Excluded | The article was excluded based on title and abstract screening as it did not meet the inclusion and exclusion criteria. |
| Short-term meditation training influences brain energy metabolism: A pilot study on (31) P MR spectroscopy. | 2021 | Gizewski ER and Steiger R and Waibel M and Pereverzyev S and Sommer PJD and Siedentopf C and Grams AE and Lenhart L and Singewald N | Excluded | The article was excluded based on title and abstract screening as it did not meet the inclusion and exclusion criteria. |
| The Effects of a Korean Ginseng, GINST15, on Hypo-Pituitary-Adrenal and Oxidative Activity Induced by Intense Work Stress. | 2018 | Flanagan SD and DuPont WH and Caldwell LK and Hardesty VH and Barnhart EC and Beeler MK and Post EM and Volek JS and Kraemer WJ | Excluded | The article was excluded based on title and abstract screening as it did not meet the inclusion and exclusion criteria. |
| Experimental Evidences Supporting the Benefits of Exercise Training in Heart Failure. | 2017 | Ichige MHA and Pereira MG and Brum PC and Michelini LC | Excluded | The article was excluded based on title and abstract screening as it did not meet the inclusion and exclusion criteria. |
| α-Synuclein Aggregates with β-Amyloid or Tau in Human Red Blood Cells: Correlation with Antioxidant Capability and Physical Exercise in Human Healthy Subjects. | 2018 | Daniele S and Pietrobono D and Fusi J and Iofrida C and Chico L and Petrozzi L and Gerfo AL and Baldacci F and Galetta F and Siciliano G and Bonuccelli U and Santoro G and Trincavelli ML and Franzoni F and Martini C | Excluded | The article was excluded based on title and abstract screening as it did not meet the inclusion and exclusion criteria. |
| Long-term aerobic exercise increases redox-active iron through nitric oxide in rat hippocampus. | 2014 | Chen Q and Xiao DS | Excluded | The article was excluded based on title and abstract screening as it did not meet the inclusion and exclusion criteria. |
| Effects of Exercise on Inflammatory, Oxidative, and Neurotrophic Biomarkers on Cognitively Impaired Individuals Diagnosed With Dementia or Mild Cognitive Impairment: A Systematic Review and Meta-Analysis. | 2019 | Stigger FS and Zago Marcolino MA and Portela KM and Plentz RDM | Excluded | The article was excluded based on title and abstract screening as it did not meet the inclusion and exclusion criteria. |
| Exercise combined with a probiotics treatment alters the microbiome, but moderately affects signalling pathways in the liver of male APP/PS1 transgenic mice. | 2020 | Téglás T and Ábrahám D and Jókai M and Kondo S and Mohammadi R and Fehér J and Szabó D and Wilhelm M and Radák Z | Excluded | The article was excluded based on title and abstract screening as it did not meet the inclusion and exclusion criteria. |
| COXPD9 in an individual from Puerto Rico and literature review. | 2021 | Alsharhan H and Muraresku C and Ganetzky RD | Excluded | The article was excluded based on title and abstract screening as it did not meet the inclusion and exclusion criteria. |
| One-Week High-Intensity Interval Training Increases Hippocampal Plasticity and Mitochondrial Content without Changes in Redox State. | 2020 | Dos Santos JR and Bortolanza M and Ferrari GD and Lanfredi GP and do Nascimento GC and Azzolini AECS and Del Bel E and de Campos AC and Faça VM and Vulczak A and Alberici LC | Excluded | The article was excluded based on title and abstract screening as it did not meet the inclusion and exclusion criteria. |
| Differential proteomic and behavioral effects of long-term voluntary exercise in wild-type and APP-overexpressing transgenics. | 2015 | Rao SK and Ross JM and Harrison FE and Bernardo A and Reiserer RS and Reiserer RS and Mobley JA and McDonald MP | Excluded | The article was excluded based on title and abstract screening as it did not meet the inclusion and exclusion criteria. |
| One-week cocoa flavanol intake increases prefrontal cortex oxygenation at rest and during moderate-intensity exercise in normoxia and hypoxia. | 2018 | Decroix L and Tonoli C and Lespagnol E and Balestra C and Descat A and Drittij-Reijnders MJ and Blackwell JR and Stahl W and Jones AM and Weseler AR and Bast A and Meeusen R and Heyman E | Excluded | The article was excluded based on title and abstract screening as it did not meet the inclusion and exclusion criteria. |
| Irisin lowers blood pressure by activating the Nrf2 signaling pathway in the hypothalamic paraventricular nucleus of spontaneously hypertensive rats. | 2020 | Huo CJ and Yu XJ and Sun YJ and Li HB and Su Q and Bai J and Li Y and Liu KL and Qi J and Zhou SW and Jia N and Zhu GQ and Liu JJ and Kang YM | Excluded | The article was excluded based on title and abstract screening as it did not meet the inclusion and exclusion criteria. |
| Treadmill Exercise Exerts Neuroprotection and Regulates Microglial Polarization and Oxidative Stress in a Streptozotocin-Induced Rat Model of Sporadic Alzheimer's Disease. | 2017 | Lu Y and Dong Y and Tucker D and Wang R and Ahmed ME and Brann D and Zhang Q | Excluded | The article was excluded based on title and abstract screening as it did not meet the inclusion and exclusion criteria. |
| Physical activity as a promoter of stress resilience: An analysis of behavioral effects and brain connectivity with cytochrome c-oxidase activity in adult male Wistar rats. | 2024 | Begega A and López M and Cuesta-López I and Jove CI and Izquierdo MC | Excluded | The article was excluded based on title and abstract screening as it did not meet the inclusion and exclusion criteria. |
| Hypoxic exercise training reduces senescent T-lymphocyte subsets in blood. | 2011 | Wang JS and Chen WL and Weng TP | Excluded | The article was excluded based on title and abstract screening as it did not meet the inclusion and exclusion criteria. |
| Lysosomal Proteolysis Is Associated With Exercise-Induced Improvement of Mitochondrial Quality Control in Aged Hippocampus. | 2017 | Luo L and Dai JR and Guo SS and Lu AM and Gao XF and Gu YR and Zhang XF and Xu HD and Wang Y and Zhu Z and Wood LJ and Qin ZH | Excluded | The article was excluded based on title and abstract screening as it did not meet the inclusion and exclusion criteria. |
| Natriuretic peptides enhance the oxidative capacity of human skeletal muscle. | 2012 | Engeli S and Birkenfeld AL and Badin PM and Bourlier V and Louche K and Viguerie N and Thalamas C and Montastier E and Larrouy D and Harant I and de Glisezinski I and Lieske S and Reinke J and Beckmann B and Langin D and Jordan J and Moro C | Excluded | The article was excluded based on title and abstract screening as it did not meet the inclusion and exclusion criteria. |
| Aerobic and neuromuscular training: effect on the capacity, efficiency, and fatigability of patients with traumatic brain injuries. | 1990 | Jankowski LW and Sullivan SJ | Excluded | The article was excluded based on title and abstract screening as it did not meet the inclusion and exclusion criteria. |
| High Intensity Interval Training Ameliorates Mitochondrial Dysfunction in the Left Ventricle of Mice with Type 2 Diabetes. | 2019 | Bækkerud FH and Salerno S and Ceriotti P and Morland C and Storm-Mathisen J and Bergersen LH and Høydal MA and Catalucci D and Stølen TO | Excluded | The article was excluded based on title and abstract screening as it did not meet the inclusion and exclusion criteria. |
| Utility of Growth Differentiation Factor-15, A Marker of Oxidative Stress and Inflammation, in Chronic Heart Failure: Insights From the HF-ACTION Study. | 2017 | Sharma A and Stevens SR and Lucas J and Fiuzat M and Adams KF and Whellan DJ and Donahue MP and Kitzman DW and Piña IL and Zannad F and Kraus WE and O'Connor CM and Felker GM | Excluded | The article was excluded based on title and abstract screening as it did not meet the inclusion and exclusion criteria. |
| Influence of high altitude on cerebral blood flow and fuel utilization during exercise and recovery. | 2014 | Smith KJ and MacLeod D and Willie CK and Lewis NC and Hoiland RL and Ikeda K and Tymko MM and Donnelly J and Day TA and MacLeod N and Lucas SJ and Ainslie PN | Excluded | The article was excluded based on title and abstract screening as it did not meet the inclusion and exclusion criteria. |
| Role of skeletal muscles impairment and brain oxygenation in limiting oxidative metabolism during exercise after bed rest. | 2010 | Porcelli S and Marzorati M and Lanfranconi F and Vago P and Pisot R and Grassi B | Excluded | The article was excluded based on title and abstract screening as it did not meet the inclusion and exclusion criteria. |
| Effect of Exercise Intensity on Neurotrophic Factors and Blood-Brain Barrier Permeability Induced by Oxidative-Nitrosative Stress in Male College Students. | 2017 | Roh HT and Cho SY and Yoon HG and So WY | Excluded | The article was excluded based on title and abstract screening as it did not meet the inclusion and exclusion criteria. |
| Aging-related Increase in Rho Kinase Activity in the Nigral Region Is Counteracted by Physical Exercise. | 2016 | Muñoz A and Corrêa CL and Villar-Cheda B and Costa-Besada MA and Labandeira-Garcia JL | Excluded | The article was excluded based on title and abstract screening as it did not meet the inclusion and exclusion criteria. |
| Effects of exercise on markers of oxidative stress: an Ancillary analysis of the Alberta Physical Activity and Breast Cancer Prevention Trial. | 2016 | Friedenreich CM and Pialoux V and Wang Q and Shaw E and Brenner DR and Waltz X and Conroy SM and Johnson R and Woolcott CG and Poulin MJ and Courneya KS | Excluded | The article was excluded based on title and abstract screening as it did not meet the inclusion and exclusion criteria. |
| Exercise preconditioning and low dose copper nanoparticles exhibits cardioprotection through targeting GSK-3β phosphorylation in ischemia/reperfusion induced myocardial infarction. | 2018 | Sharma AK and Kumar A and Sahu M and Sharma G and Datusalia AK and Rajput SK | Excluded | The article was excluded based on title and abstract screening as it did not meet the inclusion and exclusion criteria. |
| Oxidative stress and aging: studies on an East-Sicilian, ultraoctagenarian population living in institutes or at home. | 2004 | Maugeri D and Santangelo A and Bonanno MR and Testai M and Abbate S and Lo Giudice F and Mamazza C and Pugllsi N and Panebianco P | Excluded | The article was excluded based on title and abstract screening as it did not meet the inclusion and exclusion criteria. |
| Exercise training attenuates renovascular hypertension partly via RAS- ROS- glutamate pathway in the hypothalamic paraventricular nucleus. | 2016 | Zhang Y and Yu XJ and Chen WS and Gao HL and Liu KL and Shi XL and Fan XY and Jia LL and Cui W and Zhu GQ and Liu JJ and Kang YM | Excluded | The article was excluded based on title and abstract screening as it did not meet the inclusion and exclusion criteria. |
| Influence of l-carnosine on pro-antioxidant status in elite kayakers and canoeists. | 2014 | Slowinska-Lisowska M and Zembron-Lacny A and Rynkiewicz M and Rynkiewicz T and Kopec W | Excluded | The article was excluded based on title and abstract screening as it did not meet the inclusion and exclusion criteria. |
| Acylcarnitines as markers of exercise-associated fuel partitioning, xenometabolism, and potential signals to muscle afferent neurons. | 2017 | Zhang J and Light AR and Hoppel CL and Campbell C and Chandler CJ and Burnett DJ and Souza EC and Casazza GA and Hughen RW and Keim NL and Newman JW and Hunter GR and Fernandez JR and Garvey WT and Harper ME and Fiehn O and Adams SH | Excluded | The article was excluded based on title and abstract screening as it did not meet the inclusion and exclusion criteria. |
| Endurance exercise training and diferuloyl methane supplement: changes in neurotrophic factor and oxidative stress induced by lead in rat brain. | 2013 | Dabidi RV and Hosseinzadeh S and Mahjoub S and Hosseinzadeh M and Myers J | Excluded | The article was excluded based on title and abstract screening as it did not meet the inclusion and exclusion criteria. |
| Alterations of the oxidative status in rat hippocampus and prodepressant effect of chronic testosterone enanthate administration. | 2017 | Joksimović J and Selaković D and Jakovljević V and Mihailović V and Katanić J and Boroja T and Rosić G | Excluded | The article was excluded based on title and abstract screening as it did not meet the inclusion and exclusion criteria. |
| Control of Protein and Energy Metabolism in the Pituitary Gland in Response to Three-Week Running Training in Adult Male Mice. | 2021 | Walz C and Brenmoehl J and Trakooljul N and Noce A and Caffier C and Ohde D and Langhammer M and Wimmers K and Ponsuksili S and Hoeflich A | Excluded | The article was excluded based on title and abstract screening as it did not meet the inclusion and exclusion criteria. |
| Impact of exercise on mitochondrial transcription factor expression and damage in the striatum of a chronic mouse model of Parkinson's disease. | 2011 | Patki G and Lau YS | Excluded | The article was excluded based on title and abstract screening as it did not meet the inclusion and exclusion criteria. |
| The swimming control group in spatial reference memory task: analysis of its motor cortex activity. | 2020 | Cid-Duarte S and Gutiérrez-Menéndez A and Zorzo C and Arias JL and Méndez M | Excluded | The article was excluded based on title and abstract screening as it did not meet the inclusion and exclusion criteria. |
| The exercise redox paradigm in the Down's syndrome: improvements in motor function and increases in blood oxidative status in young adults. | 2008 | Aguiar AS Jr and Tuon T and Albuquerque MM and Rocha GS and Speck AE and Araújo JC and Dafré AL and Prediger RD and Pinho RA | Excluded | The article was excluded based on title and abstract screening as it did not meet the inclusion and exclusion criteria. |
| Exercise training can induce cardiac autophagy at end-stage chronic conditions: insights from a graft-versus-host-disease mouse model. | 2014 | Fiuza-Luces C and Delmiro A and Soares-Miranda L and González-Murillo Á and Martínez-Palacios J and Ramírez M and Lucia A and Morán M | Excluded | The article was excluded based on title and abstract screening as it did not meet the inclusion and exclusion criteria. |
| Immune and inflammation responses to a 3-day period of intensified running versus cycling. | 2014 | Nieman DC and Luo B and Dréau D and Henson DA and Shanely RA and Dew D and Meaney MP | Excluded | The article was excluded based on title and abstract screening as it did not meet the inclusion and exclusion criteria. |
| Treadmill Exercise Attenuates α-Synuclein Levels by Promoting Mitochondrial Function and Autophagy Possibly via SIRT1 in the Chronic MPTP/P-Induced Mouse Model of Parkinson's Disease. | 2017 | Koo JH and Cho JY | Excluded | The article was excluded based on title and abstract screening as it did not meet the inclusion and exclusion criteria. |
| Protection of swimming-induced oxidative stress in some vital organs by the treatment of composite extract of Withania somnifera, Ocimum sanctum and Zingiber officinalis in male rat. | 2009 | Misra DS and Maiti R and Ghosh D | Excluded | The article was excluded based on title and abstract screening as it did not meet the inclusion and exclusion criteria. |
| Regulation of trace elements and redox status in striatum of adult rats by long-term aerobic exercise depends on iron uptakes. | 2017 | Wu HB and Xiao DS | Excluded | The article was excluded based on title and abstract screening as it did not meet the inclusion and exclusion criteria. |
| Peripheral brain-derived neurotrophic factor is related to cardiovascular risk factors in active and inactive elderly men. | 2016 | Zembron-Lacny A and Dziubek W and Rynkiewicz M and Morawin B and Woźniewski M | Excluded | The article was excluded based on title and abstract screening as it did not meet the inclusion and exclusion criteria. |
| Resistance training in patients with single, large-scale deletions of mitochondrial DNA. | 2008 | Murphy JL and Blakely EL and Schaefer AM and He L and Wyrick P and Haller RG and Taylor RW and Turnbull DM and Taivassalo T | Excluded | The article was excluded based on title and abstract screening as it did not meet the inclusion and exclusion criteria. |
| Unchanged cerebral blood flow and oxidative metabolism after acclimatization to high altitude. | 2002 | Møller K and Paulson OB and Hornbein TF and Colier WN and Paulson AS and Roach RC and Holm S and Knudsen GM | Excluded | The article was excluded based on title and abstract screening as it did not meet the inclusion and exclusion criteria. |
| Aspects of human bioenergetics as studied in vivo by magnetic resonance spectroscopy. | 1998 | Barbiroli B and Iotti S and Lodi R | Excluded | The article was excluded based on title and abstract screening as it did not meet the inclusion and exclusion criteria. |
| Impact of treadmill running and sex on hippocampal neurogenesis in the mouse model of amyotrophic lateral sclerosis. | 2012 | Ma X and Hamadeh MJ and Christie BR and Foster JA and Tarnopolsky MA | Excluded | The article was excluded based on title and abstract screening as it did not meet the inclusion and exclusion criteria. |
| Secondary' 4216/ND1 and 13708/ND5 Leber's hereditary optic neuropathy mitochondrial DNA mutations do not further impair in vivo mitochondrial oxidative metabolism when associated with the 11778/ND4 mitochondrial DNA mutation. | 2000 | Lodi R and Montagna P and Cortelli P and Iotti S and Cevoli S and Carelli V and Barbiroli B | Excluded | The article was excluded based on title and abstract screening as it did not meet the inclusion and exclusion criteria. |
| Proteomic Profile of Carbonylated Proteins Screen the Regulation of Calmodulin-Dependent Protein Kinases-AMPK-Beclin1 in Aerobic Exercise-Induced Autophagy in Middle-Aged Rat Hippocampus. | 2019 | Liu W and Xia Y and Kuang H and Wang Z and Liu S and Tang C and Yin D | Excluded | The article was excluded based on title and abstract screening as it did not meet the inclusion and exclusion criteria. |
| Neuroprotective effects of swimming training in a mouse model of Parkinson's disease induced by 6-hydroxydopamine. | 2014 | Goes AT and Souza LC and Filho CB and Del Fabbro L and De Gomes MG and Boeira SP and Jesse CR | Excluded | The article was excluded based on title and abstract screening as it did not meet the inclusion and exclusion criteria. |
| Enforced expression of protein kinase C in skeletal muscle causes physical inactivity, fatty liver and insulin resistance in the brain. | 2010 | Hennige AM and Heni M and Machann J and Staiger H and Sartorius T and Hoene M and Lehmann R and Weigert C and Peter A and Bornemann A and Kroeber S and Pujol A and Franckhauser S and Bosch F and Schick F and Lammers R and Häring HU | Excluded | The article was excluded based on title and abstract screening as it did not meet the inclusion and exclusion criteria. |
| [Mechanisms of cardiovascular co-morbidity in patients with obstructive sleep apnoea syndrome]. | 2012 | Meier M and Andreas S | Excluded | The article was excluded based on title and abstract screening as it did not meet the inclusion and exclusion criteria. |
| Short-term dichloroacetate treatment improves indices of cerebral metabolism in patients with mitochondrial disorders. | 1995 | De Stefano N and Matthews PM and Ford B and Genge A and Karpati G and Arnold DL | Excluded | The article was excluded based on title and abstract screening as it did not meet the inclusion and exclusion criteria. |
| Exploring the consequences of mitochondrial differences arising through hybridization of sunfish. | 2014 | Mathers KE and Cox JA and Wang Y and Moyes CD | Excluded | The article was excluded based on title and abstract screening as it did not meet the inclusion and exclusion criteria. |
| Treadmill exercise prevents diabetes-induced increases in lipid peroxidation and decreases in Cu,Zn-superoxide dismutase levels in the hippocampus of Zucker diabetic fatty rats. | 2015 | Kim JW and Chae J and Nam SM and Kim YN and Yoo DY and Choi JH and Jung HY and Song W and Hwang IK and Seong JK and Yoon YS | Excluded | The article was excluded based on title and abstract screening as it did not meet the inclusion and exclusion criteria. |
| Prunus mume extract ameliorates exercise-induced fatigue in trained rats. | 2008 | Kim S and Park SH and Lee HN and Park T | Excluded | The article was excluded based on title and abstract screening as it did not meet the inclusion and exclusion criteria. |
| Innovative approach to interpret the variability of biomarkers after ultra-endurance exercise: the multifactorial analysis. | 2014 | Vassalle C and Piaggi P and Weltman N and Prontera C and Garbella E and Menicucci D and Lubrano V and Piarulli A and Castagnini C and Passera M and Pellegrini S and Metelli MR and Bedini R and Gemignani A and Pingitore A | Excluded | The article was excluded based on title and abstract screening as it did not meet the inclusion and exclusion criteria. |
| Myeloperoxidase levels predict executive function. | 2012 | Haslacher H and Perkmann T and Lukas I and Barth A and Ponocny-Seliger E and Michlmayr M and Scheichenberger V and Wagner O and Winker R | Excluded | The article was excluded based on title and abstract screening as it did not meet the inclusion and exclusion criteria. |
| Exercise intolerance and developmental delay associated with a novel mitochondrial ND5 mutation. | 2015 | Fang H and Shi H and Li X and Sun D and Li F and Li B and Ding Y and Ma Y and Liu Y and Zhang Y and Shen L and Bai Y and Yang Y and Lu J | Excluded | The article was excluded based on title and abstract screening as it did not meet the inclusion and exclusion criteria. |
| Assessment of the health status in the Massa Lombarda cohort: a preliminary description of the program evaluating cardio-cerebro-vascular disease risk factors and quality of life in an elderly population. | 2004 | Nascetti S and Linarello S and Scurti M and Grandi E and Gaddoni M and Noera G and Gaddi A | Excluded | The article was excluded based on title and abstract screening as it did not meet the inclusion and exclusion criteria. |
| Abnormal in vivo skeletal muscle energy metabolism in Huntington's disease and dentatorubropallidoluysian atrophy. | 2000 | Lodi R and Schapira AH and Manners D and Styles P and Wood NW and Taylor DJ and Warner TT | Excluded | The article was excluded based on title and abstract screening as it did not meet the inclusion and exclusion criteria. |
| Maintained cerebral metabolic ratio during exercise in patients with beta-adrenergic blockade. | 2009 | Gam CM and Rasmussen P and Secher NH and Seifert T and Larsen FS and Nielsen HB | Excluded | The article was excluded based on title and abstract screening as it did not meet the inclusion and exclusion criteria. |
| Environmental factors and membrane polyunsaturated fatty acids in schizophrenia. | 2003 | Reddy RD and Yao JK | Excluded | The article was excluded based on title and abstract screening as it did not meet the inclusion and exclusion criteria. |
| Correlation between regional cerebral blood flow and oxidative metabolism. In vivo studies in man. | 1976 | Raichle ME and Grubb RL Jr and Gado MH and Eichling JO and Ter-Pogossian MM | Excluded | The article was excluded based on title and abstract screening as it did not meet the inclusion and exclusion criteria. |
| [Clinical biochemical evaluation of central fatigue with 24-hour continuous exercise]. | 2005 | Ohta M and Hirai N and Ono Y and Ohara M and Saito S and Horiguchi S and Watanabe M and Tokashiki A and Kawai A and Andou T and Shioji I and Noguchi T and Morizuka M and Suzuki M and Imanishi A and Takeda N and Machida K | Excluded | The article was excluded based on title and abstract screening as it did not meet the inclusion and exclusion criteria. |
| What limits [V(·)]O(2max)? A symposium held at the BASES Conference, 6 September 2010. | 2012 | Spurway NC and Ekblom B and Noakes TD and Wagner PD | Excluded | The article was excluded based on title and abstract screening as it did not meet the inclusion and exclusion criteria. |
| Brain energy metabolism studied by 31P-MR spectroscopy in a case of migraine with prolonged aura. | 1992 | Sacquegna T and Lodi R and De Carolis P and Tinuper P and Cortelli P and Zaniol P and Funicello R and Montagna P and Barbiroli B | Excluded | The article was excluded based on title and abstract screening as it did not meet the inclusion and exclusion criteria. |
| Epilepsia partialis continua associated with NADH-coenzyme Q reductase deficiency. | 1995 | Antozzi C and Franceschetti S and Filippini G and Barbiroli B and Savoiardo M and Fiacchino F and Rimoldi M and Lodi R and Zaniol P and Zeviani M | Excluded | The article was excluded based on title and abstract screening as it did not meet the inclusion and exclusion criteria. |
| Positron emission tomography and magnetic resonance spectroscopy of cerebral glycolysis in children with congenital lactic acidosis. | 1995 | Duncan DB and Herholz K and Kugel H and Roth B and Ruitenbeek W and Heindel W and Wienhard K and Heiss WD | Excluded | The article was excluded based on title and abstract screening as it did not meet the inclusion and exclusion criteria. |
| A 31P-magnetic resonance spectroscopy and biochemical study of the mo(vbr) mouse: potential model for the mitochondrial encephalomyopathies. | 1997 | Tracey I and Dunn JF and Radda GK | Excluded | The article was excluded based on title and abstract screening as it did not meet the inclusion and exclusion criteria. |
| LunHab: interactive effects of a 10 day sustained exposure to hypoxia and bedrest on aerobic exercise capacity in male lowlanders. | 2017 | Keramidas ME and Mekjavic IB and Eiken O | Excluded | The article was excluded based on title and abstract screening as it did not meet the inclusion and exclusion criteria. |
| Swimming exercise protective effect on waterpipe tobacco smoking-induced impairment of memory and oxidative stress. | 2019 | Alzoubi KH and Halboup AM and Alomari MA and Khabour OF | Excluded | The article was excluded based on title and abstract screening as it did not meet the inclusion and exclusion criteria. |
| Integrated brain restoration after ischemic stroke--medical management, risk factors, nutrients, and other interventions for managing inflammation and enhancing brain plasticity. | 2009 | Kidd PM | Excluded | The article was excluded based on title and abstract screening as it did not meet the inclusion and exclusion criteria. |
| Short-term treadmill exercise increased tau insolubility and neuroinflammation in tauopathy model mice. | 2016 | Elahi M and Motoi Y and Matsumoto SE and Hasan Z and Ishiguro K and Hattori N | Excluded | The article was excluded based on title and abstract screening as it did not meet the inclusion and exclusion criteria. |
| Towards a comprehensive theory of obesity and a healthy diet: The causal role of oxidative stress in food addiction and obesity. | 2020 | Tobore TO | Excluded | The article was excluded based on title and abstract screening as it did not meet the inclusion and exclusion criteria. |
| Physical-Exercise-Induced Antioxidant Effects on the Brain and Skeletal Muscle. | 2022 | Souza J and da Silva RA and da Luz Scheffer D and Penteado R and Solano A and Barros L and Budde H and Trostchansky A and Latini A | Excluded | The article was excluded based on title and abstract screening as it did not meet the inclusion and exclusion criteria. |
| Repeated Supramaximal Exercise-Induced Oxidative Stress: Effect of β-Alanine Plus Creatine Supplementation. | 2016 | Belviranli M and Okudan N and Revan S and Balci S and Gokbel H | Excluded | The article was excluded based on title and abstract screening as it did not meet the inclusion and exclusion criteria. |
| Regulation of exercise blood flow: Role of free radicals. | 2016 | Trinity JD and Broxterman RM and Richardson RS | Excluded | The article was excluded based on title and abstract screening as it did not meet the inclusion and exclusion criteria. |
| Effects of Probiotic Supplementation on Exercise and the Underlying Mechanisms. | 2023 | Zhang L and Zhang R and Li L | Excluded | The article was excluded based on title and abstract screening as it did not meet the inclusion and exclusion criteria. |
| Fish oil and treadmill exercise have age-dependent effects on episodic memory and oxidative state of the hippocampus. | 2017 | Macêdo PFC and de Melo JSV and Costa LAR and Braz GRF and de Sousa SM and Lagranha CJ and Hornsby MB | Excluded | The article was excluded based on title and abstract screening as it did not meet the inclusion and exclusion criteria. |
| Effects of treadmill exercise and chronic stress on anxiety-like behavior, neuronal activity, and oxidative stress in basolateral amygdala in morphine-treated rats. | 2023 | Shahidani S and Jokar Z and Alaei H and Reisi P | Excluded | The article was excluded based on title and abstract screening as it did not meet the inclusion and exclusion criteria. |
| Modulatory calcineurin-interacting proteins 1 and 2 function as calcineurin facilitators in vivo. | 2006 | Sanna B and Brandt EB and Kaiser RA and Pfluger P and Witt SA and Kimball TR and van Rooij E and De Windt LJ and Rothenberg ME and Tschop MH and Benoit SC and Molkentin JD | Excluded | The article was excluded based on title and abstract screening as it did not meet the inclusion and exclusion criteria. |
| Oxidative metabolism in YAC128 mouse model of Huntington's disease. | 2015 | Hamilton J and Pellman JJ and Brustovetsky T and Harris RA and Brustovetsky N | Excluded | The article was excluded based on title and abstract screening as it did not meet the inclusion and exclusion criteria. |
| Effectiveness of supplementation with date seed (Phoenix dactylifera) as a functional food on inflammatory markers, muscle damage, and BDNF following high-intensity interval training: a randomized, double-blind, placebo-controlled trial. | 2023 | Moslemi E and Dehghan P and Khalafi M | Excluded | The article was excluded based on title and abstract screening as it did not meet the inclusion and exclusion criteria. |
| Treadmill running and Levisticum Officinale extract protect against LPS-induced memory deficits by modulating neurogenesis, neuroinflammation and oxidative stress. | 2023 | Amraie E and Pouraboli I and Salehi H and Rajaei Z | Excluded | The article was excluded based on title and abstract screening as it did not meet the inclusion and exclusion criteria. |
| Endurance exercise does not modify nerve fibre morphology in the rat soleus nerve. | 1984 | Key B and Parker AW and Giorgi PP | Excluded | The article was excluded based on title and abstract screening as it did not meet the inclusion and exclusion criteria. |
| [Clinical and investigative approaches in mitochondrial diseases. A review of 15 cases]. | 1994 | Arpa J and Campos Y and Cruz Martínez A and Gutiérrez Molina M and Arenas J and Alonso M and Plaza I and Morales C and Palomo F and Barreiro P and et al. | Excluded | The article was excluded based on title and abstract screening as it did not meet the inclusion and exclusion criteria. |
| Tai Chi is an effective form of exercise to reduce markers of frailty in older age. | 2020 | Kasim NF and Veldhuijzen van Zanten J and Aldred S | Excluded | The article was excluded based on title and abstract screening as it did not meet the inclusion and exclusion criteria. |
| Nutraceuticals and Physical Activity as Antidepressants: The Central Role of the Gut Microbiota. | 2022 | Donati Zeppa S and Ferrini F and Agostini D and Amatori S and Barbieri E and Piccoli G and Sestili P and Stocchi V | Excluded | The article was excluded based on title and abstract screening as it did not meet the inclusion and exclusion criteria. |
| ApoE Genotype-Dependent Response to Antioxidant and Exercise Interventions on Brain Function. | 2020 | Chaudhari K and Wong JM and Vann PH and Como T and O'Bryant SE and Sumien N | Excluded | The article was excluded based on title and abstract screening as it did not meet the inclusion and exclusion criteria. |
| The effect of Tribulus terrestris supplementation on inflammation, oxidative stress, and performance of recreational runners: study protocol for a randomized placebo-controlled trial. | 2022 | Nejati M and Dehghan P and Khani M and Sarbakhsh P | Excluded | The article was excluded based on title and abstract screening as it did not meet the inclusion and exclusion criteria. |
| Effect of propranolol plus exercise on melatonin and growth hormone levels in children with growth delay. | 2001 | Muñoz-Hoyos A and Hubber E and Escames G and Molina-Carballo A and Macías M and Valenzuela-Ruiz A and Fernández-García JM and Acuña-Castroviejo D | Excluded | The article was excluded based on title and abstract screening as it did not meet the inclusion and exclusion criteria. |
| Neuroprotective Benefits of Exercise and MitoQ on Memory Function, Mitochondrial Dynamics, Oxidative Stress, and Neuroinflammation in D-Galactose-Induced Aging Rats. | 2021 | Jeong JH and Koo JH and Yook JS and Cho JY and Kang EB | Excluded | The article was excluded based on title and abstract screening as it did not meet the inclusion and exclusion criteria. |
| Treadmill exercise attenuates 3,4-methylenedioxymethamphetamine-induced memory impairment through a decrease apoptosis in male rat hippocampus. | 2017 | Gharebaghi A and Amiri I and Salehi I and Shahidi S and Komaki A and Mehdizadeh M and Moravej FG and Asl SS | Excluded | The article was excluded based on title and abstract screening as it did not meet the inclusion and exclusion criteria. |
| Biological Rationale for Regular Physical Exercise as an Effective Intervention for the Prevention and Treatment of Depressive Disorders. | 2016 | Pareja-Galeano H and Mayero S and Perales M and Garatachea N and Santos-Lozano A and Fiuza-Luces C and Emanuele E and Gálvez BG and Sanchis-Gomar F and Lucia A | Excluded | The article was excluded based on title and abstract screening as it did not meet the inclusion and exclusion criteria. |
| Targeting skeletal muscle mitochondria to prevent type 2 diabetes in youth. | 2015 | Gordon JW and Dolinsky VW and Mughal W and Gordon GR and McGavock J | Excluded | The article was excluded based on title and abstract screening as it did not meet the inclusion and exclusion criteria. |
| Exercise-Boosted Mitochondrial Remodeling in Parkinson's Disease. | 2022 | Magaña JC and Deus CM and Giné-Garriga M and Montané J and Pereira SP | Excluded | The article was excluded based on title and abstract screening as it did not meet the inclusion and exclusion criteria. |
| Mild regular treadmill exercise ameliorated the detrimental effects of acute sleep deprivation on spatial memory. | 2021 | Sahin L and Cevik OS and Cevik K and Guven C and Taskin E and Kocahan S | Excluded | The article was excluded based on title and abstract screening as it did not meet the inclusion and exclusion criteria. |
| A randomized trial of coenzyme Q10 in mitochondrial disorders. | 2010 | Glover EI and Martin J and Maher A and Thornhill RE and Moran GR and Tarnopolsky MA | Excluded | The article was excluded based on title and abstract screening as it did not meet the inclusion and exclusion criteria. |
| Effect of NAC treatment and physical activity on neuroinflammation in subchronic Parkinsonism; is physical activity essential? | 2018 | Gil-Martínez AL and Cuenca L and Sánchez C and Estrada C and Fernández-Villalba E and Herrero MT | Excluded | The article was excluded based on title and abstract screening as it did not meet the inclusion and exclusion criteria. |
| Impact of swimming exercise on inflammation in medullary areas of sympathetic outflow control in spontaneously hypertensive rats. | 2018 | Maglione AV and Taranto P and Hamermesz B and Souza JS and Cafarchio EM and Ogihara CA and Maciel RMB and Giannocco G and Sato MA | Excluded | The article was excluded based on title and abstract screening as it did not meet the inclusion and exclusion criteria. |
| Whole-body vibration promotes lipid mobilization in hypothalamic obesity rat. | 2021 | de Andrade BZ and Zazula MF and Bittencourt Guimarães AT and Sagae Schneider SC and Boaretto ML and Felicio Poncio AC and Hoff Nunes Maciel JI and de Oliveira CMT and Costa RM and Flor Bertolini GR and Chasko Ribeiro LF | Excluded | The article was excluded based on title and abstract screening as it did not meet the inclusion and exclusion criteria. |
| NADPH Oxidase Activity in Cerebral Arterioles Is a Key Mediator of Cerebral Small Vessel Disease-Implications for Prevention. | 2015 | McCarty MF | Excluded | The article was excluded based on title and abstract screening as it did not meet the inclusion and exclusion criteria. |
| Uric Acid and Cortisol Levels in Plasma Correlate with Pre-Competition Anxiety in Novice Athletes of Combat Sports. | 2022 | de Oliveira LFG and Souza-Junior TP and Fechio JJ and Gomes-Santos JAF and Sampaio RC and Vardaris CV and Lambertucci RH and de Barros MP | Excluded | The article was excluded based on title and abstract screening as it did not meet the inclusion and exclusion criteria. |
| Effect of Treadmill Exercise and Trans-Cinnamaldehyde against d-Galactose- and Aluminum Chloride-Induced Cognitive Dysfunction in Mice. | 2020 | Ryu JS and Kang HY and Lee JK | Excluded | The article was excluded based on title and abstract screening as it did not meet the inclusion and exclusion criteria. |
| Elevated Lactate by High-Intensity Interval Training Regulates the Hippocampal BDNF Expression and the Mitochondrial Quality Control System. | 2021 | Hu J and Cai M and Shang Q and Li Z and Feng Y and Liu B and Xue X and Lou S | Excluded | The article was excluded based on title and abstract screening as it did not meet the inclusion and exclusion criteria. |
| Role of Lifestyle in Neuroplasticity and Neurogenesis in an Aging Brain. | 2020 | Maharjan R and Diaz Bustamante L and Ghattas KN and Ilyas S and Al-Refai R and Khan S | Excluded | The article was excluded based on title and abstract screening as it did not meet the inclusion and exclusion criteria. |
| Determinants of the maximal functional reserve during repeated supramaximal exercise by humans: The roles of Nrf2/Keap1, antioxidant proteins, muscle phenotype and oxygenation. | 2023 | Galvan-Alvarez V and Martin-Rincon M and Gallego-Selles A and Martínez Canton M and HamedChaman N and Gelabert-Rebato M and Perez-Valera M and García-Gonzalez E and Santana A and Holmberg HC and Boushel R and Hallén J and Calbet JAL | Excluded | The article was excluded based on title and abstract screening as it did not meet the inclusion and exclusion criteria. |
| Improved brain and muscle mitochondrial respiration with CoQ. An in vivo study by 31P-MR spectroscopy in patients with mitochondrial cytopathies. | 1999 | Barbiroli B and Iotti S and Lodi R | Excluded | The article was excluded based on title and abstract screening as it did not meet the inclusion and exclusion criteria. |
| The rate of training response to aerobic exercise affects brain function of rats | 2016 | Marton, O and Koltai, E and Takeda, M and Mimura, T and Pajk, M and Abraham, D and Koch, LG and Britton, SL and Higuchi, M and Boldogh, I and Radak, Z | Included |  |
| RhTFAM treatment stimulates mitochondrial oxidative metabolism and improves memory in aged mice. | 2012 | Thomas RR and Khan SM and Smigrodzki RM and Onyango IG and Dennis J and Khan OM and Portelli FR and Bennett JP Jr | Excluded | The article was excluded based on title and abstract screening as it did not meet the inclusion and exclusion criteria. |
| Developments in Neuroprotection for HIV-Associated Neurocognitive Disorders (HAND). | 2022 | Kolson DL | Excluded | The article was excluded based on title and abstract screening as it did not meet the inclusion and exclusion criteria. |
| Endurance Exercise Prevents Metabolic Distress-induced Senescence in the Hippocampus. | 2019 | Jang Y and Kwon I and Cosio-Lima L and Wirth C and Vinci DM and Lee Y | Excluded | The article was excluded based on title and abstract screening as it did not meet the inclusion and exclusion criteria. |
| Molecular mechanisms in Alzheimer's disease and the impact of physical exercise with advancements in therapeutic approaches. | 2021 | Siddappaji KK and Gopal S | Excluded | The article was excluded based on title and abstract screening as it did not meet the inclusion and exclusion criteria. |
| Regulation of oxidative phosphorylation is different in electrically- and cortically-stimulated skeletal muscle. | 2018 | Korzeniewski B | Excluded | The article was excluded based on title and abstract screening as it did not meet the inclusion and exclusion criteria. |
| Recent advances in the study of the neurobiological mechanisms behind the effects of physical activity on mood, resilience and emotional disorders. | 2023 | Chen C and Nakagawa S | Excluded | The article was excluded based on title and abstract screening as it did not meet the inclusion and exclusion criteria. |
| A self-selected 16:8 time-restricted eating quasi-experimental intervention improves various markers of cardiovascular health in middle-age male cyclists. | 2023 | Waldman HS and Witt CR and Grozier CD and McAllister MJ | Excluded | The article was excluded based on title and abstract screening as it did not meet the inclusion and exclusion criteria. |
| Lipid Peroxidation Markers in Coronary Artery Disease Patients with Possible Vascular Mild Cognitive Impairment. | 2017 | Suridjan I and Herrmann N and Adibfar A and Saleem M and Andreazza A and Oh PI and Lanctôt KL | Excluded | The article was excluded based on title and abstract screening as it did not meet the inclusion and exclusion criteria. |
| Influence of physical exercise on β-amyloid, α-synuclein and tau accumulation: an in vitro model of oxidative stress in human red blood cells. | 2017 | Iofrida C and Daniele S and Pietrobono D and Fusi J and Galetta F and Trincavelli ML and Bonuccelli U and Franzoni F and Martini C | Excluded | The article was excluded based on title and abstract screening as it did not meet the inclusion and exclusion criteria. |
| Impact of adult growth hormone deficiency on metabolic profile and cardiovascular risk [Review]. | 2015 | Giovannini L and Tirabassi G and Muscogiuri G and Di Somma C and Colao A and Balercia G | Excluded | The article was excluded based on title and abstract screening as it did not meet the inclusion and exclusion criteria. |
| Proposed Neuroimmune Roles of Dimethyl Fumarate, Bupropion, S-Adenosylmethionine, and Vitamin D(3) in Affording a Chronically Ill Patient Sustained Relief from Inflammation and Major Depression. | 2020 | Sachinvala ND and Teramoto N and Stergiou A | Excluded | The article was excluded based on title and abstract screening as it did not meet the inclusion and exclusion criteria. |
| Exercise priming with transcranial direct current stimulation: a study protocol for a randomized, parallel-design, sham-controlled trial in mild cognitive impairment and Alzheimer's disease. | 2021 | Liu CS and Herrmann N and Song BX and Ba J and Gallagher D and Oh PI and Marzolini S and Rajji TK and Charles J and Papneja P and Rapoport MJ and Andreazza AC and Vieira D and Kiss A and Lanctôt KL | Excluded | The article was excluded based on title and abstract screening as it did not meet the inclusion and exclusion criteria. |
| The effects of exercise on oxidative stress (TBARS) and BDNF in severely depressed inpatients. | 2014 | Schuch FB and Vasconcelos-Moreno MP and Borowsky C and Zimmermann AB and Wollenhaupt-Aguiar B and Ferrari P and de Almeida Fleck MP | Excluded | The article was excluded based on title and abstract screening as it did not meet the inclusion and exclusion criteria. |
| The Treadmill Exercise Protects against Dopaminergic Neuron Loss and Brain Oxidative Stress in Parkinsonian Rats | 2017 | Da Costa, R.O. and Gadelha-Filho, C.V.J. and Da Costa, A.E.M. and Feitosa, M.L. and De Araújo, D.P. and De Lucena, J.D. and De Aquino, P.E.A. and Lima, F.A.V. and Neves, K.R.T. and De Barros Viana, G.S. | Included |  |
| Cardiorenal metabolic syndrome and diabetic cognopathy. | 2013 | Hayden MR and Banks WA and Shah GN and Gu Z and Sowers JR | Excluded | The article was excluded based on title and abstract screening as it did not meet the inclusion and exclusion criteria. |
| Optimized Curcumin, Pomegranate Extract, and Methylsulfonylmethane Reduce Acute, Systemic Inflammatory Response to a Half-marathon Race. | 2022 | Tanner EA and Gary MA and Michalik S and Davis AA and McFarlin BK | Excluded | The article was excluded based on title and abstract screening as it did not meet the inclusion and exclusion criteria. |
| Exercise-induced BDNF production by mononuclear cells of sedentary and physically active obese men. | 2020 | Elsner VR and Dorneles GP and Santos MA and da Silva IM and Romão P and Peres A | Excluded | The article was excluded based on title and abstract screening as it did not meet the inclusion and exclusion criteria. |
| Fuel economy in food-deprived skeletal muscle: signaling pathways and regulatory mechanisms. | 2007 | de Lange P and Moreno M and Silvestri E and Lombardi A and Goglia F and Lanni A | Excluded | The article was excluded based on title and abstract screening as it did not meet the inclusion and exclusion criteria. |
| Interventions on Gut Microbiota for Healthy Aging. | 2022 | Donati Zeppa S and Agostini D and Ferrini F and Gervasi M and Barbieri E and Bartolacci A and Piccoli G and Saltarelli R and Sestili P and Stocchi V | Excluded | The article was excluded based on title and abstract screening as it did not meet the inclusion and exclusion criteria. |
| Fluctuating hearing loss, episodic headache, and stroke with platelet hyperaggregability: coexistence of auditory neuropathy and cochlear hearing loss. | 2006 | Nobutoki T and Sasaki M and Fukumizu M and Hanaoka S and Sugai K and Anzai Y and Kaga M | Excluded | The article was excluded based on title and abstract screening as it did not meet the inclusion and exclusion criteria. |
| The effect of 24-hour sleep deprivation and anaerobic exercise on the expression of BAX, BCL2, BMAL1 and CCAR2 genes in peripheral blood mononuclear cells after L-arginine supplementation. | 2023 | Norouzi Kamareh M and Samadi M and Arabzadeh E and Abdollahi M and Sheidaei S and Riyahi Malayeri S and Schlicht J and Shirvani H and Rostamkhani F | Excluded | The article was excluded based on title and abstract screening as it did not meet the inclusion and exclusion criteria. |
| Moderate Exercise Prevents Functional Remodeling of the Anterior Pituitary Gland in Diet-Induced Insulin Resistance in Rats: Role of Oxidative Stress and Autophagy. | 2016 | Mercau ME and Repetto EM and Perez MN and Martinez Calejman C and Sanchez Puch S and Finkielstein CV and Cymeryng CB | Excluded | The article was excluded based on title and abstract screening as it did not meet the inclusion and exclusion criteria. |
| Endonuclease VIII-like 3 (Neil3) DNA glycosylase promotes neurogenesis induced by hypoxia-ischemia. | 2011 | Sejersted Y and Hildrestrand GA and Kunke D and Rolseth V and Krokeide SZ and Neurauter CG and Suganthan R and Atneosen-Åsegg M and Fleming AM and Saugstad OD and Burrows CJ and Luna L and Bjørås M | Excluded | The article was excluded based on title and abstract screening as it did not meet the inclusion and exclusion criteria. |
| The tortuous path of lactate shuttle discovery: From cinders and boards to the lab and ICU. | 2020 | Brooks GA | Excluded | The article was excluded based on title and abstract screening as it did not meet the inclusion and exclusion criteria. |
| Neuroimmunological effects of physical exercise in depression. | 2012 | Eyre H and Baune BT | Excluded | The article was excluded based on title and abstract screening as it did not meet the inclusion and exclusion criteria. |
| Effect of breathing exercises on oxidative stress biomarkers in humans: A systematic review and meta-analysis. | 2023 | Li TT and Wang HY and Zhang H and Zhang PP and Zhang MC and Feng HY and Duan XY and Liu WB and Wang XW and Sun ZG | Excluded | The article was excluded based on title and abstract screening as it did not meet the inclusion and exclusion criteria. |
| Moving to Improve Mental Health - The Role of Exercise in Cognitive Function: A Narrative Review. | 2024 | Pujari V | Excluded | The article was excluded based on title and abstract screening as it did not meet the inclusion and exclusion criteria. |
| Effects of treadmill exercise on the regulatory mechanisms of mitochondrial dynamics and oxidative stress in the brains of high-fat diet fed rats. | 2019 | Koo JH and Kang EB | Excluded | The article was excluded based on title and abstract screening as it did not meet the inclusion and exclusion criteria. |
| Aging and Neurodegeneration: A Tangle of Models and Mechanisms. | 2016 | Chakrabarti S and Mohanakumar KP | Excluded | The article was excluded based on title and abstract screening as it did not meet the inclusion and exclusion criteria. |
| Therapeutic strategies in Friedreich's ataxia. | 2004 | Schöls L and Meyer Ch and Schmid G and Wilhelms I and Przuntek H | Excluded | The article was excluded based on title and abstract screening as it did not meet the inclusion and exclusion criteria. |
| The impact of an ultra-trail on the dynamic of cardiac, inflammatory, renal and oxidative stress biological markers correlated with electrocardiogram and echocardiogram. | 2021 | Le Goff C and Kaux JF and Dulgheru R and Seidel L and Pincemail J and Cavalier E and Melon P | Excluded | The article was excluded based on title and abstract screening as it did not meet the inclusion and exclusion criteria. |
| Possible causes of Parkinson's disease. | 2021 | Adams JD | Excluded | The article was excluded based on title and abstract screening as it did not meet the inclusion and exclusion criteria. |
| Prevention of progression in Parkinson's disease. | 2018 | Aaseth J and Dusek P and Roos PM | Excluded | The article was excluded based on title and abstract screening as it did not meet the inclusion and exclusion criteria. |
| Biomarkers of exercise-induced myocardial stress in relation to inflammatory and oxidative stress. | 2007 | König D and Neubauer O and Nics L and Kern N and Berg A and Bisse E and Wagner KH | Excluded | The article was excluded based on title and abstract screening as it did not meet the inclusion and exclusion criteria. |
| The effects of carbohydrate supplementation during the second of two prolonged cycling bouts on immunoendocrine responses. | 2005 | Li TL and Gleeson M | Excluded | The article was excluded based on title and abstract screening as it did not meet the inclusion and exclusion criteria. |
| Children with Autism Spectrum Disorder Exhibit Elevated Physical Activity and Reduced Sedentary Behavior. | 2023 | Alhowikan AM and Elamin NE and Aldayel SS and AlSiddiqi SA and Alrowais FS and Hassan WM and El-Ansary A and Alghamdi FA and Al-Ayadhi LY | Excluded | The article was excluded based on title and abstract screening as it did not meet the inclusion and exclusion criteria. |
| A neural signaling triumvirate that influences ageing and age-related disease: insulin/IGF-1, BDNF and serotonin. | 2004 | Mattson MP and Maudsley S and Martin B | Excluded | The article was excluded based on title and abstract screening as it did not meet the inclusion and exclusion criteria. |
| Glutathione Peroxidase Activity Is Altered in Vascular Cognitive Impairment-No Dementia and Is a Potential Marker for Verbal Memory Performance. | 2021 | Ahmed M and Herrmann N and Chen JJ and Saleem M and Oh PI and Andreazza AC and Kiss A and Lanctôt KL | Excluded | The article was excluded based on title and abstract screening as it did not meet the inclusion and exclusion criteria. |
| Move Your Body toward Healthy Aging: Potential Neuroprotective Mechanisms of Irisin in Alzheimer's Disease. | 2023 | Bellettini-Santos T and Batista-Silva H and Marcolongo-Pereira C and Quintela-Castro FCA and Barcelos RM and Chiepe KCMB and Rossoni JV Jr and Passamani-Ambrosio R and da Silva BS and Chiarelli-Neto O and Garcez ML | Excluded | The article was excluded based on title and abstract screening as it did not meet the inclusion and exclusion criteria. |
| Mitochondrial P2X7 Receptor Localization Modulates Energy Metabolism Enhancing Physical Performance. | 2021 | Sarti AC and Vultaggio-Poma V and Falzoni S and Missiroli S and Giuliani AL and Boldrini P and Bonora M and Faita F and Di Lascio N and Kusmic C and Solini A and Novello S and Morari M and Rossato M and Wieckowski MR and Giorgi C and Pinton P and Di Virgilio F | Excluded | The article was excluded based on title and abstract screening as it did not meet the inclusion and exclusion criteria. |
| Treadmill exercise protects against pentylenetetrazol-induced seizures and oxidative stress after traumatic brain injury | 2013 | Silva, L.F.A. and Hoffmann, M.S. and Gerbatin, R.D.R. and Fiorin, F.D.S. and Dobrachinski, F. and Mota, B.C. and Wouters, A.T.B. and Pavarini, S.P. and Soares, F.A.A. and Fighera, M.R. and Royes, L.F.F. | Included |  |
| Neuroprotective Effect of Schisandra Chinensis on Methyl-4-Phenyl-1,2,3,6-Tetrahydropyridine-Induced Parkinsonian Syndrome in C57BL/6 Mice. | 2019 | Li CL and Tsuang YH and Tsai TH | Excluded | The article was excluded based on title and abstract screening as it did not meet the inclusion and exclusion criteria. |
| Nitric Oxide Pathways in Neurovascular Coupling Under Normal and Stress Conditions in the Brain: Strategies to Rescue Aberrant Coupling and Improve Cerebral Blood Flow. | 2021 | Lourenço CF and Laranjinha J | Excluded | The article was excluded based on title and abstract screening as it did not meet the inclusion and exclusion criteria. |
| A Role for Advanced Glycation End Products in Molecular Ageing. | 2023 | Zgutka K and Tkacz M and Tomasiak P and Tarnowski M | Excluded | The article was excluded based on title and abstract screening as it did not meet the inclusion and exclusion criteria. |
| Gut Microbiota, Nitric Oxide, and Microglia as Prerequisites for Neurodegenerative Disorders. | 2017 | Tse JKY | Excluded | The article was excluded based on title and abstract screening as it did not meet the inclusion and exclusion criteria. |
| Metabolic syndrome induced by anticancer treatment in childhood cancer survivors. | 2017 | Chueh HW and Yoo JH | Excluded | The article was excluded based on title and abstract screening as it did not meet the inclusion and exclusion criteria. |
| Dissociated coupling between cerebral oxygen metabolism and perfusion in the prefrontal cortex during exercise: a NIRS study. | 2023 | Hiura M and Funaki A and Shibutani H and Takahashi K and Katayama Y | Excluded | The article was excluded based on title and abstract screening as it did not meet the inclusion and exclusion criteria. |
| Effects of increased daily physical activity on mental health and depression biomarkers in postmenopausal women. | 2019 | Takahashi M and Lim PJ and Tsubosaka M and Kim HK and Miyashita M and Suzuki K and Tan EL and Shibata S | Excluded | The article was excluded based on title and abstract screening as it did not meet the inclusion and exclusion criteria. |
| Weight-loss-independent benefits of exercise on liver steatosis and stiffness in Japanese men with NAFLD. | 2021 | Oh S and Tsujimoto T and Kim B and Uchida F and Suzuki H and Iizumi S and Isobe T and Sakae T and Tanaka K and Shoda J | Excluded | The article was excluded based on title and abstract screening as it did not meet the inclusion and exclusion criteria. |
| Functional impairment of skeletal muscle oxidative metabolism during knee extension exercise after bed rest. | 2011 | Salvadego D and Lazzer S and Marzorati M and Porcelli S and Rejc E and Simunic B and Pisot R and di Prampero PE and Grassi B | Excluded | The article was excluded based on title and abstract screening as it did not meet the inclusion and exclusion criteria. |
| Examining the Mechanisms behind Exercise's Multifaceted Impacts on Body Composition, Cognition, and the Gut Microbiome in Cancer Survivors: Exploring the Links to Oxidative Stress and Inflammation. | 2023 | Matei B and Winters-Stone KM and Raber J | Excluded | The article was excluded based on title and abstract screening as it did not meet the inclusion and exclusion criteria. |
| What and How Can Physical Activity Prevention Function on Parkinson's Disease? | 2020 | Fan B and Jabeen R and Bo B and Guo C and Han M and Zhang H and Cen J and Ji X and Wei J | Excluded | The article was excluded based on title and abstract screening as it did not meet the inclusion and exclusion criteria. |
| Enriched environment inhibits mouse pancreatic cancer growth and down-regulates the expression of mitochondria-related genes in cancer cells. | 2015 | Li G and Gan Y and Fan Y and Wu Y and Lin H and Song Y and Cai X and Yu X and Pan W and Yao M and Gu J and Tu H | Excluded | The article was excluded based on title and abstract screening as it did not meet the inclusion and exclusion criteria. |
| [Physical activity in the elderly with other major neurocognitive disorders]. | 2022 | Manckoundia P and Dipanda M and Mourey F | Excluded | The article was excluded based on title and abstract screening as it did not meet the inclusion and exclusion criteria. |
| Mobilising patients with severe acquired brain injury in intensive care (MAWERIC) - Protocol for a randomised cross-over trial. | 2022 | Riberholt CG and Olsen MH and Berg RMG and Møller K | Excluded | The article was excluded based on title and abstract screening as it did not meet the inclusion and exclusion criteria. |
| Concussion history in rugby union players is associated with depressed cerebrovascular reactivity and cognition. | 2021 | Owens TS and Calverley TA and Stacey BS and Rose G and Fall L and Tsukamoto H and Jones G and Corkill R and Tuaillon E and Hirtz C and Lehmann S and Marchi N and Marley CJ and Bailey DM | Excluded | The article was excluded based on title and abstract screening as it did not meet the inclusion and exclusion criteria. |
| Addition of candesartan to angiotensin converting enzyme inhibitor therapy in patients with chronic heart failure does not reduce levels of oxidative stress. | 2002 | Ellis GR and Nightingale AK and Blackman DJ and Anderson RA and Mumford C and Timmins G and Lang D and Jackson SK and Penney MD and Lewis MJ and Frenneaux MP and Morris-Thurgood J | Excluded | The article was excluded based on title and abstract screening as it did not meet the inclusion and exclusion criteria. |
| Corrigendum to "The Treadmill Exercise Protects against Dopaminergic Neuron Loss and Brain Oxidative Stress in Parkinsonian Rats". | 2018 | da Costa RO and Gadelha-Filho CVJ and da Costa AEM and Feitosa ML and de Araújo DP and de Lucena JD and de Aquino PEA and Lima FAV and Neves KRT and de Barros Viana GS | Excluded | The article was excluded based on title and abstract screening as it did not meet the inclusion and exclusion criteria. |
| Chronic 5-Aminoimidazole-4-Carboxamide-1-β-d-Ribofuranoside Treatment Induces Phenotypic Changes in Skeletal Muscle, but Does Not Improve Disease Outcomes in the R6/2 Mouse Model of Huntington's Disease. | 2017 | Paré MF and Jasmin BJ | Excluded | The article was excluded based on title and abstract screening as it did not meet the inclusion and exclusion criteria. |
| Evolutionary aspects of human exercise--born to run purposefully. | 2012 | Mattson MP | Excluded | The article was excluded based on title and abstract screening as it did not meet the inclusion and exclusion criteria. |
| The APEX trial: Effects of allopurinol on exercise capacity, coronary and peripheral endothelial function, and natriuretic peptides in patients with cardiac syndrome X. | 2018 | Lim TK and Noman A and Choy AMJ and Khan F and Struthers AD and Lang CC | Excluded | The article was excluded based on title and abstract screening as it did not meet the inclusion and exclusion criteria. |
| Different patterns of chronic hypoxia lead to hierarchical adaptive mechanisms in goldfish metabolism. | 2022 | Thoral E and Farhat E and Roussel D and Cheng H and Guillard L and Pamenter ME and Weber JM and Teulier L | Excluded | The article was excluded based on title and abstract screening as it did not meet the inclusion and exclusion criteria. |
| FoxO6 and PGC-1α form a regulatory loop in myogenic cells. | 2013 | Chung SY and Huang WC and Su CW and Lee KW and Chi HC and Lin CT and Chen ST and Huang KM and Tsai MS and Yu HP and Chen SL | Excluded | The article was excluded based on title and abstract screening as it did not meet the inclusion and exclusion criteria. |
| Roles of the lipid peroxidation product 4-hydroxynonenal in obesity, the metabolic syndrome, and associated vascular and neurodegenerative disorders. | 2009 | Mattson MP | Excluded | The article was excluded based on title and abstract screening as it did not meet the inclusion and exclusion criteria. |
| Pathophysiological Mechanisms Explaining the Association Between Low Skeletal Muscle Mass and Cognitive Function. | 2022 | Oudbier SJ and Goh J and Looijaard SMLM and Reijnierse EM and Meskers CGM and Maier AB | Excluded | The article was excluded based on title and abstract screening as it did not meet the inclusion and exclusion criteria. |
| Exacerbation of Brain Injury by Post-Stroke Exercise Is Contingent Upon Exercise Initiation Timing. | 2017 | Li F and Geng X and Khan H and Pendy JT Jr and Peng C and Li X and Rafols JA and Ding Y | Excluded | The article was excluded based on title and abstract screening as it did not meet the inclusion and exclusion criteria. |
| Alzheimer's Disease: An Exacerbation of Senile Phenoptosis. | 2015 | Isaev NK and Stelmashook EV and Genrikhs EE and Oborina MV and Kapkaeva MR and Skulachev VP | Excluded | The article was excluded based on title and abstract screening as it did not meet the inclusion and exclusion criteria. |
| Research on the Oxidative Stress Response of Human Body Caused by Different Nutritional Supplements and the Improvement Effect of Exercise. | 2022 | Zhang S | Excluded | The article was excluded based on title and abstract screening as it did not meet the inclusion and exclusion criteria. |
| Why does the Y326I mutant of monoamine oxidase B decompose an endogenous amphetamine at a slower rate than the wild type enzyme? Reaction step elucidated by multiscale molecular simulations. | 2018 | Pregeljc D and Jug U and Mavri J and Stare J | Excluded | The article was excluded based on title and abstract screening as it did not meet the inclusion and exclusion criteria. |
| Effects of bovine colostrum supplementation on upper respiratory illness in active males. | 2014 | Jones AW and Cameron SJ and Thatcher R and Beecroft MS and Mur LA and Davison G | Excluded | The article was excluded based on title and abstract screening as it did not meet the inclusion and exclusion criteria. |
| The Interrelated Multifactorial Actions of Cortisol and Klotho: Potential Implications in the Pathogenesis of Parkinson's Disease. | 2022 | Luthra NS and Clow A and Corcos DM | Excluded | The article was excluded based on title and abstract screening as it did not meet the inclusion and exclusion criteria. |
| Severe physical exertion, oxidative stress, and acute lung injury. | 2011 | Shah NR and Iqbal MB and Barlow A and Bayliss J | Excluded | The article was excluded based on title and abstract screening as it did not meet the inclusion and exclusion criteria. |
| Effects of the FNDC5/Irisin on Elderly Dementia and Cognitive Impairment. | 2022 | Peng J and Wu J | Excluded | The article was excluded based on title and abstract screening as it did not meet the inclusion and exclusion criteria. |
| L-carnitine and Acetyl-L Carnitine: A Possibility for Treating Alterations Induced by Obesity in the Central Nervous System. | 2023 | da Silva LE and de Oliveira MP and da Silva MR and Abel JDS and Tartari G and de Aguiar da Costa M and Ludvig Gonçalves C and Rezin GT | Excluded | The article was excluded based on title and abstract screening as it did not meet the inclusion and exclusion criteria. |
| Habitual physical activity is associated with the maintenance of neutrophil migratory dynamics in healthy older adults. | 2016 | Bartlett DB and Fox O and McNulty CL and Greenwood HL and Murphy L and Sapey E and Goodman M and Crabtree N and Thøgersen-Ntoumani C and Fisher JP and Wagenmakers AJ and Lord JM | Excluded | The article was excluded based on title and abstract screening as it did not meet the inclusion and exclusion criteria. |
| The spectrum of exercise tolerance in mitochondrial myopathies: a study of 40 patients. | 2003 | Taivassalo T and Jensen TD and Kennaway N and DiMauro S and Vissing J and Haller RG | Excluded | The article was excluded based on title and abstract screening as it did not meet the inclusion and exclusion criteria. |
| [Effects of exhaustive exercise on the expression of PHB1 and the function of mitochondria in rats]. | 2017 | Fang W and Li Z and Liu XH and Liu ZM and Dun Y and Feng H | Excluded | The article was excluded based on title and abstract screening as it did not meet the inclusion and exclusion criteria. |
| Neuroprotective Effects of Forced Exercise and Bupropion on Chronic Methamphetamine-induced Cognitive Impairment via Modulation of cAMP Response Element-binding Protein/Brain-derived Neurotrophic Factor Signaling Pathway, Oxidative Stress, and Inflammatory Biomarkers in Rats. | 2018 | Taheri P and Keshavarzi S and Ebadi M and Motaghinejad M and Motevalian M | Excluded | The article was excluded based on title and abstract screening as it did not meet the inclusion and exclusion criteria. |
| Exhausting exercise and tissue-specific expression of monocarboxylate transporters in rainbow trout. | 2013 | Omlin T and Weber JM | Excluded | The article was excluded based on title and abstract screening as it did not meet the inclusion and exclusion criteria. |
| Environment and Gene Association With Obesity and Their Impact on Neurodegenerative and Neurodevelopmental Diseases. | 2020 | Flores-Dorantes MT and Díaz-López YE and Gutiérrez-Aguilar R | Excluded | The article was excluded based on title and abstract screening as it did not meet the inclusion and exclusion criteria. |
| Mitochondrial reactive oxygen species generation in blood cells is associated with disease severity and exercise intolerance in heart failure patients. | 2019 | Shirakawa R and Yokota T and Nakajima T and Takada S and Yamane M and Furihata T and Maekawa S and Nambu H and Katayama T and Fukushima A and Saito A and Ishimori N and Dela F and Kinugawa S and Anzai T | Excluded | The article was excluded based on title and abstract screening as it did not meet the inclusion and exclusion criteria. |
| Protective Effects of Whey Protein Hydrolysate, Treadmill Exercise, and Their Combination against Scopolamine-Induced Cognitive Deficit in Mice. | 2023 | Chang YB and Jung EJ and Suh HJ and Choi HS | Excluded | The article was excluded based on title and abstract screening as it did not meet the inclusion and exclusion criteria. |
| Splice mutations preserve myophosphorylase activity that ameliorates the phenotype in McArdle disease. | 2009 | Vissing J and Duno M and Schwartz M and Haller RG | Excluded | The article was excluded based on title and abstract screening as it did not meet the inclusion and exclusion criteria. |
| Obesity and Oxidative Stress in Older Adults At Risk for Dementia: A Magnetic Resonance Spectroscopy Study. | 2021 | Turner A and Hoyos C and Mowszowski L and LaMonica H and Lagopoulos J and DeMayo MM and Ireland C and Hickie IB and Naismith SL and Duffy SL | Excluded | The article was excluded based on title and abstract screening as it did not meet the inclusion and exclusion criteria. |
| Coenzyme Q10 Levels Associated With Cognitive Functioning and Executive Function in Older Adults. | 2023 | Fernández-Portero C and Amián JG and Bella R and López-Lluch G and Alarcón D | Excluded | The article was excluded based on title and abstract screening as it did not meet the inclusion and exclusion criteria. |
| Epicatechin Plus Treadmill Exercise are Neuroprotective Against Moderate-stage Amyloid Precursor Protein/Presenilin 1 Mice. | 2016 | Zhang Z and Wu H and Huang H | Excluded | The article was excluded based on title and abstract screening as it did not meet the inclusion and exclusion criteria. |
| Underlying inflammation has no impact on the oxidative stress response to acute mental stress. | 2014 | Wadley AJ and Veldhuijzen van Zanten JJ and Paine NJ and Drayson MT and Aldred S | Excluded | The article was excluded based on title and abstract screening as it did not meet the inclusion and exclusion criteria. |
| A combination of soy isoflavone supplementation and exercise improves lipid profiles and protects antioxidant defense-systems against exercise-induced oxidative stress in ovariectomized rats. | 2007 | Oh HY and Lim S and Lee JM and Kim DY and Ann ES and Yoon S | Excluded | The article was excluded based on title and abstract screening as it did not meet the inclusion and exclusion criteria. |
| Lactate: link between glycolytic and oxidative metabolism. | 2007 | Brooks GA | Excluded | The article was excluded based on title and abstract screening as it did not meet the inclusion and exclusion criteria. |
| Lactate as a supplemental fuel for synaptic transmission and neuronal network oscillations: Potentials and limitations. | 2023 | Kann O | Excluded | The article was excluded based on title and abstract screening as it did not meet the inclusion and exclusion criteria. |
| The effect of stress on the antioxidative potential of serum: implications for Alzheimer's disease. | 2006 | van Rensburg SJ and van Zyl JM and Potocnik FC and Daniels WM and Uys J and Marais L and Hon D and van der Walt BJ and Erasmus RT | Excluded | The article was excluded based on title and abstract screening as it did not meet the inclusion and exclusion criteria. |
| Increased capillaries in mitochondrial myopathy: implications for the regulation of oxygen delivery. | 2012 | Taivassalo T and Ayyad K and Haller RG | Excluded | The article was excluded based on title and abstract screening as it did not meet the inclusion and exclusion criteria. |
| Effects of traditional Chinese exercise on vascular function in patients with Alzheimer's disease: A protocol for systematic review and network meta-analysis of randomized controlled trials. | 2023 | Li J and Wang C and Zhang P | Excluded | The article was excluded based on title and abstract screening as it did not meet the inclusion and exclusion criteria. |
| TBHQ-Overview of Multiple Mechanisms against Oxidative Stress for Attenuating Methamphetamine-Induced Neurotoxicity. | 2020 | Zhao YL and Zhao W and Liu M and Liu L and Wang Y | Excluded | The article was excluded based on title and abstract screening as it did not meet the inclusion and exclusion criteria. |
| Diffuse correlation spectroscopy and frequency-domain near-infrared spectroscopy for measuring microvascular blood flow in dynamically exercising human muscles. | 2019 | Quaresima V and Farzam P and Anderson P and Farzam PY and Wiese D and Carp SA and Ferrari M and Franceschini MA | Excluded | The article was excluded based on title and abstract screening as it did not meet the inclusion and exclusion criteria. |
| Comparison of selected prooxidant-antioxidant balance and bone metabolism indicators and BDNF levels between older women with different levels of physical activity. | 2023 | Sadowska-Krępa E and Rzetecki A and Zając-Gawlak I and Nawrat-Szołtysik A and Rozpara M and Mikuľáková W and Stanek A and Pałka T | Excluded | The article was excluded based on title and abstract screening as it did not meet the inclusion and exclusion criteria. |
| Exercise-induced angiogenesis is dependent on metabolically primed ATF3/4(+) endothelial cells. | 2021 | Fan Z and Turiel G and Ardicoglu R and Ghobrial M and Masschelein E and Kocijan T and Zhang J and Tan G and Fitzgerald G and Gorski T and Alvarado-Diaz A and Gilardoni P and Adams CM and Ghesquière B and De Bock K | Excluded | The article was excluded based on title and abstract screening as it did not meet the inclusion and exclusion criteria. |
| An extract of Lycium barbarum mimics exercise to improve muscle endurance through increasing type IIa oxidative muscle fibers by activating ERRγ. | 2020 | Meng J and Lv Z and Sun C and Qiao X and Chen C | Excluded | The article was excluded based on title and abstract screening as it did not meet the inclusion and exclusion criteria. |
| Blood Flow during Handgrip Exercise in COPD: Effect of Vitamin C. | 2016 | Hartmann SE and Waltz X and Leigh R and Anderson TJ and Poulin MJ | Excluded | The article was excluded based on title and abstract screening as it did not meet the inclusion and exclusion criteria. |
| A split-label design for simultaneous measurements of perfusion in distant slices by pulsed arterial spin labeling. | 2021 | Baligand C and Hirschler L and Veeger TTJ and Václavů L and Franklin SL and van Osch MJP and Kan HE | Excluded | The article was excluded based on title and abstract screening as it did not meet the inclusion and exclusion criteria. |
| Effects of dietary iron deficiency on muscle fiber characteristics and whole-body distribution of hemoglobin in mice. | 1983 | Ohira Y and Gill SL | Excluded | The article was excluded based on title and abstract screening as it did not meet the inclusion and exclusion criteria. |
| The possible contribution of angiitis to the onset of benign paroxysmal positional vertigo (BPPV). | 2010 | Goto F and Hayashi K and Kunihiro T and Ogawa K | Excluded | The article was excluded based on title and abstract screening as it did not meet the inclusion and exclusion criteria. |
| Manganese superoxide dismutase and oxidative stress modulation. | 2015 | Bresciani G and da Cruz IB and González-Gallego J | Excluded | The article was excluded based on title and abstract screening as it did not meet the inclusion and exclusion criteria. |
| Endocannabinoids and aging-Inflammation, neuroplasticity, mood and pain. | 2021 | Park Y and Watkins BA | Excluded | The article was excluded based on title and abstract screening as it did not meet the inclusion and exclusion criteria. |
| The effects of probiotic supplements on oxidative stress and inflammation in subjects with mild and moderate Alzheimer's disease: a randomized, double-blind, placebo-controlled study. | 2024 | Akhgarjand C and Vahabi Z and Shab-Bidar S and Anoushirvani A and Djafarian K | Excluded | The article was excluded based on title and abstract screening as it did not meet the inclusion and exclusion criteria. |
| Ketone Bodies in the Brain Beyond Fuel Metabolism: From Excitability to Gene Expression and Cell Signaling. | 2021 | García-Rodríguez D and Giménez-Cassina A | Excluded | The article was excluded based on title and abstract screening as it did not meet the inclusion and exclusion criteria. |
| Functional evaluation techniques in mitochondrial disorders. | 1998 | Argov Z | Excluded | The article was excluded based on title and abstract screening as it did not meet the inclusion and exclusion criteria. |
| Can serum 8-hydroxy-2'-deoxyguanosine levels reflect the severity of pulmonary arterial hypertension? | 2021 | Coskun FY and Taysı S and Kayıkçıoğlu M | Excluded | The article was excluded based on title and abstract screening as it did not meet the inclusion and exclusion criteria. |
| Long-term coenzyme Q10 therapy for a mitochondrial encephalomyopathy with cytochrome c oxidase deficiency: a 31P NMR study. | 1989 | Nishikawa Y and Takahashi M and Yorifuji S and Nakamura Y and Ueno S and Tarui S and Kozuka T and Nishimura T | Excluded | The article was excluded based on title and abstract screening as it did not meet the inclusion and exclusion criteria. |
| Muscle high-energy phosphates in central nervous system disorders. The phosphorus MRS experience. | 1997 | Argov Z and De Stefano N and Arnold DL | Excluded | The article was excluded based on title and abstract screening as it did not meet the inclusion and exclusion criteria. |
| The Relationship between Oxidative Stress and Subjective Sleep Quality in People with Coronary Artery Disease. | 2022 | Feng V and Tumati S and Wang R and Bawa KK and Gallagher D and Herrmann N and Marzolini S and Oh P and Andreazza A and Lanctôt KL | Excluded | The article was excluded based on title and abstract screening as it did not meet the inclusion and exclusion criteria. |
| [Cardiovascular aging]. | 2008 | Domenech RJ and Macho P | Excluded | The article was excluded based on title and abstract screening as it did not meet the inclusion and exclusion criteria. |
| Changes in cardiopulmonary reserve and peripheral arterial function concomitantly with subclinical inflammation and oxidative stress in patients with heart failure with preserved ejection fraction. | 2014 | Vitiello D and Harel F and Touyz RM and Sirois MG and Lavoie J and Myers J and Ducharme A and Racine N and O'Meara E and Gayda M and Chabot-Blanchet M and Rouleau JL and de Denus S and White M | Excluded | The article was excluded based on title and abstract screening as it did not meet the inclusion and exclusion criteria. |
| Cardiac and skeletal muscle adaptations to voluntary wheel running in the mouse. | 2001 | Allen DL and Harrison BC and Maass A and Bell ML and Byrnes WC and Leinwand LA | Excluded | The article was excluded based on title and abstract screening as it did not meet the inclusion and exclusion criteria. |
| Peripheral-specific y2 receptor knockdown protects mice from high-fat diet-induced obesity. | 2011 | Shi YC and Lin S and Castillo L and Aljanova A and Enriquez RF and Nguyen AD and Baldock PA and Zhang L and Bijker MS and Macia L and Yulyaningsih E and Zhang H and Lau J and Sainsbury A and Herzog H | Excluded | The article was excluded based on title and abstract screening as it did not meet the inclusion and exclusion criteria. |
| Osteoarthritis, cerebrovascular dysfunction and the common denominator of inflammation: a narrative review. | 2018 | Al-Khazraji BK and Appleton CT and Beier F and Birmingham TB and Shoemaker JK | Excluded | The article was excluded based on title and abstract screening as it did not meet the inclusion and exclusion criteria. |
| Quantitation of NAD+: Why do we need to measure it? | 2018 | Liu Y and Clement J and Grant R and Sachdev P and Braidy N | Excluded | The article was excluded based on title and abstract screening as it did not meet the inclusion and exclusion criteria. |
| Work at high altitude and oxidative stress: antioxidant nutrients. | 2002 | Askew EW | Excluded | The article was excluded based on title and abstract screening as it did not meet the inclusion and exclusion criteria. |
| Butylphthalide soft capsules combined with modified tonic exercise therapy on neurological function and ability of daily living of patients with stroke hemiplegia. | 2021 | Lv Y and Zhang Q and Rong L and Wei X and Liu H and Li Z | Excluded | The article was excluded based on title and abstract screening as it did not meet the inclusion and exclusion criteria. |
| Lifestyle-induced metabolic inflexibility and accelerated ageing syndrome: insulin resistance, friend or foe? | 2009 | Nunn AV and Bell JD and Guy GW | Excluded | The article was excluded based on title and abstract screening as it did not meet the inclusion and exclusion criteria. |
| Weight loss and Alzheimer's disease: temporal and aetiologic connections. | 2013 | Sergi G and De Rui M and Coin A and Inelmen EM and Manzato E | Excluded | The article was excluded based on title and abstract screening as it did not meet the inclusion and exclusion criteria. |
| Tryptophan Metabolism and Related Pathways in Psychoneuroimmunology: The Impact of Nutrition and Lifestyle. | 2020 | Gostner JM and Geisler S and Stonig M and Mair L and Sperner-Unterweger B and Fuchs D | Excluded | The article was excluded based on title and abstract screening as it did not meet the inclusion and exclusion criteria. |
| Normal in vivo skeletal muscle oxidative metabolism in sporadic inclusion body myositis assessed by 31P-magnetic resonance spectroscopy. | 1998 | Lodi R and Taylor DJ and Tabrizi SJ and Hilton-Jones D and Squier MV and Seller A and Styles P and Schapira AH | Excluded | The article was excluded based on title and abstract screening as it did not meet the inclusion and exclusion criteria. |
| Development and validation of a macroarray system--MutaCHIP ARTERO--for the detection of genetic variants involved in the pathogenesis of atherosclerosis. | 2014 | Chaykovska L and Zientara A and Reser D and Weise A and Reichert W and Hocher B | Excluded | The article was excluded based on title and abstract screening as it did not meet the inclusion and exclusion criteria. |
| Diagnostic and prognostic value of urine biomarkers among women with dysfunctional voiding. | 2022 | Jiang YH and Jhang JF and Ho HC and Hsu YH and Kuo HC | Excluded | The article was excluded based on title and abstract screening as it did not meet the inclusion and exclusion criteria. |
| MicroRNAs as Critical Biomarkers of Major Depressive Disorder: A Comprehensive Perspective. | 2021 | Ortega MA and Alvarez-Mon MA and García-Montero C and Fraile-Martinez O and Lahera G and Monserrat J and Muñoz-Merida L and Mora F and Rodríguez-Jiménez R and Fernandez-Rojo S and Quintero J and Álvarez-Mon M | Excluded | The article was excluded based on title and abstract screening as it did not meet the inclusion and exclusion criteria. |
| Mouse thymocyte apoptosis and cell loss in response to exercise and antioxidant administration. | 2005 | Quadrilatero J and Hoffman-Goetz L | Excluded | The article was excluded based on title and abstract screening as it did not meet the inclusion and exclusion criteria. |
| Riboflavin-responsive oxidative phosphorylation complex I deficiency caused by defective ACAD9: new function for an old gene. | 2011 | Gerards M and van den Bosch BJ and Danhauser K and Serre V and van Weeghel M and Wanders RJ and Nicolaes GA and Sluiter W and Schoonderwoerd K and Scholte HR and Prokisch H and Rötig A and de Coo IF and Smeets HJ | Excluded | The article was excluded based on title and abstract screening as it did not meet the inclusion and exclusion criteria. |
| Demonstration of a day-night rhythm in human skeletal muscle oxidative capacity. | 2016 | van Moorsel D and Hansen J and Havekes B and Scheer FAJL and Jörgensen JA and Hoeks J and Schrauwen-Hinderling VB and Duez H and Lefebvre P and Schaper NC and Hesselink MKC and Staels B and Schrauwen P | Excluded | The article was excluded based on title and abstract screening as it did not meet the inclusion and exclusion criteria. |
| Biomarkers for Detecting Mitochondrial Disorders. | 2018 | Finsterer J and Zarrouk-Mahjoub S | Excluded | The article was excluded based on title and abstract screening as it did not meet the inclusion and exclusion criteria. |
| The Preventive Effects of Pterostilbene on the Exercise Intolerance and Circadian Misalignment of Mice Subjected to Sleep Restriction. | 2020 | Liu Y and Lang H and Zhou M and Huang L and Hui S and Wang X and Chen K and Mi M | Excluded | The article was excluded based on title and abstract screening as it did not meet the inclusion and exclusion criteria. |
| A Promising Strategy to Treat Neurodegenerative Diseases by SIRT3 Activation. | 2023 | Tyagi A and Pugazhenthi S | Excluded | The article was excluded based on title and abstract screening as it did not meet the inclusion and exclusion criteria. |
| Glutamate and neurotrophic factors in neuronal plasticity and disease. | 2008 | Mattson MP | Excluded | The article was excluded based on title and abstract screening as it did not meet the inclusion and exclusion criteria. |
| Fish oil supplementation augments post-exercise immune function in young males. | 2012 | Gray P and Gabriel B and Thies F and Gray SR | Excluded | The article was excluded based on title and abstract screening as it did not meet the inclusion and exclusion criteria. |
| Mitophagy in human health, ageing and disease. | 2023 | Picca A and Faitg J and Auwerx J and Ferrucci L and D'Amico D | Excluded | The article was excluded based on title and abstract screening as it did not meet the inclusion and exclusion criteria. |
| Physiological and environmental factors affecting cancer risk and prognosis in obesity. | 2023 | Seke Etet PF and Vecchio L and Nwabo Kamdje AH and Mimche PN and Njamnshi AK and Adem A | Excluded | The article was excluded based on title and abstract screening as it did not meet the inclusion and exclusion criteria. |
| 31P magnetization transfer measurements of Pi→ATP flux in exercising human muscle. | 2016 | Sleigh A and Savage DB and Williams GB and Porter D and Carpenter TA and Brindle KM and Kemp GJ | Excluded | The article was excluded based on title and abstract screening as it did not meet the inclusion and exclusion criteria. |
| Creatine supplementation with exercise reduces α-synuclein oligomerization and necroptosis in Parkinson's disease mouse model. | 2024 | Leem YH and Park JS and Park JE and Kim DY and Kim HS | Excluded | The article was excluded based on title and abstract screening as it did not meet the inclusion and exclusion criteria. |
| Miracle Berry as a Potential Supplement in the Control of Metabolic Risk Factors in Cancer. | 2020 | Gómez de Cedrón M and Wagner S and Reguero M and Menéndez-Rey A and de Molina AR | Excluded | The article was excluded based on title and abstract screening as it did not meet the inclusion and exclusion criteria. |
| Enhanced antioxidant capacity and anti-ageing biomarkers after diet micronutrient supplementation. | 2014 | Balcerczyk A and Gajewska A and Macierzyńska-Piotrowska E and Pawelczyk T and Bartosz G and Szemraj J | Excluded | The article was excluded based on title and abstract screening as it did not meet the inclusion and exclusion criteria. |
| Structured Docosahexaenoic Acid (DHA) Enhances Motility and Promotes the Antioxidant Capacity of Aged C. elegans. | 2023 | Mora I and Pérez-Santamaria A and Tortajada-Pérez J and Vázquez-Manrique RP and Arola L and Puiggròs F | Excluded | The article was excluded based on title and abstract screening as it did not meet the inclusion and exclusion criteria. |
| SIRT6 regulates metabolic homeostasis in skeletal muscle through activation of AMPK. | 2017 | Cui X and Yao L and Yang X and Gao Y and Fang F and Zhang J and Wang Q and Chang Y | Excluded | The article was excluded based on title and abstract screening as it did not meet the inclusion and exclusion criteria. |
| Disentangling Mitochondria in Alzheimer's Disease. | 2021 | Johri A | Excluded | The article was excluded based on title and abstract screening as it did not meet the inclusion and exclusion criteria. |
| [Effect of melatonin on activity of superoxide dismutase (CuZn-SOD) in erythrocytes of patients during short- and long-term hypokinesis]. | 2010 | Mrowicka M and Garncarek P and Miller E and Kedziora J and Smigielski J and Malinowska K and Mrowicki J | Excluded | The article was excluded based on title and abstract screening as it did not meet the inclusion and exclusion criteria. |
| Endothelial Senescence and Its Impact on Angiogenesis in Alzheimer's Disease. | 2023 | Georgieva I and Tchekalarova J and Iliev D and Tzoneva R | Excluded | The article was excluded based on title and abstract screening as it did not meet the inclusion and exclusion criteria. |
| The Immunopathogenesis of Alzheimer's Disease Is Related to the Composition of Gut Microbiota. | 2021 | Leblhuber F and Ehrlich D and Steiner K and Geisler S and Fuchs D and Lanser L and Kurz K | Excluded | The article was excluded based on title and abstract screening as it did not meet the inclusion and exclusion criteria. |
| Mitochondrial oxidative phosphorylation is impaired in patients with congenital lipodystrophy. | 2012 | Sleigh A and Stears A and Thackray K and Watson L and Gambineri A and Nag S and Campi VI and Schoenmakers N and Brage S and Carpenter TA and Murgatroyd PR and O'Rahilly S and Kemp GJ and Savage DB | Excluded | The article was excluded based on title and abstract screening as it did not meet the inclusion and exclusion criteria. |
| The Role of Irisin in Alzheimer's Disease. | 2018 | Kim OY and Song J | Excluded | The article was excluded based on title and abstract screening as it did not meet the inclusion and exclusion criteria. |
| [An influence of submaximal (submineximal) doses of mexidol on oxidant stress and inflammation in the acute period of ischemic stroke]. | 2018 | Godunova AR and Rakhimova AA and Leontyeva OI and Talipova IG and Yakhin RM and Musin SG | Excluded | The article was excluded based on title and abstract screening as it did not meet the inclusion and exclusion criteria. |
| Aerobic Glycolysis: A DeOxymoron of (Neuro)Biology. | 2022 | Schurr A and Passarella S | Excluded | The article was excluded based on title and abstract screening as it did not meet the inclusion and exclusion criteria. |
| Epigenetic oxidative redox shift (EORS) theory of aging unifies the free radical and insulin signaling theories. | 2010 | Brewer GJ | Excluded | The article was excluded based on title and abstract screening as it did not meet the inclusion and exclusion criteria. |
| Identification of the redox-stress signaling threshold (RST): Increased RST helps to delay aging in C. elegans. | 2022 | Meng J and Lv Z and Wang Y and Chen C | Excluded | The article was excluded based on title and abstract screening as it did not meet the inclusion and exclusion criteria. |
| Exploring the Intricate Nexus of Sarcopenia and Cognitive Impairment. | 2023 | Xing Y and Li X and Ma L | Excluded | The article was excluded based on title and abstract screening as it did not meet the inclusion and exclusion criteria. |
| Mitochondrial Fatty Acid Oxidation Disorders Associated with Short-Chain Enoyl-CoA Hydratase (ECHS1) Deficiency. | 2018 | Sharpe AJ and McKenzie M | Excluded | The article was excluded based on title and abstract screening as it did not meet the inclusion and exclusion criteria. |
| Gut microbiota-mitochondrial inter-talk in non-alcoholic fatty liver disease. | 2022 | Zhang Q and Xing W and Wang Q and Tang Z and Wang Y and Gao W | Excluded | The article was excluded based on title and abstract screening as it did not meet the inclusion and exclusion criteria. |
| Mechanistic insights into the therapeutic use of high-dose allopurinol in angina pectoris. | 2011 | Rajendra NS and Ireland S and George J and Belch JJ and Lang CC and Struthers AD | Excluded | The article was excluded based on title and abstract screening as it did not meet the inclusion and exclusion criteria. |
| Regional blood flow supply and demand in heart failure. | 1990 | Levine TB and Levine AB | Excluded | The article was excluded based on title and abstract screening as it did not meet the inclusion and exclusion criteria. |
| Unlocking Modifiable Risk Factors for Alzheimer's Disease: Does the Oral Microbiome Hold Some of the Keys? | 2023 | Loughman A and Adler CJ and Macpherson H | Excluded | The article was excluded based on title and abstract screening as it did not meet the inclusion and exclusion criteria. |
| Cognitive Impairment in Parkinson's Disease: Epidemiology, Clinical Profile, Protective and Risk Factors. | 2021 | Gonzalez-Latapi P and Bayram E and Litvan I and Marras C | Excluded | The article was excluded based on title and abstract screening as it did not meet the inclusion and exclusion criteria. |
| Respiratory chain complex-I defect mimicking myasthenia. | 2002 | Finsterer J and Oberman I and Reitner A | Excluded | The article was excluded based on title and abstract screening as it did not meet the inclusion and exclusion criteria. |
| Naked mole-rat skeletal muscle mitochondria exhibit minimal functional plasticity in acute or chronic hypoxia. | 2021 | Cheng H and Munro D and Huynh K and Pamenter ME | Excluded | The article was excluded based on title and abstract screening as it did not meet the inclusion and exclusion criteria. |
| Salivary proteomic profile of response to different resistance training protocols: A case report. | 2024 | Santos KO and Filho DMP and Ventura TMO and Thomassian LTG and Macedo AG and Buzalaf MAR and Braga AS and Faria MH and Magalhães AC | Excluded | The article was excluded based on title and abstract screening as it did not meet the inclusion and exclusion criteria. |
| Reduced oxidative phosphorylation and proton efflux suggest reduced capillary blood supply in skeletal muscle of patients with dermatomyositis and polymyositis: a quantitative 31P-magnetic resonance spectroscopy and MRI study. | 2002 | Cea G and Bendahan D and Manners D and Hilton-Jones D and Lodi R and Styles P and Taylor DJ | Excluded | The article was excluded based on title and abstract screening as it did not meet the inclusion and exclusion criteria. |
| Carnosine, Small but Mighty-Prospect of Use as Functional Ingredient for Functional Food Formulation. | 2021 | Jukić I and Kolobarić N and Stupin A and Matić A and Kozina N and Mihaljević Z and Mihalj M and Šušnjara P and Stupin M and Ćurić ŽB and Selthofer-Relatić K and Kibel A and Lukinac A and Kolar L and Kralik G and Kralik Z and Széchenyi A and Jozanović M and Galović O and Medvidović-Kosanović M and Drenjančević I | Excluded | The article was excluded based on title and abstract screening as it did not meet the inclusion and exclusion criteria. |
| Acute pH alterations do not impact cardiac mitochondrial respiration in naked mole-rats or mice. | 2022 | Huynh KW and Sabir S and Cheng H and Pamenter ME | Excluded | The article was excluded based on title and abstract screening as it did not meet the inclusion and exclusion criteria. |
| Lifestyle Modifications and Nutritional Interventions in Aging-Associated Cognitive Decline and Alzheimer's Disease. | 2019 | Bhatti GK and Reddy AP and Reddy PH and Bhatti JS | Excluded | The article was excluded based on title and abstract screening as it did not meet the inclusion and exclusion criteria. |
| The intestinal microbiome and its relevance for functionality in older persons. | 2019 | Ticinesi A and Tana C and Nouvenne A | Excluded | The article was excluded based on title and abstract screening as it did not meet the inclusion and exclusion criteria. |
| 9- and 13-Hydroxy-octadecadienoic acids (9+13 HODE) are inversely related to granulocyte colony stimulating factor and IL-6 in runners after 2h running. | 2016 | Nieman DC and Meaney MP and John CS and Knagge KJ and Chen H | Excluded | The article was excluded based on title and abstract screening as it did not meet the inclusion and exclusion criteria. |
| Prognostic relevance of ischemia-modified albumin and NT-proBNP in patients with peripheral arterial occlusive disease. | 2015 | Falkensammer J and Frech A and Duschek N and Gasteiger S and Stojakovic T and Scharnagl H and Huber K and Fraedrich G and Greiner A | Excluded | The article was excluded based on title and abstract screening as it did not meet the inclusion and exclusion criteria. |
| Importance of Bmal1 in Alzheimer's disease and associated aging-related diseases: Mechanisms and interventions. | 2022 | Fan R and Peng X and Xie L and Dong K and Ma D and Xu W and Shi X and Zhang S and Chen J and Yu X and Yang Y | Excluded | The article was excluded based on title and abstract screening as it did not meet the inclusion and exclusion criteria. |
| The mediation roles of intermuscular fat and inflammation in muscle mitochondrial associations with cognition and mobility. | 2024 | Tian Q and Lee PR and Yang Q and Moore AZ and Landman BA and Resnick SM and Ferrucci L | Excluded | The article was excluded based on title and abstract screening as it did not meet the inclusion and exclusion criteria. |
| Fluoxetine and environmental enrichment similarly reverse chronic social stress-related depression- and anxiety-like behavior, but have differential effects on amygdala gene expression. | 2021 | Cordner ZA and Marshall-Thomas I and Boersma GJ and Lee RS and Potash JB and Tamashiro KLK | Excluded | The article was excluded based on title and abstract screening as it did not meet the inclusion and exclusion criteria. |
| Metabolic modulation predicts heart failure tests performance. | 2019 | Contaifer D Jr and Buckley LF and Wohlford G and Kumar NG and Morriss JM and Ranasinghe AD and Carbone S and Canada JM and Trankle C and Abbate A and Van Tassell BW and Wijesinghe DS | Excluded | The article was excluded based on title and abstract screening as it did not meet the inclusion and exclusion criteria. |
| [The Clinical Efficacy and Possible Mechanism of Combination Treatment of Cerebral Ischemic Stroke with Ginkgo Biloba Extract and Low-Frequency Repetitive Transcranial Magnetic Stimulation]. | 2021 | Peng Y and Lin Y and Yu NW and Liao XL and Shi L | Excluded | The article was excluded based on title and abstract screening as it did not meet the inclusion and exclusion criteria. |
| A potential role for free radical-mediated skeletal muscle soreness in the pathophysiology of acute mountain sickness. | 2001 | Bailey DM and Davies B and Young IS and Hullin DA and Seddon PS | Excluded | The article was excluded based on title and abstract screening as it did not meet the inclusion and exclusion criteria. |
| Anxiety disorders in childhood are associated with youth IL-6 levels: A mediation study including metabolic stress and childhood traumatic events. | 2019 | de Baumont A and Bortoluzzi A and Wollenhaupt de Aguiar B and Scotton E and Pinto Guimarães LS and Kapczinski F and Belem da Silva CT and Manfro GG | Excluded | The article was excluded based on title and abstract screening as it did not meet the inclusion and exclusion criteria. |
| Exogenous Ketone Supplements in Athletic Contexts: Past, Present, and Future. | 2022 | Evans M and McClure TS and Koutnik AP and Egan B | Excluded | The article was excluded based on title and abstract screening as it did not meet the inclusion and exclusion criteria. |
| Therapies for Prevention and Treatment of Alzheimer's Disease. | 2016 | Mendiola-Precoma J and Berumen LC and Padilla K and Garcia-Alcocer G | Excluded | The article was excluded based on title and abstract screening as it did not meet the inclusion and exclusion criteria. |
| Magnetic resonance spectroscopy reveals mitochondrial dysfunction in amyotrophic lateral sclerosis. | 2020 | Sassani M and Alix JJ and McDermott CJ and Baster K and Hoggard N and Wild JM and Mortiboys HJ and Shaw PJ and Wilkinson ID and Jenkins TM | Excluded | The article was excluded based on title and abstract screening as it did not meet the inclusion and exclusion criteria. |
| Microvascular response to exercise varies along the length of the tibialis anterior muscle. | 2022 | Veeger TTJ and Hirschler L and Baligand C and Franklin SL and Webb AG and de Groot JH and van Osch MJP and Kan HE | Excluded | The article was excluded based on title and abstract screening as it did not meet the inclusion and exclusion criteria. |
| Assessment of the effects of glutamic acid decarboxylase antibodies and trace elements on cognitive performance in older adults. | 2015 | Alghadir AH and Gabr SA and Al-Eisa ES | Excluded | The article was excluded based on title and abstract screening as it did not meet the inclusion and exclusion criteria. |
| Depression and markers of inflammation as predictors of all-cause mortality in heart failure. | 2016 | Mommersteeg PMC and Schoemaker RG and Naudé PJW and Eisel ULM and Garrelds IM and Schalkwijk CG and Westerhuis BWJJM and Kop WJ and Denollet J | Excluded | The article was excluded based on title and abstract screening as it did not meet the inclusion and exclusion criteria. |
| Nutritional and Wellness Strategies for Neurological and Psychiatric Recovery From Post-COVID Syndrome and Post-acute Sequelae of COVID-19. | 2023 | Schaefer J and Khanna D | Excluded | The article was excluded based on title and abstract screening as it did not meet the inclusion and exclusion criteria. |
| Associations of Social Isolation and Loneliness With Later Dementia. | 2022 | Shen C and Rolls ET and Cheng W and Kang J and Dong G and Xie C and Zhao XM and Sahakian BJ and Feng J | Excluded | The article was excluded based on title and abstract screening as it did not meet the inclusion and exclusion criteria. |
| [Ropinirole Hydrochloride, a Candidate Drug for ALS Treatment]. | 2019 | Takahashi S and Morimoto S and Okano H | Excluded | The article was excluded based on title and abstract screening as it did not meet the inclusion and exclusion criteria. |
| The "Angiogenic Switch" and Functional Resources in Cyclic Sports Athletes. | 2021 | Balberova OV and Bykov EV and Shnayder NA and Petrova MM and Gavrilyuk OA and Kaskaeva DS and Soloveva IA and Petrov KV and Mozheyko EY and Medvedev GV and Nasyrova RF | Excluded | The article was excluded based on title and abstract screening as it did not meet the inclusion and exclusion criteria. |
| Ghrelin alleviates depression-like behaviour in rats subjected to high-fat diet and diurnal rhythm disturbance. | 2022 | Pawar GR and Agrawal YO and Nakhate KT and Patil CR and Sharma C and Ojha S and Mahajan UB and Goyal SN | Excluded | The article was excluded based on title and abstract screening as it did not meet the inclusion and exclusion criteria. |
| Effects of 21 days of bed rest and whey protein supplementation on plantar flexor muscle fatigue resistance during repeated shortening contractions. | 2020 | Bosutti A and Mulder E and Zange J and Bühlmeier J and Ganse B and Degens H | Excluded | The article was excluded based on title and abstract screening as it did not meet the inclusion and exclusion criteria. |
| Identification of a novel dehydroergosterol enhancing microglial anti-inflammatory activity in a dairy product fermented with Penicillium candidum. | 2015 | Ano Y and Kutsukake T and Hoshi A and Yoshida A and Nakayama H | Excluded | The article was excluded based on title and abstract screening as it did not meet the inclusion and exclusion criteria. |
| Carbon monoxide intoxication. | 2015 | Bleecker ML | Excluded | The article was excluded based on title and abstract screening as it did not meet the inclusion and exclusion criteria. |
| Effect of melatonin on heart failure: design for a double-blinded randomized clinical trial. | 2020 | Sadeghi M and Khosrawi S and Heshmat-Ghahdarijani K and Gheisari Y and Roohafza H and Mansoorian M and Hoseini SG | Excluded | The article was excluded based on title and abstract screening as it did not meet the inclusion and exclusion criteria. |
| Calorie Restriction-Regulated Molecular Pathways and Its Impact on Various Age Groups: An Overview. | 2022 | Rachakatla A and Kalashikam RR | Excluded | The article was excluded based on title and abstract screening as it did not meet the inclusion and exclusion criteria. |
| Biomarker Signatures of Two Phenotypical Prefrailty Types in the Irish Longitudinal Study on Ageing. | 2022 | Piankova P and Romero-Ortuno R and O'Halloran AM | Excluded | The article was excluded based on title and abstract screening as it did not meet the inclusion and exclusion criteria. |
| Plasma GSH levels and Alzheimer's disease. A prospective approach.: Results from the HELIAD study. | 2021 | Charisis S and Ntanasi E and Yannakoulia M and Anastasiou CA and Kosmidis MH and Dardiotis E and Hadjigeorgiou G and Sakka P and Veskoukis AS and Kouretas D and Scarmeas N | Excluded | The article was excluded based on title and abstract screening as it did not meet the inclusion and exclusion criteria. |
| Mitochondria and diabetes. Genetic, biochemical, and clinical implications of the cellular energy circuit. | 1996 | Gerbitz KD and Gempel K and Brdiczka D | Excluded | The article was excluded based on title and abstract screening as it did not meet the inclusion and exclusion criteria. |
| Efficacy and Safety of Elamipretide in Individuals With Primary Mitochondrial Myopathy: The MMPOWER-3 Randomized Clinical Trial. | 2023 | Karaa A and Bertini E and Carelli V and Cohen BH and Enns GM and Falk MJ and Goldstein A and Gorman GS and Haas R and Hirano M and Klopstock T and Koenig MK and Kornblum C and Lamperti C and Lehman A and Longo N and Molnar MJ and Parikh S and Phan H and Pitceathly RDS and Saneto R and Scaglia F and Servidei S and Tarnopolsky M and Toscano A and Van Hove JLK and Vissing J and Vockley J and Finman JS and Brown DA and Shiffer JA and Mancuso M | Excluded | The article was excluded based on title and abstract screening as it did not meet the inclusion and exclusion criteria. |
| Gut microbiota composition in patients with newly diagnosed bipolar disorder and their unaffected first-degree relatives. | 2019 | Coello K and Hansen TH and Sørensen N and Munkholm K and Kessing LV and Pedersen O and Vinberg M | Excluded | The article was excluded based on title and abstract screening as it did not meet the inclusion and exclusion criteria. |
| Safety, pharmacodynamics, and potential benefit of omaveloxolone in Friedreich ataxia. | 2019 | Lynch DR and Farmer J and Hauser L and Blair IA and Wang QQ and Mesaros C and Snyder N and Boesch S and Chin M and Delatycki MB and Giunti P and Goldsberry A and Hoyle C and McBride MG and Nachbauer W and O'Grady M and Perlman S and Subramony SH and Wilmot GR and Zesiewicz T and Meyer C | Excluded | The article was excluded based on title and abstract screening as it did not meet the inclusion and exclusion criteria. |
| Alteration of skeletal muscle energy metabolism assessed by (31) P MRS in clinical routine: Part 2. Clinical application. | 2023 | Naëgel A and Ratiney H and Karkouri J and Kennouche D and Royer N and Slade JM and Morel J and Croisille P and Viallon M | Excluded | The article was excluded based on title and abstract screening as it did not meet the inclusion and exclusion criteria. |
| Part IX • Chapter 26 - Influence of exercise-induced oxidative stress on the central nervous system | 2000 | Somani, Satu M. and Husain, Kazim and Sen, Chandan K. and Packer, Lester and Hänninen, Osmo O.P. | Excluded | The article was excluded based on title and abstract screening as it did not meet the inclusion and exclusion criteria. |
| Voluntary wheel running, but not a diet containing (−)-epigallocatechin-3-gallate and β-alanine, improves learning, memory and hippocampal neurogenesis in aged mice | 2014 | Gibbons, Trisha E. and Pence, Brandt D. and Petr, Geraldine and Ossyra, Jessica M. and Mach, Houston C. and Bhattacharya, Tushar K. and Perez, Samuel and Martin, Stephen A. and McCusker, Robert H. and Kelley, Keith W. and Rhodes, Justin S. and Johnson, Rodney W. and Woods, Jeffrey A. | Excluded | The article was excluded based on title and abstract screening as it did not meet the inclusion and exclusion criteria. |
| The pleiotropic neuroprotective effects of resveratrol in cognitive decline and Alzheimer’s disease pathology: From antioxidant to epigenetic therapy | 2021 | Griñán-Ferré, Christian and Bellver-Sanchis, Aina and Izquierdo, Vanessa and Corpas, Rubén and Roig-Soriano, Joan and Chillón, Miguel and Andres-Lacueva, Cristina and Somogyvári, Milán and Sőti, Csaba and Sanfeliu, Coral and Pallàs, Mercè | Excluded | The article was excluded based on title and abstract screening as it did not meet the inclusion and exclusion criteria. |
| Excitotoxins | 2007 | Mattson, M.P. and Fink, George | Excluded | The article was excluded based on title and abstract screening as it did not meet the inclusion and exclusion criteria. |
| Treadmill exercise slows cognitive deficits in aging rats by antioxidation and inhibition of amyloid production | 2013 | Yu, F and Xu, B and Song, CH and Ji, L and Zhang, XL | Included |  |
| Chapter 37 - Antioxidant diets and functional foods attenuate dementia and cognition in elderly subjects | 2022 | Wichansawakun, Sanit and Chupisanyarote, Kanokkarn and Wongpipathpong, Wannisa and Kaur, Ginpreet and Buttar, Harpal S. and Singh, Ram B. and Watanabe, Shaw and Isaza, Adrian A. | Excluded | The article was excluded based on title and abstract screening as it did not meet the inclusion and exclusion criteria. |
| Converging protective pathways: Exploring the linkage between physical exercise, extracellular vesicles and oxidative stress | 2023 | Lisi, Veronica and Senesi, Giorgia and Balbi, Carolina | Excluded | The article was excluded based on title and abstract screening as it did not meet the inclusion and exclusion criteria. |
| Mechanism of CNS regulation by irisin, a multifunctional protein | 2022 | Qi, Jing-yu and Yang, Liu-kun and Wang, Xin-shang and Wang, Min and Li, Xu-bo and Feng, Ban and Wu, Yu-mei and Liu, Shui-bing and Zhang, Kun | Excluded | The article was excluded based on title and abstract screening as it did not meet the inclusion and exclusion criteria. |
| Exercise-induced Brain-derived neurotrophic factor regulation in the brain dysfunctions | 2023 | Rezaee, Z. and Marandi, S.M. and Alaei, H. and Esfarjani, F. | Excluded | The article was excluded based on title and abstract screening as it did not meet the inclusion and exclusion criteria. |
| Caffeine effects on systemic metabolism, oxidative-inflammatory pathways, and exercise performance | 2020 | Barcelos, Rômulo P and Lima, Frederico D and Carvalho, Nelson R and Bresciani, Guilherme and Royes, Luiz FF | Excluded | The article was excluded based on title and abstract screening as it did not meet the inclusion and exclusion criteria. |
| Oxygen-enriched air reduces breathing gas consumption over air | 2022 | Schipke, J.D. and Deussen, A. and Moeller, F. and Hoffmann, U. and Muth, T. and Zenske, A. and Koch, A. | Excluded | The article was excluded based on title and abstract screening as it did not meet the inclusion and exclusion criteria. |
| Ultra-Endurance Associated With Moderate Exercise in Rats Induces Cerebellar Oxidative Stress and Impairs Reactive GFAP Isoform Profile | 2020 | de Souza, R.F. and Augusto, R.L. and de Moraes, S.R.A. and de Souza, F.B. and Gonçalves, L.V.D.P. and Pereira, D.D. and Moreno, G.M.M. and de Souza, F.M.A. and Andrade-da-Costa, B.L.D.S. | Included |  |
| Chapter 11 - Excitotoxicity | 2019 | Mattson, M.P. and Fink, George | Excluded | The article was excluded based on title and abstract screening as it did not meet the inclusion and exclusion criteria. |
| Photoperiod Is Associated With Hippocampal Volume in a Large Community Sample | 2015 | Miller, MA and Leckie, RL and Donofry, SD and Gianaros, PJ and Erickson, KI and Manuck, SB and Roecklein, KA | Excluded | The article was excluded based on title and abstract screening as it did not meet the inclusion and exclusion criteria. |
| Olfactory bulbectomy and raphe nucleus relationship: a new vision for well-known depression model | 2020 | Ozcan, H and Aydin, N and Aydin, MD and Oral, E and Gündogdu, C and Sipal, S and Halici, Z | Excluded | The article was excluded based on title and abstract screening as it did not meet the inclusion and exclusion criteria. |
| Resilience Against Traumatic Stress: Current Developments and Future Directions | 2018 | Snijders, C and Pries, LK and Sgammeglia, N and Al Jowf, G and Youssef, NA and de Nijs, L and Guloksuz, S and Rutten, BPF | Excluded | The article was excluded based on title and abstract screening as it did not meet the inclusion and exclusion criteria. |
| Reactions of sheep towards three sets of emotional stimuli: (In)Consistency in respect to stimulus valence and sheep identity | 2016 | Gygax, L and Vögeli, S | Excluded | The article was excluded based on title and abstract screening as it did not meet the inclusion and exclusion criteria. |
| STRESS-INDUCED CHANGES OF EXTRACELLULAR 5-HYDROXYINDOLEACETIC ACID CONCENTRATIONS FOLLOWED IN THE NUCLEUS-RAPHE-DORSALIS AND THE FRONTAL-CORTEX OF THE RAT | 1993 | CLEMENT, HW and SCHAFER, F and RUWE, C and GEMSA, D and WESEMANN, W | Excluded | The article was excluded based on title and abstract screening as it did not meet the inclusion and exclusion criteria. |
| Flat-floored Air-lifted Platform: A New Method for Combining Behavior with Microscopy or Electrophysiology on Awake Freely Moving Rodents | 2014 | Kislin, M and Mugantseva, E and Molotkov, D and Kulesskaya, N and Khirug, S and Kirilkin, I and Pryazhnikov, E and Kolikova, J and Toptunov, D and Yuryev, M and Giniatullin, R and Voikar, V and Rivera, C and Rauvala, H and Khiroug, L | Excluded | The article was excluded based on title and abstract screening as it did not meet the inclusion and exclusion criteria. |
| Phenotypic characterization of paroxysmal dyskinesia in Maltese dogs | 2020 | Polidoro, D and Ham, LV and Santens, P and Cornelis, I and Charalambous, M and Broeckx, BJG and Bhatti, SFM | Excluded | The article was excluded based on title and abstract screening as it did not meet the inclusion and exclusion criteria. |
| MAN AND ENVIRONMENT | 1993 | HADAC, E | Excluded | The article was excluded based on title and abstract screening as it did not meet the inclusion and exclusion criteria. |
| Adaptogenic properties of six <i>Rasayana</i> herbs used in ayurvedic medicine | 1999 | Rege, NN and Thatte, UM and Dahanukar, SA | Excluded | The article was excluded based on title and abstract screening as it did not meet the inclusion and exclusion criteria. |
| SIGNIFICANCE OF ANIMAL STUDIES FOR PERINATAL RESEARCH | 1993 | RUDOLPH, AM | Excluded | The article was excluded based on title and abstract screening as it did not meet the inclusion and exclusion criteria. |
| Brain-derived neurotrophic factor in serum as an animal welfare indicator of environmental enrichment in pigs | 2018 | Rault, JL and Lawrence, AJ and Ralph, CR | Excluded | The article was excluded based on title and abstract screening as it did not meet the inclusion and exclusion criteria. |
| Potential of Whole-Body Vibration in Parkinson's Disease: A Systematic Review and Meta-Analysis of Human and Animal Studies | 2022 | Arauz, YLA and Ahuja, G and Kamsma, YPT and Kortholt, A and van der Zee, EA and van Heuvelen, MJG | Excluded | The article was excluded based on title and abstract screening as it did not meet the inclusion and exclusion criteria. |
| Exercise and BDNF reduce Aβ production by enhancing α-secretase processing of APP | 2017 | Nigam, SM and Xu, SH and Kritikou, JS and Marosi, K and Brodin, L and Mattson, MP | Excluded | The article was excluded based on title and abstract screening as it did not meet the inclusion and exclusion criteria. |
| Music exposure attenuates anxiety- and depression-like behaviors and increases hippocampal spine density in male rats | 2019 | Papadakakis, A and Sidiropoulou, K and Panagis, G | Excluded | The article was excluded based on title and abstract screening as it did not meet the inclusion and exclusion criteria. |
| The developmental consequences of early adverse care on infant macaques: A cross-fostering study | 2022 | McCormack, KM and Howell, BR and Higgins, M and Bramlett, S and Guzman, D and Morin, EL and Villongco, C and Liu, Y and Meyer, J and Sanchez, MM | Excluded | The article was excluded based on title and abstract screening as it did not meet the inclusion and exclusion criteria. |
| Boldness and physiological variation in round goby populations along their Baltic Sea invasion front | 2023 | Galli, A and Behrens, JW and Gesto, M and Moran, NP | Excluded | The article was excluded based on title and abstract screening as it did not meet the inclusion and exclusion criteria. |
| Stimulation of the β<sub>3</sub>-adrenoceptor as a novel treatment strategy for anxiety and depressive disorders | 2008 | Stemmelin, J and Cohen, C and Terranova, JP and Lopez-Grancha, M and Pichat, P and Bergis, O and Decobert, M and Santucci, V and Francon, D and Alonso, R and Stahl, SM and Keane, P and Avenet, P and Scatton, B and le Fur, G and Griebel, G | Excluded | The article was excluded based on title and abstract screening as it did not meet the inclusion and exclusion criteria. |
| EFFECTS OF REARING CONDITION ON ACTIVITY-INDUCED WEIGHT | 1995 | NESS, JW and MARSHALL, TR and ARAVICH, PF | Excluded | The article was excluded based on title and abstract screening as it did not meet the inclusion and exclusion criteria. |
| Antidepressant-like effect of <i>Butea superba</i> in mice exposed to chronic mild stress and its possible mechanism of action | 2014 | Mizuki, D and Matsumoto, K and Tanaka, K and Le, XT and Fujiwara, H and Ishikawa, T and Higuchi, Y | Excluded | The article was excluded based on title and abstract screening as it did not meet the inclusion and exclusion criteria. |
| Panting in reindeer (<i>Rangifer tarandus</i>) | 2000 | Aas-Hansen, O and Folkow, LP and Blix, AS | Excluded | The article was excluded based on title and abstract screening as it did not meet the inclusion and exclusion criteria. |
| Perinatal inflammatory cytokine challenge results in distinct neurobehavioral alterations in rats: implication in psychiatric disorders of developmental origin | 2004 | Tohmi, M and Tsuda, N and Watanabe, Y and Kakita, A and Nawa, H | Excluded | The article was excluded based on title and abstract screening as it did not meet the inclusion and exclusion criteria. |
| Environmental enrichment in mice decreases anxiety, attenuates stress responses and enhances natural killer cell activity | 2004 | Benaroya-Milshtein, N and Hollander, N and Apter, A and Kukulansky, T and Raz, N and Wilf, A and Yaniv, I and Pick, CG | Excluded | The article was excluded based on title and abstract screening as it did not meet the inclusion and exclusion criteria. |
| Brain and arterial blood temperatures of free-ranging oryx (<i>Oryx gazella</i>) | 2002 | Maloney, SK and Fuller, A and Mitchell, G and Mitchell, D | Excluded | The article was excluded based on title and abstract screening as it did not meet the inclusion and exclusion criteria. |
| PRENATAL ETHANOL EXPOSURE DIFFERENTIALLY AFFECTS HIPPOCAMPAL NEUROGENESIS IN THE ADOLESCENT AND AGED BRAIN | 2014 | Gil-Mohapel, J and Titterness, AK and Patten, AR and Taylor, S and Ratzlaff, A and Ratzlaff, T and Helfer, J and Christie, BR | Excluded | The article was excluded based on title and abstract screening as it did not meet the inclusion and exclusion criteria. |
| SPECIALIZED BRAIN COOLING IN HUMANS | 1993 | BRENGELMANN, GL | Excluded | The article was excluded based on title and abstract screening as it did not meet the inclusion and exclusion criteria. |
| Microglial translocator protein and stressor-related disorder | 2020 | Kikutani, K and Giga, H and Hosokawa, K and Shime, N and Aizawa, H | Excluded | The article was excluded based on title and abstract screening as it did not meet the inclusion and exclusion criteria. |
| Purposeful Activity in Psychiatric Rehabilitation: Is Neurogenesis a Key Player? | 2016 | Cheung, JSC and Chan, JNM and Lau, BWM and Ngai, SPC | Excluded | The article was excluded based on title and abstract screening as it did not meet the inclusion and exclusion criteria. |
| Myricetin Inhibited Fear and Anxiety-Like Behaviors by HPA Axis Regulation and Activation of the BDNF-ERK Signaling Pathway in Posttraumatic Stress Disorder Rats | 2022 | Sur, B and Lee, B | Excluded | The article was excluded based on title and abstract screening as it did not meet the inclusion and exclusion criteria. |
| Maternal Supplementation With Avocado (<i>Persea americana</i> Mill.) Pulp and Oil Alters Reflex Maturation, Physical Development, and Offspring Memory in Rats | 2019 | de Melo, MFFT and Pereira, DE and Moura, RD and da Silva, EB and de Melo, FALT and Dias, CDQ and Silva, MDA and de Oliveira, MEG and Viera, VB and Pintado, MME and dos Santos, SG and Soares, JKB | Excluded | The article was excluded based on title and abstract screening as it did not meet the inclusion and exclusion criteria. |
| Mechanisms for the control of respiratory evaporative heat loss in panting animals | 2006 | Robertshaw, D | Excluded | The article was excluded based on title and abstract screening as it did not meet the inclusion and exclusion criteria. |
| Glia atrophy in the hippocampus of chronic unpredictable stress-induced depression model rats is reversed by electroacupuncture treatment | 2011 | Liu, QO and Li, B and Zhu, HY and Wang, YQ and Yu, J and Wu, GC | Excluded | The article was excluded based on title and abstract screening as it did not meet the inclusion and exclusion criteria. |
| Disease progression in a mouse model of amyotrophic lateral sclerosis: the influence of chronic stress and corticosterone | 2011 | Fidler, JA and Treleaven, CM and Frakes, A and Tamsett, TJ and McCrate, M and Cheng, SH and Shihabuddin, LS and Kaspar, BK and Dodge, JC | Excluded | The article was excluded based on title and abstract screening as it did not meet the inclusion and exclusion criteria. |
| Perinatal effects of scorpion venoms: maternal and offspring development | 2017 | Dorce, ALC and Martins, AD and Dorce, VAC and Nencioni, ALA | Excluded | The article was excluded based on title and abstract screening as it did not meet the inclusion and exclusion criteria. |
| Voluntary Running in Young Adult Mice Reduces Anxiety-Like Behavior and Increases the Accumulation of Bioactive Lipids in the Cerebral Cortex | 2013 | Santos-Soto, IJ and Chorna, N and Carballeira, NM and Vélez-Bartolomei, JG and Méndez-Merced, AT and Chornyy, AP and de Ortiz, SP | Excluded | The article was excluded based on title and abstract screening as it did not meet the inclusion and exclusion criteria. |
| Metabolism and Aging: Effects of Cold Exposure on Metabolic Rate, Body Composition, and Longevity in Mice | 2009 | Vaanholt, LM and Daan, S and Schubert, KA and Visser, GH | Excluded | The article was excluded based on title and abstract screening as it did not meet the inclusion and exclusion criteria. |
| Mechanisms for sex differences in energy homeostasis | 2019 | Wang, CM and Xu, Y | Excluded | The article was excluded based on title and abstract screening as it did not meet the inclusion and exclusion criteria. |
| Differential effects of voluntary wheel running and toy rotation on the mRNA expression of neurotrophic factors and <i>FKBP5</i> in a post-traumatic stress disorder rat model with the shuttle-box task | 2018 | Tanichi, M and Toda, H and Shimizu, K and Koga, M and Saito, T and Enomoto, S and Boku, S and Asai, F and Mitsui, Y and Nagamine, M and Fujita, M and Yoshino, A | Excluded | The article was excluded based on title and abstract screening as it did not meet the inclusion and exclusion criteria. |
| Prenatal Phencyclidine Exposure Alters Hippocampal Cell Proliferation in Offspring Rats | 2009 | Tanimura, A and Liu, J and Namba, T and Seki, T and Matsubara, Y and Itoh, M and Suzuki, T and Arai, H | Excluded | The article was excluded based on title and abstract screening as it did not meet the inclusion and exclusion criteria. |
| Childhood Threat Is Associated With Lower Resting-State Connectivity Within a Central Visceral Network | 2022 | Banihashemi, L and Peng, CW and Rangarajan, A and Karim, HT and Wallace, ML and Sibbach, BM and Singh, J and Stinley, MM and Germain, A and Aizenstein, HJ | Excluded | The article was excluded based on title and abstract screening as it did not meet the inclusion and exclusion criteria. |
| Exercise induces age-dependent changes on epigenetic parameters in rat hippocampus: A preliminary study | 2013 | Elsner, VR and Lovatel, GA and Moysés, F and Bertoldi, K and Spindler, C and Cechinel, LR and Muotri, AR and Siqueira, IR | Excluded | The article was excluded based on title and abstract screening as it did not meet the inclusion and exclusion criteria. |
| Neuroendocrine and behavioral response to social confrontation: residents versus intruders, active versus passive coping styles | 2005 | Ebner, K and Wotjak, CT and Landgraf, R and Engelmann, M | Excluded | The article was excluded based on title and abstract screening as it did not meet the inclusion and exclusion criteria. |
| Probable involvement of serotonin in the increased permeability of the blood-brain barrier by forced swimming. An experimental study using Evans blue and I-131-sodium tracers in the rat | 1996 | Sharma, HS and Westman, J and Navarro, JC and Dey, PK and Nyberg, F | Excluded | The article was excluded based on title and abstract screening as it did not meet the inclusion and exclusion criteria. |
| Effects of exercise on male copulatory behavior after β-adrenoreceptor blockade | 2009 | Thom, NJ and Holmes, PV and Dishman, RK | Excluded | The article was excluded based on title and abstract screening as it did not meet the inclusion and exclusion criteria. |
| Activation of rat pituitary-adrenocortical and sympatho-adrenomedullary system in response to different stressors | 2005 | Gavrilovic, L and Dronjak, S | Excluded | The article was excluded based on title and abstract screening as it did not meet the inclusion and exclusion criteria. |
| Dl-3-n-butylphthalide prevents chronic restraint stress-induced depression-like behaviors and cognitive impairment via regulating CaMKII/CREB/BDNF signaling pathway in hippocampus | 2022 | Shen, J and Yang, L and Wei, WS | Excluded | The article was excluded based on title and abstract screening as it did not meet the inclusion and exclusion criteria. |
| Depresssion, anxiety and other cognitive consequences of social isolation: Drug and non-drug treatments | 2021 | Zadeh, ZT and Rahmani, S and Alidadi, F and Joushi, S and Esmaeilpour, K | Excluded | The article was excluded based on title and abstract screening as it did not meet the inclusion and exclusion criteria. |
| Protective and damaging effects of mediators of stress - Elaborating and testing the concepts of allostasis and allostatic load | 1999 | McEwen, BS and Seeman, T | Excluded | The article was excluded based on title and abstract screening as it did not meet the inclusion and exclusion criteria. |
| The gut microbiome and Alzheimer's disease: Complex and bidirectional interactions | 2022 | Tarawneh, R and Penhos, E | Excluded | The article was excluded based on title and abstract screening as it did not meet the inclusion and exclusion criteria. |
| Early postnatal handling alters social behavior, learning, and memory of pre- and postnatal VPA-induced rat models of autism in a context-based manner | 2022 | Shahrbabaki, SSV and Jonaidi, H and Sheibani, V and Bashiri, H | Excluded | The article was excluded based on title and abstract screening as it did not meet the inclusion and exclusion criteria. |
| A memory-based neuronal substrate model of psychogenic non-epileptic seizure and posttraumatic stress disorder | 2019 | Dawood, S | Excluded | The article was excluded based on title and abstract screening as it did not meet the inclusion and exclusion criteria. |
| Effects of exercise on NMDA receptor subunit contributions to bidirectional synaptic plasticity in the mouse dentate gyrus | 2007 | Vasuta, C and Caunt, C and James, R and Samadi, S and Schibuk, E and Kannangara, T and Titterness, AK and Christie, BR | Excluded | The article was excluded based on title and abstract screening as it did not meet the inclusion and exclusion criteria. |
| α4βδ-GABA<sub>A</sub> receptors in dorsal hippocampal CA1 of adolescent female rats traffic to the plasma membrane of dendritic spines following voluntary exercise and contribute to protection of animals from activity-based anorexia through localization at excitatory synapses | 2018 | Aoki, C and Chen, YW and Chowdhury, TG and Piper, W | Excluded | The article was excluded based on title and abstract screening as it did not meet the inclusion and exclusion criteria. |
| Hippocampal amino-3-hydroxy-5-methyl-4-isoxazolepropionic acid GluA1 (AMPA GluA1) receptor subunit involves in learning and memory improvement following treatment with <i>Centella asiatica</i> extract in adolescent rats | 2018 | Yeo, NABMY and Muthuraju, S and Wong, JH and Mohammed, FR and Senik, MH and Zhang, JL and Yusof, SR and Jaafar, H and Adenan, MI and Mohamad, H and Muhammad, TST and Abdullah, JM | Excluded | The article was excluded based on title and abstract screening as it did not meet the inclusion and exclusion criteria. |
| Chronic all-trans retinoic acid administration induced hyperactivity of HPA axis and behavioral changes in young rats | 2010 | Cai, L and Yan, XB and Chen, XN and Meng, QY and Zhou, JN | Excluded | The article was excluded based on title and abstract screening as it did not meet the inclusion and exclusion criteria. |
| Environmental Enrichment Confers Stress Resiliency to Social Defeat through an Infralimbic Cortex-Dependent Neuroanatomical Pathway | 2011 | Lehmann, ML and Herkenham, M | Excluded | The article was excluded based on title and abstract screening as it did not meet the inclusion and exclusion criteria. |
| Enriched environment improves motor function in intact and unilateral dopamine-depleted rats | 2006 | Jadavji, NM and Kolb, B and Metz, GA | Excluded | The article was excluded based on title and abstract screening as it did not meet the inclusion and exclusion criteria. |
| Lessons from nature: Leveraging the freeze-tolerant wood frog as a model to improve organ cryopreservation and biobanking | 2022 | Al-attar, R and Storey, KB | Excluded | The article was excluded based on title and abstract screening as it did not meet the inclusion and exclusion criteria. |
| Endotoxaemia does not limit heat tolerance in rats: the role of plasma lipoproteins | 2000 | Caputa, M and Dokladny, K and Kurowicka, B | Excluded | The article was excluded based on title and abstract screening as it did not meet the inclusion and exclusion criteria. |
| Evaluation of a modified rat model for functional dyspepsia | 2018 | Liang, QK and Yan, Y and Mao, LF and Du, XJ and Liang, JJ and Liu, JH and Wang, LD and Li, HF | Excluded | The article was excluded based on title and abstract screening as it did not meet the inclusion and exclusion criteria. |
| Pharmacokinetics and tissue distribution of orally administrated imidazole dipeptides in carnosine synthase gene knockout mice | 2022 | Sakano, T and Egusa, AS and Kawauchi, Y and Wu, JW and Nishimura, T and Nakao, N and Kuramoto, A and Kawashima, T and Shiotani, S and Okada, Y and Sato, K and Yanai, N | Excluded | The article was excluded based on title and abstract screening as it did not meet the inclusion and exclusion criteria. |
| Twenty Years of Cerebellar Degeneration Research at the Department of Pathological Physiology, Faculty of Medicine, Pilsen, Charles University | 2018 | Vozeh, F | Excluded | The article was excluded based on title and abstract screening as it did not meet the inclusion and exclusion criteria. |
| A reward increases running-wheel performance without changing cell proliferation, neuronal differentiation or cell death in the dentate gyrus of C57BL/6 mice | 2009 | Klaus, F and Hauser, T and Slomianka, L and Lipp, HP and Amrein, I | Excluded | The article was excluded based on title and abstract screening as it did not meet the inclusion and exclusion criteria. |
| The relevance of hierarchies, territories, defeat for depression in humans: hypotheses and clinical predictions | 2001 | Rohde, P | Excluded | The article was excluded based on title and abstract screening as it did not meet the inclusion and exclusion criteria. |
| Possible effects of voluntary exercise intensity on anxiety-like behavior and its underlying molecular mechanisms in the hippocampus: Results from a study in Hatano rats | 2022 | Asano, H and Moriya, S and Hatakeyama, T and Kobayashi, S and Akimoto, T and Ohta, R and Kawaguchi, M | Excluded | The article was excluded based on title and abstract screening as it did not meet the inclusion and exclusion criteria. |
| Voluntary exercise blocks Western diet-induced gene expression of the chemokines CXCL10 and CCL2 in the prefrontal cortex | 2016 | Carlin, JL and Grissom, N and Ying, Z and Gomez-Pinilla, F and Reyes, TM | Excluded | The article was excluded based on title and abstract screening as it did not meet the inclusion and exclusion criteria. |
| Endogenous opioid peptides stimulate post-exercise insulin response to glucose in rats | 1996 | Bouix, O and Najimi, A and Lenoir, V and Kerdelhue, B and Orsetti, A | Excluded | The article was excluded based on title and abstract screening as it did not meet the inclusion and exclusion criteria. |
| Leptin receptor knockout-induced depression-like behaviors and attenuated antidepressant effects of exercise are associated with STAT3/SOCS3 signaling | 2017 | Liu, WN and Liu, JT and Xia, J and Xue, XL and Wang, HM and Qi, ZT and Ji, L | Excluded | The article was excluded based on title and abstract screening as it did not meet the inclusion and exclusion criteria. |
| The beneficial effects of regular exercise on cognition in REM sleep deprivation: Behavioral, electrophysiological and molecular evidence | 2012 | Zagaar, M and Alhaider, I and Dao, A and Levine, A and Alkarawi, A and Alzubaidy, M and Alkadhi, K | Excluded | The article was excluded based on title and abstract screening as it did not meet the inclusion and exclusion criteria. |
| Water Maze Spatial Learning Enhances Social Recognition Ability in Aged Rats | 2012 | Zeng, Q and Han, J and Wang, B and An, S and Duan, Y and Dong, S and Ma, J and Yang, L and Cao, X | Excluded | The article was excluded based on title and abstract screening as it did not meet the inclusion and exclusion criteria. |
| Born to choose: the origins and value of the need for control | 2010 | Leotti, LA and Iyengar, SS and Ochsner, KN | Excluded | The article was excluded based on title and abstract screening as it did not meet the inclusion and exclusion criteria. |
| Transgenerational epigenetic influences of paternal environmental exposures on brain function and predisposition to psychiatric disorders | 2019 | Yeshurun, S and Hannan, AJ | Excluded | The article was excluded based on title and abstract screening as it did not meet the inclusion and exclusion criteria. |
| Inflammation increases the development of depression behaviors in male rats after spinal cord injury | 2021 | Brakel, K and Aceves, M and Garza, A and Yoo, C and Escobedo, G and Panchani, N and Shapiro, L and Hook, M | Excluded | The article was excluded based on title and abstract screening as it did not meet the inclusion and exclusion criteria. |
| GABAergic interneurons' feedback inhibition of dorsal raphe-projecting pyramidal neurons of the medial prefrontal cortex suppresses feeding of adolescent female mice undergoing activity-based anorexia | 2022 | Du, MZ and Santiago, A and Akiz, C and Aoki, C | Excluded | The article was excluded based on title and abstract screening as it did not meet the inclusion and exclusion criteria. |
| ZYKR1, a novel, potent, and peripherally selective kappa opioid receptor agonist reduces visceral pain and pruritus in animal models | 2022 | Jain, MR and Patel, RB and Prajapati, KD and Vyas, P and Bandyopadhyay, D and Prajapati, V and Bahekar, R and Patel, PN and Kawade, HM and Kokare, DM and Pawar, V and Desai, R | Excluded | The article was excluded based on title and abstract screening as it did not meet the inclusion and exclusion criteria. |
| INFLUENCE OF THE PARAVENTRICULAR NUCLEUS OF THE HYPOTHALAMUS IN THE ALTERATION OF NEUROENDOCRINE FUNCTIONS INDUCED BY INTERMITTENT FOOTSHOCK OR INTERLEUKIN | 1991 | RIVEST, S and RIVIER, C | Excluded | The article was excluded based on title and abstract screening as it did not meet the inclusion and exclusion criteria. |
| New epistemological foundations for cultural psychology: from an atomistic to a self-organizing view of living systems | 2014 | De Pascale, A | Excluded | The article was excluded based on title and abstract screening as it did not meet the inclusion and exclusion criteria. |
| Corticosterone primes the neuroinflammatory response to DFP in mice: potential animal model of Gulf War Illness | 2015 | O'Callaghan, JP and Kelly, KA and Locker, AR and Miller, DB and Lasley, SM | Excluded | The article was excluded based on title and abstract screening as it did not meet the inclusion and exclusion criteria. |
| Physiological Response of Some Economically Important Freshwater Salmonids to Catch-and-Release Fishing | 2008 | Wedemeyer, GA and Wydoski, RS | Excluded | The article was excluded based on title and abstract screening as it did not meet the inclusion and exclusion criteria. |
| Short Road Transport and Slaughter Stress Affects the Expression Profile of Serotonin Receptors, Adrenocortical, and Hematochemical Responses in Horses | 2024 | Bruschetta, G and Zanghì, G and Giunta, RP and Ferlazzo, AM and Satué, K and D'Ascola, A and Fazio, E | Excluded | The article was excluded based on title and abstract screening as it did not meet the inclusion and exclusion criteria. |
| ACTIVATION OF 5-HT1C-RECEPTORS SUPPRESSES EXCESSIVE WHEEL RUNNING INDUCED BY SEMISTARVATION IN THE RAT | 1992 | WILCKENS, T and SCHWEIGER, U and PIRKE, KM | Excluded | The article was excluded based on title and abstract screening as it did not meet the inclusion and exclusion criteria. |
| Voluntary forced use of the impaired limb following stroke facilitates functional recovery in the rat | 2014 | Livingston-Thomas, JM and McGuire, EP and Doucette, TA and Tasker, RA | Excluded | The article was excluded based on title and abstract screening as it did not meet the inclusion and exclusion criteria. |
| Mapping brain function in freely moving subjects | 2004 | Holschneider, DP and Maarek, JMI | Excluded | The article was excluded based on title and abstract screening as it did not meet the inclusion and exclusion criteria. |
| Applying ketamine to alleviate the PTSD-like effects by regulating the HCN1-related BDNF | 2018 | Hou, LW and Qi, YR and Sun, HW and Wang, G and Li, Q and Wang, YY and Zhang, ZJ and Du, ZD and Sun, L | Excluded | The article was excluded based on title and abstract screening as it did not meet the inclusion and exclusion criteria. |
| Hippocampal Neurogenesis Levels Predict WATERMAZE Search Strategies in the Aging Brain | 2013 | Gil-Mohapel, J and Brocardo, PS and Choquette, W and Gothard, R and Simpson, JM and Christie, BR | Excluded | The article was excluded based on title and abstract screening as it did not meet the inclusion and exclusion criteria. |
| Intensity of anxiety is modified via complex integrative stress circuitries | 2016 | Smith, JP and Prince, MA and Achua, JK and Robertson, JM and Anderson, RT and Ronan, PJ and Summers, CH | Excluded | The article was excluded based on title and abstract screening as it did not meet the inclusion and exclusion criteria. |
| Iron deficiency beyond erythropoiesis: should we be concerned? | 2018 | Musallam, KM and Taher, AT | Excluded | The article was excluded based on title and abstract screening as it did not meet the inclusion and exclusion criteria. |
| Reduced alcohol consumption in mice with access to a running wheel | 2009 | Ehringer, MA and Hoft, NR and Zunhammer, M | Excluded | The article was excluded based on title and abstract screening as it did not meet the inclusion and exclusion criteria. |
| CEREBROSPINAL-FLUID DYNORPHIN-CONVERTING ENZYME-ACTIVITY IS INCREASED BY VOLUNTARY EXERCISE IN THE SPONTANEOUSLY HYPERTENSIVE RAT | 1993 | PERSSON, S and JONSDOTTIR, I and THOREN, P and POST, C and NYBERG, F and HOFFMANN, P | Excluded | The article was excluded based on title and abstract screening as it did not meet the inclusion and exclusion criteria. |
| A rodent model of premenstrual dysphoria: Progesterone withdrawal induces depression-like behavior that is differentially sensitive to classes of antidepressants | 2012 | Li, Y and Pehrson, AL and Budac, DP and Sánchez, C and Gulinello, M | Excluded | The article was excluded based on title and abstract screening as it did not meet the inclusion and exclusion criteria. |
| Brain derived neurotrophic factor mediates accelerated recovery of regenerative electrical stimulation in an animal model of stress urinary incontinence | 2021 | Balog, BM and Deng, KL and Askew, T and Kuang, M and Hanzlicek, B and Damaser, MS | Excluded | The article was excluded based on title and abstract screening as it did not meet the inclusion and exclusion criteria. |
| Bi-directional regulation of AIMP2 and its splice variant on PARP-1-dependent neuronal cell death; Therapeutic implication for Parkinson's disease | 2024 | Lee, MH and Um, KH and Lee, SW and Sun, YJ and Gu, DH and Jo, YO and Kim, SH and Seol, W and Hwang, H and Baek, K and Choi, JW | Excluded | The article was excluded based on title and abstract screening as it did not meet the inclusion and exclusion criteria. |
| Periodic acceleration (pGz) prior to whole body Ischemia reperfusion injury provides early cardioprotective preconditioning | 2010 | Adams, JA and Wu, H and Bassuk, JA and Arias, J and Uryash, A and Jorapur, V and Lamas, GA and Kurlansky, P | Excluded | The article was excluded based on title and abstract screening as it did not meet the inclusion and exclusion criteria. |
| EXOGENOUS PROGESTERONE EXACERBATES RUNNING RESPONSE OF ADOLESCENT FEMALE MICE TO REPEATED FOOD RESTRICTION STRESS BY CHANGING α4-GABA<sub>A</sub> RECEPTOR ACTIVITY OF HIPPOCAMPAL PYRAMIDAL CELLS | 2015 | Wable, GS and Chen, YW and Rashid, S and Aoki, C | Excluded | The article was excluded based on title and abstract screening as it did not meet the inclusion and exclusion criteria. |
| Contribution of glia cells specifically astrocytes in the pathology of depression: immunohistochemical study in different brain areas | 2020 | Eldomiaty, MA and Makarenko, O and Hassan, ZA and Almasry, SM and Petrov, P and Elnaggar, AM | Excluded | The article was excluded based on title and abstract screening as it did not meet the inclusion and exclusion criteria. |
| THE EFFECTS OF VOLUNTARY RUNNING EXERCISE COINCIDENCE WITH SOCIAL ISOLATION AFTER EARLY WEANING ON MONOAMINERGIC AXONAL DEVELOPMENT | 2013 | Ishikawa, J and Ishikawa, A | Excluded | The article was excluded based on title and abstract screening as it did not meet the inclusion and exclusion criteria. |
| Targeting mediodorsal thalamic CB1 receptors to inhibit dextromethorphan-induced anxiety/exploratory-related behaviors in rats: The post-weaning effect of exercise and enriched environment on adulthood anxiety | 2023 | Banaei-Boroujeni, G and Rezayof, A and Alijanpour, S and Nazari-Serenjeh, F | Excluded | The article was excluded based on title and abstract screening as it did not meet the inclusion and exclusion criteria. |
| Anxiolytic, anti-nociceptive and body weight reducing effects of L-lysine in rats: Relationship with brain serotonin an <i>In-Vivo</i> and <i>In-Silico</i> study | 2022 | Jalal, K and Khan, F and Nawaz, S and Afroz, R and Khan, K and Ali, SB and Hao, LL and Khan, SA and Kazi, M and Uddin, R and Haleem, DJ | Excluded | The article was excluded based on title and abstract screening as it did not meet the inclusion and exclusion criteria. |
| Effect of orally administered L-tryptophan on serotonin, melatonin, and the innate immune response in the rat | 2004 | Esteban, S and Nicolaus, C and Garmundi, A and Rial, REV and Rodríguez, AB and Ortega, E and Ibars, CB | Excluded | The article was excluded based on title and abstract screening as it did not meet the inclusion and exclusion criteria. |
| Neurobiological Consequences of Sleep Deprivation | 2013 | Alkadhi, K and Zagaar, M and Alhaider, I and Salim, S and Aleisa, A | Excluded | The article was excluded based on title and abstract screening as it did not meet the inclusion and exclusion criteria. |
| Neurohypophyseal implications in psychological stress | 2001 | Scantamburlo, G and Ansseau, M and Legros, JJ | Excluded | The article was excluded based on title and abstract screening as it did not meet the inclusion and exclusion criteria. |
| S-Ketamine Rapidly Reverses Synaptic and Vascular Deficits of Hippocampus in Genetic Animal Model of Depression | 2017 | Ardalan, M and Wegener, G and Rafati, AH and Nyengaard, JR | Excluded | The article was excluded based on title and abstract screening as it did not meet the inclusion and exclusion criteria. |
| Melatonin treatment improves cognitive deficits by altering inflammatory and neurotrophic factors in the hippocampus of obese mice | 2022 | Mansouri, S and Salari, AA and Abedi, A and Mohammadi, P and Amani, M | Excluded | The article was excluded based on title and abstract screening as it did not meet the inclusion and exclusion criteria. |
| The effect of early handling on anxiety-like behaviors of rats exposed to valproic acid pre-and post-natally | 2022 | Shafaghi, A and Shahrbabaki, SSV and Aminzadeh, A and Heidari, MR and Meymandi, MS and Bashiri, H | Excluded | The article was excluded based on title and abstract screening as it did not meet the inclusion and exclusion criteria. |
| Evidence that the bed nucleus of the stria terminalis contributes to the modulation of hypophysiotropic corticotropin-releasing factor cell responses to systemic interleukin-1β | 2003 | Crane, JW and Buller, KM and Day, TA | Excluded | The article was excluded based on title and abstract screening as it did not meet the inclusion and exclusion criteria. |
| Neuropeptides in depression: Role of VGF | 2009 | Thakker-Varia, S and Alder, J | Excluded | The article was excluded based on title and abstract screening as it did not meet the inclusion and exclusion criteria. |
| Characterization of <SUP>11</SUP>C-GSK1482160 for Targeting the P2X7 Receptor as a Biomarker for Neuroinflammation | 2017 | Territo, PR and Meyer, JA and Peters, JS and Riley, AA and McCarthy, BP and Gao, MZ and Wang, M and Green, MA and Zheng, QH and Hutchins, GD | Excluded | The article was excluded based on title and abstract screening as it did not meet the inclusion and exclusion criteria. |
| Environmental Enrichment Increased<i> Bdnf</i> Transcripts in the Prefrontal Cortex: Implications for an Epigenetically Controlled Mechanism | 2023 | Costa, GA and Silva, NKDT and Marianno, P and Chivers, P and Bailey, A and Camarini, R | Excluded | The article was excluded based on title and abstract screening as it did not meet the inclusion and exclusion criteria. |
| Exercise activates compensatory thermoregulatory reaction in rats: a modeling study | 2015 | Yoo, Y and LaPradd, M and Kline, H and Zaretskaia, MV and Behrouzvaziri, A and Rusyniak, DE and Molkov, YI and Zaretsky, DV | Excluded | The article was excluded based on title and abstract screening as it did not meet the inclusion and exclusion criteria. |
| Alzheimer's disease and type 2 diabetes mellitus: similarities in pathomechanisms lead to therapeutic opportunities | 2021 | Bednarz, K and Siuda, J | Excluded | The article was excluded based on title and abstract screening as it did not meet the inclusion and exclusion criteria. |
| Maternal exposure to fluoxetine during gestation and lactation affects the DNA methylation programming of rat's offspring: Modulation by folic acid supplementation | 2014 | Toffoli, LV and Rodrigues, GM and Oliveira, JF and Silva, AS and Moreira, EG and Pelosi, GG and Gomes, MV | Excluded | The article was excluded based on title and abstract screening as it did not meet the inclusion and exclusion criteria. |
| BLOOD-FLOW TO RESPIRATORY MUSCLES AND MAJOR ORGANS DURING INSPIRATORY FLOW RESISTIVE LOADS | 1993 | PANG, LM and KIM, YJ and BAZZY, AR | Excluded | The article was excluded based on title and abstract screening as it did not meet the inclusion and exclusion criteria. |
| Transcriptome Analysis on Maternal Separation Rats With Depression-Related Manifestations Ameliorated by Electroacupuncture | 2019 | Zheng, YJ and He, J and Guo, LL and Yao, L and Zheng, XR and Yang, ZH and Xia, YC and Wu, XL and Su, Y and Xu, NG and Chen, YJ | Excluded | The article was excluded based on title and abstract screening as it did not meet the inclusion and exclusion criteria. |
| Effects of on-farm hatching on short term stress indicators, weight gain, and cognitive ability in layer chicks | 2022 | Witjes, VL and Bruckmaier, RM and Gebhardt-Henrich, SG and Toscano, MJ | Excluded | The article was excluded based on title and abstract screening as it did not meet the inclusion and exclusion criteria. |
| Too little sleep gradually desensitizes the serotonin 1A receptor system | 2005 | Roman, V and Walstra, I and Luiten, PGM and Meerlo, P | Excluded | The article was excluded based on title and abstract screening as it did not meet the inclusion and exclusion criteria. |
| Efficacy of a growth hormone-releasing hormone agonist in a murine model of cardiometabolic heart failure with preserved ejection fraction | 2023 | Kanashiro-Takeuchi, RM and Takeuchi, LM and Dulce, RA and Kazmierczak, K and Balkan, W and Cai, RZ and Sha, W and Schally, AV and Hare, JM | Excluded | The article was excluded based on title and abstract screening as it did not meet the inclusion and exclusion criteria. |
| A canine <i>BCAN</i> microdeletion associated with episodic falling syndrome | 2012 | Gill, JL and Tsai, KL and Krey, C and Noorai, RE and Vanbellinghen, JF and Garosi, LS and Shelton, GD and Clark, LA and Harvey, RJ | Excluded | The article was excluded based on title and abstract screening as it did not meet the inclusion and exclusion criteria. |
| Adult neurogenesis and schizophrenia: is there a link? | 2007 | Reif, A and Schmitt, A and Fritzen, S and Lesch, KP | Excluded | The article was excluded based on title and abstract screening as it did not meet the inclusion and exclusion criteria. |
| Running Wild: Neurogenesis in the Hippocampus Across the Lifespan in Wild and Laboratory-Bred Norway Rats | 2009 | Epp, JR and Barker, JM and Galea, LAM | Excluded | The article was excluded based on title and abstract screening as it did not meet the inclusion and exclusion criteria. |
| The comparative distributions of the monoamine transporters in the rodent, monkey, and human amygdala | 2008 | Smith, HR and Porrino, LJ | Excluded | The article was excluded based on title and abstract screening as it did not meet the inclusion and exclusion criteria. |
| Environmental Enrichment Modulates Drug Addiction and Binge-Like Consumption of Highly Rewarding Substances: A Role for Anxiety and Compulsivity Brain Systems? | 2018 | Rodríguez-Ortega, E and Cubero, I | Excluded | The article was excluded based on title and abstract screening as it did not meet the inclusion and exclusion criteria. |
| Role of Social Encounter-Induced Activation of Prefrontal Serotonergic Systems in the Abnormal Behaviors of Isolation-Reared Mice | 2013 | Ago, Y and Araki, R and Tanaka, T and Sasaga, A and Nishiyama, S and Takuma, K and Matsuda, T | Excluded | The article was excluded based on title and abstract screening as it did not meet the inclusion and exclusion criteria. |
| Modulation of mitochondrial phenotypes by endurance exercise contributes to neuroprotection against a MPTP-induced animal model of PD | 2018 | Jang, Y and Kwon, I and Song, W and Cosio-Lima, LM and Taylor, S and Lee, Y | Excluded | The article was excluded based on title and abstract screening as it did not meet the inclusion and exclusion criteria. |
| What Goes Around Can Come Around: An Unexpected Deleterious Effect of Using Mouse Running Wheels for Environmental Enrichment | 2017 | Leduc, RYM and Rauw, G and Baker, GB and McDermid, HE | Excluded | The article was excluded based on title and abstract screening as it did not meet the inclusion and exclusion criteria. |
| The new neuron formation in the adult hippocampus:: Neurogenesis | 2007 | Ramirez-Rodríguez, G and Benítez-King, G and Kempermann, G | Excluded | The article was excluded based on title and abstract screening as it did not meet the inclusion and exclusion criteria. |
| Cell Proliferation in the Adult Chicken Hippocampus Correlates With Individual Differences in Time Spent in Outdoor Areas and Tonic Immobility | 2020 | Armstrong, EA and Voelkl, B and Voegeli, S and Gebhardt-Henrich, SG and Guy, JH and Sandilands, V and Boswell, T and Toscano, MJ and Smulders, TV | Excluded | The article was excluded based on title and abstract screening as it did not meet the inclusion and exclusion criteria. |
| Insulin-Like Growth Factor I Produces an Antidepressant-Like Effect and Elicits N-MethylD-Aspartate Receptor Independent Long-Term Potentiation of Synaptic Transmission in Medial Prefrontal Cortex and Hippocampus | 2016 | Burgdorf, J and Zhang, XL and Colechio, EM and Ghoreishi-Haack, N and Gross, A and Kroes, RA and Stanton, PK and Moskal, JR | Excluded | The article was excluded based on title and abstract screening as it did not meet the inclusion and exclusion criteria. |
| Neuroprotective strategies for retinal disease | 2018 | Pardue, MT and Allen, RS | Excluded | The article was excluded based on title and abstract screening as it did not meet the inclusion and exclusion criteria. |
| Duration- and environment-dependent effects of repeated voluntary exercise on anxiety and cued fear in mice | 2015 | Dubreucq, S and Marsicano, G and Chaouloff, F | Excluded | The article was excluded based on title and abstract screening as it did not meet the inclusion and exclusion criteria. |
| AMPK Signalling and Defective Energy Metabolism in Amyotrophic Lateral Sclerosis | 2016 | Perera, ND and Turner, BJ | Excluded | The article was excluded based on title and abstract screening as it did not meet the inclusion and exclusion criteria. |
| <i>In vitro</i> opioid receptor affinity and <i>in vivo</i> behavioral studies of <i>Nelumbo nucifera</i> flower | 2015 | Kumarihamy, M and Leon, F and Pettaway, S and Wilson, L and Lambert, JA and Wang, M and Hill, C and McCurdy, CR and ElSohly, MA and Cutler, SJ and Muhammad, I | Excluded | The article was excluded based on title and abstract screening as it did not meet the inclusion and exclusion criteria. |
| Seasonal differences in the morphology and spine density of hippocampal neurons in wild ground squirrels | 2022 | Brinkman, B and Ngwenya, A and Fjordbotten, K and Stephen, O and Iwaniuk, AN | Excluded | The article was excluded based on title and abstract screening as it did not meet the inclusion and exclusion criteria. |
| The effect of abscisic acid chronic treatment on neuroinflammatory markers and memory in a rat model of high-fat diet induced neuroinflammation | 2016 | Sánchez-Sarasúa, S and Moustafa, S and García-Avilés, A and López-Climent, MF and Gómez-Cadenas, A and Olucha-Bordonau, FE and Sánchez-Pérez, AM | Excluded | The article was excluded based on title and abstract screening as it did not meet the inclusion and exclusion criteria. |
| Failure of ATP supply to match ATP demand: The mechanism of toxicity of the lampricide, 3-trifluoromethyl-4-nitrophenol (TFM), used to control sea lamprey (<i>Petromyzon marinus</i>) populations in the Great Lakes | 2009 | Birceanu, O and McClelland, GB and Wang, YXS and Wilkie, MP | Excluded | The article was excluded based on title and abstract screening as it did not meet the inclusion and exclusion criteria. |
| Rapid eye movement sleep deprivation contributes to reduction of neurogenesis in the hippocampal dentate gyrus of the adult rat | 2008 | Guzman-Marin, R and Suntsova, N and Bashir, T and Nienhuis, R and Szymusiak, R and McGinty, D | Excluded | The article was excluded based on title and abstract screening as it did not meet the inclusion and exclusion criteria. |
| Neuropeptides and obesity | 2000 | Beck, B | Excluded | The article was excluded based on title and abstract screening as it did not meet the inclusion and exclusion criteria. |
| Pretreatment with combined low-level laser therapy and methylene blue improves learning and memory in sleep-deprived mice | 2022 | Marzabadi, EA and Meftahi, GH and Refahi, S | Excluded | The article was excluded based on title and abstract screening as it did not meet the inclusion and exclusion criteria. |
| Evidence-based practice use of quick-release bromocriptine across the natural history of type 2 diabetes mellitus | 2016 | Schwartz, SS and Zangeneh, F | Excluded | The article was excluded based on title and abstract screening as it did not meet the inclusion and exclusion criteria. |
| Brain, drugs, its neurobiological mechanisms | 2010 | Díaz, MM and Contreras, AER and Gómez, BP and Romano, A and Caynas, S and García, OP | Excluded | The article was excluded based on title and abstract screening as it did not meet the inclusion and exclusion criteria. |
| THERMOREGULATION IN LABORATORY MAMMALS AND HUMANS EXPOSED TO ANTICHOLINESTERASE AGENTS | 1994 | GORDON, CJ | Excluded | The article was excluded based on title and abstract screening as it did not meet the inclusion and exclusion criteria. |
| Physiological and behavioural responses in blue foxes (<i>Alopex lagopus</i>):: comparisons between space quantity and floor material | 2001 | Korhonen, H and Jauhiainen, L and Niemelä, P and Harri, M and Sauna-aho, R | Excluded | The article was excluded based on title and abstract screening as it did not meet the inclusion and exclusion criteria. |
| Depression of cortical activity in humans by mild hypercapnia | 2012 | Thesen, T and Leontiev, O and Song, T and Dehghani, N and Hagler, DJ and Huang, MX and Buxton, R and Halgren, E | Excluded | The article was excluded based on title and abstract screening as it did not meet the inclusion and exclusion criteria. |
| Neurotransmitters of the limbic system.: Hippocampus.: GABA and memory.: Part one. | 2007 | Castro-Sierra, E and de León, FCP and Domínguez, LFG and Rivera, AP | Excluded | The article was excluded based on title and abstract screening as it did not meet the inclusion and exclusion criteria. |
| The Fibre Requirements of Horses and the Consequences and Causes of Failure to Meet Them | 2023 | Ermers, C and McGilchrist, N and Fenner, K and Wilson, B and McGreevy, P | Excluded | The article was excluded based on title and abstract screening as it did not meet the inclusion and exclusion criteria. |
| NOTES OF BRITISH ARCHITECTURAL CULTURE 1945-1965 | 2012 | Frampton, K | Excluded | The article was excluded based on title and abstract screening as it did not meet the inclusion and exclusion criteria. |
