# Supplementary material for: Biochemical neuroplasticity in the cerebellum after physical exercise: Systematic review and meta-analysis
Source: PLoS One. 2025 Aug 1;20(8):e0309259. doi: 10.1371/journal.pone.0309259 (PMC12316286; doi:10.1371/journal.pone.0309259)
Supplement: S6 File — (DOCX) [file pone.0309259.s006.docx]

***Supplementary Material 3***

BIOCHEMICAL NEUROPLASTICITY IN THE CEREBELLUM AFTER PHYSICAL EXERCISE: SYSTEMATIC REVIEW AND META-ANALYSIS

Marcio Gonçalves Corrêa ^1^ ^¶^, Thais Alves Lobão ^1^ ^¶^, Gabriel Mesquita da Conceição Bahia^1^, Erica Miranda Sanches Aires ^1^, Rebeca da Costa Gomes^1*^, Jeffeson Hildo Medeiros de Queiroz^1^, Marta Chagas Monteiro^2^, Carlomagno Pacheco Bahia^1*^

**Correspondence:** Corresponding Author: [carlomagno@ufpa.br](mailto:carlomagno@ufpa.br) or [carlomagnobahia@gmail.com](mailto:carlomagnobahia@gmail.com)

**Publication bias quantitative analysis - Funnel plot**

**
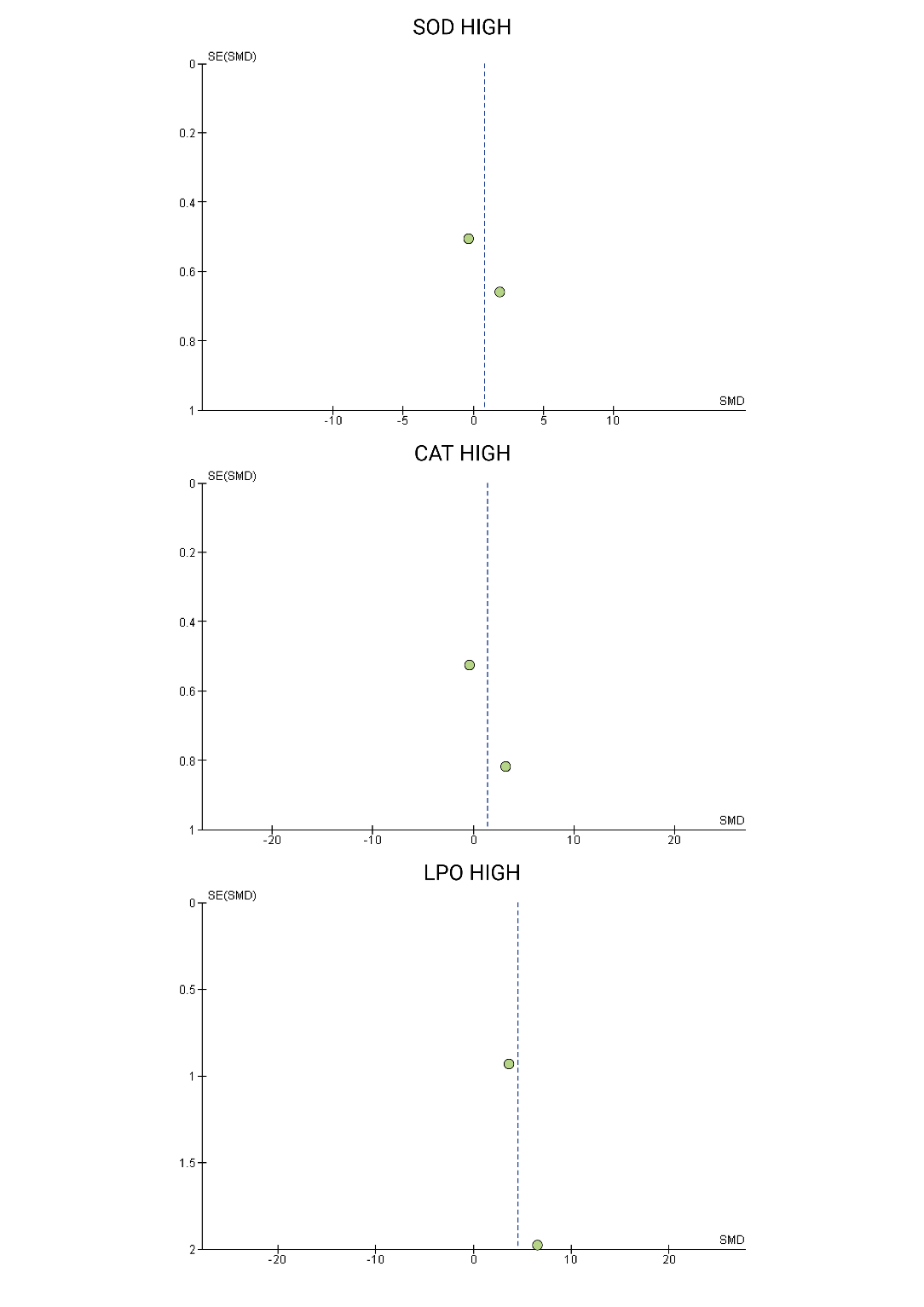
**

**Figure 1 :** Control versus Phisycal Exercice in high volume

**
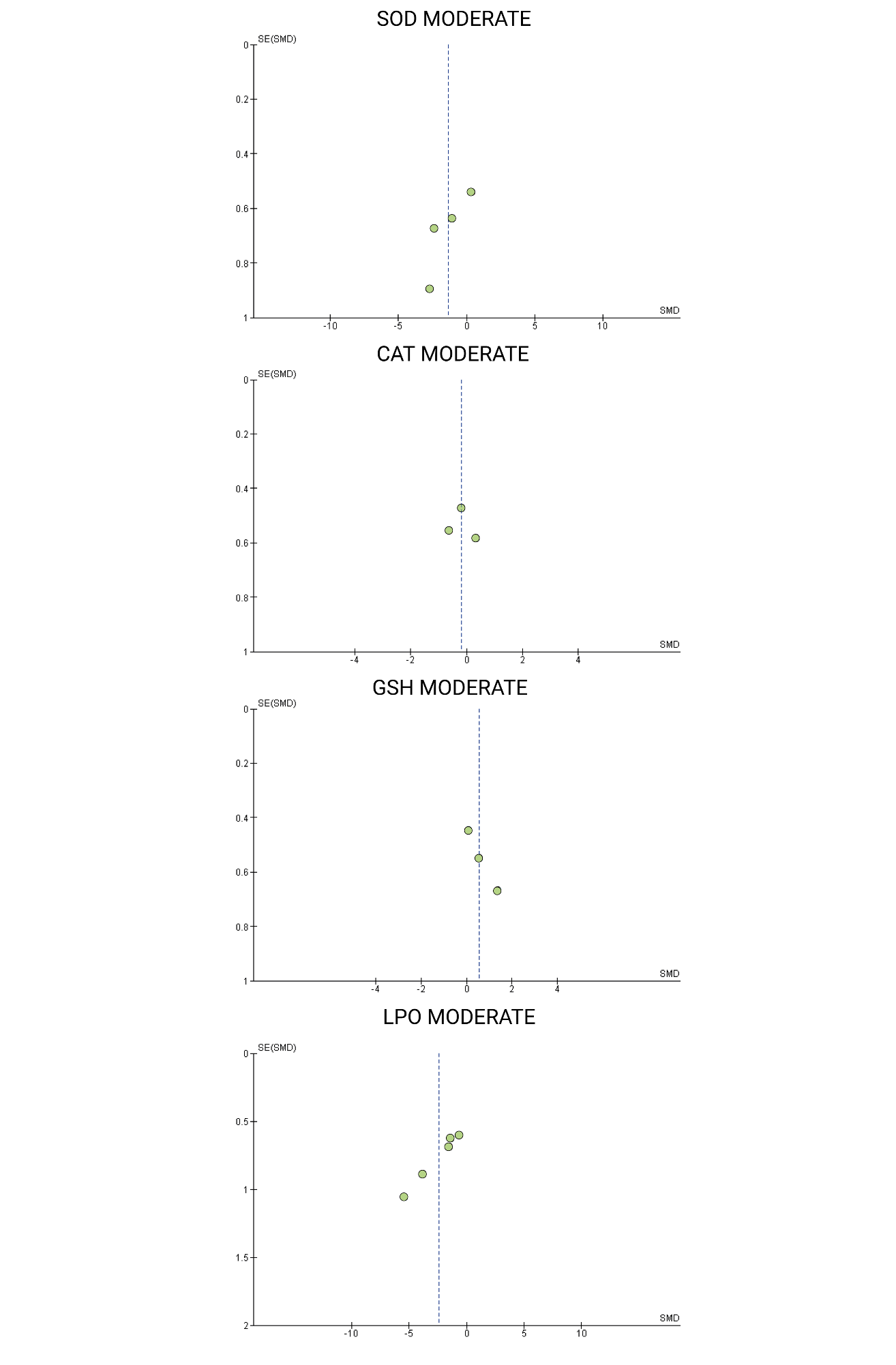
**

**Figure 2 :** Control versus Phisycal Exercice in moderate volume
