# Supplementary material for: Biochemical neuroplasticity in the cerebellum after physical exercise: Systematic review and meta-analysis
Source: PLoS One. 2025 Aug 1;20(8):e0309259. doi: 10.1371/journal.pone.0309259 (PMC12316286; doi:10.1371/journal.pone.0309259)
Supplement: Attachment — Submitted filename: PONE-D-24-33520 JAN.docx [file pone.0309259.s008.docx]

The reviewer comments for the above **Manuscript Number:** PONE-D-24-33520_R1
**Manuscript Title:** BIOCHEMICAL NEUROPLASTICITY IN THE CEREBELLUM AFTER PHYSICAL EXERCISE: SYSTEMATIC REVIEW AND META-ANALYSIS

The manuscript presents a study on cerebellar biochemical neuroplasticity, focusing on the role of calcium ions and high-intensity physical exercise. However, it is noted that the study was excluded from studies evaluating these factors, which could be addressed in future iterations.

- In study selection you had written “Among 3,107 articles, 767 were duplicates and 2,340 were excluded after the title”. If you see in Figure values are mentioned as 3.107, 2.340 please correct in figure as 3,107 and 2,340.
- Can you justify the sentence “2,086 articles were eligible for qualitative and quantitative analyses.” What is the number 2,086 found in abstract part please specify it.
- In selection criteria if you would have mention certain time period it will good as my point of view.
- In funnel plot diagram looks fine, but please highlight the plot area by using shape fill option in word document, for better view.
- In abbreviation section you mentioned as “PE Physical exercise group” in some areas found it as “PE group” clarify and modify accordingly.
- The discussion depth on adaptive versus maladaptive neuroplasticity mechanisms is well-emphasized, but more emphasis on real-world applications or translating these findings into human models would strengthen the relevance of the research.
- In my view the references would be recent if possible.
- The study also discusses the heterogeneity of the included studies, which could be addressed in greater depth.
- The study also discusses the high heterogeneity in some metrics, which reduces interpretability and could be improved by discussing the reasons behind this higher variability.
- The visual clarity of the figures could be improved, and the supplementary material could be streamlined for key elements.
