# Supplementary material for: Biochemical neuroplasticity in the cerebellum after physical exercise: Systematic review and meta-analysis
Source: PLoS One. 2025 Aug 1;20(8):e0309259. doi: 10.1371/journal.pone.0309259 (PMC12316286; doi:10.1371/journal.pone.0309259)
Supplement: Attachment — Submitted filename: Comments.docx [file pone.0309259.s010.docx]

- Please check for grammatical and language errors.
- Please review the article thoroughly the title in supplementary material 1 it was showing as “BIOCHEMISTRY NEURAL PLASTICITY IN THE CEREBELLUM PROMOTED BY PHYSICAL EXERCISE: A SYSTEMATIC REVIEW AND METANALYSIS” but the main title is BIOCHEMICAL NEUROPLASTICITY can you please justify it.
- Can you please define what is “biochemistry changes”
- The forest plots are so ambiguous not visible properly
- The methodology section needs to be refined
- Please make sure the abbreviations are correctly defined PE (I)
- Please mention the section uniformly throughout the manuscript “Six out of the 2,340 articles” or “Among 3,107 articles, 767 duplicates and 2,330 articles did not meet the eligibility criteria 172 after title and abstract reading.” Recheck and write in a uniform manner.
- Where is the Risk of Bias diagram?
