# Supplementary material for: Biochemical neuroplasticity in the cerebellum after physical exercise: Systematic review and meta-analysis
Source: PLoS One. 2025 Aug 1;20(8):e0309259. doi: 10.1371/journal.pone.0309259 (PMC12316286; doi:10.1371/journal.pone.0309259)
Supplement: Attachment — Submitted filename: Response to Reviewers.docx [file pone.0309259.s011.docx]

The manuscript: PONE-D-24-33520_R1 under the Title: **“BIOCHEMICAL NEUROPLASTICITY IN THE CEREBELLUM AFTER PHYSICAL EXERCISE: SYSTEMATIC REVIEW AND META-ANALYSIS”**.

**Response to reviewers' comments:**

- Please check for grammatical and language errors.

**Authors' response:** A grammatical analysis was performed across all text to verify language errors.

- Please review the article thoroughly the title in supplementary material 1 it was showing as “BIOCHEMISTRY NEURAL PLASTICITY IN THE CEREBELLUM PROMOTED BY PHYSICAL EXERCISE: A SYSTEMATIC REVIEW AND METANALYSIS” but the main title is BIOCHEMICAL NEUROPLASTICITY can you please justify it.

**Authors' response:** The title of supplementary material 1 has been corrected to **“BIOCHEMICAL NEUROPLASTICITY IN THE CEREBELLUM AFTER PHYSICAL EXERCISE: SYSTEMATIC REVIEW AND META-ANALYSIS”.**

- Can you please define what is “biochemistry changes”

**Authors' response:** We have chosen to revise the summary and no longer use the term "biochemistry changes." Note, we have reworded the abstract to the Plos One format.

- The forest plots are so ambiguous not visible properly.

**Authors' response:** We improved the visibility and resolution of the Forest plots, including increasing the size of all graphs. Furthermore, all the images (figures) were updated to the Pace Correct, the Plos One system.

- The methodology section needs to be refined

**Authors' response:** The methods section has been refined according to the journal's standards.

- Please make sure the abbreviations are correctly defined PE (I)

**Authors' response:** The abbreviation was corrected.

- Please mention the section uniformly throughout the manuscript “Six out of the 2,340 articles” or “Among 3,107 articles, 767 duplicates and 2,330 articles did not meet the eligibility criteria 172 after title and abstract reading.” Recheck and write in a uniform manner.

**Authors' response:** The writing has been standardized throughout the entire text.

- Where is the Risk of Bias diagram?

**Authors' response:** The figures have been added as requested.

Note: All changes are yellow highlighted throughout the text.
