# Supplementary material for: Biochemical neuroplasticity in the cerebellum after physical exercise: Systematic review and meta-analysis
Source: PLoS One. 2025 Aug 1;20(8):e0309259. doi: 10.1371/journal.pone.0309259 (PMC12316286; doi:10.1371/journal.pone.0309259)
Supplement: Attachment — Submitted filename: PONE-D-24-33520_R3.docx [file pone.0309259.s012.docx]

PONE-D-24-33520_R3

**Reviewer comments**

Title: BIOCHEMICAL NEUROPLASTICITY IN THE CEREBELLUM AFTER PHYSICAL

EXERCISE: SYSTEMATIC REVIEW AND META-ANALYSIS

The manuscript discusses the topic of how physical exercise (PE) modulates cerebellar biochemical neuroplasticity, offering valuable insights to neuroscience, sports science, and clinical rehabilitation. The systematic review and meta-analysis add rigor to the topic, providing insights valuable to these domains. The article provides an original synthesis of literature, identifying both adaptive and maladaptive responses of the cerebellum to varying PE protocols.

However, there are some minor comments. The operational definition of "Biochemical Neuroplasticity" is often used frequently and broadly, and the authors acknowledge heterogeneity in outcomes like SOD and LPO but do not explore sources of this heterogeneity justify. A subgroup or sensitivity analysis could strengthen the findings.

The manuscript draws conclusions about adaptive vs. maladaptive effects based on the direction of LPO and enzyme activity. However, some conclusions in the discussion are not always statistically significant in the meta-analysis. Clarifying this discrepancy and avoiding overgeneralization are recommended.

Visual quality of figures should be improved, with axis labels, subgroup definitions, and confidence intervals legible. Abbreviation consistency should be ensured, and the data extraction table should be simplified for clarity. Risk of bias visuals should be clearly referred to in figure captions and brief interpretation of findings.

Mechanistic pathways should be well-annotated and discussed in-text, and references should be formatted according to PLOS ONE guidelines.
